# Peptidomimetic plasmepsin inhibitors with potent anti-malarial activity and selectivity against cathepsin D

**DOI:** 10.1016/j.ejmech.2018.11.068

**Published:** 2019-02-01

**Authors:** Rimants Zogota, Linda Kinena, Chrislaine Withers-Martinez, Michael J. Blackman, Raitis Bobrovs, Teodors Pantelejevs, Iveta Kanepe-Lapsa, Vita Ozola, Kristaps Jaudzems, Edgars Suna, Aigars Jirgensons

**Affiliations:** a Latvian Institute of Organic Synthesis, Aizkraukles 21, Riga, LV, 1006, Latvia; b Malaria Biochemistry Laboratory, The Francis Crick Institute, 1 Midland Road, London, NW1 1AT, UK; c Faculty of Infectious and Tropical Diseases, London School of Hygiene & Tropical Medicine, London, WC1E 7HT, UK

**Keywords:** Plasmepsins, Malaria, *Plasmodium falciparum*, Cathepsin D, Inhibitors, Hydroxyethylamine

## Abstract

Following up the open initiative of anti-malarial drug discovery, a GlaxoSmithKline (GSK) phenotypic screening hit was developed to generate hydroxyethylamine based plasmepsin (Plm) inhibitors exhibiting growth inhibition of the malaria parasite *Plasmodium falciparum* at nanomolar concentrations. Lead optimization studies were performed with the aim of improving Plm inhibition selectivity *versus* the related human aspartic protease cathepsin D (Cat D). Optimization studies were performed using Plm IV as a readily accessible model protein, the inhibition of which correlates with anti-malarial activity. Guided by sequence alignment of Plms and Cat D, selectivity-inducing structural motifs were modified in the S3 and S4 sub-pocket occupying substituents of the hydroxyethylamine inhibitors. This resulted in potent anti-malarials with an up to 50-fold Plm IV/Cat D selectivity factor. More detailed investigation of the mechanism of action of the selected compounds revealed that they inhibit maturation of the *P. falciparum* subtilisin-like protease SUB1, and also inhibit parasite egress from erythrocytes. Our results indicate that the anti-malarial activity of the compounds is linked to inhibition of the SUB1 maturase plasmepsin subtype Plm X.

## Highlights

• Peptidomimimetic plasmepsin inhibitors are developed using Plm IV as a model enzyme.
• Up to 50-fold selectivity against Cathepsin D is reached.
• Compounds show growth inhibition of *P. falciparum* at nanomolar concentrations.
• Inhibition of SUB1 maturation and parasite egress imply (co)inhibition of Plm X.

## Graphical abstract

**Figure:**
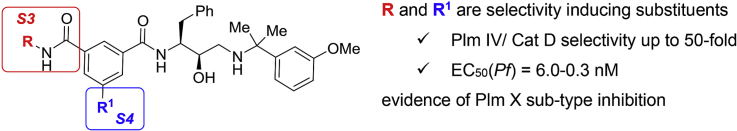

## 1 Introduction

Malaria is a life-threatening disease caused by *Plasmodium* parasites which are transmitted by mosquitoes [1]. More than half of the earth's population lives in malaria endemic areas, rendering the disease a global health problem. Extensive eradication campaigns have been implemented, leading to considerably reduced malaria morbidity [2]. A key future goal, according to the Global Technical Strategy for Malaria 2016–2030, is a 90% reduction in clinical cases and deaths by 2030 as compared with 2015 [3]. However, these efforts are impeded by widespread resistance of the parasite to all currently used drugs, including artemisinins, the current front line drug class [[4], [5], [6]]. Consequently, new antimalarial drugs with new modes of action are urgently needed. Their development faces notable hurdles, one of which is a low expected profit after market approval. This has prompted several open innovation initiatives by private and academic organizations, including the disclosure of preclinical research data to the scientific community [[7], [8], [9], [10]]. To support one such initiative, GlaxoSmithKline (GSK) recently published the results of a large-scale cell-based (phenotypic) HTS screening campaign that provided a number of starting points for anti-malarial drug discovery [7]. From the pool of parasite growth inhibitory compounds we selected hydroxyethylamine derivative **1a** for further development (Table 1) [11]. In our previous studies we showed that compound **1a** is an inhibitor of the *Plasmodium falciparum* aspartic proteases - plasmepsin subtypes Plm I, Plm II and Plm IV with particularly high potency against Plm IV. Structurally simplified potent Plm IV inhibitors **1b,c** were developed as compound **1a** analogues, retaining high potency in *P. falciparum* growth assays (see Table 1).

**Table 1:**
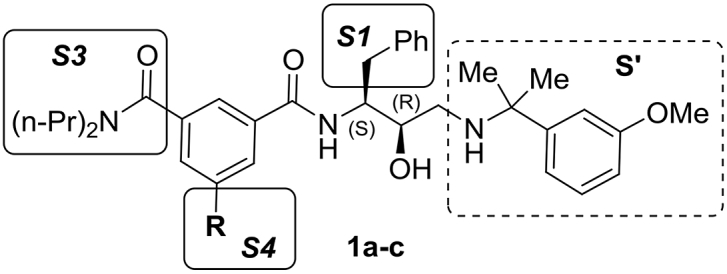
Representative Plm inhibitors **1a-c** from previous studies [11]

Comp.	R	IC_50_ Plm IV, μΜ	IC_50_ Cat D, μΜ	EC_50_ *Pf* growth, μΜ
(*S*,*R*)-**1a**		0.029	0.043	0.002
(*S*,*R*)-**1b**		0.024	0.042	0.006
(*S*,*R*)-**1c**	Ph	0.006	0.054	0.002

It is important to note that despite more than two decades of research on plasmepsin inhibitor discovery, only a few compounds (including inhibitors **1a-c**) exhibiting parasite growth inhibition at low nanomolar concentration have been identified [11,12]. This is likely attributable to the fact that most of the efforts so far have been devoted to inhibiting plasmepsin subtypes involved in hemoglobin digestion (Plms I-IV) [[13], [14], [15], [16], [17], [18], [19], [20], [21], [22]]. Gene disruption studies have revealed that none of these hemoglobinase Plms are essential in the parasite asexual blood stage lifecycle, indicating a high degree of redundancy in the hemoglobin catabolic pathway [[23], [24], [25]]. In contrast, the three other plasmepsin subtypes expressed in the *P. falciparum* blood stages, Plms V [[26], [27], [28]], IX, and X [[29], [30], [31], [32]], all appear to be essential for parasite viability. The hemoglobinase plasmepsins (Plm I, II, IV) share high sequence homology with Plms IX and X, but not Plm V. It might therefore be expected that inhibitors developed to target the hemoglobinase Plms would exhibit activity in cell-based assays only if they additionally target Plms IX and/or Plm X. Recombinant expression of both Plms IX and X has been recently reported [29,30], but this could be achieved only in higher eukaryotic protein expression systems, such as insect or mammalian cells. For our further work to develop the hydroxyethylamine based inhibitors (*S*,*R*)-**1a-c** as anti-malarials we therefore used Plm IV as a readily accessible model plasmepsin, an approach also supported by the previously observed good correlation between inhibition of this enzyme and potency in parasite growth assays in erythrocytes [11,20].

The successful development of protease inhibitors as drugs requires optimization of on-target potency and minimization of undesirable off-target activity, particularly against related host proteases. The human lysosomal aspartic protease cathepsin D (Cat D) plays critical roles in protein catabolism and retinal function [33]. Recent work focused on development of inhibitors of the human aspartic protease β-secretase (BACE1) revealed the importance of ensuring selectivity against Cat D in order to avoid off-target ocular toxicity [34,35]. In view of this, we decided that the next step for optimization of the hydroxyethylamine based Plm inhibitors **1** should aim to improve their selectivity for their malarial target(s) over human Cat D.

## 2 Results and discussion

### 2.1 Structural factors determining the selectivity of inhibitor binding to Plms *vs* Cat D

**Fig. 1:**
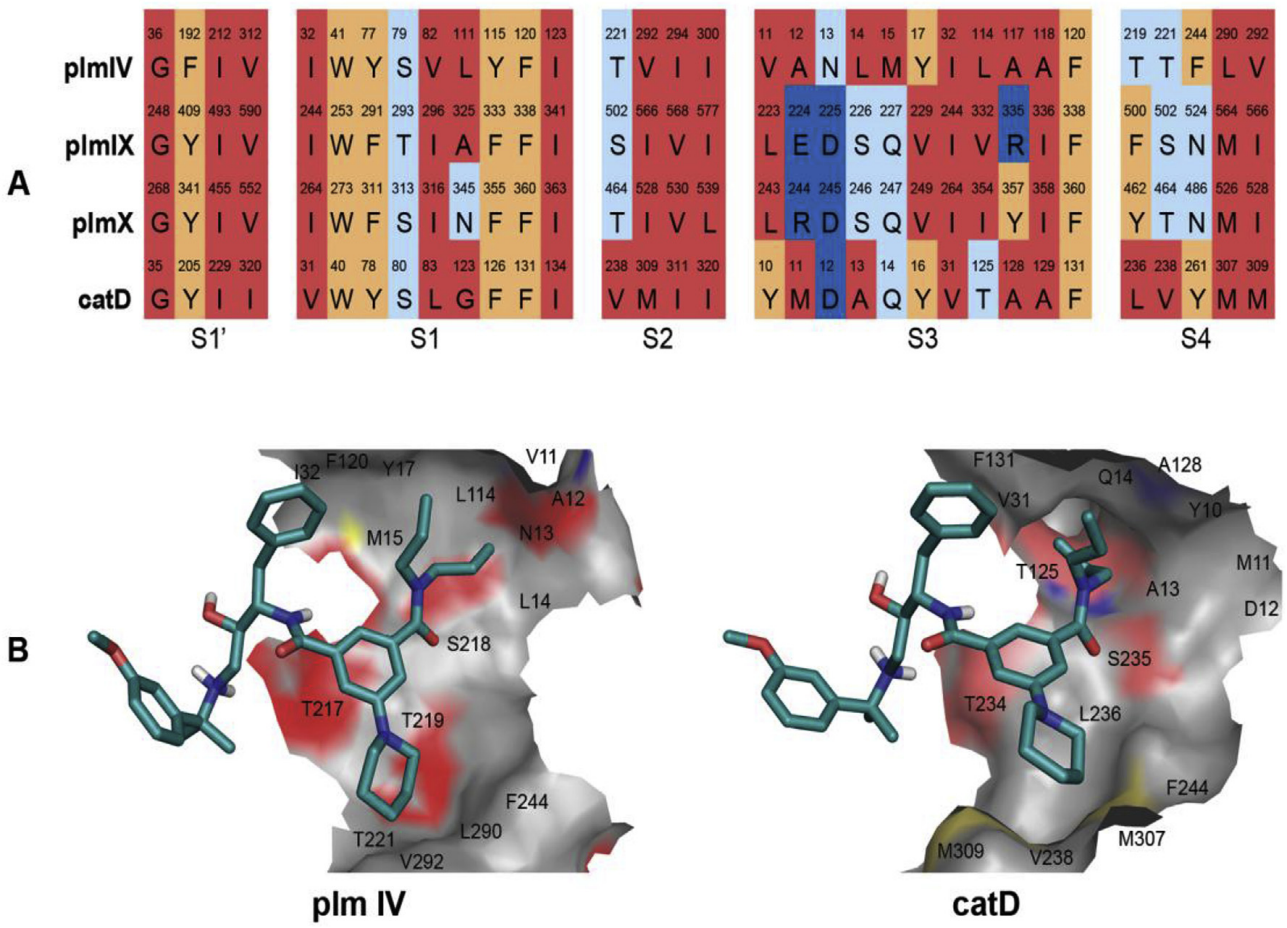
(A) Structure-based sequence alignment of the amino acid residues making up the S1′ and S1—S4 pockets of Plm IV, Plm IX, Plm X and Cat D. (B) Surface representation of the S3 and S4 pockets in Plm IV and Cat D docking models with the hydroxyethylamine based inhibitor (*S*,*R*)-**1b**. The figure was prepared in PyMol [37]. Surface oxygen atoms are colored in red, nitrogens in blue, sulfurs in yellow and hydrogens and carbons in grey. (For interpretation of the references to color in this figure legend, the reader is referred to the Web version of this article.)

Our previous SAR investigations revealed that the substituents of the inhibitor (*S*,*R*)-**1** occupying the prime sub-pockets (part S’, Table 1) are optimal for Plm inhibition [11]. Therefore, we focused our efforts on optimisation of selectivity inducing motifs in the substituents occupying the non-prime sub-pockets (Table 1). To assess the differences in inhibitor recognition between Plms IV, IX, X and Cat D, we generated a structure-based sequence alignment of these proteins and compared their interactions with inhibitor **1b** in docking models (Fig. 1). Since Plm IX and X lack experimentally determined structures and in order to avoid possible inaccuracies associated with the use of homology models, the docking studies were performed on the crystal structure of Plm IV (PDB ID 2ANL), which is the closest homologue with an available crystal structure [36]. As can be seen from Fig. 1A, the S3 sub-pocket shows the largest differences in amino acid composition between the Plms and Cat D. Additionally, this revealed that the S3 sub-pocket of Plm IV is wider, more shallow and more hydrophobic than that of Cat D (Fig. 1B). For these reasons, selectivity improvement was first attempted by modifying the *N,N*-dipropylamide moiety in inhibitors (*S*,*R*)-**1** which occupies the S3 sub-pocket.

Several *N,N*-disubstituted amide analogues (*S*,*R*)-**2a,b,d-f** were synthesized (see Section 2.3) bearing hydrophobic groups that would prefer the more hydrophobic S3 sub-pocket of Plm IV. Unexpectedly the *N,N-*diethyl and *N,N-*dimethyl substituted analogues (*S*,*R*)-**2a** and (*S*,*R*)-**2b** showed the highest selectivity factor for Plm IV inhibition over Cat D, even though our docking studies indicated that the wider S3 sub-pocket of Plm IV could accommodate bulkier groups. Analogue (*S*,*R*)-**2c** bearing *N-*hydroxyethyl groups showed 3-fold weaker Plm IV inhibition potency than compound (*S*,*R*)-**2a**. Introduction of larger linear substituents such as *N,N*-dipropyl (compound (*S*,*R*)-**1b**), *N,N*-di(methoxyethyl) (compound (*S*,*R*)-**2d**) and *N,N*-di(3,3,3-trifluoropropyl) (compound (*S*,*R*)-**2e**) resulted in improved Cat D inhibition and correspondingly lower selectivity factors, suggesting that these groups fit well in the deep S3 sub-pocket of Cat D. Analogue (*S*,*R*)-**2f** bearing a *N,N*-diisobutyl amide showed poor inhibition of both enzymes, indicating that this group is too large to fit into the S3 sub-pocket of both Plm IV and Cat D.

**Fig. 2:**
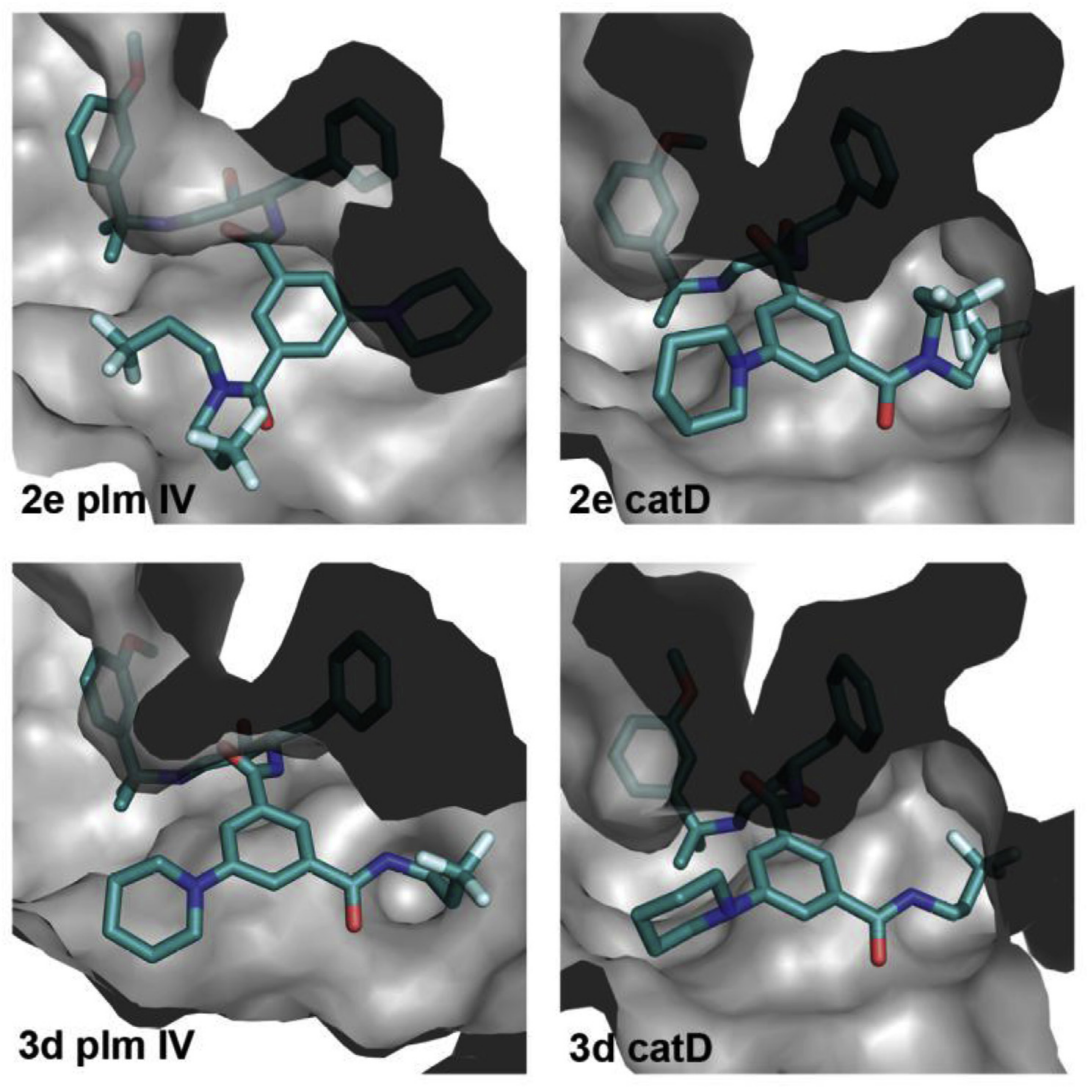
Docking models of compounds (*S*,*R*)-**2e** and (*S*,*R*)-**3d** in crystal structures of Plm IV and Cat D.

**Fig. 3:**
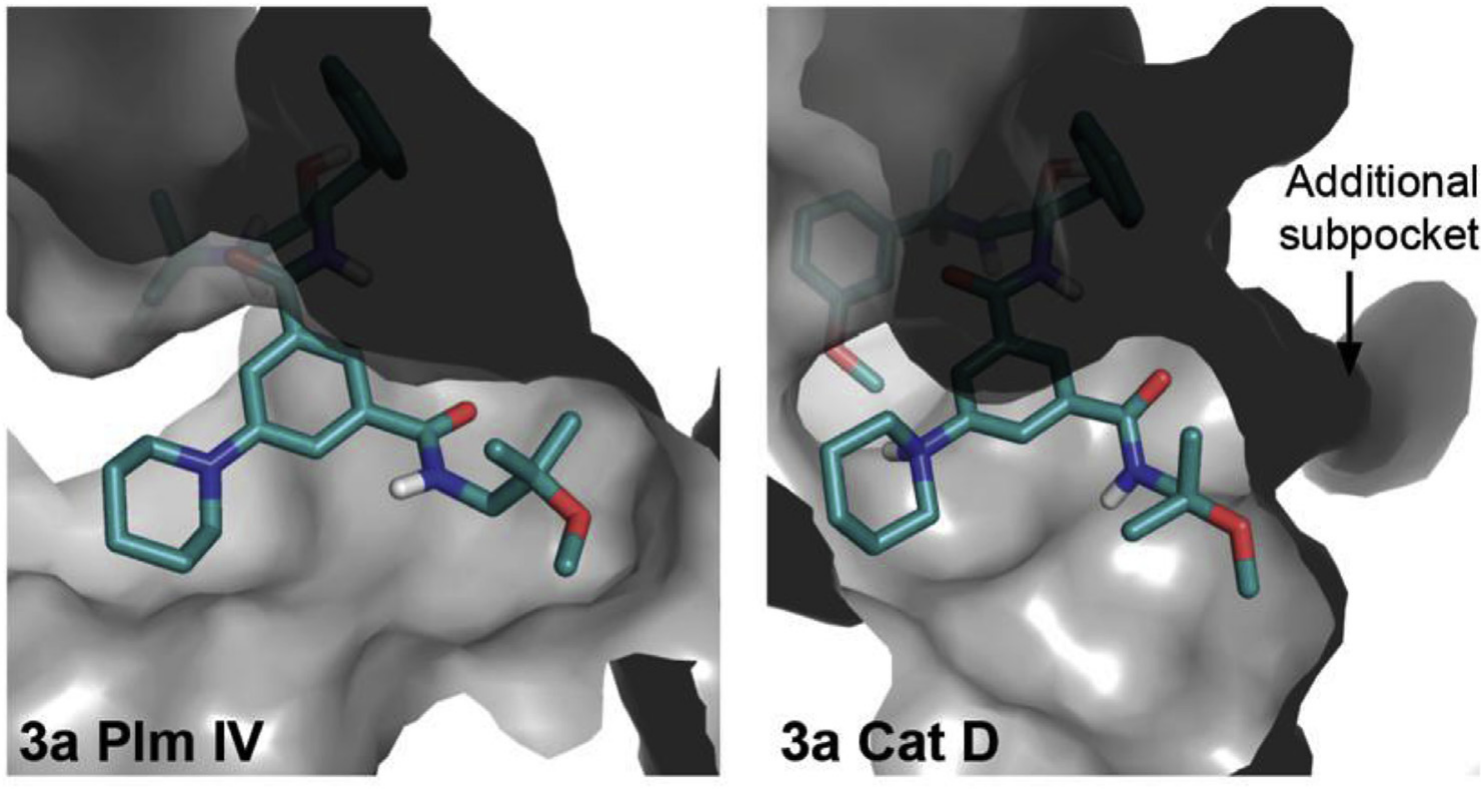
Docking models of compound **3a** in complex with Plm IV and Cat D.

We further explored *N-*monosubstituted amide analogues (*S*,*R*)-**3a-m** (see Section 2.3 for synthesis). The best compounds in this series showed similar Plm IV inhibition potency compared to the most potent *N,N*-disubstituted amides (*S*,*R*)-**2**. Gratifyingly, these appeared to be less potent Cat D inhibitors, leading to improved selectivity factors. The removal of one *N*-substituent was more beneficial for compounds with larger or branched substituents (e.g. (*S*,*R*)-**2e** and (*S*,*R*)-**2d** compared to (*S*,*R*)-**3d** and (*S*,*R*)-**3f**) while for compounds bearing smaller substituents a slight decrease in Plm IV inhibitory activity was observed e.g. (*S*,*R*)-**1b** (Table 2) compared to (*S*,*R*)-**1d** (Table 3) (see Fig. 2 for docking of compounds **2e** and **3d** into Plm IV and Cat D representing the difference of steric requirements). The docking studies suggested that the introduction of a hydrogen bond donor (inhibitors (*S*,*R*)-**3c,e,i**,**j**) or hydrogen bond acceptor ((*S*,*R*)-**3a**,**l,f)** group in the S3 sub-pocket substituents could potentially enable additional electrostatic interactions with electron-rich functional groups in this pocket. Hydrogen bond donor groups could interact with Asn13 and Leu14 backbone carbonyl in Plm IV; and Asp323, Tyr15, Gln14 and Ala13 in CatD, whereas for the hydrogen bond acceptors the most likely interactions are with Asn13 in Plm IV and Gln14 in Cat D (Fig. S1, see supporting information). Although the introduction of hydrogen bond donor or acceptor groups in the S3 sub-pocket occupying substituent reduced Plm IV inhibition activity (up to 2 times if comparing **1b** and **3a)**, it also produced the most selective ligand in this series - (*S*,*R*)-**3a**, as the activity decrease for Cat D is even higher (up to 19 times if comparing (*S*,*R*)-**1b** and (*S*,*R*)-**3a**). The docking studies suggest that the relatively higher drop in activity against Cat D for the compounds bearing the hydrogen bond donor or acceptor groups is due to an unfilled hydrophobic sub-pocket (resulting in an entropic penalty of solvating the non-polar sub-pocket). That is, the position of the amide substituent remains the same in both enzymes due to additional interactions with aforementioned residues (Fig. 3), but by doing so it creates a situation where the Cat D hydrophobic sub-pocket is filled with water resulting in an entropic penalty and reduced Cat D inhibition activity.

**Table 2:**
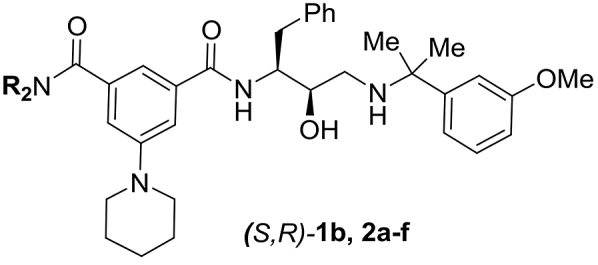
SAR of *N,N-*disubstituted amide analogues (*S*,*R*)-**1b**, **2a-f**

Comp.	R	IC_50_ Plm IV, μΜ	IC_50_ Cat D, μΜ	S^a^
(*S*,*R*)-**1b**	*n*-Pr	0.024^b^	0.042^b^	1.8
(*S*,*R*)-**2a**	Et	0.014	0.25	17.9
(*S*,*R*)-**2b**	Me	0.087	0.5	5.7
(*S*,*R*)-**2c**	HOCH_2_CH_2_	0.068	0.27	4.0
(*S*,*R*)-**2d**	MeOCH_2_CH_2_	0.037	0.10	2.7
(*S*,*R*)-**2e**	CF_3_CH_2_CH_2_	0.21	0.12	0.57
(*S*,*R*)-**2f**	(CH_3_)_2_CHCH_2_	0.5	1.3	2.6

a Selectivity factor of Plm IV/Cat D inhibition.

b Data from literature [11].

**Table 3:**
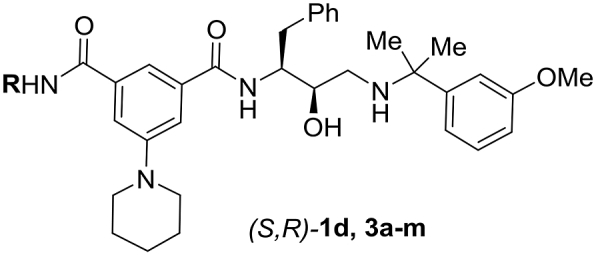
SAR of *N-*mono-substituted amide analogues (*S*,*R*)-**1d**, **3a-m**

Comp.	R	IC_50_ Plm IV, μΜ	IC_50_ Cat D, μΜ	S^a^
(*S*,*R*)-**1d**	n-Pr	0.038^b^	0.11^b^	2.9
(*S*,*R*)-**3a**	MeOC(CH_3_)_2_CH_2_	0.048	2.1	43.8
(*S*,*R*)-**3b**	c-PrCH_2_	0.030	0.76	25.3
(*S*,*R*)-**3c**	HOCH_2_CH_2_CH_2_	0.093	2.25	24.2
(*S*,*R*)-**3d**	CF_3_CH_2_CH_2_	0.024	0.58	24.2
(*S*,*R*)-**3e**	HOC(CH_3_)_2_CH_2_	0.10	1.66	16.6
(*S*,*R*)-**3f**	MeOCH_2_CH_2_	0.05	0.75	15.0
(*S*,*R*)-**3g**	Me_2_NCH_2_CH_2_	0.36	4.8	13.3
(*S*,*R*)-**3h**	t-BuCH_2_	0.027	0.40	14.8
(*S*,*R*)-**3i**	HOCH_2_C(CH_3_)_2_	0.12	1.46	12.2
(*S*,*R*)-**3j**	HOCH_2_CH_2_	0.21	1.42	6.8
(*S*,*R*)-**3k**	PhCH_2_	0.038	0.22	5.8
(*S*,*R*)-**3l**	t-BuOCH_2_CH_2_** **	0.031	0.15	4.8
(*S*,*R*)-**3m**	c-HexCH_2_	0.09	0.15	1.7

a Selectivity factor of Plm IV/Cat D inhibition.

b Data from literature [11].

Despite varying hydrogen bond donor and acceptor groups in the S3 sub-pocket filling substituents, docking studies also suggest that the main factor affecting Plm IV and Cat D inhibition potency is the size and shape of the substituent; branched and long substituents cannot fit into the narrow S3 recess of Cat D whereas the open S3 sub-pocket of Plm IV can accommodate such substituents. The weakest inhibitor of the *N*-mono-substituted amide analogues was compound (*S*,*R*)-**3g**. This could be explained by the protonated amine group as the amide substituent which, according to the docking studies, tends to form hydrogen bonds and ionic interactions with residues outside of the S3 sub-pocket. Altogether these results indicate that the selectivity against Cat D can be improved by targeting the S3 sub-pocket with mono-substituted amide moieties containing linear or branched hydrophobic groups.

The S4 sub-pocket is another inhibitor binding region which is notably distinct between Plm IV and Cat D (Fig. 1A). Although predominantly hydrophobic in both enzymes, the S4 sub-pocket of Plm IV is flatter and more solvent exposed. Therefore, we investigated SAR for substituents occupying the S4 sub-pocket in the series of compounds (*S*,*R*)-**4a-g** (Table 4, see Section 2.3. for synthesis). Installation of an *N,N*-dipropylamide group (compound (*S*,*R*)-**4g**) resulted in practically unchanged Plm IV inhibitory activity compared to parent compound **1b**, however this was paralleled by a more than 15-fold drop in activity against Cat D resulting in improvement of the selectivity factor. Installation of fluorine or removal of the S4 filling substituent provided compounds **4b,c** with slightly decreased Plm IV inhibitory potency, whereas inhibition of Cat D was considerably lower which again improved the selectivity factor. Installation of chlorine in the benzene ring (compound **4d)** improved the activity for both Plm IV and Cat D. Cyano, methyl and trifluoromethyl groups in the S4 sub-pocket (compounds **4a,e-f**) made less difference for inhibitor binding to Plm IV and Cat D. Collectively, these effects of S4 occupying substituents are difficult to explain from our molecular modelling data, and presumably arise from an interplay of hydrophobic and polar non-covalent interactions.

**Table 4:**
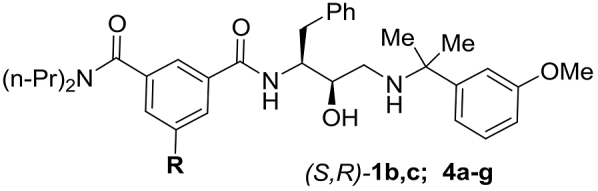
SAR of phenylgroup substitution in analogues **1b,c**, **4a-g**

Comp.	R	IC_50_ Plm IV, μΜ	IC_50_ Cat D, μΜ	S^a^
(*S*,*R*)-**1b**	1-piperidinyl	0.024^b^	0.042^b^	1.8
(*S*,*R*)-**1c**	Ph	0.006^b^	0.054^b^	9.0
(*S*,*R*)-**4a**	Me	0.023	0.21	9.1
(*S*,*R*)-**4b**	H	0.058	1.15	19.8
(*S*,*R*)-**4c**	F	0.050	1.0	20
(*S*,*R*)-**4d**	Cl	0.008	0.096	12.0
(*S*,*R*)-**4e**	CF_3_	0.015	0.067	4.5
(*S*,*R*)**-4f**	CN	0.059	0.56	9.5
(*S*,*R*)**-4g**	(n-Pr)_2_NC(=O)	0.018	0.7	38.9

a Selectivity factor of Plm IV/Cat D inhibition.

b Data from literature [11].

Considering that the lack of an S4 occupying substituent considerably improves selectivity against Cat D, analogues (*S*,*R*)-**5a,b** of the most potent *N-*mono-substituted amide inhibitors (*S*,*R*)-**3d**,**h** were prepared and tested (Table 5, see Section 2.3. for synthesis). As expected, a slight drop in Plm IV inhibitory potency was observed; however the modifications appeared to be additive for a considerable improvement in the selectivity factor.

**Table 5:**
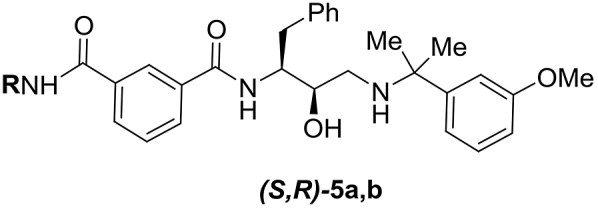
Combining selectivity inducing structural motives in analogues (*S*,*R*)-**5a,b**

Comp.	R	IC_50_ Plm IV, μΜ	IC_50_ Cat D, μΜ	S^a^
(*S*,*R*)-**5a**	t-BuCH_2_	0.076	3.8	50.0
(*S*,*R*)-**5b**	CF_3_CH_2_CH_2_	0.15	4.9	32.6

a Selectivity factor of Plm IV/Cat D inhibition.

### 2.2 Relation of Plm subtype inhibition with *P. falciparum* growth inhibition

The capacity to inhibit growth in vitro of asexual blood stage *P. falciparum* was determined for selected compounds (*S*,*R*)-**2a**,**3a,b,d,h**,**4b,c,g**,**5a,b** (Table 6). In all cases, the compounds showed strong growth inhibitory potency with EC_50_ values in the low nanomolar range. Inhibitory activity against Plm I, Plm II and Plm IV was determined for the same compounds. This revealed no correlation between inhibitory potency in the parasite growth assay and inhibition of Plm I and II enzyme activity. In contrast, there was a much better correlation between parasite growth inhibition potency and Plm IV inhibition potency, although even here there were some notable exceptions; for instance, the most potent growth inhibitory compound, (*S*,*R*)-**4b,** was a 3-fold weaker inhibitor of Plm IV than compound (*S*,*R*)-**4g**, yet showed a 20-fold better inhibition of parasite growth than (*S*,*R*)-**4g**.

**Table 6:** Plm I, II, IV inhibition and *P. falciparum* growth inhibition activity of selected compounds.

Comp.	IC_50_ Plm I, μΜ	IC_50_ Plm II, μΜ	IC_50_ Plm IV, μΜ	S^a^	EC_50_^b^ *Pf* Growth, nΜ
(*S*,*R*)-**2a**	0.8	0.16	0.014	17.9	1.5
(*S*,*R*)-**3a**	7.4	5.4	0.048	43.8	2.0
(*S*,*R*)-**3b**	1.8	0.5	0.030	25.3	1.8
(*S*,*R*)-**3d**	2.5	2.2	0.024	24.2	2.0
(*S*,*R*)-**3h**	2.0	0.85	0.027	14.8	6.0
(*S*,*R*)-**4b**	3.1	1.7	0.058	19.8	0.3
(*S*,*R*)-**4c**	1.1	1.1	0.050	20	1.5
(*S*,*R*)-**4g**	0.78	0.27	0.018	38.9	6.0
(*S*,*R*)-**5a**	5.6	7.1	0.076	50.0	2.0
(*S*,*R*)-**5b**	10.3	10.4	0.15	32.6	6.0

a Selectivity factor of Plm IV/Cat D inhibition.

b The EC_50_ values for *P. falciparum* growth were determined using a SYBR Green-based assay with an incubation time of 96 h (2 erythrocytic cycles). Samples were each measured in triplicate, in 2 separate biological assays. Compound TCMDC-134674^11,20^ was used as a positive control (see Supporting Information).

**Fig. 4:**
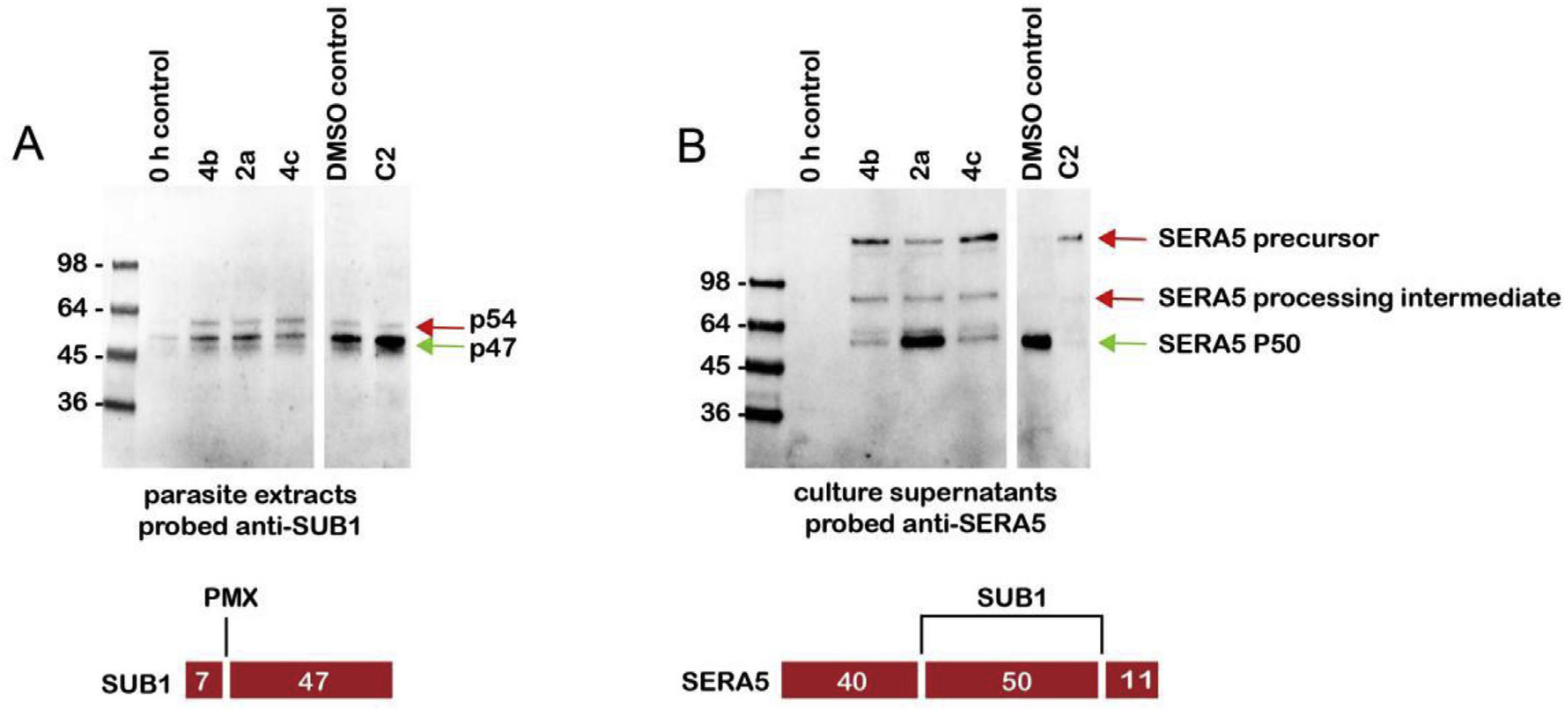
Inhibition of *P. falciparum* SUB1 maturation and egress by selected compounds indicates that they target Plm X. (A) Synchronous cultures of immature intracellular parasites were treated for ∼8 h with compounds (*S*,*R*)-**2a** or (*S*,*R*)-**4b,c** (10 nM), or vehicle only (DMSO, 1% v/v), or the cGMP-dependent protein kinase inhibitor (4-[7-[(dimethylamino)methyl]-2-(4-fluorphenyl)imidazo[1,2-α]pyridine-3-yl]pyrimidin-2-amine (compound 2; C2, 2 μM) which inhibits egress but not SUB1 maturation. Extracts of the parasites were then analyzed by Western blot, probing with an antibody to SUB1. The positions of migration of the SUB1 p54 and p47 forms (green arrow) are indicated. The schematic below indicates the mode by which Plm X converts SUB1 p54 to the terminal p47 form. (B) Parasites treated for ∼ 24 h as in (A) were transferred to fresh medium containing the various compounds and allowed to undergo egress for 4 h before the culture supernatants were analysed by Western blot, probing with antibodies to the parasite protein SERA5. The positions of migration of the SERA5 precursor, a processing intermediate and the terminal P50 form (green arrow) are indicated. The schematic below indicates the mode by which SUB1 converts the SERA5 precursor to the P50 form. (For interpretation of the references to color in this figure legend, the reader is referred to the Web version of this article.)

These results implied that the important parasite target(s) engaged by the growth inhibitory compounds are not the hemoglobinase plasmepsins. Recent reports have shown that inhibitors of the non-hemoglobinase plasmepsins Plm IX and Plm X (which are structurally similar to Plm I, II and IV) can potently block parasite replication [29,30]. A key biological function of Plm X is the proteolytic maturation of SUB1, a parasite subtilisin-like serine protease that plays an essential role in regulating parasite release (egress) from the infected host erythrocyte [38]. SUB1 maturation comprises 2 steps in which the initial ∼82 kDa pre-proenzyme is cleaved to form first a 54 kDa protein (p54) then a 47 kDa terminal product (p47) which accumulates during the latter ∼12 h of intra-erythrocytic parasite development. Whilst the first SUB1 processing step is autocatalytic, the second p54-to-p47 step is believed to be mediated by Plm X [29,30]. We used a Western blot-based assay to examine the effects of selected compounds (*S*,*R*)-**2a**, (*S*,*R*)-**4b** and (*S*,*R*)-**4c** on both SUB1 maturation and parasite egress (Fig. 4).

Egress was quantified by measuring the release of a soluble parasite protein called SERA5 into parasite culture supernatants. SERA5 is also a SUB1 substrate, and is generally released in a P50 form that results from SUB1-mediated cleavage of a larger precursor. Release of correctly processed SERA5 P50 is therefore an indicator of both SUB1 activity and efficiency of egress. As shown in Fig. 4A, treatment of developing intracellular parasites with the three Plm inhibitors (*S*,*R*)-**2a**, (*S*,*R*)-**4b** and (*S*,*R*)-**4c** resulted in a relative enrichment of the p54 form of SUB1, indicating inhibition of conversion of p54 to the terminal p47 form. Analysis of culture supernatants from treated parasites allowed to proceed to egress (Fig. 4B) showed that compounds (*S*,*R*)-**4b** and (*S*,*R*)-**4c** both produced a clear reduction in SERA5 P50 release, whilst all three compounds produced an increase in the release of SERA5 precursor or processing intermediates, indicating defects in egress and SERA5 processing. These results strongly suggest that the mechanism of parasite growth inhibition by compounds (*S*,*R*)-**4b** and (*S*,*R*)-**4c** (as well as possibly (*S*,*R*)-**2a**) involves Plm X inhibition, since the effects on egress were linked to inhibition of SUB1 maturation and its enzymatic activity against an endogenous substrate. Given their structural similarity, it is likely that the other compounds **3a,b,d,h**, **4g** and **5a,b** exerting potency in the parasite growth assay (Table 6) also target Plm X.

### 2.3 Synthesis of inhibitors (*S*,*R*)–**2-5**

**Scheme 1:**
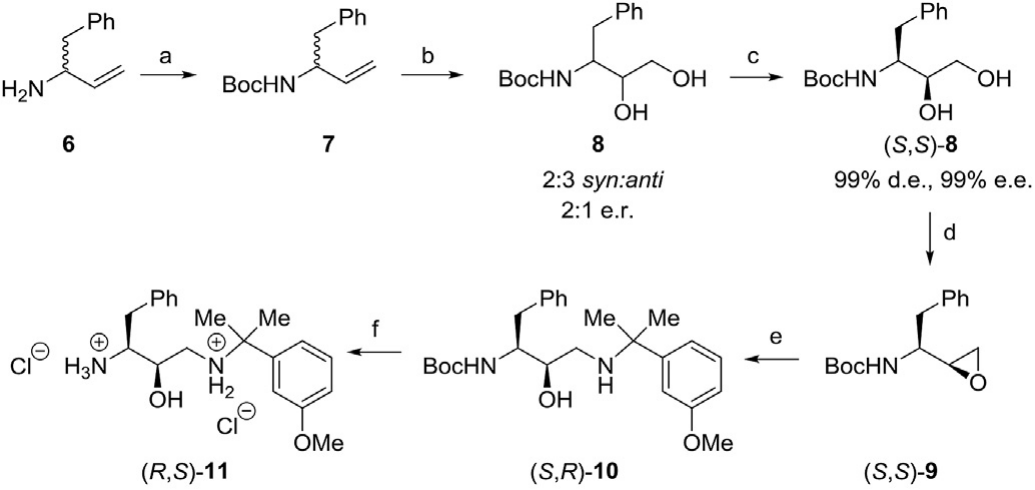
Synthesis of amino alcohol intermediate (*R*,*S*)–**11**. Reagents and conditions: a) Boc_2_O (1.25 equiv), NEt_3_ (2 equiv), DCM, rt, 2 h, 87%. b) AD-mix-α (1 equiv), 1:1 (v/v) *t*-BuOH:water, rt, 20 h c) preparative HPLC on chiral stationary phase (*Chiralpak-ID*), 25% in two steps. d) Ph_3_P (1.1 equiv), DEAD (1.1 equiv), CHCl_3_, 85 °C, 48 h, 64%. e) 2-(3-Methoxyphenyl)propan-2-amine [11] (1.05 equiv), *i*-PrOH, 70 °C, 40 h, 73%. f) 4 M HCl in dioxane, rt, 6 h, 99%.

**Scheme 2:**
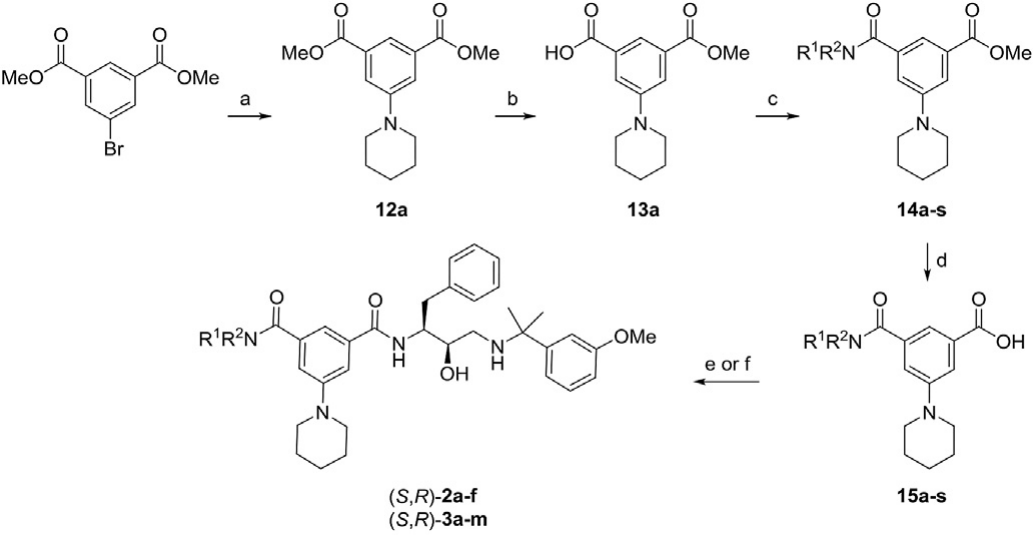
Synthesis of Plm inhibitors (*S*,*R*)–**2a-f** and (*S*,*R*)–**3a-m**. Reagents and conditions: a) piperidine (1 equiv), Pd(OAc)_2_ (5 mol%), *rac*-BINAP (5 mol%), Cs_2_CO_3_ (1.5 equiv), toluene, 100 °C, 18 h, 88%. b) General procedure A: aqueous 1 M NaOH (1 equiv), MeOH, rt, 16 h c) General procedure B: R^1^R^2^NH (1.2 equiv), HBTU (1 equiv), NEt_3_ (2 equiv), DMF, rt, 2 h d) General procedure C: aqueous 1 M NaOH (1.5 equiv), MeOH, 50 °C, 18 h e) General procedure D: amino alcohol (*R*,*S*)–**11** (1.0 equiv), HBTU (1.0 equiv), NEt_3_ (4 equiv), DMF, rt, 16 h f) amino alcohol *rac*–**11** (1.0 equiv), HBTU (1.0 equiv), NEt_3_ (4 equiv), DMF, rt, 16 h; then separation of enantiomers by HPLC on chiral stationary phase (*Chiralpak-ID*).

**Scheme 3:**
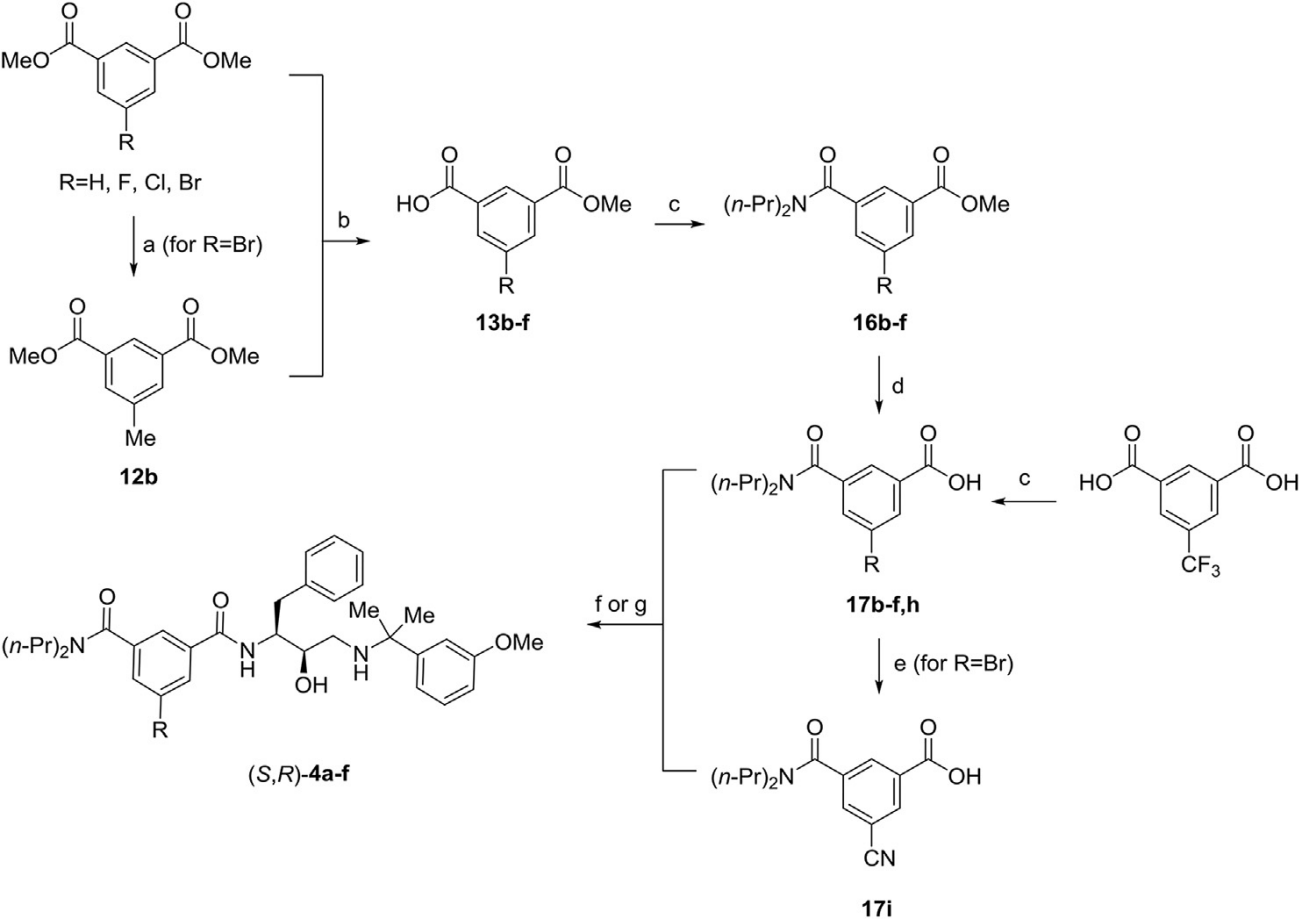
Synthesis of Plm inhibitors (*S*,*R*)–**4a-f**. Reagents and conditions: a) MeB(OH)_2_ (1.2 equiv), Pd(dppf)Cl_2_xCH_2_Cl_2_ (5 mol%), K_3_PO_4_ (3 equiv), toluene, 90 °C, 18 h b) General procedure A: aqueous 1 M NaOH (1 equiv), MeOH, rt, 16 h c) General procedure B: (*n*-Pr)_2_NH (1.2 equiv), HBTU (1 equiv), NEt_3_ (2 equiv), DMF, rt, 2 h d) General procedure C: aqueous 1 M NaOH (1.5 equiv), MeOH, 50 °C, 18 h e) CuCN (2 equiv), NMP, 160 °C, 6 h f) General procedure D: amino alcohol (*R*,*S*)–**11** (1.0 equiv), HBTU (1.0 equiv), NEt_3_ (4 equiv), DMF, rt, 16 h g) amino alcohol *rac*–**11** (1.0 equiv), HBTU (1.0 equiv), NEt_3_ (4 equiv), DMF, rt, 16 h; then separation of enantiomers by HPLC on chiral stationary phase (*Chiralpak-ID*).

**Scheme 4:**
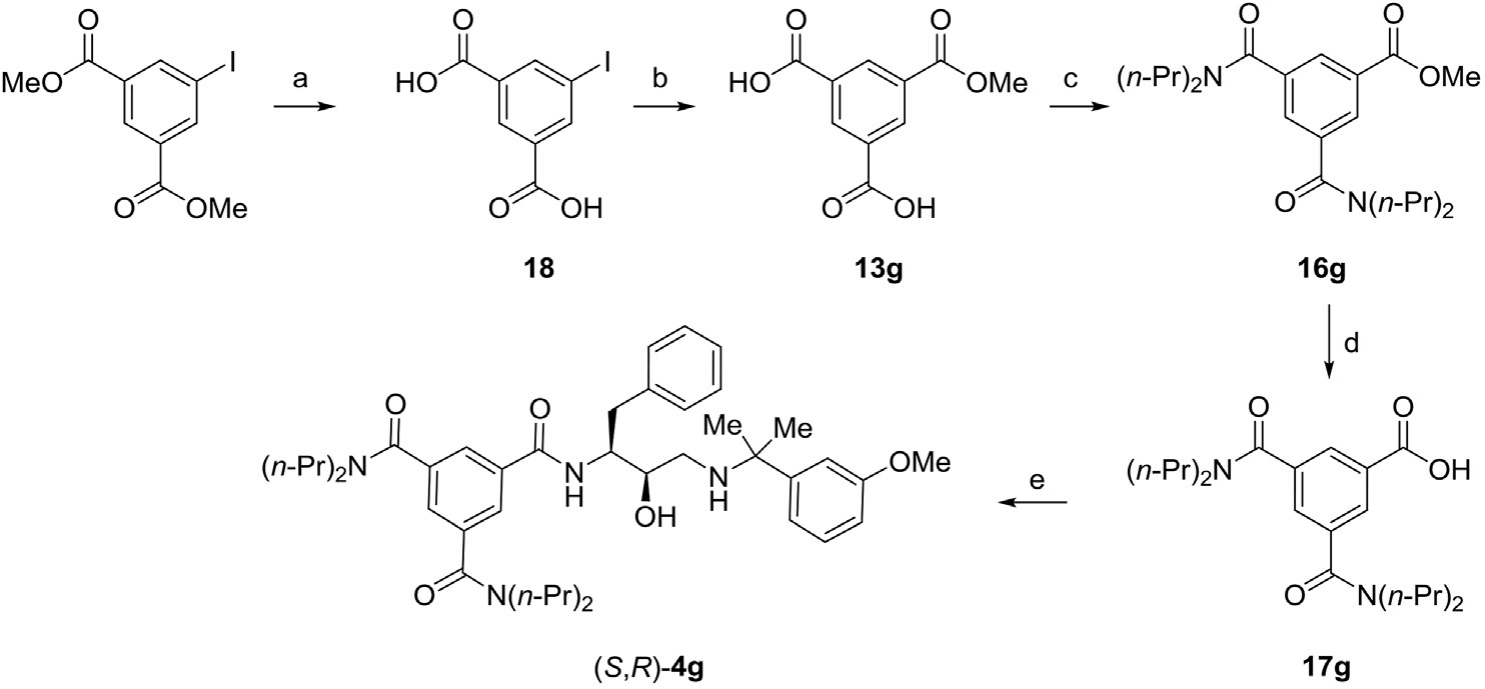
Synthesis of Plm inhibitor (*S*,*R*)–**4g**. Reagents and conditions: a) aqueous 1 N NaOH (3 equiv), MeOH, 40 °C, 18 h b) Pd(dppf)Cl_2_xCH_2_Cl_2_ (10 mol%), NEt_3_ (2.2 equiv), CO (70 psi), MeOH, 100 °C, 18 h c) General procedure B: (*n*-Pr)_2_NH (2.2 equiv), HBTU (2 equiv), NEt_3_ (4 equiv), DMF, rt, 2 h d) General procedure C: aqueous 1 M NaOH (1.5 equiv), MeOH, 50 °C, 18 h e) General procedure D: amino alcohol (*R*,*S*)–**11** (1.0 equiv), HBTU (1.0 equiv), NEt_3_ (4 equiv), DMF, rt, 16 h.

**Scheme 5:**
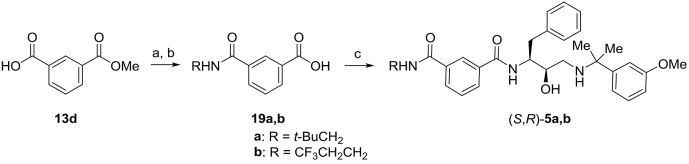
Synthesis of Plm inhibitors **5a**,**b**. Reagents and conditions: a) General procedure B: (*n*-Pr)_2_NH (1.2 equiv), HBTU (1 equiv), NEt_3_ (2 equiv), DMF, rt, 2 h b) General procedure C: aqueous 1 M NaOH (1.5 equiv), MeOH, 50 °C, 18 h c) General procedure D: amino alcohol (*R*,*S*)–**11** (1.0 equiv), HBTU (1.0 equiv), NEt_3_ (4 equiv), DMF, rt, 16 h.

Plm inhibitors (*S*,*R*)–**2-5** were synthesized from substituted benzoic acids **15**, **17** and **19** and either enantiomerically pure amino alcohol (*R*,*S*)–**11** or the corresponding racemate *rac*-**11** (Scheme 1, Scheme 2, Scheme 3, Scheme 4, Scheme 5). Inhibitors (*S*,*R*)–**2b-e**, (*S*,*R*)–**3a,c,e-g,i,j,l,m**, (*S*,*R*)–**4c,f,g** and (*S*,*R*)–**5a,b** were obtained from enantiomerically pure amino alcohol (*R*,*S*)–**11**, whereas targets **2a**,**f**, **3b,d,h,k** and **4a,b,d,e** were obtained as mixtures of stereoisomers from racemic alcohol *rac*-**11**. Enantiomerically pure inhibitors (*S*,*R*)–**2a**,**f**, (*S*,*R*)–**3b,d,h,k** and (*S*,*R*)–**4a,b,d,e** were obtained by separation of diastereomers using chromatography on silica gel, followed by separation of enantiomers using the chromatography on a chiral stationary phase.

Synthesis of aminoalcohol **11** commenced with *N*-Boc protection of *rac*-**6** (Scheme 1). Dihydroxylation of the resulting carbamate *rac*–**7** with AD-mix-α afforded diol **8** as a 2:3 mixture of *syn:anti* diastereomers and a 2:1 mixture of enantiomers. Enantiomerically pure diol (*S*,*S*)–**8** was obtained by an initial separation of *syn/anti* diastereomers using chromatography on silica gel, followed by separation of *syn*–**8** enantiomers by chromatography on a chiral stationary phase. The major enantiomer turned out to be the desired diol (*S*,*S*)–**8** as evidenced by comparison of optical rotation data with that from the literature [39]. Conversion of (*S*,*S*)–**8** into epoxide (*S*,*S*)–**9** under Mitsunobu conditions was followed by aminolysis with 2-(3-methoxyphenyl)propan-2-amine [11] to afford *N*-Boc protected amino alcohol (*S*,*R*)–**10**. Finally, the cleavage of *N*-Boc protecting group yielded amino alcohol (*R*,*S*)–**11**.

Benzoic acids **15a-s** were prepared from dimethyl 5-bromoisophthalate (Scheme 2). Pd-catalyzed amination with piperidine afforded **12a**, which was hydrolysed to isophthalic monoester **13a** under basic conditions. Subsequent HBTU-mediated condensation with amines afforded amides **14a-s**, which were hydrolysed to benzoic acids **15a-s**. (see Table 7)

**Table 7:** Substitution pattern of compounds in Scheme 2.

R^1^	R^2^	No.	No.	No.	Comments
Et	Et	**14a**	**15a**	(*S*,*R*)-**2a**	from *rac*–**11** (step f)
Me	Me	**14b**	**15b**	(*S*,*R*)-**2b**	
HOCH_2_CH_2_	HOCH_2_CH_2_	**14c**	**15c**	(*S*,*R*)-**2c**	
MeOCH_2_CH_2_	MeOCH_2_CH_2_	**14d**	**15d**	(*S*,*R*)-**2d**	
CF_3_CH_2_CH_2_	CF_3_CH_2_CH_2_	**14e**	**15e**	(*S*,*R*)-**2e**	
(CH_3_)_2_CHCH_2_	(CH_3_)_2_CHCH_2_	**14f**	**15f**	(*S*,*R*)-**2f**	from *rac*–**11** (step f)
H	MeOC(CH_3_)_2_CH_2_	**14g**	**15g**	(*S*,*R*)-**3a**	
H	c-PrCH_2_	**14h**	**15h**	(*S*,*R*)-**3b**	from *rac*–**11** (step f)
H	HOCH_2_CH_2_CH_2_	**14i**	**15i**	(*S*,*R*)-**3c**	
H	CF_3_CH_2_CH_2_	**14j**	**15j**	(*S*,*R*)-**3d**	from *rac*–**11** (step f)
H	HOC(CH_3_)_2_CH_2_	**14k**	**15k**	(*S*,*R*)-**3e**	
H	MeOCH_2_CH_2_	**14l**	**15l**	(*S*,*R*)-**3f**	
H	Me_2_NCH_2_CH_2_	**14m**	**15m**	(*S*,*R*)-**3g**	
H	t-BuCH_2_	**14n**	**15n**	(*S*,*R*)-**3h**	from *rac*–**11** (step f)
H	HOCH_2_C(CH_3_)_2_	**14o**	**15o**	(*S*,*R*)-**3i**	
H	HOCH_2_CH_2_	**14p**	**15p**	(*S*,*R*)-**3j**	
H	PhCH_2_	**14q**	**15q**	(*S*,*R*)-**3k**	from *rac*–**11** (step f)
H	t-BuOCH_2_CH_2_	**14r**	**15r**	(*S*,*R*)-**3l**	
H	c-HexCH_2_	**14s**	**15s**	(*S*,*R*)-**3m**	

Similar synthetic approach was also used for the preparation of acids **17b-i** (Scheme 3). Accordingly, monoesters **13c-f** were obtained from commercially available dimethyl isophthates. The synthesis of methyl ester **13b** required an initial Pd-catalyzed alkylation of dimethyl 5-bromoisophthalate with methylboronic acid, followed by hydrolysis of one of the two ester moieties. Benzoic acids **13b-f** were converted into amides **16b-f** and ester moieties were hydrolysed to afford acids **17b-f**. Benzoic acid **17h** was obtained directly from trifluoromethyl isophthalic acid and *n*-Pr_2_NH in the presence of HBTU, whereas the synthesis of **17i** was accomplished by Cu(I)-catalyzed substitution of bromide in benzoic acid **17c** for cyano group (Scheme 3). (see Table 8)

**Table 8:** Substitution pattern of compounds in Scheme 3.

R	No.	No.	No.	No.	No.	Comments
Me	**12b**	**13b**	**16b**	**17b**	(*S*,*R*)–**4a**	from *rac*–**11** (step g)
Br	**-**	**13c**	**16c**	**17c**	–	
H	**-**	**13d**	**16d**	**17d**	(*S*,*R*)–**4b**	from *rac*–**11** (step g)
F	**-**	**13e**	**16e**	**17e**	(*S*,*R*)–**4c**	
Cl	**-**	**13f**	**16f**	**17f**	(*S*,*R*)–**4d**	from *rac*–**11** (step g)
CF_3_	**-**	**-**	**-**	**17h**	(*S*,*R*)–**4e**	from *rac*–**11** (step g)
CN	**-**	**-**	**-**	**17i**	(*S*,*R*)–**4f**	

Synthesis of benzoic acid **17g** (Scheme 4) commenced with hydrolysis of commercially available dimethyl iodo-isophthalate to the corresponding isophthalic acid **18**, followed by Pd-catalyzed methoxycarbonylation [40] to afford ester **13g**. Subsequent conversion to diamide **16g** in the presence of HBTU was followed by ester hydrolysis to form benzoic acid **17g** (Scheme 4). The amide bond formation-hydrolysis sequence was also used for the preparation of benzoic acids **19a,b** (Scheme 5).

## 3 Summary

The optimization of hydroxyethylamine based Plm inhibitors was performed with the aim of improving selectivity against the related human aspartic protease Cat D. The studies were performed using Plm IV as a readily accessible model protein, the inhibition of which was previously found to correlate with *Plasmodium falciparum* growth inhibition. Based on sequence alignment of Plm IV and Cat D, putative selectivity inducing structural motifs were sought in S3 and S4 sub-pocket-occupying substituents of the inhibitors. Installation of an S3 sub-pocket targeting mono-substituted amide moiety in compounds (*S*,*R*)-**3** containing linear or branched hydrophobic groups resulted in up to 40-fold selectivity (compound (*S*,*R*)-**3a**) against Cat D. Plm IV inhibitors (*S*,*R*)-**4b,c** with no substituents or fluorine targeting the S4 sub-pocket led to 20-fold selectivity against Cat D, though with some loss of Plm IV inhibition potency. Surprisingly, installation of amide as the S4 sub-pocket filling group in compound (*S*,*R*)-**4g** resulted in potent Plm IV inhibition with almost 40-fold selectivity against Cat D. Selectivity-inducing factors in S3 and S4 positions were additive as evidenced by compound (*S*,*R*)-**5a** (50-fold selectivity). Determination of *P. falciparum* growth inhibition potency for ten of the new Plm inhibitors showed them to display activities in the low nanomolar range. The potent anti-malarial activity did not correlate with the relatively weak inhibition of Plm I and II, whilst in contrast there was a much better correlation with Plm IV inhibition. More detailed investigation of the mechanism of action of the selected compounds showed that they interfered with parasite egress and maturation of the parasite serine protease SUB1, indicating a strong link between anti-malarial activity and inhibition of the non-hemoglobinase plasmepsin and SUB1 maturase Plm X. Future studies should clarify whether cooperative Plm IV and Plm X inhibition or only Plm X inhibition is necessary to achieve optimal anti-malarial activity.

## Appendix A Supplementary data

EJMECH-D-18-02423R1_SI_Revised EJMECH-D-18-02423R1_SI_Revised
