# Supplementary material for: Peptidomimetic plasmepsin inhibitors with potent anti-malarial activity and selectivity against cathepsin D
Source: Eur J Med Chem. 2019 Feb 1;163:344–52. doi: 10.1016/j.ejmech.2018.11.068 (PMC6336538; doi:10.1016/j.ejmech.2018.11.068)
Supplement: EJMECH-D-18-02423R1_SI_Revised [file mmc1.pdf]

Supporting Information

Peptidomimetic Plasmepsin Inhibitors with Potent anti-Malarial activity and Selectivity Against Cathepsin D

Rimants Zogota[a]*, Linda Kinena*[a]*, Chrislaine Withers-Martinez*[b]*, Michael J Blackman*[b,c]*, Raitis Bobrovs*[a]*, Teodors Pantelejevs*[a]*, Iveta Kanepe-Lapsa*[a]*, Vita Ozola*[a]*, Kristaps Jaudzems*[a]*, Edgars Suna*[a*]*, Aigars Jirgensons*[a*]

[a]Latvian Institute of Organic Synthesis, Aizkraukles 21, Riga LV-1006, Latvia

[b]Malaria Biochemistry Laboratory, The Francis Crick Institute, 1 Midland Road, London NW1 1AT, UK

[c]Faculty of Infectious and Tropical Diseases, London School of Hygiene & Tropical Medicine, London WC1E 7HT, UK

Contents

General Information. S2
1. Synthesis S2
2. Molecular modeling S48
3. Biological assays S49
3.1. Enzymatic assay S49
3.2. Red blood cell assay S49
Spectral Data S50

General Information.

Unless otherwise noted, all chemicals were used as obtained from commercial sources and all reactions were performed under nitrogen or argon atmosphere in glassware dried in an oven (120 °C) and cooled under a stream of nitrogen or argon. Toluene, THF and 1,4-dioxane were distilled from sodium/benzophenone prior the use. Dry CH_2Cl_2 and Et_2O were obtained by passing commercially available anhydrous solvents through activated alumina columns.

Analytical thin-layer chromatography (TLC) was performed on precoated silica gel F-254 plates. High-resolution mass spectra were recorded on a mass spectrometer with a time-of-flight (TOF) mass analyzer. Nuclear magnetic resonance spectra were recorded on NMR spectrometers at the following frequencies: ^{1}H, 300 or 400 MHz; 13C {1H}, 75 or 101 MHz. Chemical shifts were referenced to Me_4Si as an internal reference or to residual solvent peaks.

The purity of all target inhibitors was confirmed to be ≥95% by reversed-phase UPLC-MS assay and by HRMS-ESI technique.

1. Synthesis

General procedure A for hydrolysis of diesters No.13a-f. An aqueous 1 M NaOH solution (1.0 eq) was added to a solution of diester **No.12a-b** (1.0 eq) in MeOH (1 mL/0.1 mmol of the diester). After stirring at room temperature for 16 h, the solution was acidified with aqueous 5% $KHSO_4$ solution to pH 3, diluted with water (20 mL) and extracted with EtOAc (3 x 20 mL). Combined organic extracts were washed with brine (20 mL), dried over anhydrous Na_2SO_4 and evaporated under reduced pressure to afford monoester.

General procedure B for the synthesis of amides No.14a-s; 16b-g. An oven-dried 20 mL pressure vial was charged with monoester **No.13a-f** (1.0 eq) and dissolved in anhydrous DMF (0.5 mL/0.1 mmol of the monoester). The corresponding amine (1.2 eq) was added, followed by HBTU (1.0 eq). The resulting solution was stirred at 0 °C for 5 min, then TEA (2.0 eq) was added and stirring at 0 °C was continued for 0.5 h. The resulting mixture was warmed to room temperature and stirring was continued for 2 h. The brownish solution was diluted with water (20 mL) and extracted with EtOAc (3 x 15 mL). Combined organic extracts were washed with water (15 mL), brine (15 mL), dried over anhydrous Na_2SO_4 and evaporated under reduced pressure. The residue was purified by silica gel column chromatography.

General procedure C for hydrolysis of benzoates No.15a-s; 17b-g; 19a-b. An aqueous 1 M NaOH solution (1.5 eq) was added to a solution of benzoate **No.14a-s; 16b-g** (1.0 eq) in MeOH (1 mL/0.1 mmol of the benzoate). The resulting solution was stirred at 50 °C for 18 h. After cooling to room

temperature, all volatiles were removed under reduced pressure. The white solid was diluted with water (20 mL) and acidified to pH 1 with aqueous 1M HCl solution. The aqueous layer was extracted with EtOAc (3 x 20 mL). Combined organic extracts were washed with brine (20 mL), dried over anhydrous Na_2SO_4 and evaporated under reduced pressure to afford benzoic acid.

General procedure D for the synthesis of inhibitor No.2a-f; 3a-m; 4a-g; 5a-b. An oven-dried 10 mL pressure vial was cooled under a stream of argon and charged with hydroxyethylamine hydrochloride (1.0 eq), benzoic acid (1.0 eq), HBTU (1.0 eq), and anhydrous DMF (1.2 mL/0.1 mmol of the hydroxyethylamine hydrochloride). The resulting solution was stirred at 0 °C for 5 min, then TEA (4.0 eq) was added and stirring at 0 °C was continued for 0.5 h. After warming to room temperature and stirring for 16 h, the brownish solution was diluted with water (20 mL) and extracted with EtOAc (3 x 15 mL). Combined organic extracts were washed with water (15 mL), brine (15 mL), dried over anhydrous Na_2SO_4. Evaporation under reduced pressure afforded residue, which was purified by silica gel column chromatography.

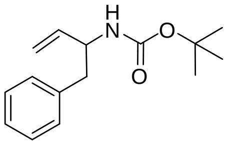

***tert*-Butyl *N*-(1-phenylbut-3-en-2-yl)carbamate (7).**

To a stirred solution of amine **6** (2.70 g, 18.4 mmol, 1.0 eq) in anhydrous DCM (50 mL) was added TEA (5.12 mL, 36.7 mmol, 2.0 eq), followed by di-*tert*-butyl dicarbonate (5.01 g, 23.0 mmol, 1.25 eq). After stirring at room temperature for 2 h the yellowish solution was extracted with DCM (3 x 30 mL), combined organic layers were dried over Na_2SO_4 and evaporated under reduced pressure. The crude product was purified by column chromatography on silica gel using gradient elution from 2% EtOAc in petroleum ether to 10% EtOAc in petroleum ether. Fraction spots on TLC were visualized using $KMnO_4$. The fractions containing the product **7** were evaporated to dryness under reduced pressure to give 3.95 g (87% yield) of **7** as a white solid. ^{1}H-NMR spectrum was identical to that from the literature.[1]

^{1}H-NMR (400 MHz, $CDCl_3$) δ: 7.32-7.27 (m, 2H, Ar-H), 7.25-7.16 (m, 3H, Ar-H), 5.85-5.75 (m, 1H, CH), 5.14-5.05 (m, 2H, CH_2), 4.54-4.33 (m, 2H, CH_2), 2.84 (d, J = 6.0 Hz, 2H, CH_2), 1.41 (s, 9H, CH_3×3) ppm. HRMS-ESI *(m/z)*: $[M+Na]^+$ Calcd for $C_{15}H_{21}NO_2Na$ 270.1470; Found 270.1473.

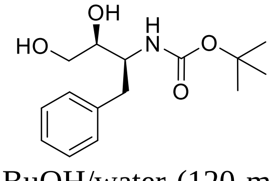

***tert*-Butyl *N*-[(2*S*,3*S*)-3,4-dihydroxy-1-phenylbutan-2-yl]carbamate ((*S*,*S*)-8).**

To a mixture of alkene **7** (3.94 g, 16.0 mmol, 1.0 eq) and 1:1 (v:v) *t*-BuOH/water (120 mL) was added AD-mix-α (12.42 g, 16.0 mmol, 1.0 eq). After stirring at room

[1] Jaudzems, K.; Tars, K.; Maurops, G.; Ivdra, N.; Otikovs, M.; Leitans, J.; Kanepe-Lapsa, I.; Domraceva, I.; Mutule, I.; Trapencieris, P.; Blackman, M. J.; Jirgensons, A. *ACS Med. Chem. Lett.* **2014**, *5*, 373–377.

temperature for 20 h the orange solution was cooled to 0 °C (crushed ice) and quenched with solid Na_2SO_3 (12.42 g). The stirring was continued for 0.5 h whereupon the orange color of the solution turned brown. The suspension was extracted with EtOAc (3 x 40 mL), dried over Na_2SO_4 and evaporated under reduced pressure. The crude product was purified by column chromatography on silica gel (eluent 10% *i*-PrOH in hexane). Fraction spots were visualized on thin layer chromatography silica gel using $KMnO_4$. The fractions containing the product were evaporated to dryness under reduced pressure. Enantiomerically pure product was obtained by preparative HPLC on chiral stationary phase (*Chiralpak-ID*) using 5% *i*-PrOH in hexanes as a mobile phase (flow rate 40 mL/min, detector UV 220 nm), to give 1.11 g (25% yield) of (*S*,*S*)-**8** as a white solid. ^{1}H-NMR (400 MHz, $CDCl_3$) δ: 7.35-7.30 (m, 2H, Ar-H), 7.26-7.22 (m, 3H, Ar-H), 4.54 (d, *J* = 8.7 Hz, 1H, NH), 3.89-3.79 (m, 1H, CH), 3.71-3.57 (m, 2H, CH, OH), 3.41-3.27 (m, 2H, OCH_2, OH), 3.10 (dd, *J* = 14.3, 4.3 Hz, 1H, OCH_2), 2.91 (dd, *J* = 14.3, 7.8 Hz, 1H, CH_2), 2.76 (d, *J* = 8.7 Hz, 1H, CH_2), 1.38 (s, 9H, CH_3×3) ppm. HRMS-ESI *(m/z)*: $[M+Na]^+$ Calcd for $C_{15}H_{23}NO_4Na$ 304.1525; Found 304.1521. Optical rotation $[\alpha]^{20}_D$ +10.1 (*c* 1.01, $CHCl_3$); Lit. [2] $[\alpha]^{23}_D$ +8.06 (*c* 0.62, $CHCl_3$)

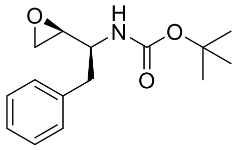

***tert*-Butyl *N*-[(1*S*)-1-[(2*S*)-oxiran-2-yl]-2-phenylethyl]carbamate ((*S*,*S*)-9)**. Ph_3P (1.13 g, 4.30 mmol, 1.1 eq) was added to a solution of diol (*S*,*S*)-**8** (1.10 g, 3.91 mmol, 1.0 eq) in anhydrous $CHCl_3$ (14 mL). Diethyl azodicarboxylate (0.68 mL, 4.30 mmol, 1.1 eq) was then added dropwise over a period of 10 min. Upon completion of the addition the light brown solution was stirred at 85 °C for 48 h. After cooling to room temperature water (20 mL) was added and the solution was extracted with DCM (3 x 20 mL), dried over Na_2SO_4 and evaporated under reduced pressure. The crude product was purified by column chromatography on silica gel using gradient elution from 5% EtOAc in petroleum ether to 15% EtOAc in petroleum ether to give product (*S*,*S*)-**9** as a white solid (655 mg, 64% yield). ^{1}H-NMR (400 MHz, $CDCl_3$) δ: 7.34-7.29 (m, 2H, Ar-H), 7.26-7.20 (m, 3H, Ar-H), 4.43 (br s, 1H, NH), 3.69 (br s, 1H, CH), 2.97 (dd, *J* = 14.0, 5.1 Hz, 1H, CH), 2.94-2.83 (m, 2H, CH_2), 2.80 (t, *J* = 4.9 Hz, 1H, CH_2), 2.77-2.73 (m, 1H, CH_2), 1.38 (s, 9H, CH_3×3) ppm. HRMS-ESI *(m/z)*: $[M+Na]^+$ Calcd for $C_{15}H_{21}NO_3Na$ 286.1419; Found 286.1419. Optical rotation $[\alpha]^{20}_D$ +3.0 (*c* 0.34, $CHCl_3$). Lit. [2] $[\alpha]^{23}_D$ +6.45 (*c* 1.86, $CHCl_3$)

[2] Ghosh, A. K.; Fidanze, S. *J. Org. Chem.*, **1998**, *63*, 6146-6152.

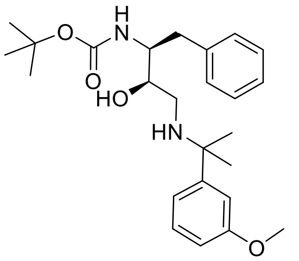

***tert*-Butyl *N*-[(2*S*,3*R*)-3-hydroxy-4-{[2-(3-methoxyphenyl)propan-2-yl]amino}-1-phenylbutan-2-yl]carbamate ((*S*,*R*)-10)**.

To a stirred solution of epoxide (*S*,*S*)-**9** (653 mg, 2.48 mmol, 1.0 eq) in anhydrous *i*-PrOH (7 mL) was added 2-(3-methoxyphenyl)propan-2-amine (430 mg, 2.60 mmol, 1.05 eq). After stirring at 70 ^{o}C for 40 h the yellowish solution was cooled to room temperature and then concentrated under reduced pressure. The crude product was purified by column chromatography on silica gel using gradient elution from 25% EtOAc in petroleum ether to 100% EtOAc to give product (*S*,*R*)-**10** as a colorless oil (775 mg, 73% yield). ^{1}H-NMR spectrum was identical to that from the literature.[3]

^{1}H-NMR (400 MHz, $CDCl_3$) δ: 7.30-7.16 (m, 6H, Ar-H), 7.04-6.91 (m, 2H, Ar-H), 6.75 (dd, *J* = 8.2, 2.5 Hz, 1H, Ar-H), 4.54 (d, *J* = 9.3 Hz, 1H, NH), 3.80 (s, 3H, OCH_3), 3.79-3.74 (m, 1H, CH), 3.30 (dt, *J* = 8.0, 4.6 Hz, 1H, CH), 2.96 (dd, *J* = 14.1, 4.6 Hz, 1H, CH_2), 2.81 (dd, *J* = 14.1, 8.0 Hz, 1H, CH_2), 2.46 (dd, *J* = 12.4, 4.6 Hz, 1H, CH_2), 2.38 (dd, *J* = 12.4, 3.2 Hz, 1H, CH_2), 1.45 (s, 6H, CH_3×2), 1.36 (s, 9H, CH_3×3) ppm. HRMS-ESI *(m/z)*: $[M+H]^+$ Calcd for $C_{25}H_{37}N_2O_4$ 429.2753; Found 429.2754. Optical rotation $[\alpha]^{20}_{D}$ –12.4 (*c* 0.43, $CHCl_3$).

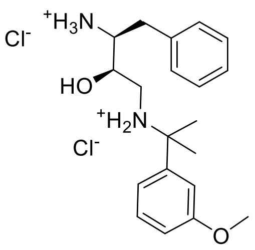

(2*R*,3*S*)-2-Hydroxy-*N*^{1}-[2-(3-methoxyphenyl)propan-2-yl]-4-phenylbutane-1,3-diaminium chloride ((*R*,*S*)-11).

HCl (4M solution in 1,4-dioxane, 8.2 mL, 33.0 mmol, 19.0 eq) was added to the amino alcohol (*S*,*R*)-**10** (744 mg, 1.74 mmol, 1.0 eq). After stirring at room temperature for 6 h the colorless solution was concentrated under reduced pressure to obtain hydroxyethylamine hydrochloride (*R*,*S*)-**11** (697 mg, 100% yield) as a light yellow solid, which was used in subsequent steps without purification.

^{1}H-NMR (400 MHz, DMSO-d_6) δ: 9.75 (t, *J* = 10.4 Hz, 1H, NH), 9.28 (t, *J* = 11.0 Hz, 1H, NH), 8.18 (d, *J* = 5.4 Hz, 2H, NH_2), 7.39-7.33 (m, 1H, Ar-H), 7.28-7.20 (m, 4H, Ar-H), 7.17-7.13 (m, 2H, Ar-H), 7.12-7.08 (m, 1H, Ar-H), 6.99-6.95 (m, 1H, Ar-H), 6.22-6.14 (m, 1H, NH), 4.11-4.02 (m, 1H, CH), 3.80 (s, 3H, OCH_3), 3.55-3.49 (m, 2H, CH, OH), 2.86-2.61 (m, 3H, CH_2, CH_2), 2.47-2.36 (m, 1H, CH_2), 1.67 (s, 3H, CH_3), 1.66 (s, 3H, CH_3) ppm. ^{13}C-NMR (101 MHz, DMSO-d_6) δ: 159.6, 140.9, 136.0, 129.9, 129.3, 128.6, 126.9, 118.3, 114.1, 112.2, 72.2, 70.6, 66.2, 61.4, 55.4, 54.6, 45.0, 33.2, 25.6, 25.1 ppm. HRMS-ESI *(m/z)*: $[M+H]^+$ Calcd for $C_{20}H_{29}N_2O_2$ 329.2229; Found 329.2239.

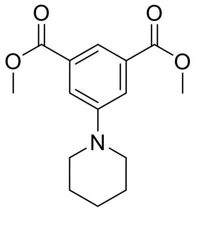

Dimethyl 5-(piperidin-1-yl)isophthalate (12a).

An oven-dried pressure tube (200 mL) was cooled under stream of argon and charged with dimethyl 5-bromoisophthalate (2.00 g, 7.32 mmol, 1.0 eq), palladium(II) acetate (82 mg, 0.37 mmol, 0.05 eq), *rac*-BINAP (228 mg, 0.37 mmol,

0.05 eq), Cs_2CO_3 (3.58 g, 11.0 mmol, 1.5 eq) and piperidine (0.72 mL, 7.32 mmol, 1.0 eq). The substances were degassed and anhydrous toluene (15 mL) was added. After heating at 100 ^{o}C for 18 h, the brownish suspension was cooled to room temperature and filtered through Celite. The filter plug was washed with EtOAc and filtrate was dried over anhydrous Na_2SO_4. Volatiles were evaporated under reduced pressure. The crude product was purified by column chromatography on silica gel using 10% EtOAc in petroleum ether to give product **12a** as a yellow solid (1.78 g, 88% yield). ^{1}H-NMR spectrum was identical to that from the literature.[3]

^{1}H-NMR (400 MHz, DMSO-d_6) δ: 7.85 (dd, *J* = 1.4, 1.4 Hz, 1H, Ar-H), 7.66 (d, *J* = 1.4 Hz, 2H, Ar-H), 3.86 (s, 6H, OCH_3×2), 3.24 (t, *J* = 4.9 Hz, 4H, NCH_2×2), 1.68-1.50 (m, 6H, CH_2×3) ppm. HRMS-ESI *(m/z)*: $[M+H]^+$ Calcd for $C_{15}H_{20}NO_4$ 278.1392; Found 278.1404.

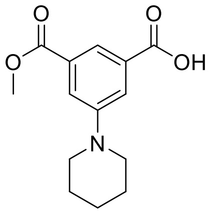

3-(Methoxycarbonyl)-5-(piperidin-1-yl)benzoic acid (13a).

The title compound was obtained as a light yellow solid material (1.03 g, 72% yield) from diester **12a** (1.52 g, 5.50 mmol, 1.0 eq) and aqueous 1 M NaOH solution (5.50 mL, 5.50 mmol, 1.0 eq) by following general procedure A. Pure material was obtained by column chromatography using gradient elution from 30% EtOAc in petroleum ether to 100% EtOAc. ^{1}H-NMR spectrum was identical to that from the literature.[4]

^{1}H-NMR (400 MHz, $CDCl_3$) δ: 8.16 (dd, *J* = 1.4, 1.4 Hz, 1H, Ar-H), 7.81 (d, *J* = 1.4 Hz, 2H, Ar-H), 3.94 (s, 3H, OCH_3), 3.31-3.26 (m, 4H, NCH_2×2), 1.77-1.69 (m, 4H, CH_2×2), 1.66-1.59 (m, 2H, CH_2) ppm. HRMS-ESI *(m/z)*: $[M+H]^+$ Calcd for $C_{14}H_{18}NO_4$ 264.1236; Found 264.1240.

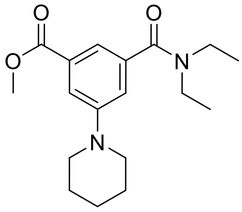

Methyl 3-(diethylcarbamoyl)-5-(piperidin-1-yl)benzoate (14a).

The title compound was obtained as a colorless oil (241 mg, 91% yield) from monoester **13a** (220 mg, 0.84 mmol, 1.0 eq), diethylamine (0.10 mL, 1.00 mmol, 1.2 eq), HBTU (317 mg, 0.84 mmol, 1.0 eq) and TEA (0,23 mL, 1.70 mmol, 2.0 eq) by following general procedure B. Pure material was obtained by column chromatography using gradient elution from 50% EtOAc in petroleum ether to 100% EtOAc.

^{1}H-NMR (400 MHz, $CDCl_3$) δ: 7.60 (dd, *J* = 2.6, 1.4 Hz, 1H, Ar-H), 7.42 (dd, *J* = 1.4, 1.4 Hz, 1H, Ar-H), 7.07 (dd, *J* = 2.6, 1.4 Hz, 1H, Ar-H), 3.90 (s, 3H, OCH_3), 3.58-3.50 (m, 2H, NCH_2), 3.29-3.20 (m, 6H, NCH_2×3), 1.73-1.66 (m, 4H, CH_2×2), 1.64-1.56 (m, 2H, CH_2), 1.29-1.20 (m, 3H, CH_3), 1.16-1.07 (m, 3H, CH_3) ppm. ^{13}C-NMR (101 MHz, $CDCl_3$) δ: 171.0, 167.1, 152.2, 138.4, 131.0, 118.1,

---

[3] Jaudzems, K.; Tars, K.; Maurops, G.; Ivdra, N.; Otikovs, M.; Leitans, J.; Kanepe-Lapsa, I.; Domraceva, I.; Mutule, I.; Trapencieris, P.; Blackman, M. J.; Jirgensons, A. *ACS Med. Chem. Lett.* **2014**, *5*, 373–377.

[4] Jaudzems, K.; Tars, K.; Maurops, G.; Ivdra, N.; Otikovs, M.; Leitans, J.; Kanepe-Lapsa, I.; Domraceva, I.; Mutule, I.; Trapencieris, P.; Blackman, M. J.; Jirgensons, A. *ACS Med. Chem. Lett.* **2014**, *5*, 373–377.

117.6, 117.3, 52.3, 50.1, 25.7, 24.3 ppm. HRMS-ESI *(m/z)*: $[M+H]^+$ Calcd for $C_{18}H_{27}N_2O_3$ 319.2022; Found 319.2028.

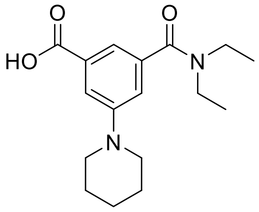

3-(Diethylcarbamoyl)-5-(piperidin-1-yl)benzoic acid (15a).

The title compound was obtained as a pink solid (134 mg, 60% yield) from benzoate **14a** (235 mg, 0.74 mmol, 1.0 eq) and aqueous 1 M NaOH solution (1.11 mL, 1.11 mmol, 1.5 eq) by following general procedure C. The crude product **15a** was used further without purification.

^{1}H-NMR (400 MHz, DMSO-d_6) δ: 7.46 (dd, *J* = 2.7, 1.4 Hz, 1H, Ar-H), 7.19-7.17 (m, 1H, Ar-H), 7.05 (dd, *J* = 2.7, 1.4 Hz, 1H, Ar-H), 3.50-3.26 (m, 2H, overlapped with DMSO water, NCH_2), 3.25-3.12 (m, 6H, NCH_2×3), 1.65-1.51 (m, 6H, CH_2×3), 1.19-0.99 (m, 6H, CH_3×2) ppm. ^{13}C-NMR (101 MHz, DMSO-d_6) δ: 169.6, 167.2, 151.3, 138.4, 131.7, 116.8, 116.1, 115.9, 49.0, 25.0, 23.8 ppm. HRMS-ESI *(m/z)*: $[M+H]^+$ Calcd for $C_{17}H_{25}N_2O_3$ 305.1865; Found 305.1869.

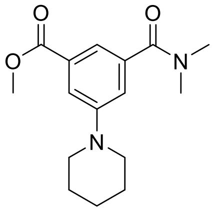

Methyl 3-(dimethylcarbamoyl)-5-(piperidin-1-yl)benzoate (14b).

The title compound was obtained as a colorless oil (130 mg, 91% yield) from monoester **13a** (130 mg, 0.49 mmol, 1.0 eq), dimethylamine (2M solution in THF, 0.40 mL, 0.79 mmol, 1.6 eq), HBTU (187 mg, 0.49 mmol, 1.0 eq) and TEA (0.14 mL, 0.99 mmol, 2.0 eq) by following general procedure B. Pure material was obtained by column chromatography using gradient elution from 20% EtOAc in petroleum ether to 50% EtOAc in petroleum ether.

^{1}H-NMR (400 MHz, $CDCl_3$) δ: 7.60 (dd, *J* = 2.6, 1.5 Hz, 1H. Ar-H), 7.45-7.42 (m, 1H, Ar-H), 7.12 (dd, *J* = 2.6, 1.5 Hz, 1H, Ar-H), 3.89 (s, 3H, OCH_3), 3.26-3.20 (m, 4H, NCH_2×2), 3.09 (s, 3H, NCH_3), 2.97 (s, 3H, NCH_3), 1.73-1.65 (m, 4H, CH_2×2), 1.62-1.56 (m, 2H, CH_2) ppm. ^{13}C-NMR (101 MHz, $CDCl_3$) δ: 171.4, 167.1, 152.2, 137.5, 131.0, 118.7, 117.9, 52.3, 50.0, 39.7, 35.4, 25.7, 24.3 ppm. HRMS-ESI *(m/z)*: $[M+H]^+$ Calcd for $C_{16}H_{23}N_2O_3$ 291.1709; Found 291.1706.

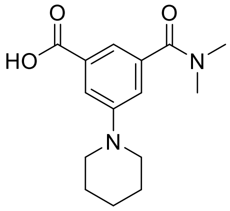

3-(Dimethylcarbamoyl)-5-(piperidin-1-yl)benzoic acid (15b).

The title compound was obtained as a yellow solid (44 mg, 40% yield) from benzoate **14b** (115 mg, 0.40 mmol, 1.0 eq) and aqueous 1 M NaOH solution (0.59 mL, 0.59 mmol, 1.5 eq) by following general procedure C. The crude product **15b** was used further without purification.

^{1}H-NMR (400 MHz, CD_3OD) δ: 7.65 (dd, *J* = 2.6, 1.4 Hz, 1H, Ar-H), 7.43-7.40 (m, 1H, Ar-H), 7.17 (dd, *J* = 2.6, 1.4 Hz, 1H, Ar-H), 3.28-3.23 (m, 4H, NCH_2×2), 3.10 (s, 3H, NCH_3), 3.00 (s, 3H, NCH_3), 1.76-1.68 (m, 4H, CH_2×2), 1.67-1.59 (m, 2H, CH_2) ppm. ^{13}C-NMR (101 MHz, CD_3OD) δ: 173.4,

169.5, 153.6, 138.4, 133.1, 119.5, 119.1, 118.9, 51.2, 40.0, 35.6, 26.6, 25.3 ppm. HRMS-ESI *(m/z)*: $[M+H]^+$ Calcd for $C_{15}H_{21}N_2O_3$ 277.1552; Found 277.1558.

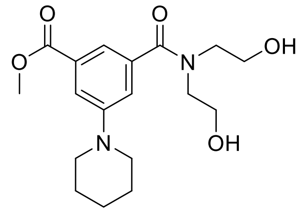

Methyl 3-(bis(2-hydroxyethyl)carbamoyl)-5-(piperidin-1-yl)benzoate (14c).

The title compound was obtained as a yellowish oil (174 mg, 64% yield) from monoester **13a** (205 mg, 0.78 mmol, 1.0 eq), diethanolamine (90 µL, 0.93 mmol, 1.2 eq), HBTU (295 mg, 0.78 mmol, 1.0 eq) and TEA (0.22 mL, 1.56 mmol, 2.0 eq) by following general procedure B. Pure material was obtained by column chromatography using gradient elution from 100% EtOAc to 10% MeOH in EtOAc.

^{1}H-NMR (400 MHz, $CDCl_3$) δ: 7.61-7.59 (m, 1H, Ar-H), 7.50-7.48 (m, 1H, Ar-H), 7.20-7.17 (m, 1H, Ar-H), 4.03-3.94 (m, 2H, NCH_2), 3.89 (s, 3H, OCH_3), 3.76-3.67 (m, 4H, NCH_2, OCH_2), 3.50-3.41 (m, 2H, OCH_2), 3.26-3.20 (m, 4H, NCH_2×2), 1.72-1.65 (m, 4H, CH_2×2), 1.63-1.55 (m, 2H, CH_2) ppm. ^{13}C-NMR (101 MHz, $CDCl_3$) δ: 173.5, 167.1, 152.1, 137.4, 131.2, 118.7, 117.9, 117.8, 61.4, 53.6, 52.4, 50.0, 25.6, 24.3 ppm. HRMS-ESI *(m/z)*: $[M+H]^+$ Calcd for $C_{18}H_{27}N_2O_5$ 351.1920; Found 351.1929.

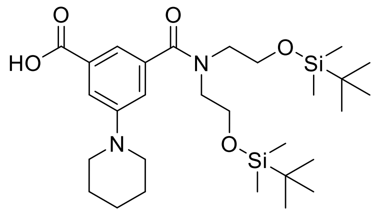

3-[Bis({2-[(*tert*-butyldimethylsilyl)oxy]ethyl})carbamoyl]-5-(piperidin-1-yl)benzoic acid (15c).

To a stirred solution of benzoate **14c** (173 mg, 0.49 mmol, 1.0 eq) in anhydrous DMF (6 mL) was added imidazole (202 mg, 2.96 mmol, 6.0 eq), followed by *tert*-butyldimethylsilyl chloride (223 mg, 1.48 mmol, 3.0 eq). After stirring at room temperature for 18 h water (20 mL) was added and the colorless solution was extracted with DCM (3 x 20 mL), combined organic layers were washed with brine (20 mL), dried over Na_2SO_4 and evaporated under reduced pressure. The residue was purified by column chromatography on silica gel using 30% EtOAc in petroleum ether to give a protected intermediate as a yellow oil (257 mg, 90% yield) that was used in the next step.

^{1}H-NMR (400 MHz, $CDCl_3$) δ: 7.66-7.57 (m, 1H, Ar-H), 7.49-7.41 (m, 1H, Ar-H), 7.13-7.03 (m, 1H, Ar-H), 3.92-3.86 (m, 5H, NCH_2, OCH_3), 3.74-3.61 (m, 4H, NCH_2, OCH_2), 3.53-3.45 (m, 2H, OCH_2), 3.26-3.19 (m, 4H, NCH_2×2), 1.76-1.65 (m, 4H, CH_2×2), 1.63-1.55 (m, 2H, CH_2), 0.91 (s, 9H, CH_3×3), 0.84 (s, 9H, CH_3×3), 0.09 (s, 6H, $SiCH_3$×2), -0.01 (s, 6H, $SiCH_3$×2) ppm. ^{13}C-NMR (101 MHz, $CDCl_3$) δ: 171.9, 167.1, 152.2, 138.1, 131.1, 118.4, 117.7, 117.6, 61.3, 52.3, 50.1, 48.4, 26.0, 25.8, 25.7, 24.3, 18.4, -3.4, -5.2, -5.3 ppm. HRMS-ESI *(m/z)*: $[M+H]^+$ Calcd for $C_{30}H_{55}N_2O_5Si_2$ 579.3650; Found 579.3636.

The title compound was obtained as a colorless oil (50 mg, 20% yield) from protected intermediate (253 mg, 0.44 mmol, 1.0 eq) and aqueous 1 M NaOH solution (0.66 mL, 0.66 mmol, 1.5 eq) by following general procedure C. Pure material was obtained by column chromatography using gradient elution from 30% EtOAc in petroleum ether to 100% EtOAc.

^{1}H-NMR (400 MHz, $CDCl_3$) δ: 7.64 (dd, *J* = 2.6, 1.4 Hz, 1H, Ar-H), 7.51 (dd, *J* = 1.4, 1.4 Hz, 1H, Ar-H), 7.13 (dd, *J* = 2.6, 1.4 Hz, 1H, Ar-H), 3.96-3.87 (m, 2H, NCH_2), 3.70 (t, *J* = 5.5 Hz, 2H, NCH_2), 3.65 (t, *J* = 6.1 Hz, 2H, OCH_2), 3.56-3.46 (m, 2H, OCH_2), 3.28-3.20 (m, 4H, NCH_2×2), 1.75-1.65 (m, 4H, CH_2×2), 1.64-1.56 (m, 2H, CH_2), 0.92 (s, 9H, CH_3×3), 0.85 (s, 9H, CH_3×3), 0.10 (s, 6H, $SiCH_3$×2), -0.01 (s, 6H, $SiCH_3$×2) ppm. ^{13}C-NMR (101 MHz, $CDCl_3$) δ: 171.8, 171.1, 152.2, 138.1, 130.3, 119.2, 118.3, 118.1, 61.2, 52.5, 50.1, 48.5, 29.8, 26.0, 25.7, 24.3, 18.4, -5.2, -5.3 ppm. HRMS-ESI *(m/z)*: $[M+H]^+$ Calcd for $C_{29}H_{53}N_2O_5Si_2$ 565.3493; Found 565.3477.

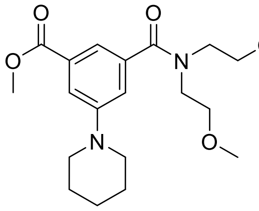

Methyl 3-[bis(2-methoxyethyl)carbamoyl]-5-(piperidin-1-yl)benzoate (14d).

The title compound was obtained as a yellowish oil (176 mg, 94% yield) from monoester **13a** (130 mg, 0.49 mmol, 1.0 eq), bis(2-methoxyethyl)amine (72 µL, 0.49 mmol, 1.0 eq), HBTU (187 mg, 0.49 mmol, 1.0 eq) and TEA (0.14 mL, 0.99 mmol, 2.0 eq) by following general procedure B. Pure material was obtained by column chromatography using gradient elution from 30% EtOAc in petroleum ether to 100% EtOAc.

^{1}H-NMR (400 MHz, $CDCl_3$) δ: 7.59 (dd, *J* = 2.6, 1.4 Hz, 1H, Ar-H), 7.47 (dd, *J* = 1.4, 1.4 Hz, 1H, Ar-H), 7.15 (dd, *J* = 2.6, 1.4 Hz, 1H, Ar-H), 3.89 (s, 3H, OCH_3), 3.78-3.71 (m, 2H, NCH_2), 3.69-3.63 (m, 2H, NCH_2), 3.55-3.48 (m, 2H, OCH_2), 3.47-3.41 (m, 2H, OCH_2), 3.38 (s, 3H, OCH_3), 3.31-3.19 (m, 7H, NCH_2×2, OCH_3), 1.74-1.66 (m, 4H, CH_2×2), 1.63-1.56 (m, 2H, CH_2) ppm. ^{13}C-NMR (101 MHz, $CDCl_3$) δ: 171.9, 166.8, 152.4, 136.4, 131.4, 118.3, 117.6, 116.6, 110.2, 52.5, 49.9, 25.6, 24.3 ppm. HRMS-ESI *(m/z)*: $[M+H]^+$ Calcd for $C_{20}H_{31}N_2O_5$ 379.2223; Found 379.2233.

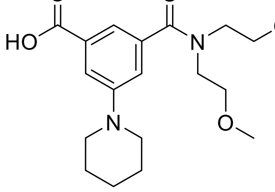

3-[Bis(2-methoxyethyl)carbamoyl]-5-(piperidin-1-yl)benzoic acid (15d).

The title compound was obtained as a reddish oil (151 mg, 96% yield) from benzoate **14d** (163 mg, 0.43 mmol, 1.0 eq) and aqueous 1 M NaOH solution (0.65 mL, 0.65 mmol, 1.5 eq) by following general procedure C. The crude product **15d** was used further without purification.

^{1}H-NMR (400 MHz, CD_3OD) δ: 7.63 (dd, *J* = 2.6, 1.4 Hz, 1H, Ar-H), 7.43 (dd, *J* = 1.4, 1.4 Hz, 1H, Ar-H), 7.22 (dd, *J* = 2.6, 1.4 Hz, 1H, Ar-H), 3.78-3.72 (m, 2H, NCH_2), 3.70-3.63 (m, 2H, NCH_2), 3.57-3.45 (m, 4H, OCH_2×2), 3.40 (s, 3H, OCH_3), 3.29-3.23 (m, 7H, NCH_2×2, OCH_3), 1.77-1.68 (m,

4H, CH_2×2), 1.67-1.60 (m, 2H, CH_2) ppm. ^{13}C-NMR (101 MHz, CD_3OD) δ: 174.3, 169.4, 153.3, 138.6, 133.0, 119.8, 119.1, 118.8, 71.3, 59.1, 51.2, 46.2, 26.6, 25.3 ppm. HRMS-ESI *(m/z)*: $[M+H]^+$ Calcd for $C_{19}H_{29}N_2O_5$ 365.2076; Found 365.2086.

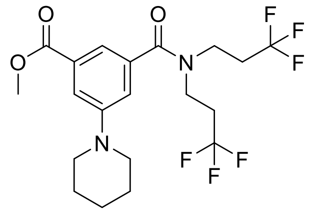

Methyl 3-[bis(3,3,3-trifluoropropyl)carbamoyl]-5-(piperidin-1 yl)benzoate (14e).

The title compound was obtained as a colorless oil (119 mg, 53% yield) from monoester **13a** (130 mg, 0.49 mmol, 1.0 eq), bis(3,3,3-trifluoropropyl)amine (103 mg, 0.49 mmol, 1.0 eq), HBTU (187 mg, 0.49 mmol, 1.0 eq) and TEA (0.14 mL, 0.99 mmol, 2.0 eq) by following general procedure B. Pure material was obtained by column chromatography using gradient elution from 10% EtOAc in petroleum ether to 50% EtOAc in petroleum ether.

^{1}H-NMR (400 MHz, $CDCl_3$) δ: 7.65 (dd, *J* = 2.6, 1.4 Hz, 1H, Ar-H), 7.35 (dd, *J* = 1.4, 1.4 Hz, 1H, Ar-H), 7.02 (dd, *J* = 2.6, 1.4 Hz, 1H, Ar-H), 3.91 (s, 3H, OCH_3), 3.72-3.51 (m, 4H, NCH_2×2), 3.29-3.21 (m, 4H, NCH_2×2), 2.70-2.22 (m, 4H, CH_2×2), 1.73-1.67 (m, 4H, CH_2×2), 1.65-1.57 (m, 2H, CH_2) ppm. ^{13}C-NMR (101 MHz, $CDCl_3$) δ: 172.0, 167.1, 151.9, 137.8, 131.0, 118.9, 118.0, 117.7, 110.2, 59.0, 52.3, 50.1, 25.7, 24.3 ppm. ^{19}F-NMR (376 MHz, $CDCl_3$) δ: -70.9, -72.8 ppm. HRMS-ESI *(m/z)*: $[M+H]^+$ Calcd for $C_{20}H_{25}N_2O_3F_6$ 455.1769; Found 455.1775.

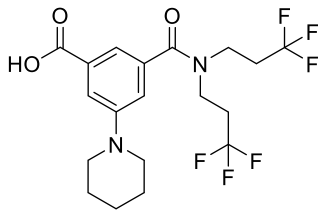

3-[Bis(3,3,3-trifluoropropyl)carbamoyl]-5-(piperidin-1-yl)benzoic acid (15e).

The title compound was obtained as a light yellow solid (107 mg, 99% yield) from benzoate **14e** (111 mg, 0.24 mmol, 1.0 eq) and aqueous 1 M NaOH solution (0.37 mL, 0.37 mmol, 1.5 eq) by following general procedure C. The crude product **15e** was used further without purification.

^{1}H-NMR (400 MHz, CD_3OD) δ: 7.68 (dd, *J* = 2.6, 1.4 Hz, 1H, Ar-H), 7.39 (dd, *J* = 1.4, 1.4 Hz, 1H, Ar-H), 7.14 (dd, *J* = 2.6, 1.4 Hz, 1H. Ar-H), 3.81-3.70 (m, 2H, NCH_2), 3.65-3.54 (m, 2H, NCH_2), 3.29-3.25 (m, 4H, NCH_2×2), 2.72-2.60 (m, 2H, CH_2), 2.58-2.47 (m, 2H, CH_2), 1.76-1.68 (m, 4H, CH_2×2), 1.67-1.60 (m, 2H, CH_2) ppm. ^{13}C-NMR (101 MHz, CD_3OD) δ: 174.0, 169.3, 153.6, 137.8, 133.4, 119.2, 118.7, 118.1, 51.0, 44.3, 40.2, 26.6, 25.3 ppm. HRMS-ESI *(m/z)*: $[M+H]^+$ Calcd for $C_{19}H_{23}N_2O_3F_6$ 441.1613; Found 441.1617.

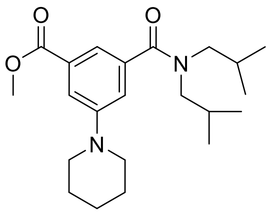

Methyl 3-(diisobutylcarbamoyl)-5-(piperidin-1-yl)benzoate (14f).
The title compound was obtained as a colorless oil (193 mg, 78% yield) from monoester **13a** (173 mg, 0.66 mmol, 1.0 eq), diisobutylamine (0.14 mL, 0.79 mmol, 1.2 eq), HBTU (249 mg, 0.66 mmol, 1.0 eq) and TEA (0.18 mL, 1.31 mmol, 2.0 eq) by following general procedure B. Pure material was obtained by column chromatography using gradient elution from 10% EtOAc in petroleum ether to 50% EtOAc in petroleum ether.

^{1}H-NMR (300 MHz, $CDCl_3$) δ: 7.59 (dd, *J* = 2.6, 1.4 Hz, 1H, Ar-H), 7.37 (dd, *J* = 1.4, 1.4 Hz, 1H, Ar-H), 7.05 (dd, *J* = 2.6, 1.4 Hz, 1H, Ar-H), 3.90 (s, 3H, OCH_3), 3.35 (d, *J* = 7.5 Hz, 2H, NCH_2), 3.26-3.18 (m, 4H, NCH_2×2), 3.09 (d, *J* = 7.5 Hz, 2H, NCH_2), 2.20-2.04 (m, 1H, CH), 1.96-1.77 (m, 1H, CH), 1.75-1.64 (m, 4H, CH_2×2), 1.64-1.55 (m, 2H, CH_2), 0.99 (d, *J* = 6.6 Hz, 6H, CH_3×2), 0.75 (d, *J* = 6.6 Hz, 6H, CH_3×2) ppm. ^{13}C-NMR (101 MHz, $CDCl_3$) δ: 172.2, 167.2, 152.3, 138.6, 130.9, 119.0, 118.0, 117.6, 56.6, 52.3, 51.2, 50.2, 26.9, 26.3, 25.7, 24.3, 20.4, 20.0 ppm. HRMS-ESI *(m/z)*: $[M+H]^+$ Calcd for $C_{22}H_{35}N_2O_3$ 375.2648; Found 375.2663.

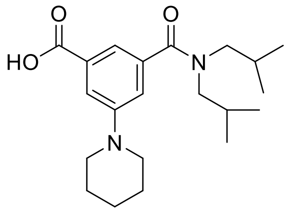

3-(Diisobutylcarbamoyl)-5-(piperidin-1-yl)benzoic acid (15f).
The title compound was obtained as a yellowish solid (176 mg, 100% yield) from benzoate **14f** (183 mg, 0.49 mmol, 1.0 eq) and aqueous 1 M NaOH solution (0.73 mL, 0.73 mmol, 1.5 eq) by following general procedure C. The crude product **15f** was used further without purification.

^{1}H-NMR (400 MHz, $CDCl_3$) δ: 7.64-7.61 (m, 1H, Ar-H), 7.45-7.41 (m, 1H, Ar-H), 7.10-7.05 (m, 1H, Ar-H), 3.35 (d, *J* = 7.5 Hz, 2H, NCH_2), 3.23 – 3.17 (m, 4H, NCH_2×2), 3.09 (d, *J* = 7.5 Hz, 2H, NCH_2), 2.16-2.04 (m, 1H, CH), 1.90-1.78 (m, 1H, CH), 1.71-1.64 (m, 4H, CH_2×2), 1.61-1.53 (m, 2H, CH_2), 0.97 (d, *J* = 6.6 Hz, 6H, CH_3×2), 0.73 (d, *J* = 6.6 Hz, 6H, CH_3×2) ppm. ^{13}C-NMR (101 MHz, $CDCl_3$) δ: 172.4, 152.2, 138.3, 119.1, 118.5, 118.2, 56.7, 51.3, 50.2, 26.9, 26.3, 25.7, 24.3, 20.4, 20.0 ppm. HRMS-ESI *(m/z)*: $[M+H]^+$ Calcd for $C_{21}H_{33}N_2O_3$ 361.2491; Found 361.2509.

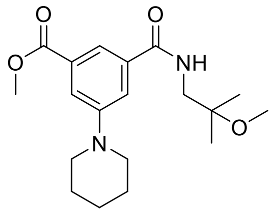

Methyl 3-[(2-methoxy-2-methylpropyl)carbamoyl]-5-(piperidin-1-yl)benzoate (14g).
The title compound was obtained as a colorless oil (169 mg, 85% yield) from monoester **13a** (150 mg, 0.57 mmol, 1.0 eq), 2-methoxy-2-methylpropan-1-amine (70 mg, 0.68 mmol, 1.2 eq), HBTU (216 mg, 0.57 mmol, 1.0 eq) and TEA (0.16 mL, 1.14 mmol, 2.0 eq) by following general procedure B. Pure material was obtained by column chromatography using gradient elution from 30% EtOAc in petroleum ether to 100% EtOAc.

^{1}H-NMR (400 MHz, $CDCl_3$) δ: 7.74-7.67 (m, 2H, Ar-H), 7.64-7.59 (m, 1H, Ar-H), 6.53-6.46 (m, 1H, NH), 3.92 (s, 3H, OCH_3), 3.49 (d, *J* = 5.6 Hz, 2H, NCH_2), 3.29-3.25 (m, 4H, NCH_2×2), 3.23 (s, 3H, OCH_3), 1.74-1.67 (m, 4H, CH_2×2), 1.64-1.56 (m, 2H, CH_2), 1.22 (s, 6H, CH_3×2) ppm. ^{13}C-NMR (101 MHz, $CDCl_3$) δ: 167.4, 167.0, 136.0, 131.2, 119.5, 116.9, 74.5, 52.4, 50.1, 49.6, 48.2, 25.7, 24.3, 22.7 ppm. HRMS-ESI *(m/z)*: $[M+H]^+$ Calcd for $C_{19}H_{29}N_2O_4$ 349.2127; Found 349.2118.

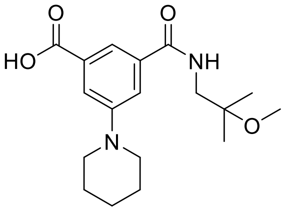

3-[(2-Methoxy-2-methylpropyl)carbamoyl]-5-(piperidin-1-yl)benzoic acid (15g).

The title compound was obtained as a brownish solid (49 mg, 33% yield) from benzoate **14g** (154 mg, 0.44 mmol, 1.0 eq) and aqueous 1M NaOH solution (0.66 mL, 0.66 mmol, 1.5 eq) by following general procedure C. The crude product **15g** was used further without purification.

^{1}H-NMR (400 MHz, CD_3OD) δ: 8.02-7.98 (m, 1H, Ar-H), 7.89-7.84 (m, 1H, Ar-H), 7.75-7.72 (m, 1H, Ar-H), 3.47 (s, 2H, CH_2), 3.39-3.34 (m, 4H, NCH_2×2), 3.28 (s, 3H, OCH_3), 1.85-1.76 (m, 4H, CH_2×2), 1.70-1.63 (m, 2H, CH_2), 1.22 (s, 6H, CH_3×2) ppm. ^{13}C-NMR (101 MHz, CD_3OD) δ: 169.7, 169.0, 137.4, 133.4, 121.8, 121.3, 76.5, 52.6, 49.9, 48.3, 26.4, 24.7, 23.2 ppm. HRMS-ESI *(m/z)*: $[M+H]^+$ Calcd for $C_{18}H_{27}N_2O_4$ 335.1971; Found 335.1961.

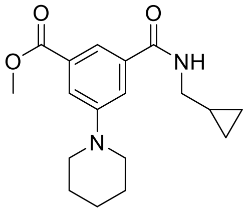

Methyl 3-[(cyclopropylmethyl)carbamoyl]-5-(piperidin-1-yl)benzoate (14h).

The title compound was obtained as a colorless oil (225 mg, 85% yield) from monoester **13a** (220 mg, 0.83 mmol, 1.0 eq), 1-cyclopropylmethanamine (87 μL, 1.00 mmol, 1.2 eq), HBTU (317 mg, 0.83 mmol, 1.0 eq) and TEA (0.23 mL, 1.67 mmol, 2.0 eq) by following general procedure B. Pure material was obtained by column chromatography using 50% EtOAc in petroleum ether.

^{1}H-NMR (400 MHz, $CDCl_3$) δ: 7.69-7.67 (m, 2H, Ar-H), 7.63 (dd, *J* = 2.7, 1.5 Hz, 1H, Ar-H), 6.33-6.25 (m, 1H, NH), 3.93 (s, 3H, OCH_3), 3.31 (dd, *J* = 7.2, 5.4 Hz, 2H, NCH_2), 3.29-3.25 (m, 4H, NCH_2×2), 1.74-1.66 (m, 4H, CH_2×2), 1.64-1.57 (m, 2H, CH_2), 1.13-1.01 (m, 1H, CH, cyclopropyl), 0.62-0.53 (m, 2H, CH_2, cyclopropyl), 0.33-0.24 (m, 2H, CH_2, cyclopropyl) ppm. ^{13}C-NMR (101 MHz, $CDCl_3$) δ: 167.2, 167.1, 152.4, 135.9, 131.1, 119.5, 119.4, 116.7, 52.4, 50.0, 45.2, 25.7, 24.3, 10.9, 3.7 ppm. HRMS-ESI *(m/z)*: $[M+H]^+$ Calcd for $C_{18}H_{25}N_2O_3$ 317.1865; Found 317.1873.

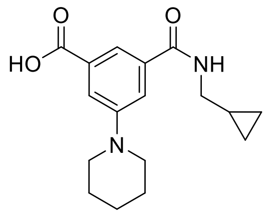

3-[(Cyclopropylmethyl)carbamoyl]-5-(piperidin-1-yl)benzoic acid (15h). The title compound was obtained as a white solid (177 mg, 84% yield) from benzoate **14h** (220 mg, 0.70 mmol, 1.0 eq) and aqueous 1 M NaOH solution (1.04 mL, 1.04 mmol, 1.5 eq) by following general procedure C. The crude product **15h** was used further without purification.

^{1}H-NMR (400 MHz, DMSO-d_6) δ: 8.66 (t, *J* = 5.7 Hz, 1H, NH), 7.83-7.80 (m, 1H, Ar-H), 7.61-7.58 (m, 1H, Ar-H), 7.54 (dd, *J* = 2.6, 1.4 Hz, 1H, Ar-H), 3.25-3.20 (m, 4H, NCH_2×2), 3.16-3.09 (m, 2H, NCH_2), 1.67-1.60 (m, 4H, CH_2×2), 1.59-1.53 (m, 2H, CH_2), 1.08-0.98 (m, 1H, CH, cyclopropyl), 0.47-0.38 (m, 2H, CH_2, cyclopropyl), 0.27-0.19 (m, 2H, CH_2, cyclopropyl) ppm. ^{13}C-NMR (101 MHz, DMSO-d_6) δ: 167.4, 165.7, 165.6, 151.5, 135.7, 131.7, 118.4, 118.0, 49.3, 43.6, 25.1, 23.8, 11.0, 3.4 ppm. HRMS-ESI *(m/z)*: $[M+H]^+$ Calcd for $C_{17}H_{23}N_2O_3$ 303.1709; Found 303.1720.

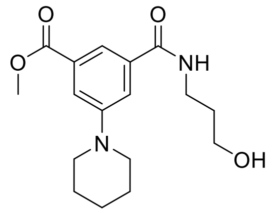

Methyl 3-[(3-hydroxypropyl)carbamoyl]-5-(piperidin-1-yl)benzoate (14i). The title compound was obtained as a light yellow oil (102 mg, 60% yield) from monoester **13a** (140 mg, 0.53 mmol, 1.0 eq), 3-amino-1-propanol (48 mg, 0.64 mmol, 1.2 eq), HBTU (202 mg, 0.53 mmol, 1.0 eq) and TEA (0.15 mL, 1.06 mmol, 2.0 eq) by following general procedure B. Pure material was obtained by column chromatography using gradient elution from 100% EtOAc to 2% MeOH in EtOAc.

^{1}H-NMR (400 MHz, $CDCl_3$) δ: 7.69-7.66 (m, 2H, Ar-H), 7.63-7.60 (m, 1H, Ar-H), 6.75-6.66 (m, 1H, NH), 3.91 (s, 3H, OCH_3), 3.70 (t, *J* = 5.7 Hz, 2H, NCH_2), 3.63 (q, *J* = 6.2 Hz, 2H, OCH_2), 3.29-3.24 (m, 4H, NCH_2×2), 3.21 (s, 1H, OH), 1.80 (quintet, *J* = 5.7 Hz, 2H, CH_2), 1.74-1.65 (m, 4H, CH_2×2), 1.64-1.56 (m, 2H, CH_2) ppm. ^{13}C-NMR (101 MHz, $CDCl_3$) δ: 168.4, 167.0, 152.4, 135.3, 131.2, 119.6, 119.3, 116.7, 59.6, 52.4, 50.0, 37.1, 32.4, 25.7, 24.3 ppm. HRMS-ESI *(m/z)*: $[M+H]^+$ Calcd for $C_{17}H_{25}N_2O_4$ 321.1814; Found 321.1824.

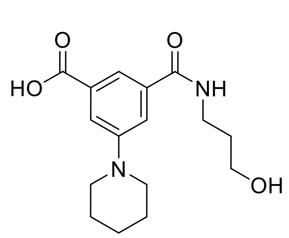

3-[(3-Hydroxypropyl)carbamoyl]-5-(piperidin-1-yl)benzoic acid (15i). The title compound was obtained as a yellow solid (84 mg, 94% yield) from benzoate **14i** (93 mg, 0.29 mmol, 1.0 eq) and aqueous 1 M NaOH solution (0.44 mL, 0.44 mmol, 1.5 eq) by following general procedure C. Pure material was obtained by the reversed-phase column chromatography (30 g KP-C18-HS column, flow rate 15 mL/min) using gradient elution from 100% water to 100% MeCN.

^{1}H-NMR (400 MHz, CD_3OD) δ: 8.61-8.59 (m, 1H, Ar-H), 8.49 (dd, *J* = 2.4, 1.5 Hz, 1H, Ar-H), 8.42 (dd, *J* = 2.4, 1.5 Hz, 1H, Ar-H), 3.78-3.71 (m, 4H, NCH_2×2), 3.67 (t, *J* = 6.2 Hz, 2H, NCH_2), 3.52 (t, *J* = 7.0 Hz, 2H, OCH_2), 2.15-2.06 (m, 4H, CH_2×2), 1.92-1.79 (m, 4H, CH_2×2) ppm. ^{13}C-NMR (101

MHz, CD_3OD) δ: 167.2, 166.8, 144.2, 138.8, 135.1, 130.5, 126.2, 125.7, 60.5, 58.3, 38.4, 33.1, 24.9, 22.0 ppm. HRMS-ESI *(m/z)*: $[M+H]^+$ Calcd for $C_{16}H_{23}N_2O_4$ 307.1658; Found 307.1656.

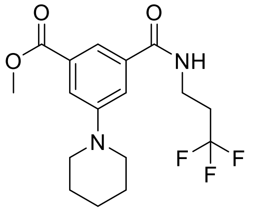

Methyl 3-(piperidin-1-yl)-5-[(3,3,3-trifluoropropyl)carbamoyl]benzoate (14j).

The title compound was obtained as a colorless oil (183 mg, 61% yield) from monoester **13a** (220 mg, 0.83 mmol, 1.0 eq), 3,3,3-trifluoropropylamine (113 mg, 1.00 mmol, 1.2 eq), HBTU (317 mg, 0.83 mmol, 1.0 eq) and TEA (0.23 mL, 1.67 mmol, 2.0 eq) by following general procedure B. Pure material was obtained by column chromatography using gradient elution from 30% EtOAc in petroleum ether to 50% EtOAc in petroleum ether.

^{1}H-NMR (400 MHz, $CDCl_3$) δ: 7.70 (dd, *J* = 2.7, 1.4 Hz, 1H, Ar-H), 7.64 (dd, *J* = 1.4, 1.4 Hz, 1H, Ar-H), 7.58 (dd, *J* = 2.7, 1.4 Hz, 1H, Ar-H), 6.47-6.40 (m, 1H, NH), 3.92 (s, 3H, OCH_3), 3.75-3.68 (m, 2H, NCH_2), 3.29-3.25 (m, 4H, NCH_2×2), 2.55-2.41 (m, 2H, CH_2), 1.74-1.66 (m, 4H, CH_2×2), 1.64-1.56 (m, 2H, CH_2) ppm. ^{13}C-NMR (101 MHz, $CDCl_3$) δ: 167.5, 166.9, 152.4, 135.2, 131.3, 119.8, 119.1, 116.6, 52.5, 50.0, 34.0, 33.8, 33.7, 25.7, 24.3 ppm. ^{19}F-NMR (376 MHz, $CDCl_3$) δ: -65.1 (t, *J* = 10.9 Hz) ppm. HRMS-ESI *(m/z)*: $[M+H]^+$ Calcd for $C_{17}H_{22}N_2O_3F_3$ 359.1583; Found 359.1586.

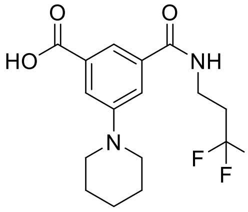

3-(Piperidin-1-yl)-5-[(3,3,3-trifluoropropyl)carbamoyl]benzoic acid (15j).

The title compound was obtained as a white solid (142 mg, 85% yield) from benzoate **14j** (173 mg, 0.48 mmol, 1.0 eq) and aqueous 1 M NaOH solution (0.72 mL, 0.72 mmol, 1.5 eq) by following general procedure C. The crude product **15j** was used further without purification.

^{1}H-NMR (400 MHz, DMSO-d_6) δ: 8.77 (t, *J* = 5.6 Hz, 1H, NH), 7.79 (dd, *J* = 1.4, 1.4 Hz, 1H, Ar-H), 7.58-7.54 (m, 2H, Ar-H), 3.52-3.45 (m, 2H, NCH_2), 3.25-3.20 (m, 4H, NCH_2×2), 2.62-2.51 (m, 2H, CH_2), 1.66-1.60 (m, 4H, CH_2×2), 1.59-1.52 (m, 2H, CH_2) ppm. ^{13}C-NMR (101 MHz, DMSO-d_6) δ: 167.3, 166.0, 151.5, 135.1, 128.2, 125.5, 118.2, 117.7, 49.2, 32.8, 32.6, 32.3, 25.0, 23.8 ppm. HRMS-ESI *(m/z)*: $[M+H]^+$ Calcd for $C_{16}H_{20}N_2O_3F_3$ 345.1426; Found 345.1440.

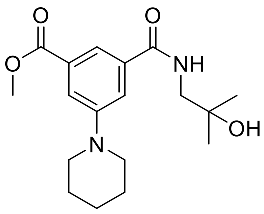

Methyl 3-[(2-hydroxy-2-methylpropyl)carbamoyl]-5-(piperidin-1-yl)benzoate (14k).

The title compound was obtained as a light yellow oil (194 mg, 80% yield) from monoester **13a** (190 mg, 0.72 mmol, 1.0 eq), 1-amino-2-methyl-2-propanol (77 mg, 0.87 mmol, 1.2 eq), HBTU (274 mg, 0.72 mmol, 1.0 eq) and TEA (0.20 mL, 1.44 mmol, 2.0 eq) by following general procedure B. Pure material was obtained by column

chromatography using gradient elution from 30% EtOAc in petroleum ether to 100% EtOAc, followed by 2% MeOH in EtOAc.

^{1}H-NMR (400 MHz, $CDCl_3$) δ: 7.72-7.70 (m, 1H, Ar-H), 7.69-7.67 (m, 1H, Ar-H), 7.64-7.60 (m, 1H, Ar-H), 6.74-6.66 (m, 1H, NH), 3.91 (s, 3H, OCH_3), 3.30-3.23 (m, 4H, NCH_2×2), 2.79 (d, J = 0.8 Hz, 2H, NCH_2), 2.52 (s, 1H, OH), 1.74-1.65 (m, 4H, CH_2×2), 1.63-1.56 (m, 2H, CH_2), 1.29 (s, 6H, CH_3×2) ppm. ^{13}C-NMR (101 MHz, $CDCl_3$) δ: 168.3, 167.0, 152.4, 135.5, 131.2, 119.6, 119.4, 116.8, 71.3, 52.4, 51.0, 50.0, 38.8, 27.6, 25.7, 24.3 ppm. HRMS-ESI *(m/z)*: $[M+H]^+$ Calcd for $C_{18}H_{27}N_2O_4$ 335.1971; Found 335.1978.

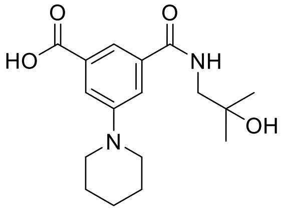

3-[(2-Hydroxy-2-methylpropyl)carbamoyl]-5-(piperidin-1-yl)benzoic acid (15k).

The title compound was obtained as a light yellow solid (139 mg, 77% yield) from benzoate **14k** (188 mg, 0.56 mmol, 1.0 eq) and aqueous 1 M NaOH solution (0.84 mL, 0.84 mmol, 1.5 eq) by following general procedure C. Pure material was obtained by the reversed-phase column chromatography (30 g KP-C18-HS column, flow rate 15 mL/min) using gradient elution from 100% water to 50% MeCN in water.

^{1}H-NMR (400 MHz, CD_3OD) δ: 7.90 (dd, J = 1.5, 1.5 Hz, 1H, Ar-H), 7.73 (dd, J = 2.6, 1.5 Hz, 1H, Ar-H), 7.62 (dd, J = 2.6, 1.5 Hz, 1H, Ar-H), 3.41 (s, 2H, NCH_2), 3.29-3.25 (m, 4H, NCH_2×2), 1.78-1.69 (m, 4H, CH_2×2), 1.67-1.59 (m, 2H, CH_2), 1.24 (s, 6H, CH_3×2) ppm. ^{13}C-NMR (101 MHz, CD_3OD) δ: 170.4, 169.5, 153.6, 136.8, 120.8, 120.3, 119.6, 72.0, 51.6, 51.3, 27.3, 26.7, 25.3 ppm. HRMS-ESI *(m/z)*: $[M+H]^+$ Calcd for $C_{17}H_{25}N_2O_4$ 321.1814; Found 321.1825.

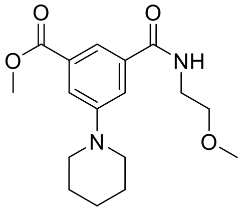

Methyl 3-[(2-methoxyethyl)carbamoyl]-5-(piperidin-1-yl)benzoate (14l).

The title compound was obtained as a colorless oil (156 mg, 99% yield) from monoester **13a** (130 mg, 0.49 mmol, 1.0 eq), 2-methoxyethylamine (47 μL, 0.54 mmol, 1.1 eq), HBTU (187 mg, 0.49 mmol, 1.0 eq) and TEA (0.14 mL, 0.99 mmol, 2.0 eq) by following general procedure B. Pure material was obtained by column chromatography using gradient elution from 30% EtOAc in petroleum ether to 100% EtOAc.

^{1}H-NMR (400 MHz, CD_3OD) δ: 7.86-7.84 (m, 1H, Ar-H), 7.70-7.68 (m, 1H, Ar-H), 7.62-7.59 (m, 1H, Ar-H), 3.91 (s, 3H, OCH_3), 3.58-3.55 (m, 4H, NCH_2, OCH_2), 3.38 (s, 3H, OCH_3), 3.29-3.24 (m, 4H, NCH_2×2), 1.77-1.69 (m, 4H, CH_2×2), 1.67-1.59 (m, 2H, CH_2) ppm. ^{13}C-NMR (101 MHz, CD_3OD) δ: 169.8, 168.3, 153.6, 136.8, 132.4, 120.4, 120.3, 119.2, 71.9, 58.9, 52.8, 51.2, 40.8, 26.7, 25.3 ppm. HRMS-ESI *(m/z)*: $[M+H]^+$ Calcd for $C_{17}H_{25}N_2O_4$ 321.1814; Found 321,1822.

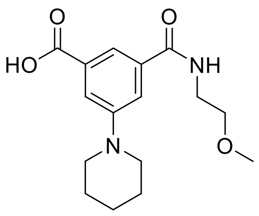

3-[(2-Methoxyethyl)carbamoyl]-5-(piperidin-1-yl)benzoic acid (15l).

The title compound was obtained as a dark red solid (85 mg, 57% yield) from benzoate **14l** (156 mg, 0.49 mmol, 1.0 eq) and aqueous 1 M NaOH solution (0.73 mL, 0.73 mmol, 1.5 eq) by following general procedure C. Pure material was obtained by the reversed-phase column chromatography (30 g KP-C18-HS column, flow rate 14 mL/min) using gradient elution from 100% water to 50% MeCN in water.

^{1}H-NMR (400 MHz, CD_3OD) δ: 7.87 (dd, *J* = 1.5, 1.5 Hz, 1H, Ar-H), 7.72 (dd, *J* = 2.6, 1.5 Hz, 1H, Ar-H), 7.60 (dd, *J* = 2.6, 1.5 Hz, 1H, Ar-H), 3.58-3.55 (m, 4H, NCH_2, OCH_2), 3.38 (s, 3H, OCH_3), 3.29-3.24 (m, 4H, NCH_2×2), 1.77-1.69 (m, 4H, CH_2×2), 1.67-1.59 (m, 2H, CH_2) ppm. ^{13}C-NMR (101 MHz, CD_3OD) δ: 170.0, 169.6, 153.6, 136.7, 133.1, 120.8, 120.2, 119.6, 71.9, 58.9, 51.3, 40.8, 26.7, 25.3 ppm. HRMS-ESI *(m/z)*: $[M+H]^+$ Calcd for $C_{16}H_{23}N_2O_4$ 307.1658; Found 307.1668.

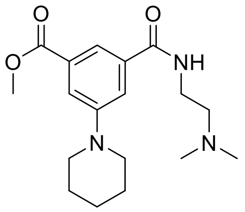

Methyl 3-{[2-(dimethylamino)ethyl]carbamoyl}-5-(piperidin-1-yl)benzoate (14m).

The title compound was obtained as a colorless oil (90 mg, 47% yield) from monoester **13a** (150 mg, 0.57 mmol, 1.0 eq), *N*,*N*-dimethylethylene-diamine (69 µL, 0.63 mmol, 1.1 eq), HBTU (216 mg, 0.57 mmol, 1.0 eq) and TEA (0.16 mL, 1.14 mmol, 2.0 eq) by following general procedure B. Pure material was obtained by column chromatography using gradient elution from 100% DCM to 6% MeOH in DCM.

^{1}H-NMR (400 MHz, $CDCl_3$) δ: 7.72-7.70 (m, 1H, Ar-H), 7.68-7.66 (m, 1H, Ar-H), 7.63-7.60 (m, 1H, Ar-H), 6.88-6.82 (m, 1H, NH), 3.91 (s, 3H, OCH_3), 3.56-3.50 (m, 2H, NCH_2), 3.29-3.23 (m, 4H, NCH_2×2), 2.56 (t, *J* = 5.9 Hz, 2H, NCH_2), 2.30 (s, 6H, CH_3×2), 1.74-1.66 (m, 4H, CH_2×2), 1.63-1.56 (m, 2H, CH_2) ppm. ^{13}C-NMR (101 MHz, $CDCl_3$) δ: 167.5, 167.1, 152.3, 135.8, 131.1, 119.5, 117.1, 58.0, 52.4, 50.1, 45.3, 37.5, 25.7, 24.3 ppm. HRMS-ESI *(m/z)*: $[M+H]^+$ Calcd for $C_{18}H_{28}N_3O_3$ 334.2131; Found 334.2132.

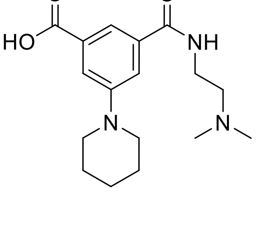

3-{[2-(Dimethylamino)ethyl]carbamoyl}-5-(piperidin-1-yl)benzoic acid (15m).

The title compound was obtained as a reddish solid (46 mg, 54% yield) from benzoate **14m** (89 mg, 0.27 mmol, 1.0 eq) and aqueous 1 M NaOH solution (0.40 mL, 0.40 mmol, 1.5 eq) by following general procedure C. Pure material was obtained by the reversed-phase column chromatography (30 g KP-C18-HS column, flow rate 25 mL/min) using gradient elution from 100% water to 50% MeCN in water.

^{1}H-NMR (400 MHz, CD_3OD) δ: 7.86-7.83 (m, 1H, Ar-H), 7.50-7.47 (m, 1H, Ar-H), 7.46 (dd, *J* = 2.6, 1.5 Hz, 1H, Ar-H), 3.85-3.79 (m, 2H, NCH_2), 3.39-3.33 (m, 2H, NCH_2), 3.18-3.12 (m, 4H, NCH_2×2), 2.89 (s, 6H, CH_3×2), 1.72-1.64 (m, 4H, CH_2×2), 1.62-1.56 (m, 2H, CH_2) ppm. ^{13}C-NMR (101 MHz, CD_3OD) δ: 170.9, 153.1, 135.2, 121.5, 119.9, 118.8, 58.0, 51.6, 43.4, 36.3, 26.8, 25.3 ppm. HRMS-ESI *(m/z)*: $[M+H]^+$ Calcd for $C_{17}H_{26}N_3O_3$ 320.1974; Found 320.1962.

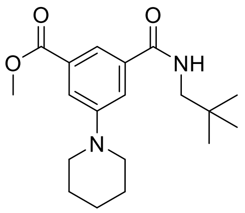

Methyl 3-(neopentylcarbamoyl)-5-(piperidin-1-yl)benzoate (14n).

The title compound was obtained as a colorless oil (148 mg, 78% yield) from monoester **13a** (150 mg, 0.57 mmol, 1.0 eq), 2,2-dimethylpropan-1-amine (74 µL, 0.63 mmol, 1.1 eq), HBTU (216 mg, 0.57 mmol, 1.0 eq) and TEA (0.16 mL, 1.14 mmol, 2.0 eq) by following general procedure B. Pure material was obtained by column chromatography using gradient elution from 10% EtOAc in petroleum ether to 20% EtOAc in petroleum ether.

^{1}H-NMR (400 MHz, $CDCl_3$) δ: 7.68 (dd, *J* = 2.7, 1.5 Hz, 1H, Ar-H), 7.65 (dd, *J* = 1.5, 1.5 Hz, 1H, Ar-H), 7.62 (dd, *J* = 2.7, 1.5 Hz, 1H, Ar-H), 6.25-6.18 (m, 1H, NH), 3.92 (s, 3H, OCH_3), 3.31-3.24 (m, 6H, NCH_2, NCH_2×2), 1.75-1.66 (m, 4H, CH_2×2), 1.64-1.57 (m, 2H, CH_2), 0.98 (s, 9H, CH_3×3) ppm. ^{13}C-NMR (101 MHz, $CDCl_3$) δ: 167.4, 167.1, 152.4, 136.2, 131.1, 119.5, 119.4, 116.4, 52.4, 51.2, 50.0, 32.4, 27.5, 25.7, 24.3 ppm. HRMS-ESI *(m/z)*: $[M+H]^+$ Calcd for $C_{19}H_{29}N_2O_3$ 333.2178; Found 333.2177.

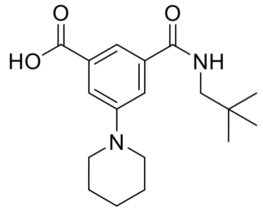

3-(Neopentylcarbamoyl)-5-(piperidin-1-yl)benzoic acid (15n).

The title compound was obtained as a white solid (133 mg, 99% yield) from benzoate **14n** (140 mg, 0.42 mmol, 1.0 eq) and aqueous 1 M NaOH solution (0.84 mL, 0.84 mmol, 2.0 eq) by following general procedure C. The crude product **15n** was used further without purification.

^{1}H-NMR (400 MHz, $CDCl_3$) δ: 7.75-7.71 (m, 2H, Ar-H), 7.68-7.65 (m, 1H, Ar-H), 6.44-6.35 (m, 1H, NH), 3.31-3.23 (m, 6H, NCH_2, NCH_2×2), 1.73-1.65 (m, 4H, CH_2×2), 1.63-1.56 (m, 2H, CH_2), 0.98 (s, 9H, CH_3×3) ppm. ^{13}C-NMR (101 MHz, $CDCl_3$) δ: 170.9, 167.6, 152.3, 136.2, 130.7, 120.2, 119.9, 117.1, 51.3, 50.0, 32.4, 27.5, 25.6, 24.3 ppm.

HRMS-ESI *(m/z)*: $[M+H]^+$ Calcd for $C_{18}H_{27}N_2O_3$ 319.2022; Found 319.2031.

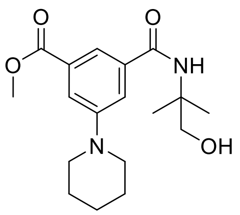

Methyl 3-[(1-hydroxy-2-methylpropan-2-yl)carbamoyl]-5-(piperidin-1-yl)benzoate (14o).

The title compound was obtained as a white solid (157 mg, 82% yield) from monoester **13a** (150 mg, 0.57 mmol, 1.0 eq), 2-amino-2-methyl-1-propanol (61

mg, 0.68 mmol, 1.2 eq), HBTU (216 mg, 0.57 mmol, 1.0 eq) and TEA (0.16 mL, 1.14 mmol, 2.0 eq) by following general procedure B. Pure material was obtained by column chromatography using gradient elution from 30% EtOAc in petroleum ether to 100% EtOAc.

^{1}H-NMR (400 MHz, $CDCl_3$) δ: 7.67 (dd, *J* = 2.7, 1.5 Hz, 1H, Ar-H), 7.60 (dd, *J* = 1.5, 1.5 Hz, 1H, Ar-H), 7.57 (dd, *J* = 2.7, 1.5 Hz, 1H, Ar-H), 6.25 (s, 1H, NH), 4.65 (s, 1H, OH), 3.92 (s, 3H, OCH_3), 3.70 (s, 2H, OCH_2), 3.30-3.23 (m, 4H, NCH_2×2), 1.74-1.66 (m, 4H, CH_2×2), 1.64-1.57 (m, 2H, CH_2), 1.42 (s, 6H, CH_3×2) ppm. ^{13}C-NMR (101 MHz, $CDCl_3$) δ: 168.2, 166.9, 152.3, 135.9, 131.1, 119.6, 119.3, 116.6, 70.8, 56.8, 52.5, 50.0, 25.6, 24.9, 24.3 ppm. HRMS-ESI *(m/z)*: $[M+H]^+$ Calcd for $C_{18}H_{27}N_2O_4$ 335.1971; Found 335.1974.

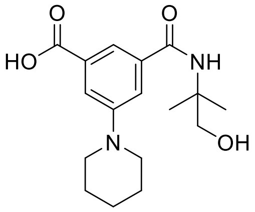

3-[(1-Hydroxy-2-methylpropan-2-yl)carbamoyl]-5-(piperidin-1-yl)benzoic acid (15o).

The title compound was obtained as an orange solid (94 mg, 66% yield) from benzoate **14o** (148 mg, 0.44 mmol, 1.0 eq) and aqueous 1 M NaOH solution (0.66 mL, 0.66 mmol, 1.5 eq) by following general procedure C. Pure material was obtained by the reversed-phase column chromatography (30 g KP-C18-HS column, flow rate 15 mL/min) using gradient elution from 100% water to 100% MeCN.

^{1}H-NMR (400 MHz, CD_3OD) δ: 7.78 (dd, *J* = 1.4, 1.4 Hz, 1H, Ar-H), 7.70 (dd, *J* = 2.7, 1.4 Hz, 1H, Ar-H), 7.52 (dd, *J* = 2.7, 1.4 Hz, 1H, Ar-H), 3.70 (s, 2H, CH_2), 3.28-3.23 (m, 4H, NCH_2×2), 1.77-1.68 (m, 4H, CH_2×2), 1.67-1.59 (m, 2H, CH_2), 1.41 (s, 6H, CH_3×2) ppm. ^{13}C-NMR (101 MHz, CD_3OD) δ: 170.5, 153.5, 138.1, 120.6, 120.2, 119.7, 69.2, 56.8, 51.4, 26.7, 25.3, 24.0, 23.2 ppm. HRMS-ESI *(m/z)*: $[M+H]^+$ Calcd for $C_{17}H_{25}N_2O_4$ 321.1814; Found 321.1814.

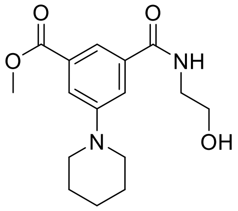

Methyl 3-[(2-hydroxyethyl)carbamoyl]-5-(piperidin-1-yl)benzoate (14p).

The title compound was obtained as a yellowish oil (97 mg, 52% yield) from monoester **13a** (160 mg, 0.61 mmol, 1.0 eq), ethanolamine (42 µL, 0.70 mmol, 1.2 eq), HBTU (230 mg, 0.61 mmol, 1.0 eq) and TEA (0.17 mL, 1.22 mmol, 2.0 eq) by following general procedure B. Pure material was obtained by column chromatography using gradient elution from 100% EtOAc to 5% MeOH in EtOAc.

^{1}H-NMR (400 MHz, $CDCl_3$) δ: 7.71-7.69 (m, 1H, Ar-H), 7.68-7.66 (m, 1H, Ar-H), 7.62-7.60 (m, 1H, Ar-H), 6.84-6.77 (m, 1H, NH), 3.90 (s, 3H, OCH_3), 3.84 (t, *J* = 5.4 Hz, 2H, OCH_2), 3.63 (quartet, *J* = 5.4 Hz, 2H, NCH_2), 3.28-3.23 (m, 4H, NCH_2×2), 1.74-1.66 (m, 4H, CH_2×2), 1.64-1.55 (m, 2H, CH_2) ppm. ^{13}C-NMR (101 MHz, $CDCl_3$) δ: 168.4, 167.0, 152.3, 135.3, 131.1, 119.6, 119.4, 117.0, 62.6, 52.4, 50.0, 43.1, 25.6, 24.3 ppm. HRMS-ESI *(m/z)*: $[M+H]^+$ Calcd for $C_{16}H_{23}N_2O_4$ 307.1658; Found 307.1672.

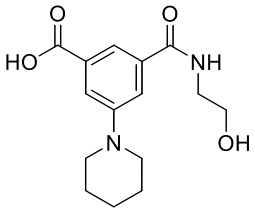

3-[(2-Hydroxyethyl)carbamoyl]-5-(piperidin-1-yl)benzoic acid (15p).

The title compound was obtained as a pink solid (67 mg, 85% yield) from benzoate **14p** (83 mg, 0.27 mmol, 1.0 eq) and aqueous 1 M NaOH solution (0.41 mL, 0.41 mmol, 1.5 eq) by following general procedure C. Pure material was obtained by the reversed-phase column chromatography (30 g KP-C18-HS column, flow rate 15 mL/min) using gradient elution from 100% water to 100% MeCN.

^{1}H-NMR (400 MHz, CD_3OD) δ: 7.89 (dd, *J* = 1.5, 1.5 Hz, 1H, Ar-H), 7.71 (dd, *J* = 2.6, 1.5 Hz, 1H, Ar-H), 7.62 (dd, *J* = 2.6, 1.5 Hz, 1H, Ar-H), 3.72 (t, *J* = 5.8 Hz, 2H, OCH_2), 3.51 (t, *J* = 5.8 Hz, 2H, NCH_2), 3.29-3.24 (m, 4H, NCH_2×2), 1.77-1.68 (m, 4H, CH_2×2), 1.67-1.59 (m, 2H, CH_2) ppm. ^{13}C-NMR (101 MHz, CD_3OD) δ: 170.2, 169.6, 153.6, 136.8, 133.1, 120.8, 120.2, 119.7, 61.6, 51.3, 43.6, 26.7, 25.3 ppm. HRMS-ESI *(m/z)*: $[M+H]^+$ Calcd for $C_{15}H_{21}N_2O_4$ 293.1501; Found 293.1510.

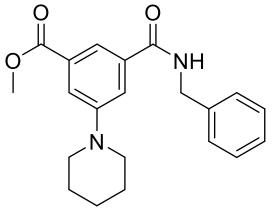

Methyl 3-(benzylcarbamoyl)-5-(piperidin-1-yl)benzoate (14q).

The title compound was obtained as a colorless oil (233 mg, 79% yield) from monoester **13a** (220 mg, 0.84 mmol, 1.0 eq), benzylamine (0.11 mL, 1.00 mmol, 1.2 eq), HBTU (317 mg, 0.84 mmol, 1.0 eq) and TEA (0.23 mL, 1.67 mmol, 2.0 eq) by following general procedure B. Pure material was obtained by column chromatography using gradient elution from 50% EtOAc in petroleum ether to 100% EtOAc.

^{1}H-NMR (400 MHz, CD_3OD) δ: 7.89 (dd, *J* = 1.5, 1.5 Hz, 1H, Ar-H), 7.70 (dd, *J* = 2.6, 1.5 Hz, 1H, Ar-H), 7.63 (dd, *J* = 2.6, 1.5 Hz, 1H, Ar-H), 7.37-7.30 (m, 4H, Ar-H), 7.27-7.22 (m, 1H, Ar-H), 4.57 (s, 2H, Ar-CH_2), 3.91 (s, 3H, OCH_3), 3.28-3.24 (m, 4H, NCH_2×2), 1.75-1.68 (m, 4H, CH_2×2), 1.66-1.59 (m, 2H, CH_2) ppm. ^{13}C-NMR (101 MHz, CD_3OD) δ: 169.6, 168.3, 153.7, 140.2, 136.8, 132.4, 129.5, 128.6, 128.2, 120.5, 120.3, 119.2, 52.8, 51.2, 44.6, 26.7, 25.2 ppm. HRMS-ESI *(m/z)*: $[M+H]^+$ Calcd for $C_{21}H_{25}N_2O_3$ 353.1865; Found 353.1867.

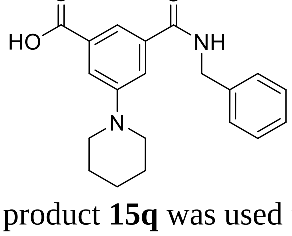

3-(Benzylcarbamoyl)-5-(piperidin-1-yl)benzoic acid (15q).

The title compound was obtained as a white solid (175 mg, 78% yield) from benzoate **14q** (233 mg, 0.66 mmol, 1.0 eq) and aqueous 1 M NaOH solution (0.99 mL, 0.99 mmol, 1.5 eq) by following general procedure C. The crude product **15q** was used further without purification.

^{1}H-NMR (400 MHz, DMSO-d_6) δ: 9.14 (t, *J* = 6.0 Hz, 1H, NH), 7.86 (dd, *J* = 1.4, 1.4 Hz, 1H, Ar-H), 7.65 (dd, *J* = 2.6, 1.4 Hz, 1H, Ar-H), 7.56 (dd, *J* = 2.6, 1.4 Hz, 1H, Ar-H), 7.36-7.29 (m, 4H, Ar-H), 7.27-7.21 (m, 1H, Ar-H), 4.47 (d, *J* = 6.0 Hz, 2H, Ar-CH_2), 3.25-3.20 (m, 4H, NCH_2×2), 1.66-1.60 (m, 4H, CH_2×2), 1.58-1.52 (m, 2H, CH_2) ppm. ^{13}C-NMR (101 MHz, DMSO-d_6) δ: 167.3, 165.8,

151.5, 139.6, 135.3, 131.7, 128.3, 127.3, 126.8, 118.4, 118.1, 118.0, 49.2, 42.7, 25.0, 23.8 ppm. HRMS-ESI *(m/z)*: [M+H]$^+$ Calcd for $C_{20}H_{23}N_2O_3$ 339.1709; Found 339.1715.

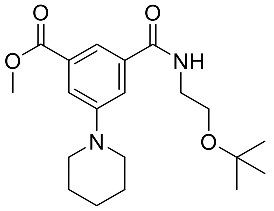

Methyl 3-{[2-(*tert*-butoxy)ethyl]carbamoyl}-5-(piperidin-1-yl)benzoate (14r).

The title compound was obtained as a colorless oil (153 mg, 85% yield) from monoester **13a** (130 mg, 0.49 mmol, 1.0 eq), 2-*tert*-butoxyethan-1-amine (64 µL, 0.54 mmol, 1.1 eq), HBTU (187 mg, 0.49 mmol, 1.0 eq) and TEA (0.14 mL, 0.99 mmol, 2.0 eq) by following general procedure B. Pure material was obtained by column chromatography using gradient elution from 15% EtOAc in petroleum ether to 50% EtOAc in petroleum ether.

^{1}H-NMR (400 MHz, $CDCl_3$) δ: 7.70-7.67 (m, 2H, Ar-H), 7.60 (dd, *J* = 2.7, 1.6 Hz, 1H, Ar-H), 6.64-6.55 (m, 1H, Ar-H), 3.91 (s, 3H, OCH_3), 3.63-3.57 (m, 2H, OCH_2), 3.56-3.51 (m, 2H, NCH_2), 3.30-3.24 (m, 4H, NCH_2×2), 1.74-1.66 (m, 4H, CH_2×2), 1.64-1.57 (m, 2H, CH_2), 1.21 (s, 9H, CH_3×3) ppm. ^{13}C-NMR (101 MHz, $CDCl_3$) δ: 167.2, 167.0, 152.3, 136.0, 131.2, 119.4, 119.3, 116.9, 73.4, 60.6, 52.4, 50.0, 40.6, 27.7, 25.7, 24.3 ppm. HRMS-ESI *(m/z)*: [M+H]$^+$ Calcd for $C_{20}H_{31}N_2O_4$ 363.2284; Found 363.2292.

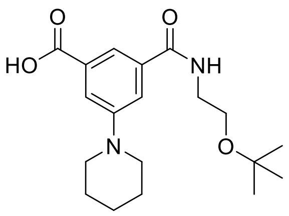

3-{[2-(*tert*-Butoxy)ethyl]carbamoyl}-5-(piperidin-1-yl)benzoic acid (15r).

The title compound was obtained as a pink solid (107 mg, 84% yield) from benzoate **14r** (133 mg, 0.37 mmol, 1.0 eq) and aqueous 1 M NaOH solution (0.55 mL, 0.55 mmol, 1.5 eq) by following general procedure C. Pure material was obtained by the reversed-phase column chromatography (30 g KP-C18-HS column, flow rate 14 mL/min) using gradient elution from 100% water to 50% MeCN in water.

^{1}H-NMR (400 MHz, CD_3OD) δ: 8.48-8.43 (m, 1H, Ar-H), 8.39-8.33 (m, 1H, Ar-H), 8.30-8.24 (m, 1H, Ar-H), 3.67-3.62 (m, 4H, NCH_2×2), 3.61-3.57 (m, 2H, OCH_2), 3.55-3.50 (m, 2H, NCH_2), 2.10-2.00 (m, 4H, CH_2×2), 1.84-1.74 (m, 2H, CH_2), 1.21 (s, 9H, CH_3×3) ppm. ^{13}C-NMR (101 MHz, CD_3OD) δ: 138.4, 134.7, 74.5, 61.3, 42.1, 27.8, 25.2 ppm. HRMS-ESI *(m/z)*: [M+H]$^+$ Calcd for $C_{19}H_{29}N_2O_4$ 349.2127; Found 349.2128.

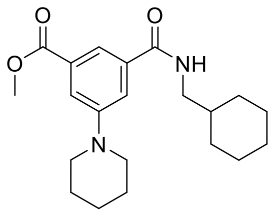

Methyl 3-[(cyclohexylmethyl)carbamoyl]-5-(piperidin-1-yl)benzoate (14s).

The title compound was obtained as a colorless oil (156 mg, 76% yield) from monoester **13a** (150 mg, 0.57 mmol, 1.0 eq), cyclohexanemethylamine (77 mg, 0.68 mmol, 1.2 eq), HBTU (216 mg, 0.57 mmol, 1.0 eq) and TEA (0.16 mL, 1.14 mmol, 2.0 eq)

by following general procedure B. Pure material was obtained by column chromatography using gradient elution from 20% EtOAc in petroleum ether to 35% EtOAc in petroleum ether.

^{1}H-NMR (400 MHz, $CDCl_3$) δ: 7.67 (dd, *J* = 2.6, 1.5 Hz, 1H, Ar-H), 7.65 (dd, *J* = 1.5, 1.5 Hz, 1H, Ar-H), 7.62 (dd, *J* = 2.6, 1.5 Hz, 1H, Ar-H), 6.28-6.21 (m, 1H, NH), 3.92 (s, 3H, OCH_3), 3.33-3.24 (m, 6H, NCH_2×2, Cy- CH_2), 1.82-1.52 (m, 12H, CH_2×6), 1.32-1.12 (m, 3H, CH, CH_2), 1.06-0.92 (m, 2H, CH_2) ppm. ^{13}C-NMR (101 MHz, $CDCl_3$) δ: 167.3, 167.1, 152.4, 136.1, 131.1, 119.5, 119.4, 116.6, 52.4, 50.0, 46.5, 38.2, 31.1, 26.5, 26.0, 25.7, 24.3 ppm. HRMS-ESI *(m/z)*: $[M+H]^+$ Calcd for $C_{21}H_{31}N_2O_3$ 359.2335; Found 359.2349.

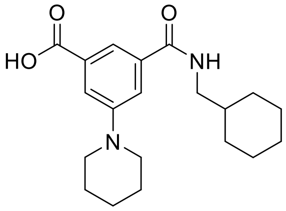

3-[(Cyclohexylmethyl)carbamoyl]-5-(piperidin-1-yl)benzoic acid (15s). The title compound was obtained as a light yellow solid (137 mg, 98% yield) from benzoate **14s** (146 mg, 0.41 mmol, 1.0 eq) and aqueous 1 M NaOH solution (0.61 mL, 0.61 mmol, 1.5 eq) by following general procedure C. The crude product **15s** was used further without purification.

^{1}H-NMR (400 MHz, CD_3OD) δ: 7.87 (dd, *J* = 1.5, 1.5 Hz, 1H, Ar-H), 7.71 (dd, *J* = 2.6, 1.5 Hz, 1H, Ar-H), 7.59 (dd, *J* = 2.6, 1.5 Hz, 1H, Ar-H), 3.29-3.24 (m, 4H, NCH_2×2), 3.21 (d, *J* = 7.0 Hz, 2H, Cy- CH_2), 1.83-1.58 (m, 12H, CH_2×6), 1.37-1.15 (m, 3H, CH, CH_2), 1.07-0.94 (m, 2H, CH_2) ppm. ^{13}C-NMR (101 MHz, CD_3OD) δ: 170.0, 169.6, 153.6, 137.1, 133.0, 120.7, 120.2, 119.6, 51.3, 47.4, 39.2, 32.1, 27.6, 27.0, 26.7, 25.3 ppm. HRMS-ESI *(m/z)*: $[M+H]^+$ Calcd for $C_{20}H_{29}N_2O_3$ 345.2178; Found 345.2178.

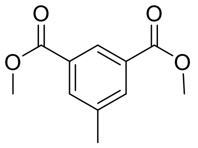

Dimethyl 5-methylisophthalate (12b).
An oven-dried 20 mL pressure vial was cooled under stream of argon and charged with dimethyl 5-bromoisophthalate (500 mg, 1.83 mmol, 1.0 eq), methylboronic acid (132 mg, 2.20 mmol, 1.2 eq), potassium phosphate (1.17 g, 5.49 mmol, 3.0 eq), $Pd(dppf)Cl_2{\cdot}CH_2Cl_2$ (75 mg, 0.092 mmol, 0.05 eq) and anhydrous toluene (7 mL). After strirring at 90 °C for 18 h, the brown suspension was cooled to room temperature and filtered through Celite. The filter plug was washed with EtOAc and filtrate was dried over anhydrous Na_2SO_4. Volatiles were evaporated under reduced pressure. The crude product was purified by column chromatography on silica gel using 5% EtOAc in hexanes to give product **12b** as a white solid (305 mg, 80% yield). ^{1}H-NMR spectrum was identical to that from the literature.[5]

^{1}H-NMR (400 MHz, $CDCl_3$) δ: 8.51-8.46 (m, 1H, Ar-H), 8.07-8.01 (m, 2H, Ar-H), 3.93 (s, 6H, OCH_3×2), 2.45 (s, 3H, Ar-CH_3) ppm.

---

[5] Sivakumar, C.; Sultan, N. *Journal of Polymer Science: Part A: Polymer Chemistry.* **2009**, *47*, 3337-3351.

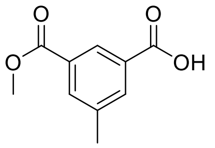

3-(Methoxycarbonyl)-5-methylbenzoic acid (13b).

The title compound was obtained as a white solid material (266 mg, 97% yield) from diester **12b** (295 mg, 1.42 mmol, 1.0 eq) and aqueous 1M NaOH solution (1.42 mL, 1.42 mmol, 1.0 eq) by following general procedure A. The crude product **13b** was used in subsequent step without purification. ^{1}H-NMR spectrum was identical to that from the literature.[6]

^{1}H-NMR (400 MHz, CD_3OD) δ: 8.46-8.41 (m, 1H, Ar-H), 8.07-8.03 (m, 2H, Ar-H), 3.93 (s, 3H, OCH_3), 2.46 (s, 3H, Ar-CH_3) ppm. HRMS-ESI *(m/z)*: $[M-H]^-$ Calcd for $C_{10}H_9O_4$ 193.0501; Found 193.0502.

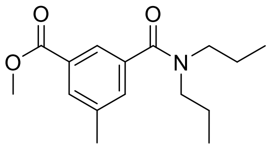

Methyl 3-(dipropylcarbamoyl)-5-methylbenzoate (16b).

The title compound was obtained as a colorless oil (246 mg, 66% yield) from monoester **13b** (261 mg, 1.34 mmol, 1.0 eq), dipropylamine (0.22 mL, 1.61 mmol, 1.2 eq), HBTU (510 mg, 1.34 mmol, 1.0 eq) and TEA (0.37 mL, 2.69 mmol, 2.0 eq) by following general procedure B. Pure material was obtained by column chromatography using 20% EtOAc in petroleum ether.

^{1}H-NMR (400 MHz, $CDCl_3$) δ: 7.88-7.86 (m, 1H, Ar-H), 7.81-7.79 (m, 1H, Ar-H), 7.38-7.35 (m, 1H, Ar-H), 3.91 (s, 3H, OCH_3), 3.49-3.40 (m, 2H, NCH_2), 3.19-3.10 (m, 2H, NCH_2), 2.41 (s, 3H, Ar-CH_3), 1.76-1.62 (m, 2H, CH_2), 1.58-1.46 (m, 2H, CH_2), 0.97 (t, J = 7.6 Hz, 3H, CH_3), 0.74 (t, J = 7.6 Hz, 3H, CH_3) ppm. HRMS-ESI *(m/z)*: $[M+H]^+$ Calcd for $C_{16}H_{24}NO_3$ 278.1756; Found 278.1758.

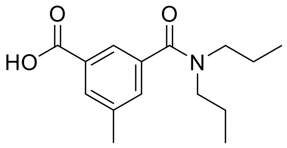

3-(Dipropylcarbamoyl)-5-methylbenzoic acid (17b).

The title compound was obtained as a white solid (227 mg, 100% yield) from benzoate **16b** (240 mg, 0.86 mmol, 1.0 eq) and aqueous 1 M NaOH solution (1.30 mL, 1.30 mmol, 1.5 eq) by following general procedure C. The crude product **17b** was used further without purification.

^{1}H-NMR (400 MHz, $CDCl_3$) δ: 7.95-7.92 (m, 1H, Ar-H), 7.89-7.86 (m, 1H, Ar-H), 7.44-7.42 (m, 1H, Ar-H), 6.48-6.07 (br s, 1H, COOH), 3.55-3.39 (m, 2H, NCH_2), 3.24-3.10 (m, 2H, NCH_2), 2.43 (s, 3H, Ar-CH_3), 1.77-1.65 (m, 2H, CH_2), 1.62-1.48 (m, 2H, CH_2), 0.99 (t, J = 7.6 Hz, 3H, CH_3), 0.76 (t, J = 7.6 Hz, 3H, CH_3) ppm. ^{13}C-NMR (101 MHz, $CDCl_3$) δ: 171.0, 170.7, 139.1, 137.7, 132.7, 131.5, 129.6, 125.4, 51.0, 46.7, 22.1, 21.4, 20.8, 11.6, 11.2 ppm. HRMS-ESI *(m/z)*: $[M+H]^+$ Calcd for $C_{15}H_{22}NO_3$ 264.1600; Found 264.1606.

[6] Gosh, A. K.; Takayama, J.; Kassekert, L. A.; Ella-Menye, J. R.; Yashchuk, S.; Agniswamy, J.; Wang, Y. F.; Aoki, M.; Amano, M.; Weber, I. T.; Mitsuya, H. *Bioorg. Med. Chem. Lett.* **2015**, *25*, 4903-4909.

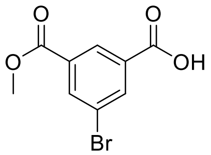

3-Bromo-5-(methoxycarbonyl)benzoic acid (13c).

The title compound was obtained as a white solid material (568 mg, 100% yield) from dimethyl 5-bromoisophthalate (600 mg, 2.20 mmol, 1.0 eq) and aqueous 1 M NaOH solution (2.20 mL, 2.20 mmol, 1.0 eq) by following general procedure A. The crude product **13c** was used in subsequent step without purification. ^{1}H-NMR spectra was identical to that from the literature.[7]

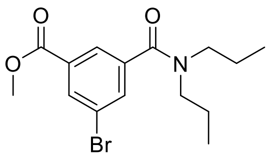

Methyl 3-bromo-5-(dipropylcarbamoyl)benzoate (16c).

The title compound was obtained as a colorless oil (484 mg, 64% yield) from monoester **13c** (568 mg, 2.19 mmol, 1.0 eq), dipropylamine (0.36 mL, 2.63 mmol, 1.2 eq), HBTU (831 mg, 2.19 mmol, 1.0 eq) and TEA (0.61 mL, 4.38 mmol, 2.0 eq) by following general procedure B. Pure material was obtained by column chromatography using 10% EtOAc in petroleum ether.

^{1}H-NMR (400 MHz, $CDCl_3$) δ: 8.20-8.18 (m, 1H, Ar-H), 7.95-7.93 (m, 1H, Ar-H), 7.70-7.67 (m, 1H, Ar-H), 3.93 (s, 3H, OCH_3), 3.45 (t, J = 7.5 Hz, 2H, NCH_2), 3.14 (t, J = 7.5 Hz, 2H, NCH_2), 1.75-1.62 (m, 2H, CH_2), 1.60-1.49 (m, 2H, CH_2), 0.98 (t, J = 7.5 Hz, 3H, CH_3), 0.77 (t, J = 7.5 Hz, 3H, CH_3) ppm. ^{13}C-NMR (101 MHz, $CDCl_3$) δ: 169.1, 165.3, 139.5, 134.0, 133.2, 132.2, 126.3, 122.8, 52.8, 50.9, 46.7, 22.1, 20.8, 11.6, 11.2 ppm. HRMS-ESI *(m/z)*: $[M+H]^+$ Calcd for $C_{15}H_{21}NO_3Br$ 342.0705; Found 342.0691.

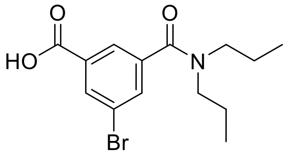

3-Bromo-5-(dipropylcarbamoyl)benzoic acid (17c).

The title compound was obtained as a colorless oil (454 mg, 100% yield) from benzoate **16c** (473 mg, 1.38 mmol, 1.0 eq) and aqueous 1 M NaOH solution (2.07 mL, 2.07 mmol, 1.5 eq) by following general procedure C. The crude product **17c** was used further without purification.

^{1}H-NMR (400 MHz, $CDCl_3$) δ: 10.83 (s, 1H, COOH), 8.26-8.23 (m, 1H, Ar-H), 8.02-7.99 (m, 1H, Ar-H), 7.76-7.73 (m, 1H, Ar-H), 3.47 (t, J = 7.7 Hz, 2H, NCH_2), 3.16 (t, J = 7.7 Hz, 2H, NCH_2), 1.77-1.64 (m, 2H, CH_2), 1.62-1.49 (m, 2H, CH_2), 0.99 (t, J = 7.4 Hz, 3H, CH_3), 0.78 (t, J = 7.4 Hz, 3H, CH_3) ppm. ^{13}C-NMR (101 MHz, $CDCl_3$) δ: 169.3, 168.9, 139.3, 134.7, 133.8, 131.7, 126.8, 122.9, 51.0, 46.9, 22.1, 20.8, 11.6, 11.2 ppm. HRMS-ESI *(m/z)*: $[M+H]^+$ Calcd for $C_{14}H_{19}NO_3Br$ 328.0548; Found 328.0557.

[7] Choi, K.; Hamilton, A. D. *J. Am. Chem. Soc.* **2003**, *125*, 10241–10249.

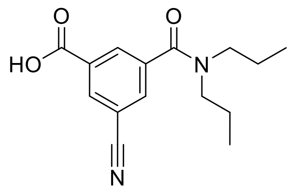

3-Cyano-5-(dipropylcarbamoyl)benzoic acid (17i).

Copper(I) cyanide (82 mg, 0.92 mmol, 2.0 equiv) was added to a solution of benzoic acid **17c** (150 mg, 0.46 mmol, 1.0 equiv) in *N*-methyl-2-pyrrolidone (1 mL). After 6 h of stirring at 160 °C, the colorless solution was cooled to room temperature and aqueous 2N HCl solution (10 mL) and EtOAc (15 mL) were added. The organic layer was washed with an aqueous 2N HCl solution (2 x 10 mL), then brine (2 x 10 mL), dried over Na_2SO_4 and evaporated under reduced pressure. The residue was purified by the reversed-phase column chromatography (30 g KP-C18-HS column, flow rate 18 mL/min) using gradient elution from 100% water to 100% MeCN to give product **17i** as a brown oil (20 mg, 16% yield).

^{1}H-NMR (400 MHz, $CDCl_3$) δ: 8.62 (s, 1H, COOH), 8.39-8.34 (m, 1H, Ar-H), 8.29-8.25 (m, 1H, Ar-H), 7.89-7.84 (m, 1H, Ar-H), 3.49 (t, *J* = 7.7 Hz, 2H, NCH_2), 3.14 (t, *J* = 7.7 Hz, 2H, NCH_2), 1.72 (sextet, *J* = 7.4 Hz, 2H, CH_2), 1.56 (sextet, *J* = 7.4 Hz, 2H, CH_2), 0.99 (t, *J* = 7.4 Hz, 3H, CH_3), 0.77 (t, *J* = 7.4 Hz, 3H, CH_3) ppm. ^{13}C-NMR (101 MHz, $CDCl_3$) δ: 168.8, 167.4, 138.7, 134.5, 134.3, 132.1, 117.2, 113.7, 51.1, 47.1, 22.1, 20.8, 11.6, 11.2 ppm. IR (KBr, cm^1) 2236 (C≡N), 1724 (C=O). HRMS-ESI *(m/z)*: $[M+H]^+$ Calcd for $C_{15}H_{19}N_2O_3$ 275.1396; Found 275.1406.

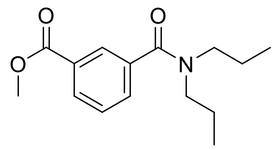

Methyl 3-(dipropylcarbamoyl)benzoate (16d).

The title compound was obtained as a colorless oil (426 mg, 97% yield) from 3-methoxycarbonylbenzoic acid (300 mg, 1.66 mmol, 1.0 eq) (**13d**), dipropylamine (0.27 mL, 2.00 mmol, 1.2 eq), HBTU (632 mg, 1.66 mmol, 1.0 eq) and TEA (0.46 mL, 3.33 mmol, 2.0 eq) by following general procedure B. Pure material was obtained by column chromatography using gradient elution from 10% EtOAc in petroleum ether to 25% EtOAc in petroleum ether.

^{1}H-NMR (400 MHz, $CDCl_3$) δ: 8.08-8.01 (m, 2H, Ar-H), 7.56 (ddd, *J* = 7.6, 1.8, 1.3 Hz, 1H, Ar-H), 7.50-7.45 (m, 1H, Ar-H), 3.93 (s, 3H, OCH_3), 3.50-3.40 (m, 2H, NCH_2), 3.21-3.08 (m, 2H, NCH_2), 1.76-1.64 (m, 2H, CH_2), 1.58-1.46 (m, 2H, CH_2), 1.04-0.93 (m, 3H, CH_3), 0.82-0.67 (m, 3H, CH_3) ppm. ^{13}C-NMR (101 MHz, $CDCl_3$) δ: 170.8, 166.6, 137.8, 131.2, 130.4, 130.3, 128.8, 127.8, 52.4, 50.9, 46.6, 22.1, 20.9, 11.6, 11.2 ppm. HRMS-ESI *(m/z)*: $[M+H]^+$ Calcd for $C_{15}H_{22}NO_3$ 264.1600; Found 264.1598.

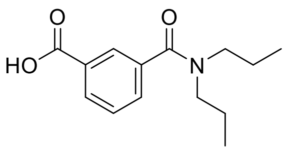

3-(Dipropylcarbamoyl)benzoic acid (17d).

The title compound was obtained as a white solid material (382 mg, 97% yield) from benzoate **16d** (416 mg, 1.58 mmol, 1.0 eq) and aqueous 1 M

NaOH solution (2.37 mL, 2.37 mmol, 1.5 eq) by following general procedure C. The crude product **17d** was used further without purification. ^{1}H-NMR spectra was identical to that from the literature.[8]
^{1}H-NMR (300 MHz, $CDCl_3$) δ: 10.29 (br s, 1H, COOH), 8.23-8.01 (m, 2H, Ar-H), 7.66-7.58 (m, 1H, Ar-H), 7.55-7.46 (m, 1H, Ar-H), 3.59-3.37 (m, 2H, NCH_2), 3.24-3.06 (m, 2H, NCH_2), 1.82-1.63 (m, 2H, CH_2), 1.61-1.46 (m, 2H, CH_2), 0.99 (t, J = 7.4 Hz, 3H, CH_3), 0.76 (t, J = 7.4 Hz, 3H, CH_3) ppm.

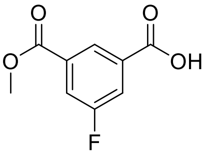

3-Fluoro-5-(methoxycarbonyl)benzoic acid (13e).
The title compound was obtained as a white solid material (213 mg, 91% yield) from dimethyl 5-fluoroisophthalate (250 mg, 1.18 mmol, 1.0 eq) and aqueous 1 M NaOH solution (1.18 mL, 1.18 mmol, 1.0 eq) by following general procedure A. The crude product **13e** was used in subsequent step without purification.
^{1}H-NMR (400 MHz, CD_3OD) δ: 8.44 (dd, J = 1.5, 1.5 Hz, 1H, Ar-H), 7.95-7.89 (m, 2H, Ar-H), 3.95 (s, 3H, OCH_3) ppm. ^{13}C-NMR (101 MHz, CD_3OD) δ: 167.3 (d, J = 3.0 Hz), 166.4 (d, J = 3.0 Hz), 165.0, 162.6, 134.1 (d, J = 7.5 Hz), 127.4 (d, J = 3.0 Hz), 121.6 (d, J = 23.4 Hz), 121.2 (d, J = 23.7 Hz), 53.2 ppm. HRMS-ESI *(m/z)*: $[M-H]^-$ Calcd for $C_9H_6O_4F$ 197.0250; Found 197.0247.

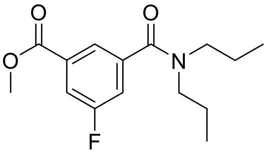

Methyl 3-(dipropylcarbamoyl)-5-fluorobenzoate (16e).
The title compound was obtained as a colorless oil (163 mg, 58% yield) from monoester **13e** (199 mg, 1.00 mmol, 1.0 eq), dipropylamine (0.16 mL, 1.20 mmol, 1.2 eq), HBTU (381 mg, 1.00 mmol, 1.0 eq) and TEA (0.28 mL, 2.01 mmol, 2.0 eq) by following general procedure B. Pure material was obtained by column chromatography using gradient elution from 25% EtOAc in petroleum ether to 50% EtOAc in petroleum ether.
^{1}H-NMR (400 MHz, $CDCl_3$) δ: 7.81 (dd, J = 1.4, 1.4 Hz, 1H, Ar-H), 7.74 (ddd, J = 8.9, 2.6, 1.4 Hz, 1H, Ar-H), 7.29-7.26 (m, 1H, Ar-H), 3.94 (s, 3H, OCH_3), 3.45 (t, J = 7.7 Hz, 2H, NCH_2), 3.14 (t, J = 7.7 Hz, 2H, NCH_2), 1.74-1.63 (m, 2H, CH_2), 1.60-1.47 (m, 2H, CH_2), 0.99 (t, J = 7.3 Hz, 3H, CH_3), 0.76 (t, J = 7.3 Hz, 3H, CH_3) ppm. ^{13}C-NMR (101 MHz, $CDCl_3$) δ: 169.3 (d, J = 2.3 Hz), 165.4 (d, J = 3.0 Hz), 163.7, 161.2, 139.8 (d, J = 6.8 Hz), 123.5 (d, J = 3.3 Hz), 118.5 (d, J = 22.9 Hz), 117.3 (d, J = 23.1 Hz), 52.7, 50.9, 46.7, 22.1, 20.8, 11.6, 11.2 ppm. HRMS-ESI *(m/z)*: $[M+H]^+$ Calcd for $C_{15}H_{21}NO_3F$ 282.1505; Found 282.1518.

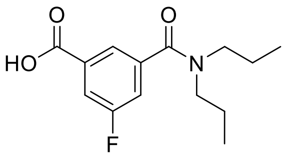

3-(Dipropylcarbamoyl)-5-fluorobenzoic acid (17e).
The title compound was obtained as a colorless sticky oil (146 mg, 100% yield) from benzoate **16e** (154 mg, 0.55 mmol, 1.0 eq) and aqueous 1 M

[8] Bjoerklund, C.; Oscarson, S.; Benkestock, K.; Borkakoti, N.; Jansson, K.; Lindberg, J.; Vrang, L.; Hallberg, A.; Rosenquist, A.; Samuelsson, B.. *J. Med. Chem.* **2010**, *53*, 1458–1464.

NaOH solution (0.82 mL, 0.82 mmol, 1.5 eq) by following general procedure C. The crude product **17e** was used further without purification.

^{1}H-NMR (400 MHz, $CDCl_3$) δ: 10.46 (br s, 1H, COOH), 7.89-7.85 (m, 1H, Ar-H), 7.78 (ddd, *J* = 8.7, 2.7, 1.3 Hz, 1H, Ar-H), 7.32 (ddd, *J* = 8.1, 2.7, 1.3 Hz, 1H, Ar-H), 3.47 (t, *J* = 7.8 Hz, 2H, NCH_2), 3.15 (t, *J* = 7.8 Hz, 2H, NCH_2), 1.75-1.64 (m, 2H, CH_2), 1.61-1.49 (m, 2H, CH_2), 0.98 (t, *J* = 7.3 Hz, 3H, CH_3), 0.77 (t, *J* = 7.3 Hz, 3H, CH_3) ppm. ^{13}C-NMR (101 MHz, $CDCl_3$) δ: 169.5 (d, *J* = 2.2 Hz), 169.2 (d, *J* = 2.3 Hz), 163.7, 161.2, 139.5 (d, *J* = 6.7 Hz), 124.0 (d, *J* = 3.1 Hz), 119.2 (d, *J* = 22.9 Hz), 117.9 (d, *J* = 23.0 Hz), 51.0, 46.9, 22.1, 20.8, 11.6, 11.2 ppm. HRMS-ESI *(m/z)*: $[M+H]^+$ Calcd for $C_{14}H_{19}NO_3F$ 268.1349; Found 268.1356.

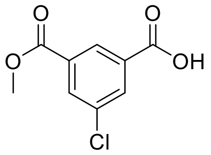

3-Chloro-5-(methoxycarbonyl)benzoic acid (13f).

The title compound was obtained as a white solid material (282 mg, 100% yield) from dimethyl 5-chloroisophthalate (300 mg, 1.31 mmol, 1.0 eq) and aqueous 1 M NaOH solution (1.31 mL, 1.31 mmol, 1.0 eq) by following general procedure A. The crude product **13f** was used in subsequent step without purification.

^{1}H-NMR (400 MHz, $CDCl_3$) δ: 8.64 (dd, *J* = 1.5, 1.5 Hz, 1H, Ar-H), 8.26 (dd, *J* = 1.5, 1.5 Hz, 2H, Ar-H), 3.98 (s, 3H, OCH_3) ppm.

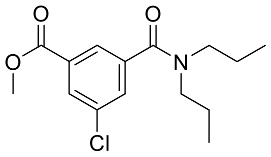

Methyl 3-chloro-5-(dipropylcarbamoyl)benzoate (16f).

The title compound was obtained as a colorless oil (300 mg, 77% yield) from monoester **13f** (282 mg, 1.31 mmol, 1.0 eq), dipropylamine (0.22 mL, 1.58 mmol, 1.2 eq), HBTU (498 mg, 1.31 mmol, 1.0 eq) and TEA (0.37 mL, 2.63 mmol, 2.0 eq) by following general procedure B. Pure material was obtained by column chromatography using 15% EtOAc in petroleum ether.

^{1}H-NMR (400 MHz, $CDCl_3$) δ: 8.06-8.00 (m, 1H, Ar-H), 7.93-7.87 (m, 1H, Ar-H), 7.56-7.50 (m, 1H, Ar-H), 3.94 (s, 3H, OCH_3), 3.49-3.41 (m, 2H, NCH_2), 3.18-3.09 (m, 2H, NCH_2), 1.75-1.64 (m, 2H, CH_2), 1.60-1.49 (m, 2H, CH_2), 0.98 (t, *J* = 7.4 Hz, 3H, CH_3), 0.77 (t, *J* = 7.4 Hz, 3H, CH_3) ppm. ^{13}C-NMR (101 MHz, $CDCl_3$) δ: 169.2, 165.4, 139.3, 135.0, 132.1, 131.2, 130.3, 125.9, 52.8, 50.9, 46.7, 22.1, 20.8, 11.6, 11.2 ppm. HRMS-ESI *(m/z)*: $[M+H]^+$ Calcd for $C_{15}H_{21}NO_3Cl$ 298.1210; Found 298.1223.

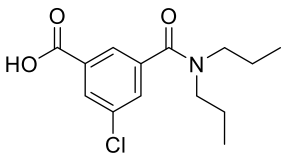

3-Chloro-5-(dipropylcarbamoyl)benzoic acid (17f).

The title compound was obtained as a white solid material (275 mg, 98% yield) from benzoate **16f** (293 mg, 0.98 mmol, 1.0 eq) and aqueous 1 M

NaOH solution (1.48 mL, 1.48 mmol, 1.5 eq) by following general procedure C. The crude product **17f** was used further without purification.

^{1}H-NMR (400 MHz, $CDCl_3$) δ: 8.09 (dd, *J* = 1.8, 1.8 Hz, 1H, Ar-H), 7.96 (dd, *J* = 1.8, 1.8 Hz, 1H, Ar-H), 7.59 (dd, *J* = 1.8, 1.8 Hz, 1H, Ar-H), 3.51-3.43 (m, 2H, NCH_2), 3.16 (t, *J* = 7.4 Hz, 2H, NCH_2), 1.77-1.65 (m, 2H, CH_2), 1.62-1.50 (m, 2H, CH_2), 0.99 (t, *J* = 7.4 Hz, 3H, CH_3), 0.78 (t, *J* = 7.4 Hz, 3H, CH_3) ppm. ^{13}C-NMR (101 MHz, $CDCl_3$) δ: 169.4, 168.9, 139.2, 135.2, 131.9, 131.5, 130.9, 126.4, 51.0, 46.8, 22.1, 20.8, 11.6, 11.2 ppm. HRMS-ESI *(m/z)*: $[M+H]^+$ Calcd for $C_{14}H_{19}NO_3Cl$ 284.1053; Found 284.1064.

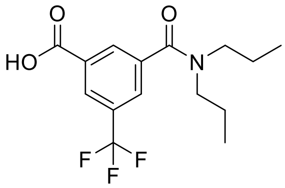

3-(Dipropylcarbamoyl)-5-(trifluoromethyl)benzoic acid (17h).

The title compound was obtained as a brownish oil (90 mg, 33% yield) from 5-(trifluoromethyl)isophthalic acid (200 mg, 0.85 mmol, 1.0 eq), dipropylamine (0.12 mL, 0.85 mmol, 1.0 eq), HBTU (324 mg, 0.85 mmol, 1.0 eq) and TEA (0.24 mL, 1.71 mmol, 2.0 eq) by following general procedure B. Pure material was obtained by column chromatography using 25% EtOAc in petroleum ether.

^{1}H-NMR (400 MHz, $CDCl_3$) δ: 8.38-8.35 (m, 1H, Ar-H), 8.27-8.24 (m, 1H, Ar-H), 7.87-7.84 (m, 1H, Ar-H), 3.55-3.43 (m, 2H, NCH_2), 3.20-3.09 (m, 2H, NCH_2), 1.80-1.66 (m, 2H, CH_2), 1.63-1.51 (m, 2H, CH_2), 1.01 (t, *J* = 7.6 Hz, 3H, CH_3), 0.78 (t, *J* = 7.6 Hz, 3H, CH_3) ppm. ^{13}C-NMR (101 MHz, $CDCl_3$) δ: 169.5, 168.6, 138.5, 132.0, 131.4, 128.4, 127.8, 124.7, 122.0, 51.1, 47.0, 22.1, 20.8, 11.6, 11.1 ppm. HRMS-ESI *(m/z)*: $[M+H]^+$ Calcd for $C_{15}H_{19}NO_3F_3$ 318.1317; Found 318.1321.

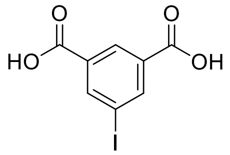

5-Iodoisophthalic acid (18).

To a solution of dimethyl 5-iodoisophthalate (500 mg, 1.56 mmol, 1.0 eq) in MeOH (20 mL) was added an aqueous 1 M NaOH solution (4.69 mL, 4.69 mmol, 3.0 eq). After stirring at 40 ^{o}C temperature for 18 h, the brown suspension was diluted with water (10 mL) and acidified to pH 3 with aqueous 1M HCl solution. The aqueous layer was extracted with EtOAc (3 x 20 mL). Combined organic extracts were washed with brine (20 mL), dried over anhydrous Na_2SO_4 and evaporated under reduced pressure to yield product **18** as a yellowish solid (455 mg, 100% yield), which was used in subsequent step without purification. ^{1}H-NMR spectrum was identical to that from the literature.[9]

^{1}H-NMR (400 MHz, CD_3OD) δ: 8.59 (dd, *J* = 1.5, 1.5 Hz, 1H, Ar-H), 8.52 (d, *J* = 1.5 Hz, 2H, Ar-H) ppm. HRMS-ESI *(m/z)*: $[M-H]^-$ Calcd for $C_8H_4O_4I$ 290.9154; Found 290.9165.

[9] Zhang, S.; Liu, Q.; Shen, M.; Hu, B.; Chen, Q.; Li, H.; Ammoureux, J. P. *Dalton Trans.* **2012**, *41*, 4692-4698.

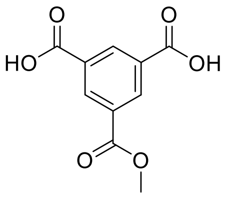

5-(Methoxycarbonyl)isophthalic acid (13g).

Isophthalic acid **18** (451 mg, 1.54 mmol, 1.0 eq), Pd(dppf)Cl_2·CH_2Cl_2 (126 mg, 0.15 mmol, 0.10 eq), TEA (0.47 mL, 3.40 mmol, 2.2 eq) and dry MeOH (10 mL) were combined in a 100 mL glass liner, which was then placed in stainless steel autoclave with a pressure gauge. The autoclave was sealed and purged three times with carbon monoxide and then pressurized to 70 psi with carbon monoxide. The mixture was heated at 100 ^oC. After 18 h the autoclave was cooled down to room temperature and the excess of carbon monoxide was carefully released under fume hood. The dark brown suspension was filtered through Celite. The filter plug was washed with EtOAc and filtrate was dried over anhydrous Na_2SO_4. Volatiles were evaporated under reduced pressure. An aqueous 1M HCl solution was added to pH 1 and then suspension was extracted with EtOAc (3 x 20 mL). Combined organic extracts were washed with brine (15 mL), dried over anhydrous Na_2SO_4 and evaporated under reduced pressure, to give 346 mg (100% yield) of **13g** as a dark brown solid, which was used in the subsequent step without purification.

^{1}H-NMR (400 MHz, DMSO-d_6) δ: 8.68-8.65 (m, 1H, Ar-H), 8.63 (d, *J* = 1.6 Hz, 2H, Ar-H), 3.92 (s, 3H, OCH_3) ppm. ^{13}C-NMR (101 MHz, DMSO-d_6) δ: 165.8, 164.9, 133.9, 133.2, 132.5, 130.6, 52.7 ppm. HRMS-ESI *(m/z)*: $[M-H]^-$ Calcd for $C_{10}H_7O_6$ 223.0243; Found 223.0250.

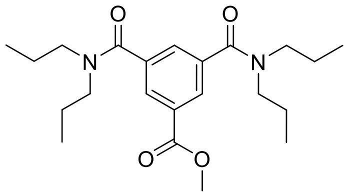

Methyl 3,5-bis(dipropylcarbamoyl)benzoate (16g).

The title compound was obtained as a colorless oil (337 mg, 57% yield) from monoester **13g** (340 mg, 1.52 mmol, 1.0 eq), dipropylamine (0.46 mL, 3.34 mmol, 2.2 eq), HBTU (1.15 g, 3.03 mmol, 2.0 eq) and TEA (0.85 mL, 6.07 mmol, 4.0 eq) by following general procedure B. Pure material was obtained by column chromatography using gradient elution from 25% EtOAc in petroleum ether to 100% EtOAc.

^{1}H-NMR (400 MHz, $CDCl_3$) δ: 8.05 (d, *J* = 1.6 Hz, 2H, Ar-H), 7.55 (dd, *J* = 1.6, 1.6 Hz, 1H, Ar-H), 3.93 (s, 3H, OCH_3), 3.45 (t, *J* = 7.5 Hz, 4H, NCH_2×2), 3.14 (t, *J* = 7.5 Hz, 4H, NCH_2×2), 1.74-1.63 (m, 4H, CH_2×2), 1.58-1.48 (m, 4H, CH_2×2), 0.98 (t, *J* = 7.3 Hz, 6H, CH_3×2), 0.75 (t, *J* = 7.3 Hz, 6H, CH_3×2) ppm. ^{13}C-NMR (101 MHz, $CDCl_3$) δ: 170.0, 165.9, 138.2, 130.7, 129.4, 128.3, 52.6, 50.9, 46.6, 22.1, 20.8, 11.6, 11.2 ppm. HRMS-ESI *(m/z)*: $[M+H]^+$ Calcd for $C_{22}H_{35}N_2O_4$ 391.2597; Found 391.2600.

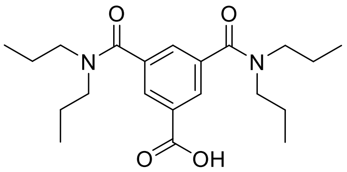

3,5-Bis(dipropylcarbamoyl)benzoic acid (17g).

The title compound was obtained as a colorless sticky oil (301 mg, 96% yield) from benzoate **13g** (324 mg, 0.83 mmol, 1.0 eq) and aqueous 1

M NaOH solution (1.24 mL, 1.24 mmol, 1.5 eq) by following general procedure C. The crude product **17g** was used further without purification.

^{1}H-NMR (400 MHz, $CDCl_3$) δ: 10.08 (br s, 1H, COOH), 8.08 (d, *J* = 1.6 Hz, 2H, Ar-H), 7.59 (dd, *J* = 1.6, 1.6 Hz, 1H, Ar-H), 3.46 (t, *J* = 7.7 Hz, 4H, NCH_2×2), 3.15 (t, *J* = 7.7 Hz, 4H, NCH_2×2), 1.75-1.64 (m, 4H, CH_2×2), 1.58-1.48 (m, 4H, CH_2×2), 0.98 (t, *J* = 7.3 Hz, 6H, CH_3×2), 0.74 (t, *J* = 7.3 Hz, 6H, CH_3×2) ppm. ^{13}C-NMR (101 MHz, $CDCl_3$) δ: 170.2, 168.9, 138.0, 130.5, 129.8, 128.8, 51.0, 46.7, 22.0, 20.8, 11.6, 11.1 ppm. HRMS-ESI *(m/z)*: $[M+H]^+$ Calcd for $C_{21}H_{33}N_2O_4$ 377.2440; Found 377.2450.

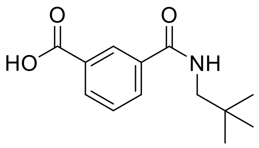

3-(Neopentylcarbamoyl)benzoic acid (19a).

The benzoate intermediate was obtained as a yellow oil (164 mg, 79% yield) from 3-methoxycarbonylbenzoic acid (150 mg, 0.83 mmol, 1.0 eq) (**13d**), 2,2-dimethylpropan-1-amine (87 mg, 1.00 mmol, 1.2 eq), HBTU (316 mg, 0.83 mmol, 1.0 eq) and TEA (0.23 mL, 1.66 mmol, 2.0 eq) by following general procedure B. Pure material was obtained by column chromatography using gradient elution from 25% EtOAc in petroleum ether to 100% EtOAc.

^{1}H-NMR (400 MHz, $CDCl_3$) δ: 8.38-8.35 (m, 1H, Ar-H), 8.18-8.13 (m, 1H, Ar-H), 8.05-8.00 (m, 1H, Ar-H), 7.56-7.49 (m, 1H, Ar-H), 6.31-6.18 (m, 1H, NH), 3.94 (s, 3H, OCH_3), 3.30 (d, *J* = 6.4 Hz, 2H, NCH_2), 0.99 (s, 9H, CH_3×3) ppm. ^{13}C-NMR (101 MHz, $CDCl_3$) δ: 166.8, 166.5, 135.5, 132.4, 131.9, 130.6, 129.0, 127.5, 52.5, 51.3, 32.4, 27.5 ppm. HRMS-ESI *(m/z)*: $[M+H]^+$ Calcd for $C_{14}H_{20}NO_3$ 250.1443; Found 250.1449.

The title compound was obtained as a white solid material (144 mg, 99% yield) from benzoate intermediate (154 mg, 0.62 mmol, 1.0 eq) and aqueous 1 M NaOH solution (0.93 mL, 0.93 mmol, 1.5 eq) by following general procedure C. The crude product **19a** was used further without purification.

^{1}H-NMR (400 MHz, CD_3OD) δ: 8.59-8.50 (m, 1H, NH), 8.48-8.45 (m, 1H, Ar-H), 8.20-8.14 (m, 1H, Ar-H), 8.03 (ddd, *J* = 7.8, 1.8, 1.2 Hz, 1H, Ar-H), 7.61-7.54 (m, 1H, Ar-H), 3.25-3.21 (m, 2H, NCH_2), 0.98 (s, 9H, CH_3×3) ppm. ^{13}C-NMR (101 MHz, CD_3OD) δ: 169.8, 169.0, 136.7, 133.4, 132.6, 132.4, 129.8, 129.6, 51.9, 33.8, 27.9 ppm. HRMS-ESI *(m/z)*: $[M+H]^+$ Calcd for $C_{13}H_{18}NO_3$ 236.1287; Found 236.1287.

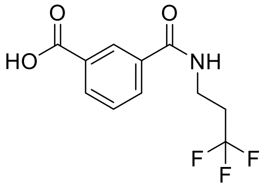

3-[(3,3,3-Trifluoropropyl)carbamoyl]benzoic acid (19b).

The benzoate intermediate was obtained as a yellow oil (127 mg, 55% yield) from 3-methoxycarbonylbenzoic acid (150 mg, 0.83 mmol, 1.0 eq) (**13d**), 3,3,3-trifluoropropylamine (94 mg, 0.83 mmol, 1.0 eq), HBTU (316 mg, 0.83 mmol, 1.0 eq) and TEA (0.23 mL, 1.66 mmol, 2.0 eq) by following general procedure B. Pure material

was obtained by column chromatography using gradient elution from 25% EtOAc in petroleum ether to 100% EtOAc.

^{1}H-NMR (400 MHz, $CDCl_3$) δ: 8.37-8.34 (m, 1H, Ar-H), 8.20-8.15 (m, 1H, Ar-H), 8.03-7.98 (m, 1H, Ar-H), 7.54 (td, *J* = 7.8, 0.7 Hz, 1H, Ar-H), 6.58-6.49 (m, 1H, NH), 3.94 (s, 3H, OCH_3), 3.77-3.70 (m, 2H, NCH_2), 2.57-2.42 (m, 2H, CH_2) ppm. ^{13}C-NMR (101 MHz, $CDCl_3$) δ: 166.8, 166.4, 134.4, 132.8, 131.8, 130.8, 129.2, 127.7, 52.6, 33.9, 33.8, 33.7 ppm. ^{19}F-NMR (376 MHz, $CDCl_3$) δ: -65.0 (t, *J* = 10.6 Hz) ppm. HRMS-ESI *(m/z)*: $[M+H]^+$ Calcd for $C_{12}H_{13}NO_3F_3$ 276.0848; Found 276.0848.

The title compound was obtained as a white solid material (114 mg, 99% yield) from benzoate intermediate (121 mg, 0.44 mmol, 1.0 eq) and aqueous 1 M NaOH solution (0.66 mL, 0.66 mmol, 1.5 eq) by following general procedure C. The crude product **19b** was used further without purification.

^{1}H-NMR (400 MHz, CD_3OD) δ: 8.48 (td, *J* = 1.8, 0.5 Hz, 1H, Ar-H), 8.21-8.16 (m, 1H, Ar-H), 8.03 (ddd, *J* = 7.8, 1.8, 1.2 Hz, 1H, Ar-H), 7.58 (td, *J* = 7.8, 0.5 Hz, 1H, Ar-H), 3.64 (t, *J* = 7.0 Hz, 2H, NCH_2), 2.61-2.46 (m, 2H, CH_2) ppm. ^{13}C-NMR (101 MHz, CD_3OD) δ: 169.3, 168.8, 135.8, 133.7, 132.6, 129.9, 129.5, 126.6, 34.5, 34.2, 33.9 ppm. HRMS-ESI *(m/z)*: $[M+H]^+$ Calcd for $C_{11}H_{11}NO_3F_3$ 262.0691; Found 262.0692.

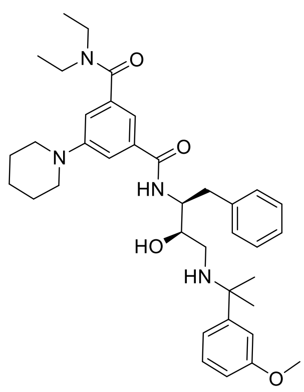

***N*^{1},*N*^{1}-Diethyl-*N*^{3}-[(2*S*,3*R*)-3-hydroxy-4-{[2-(3-methoxyphenyl)propan-2-yl]amino}-1-phenylbutan-2-yl]-5-(piperidin-1-yl)isophthalamide ((*S*,*R*)-2a)**.

The title compound was obtained from *rac*-**11** (50 mg, 0.12 mmol, 1.0 eq), benzoic acid **15a** (38 mg, 0.12 mmol, 1.0 eq), HBTU (47 mg, 0.12 mmol, 1.0 eq) and TEA (70 µL, 0.50 mmol, 4.0 eq) by following general procedure D. Purification by column chromatography on silica gel using gradient elution from 50% EtOAc in petroleum ether to 100% EtOAc, followed by 2% MeOH in EtOAc afforded *rac*-**2a** as a white solid (41 mg, 54% yield). Enantiomerically pure material (*S*,*R*)-**2a** (14 mg, 18% yield) was obtained by preparative HPLC on chiral stationary phase (*Chiralpak-IC*), using 50% EtOAc/50% $CHCl_3$/0.1% DEA as a mobile phase (flow rate 15 mL/min, detector UV 230 nm).

^{1}H-NMR (400 MHz, $CDCl_3$) δ: 7.26-7.15 (m, 7H, Ar-H, NH), 7.05-6.99 (m, 2H, Ar-H), 6.93 (dd, *J* = 2.5, 1.2 Hz, 1H, Ar-H), 6.89-6.85 (m, 1H, Ar-H), 6.78-6.73 (m, 1H, Ar-H), 6.58 (d, *J* = 8.8 Hz, 1H, Ar-H), 4.42-4.33 (m, 1H, CH), 3.78 (s, 3H, OCH_3), 3.56-3.48 (m, 2H, CH_2), 3.27-3.12 (m, 5H, NCH_2×2, CH), 3.08-2.87 (m, 2H, CH_2), 2.54-2.44 (m, 2H, CH_2), 1.71-1.65 (m, 4H, CH_2×2), 1.63-1.58 (m, 2H, CH_2), 1.54 (s, 3H, CH_3), 1.52 (s, 3H, CH_3), 1.36-1.17 (m, 6H, CH_3, CH_2, OH), 1.14-1.00 (m, 3H, CH_3) ppm. ^{13}C-NMR (101 MHz, $CDCl_3$) δ: 171.0, 168.1, 159.9, 152.2, 138.5, 137.8,

129.6, 129.5, 128.7, 126.7, 118.4, 116.2, 115.3, 113.9, 112.4, 70.7, 55.4, 53.5, 50.0, 36.7, 25.7 ppm. HRMS-ESI *(m/z)*: $[M+H]^+$ Calcd for $C_{37}H_{51}N_4O_4$ 615.3910; Found 615.3904. Optical rotation $[\alpha]^{20}_D$ –35.7 (*c* 0.62, $CHCl_3$).

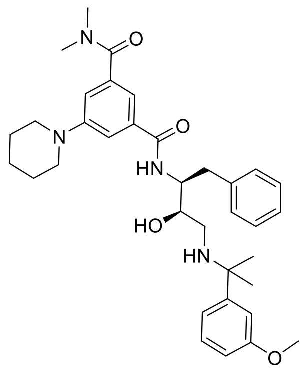

***N*^{1}-[(2*S*,3*R*)-3-Hydroxy-4-{[2-(3-methoxyphenyl)propan-2-yl]amino}-1-phenylbutan-2-yl]-*N*^{3},*N*^{3}-dimethyl-5-(piperidin-1-yl) isophthalamide ((*S*,*R*)-2b)**.

The title compound was obtained from (*R*,*S*)-**11**(25 mg, 0.062 mmol, 1.0 eq), benzoic acid **15b** (17 mg, 0.062 mmol, 1.0 eq), HBTU (24 mg, 0.062 mmol, 1.0 eq) and TEA (35 µL, 0.25 mmol, 4.0 eq) by following general procedure D. Purification by column chromatography on silica gel using gradient elution from 25% EtOAc in petroleum ether to 100% EtOAc afforded (*S*,*R*)-**2b** as a colorless sticky oil (17 mg, 47% yield).

^{1}H-NMR (400 MHz, $CDCl_3$) δ: 7.25-7.14 (m, 7H, Ar-H, NH), 7.02-6.97 (m, 3H, Ar-H), 6.94-6.89 (m, 1H, Ar-H), 6.76-6.64 (m, 2H, Ar-H), 4.39-4.29 (m, 1H, CH), 3.77 (s, 3H, OCH_3), 3.53-3.46 (m, 1H, CH), 3.23-3.15 (m, 4H, NCH_2×2), 3.08 (s, 3H, NCH_3), 3.01-2.75 (m, 5H, NCH_3, CH_2), 2.46 (d, *J* = 4.3 Hz, 2H, CH_2), 1.71-1.63 (m, 4H, CH_2×2), 1.62-1.56 (m, 2H, CH_2), 1.48 (s, 3H, CH_3), 1.47 (s, 3H, CH_3) ppm. ^{13}C-NMR (101 MHz, $CDCl_3$) δ: 171.4, 167.9, 159.8, 152.2, 148.6, 137.9, 137.6, 135.7, 129.5, 129.4, 128.6, 126.6, 118.5, 116.9, 115.6, 114.6, 112.4, 111.6, 70.8, 56.1, 55.3, 53.8, 50.0, 44.6, 36.7, 29.9, 29.0, 25.7, 24.3 ppm. HRMS-ESI *(m/z)*: $[M+H]^+$ Calcd for $C_{35}H_{47}N_4O_4$ 587.3597; Found 587.3580. Optical rotation $[\alpha]^{20}_D$ –26.7 (*c* 1.24, $CHCl_3$).

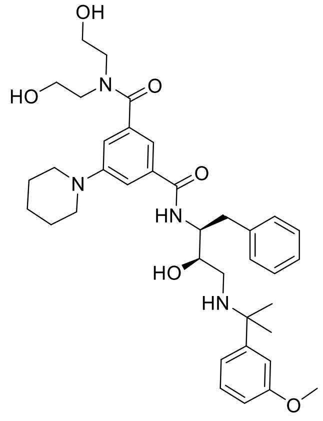

***N*^{1}-[(2*S*,3*R*)-3-Hydroxy-4-{[2-(3-methoxyphenyl)propan-2-yl]amino}-1-phenylbutan-2-yl]-*N*^{3},*N*^{3}-bis(2-hydroxyethyl)-5-(piperidin-1-yl)isophthalamide ((*S*,*R*)-2c)**.

The protected intermediate was obtained from (*R,S*)-**11** (35 mg, 0.087 mmol, 1.0 eq), benzoic acid **15c** (49 mg, 0.087 mmol, 1.0 eq), HBTU (33 mg, 0.087 mmol, 1.0 eq) and TEA (49 µL, 0.35 mmol, 4.0 eq) by following general procedure D. Purification by column chromatography on amino-functionalized silica gel using gradient elution from 25% EtOAc in petroleum ether to 50% EtOAc in petroleum ether afforded protected intermediate as a colorless oil (60 mg, 79% yield), which was used in subsequent step without purification. HRMS-ESI *(m/z)*: $[M+H]^+$ Calcd for $C_{49}H_{79}N_4O_6Si_2$ 875.5538; Found 875.5530.

To a solution of protected intermediate from above (53 mg, 0.060 mmol, 1.0 eq) in anhydrous THF (4 mL) was dropwise added TBAF (1M solution in THF, 0.24 mL, 0.24 mmol, 4.0 eq). After stirring

for 1 h at room temperature all the volatiles were evaporated under reduced pressure. The residue was diluted with water (10 mL) and extracted with EtOAc (3 x 10 mL). Combined organic layers were washed with brine (15 mL), dried over Na_2SO_4 and evaporated under reduced pressure. Purification by column chromatography on amino-functionalized silica gel using gradient elution from 100% EtOAc to 5% MeOH in EtOAc afforded (*S*,*R*)-**2c** as a white solid (16 mg, 41%).

^{1}H-NMR (400 MHz, $CDCl_3$) δ: 7.39 (d, *J* = 8.7 Hz, 1H, NH), 7.24-7.11 (m, 8H, Ar-H), 7.05 (dd, *J* = 2.4, 1.2 Hz, 1H, Ar-H), 7.01-6.94 (m, 2H, Ar-H), 6.76-6.71 (m, 1H, Ar-H), 4.36-4.27 (m, 1H, CH), 3.93-3.83 (m, 2H, OCH_2), 3.76 (s, 3H, OCH_3), 3.69-3.53 (m, 5H, OCH_2, CH_2, CH), 3.43-3.29 (m, 2H, CH_2), 3.22-3.11 (m, 4H, NCH_2×2), 2.94-2.79 (m, 2H, NCH_2), 2.50-2.39 (m, 2H, NCH_2), 1.67-1.60 (m, 4H, CH_2×2), 1.60-1.53 (m, 2H, CH_2), 1.44 (s, 3H, CH_3), 1.43 (s, 3H, CH_3) ppm. ^{13}C-NMR (101 MHz, $CDCl_3$) δ: 173.6, 168.0, 159.7, 152.2, 148.7, 138.3, 137.1, 135.6, 129.4, 128.5, 126.4, 118.5, 117.0, 115.9, 115.2, 112.5, 111.4, 71.2, 60.8, 55.9, 55.3, 54.4, 53.5, 49.9, 44.8, 36.5, 30.1, 28.8, 25.6, 24.3 ppm. HRMS-ESI *(m/z)*: $[M+H]^+$ Calcd for $C_{37}H_{51}N_4O_6$ 647.3809; Found 647.3796. Optical rotation $[\alpha]^{20}_D$ –38.7 (*c* 1.16, $CHCl_3$).

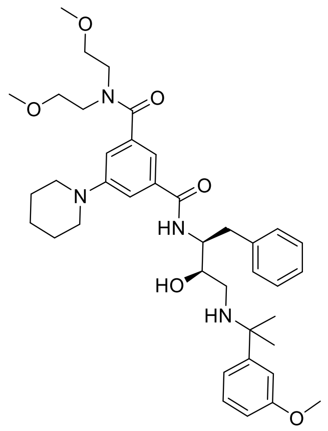

***N*^{1}-[(2*S*,3*R*)-3-Hydroxy-4-{[2-(3-methoxyphenyl)propan-2-yl]amino}-1-phenylbutan-2-yl]-*N*^{3},*N*^{3}-bis(2-methoxyethyl)-5-(piperidin-1-yl)isophthalamide ((*S*,*R*)-2d)**.

The title compound was obtained from (*R*,*S*)-**11** (35 mg, 0.087 mmol, 1.0 eq), benzoic acid **15d** (32 mg, 0.087 mmol, 1.0 eq), HBTU (33 mg, 0.087 mmol, 1.0 eq) and TEA (49 µL, 0.35 mmol, 4.0 eq) by following general procedure D. Purification by column chromatography on silica gel using gradient elution from 100% EtOAc to 10% MeOH in EtOAc afforded (*S*,*R*)-**2d** as a colorless sticky oil (18 mg, 31% yield).

^{1}H-NMR (400 MHz, $CDCl_3$) δ: 7.25-7.15 (m, 7H, Ar-H, NH), 7.05 (dd, *J* = 2.5, 1.2 Hz, 1H, Ar-H), 7.02-6.96 (m, 3H, Ar-H), 6.73 (ddd, *J* = 8.2, 2.5, 0.9 Hz, 1H, Ar-H), 6.54 (d, *J* = 8.8 Hz, 1H, Ar-H), 4.39-4.29 (m, 1H, CH), 3.77 (s, 3H, OCH_3), 3.75-3.62 (m, 4H, OCH_2×2), 3.53-3.32 (m, 8H, OCH_3, NCH_2×2, CH), 3.27-3.17 (m, 7H, OCH_3, NCH_2×2), 3.05-2.91 (m, 2H, CH_2), 2.50-2.40 (m, 2H, CH_2), 1.72-1.64 (m, 4H, CH_2×2), 1.63-1.56 (m, 2H, CH_2), 1.47 (s, 3H, CH_3), 1.46 (s, 3H, CH_3) ppm. ^{13}C-NMR (101 MHz, $CDCl_3$) δ: 172.0, 167.8, 159.7, 152.1, 148.9, 137.9, 137.7, 135.5, 129.5, 129.3, 128.7, 126.6, 118.5, 117.2, 115.4, 114.6, 112.4, 111.5, 71.0, 59.0, 55.9, 55.3, 53.6, 49.9, 44.5, 36.7, 30.0, 29.2, 25.7, 24.3 ppm. HRMS-ESI *(m/z)*: $[M+H]^+$ Calcd for $C_{39}H_{55}N_4O_6$ 675.4122; Found 675.4120. Optical rotation $[\alpha]^{20}_D$ –35.1 (*c* 1.28, $CHCl_3$).

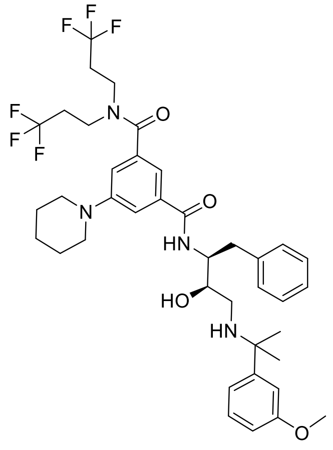

***N*[1]-[(2*S*,3*R*)-3-Hydroxy-4-{[2-(3-methoxyphenyl)propan-2-yl]amino}-1-phenylbutan-2-yl]-5-(piperidin-1-yl)-*N*[3],*N*[3]-bis(3,3,3-trifluoropropyl)isophthalamide ((*S*,*R*)-2e)**.

The title compound was obtained from (*R,S*)-**11** (35 mg, 0.087 mmol, 1.0 eq), benzoic acid **15e** (38 mg, 0.087 mmol, 1.0 eq), HBTU (33 mg, 0.087 mmol, 1.0 eq) and TEA (49 µL, 0.35 mmol, 4.0 eq) by following general procedure D. Purification by column chromatography on silica gel using gradient elution from 50% EtOAc in petroleum ether to 100% EtOAc afforded (*S*,*R*)-**2e** as a white solid (39 mg, 60% yield).

^{1}H-NMR (400 MHz, $CDCl_3$) δ: 7.25-7.17 (m, 7H, Ar-H, NH), 7.04-6.97 (m, 2H, Ar-H), 6.89 (dd, *J* = 2.6, 1.3 Hz, 1H, Ar-H), 6.84-6.82 (m, 1H, Ar-H), 6.75 (dd, *J* = 8.1, 2.6 Hz, 1H, Ar-H), 6.48 (d, *J* = 8.7 Hz, 1H, Ar-H), 4.38-4.29 (m, 1H, CH), 3.78 (s, 3H, OCH_3), 3.69-3.45 (m, 5H, NCH_2×2, CH), 3.22-3.17 (m, 4H, NCH_2×2), 3.04-2.93 (m, 2H, CH_2), 2.62-2.49 (m, 2H, CH_2), 2.48 (d, *J* = 4.3 Hz, 2H, CH_2), 2.35-2.22 (m, 2H, CH_2), 1.73-1.65 (m, 4H, CH_2×2), 1.64-1.58 (m, 2H, CH_2), 1.49 (s, 3H, CH_3), 1.48 (s, 3H, CH_3) ppm. ^{13}C-NMR (101 MHz, $CDCl_3$) δ: 171.9, 167.7, 159.8, 152.3, 137.8, 136.6, 136.1, 129.5, 129.4, 128.7, 126.7, 118.4, 115.9, 115.8, 113.6, 112.4, 111.7, 70.7, 56.4, 55.3, 53.7, 49.8, 44.6, 38.8, 36.6, 29.8, 29.6, 29.0, 25.6, 24.2 ppm. HRMS-ESI *(m/z)*: $[M+H]^+$ Calcd for $C_{39}H_{49}N_4O_4F_6$ 751.3658; Found 751.3660. Optical rotation $[\alpha]^{20}_D$ –22.4 (*c* 3.12, $CHCl_3$).

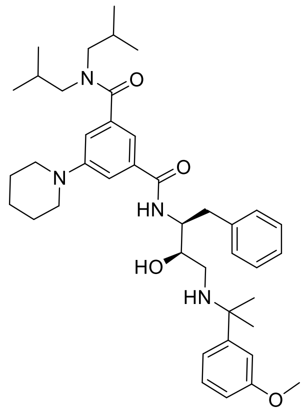

***N*[1]-[(2*S*,3*R*)-3-Hydroxy-4-{[2-(3-methoxyphenyl)propan-2-yl]amino}-1-phenylbutan-2-yl]-*N*[3],*N*[3]-diisobutyl-5-(piperidin-1-yl)isophthalamide ((*S*,*R*)-2f)**.

The title compound was obtained from *rac*-**11** (51 mg, 0.13 mmol, 1.0 eq), benzoic acid **15f** (46 mg, 0.13 mmol, 1.0 eq), HBTU (48 mg, 0.13 mmol, 1.0 eq) and TEA (71 µL, 0.51 mmol, 4.0 eq) by following general procedure D. Purification by column chromatography on silica gel using 1% MeOH in EtOAc afforded *rac*-**2f** as a white solid (58 mg, 68% yield). Enantiomerically pure material (*S*,*R*)-**2f** (28 mg, 33% yield) was obtained by preparative HPLC on chiral stationary phase (*Chiralpak-ID*), using 85% EtOAc/15% $CHCl_3$/0.1% DEA as a mobile phase (flow rate 30 mL/min, detector UV 254 nm).

^{1}H-NMR (400 MHz, $CDCl_3$) δ: 7.25-7.13 (m, 7H, Ar-H, NH), 7.03-6.99 (m, 2H, Ar-H), 6.93-6.88 (m, 1H, Ar-H), 6.85-6.80 (m, 1H, Ar-H), 6.75 (dd, *J* = 8.2, 2.4 Hz, 1H, Ar-H), 6.51 (d, *J* = 8.8 Hz, 1H, Ar-H), 4.90-4.74 (m, 2H, NH, OH), 4.41-4.29 (m, 1H, CH), 3.78 (s, 3H, OCH_3), 3.60-3.52 (m, 1H, CH), 3.41-3.28 (m, 2H, NCH_2), 3.22-3.13 (m, 4H, NCH_2×2), 3.07-2.92 (m, 4H, NCH_2, CH_2), 2.52-2.45 (m, 2H, CH_2), 2.15-2.04 (m, 1H, CH), 1.91-1.78 (m, 1H, CH), 1.72-1.65 (m, 4H, CH_2×2),

1.63-1.57 (m, 2H, CH_2), 1.47 (s, 3H, CH_3), 1.46 (s, 3H, CH_3), 0.98 (d, *J* = 6.6 Hz, 6H, CH_3×2), 0.73 (d, *J* = 6.6 Hz, 6H, CH_3×2) ppm. ^{13}C-NMR (101 MHz, $CDCl_3$) δ: 172.2, 168.0, 159.9, 152.2, 138.7, 137.7, 135.5, 129.6, 128.7, 126.7, 118.4, 117.2, 115.4, 114.6, 112.4, 112.2, 70.5, 57.2, 55.4, 53.3, 50.1, 45.2, 36.5, 28.9, 28.4, 25.7, 24.3, 20.4, 20.0 ppm. HRMS-ESI *(m/z)*: $[M+H]^+$ Calcd for $C_{41}H_{59}N_4O_4$ 671.4536; Found 671.4534. Optical rotation $[\alpha]^{20}_D$ –21.6 (*c* 2.11, $CHCl_3$).

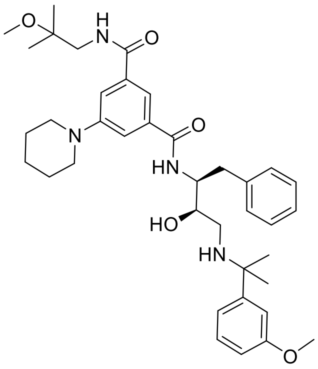

***N*¹-[(2*S*,3*R*)-3-Hydroxy-4-{[2-(3-methoxyphenyl)propan-2-yl]amino}-1-phenylbutan-2-yl]-*N*³-(2-methoxy-2-methylpropyl)-5-(piperidin-1-yl)isophthalamide ((*S*,*R*)-3a)**.

The title compound was obtained from (*R*,*S*)-**11** (30 mg, 0.075 mmol, 1.0 eq), benzoic acid **15g** (25 mg, 0.075 mmol, 1.0 eq), HBTU (28 mg, 0.075 mmol, 1.0 eq) and TEA (42 µL, 0.30 mmol, 4.0 eq) by following general procedure D. Purification by column chromatography on amino-functionalized silica gel using gradient elution from 50% EtOAc in petroleum ether to 100% EtOAc afforded (*S*,*R*)**-3a** as a white solid (30 mg, 62% yield).

^{1}H-NMR (400 MHz, $CDCl_3$) δ: 7.47 (dd, *J* = 2.6, 1.4 Hz, 1H, NH), 7.34-7.27 (m, 4H, Ar-H, NH), 7.25-7.19 (m, 4H, Ar-H), 7.06-6.99 (m, 2H, Ar-H), 6.76 (ddd, *J* = 8.2, 2.6, 0.9 Hz, 1H, Ar-H), 6.72-6.65 (m, 1H, Ar-H), 6.55-6.49 (m, 1H, Ar-H), 4.43-4.34 (m, 1H, CH), 3.80 (s, 3H, OCH_3), 3.57-3.47 (m, 3H, CH, NCH_2), 3.25 (s, 3H, OCH_3), 3.25-3.21 (m, 4H, NCH_2×2), 3.02 (d, *J* = 6.6 Hz, 2H, CH_2), 2.49 (d, *J* = 4.3 Hz, 2H, CH_2), 1.75-1.67 (m, 4H, CH_2×2), 1.66-1.60 (m, 2H, CH_2), 1.50 (s, 6H, CH_3×2), 1.24 (s, 6H, CH_3×2) ppm. ^{13}C-NMR (101 MHz, $CDCl_3$) δ: 167.8, 167.6, 159.7, 152.4, 149.0, 137.9, 136.1, 135.8, 129.5, 129.3, 128.7, 126.7, 118.5, 117.6, 117.1, 114.4, 112.4, 111.4, 74.6, 70.9, 55.8, 55.3, 53.8, 50.1, 49.6, 48.4, 44.5, 36.7, 30.1, 29.1, 25.7, 24.3, 22.6 ppm. HRMS-ESI *(m/z)*: $[M+H]^+$ Calcd for $C_{38}H_{53}N_4O_5$ 645.4016; Found 645.4012. Optical rotation $[\alpha]^{20}_D$ –39.3 (*c* 2.03, $CHCl_3$).

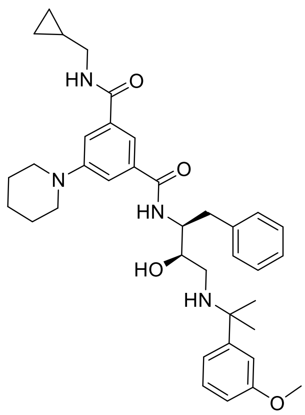

***N*¹-(Cyclopropylmethyl)-*N*³-[(2*S*,3*R*)-3-hydroxy-4-{[2-(3-methoxyphenyl)propan-2-yl]amino}-1-phenylbutan-2-yl]-5-(piperidin-1-yl)isophthalamide ((*S*,*R*)-3b)**.

The title compound was obtained from *rac*-**11** (50 mg, 0.12 mmol, 1.0 eq), benzoic acid **15h** (38 mg, 0.12 mmol, 1.0 eq), HBTU (47 mg, 0.12 mmol, 1.0 eq) and TEA (70 µL, 0.50 mmol, 4.0 eq) by following general procedure D. Purification by column chromatography on silica gel using gradient elution from 50% EtOAc in petroleum ether to 100% EtOAc, followed by 5% MeOH in EtOAc afforded *rac*-**3b** as a white solid (48 mg, 63% yield).

Enantiomerically pure material (*S*,*R*)-**3b** (17 mg, 22% yield) was obtained by semi-preparative HPLC on chiral stationary phase (*Chiralpak-IC*), using 40% EtOAc/60% $CHCl_3$/0.1% DEA as a mobile phase (flow rate 2.0 mL/min, detector UV 254 nm).

^{1}H-NMR (400 MHz, $CDCl_3$) δ: 7.46 (dd, *J* = 2.6, 1.4 Hz, 1H, NH), 7.29-7.26 (m, 1H, NH), 7.25-7.15 (m, 7H, Ar-H), 7.03-6.96 (m, 2H, Ar-H), 6.80-6.70 (m, 2H, Ar-H), 6.49-6.40 (m, 1H, Ar-H), 4.40-4.31 (m, 1H, CH), 3.77 (s, 3H, OCH_3), 3.54-3.50 (m, 1H, CH), 3.28 (ddd, *J* = 7.2, 5.4, 1.9 Hz, 2H, NCH_2), 3.22-3.16 (m, 4H, NCH_2×2), 2.97 (d, *J* = 6.7 Hz, 2H, CH_2), 2.49-2.40 (m, 2H, CH_2), 1.71-1.64 (m, 4H, CH_2×2), 1.63-1.55 (m, 2H, CH_2), 1.48 (s, 3H, CH_3), 1.47 (s, 3H, CH_3), 0.93-0.80 (m, 1H, CH), 0.60-0.50 (m, 2H, CH_2), 0.31-0.21 (m, 2H, CH_2) ppm. ^{13}C-NMR (101 MHz, $CDCl_3$) δ: 168.0, 167.3, 159.8, 152.4, 137.9, 136.1, 135.6, 129.6, 129.4, 128.7, 126.7, 118.5, 117.9, 116.9, 114.4, 112.4, 111.9, 70.7, 55.4, 53.8, 50.0, 45.2, 44.7, 36.7, 29.8, 28.5, 25.7, 24.3, 10.9, 3.8 ppm. HRMS-ESI *(m/z)*: $[M+H]^+$ Calcd for $C_{37}H_{49}N_4O_4$ 613.3754; Found 613.3769. Optical rotation $[\alpha]^{20}_D$ –40.3 (*c* 1.32, $CHCl_3$).

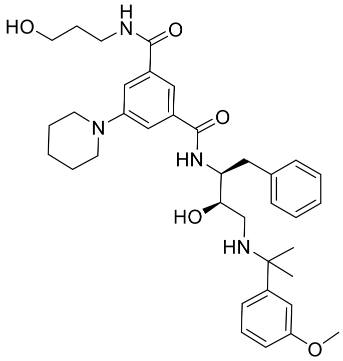

***N*¹-[(2*S*,3*R*)-3-Hydroxy-4-{[2-(3-methoxyphenyl)propan-2-yl]amino}-1-phenylbutan-2-yl]-*N*³-(3-hydroxypropyl)-5-(piperidin-1-yl)isophthalamide ((*S*,*R*)-3c)**.

The title compound was obtained from (*R*,*S*)-**11** (32 mg, 0.080 mmol, 1.0 eq), benzoic acid **15i** (24 mg, 0.080 mmol, 1.0 eq), HBTU (30 mg, 0.080 mmol, 1.0 eq) and TEA (44 µL, 0.32 mmol, 4.0 eq) by following general procedure D. Purification by column chromatography on amino-functionalized silica gel using gradient elution from 100% EtOAc to 2% MeOH in EtOAc afforded (*S*,*R*)-**3c** as a white solid (37 mg, 75% yield).

^{1}H-NMR (400 MHz, $CDCl_3$) δ: 7.46 (dd, *J* = 2.6, 1.4 Hz, 1H, NH), 7.28-7.26 (m, 1H, NH), 7.25-7.14 (m, 7H, Ar-H), 7.05-6.88 (m, 4H, Ar-H), 6.77-6.71 (m, 1H, Ar-H), 4.40-4.30 (m, 1H, CH), 3.77 (s, 3H, OCH_3), 3.68 (t, *J* = 5.5 Hz, 2H, OCH_2), 3.62-3.49 (m, 3H, NCH_2, CH), 3.22-3.15 (m, 4H, NCH_2×2), 2.93 (dd, *J* = 6.8, 3.9 Hz, 2H, CH_2), 2.54-2.41 (m, 2H, CH_2), 1.76 (quintet, *J* = 5.7 Hz, 2H, CH_2), 1.71-1.63 (m, 4H, CH_2×2), 1.62-1.56 (m, 2H, CH_2), 1.47 (s, 3H, CH_3), 1.45 (s, 3H, CH_3) ppm. ^{13}C-NMR (101 MHz, $CDCl_3$) δ: 168.3, 168.1, 159.7, 152.4, 148.8, 138.0, 135.9, 135.4, 129.5, 129.4, 128.7, 126.7, 118.5, 117.6, 117.0, 114.2, 112.5, 111.6, 70.7, 59.8, 55.9, 55.4, 54.2, 49.9, 44.5, 37.3, 36.8, 32.2, 30.4, 28.7, 25.7, 24.3 ppm. HRMS-ESI *(m/z)*: $[M+H]^+$ Calcd for $C_{36}H_{49}N_4O_5$ 617.3703; Found 617.3705. Optical rotation $[\alpha]^{20}_D$ –45.9 (*c* 2.51, $CHCl_3$).

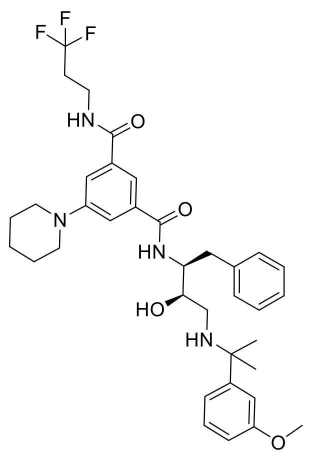

***N*[1]-[(2*S*,3*R*)-3-Hydroxy-4-{[2-(3-methoxyphenyl)propan-2-yl]amino}-1-phenylbutan-2-yl]-5-(piperidin-1-yl)-*N*[3]-(3,3,3-trifluoropropyl)isophthalamide** ((*S*,*R*)-**3d**).

The title compound was obtained from *rac*-**11** (50 mg, 0.12 mmol, 1.0 eq), benzoic acid **15j** (42 mg, 0.12 mmol, 1.0 eq), HBTU (47 mg, 0.12 mmol, 1.0 eq) and TEA (70 µL, 0.50 mmol, 4.0 eq) by following general procedure D. Purification by column chromatography on silica gel using gradient elution from 100% EtOAc to 2% MeOH in EtOAc afforded *rac*-**3d** as a colorless sticky oil (60 mg, 73% yield). Enantiomerically pure material (*S*,*R*)-**3d** (21 mg, 26% yield) was obtained by semi-preparative HPLC on chiral stationary phase (*Chiralpak-IC*), using 40% EtOAc/60% $CHCl_3$/0.1% DEA as a mobile phase (flow rate 2.5 mL/min, detector UV 254 nm).

^{1}H-NMR (400 MHz, $CDCl_3$) δ: 7.47-7.42 (m, 1H, NH), 7.30-7.26 (m, 1H, NH), 7.25-7.13 (m, 7H, Ar-H), 7.03-6.96 (m, 1H, Ar-H), 6.92-6.82 (m, 1H, Ar-H), 6.73 (ddd, *J* = 8.2, 2.5, 0.9 Hz, 1H, Ar-H), 6.70-6.54 (m, 2H, Ar-H), 4.40-4.30 (m, 1H, CH), 3.80-3.77 (m, 3H, OCH_3), 3.70-3.63 (m, 2H, NCH_2), 3.58-3.48 (m, 1H, CH), 3.25-3.17 (m, 4H, NCH_2×2), 2.98-2.92 (m, 2H, CH_2), 2.52-2.39 (m, 4H, CH_2×2), 1.72-1.64 (m, 4H, CH_2×2), 1.63-1.57 (m, 2H, CH_2), 1.49-1.45 (m, 4H, CH_3), 1.39 (d, *J* = 5.5 Hz, 2H, CH_3) ppm. ^{13}C-NMR (101 MHz, $CDCl_3$) δ: 168.0, 167.5, 159.7, 152.4, 137.8, 135.9, 135.3, 129.5, 129.4, 128.7, 126.8, 118.5, 117.5, 117.1, 114.2, 112.5, 111.9, 70.7, 56.3, 55.4, 53.8, 49.9, 44.6, 36.7, 33.9, 33.6, 30.1, 28.5, 25.7, 24.3 ppm. HRMS-ESI *(m/z)*: $[M+H]^+$ Calcd for $C_{36}H_{46}N_4O_4F_3$ 655.3471; Found 655.3477. Optical rotation $[\alpha]^{20}_D$ –31.8 (*c* 0.33, $CHCl_3$).

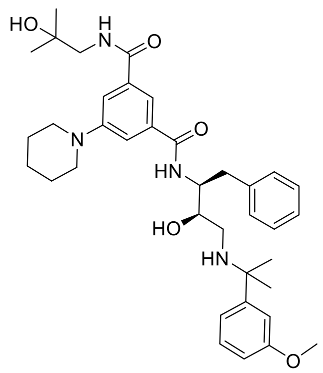

***N*[1]-(2-Hydroxy-2-methylpropyl)-*N*[3]-[(2*S*,3*R*)-3-hydroxy-4-{[2-(3-methoxyphenyl)propan-2-yl]amino}-1-phenylbutan-2-yl]-5-(piperidin-1-yl)isophthalamide** ((*S*,*R*)-**3e**).

The title compound was obtained from (*R*,*S*)-**11** (32 mg, 0.080 mmol, 1.0 eq), benzoic acid **15k** (26 mg, 0.080 mmol, 1.0 eq), HBTU (30 mg, 0.080 mmol, 1.0 eq) and TEA (44 µL, 0.32 mmol, 4.0 eq) by following general procedure D. Purification by column chromatography on amino-functionalized silica gel using gradient elution from 50% EtOAc in petroleum ether to 100% EtOAc afforded (*S*,*R*)-**3e** as a white solid (30 mg, 60% yield).

^{1}H-NMR (400 MHz, $CDCl_3$) δ: 7.43 (dd, *J* = 2.6, 1.4 Hz, 1H, NH), 7.31-7.28 (m, 1H, NH), 7.24-7.12 (m, 8H, Ar-H), 7.00-6.91 (m, 3H, Ar-H), 6.73 (ddd, *J* = 8.2, 2.5, 1.0 Hz, 1H, Ar-H), 4.39-4.30 (m, 1H, CH), 3.76 (s, 3H, OCH_3), 3.57-3.50 (m, 1H, CH), 3.49-3.38 (m, 2H, NCH_2), 3.19-3.12 (m, 4H, NCH_2×2), 2.99-2.83 (m, 2H, CH_2), 2.54-2.40 (m, 2H, CH_2), 1.69-1.61 (m, 4H, CH_2×2), 1.61-1.54

(m, 2H, CH_2), 1.46 (s, 3H, CH_3), 1.44 (s, 3H, CH_3), 1.26 (s, 6H, CH_3×2) ppm. ^{13}C-NMR (101 MHz, $CDCl_3$) δ: 168.5, 168.1, 159.7, 152.3, 148.8, 138.0, 135.9, 135.6, 129.4, 129.3, 128.7, 126.6, 118.5, 117.7, 117.1, 114.4, 112.5, 111.6, 71.2, 70.6, 55.9, 55.4, 54.4, 51.1, 49.9, 44.5, 36.7, 30.3, 28.8, 27.6, 27.5, 25.7, 24.3 ppm. HRMS-ESI *(m/z)*: $[M+H]^+$ Calcd for $C_{37}H_{51}N_4O_5$ 631.3859; Found 631.3856. Optical rotation $[\alpha]^{20}_D$ –46.9 (*c* 2.20, $CHCl_3$).

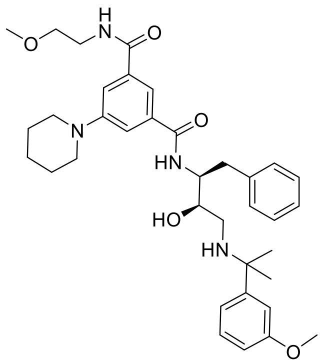

***N*¹-[(2*S*,3*R*)-3-Hydroxy-4-{[2-(3-methoxyphenyl)propan-2-yl]amino}-1-phenylbutan-2-yl]-*N*³-(2-methoxyethyl)-5-(piperidin-1-yl)isophthalamide ((*S*,*R*)-3f)**.

The title compound was obtained from (*R*,*S*)-**11** (35 mg, 0.087 mmol, 1.0 eq), benzoic acid **15l** (27 mg, 0.087 mmol, 1.0 eq), HBTU (33 mg, 0.087 mmol, 1.0 eq) and TEA (49 µL, 0.35 mmol, 4.0 eq) by following general procedure D. Purification by column chromatography on amino-functionalized silica gel using gradient elution from 25% EtOAc in petroleum ether to 100% EtOAc afforded (*S*,*R*)-**3f** as a colorless sticky oil (36 mg, 67% yield).

^{1}H-NMR (400 MHz, $CDCl_3$) δ: 7.46-7.43 (m, 1H, NH), 7.30-7.27 (m, 1H, NH), 7.25-7.16 (m, 7H, Ar-H), 7.02-6.97 (m, 2H, Ar-H), 6.77-6.71 (m, 1H, Ar-H), 6.67-6.56 (m, 2H, Ar-H), 4.40-4.30 (m, 1H, CH), 3.77 (s, 3H, OCH_3), 3.67-3.60 (m, 2H, OCH_2), 3.58-3.48 (m, 3H, NH_2, CH), 3.37 (s, 3H, OCH_3), 3.22-3.17 (m, 4H, NCH_2×2), 3.06-2.79 (m, 4H, CH_2, NH, OH), 2.46 (d, J = 4.2 Hz, 2H, CH_2), 1.72-1.64 (m, 4H, CH_2×2), 1.63-1.56 (m, 2H, CH_2), 1.48 (s, 3H, CH_3), 1.48 (s, 3H, CH_3) ppm. ^{13}C-NMR (101 MHz, $CDCl_3$) δ: 167.9, 167.4, 159.7, 152.4, 137.9, 135.9, 135.8, 129.6, 129.3, 128.7, 126.7, 118.5, 117.7, 117.1, 114.4, 112.4, 111.6, 71.3, 70.8, 59.0, 56.0, 55.3, 53.8, 50.0, 44.6, 40.0, 36.7, 30.2, 28.9, 25.7, 24.3 ppm. HRMS-ESI *(m/z)*: $[M+H]^+$ Calcd for $C_{36}H_{49}N_4O_5$ 617.3703; Found 617.3708. Optical rotation $[\alpha]^{20}_D$ –36.3 (*c* 2.39, $CHCl_3$).

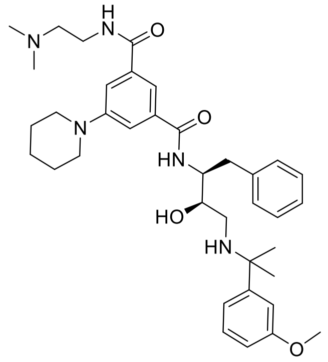

***N*¹-[2-(Dimethylamino)ethyl]-*N*³-[(2*S*,3*R*)-3-hydroxy-4-{[2-(3-methoxyphenyl)propan-2-yl]amino}-1-phenylbutan-2-yl]-5-(piperidin-1-yl)isophthalamide ((*S*,*R*)-3g)**.

The title compound was obtained from (*R*,*S*)-**11** (30 mg, 0.075 mmol, 1.0 eq), benzoic acid **15m** (24 mg, 0.075 mmol, 1.0 eq), HBTU (28 mg, 0.075 mmol, 1.0 eq) and TEA (42 µL, 0.30 mmol, 4.0 eq) by following general procedure D. Purification by column chromatography on amino-functionalized silica gel using gradient elution from 100% EtOAc to 10% MeOH in EtOAc afforded (*S*,*R*)-**3g** as a yellowish sticky oil (27 mg, 57% yield).

^{1}H-NMR (400 MHz, $CDCl_3$) δ: 7.51-7.44 (m, 1H, NH), 7.38-7.30 (m, 1H, NH), 7.29-7.26 (m, 1H, Ar-H), 7.25-7.13 (m, 6H, Ar-H), 7.02-6.95 (m, 2H, Ar-H) 6.89-6.83 (m, 1H, Ar-H), 6.81-6.76 (m, 1H, Ar-H), 6.75-6.70 (m, 1H, Ar-H), 4.40-4.31 (m, 1H, CH), 3.77 (s, 3H, OCH_3), 3.55-3.47 (m, 3H, NCH_2, CH), 3.26-3.17 (m, 4H NCH_2×2), 3.08-2.67 (m, 4H, CH_2, NH, OH), 2.51 (t, J = 6.0 Hz, 2H, NCH_2), 2.46 (d, J = 4.3 Hz, 2H, CH_2), 2.26 (s, 6H, NCH_3×2), 1.71-1.64 (m, 4H, CH_2×2), 1.62-1.55 (m, 2H, CH_2), 1.49-1.43 (m, 6H, CH_3×2) ppm. ^{13}C-NMR (101 MHz, $CDCl_3$) δ: 167.8, 167.5, 159.7, 152.3, 149.0, 138.0, 136.0, 135.7, 129.5, 129.3, 128.7, 126.7, 118.5, 117.8, 117.1, 114.5, 112.4, 111.5, 70.9, 58.0, 55.8, 55.3, 53.9, 50.1, 45.3, 44.6, 37.5, 36.7, 30.2, 29.0, 25.7, 24.3 ppm. HRMS-ESI *(m/z)*: $[M+H]^+$ Calcd for $C_{37}H_{52}N_5O_4$ 630.4019; Found 630.4048. Optical rotation $[\alpha]^{20}_{D}$ –39.2 (*c* 1.97, $CHCl_3$).

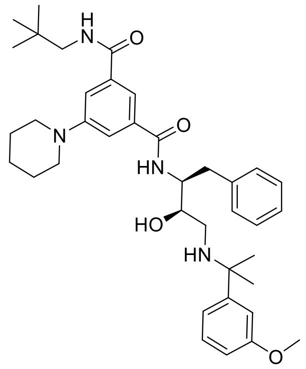

***N*^1-[(2*S*,3*R*)-3-Hydroxy-4-{[2-(3-methoxyphenyl)propan-2-yl]amino}-1-phenylbutan-2-yl]-*N*^3-neopentyl-5-(piperidin-1-yl)isophthalamide ((*S*,*R*)-3h)**.

The title compound was obtained from *rac*-**11** (60 mg, 0.15 mmol, 1.0 eq), benzoic acid **15n** (52 mg, 0.16 mmol, 1.1 eq), HBTU (57 mg, 0.15 mmol, 1.0 eq) and TEA (83 µL, 0.60 mmol, 4.0 eq) by following general procedure D. Purification by column chromatography on silica gel using gradient elution from 100% EtOAc to 10% MeOH in EtOAc afforded *rac*-**3h** as a light yellowish solid (66 mg, 70% yield). Enantiomerically pure material (*S*,*R*)-**3h** (31 mg, 33% yield) was obtained by preparative HPLC on chiral stationary phase (*Chiralpak-ID*), using 85% EtOAc/15% $CHCl_3$/0.1% DEA as a mobile phase (flow rate 20 mL/min, detector UV 254 nm).

^{1}H-NMR (400 MHz, $CDCl_3$) δ: 7.47 (dd, J = 2.6, 1.4 Hz, 1H, NH), 7.32-7.30 (m, 1H, NH), 7.24-7.16 (m, 7H, Ar-H), 7.04-6.98 (m, 2H, Ar-H), 6.84 (d, J = 8.6 Hz, 1H, Ar-H), 6.79-6.73 (m, 1H, Ar-H), 6.52-6.46 (m, 1H, Ar-H), 5.26-5.04 (m, 2H, NH, OH), 4.39-4.30 (m, 1H, CH), 3.76 (s, 3H, OCH_3), 3.73-3.67 (m, 1H, CH), 3.24 (d, J = 6.4 Hz, 2H, NCH_2), 3.21-3.17 (m, 4H, NCH_2×2), 3.06-2.92 (m, 2H, CH_2), 2.61-2.46 (m, 2H, CH_2), 1.71-1.63 (m, 4H, CH_2×2), 1.62-1.53 (m, 8H, CH_2, CH_3×2), 0.96 (s, 9H, CH_3×3) ppm. ^{13}C-NMR (101 MHz, $CDCl_3$) δ: 168.0, 167.7, 159.9, 152.4, 137.8, 136.3, 135.4, 129.7, 129.6, 128.7, 126.7, 118.3, 117.9, 117.0, 114.3, 112.4, 70.2, 57.8, 55.4, 53.6, 51.2, 50.0, 45.4, 36.3, 32.5, 28.7, 27.7, 27.5, 25.7, 24.3 ppm. HRMS-ESI *(m/z)*: $[M+H]^+$ Calcd for $C_{38}H_{53}N_4O_4$ 629.4067; Found 629.4067. Optical rotation $[\alpha]^{20}_{D}$ –40.6 (*c* 0.59, $CHCl_3$).

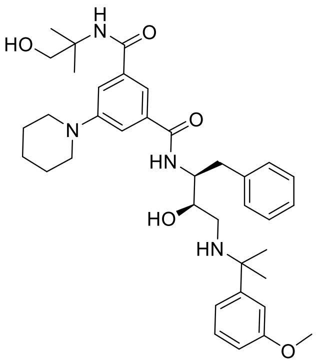

N^1-(1-Hydroxy-2-methylpropan-2-yl)-N^3-[(2*S*,3*R*)-3-hydroxy-4-{[2-(3-methoxyphenyl)propan-2-yl]amino}-1-phenylbutan-2-yl]-5-(piperidin-1-yl)isophthalamide ((*S*,*R*)-3i).

The title compound was obtained from (*R*,*S*)-**11** (30 mg, 0.075 mmol, 1.0 eq), benzoic acid **15o** (24 mg, 0.075 mmol, 1.0 eq), HBTU (28 mg, 0.075 mmol, 1.0 eq) and TEA (42 µL, 0.30 mmol, 4.0 eq) by following general procedure D. Purification by column chromatography on amino-functionalized silica gel using gradient elution from 50% EtOAc in petroleum ether to 100% EtOAc afforded (*S*,*R*)-**3i** as a white solid (27 mg, 57% yield).

^{1}H-NMR (400 MHz, $CDCl_3$) δ: 7.37 (dd, *J* = 2.7, 1.4 Hz, 1H, NH), 7.28-7.26 (m, 1H, NH), 7.25-7.10 (m, 7H, Ar-H), 7.09-7.01 (m, 1H, Ar-H), 7.00-6.94 (m, 2H, Ar-H), 6.73 (ddt, *J* = 8.1, 2.3, 1.1 Hz, 1H, Ar-H), 6.42-6.35 (m, 1H, Ar-H), 4.39-4.30 (m, 1H, CH), 3.76 (s, 3H, OCH_3), 3.67 (d, *J* = 1.2 Hz, 2H, OCH_2), 3.57-3.52 (m, 1H, CH), 3.23-3.13 (m, 4H, NCH_2×2), 3.02-2.85 (m, 2H, CH_2), 2.54-2.41 (m, 2H, CH_2), 1.70-1.63 (m, 4H, CH_2×2), 1.62-1.53 (m, 2H, CH_2), 1.46 (s, 3H, CH_3), 1.45 (s, 3H, CH_3), 1.40 (s, 6H, CH_3×2) ppm. ^{13}C-NMR (101 MHz, $CDCl_3$) δ: 168.4, 167.9, 159.7, 152.3, 148.8, 137.9, 136.2, 135.8, 129.5, 129.4, 128.7, 126.7, 118.5, 117.5, 117.0, 114.4, 112.5, 111.4, 70.7, 70.6, 56.7, 55.9, 55.3, 54.3, 50.0, 44.5, 36.7, 30.3, 28.9, 25.7, 24.7, 24.2 ppm. HRMS-ESI *(m/z)*: $[M+H]^+$ Calcd for $C_{37}H_{51}N_4O_5$ 631.3859; Found 631.3839. Optical rotation $[\alpha]^{20}_D$ –41.2 (*c* 2.00, $CHCl_3$).

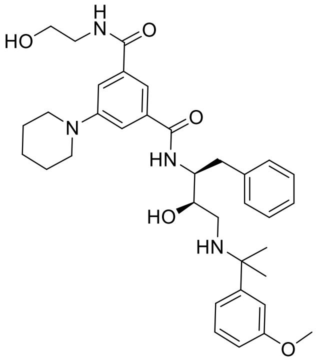

N^1-[(2*S*,3*R*)-3-Hydroxy-4-{[2-(3-methoxyphenyl)propan-2-yl]amino}-1-phenylbutan-2-yl]-N^3-(2-hydroxyethyl)-5-(piperidin-1-yl)isophthalamide ((*S*,*R*)-3j).

The title compound was obtained from (*R*,*S*)-**11** (32 mg, 0.080 mmol, 1.0 eq), benzoic acid **15p** (23 mg, 0.080 mmol, 1.0 eq), HBTU (30 mg, 0.080 mmol, 1.0 eq) and TEA (44 µL, 0.32 mmol, 4.0 eq) by following general procedure D. Purification by column chromatography on amino-functionalized silica gel using gradient elution from 100% EtOAc to 2% MeOH in EtOAc afforded (*S*,*R*)-**3j** as a white solid (37 mg, 77% yield).

^{1}H-NMR (400 MHz, $CDCl_3$) δ: 7.40-7.31 (m, 2H, NH×2), 7.25-7.08 (m, 9H, Ar-H), 7.02-6.95 (m, 2H, Ar-H), 6.74 (ddd, *J* = 8.2, 2.5, 1.1 Hz, 1H, Ar-H), 4.39-4.30 (m, 1H, CH), 3.80 (t, *J* = 4.9 Hz, 2H, OCH_2), 3.76 (s, 3H, OCH_3), 3.62-3.53 (m, 3H, CH, NCH_2), 3.22-3.10 (m, 4H, NCH_2×2), 3.00-2.81 (m, 2H, CH_2), 2.56-2.41 (m, 2H, CH_2), 1.68-1.61 (m, 4H, CH_2×2), 1.60-1.53 (m, 2H, CH_2), 1.46 (s, 3H, CH_3), 1.43 (s, 3H, CH_3) ppm. ^{13}C-NMR (101 MHz, $CDCl_3$) δ: 168.5, 168.3, 159.7, 152.2, 148.7, 138.0, 135.8, 135.4, 129.4, 128.7, 126.6, 118.5, 117.6, 117.0, 114.4, 112.5, 111.6, 70.7, 62.2, 55.9,

55.4, 54.6, 49.9, 44.6, 43.2, 36.6, 30.3, 28.6, 25.7, 24.3 ppm. HRMS-ESI *(m/z)*: $[M+H]^+$ Calcd for $C_{35}H_{47}N_4O_5$ 603.3546; Found 603.3544. Optical rotation $[\alpha]^{20}_D$ –49.7 (*c* 2.85, $CHCl_3$).

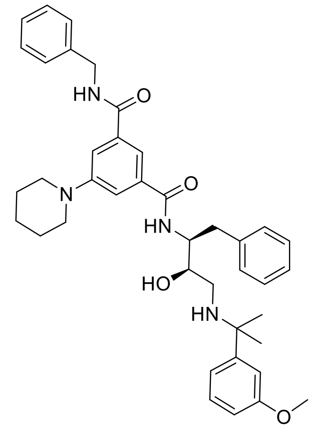

N^1-Benzyl-N^3-[(2*S*,3*R*)-3-hydroxy-4-{[2-(3-methoxyphenyl)propan-2-yl]amino}-1-phenylbutan-2-yl]-5-(piperidin-1-yl)isophthalamide ((*S*,*R*)-3k).

The title compound was obtained from *rac*-**11** (50 mg, 0.12 mmol, 1.0 eq), benzoic acid **15q** (42 mg, 0.12 mmol, 1.0 eq), HBTU (47 mg, 0.12 mmol, 1.0 eq) and TEA (70 µL, 0.50 mmol, 4.0 eq) by following general procedure D. Purification by column chromatography on silica gel using gradient elution from 100% EtOAc to 3% MeOH in EtOAc afforded *rac*-**3k** as a yellowish sticky oil (60 mg, 74% yield). Enantiomerically pure material (*S*,*R*)-**3k** (18 mg, 22% yield) was obtained by semi-preparative HPLC on chiral stationary phase (*Chiralpak-IC*), using 40% EtOAc/60% $CHCl_3$/0.1% DEA as a mobile phase (flow rate 3.0 mL/min, detector UV 254 nm). ^{1}H-NMR (400 MHz, $CDCl_3$) δ: 7.51 (dd, *J* = 1.9 Hz, 1H, NH), 7.37-7.27 (m, 6H, NH, Ar-H), 7.23-7.10 (m, 7H, Ar-H), 7.02-6.95 (m, 2H, Ar-H), 6.86-6.75 (m, 2H, Ar-H), 6.73-6.68 (m, 1H, Ar-H), 4.61 (d, *J* = 5.8 Hz, 2H, NCH_2), 4.41-4.27 (m, 1H, CH), 3.75 (s, 3H, OCH_3), 3.59-3.53 (m, 1H, CH), 3.21-3.16 (m, 4H, NCH_2×2), 2.99-2.89 (m, 2H, CH_2), 2.55-2.40 (m, 2H, CH_2), 1.70-1.62 (m, 4H, CH_2×2), 1.62-1.56 (m, 2H, CH_2), 1.50 (s, 3H, CH_3), 1.49 (s, 3H, CH_3) ppm. ^{13}C-NMR (101 MHz, $CDCl_3$) δ: 168.2, 167.2, 159.8, 152.3, 138.5, 137.8, 135.6, 131.0, 129.6, 129.5, 128.8, 128.7, 128.1, 127.6, 126.7, 118.4, 118.0, 117.2, 114.3, 112.5, 55.4, 53.8, 49.9, 44.2, 38.9, 36.4, 29.8, 29.1, 25.7, 24.3 ppm. HRMS-ESI *(m/z)*: $[M+H]^+$ Calcd for $C_{40}H_{49}N_4O_4$ 649.3754; Found 649.3755. Optical rotation $[\alpha]^{20}_D$ –35.3 (*c* 1.20, $CHCl_3$).

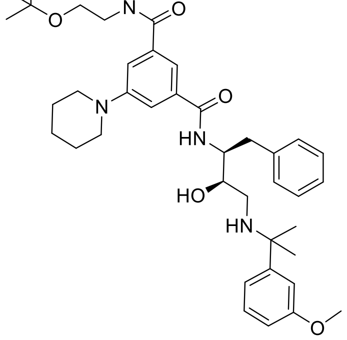

N^1-[2-(*tert*-Butoxy)ethyl]-N^3-[(2*S*,3*R*)-3-hydroxy-4-{[2-(3-methoxyphenyl)propan-2-yl]amino}-1-phenylbutan-2-yl]-5-(piperidin-1-yl)isophthalamide ((*S*,*R*)-3l).

The title compound was obtained from (*R*,*S*)-**11** (35 mg, 0.087 mmol, 1.0 eq), benzoic acid **15r** (30 mg, 0.087 mmol, 1.0 eq), HBTU (33 mg, 0.087 mmol, 1.0 eq) and TEA (49 µL, 0.35 mmol, 4.0 eq) by following general procedure D. Purification by column chromatography on amino-functionalized silica gel using gradient elution from 15% EtOAc in petroleum ether to 100% EtOAc afforded (*S*,*R*)-**3l** as a colorless sticky oil (33 mg, 57% yield).

^{1}H-NMR (400 MHz, $CDCl_3$) δ: 7.44-7.40 (m, 1H, NH), 7.30-7.27 (m, 1H, NH), 7.26-7.17 (m, 7H, Ar-H), 7.02-6.96 (m, 2H, Ar-H), 6.76-6.70 (m, 1H, Ar-H), 6.68-6.57 (m, 2H, Ar-H), 4.39-4.31 (m,

1H, CH), 3.77 (s, 3H, OCH_3), 3.62-3.45 (m, 6H, OCH_2, NCH_2, CH, NH), 3.25-3.15 (m, 4H, NCH_2×2), 3.06-2.92 (m, 3H, CH_2, OH), 2.49-2.43 (m, 2H, CH_2), 1.71-1.64 (m, 4H, CH_2×2), 1.63-1.57 (m, 2H, CH_2), 1.48 (s, 6H, CH_3×2), 1.21-1.18 (m, 9H, CH_3×3) ppm. ^{13}C-NMR (101 MHz, $CDCl_3$) δ: 167.8, 167.4, 159.7, 152.3, 148.8, 137.9, 136.1, 135.8, 129.6, 129.3, 128.7, 126.7, 118.5, 117.4, 117.0, 114.4, 112.4, 111.6, 73.4, 70.8, 60.6, 55.9, 55.3, 53.7, 50.0, 44.5, 40.8, 36.7, 30.1, 29.0, 27.7, 25.7, 24.3 ppm. HRMS-ESI *(m/z)*: $[M+H]^+$ Calcd for $C_{39}H_{55}N_4O_5$ 659.4172; Found 659.4191. Optical rotation $[\alpha]^{20}_D$ –34.6 (*c* 2.31, $CHCl_3$).

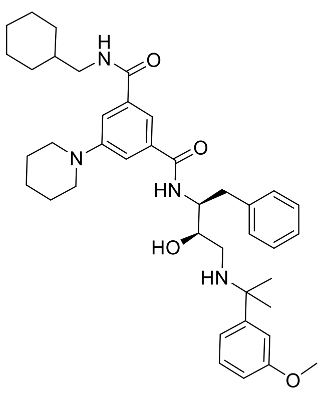

N^1-(Cyclohexylmethyl)-N^3-[(2*S*,3*R*)-3-hydroxy-4-{[2-(3-methoxyphenyl)propan-2-yl]amino}-1-phenylbutan-2-yl]-5-(piperidin-1-yl)isophthalamide ((*S*,*R*)-3m).

The title compound was obtained from (*R*,*S*)-**11** (25 mg, 0.062 mmol, 1.0 eq), benzoic acid **15s** (24 mg, 0.069 mmol, 1.1 eq), HBTU (24 mg, 0.062 mmol, 1.0 eq) and TEA (35 µL, 0.25 mmol, 4.0 eq) by following general procedure D. Purification by column chromatography on silica gel using 1% MeOH in EtOAc afforded (*S*,*R*)-**3m** as a white solid (28 mg, 69% yield).

^{1}H-NMR (400 MHz, $CDCl_3$) δ: 7.45 (dd, *J* = 2.5, 1.4 Hz, 1H, NH), 7.29-7.26 (m, 1H, NH), 7.25-7.15 (m, 7H, Ar-H), 7.03-6.97 (m, 2H, Ar-H), 6.76-6.68 (m, 2H, Ar-H), 6.32 (d, *J* = 6.3 Hz, 1H, Ar-H), 4.40-4.30 (m, 1H, CH), 3.77 (s, 3H, OCH_3), 3.54-3.48 (m, 1H, CH), 3.30-3.23 (m, 2H, NCH_2), 3.22-3.16 (m, 4H, NCH_2×2), 3.03-2.91 (m, 2H, CH_2), 2.46 (d, *J* = 4.3 Hz, 2H, CH_2), 1.80-1.71 (m, 4H, CH_2×2), 1.70-1.63 (m, 5H, CH_2×2, CH), 1.62-1.54 (m, 3H, CH_2, OH), 1.47 (s, 3H, CH_3), 1.47 (s, 3H, CH_3), 1.32-1.08 (m, 4H, CH_2×2), 1.04-0.91 (m, 2H, CH_2) ppm. ^{13}C-NMR (101 MHz, $CDCl_3$) δ: 168.0, 167.4, 159.7, 152.4, 137.9, 136.2, 135.8, 129.6, 129.3, 128.7, 126.7, 118.5, 117.7, 116.8, 114.2, 112.4, 111.6, 70.8, 55.9, 55.4, 53.9, 50.0, 46.5, 44.5, 38.8, 38.2, 36.7, 31.1, 30.3, 28.8, 26.5, 26.0, 25.7, 24.3 ppm. HRMS-ESI *(m/z)*: $[M+H]^+$ Calcd for $C_{40}H_{55}N_4O_4$ 655.4223; Found 655.4229. Optical rotation $[\alpha]^{20}_D$ –33.2 (*c* 2.00, $CHCl_3$).

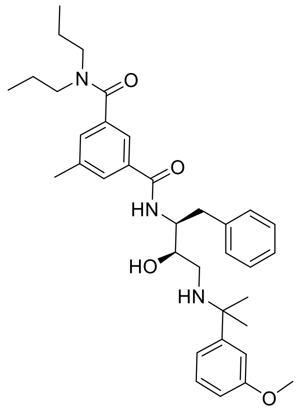

***N*[1]-[(2*S*,3*R*)-3-Hydroxy-4-{[2-(3-methoxyphenyl)propan-2-yl]amino}-1-phenylbutan-2-yl]-5-methyl-*N*[3],*N*[3]-dipropylisophthalamide ((*S*,*R*)-4a)**.

The title compound was obtained from *rac*-**11** (50 mg, 0.12 mmol, 1.0 eq), benzoic acid **17b** (33 mg, 0.12 mmol, 1.0 eq), HBTU (47 mg, 0.12 mmol, 1.0 eq) and TEA (70 µL, 0.50 mmol, 4.0 eq) by following general procedure D. Purification by column chromatography on silica gel using gradient elution from 100% EtOAc to 2% MeOH in EtOAc afforded *rac*-**4a** as a colorless sticky oil (50 mg, 70% yield). Enantiomerically pure material (*S*,*R*)-**4a** (17 mg, 24% yield) was obtained by semi-preparative HPLC on chiral stationary phase (*Chiralpak-IC*), using 60% EtOAc/40% $CHCl_3$/0.1% DEA as a mobile phase (flow rate 2.5 mL/min, detector UV 270 nm).

^{1}H-NMR (400 MHz, $CDCl_3$) δ: 7.43-7.37 (m, 2H, NH, Ar-H), 7.29-7.26 (m, 5H, Ar-H), 7.24-7.15 (m, 2H, Ar-H), 6.95-6.88 (m, 2H, Ar-H), 6.70 (ddd, *J* = 8.2, 2.6, 0.9 Hz, 1H, Ar-H), 6.52 (d, *J* = 9.0 Hz, 1H, Ar-H), 4.24-4.15 (m, 1H, CH), 3.75 (s, 3H, OCH_3), 3.56 (dd, *J* = 9.9, 3.9 Hz, 1H, CH), 3.51-3.40 (m, 2H, NCH_2), 3.19-3.08 (m, 2H, NCH_2), 3.06-2.91 (m, 2H, CH_2), 2.45 (dd, *J* = 12.3, 4.0 Hz, 1H, CH (CH_2)), 2.40 (s, 3H, Ar-CH_3), 2.27 (dd, *J* = 12.2, 9.7 Hz, 1H, CH (CH_2)), 1.75-1.65 (m, 2H, CH_2), 1.57-1.47 (m, 2H, CH_2), 1.42 (s, 3H, CH_3), 1.40 (s, 3H, CH_3), 1.04-0.95 (m, 3H, CH_3), 0.79-0.68 (m, 3H, CH_3) ppm. ^{13}C-NMR (101 MHz, $CDCl_3$) δ: 171.1, 167.4, 159.8, 148.7, 139.0, 137.9, 137.8, 134.9, 130.1, 129.5, 129.4, 128.7, 128.4, 126.7, 122.0, 118.5, 112.5, 111.5, 70.8, 56.0, 55.3, 53.9, 46.5, 44.5, 36.9, 30.0, 29.1, 22.0, 21.4, 20.9, 11.6, 11.2. ppm. HRMS-ESI *(m/z)*: $[M+H]^+$ Calcd for $C_{35}H_{48}N_3O_4$ 574.3645; Found 574.3670. Optical rotation $[\alpha]^{20}_{D}$ –32.4 (*c* 1.02, $CHCl_3$).

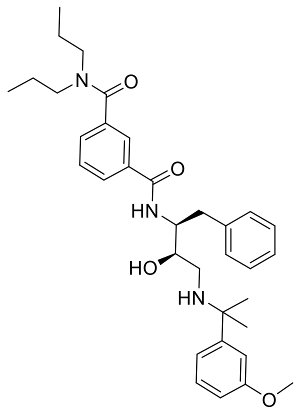

***N*[1]-[(2*S*,3*R*)-3-Hydroxy-4-{[2-(3-methoxyphenyl)propan-2-yl]amino}-1-phenylbutan-2-yl]-*N*[3],*N*[3]-dipropylisophthalamide ((*S*,*R*)-4b)**.

The title compound was obtained from *rac*-**11** (50 mg, 0.12 mmol, 1.0 eq), benzoic acid **17d** (31 mg, 0.12 mmol, 1.0 eq), HBTU (47 mg, 0.12 mmol, 1.0 eq) and TEA (70 µL, 0.50 mmol, 4.0 eq) by following general procedure D. Purification by column chromatography on silica gel using gradient elution from 100% EtOAc to 2% MeOH in EtOAc afforded *rac*-**4b** as a colorless sticky oil (45 mg, 64% yield). Enantiomerically pure material (*S*,*R*)-**4b** (16 mg, 23% yield) was obtained by semi-preparative HPLC on chiral stationary phase (*Chiralpak-IC*), using 40% EtOAc/60% $CHCl_3$/0.1% DEA as a mobile phase (flow rate 2.5 mL/min, detector UV 254 nm).

^{1}H-NMR (400 MHz, $CDCl_3$) δ: 7.67-7.62 (m, 1H, NH), 7.60-7.57 (m, 1H, Ar-H), 7.46-7.37 (m, 2H, Ar-H), 7.26-7.15 (m, 6H, Ar-H), 7.05-6.99 (m, 2H, Ar-H), 6.85 (d, *J* = 8.6 Hz, 1H, Ar-H), 6.77 (ddd,

J = 8.1, 2.3, 1.0 Hz, 1H, Ar-H), 4.46-4.35 (m, 1H, CH), 3.78 (s, 3H, OCH_3), 3.60-3.54 (m, 1H, CH), 3.50-3.40 (m, 2H, NCH_2), 3.16-3.07 (m, 2H, NCH_2), 3.03-2.95 (m, 2H, CH_2), 2.59-2.45 (m, 2H, CH_2), 1.75-1.63 (m, 2H, CH_2), 1.59-1.44 (m, 8H, CH_2, CH_3×2), 1.03-0.93 (m, 3H, CH_3), 0.77-0.69 (m, 3H, CH_3) ppm. ^{13}C-NMR (75 MHz, $CDCl_3$) δ: 170.8, 167.4, 159.9, 137.9, 137.6, 134.6, 129.6, 129.5, 128.9, 128.8, 127.7, 126.8, 125.2, 118.4, 112.5, 70.5, 55.4, 53.6, 50.9, 46.6, 45.0, 36.7, 29.9, 28.5, 22.1, 20.9, 14.3, 11.6, 11.2 ppm. HRMS-ESI *(m/z)*: $[M+H]^+$ Calcd for $C_{34}H_{46}N_3O_4$ 560.3488; Found 560.3507. Optical rotation $[\alpha]^{20}_D$ –24.1 (*c* 0.37, $CHCl_3$).

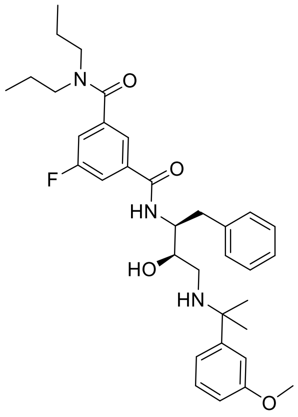

5-Fluoro-*N*^{1}-[(2*S*,3*R*)-3-hydroxy-4-{[2-(3-methoxyphenyl)propan-2-yl]amino}-1-phenylbutan-2-yl]-*N*^{3},*N*^{3}-dipropylisophthalamide ((*S*,*R*)-4c).

The title compound was obtained from (*R,S*)-**11** (19 mg, 0.047 mmol, 1.0 eq), benzoic acid **17e** (15 mg, 0.057 mmol, 1.2 eq), HBTU (18 mg, 0.047 mmol, 1.0 eq) and TEA (26 µL, 0.19 mmol, 4.0 eq) by following general procedure D. Purification by column chromatography on silica gel using gradient elution from 50% EtOAc in petroleum ether to 100% EtOAc afforded (*S*,*R*)-**4c** as a brown solid (17 mg, 62% yield).

^{1}H-NMR (400 MHz, $CDCl_3$) δ: 7.40 (ddd, *J* = 9.0, 2.5, 1.5 Hz, 1H, NH), 7.33 (dd, *J* = 1.5, 1.5 Hz, 1H, Ar-H), 7.25-7.10 (m, 8H, Ar-H), 7.01-6.97 (m, 2H, Ar-H), 6.77-6.73 (m, 1H, Ar-H), 4.40-4.30 (m, 1H, CH), 3.78 (s, 3H, OCH_3), 3.50 (dt, *J* = 6.4, 4.1 Hz, 1H, CH), 3.47-3.39 (m, 2H, NCH_2), 3.14-3.05 (m, 2H, NCH_2), 2.95 (d, *J* = 6.8 Hz, 2H, CH_2), 2.53-2.42 (m, 2H, CH_2), 1.72-1.62 (m, 2H, CH_2), 1.55-1.44 (m, 8H, CH_2, CH_3×2), 0.98 (t, *J* = 7.1 Hz, 3H, CH_3), 0.74 (t, *J* = 7.1 Hz, 3H, CH_3) ppm. ^{13}C-NMR (101 MHz, $CDCl_3$) δ: 169.4 (d, *J* = 2.2 Hz), 165.8 (d, *J* = 2.3 Hz), 163.8, 161.3, 159.8, 148.7, 139.7 (d, *J* = 6.8 Hz), 137.8, 137.5 (d, *J* = 7.1 Hz), 129.4, 128.7, 126.7, 120.5, 118.4, 116.6 (d, *J* = 23.0 Hz), 115.2 (d, *J* = 22.7 Hz), 112.7, 111.2, 70.6, 55.9, 55.3, 54.4, 50.8, 46.7, 44.4, 36.9, 30.1, 29.1, 22.0, 20.8, 11.6, 11.2 ppm. HRMS-ESI *(m/z)*: $[M+H]^+$ Calcd for $C_{34}H_{45}N_3O_4F$ 578.3394; Found 578.3397. Optical rotation $[\alpha]^{20}_D$ –32.8 (*c* 1.10, $CHCl_3$).

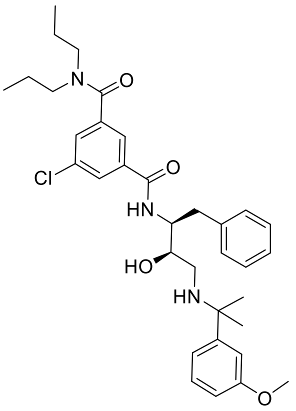

5-Chloro-N^1-[(2S,3R)-3-hydroxy-4-{[2-(3-methoxyphenyl)propan-2-yl]amino}-1-phenylbutan-2-yl]-N^3,N^3-dipropylisophthalamide ((*S*,*R*)-4d).

The title compound was obtained from *rac*-**11** (50 mg, 0.12 mmol, 1.0 eq), benzoic acid **17f** (35 mg, 0.12 mmol, 1.0 eq), HBTU (47 mg, 0.12 mmol, 1.0 eq) and TEA (70 µL, 0.50 mmol, 4.0 eq) by following general procedure D. Purification by column chromatography on silica gel using gradient elution from 100% EtOAc to 3% MeOH in EtOAc afforded *rac*-**4d** as a colorless sticky oil (34 mg, 46% yield). Enantiomerically pure material (*S*,*R*)-**4d** (12 mg, 16% yield) was obtained by preparative HPLC on chiral stationary phase (*Chiralpak-ID*), using 80% EtOAc/20% hexanes/0.1% DEA as a mobile phase (flow rate 15 mL/min, detector UV 270 nm).

^{1}H-NMR (400 MHz, $CDCl_3$) δ: 7.66-7.63 (m, 1H, NH), 7.46-7.41 (m, 1H, Ar-H), 7.40-7.35 (m, 1H, Ar-H), 7.28-7.26 (m, 1H, Ar-H), 7.24-7.15 (m, 6H, Ar-H), 7.01-6.96 (m, 2H, Ar-H), 6.78-6.73 (m, 1H, Ar-H), 4.42-4.31 (m, 1H, CH), 3.79 (s, 3H, OCH_3), 3.52 (d, J = 5.6 Hz, 1H, CH), 3.48-3.38 (m, 2H, NCH_2), 3.15-3.04 (m, 2H, NCH_2), 2.95 (d, J = 6.7 Hz, 2H, CH_2), 2.71 (br s, 2H, NH, OH), 2.56-2.42 (m, 2H, CH_2), 1.74-1.63 (m, 2H, CH_2), 1.54-1.45 (m, 8H, CH_2, CH_3×2), 1.03-0.94 (m, 3H, CH_3), 0.79-0.70 (m, 3H, CH_3) ppm. ^{13}C-NMR (101 MHz, $CDCl_3$) δ: 169.4, 165.8, 159.8, 139.3, 137.8, 136.8, 135.1, 129.4, 128.7, 128.0, 126.8, 123.2, 118.4, 112.7, 111.4, 110.2, 70.6, 56.1, 55.3, 54.4, 50.9, 46.7, 44.5, 36.9, 30.0, 29.0, 22.1, 20.8, 11.6, 11.2 ppm. HRMS-ESI *(m/z)*: $[M+H]^+$ Calcd for $C_{34}H_{45}N_3O_4Cl$ 594,3099; Found 594,3101. Optical rotation $[\alpha]^{20}_D$ –42.1 (*c* 0.82, $CHCl_3$).

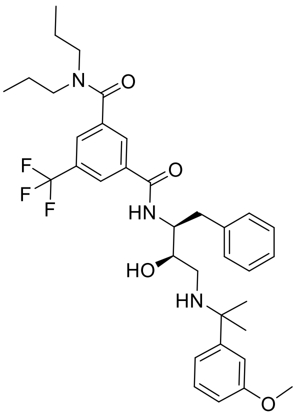

N^1-[(2S,3R)-3-Hydroxy-4-{[2-(3-methoxyphenyl)propan-2-yl]amino}-1-phenylbutan-2-yl]-N^3,N^3-dipropyl-5-(trifluoromethyl)isophthalamide ((*S*,*R*)-4e).

The title compound was obtained from *rac*-**11** (50 mg, 0.12 mmol, 1.0 eq), benzoic acid **17h** (47 mg, 0.15 mmol, 1.2 eq), HBTU (47 mg, 0.12 mmol, 1.0 eq) and TEA (70 µL, 0.50 mmol, 4.0 eq) by following general procedure D. Purification by column chromatography on silica gel using gradient elution from 100% EtOAc to 2% MeOH in EtOAc afforded *rac*-**4e** as a colorless sticky oil (30 mg, 38% yield). Enantiomerically pure material (*S*,*R*)-**4e** (15 mg, 19% yield) was obtained by preparative HPLC on chiral stationary phase (*Chiralpak-ID*), using 80% EtOAc/20% hexanes/0.1% DEA as a mobile phase (flow rate 15 mL/min, detector UV 270 nm).

^{1}H-NMR (400 MHz, $CDCl_3$) δ: 7.91-7.87 (m, 1H, Ar-H), 7.79-7.74 (m, 1H, Ar-H), 7.69-7.65 (m, 1H, Ar-H), 7.33-7.15 (m, 7H, NH, Ar-H), 7.03-6.95 (m, 2H, Ar-H), 6.75 (dd, J = 8.2, 2.6, Hz, 1H, Ar-H), 4.44-4.34 (m, 1H, CH), 3.78 (s, 3H, OCH_3), 3.58-3.52 (m, 1H, CH), 3.50-3.41 (m, 2H, NCH_2), 3.13-

3.04 (m, 2H, NCH_2), 2.95 (dd, J = 6.9, 2.4 Hz, 2H, CH_2), 2.90-2.70 (m, 2H, NH, OH), 2.50 (qd, J = 12.3, 4.2 Hz, 2H, CH_2), 1.80-1.61 (m, 2H, CH_2), 1.59-1.45 (m, 8H, CH_2, CH_3×2), 1.00 (t, J = 7.5 Hz, 3H, CH_3), 0.74 (t, J = 7.5 Hz, 3H, CH_3) ppm. ^{13}C-NMR (101 MHz, $CDCl_3$) δ: 169.4, 165.7, 159.8, 138.6, 137.7, 136.1, 131.7, 131.3, 129.5, 129.4, 128.8, 128.5, 126.8, 126.2, 124.7, 122.0, 118.4, 112.7, 111.6, 70.5, 56.3, 55.3, 54.5, 50.9, 46.8, 44.7, 36.8, 29.8, 28.9, 22.1, 20.8, 11.6, 11.1 ppm. ^{19}F-NMR (376 MHz, $CDCl_3$) δ: -62.8 ppm. HRMS-ESI *(m/z)*: $[M+H]^+$ Calcd for $C_{35}H_{45}N_3O_4F_3$ 628.3362; Found 628.3393. Optical rotation $[\alpha]^{20}_{D}$ –33.7 (*c* 1.10, $CHCl_3$).

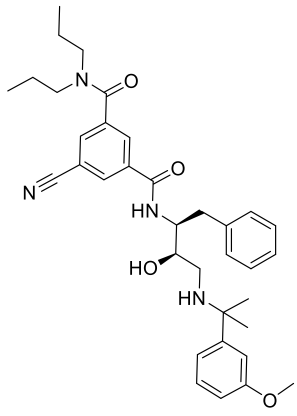

5-Cyano-*N*^{1}-[(2*S*,3*R*)-3-hydroxy-4-{[2-(3-methoxyphenyl)propan-2-yl]amino}-1-phenylbutan-2-yl]-*N*^{3},*N*^{3}-dipropylisophthalamide ((*S*,*R*)-4f).

The title compound was obtained from (*R*,*S*)-**11** (24 mg, 0.060 mmol, 1.0 eq), benzoic acid **17i** (16 mg, 0.060 mmol, 1.0 eq), HBTU (23 mg, 0.060 mmol, 1.0 eq) and TEA (33 µL, 0.24 mmol, 4.0 eq) by following general procedure D. Purification by column chromatography on amino-functionalized silica gel using gradient elution from 25% EtOAc in petroleum ether to 100% EtOAc afforded (*S*,*R*)-**4f** as a colorless sticky oil (14 mg, 40% yield).

^{1}H-NMR (400 MHz, $CDCl_3$) δ: 7.95-7.92 (m, 1H, Ar-H), 7.83-7.79 (m, 1H, Ar-H), 7.70-7.66 (m, 1H, Ar-H), 7.56-7.49 (m, 1H, NH), 7.24-7.13 (m, 6H, Ar-H), 7.02-6.96 (m, 2H, Ar-H), 6.80-6.74 (m, 1H, Ar-H), 4.37 (quintet, J = 7.0 Hz, 1H, CH), 3.79 (s, 3H, OCH_3), 3.62-3.55 (m, 1H, CH), 3.48-3.41 (m, 2H, NCH_2), 3.12-3.04 (m, 2H, NCH_2), 2.97-2.72 (m, 4H, CH_2, NH, OH), 2.58-2.44 (m, 2H, CH_2), 1.74-1.62 (m, 2H, CH_2), 1.56-1.44 (m, 8H, CH_2, CH_3×2), 0.98 (t, J = 7.3 Hz, 3H, CH_3), 0.74 (t, J = 7.3 Hz, 3H, CH_3) ppm. ^{13}C-NMR (101 MHz, $CDCl_3$) δ: 168.6, 164.9, 159.8, 148.1, 139.0, 137.8, 136.5, 132.4, 131.3, 129.5, 129.3, 128.7, 126.8, 118.4, 117.4, 113.4, 112.8, 111.4, 70.4, 56.2, 55.4, 54.8, 51.0, 44.6, 36.8, 28.9, 22.1, 20.8, 11.6, 11.2 ppm. HRMS-ESI *(m/z)*: $[M+H]^+$ Calcd for $C_{35}H_{45}N_4O_4$ 585.3441; Found 585.3419. Optical rotation $[\alpha]^{20}_{D}$ –31.3 (*c* 1.15, $CHCl_3$).

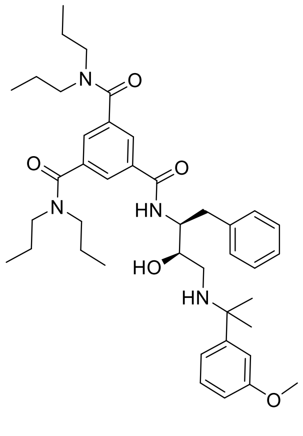

***N*^{1}-[(2*S*,3*R*)-3-Hydroxy-4-{[2-(3-methoxyphenyl)propan-2-yl]amino}-1-phenylbutan-2-yl]-*N*^{3},*N*^{3},*N*^{5},*N*^{5}-tetrapropylbenzene-1,3,5-tricarboxamide ((*S*,*R*)-4g)**.

The title compound was obtained from (*R*,*S*)-**11** (18 mg, 0.045 mmol, 1.0 eq), benzoic acid **17g** (20 mg, 0.054 mmol, 1.2 eq), HBTU (17 mg, 0.045 mmol, 1.0 eq) and TEA (25 µL, 0.18 mmol, 4.0 eq) by following general procedure D. Purification by column chromatography on silica gel using

gradient elution from 50% EtOAc in petroleum ether to 100% EtOAc afforded (*S*,*R*)-**4g** as a brownish solid (10 mg, 32% yield).

^{1}H-NMR (400 MHz, $CDCl_3$) δ: 7.66 (d, *J* = 1.5 Hz, 2H, NH, Ar-H), 7.44 (dd, *J* = 1.5, 1.5 Hz, 1H, Ar-H), 7.25-7.13 (m, 6H, Ar-H), 7.02-6.90 (m, 3H, Ar-H), 6.74 (ddd, *J* = 8.2, 2.5, 0.9 Hz, 1H, Ar-H), 4.41-4.32 (m, 1H, CH), 3.78 (s, 3H, OCH_3), 3.52-3.37 (m, 5H, CH, NCH_2×2), 3.18-3.07 (m, 4H, NCH_2×2), 3.00-2.93 (m, 2H, CH_2), 2.47 (dd, *J* = 4.3, 2.9 Hz, 2H, CH_2), 1.74-1.61 (m, 4H, CH_2×2), 1.55-1.42 (m, 10H, CH_2×2, CH_3×2), 1.02-0.93 (m, 6H, CH_3×2), 0.79-0.67 (m, 6H, CH_3×2) ppm. ^{13}C-NMR (101 MHz, $CDCl_3$) δ: 170.1, 166.2, 159.8, 138.2, 137.8, 135.2, 129.4, 128.7, 127.7, 126.7, 125.8, 118.4, 112.5, 111.4, 70.7, 55.3, 54.1, 50.9, 46.6, 44.6, 36.7, 29.8, 29.3, 22.1, 20.8, 11.6, 11.2 ppm. HRMS-ESI *(m/z)*: $[M+H]^+$ Calcd for $C_{41}H_{59}N_4O_5$ 687.4485; Found 687.4495. Optical rotation $[\alpha]^{20}_D$ –30.6 (*c* 0.79, $CHCl_3$).

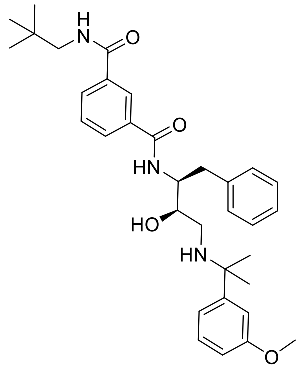

***N*^{1}-[(2*S*,3*R*)-3-Hydroxy-4-{[2-(3-methoxyphenyl)propan-2-yl]amino}-1-phenylbutan-2-yl]-*N*^{3}-neopentylisophthalamide ((*S*,*R*)-5a)**.

The title compound was obtained from (*R,S*)-**11** (30 mg, 0.075 mmol, 1.0 eq), benzoic acid **19a** (18 mg, 0.075 mmol, 1.0 eq), HBTU (28 mg, 0.075 mmol, 1.0 eq) and TEA (42 µL, 0.30 mmol, 4.0 eq) by following general procedure D. Purification by column chromatography on amino-functionalized silica gel using gradient elution from 25% EtOAc in petroleum ether to 60% EtOAc in petroleum ether afforded (*S*,*R*)-**5a** as a white solid (23 mg, 56% yield).

^{1}H-NMR (400 MHz, $CDCl_3$) δ: 8.05-8.01 (m, 1H, Ar-H), 7.90-7.85 (m, 1H, NH), 7.75-7.69 (m, 1H, NH), 7.47-7.39 (m, 1H, Ar-H), 7.29-7.26 (m, 1H, Ar-H), 7.24-7.15 (m, 6H, Ar-H), 7.02-6.95 (m, 2H, Ar-H), 6.77-6.71 (m, 1H, Ar-H), 6.38-6.29 (m, 1H, Ar-H), 4.46-4.35 (m, 1H, CH), 3.76 (s, 3H, OCH_3), 3.58-3.51 (m, 1H, CH), 3.27 (d, *J* = 6.5 Hz, 2H, NCH_2), 3.03-2.87 (m, 2H, CH_2), 2.55-2.41 (m, 2H, CH_2), 1.47 (s, 6H, CH_3×2), 0.98 (s, 9H, CH_3×3) ppm. ^{13}C-NMR (101 MHz, $CDCl_3$) δ: 166.9, 159.7, 148.8, 137.8, 135.5, 134.9, 130.2, 129.5, 129.4, 129.4, 129.0, 128.7, 126.7, 125.3, 118.5, 112.6, 111.4, 70.5, 55.9, 55.3, 54.4, 51.3, 44.4, 36.9, 32.4, 30.1, 29.0, 27.5 ppm. HRMS-ESI *(m/z)*: $[M+H]^+$ Calcd for $C_{33}H_{44}N_3O_4$ 546.3332; Found 546.3341. Optical rotation $[\alpha]^{20}_D$ –49.0 (*c* 1.73, $CHCl_3$).

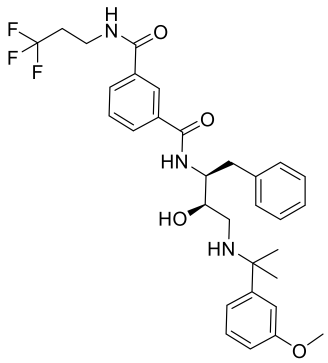

N^1-[(2*S*,3*R*)-3-Hydroxy-4-{[2-(3-methoxyphenyl)propan-2-yl]amino}-1-phenylbutan-2-yl]-N^3-(3,3,3-trifluoropropyl)isophthalamide ((*S*,*R*)-5b).

The title compound was obtained from (*R*,*S*)-**11** (30 mg, 0.075 mmol, 1.0 eq), benzoic acid **19b** (20 mg, 0.075 mmol, 1.0 eq), HBTU (28 mg, 0.075 mmol, 1.0 eq) and TEA (42 µL, 0.30 mmol, 4.0 eq) by following general procedure D. Purification by column chromatography on amino-functionalized silica gel using gradient elution from 25% EtOAc in petroleum ether to 60% EtOAc in petroleum ether afforded (*S*,*R*)-**5b** as a white solid (27 mg, 63% yield).

^{1}H-NMR (400 MHz, $CDCl_3$) δ: 8.01-7.97 (m, 1H, Ar-H), 7.90-7.86 (m, 1H, NH), 7.73-7.67 (m, 1H, NH), 7.45-7.39 (m, 1H, Ar-H), 7.33 (d, J = 8.6 Hz, 1H, Ar-H), 7.28-7.26 (m, 1H, Ar-H), 7.24-7.16 (m, 5H, Ar-H), 7.02-6.96 (m, 2H, Ar-H), 6.78-6.71 (m, 2H, Ar-H), 4.46-4.35 (m, 1H, CH), 3.77 (s, 3H, OCH_3), 3.74-3.66 (m, 2H, NCH_2), 3.59-3.53 (m, 1H, CH), 3.02-2.84 (m, 2H, CH_2), 2.59-2.40 (m, 4H, CH_2×2), 1.48 (s, 3H, CH_3), 1.47 (s, 3H, CH_3) ppm. ^{13}C-NMR (101 MHz, $CDCl_3$) δ: 166.9, 166.9, 159.7, 148.8, 137.8, 135.1, 134.5, 130.3, 129.8, 129.4, 129.1, 128.7, 127.9, 126.7, 125.4, 118.5, 112.6, 111.5, 70.4, 55.9, 55.4, 54.6, 44.4, 37.0, 33.9, 33.6, 30.2, 28.8 ppm. HRMS-ESI *(m/z)*: $[M+H]^+$ Calcd for $C_{31}H_{37}N_3O_4F_3$ 572.2736; Found 572.2737. Optical rotation $[\alpha]^{20}_D$ –45.2 (*c* 2.04, $CHCl_3$).

2. Molecular modeling

Docking was performed using the standard precision protocol in Glide. Docked complexes were further refined with Prime MM-GBSA (Molecular Mechanics/Generalized Born Surface Area) method using VSGB solvation model and OPLS3 force field. For MM-GBSA calculations, a 7-Å active region around the ligands for full molecular mechanics minimization was used. Results were visualized using UCSF Chimera v1.12 and Maestro.

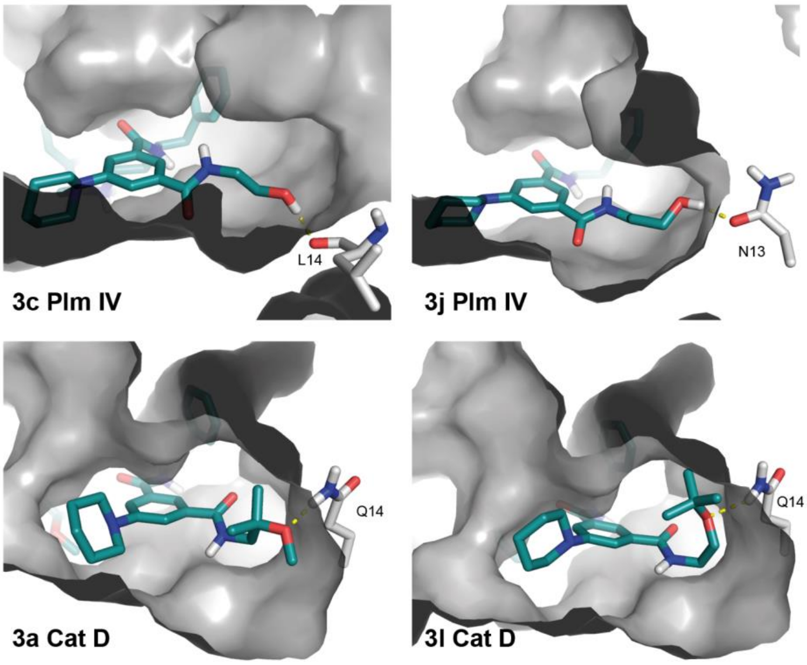

Fig. S1. Docking models of compounds containing hydrogen bond donor in S3 substituent (**3c,j** in complex with Plm IV); and compounds containing hydrogen bond acceptor group in S3 substituents (**3a,l** in complex with Cat D). Hydrogen bonds are indicated with yellow dashed line.

3. Biological assays

3.1. Enzymatic assay

A fluorescence resonance energy transfer (FRET) assay was performed to evaluate ability of compounds to inhibit PlmI, II, IV, and Cat D. K_m of the substrate was determined for each enzyme: PlmII = 2 ± 0.2 μM; PlmI = 2.7 ± 0.3μM; PlmIV = 2.8 ± 0.2μM; CatD = 1.8 ± 0.2 μM. A solution of compounds for testing (concentration 0.01−100 μM) on 96-well plate was added to the enzyme (PlmI,II,IV, or CatD) in buffer (0.1 M NaOAc, pH = 4.5, 10% glycerol). The mixture was incubated for 30 min at 37 °C. Substrate (DABCYL-Glu-Arg-Nle-Phe-Leu-Ser-Phe-Pro-EDANS, AnaSpec Inc.) was then added to reach a final concentration of 5 μM. Hydrolysis of the substrate was detected as an increase in fluorescence (Em 490 nm, Ex 336 nm) at 37 °C. The data points were collected every 1 min within 8−15 min. For the rate calculation, only the linear interval was used, which was slightly different for each enzyme. Compounds were tested in triplicate experiments. IC_{50} values were calculated using software Graph Pad Prism 5.0. Pepstatin A (IC_{50} = 0.42 ±0.02 nM (Plm II); IC_{50} = 0.9 ±0.02 nM (Plm I); IC_{50} = 0.3 ±0.04 nM (Plm IV)) and resveratrol (IC_{50} = 138 μM) were used as positive controls.

3.2. Red blood cell assay

The effects of compounds on growth of blood-stage *plasmodium falciparum* (clone 3D7) was assessed using a SYBR Green I assay, essentially as described previously.[1] Test compounds (dissolved in DMSO at concentrations ranging from 1 mM–0.1 μM) were added in triplicate to wells of flat bottomed, 96 well microtitre plates (1 μL per well). Wells were then supplemented with 100 μL per well of a synchronous *P. falciparum* parasite culture at 0.1% parasitaemia, 1% haematocrit. Each assay plate also included DMSO only control wells, as well as additional control wells containing uninfected erythrocytes only. Plates were incubated in sealed humidified gassed chambers at 37 °C for 96 h to allow the parasites to undergo two entire cycles of erythrocytic growth. Wells were then supplemented with 100 μL of a 1:5,000 dilution of stock SYBR Green I (Life Technologies, catalogue #S7563) diluted in 20 mM Tris-HCl pH 7.5, 5 mM EDTA, 0.008 % (w/v) saponin, 0.08% (v/v) Triton X100. Plates were agitated to mix, incubated for a further 1 h in the dark at room temperature, then transferred to a Cary Eclipse fluorescence spectrophotometer (Varian) equipped with a 96-well microplate reader accessory for fluorescence readings (Ex 485 nm, Em 530 nm). EC_{50} values were determined from dose-response curves obtained after subtracting background fluorescence values (obtained from the erythrocyte only wells) from all experimental readings.

Spectral Data

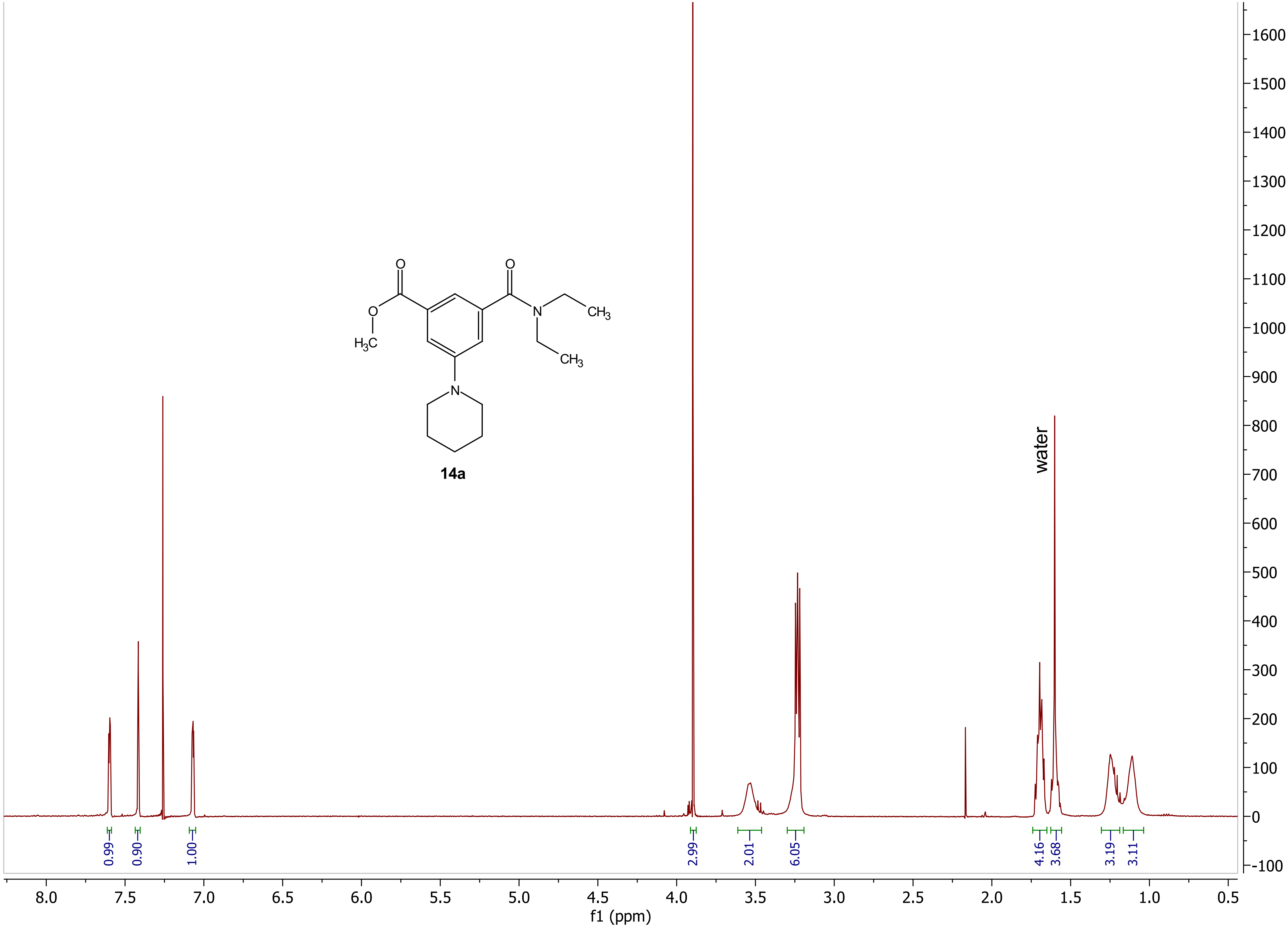

O
O
H3C
O
N
CH3
CH3
N
14a
water
0.99
0.90
1.00
2.99
2.01
6.05
4.16
3.68
3.19
3.11
8.0
7.5
7.0
6.5
6.0
5.5
5.0
4.5
4.0
3.5
3.0
2.5
2.0
1.5
1.0
0.5
f1 (ppm)
1600
1500
1400
1300
1200
1100
1000
900
800
700
600
500
400
300
200
100
0
-100

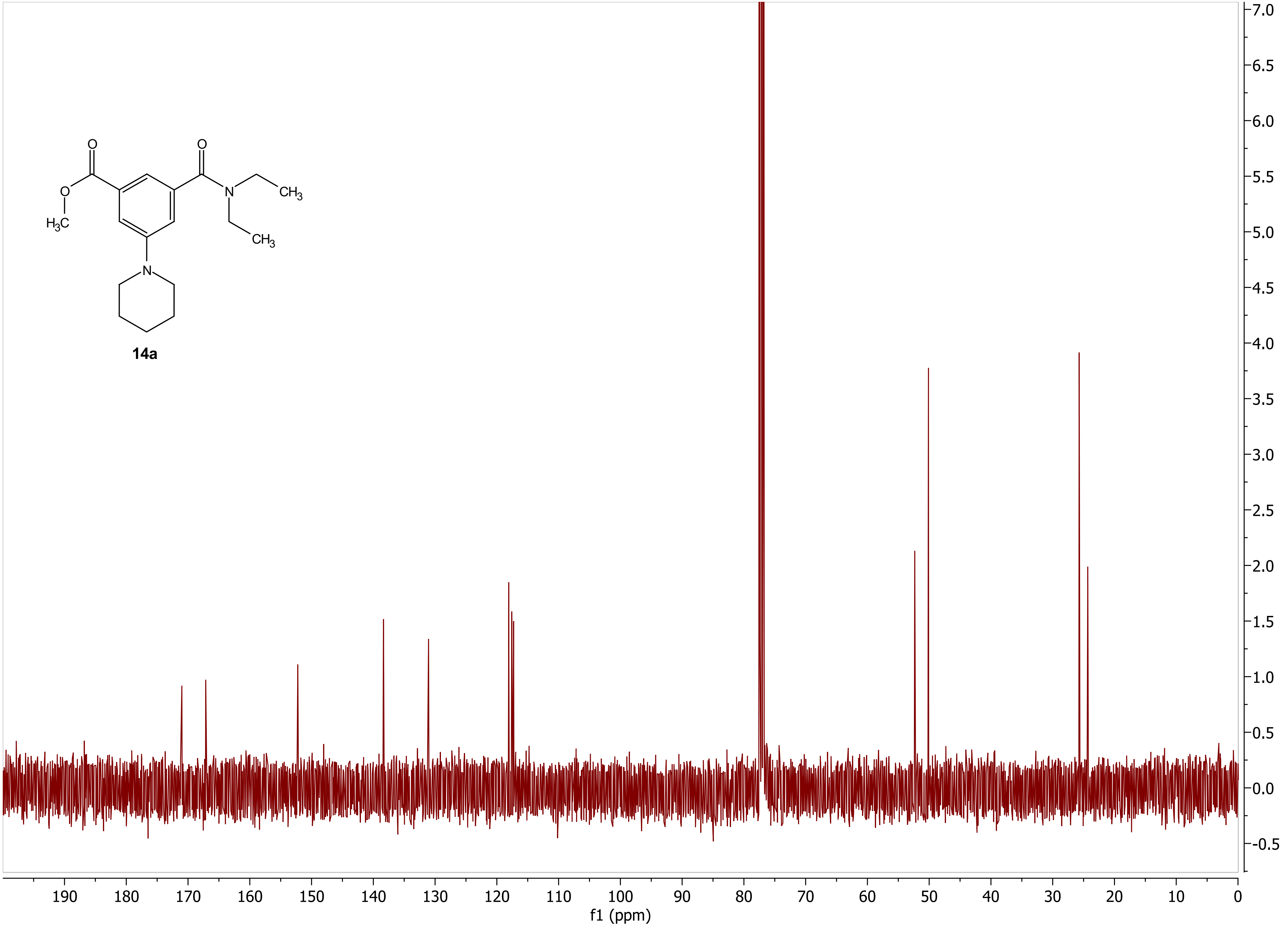

O
O
O
N
CH3
H3C
CH3
N
14a
7.0
6.5
6.0
5.5
5.0
4.5
4.0
3.5
3.0
2.5
2.0
1.5
1.0
0.5
0.0
-0.5
190
180
170
160
150
140
130
120
110
100
90
80
70
60
50
40
30
20
10
0
f1 (ppm)

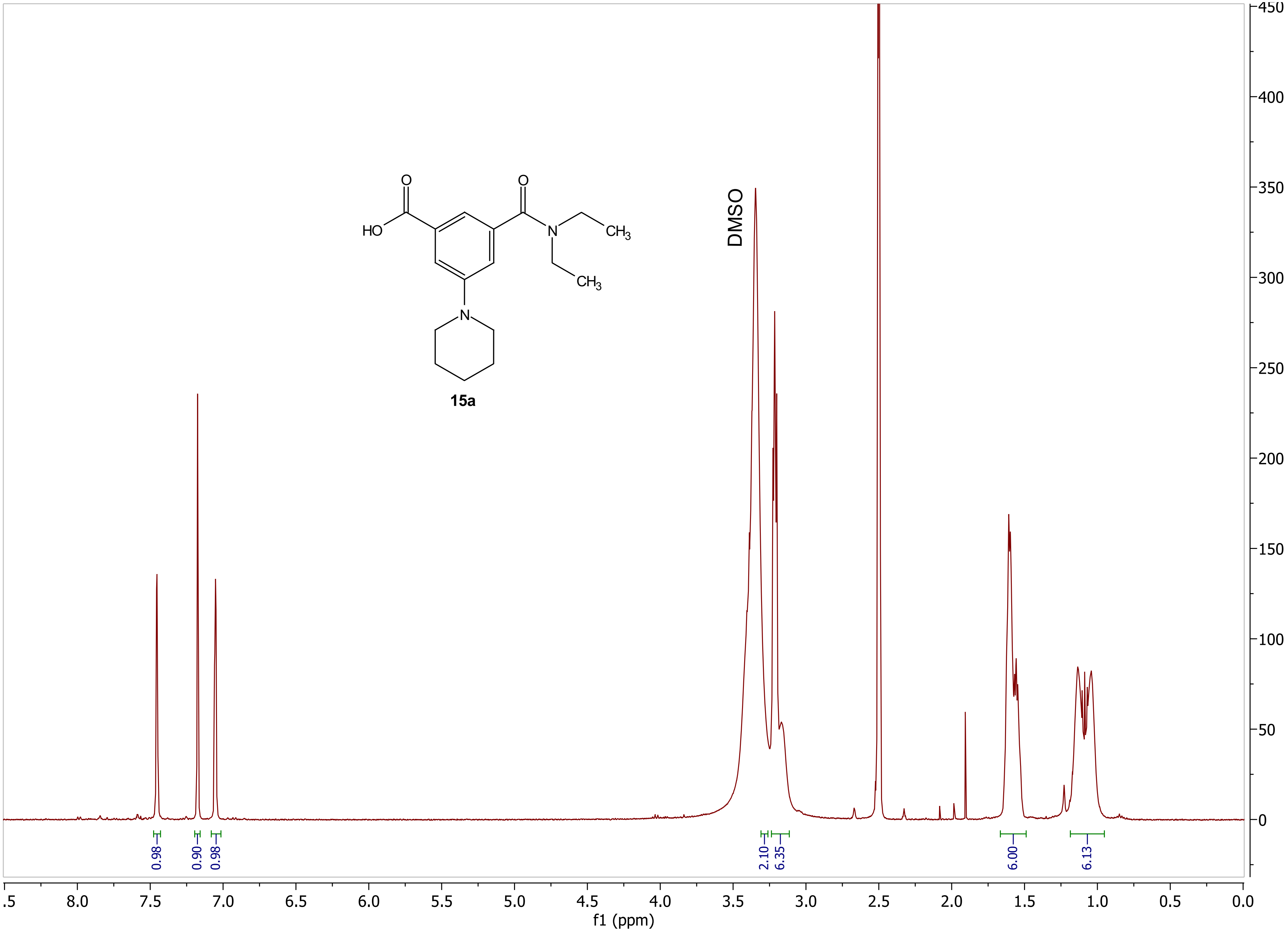

HO
O
O
N
CH3
CH3
N
15a
DMSO
0.98
0.90
0.98
2.10
6.35
6.00
6.13
f1 (ppm)

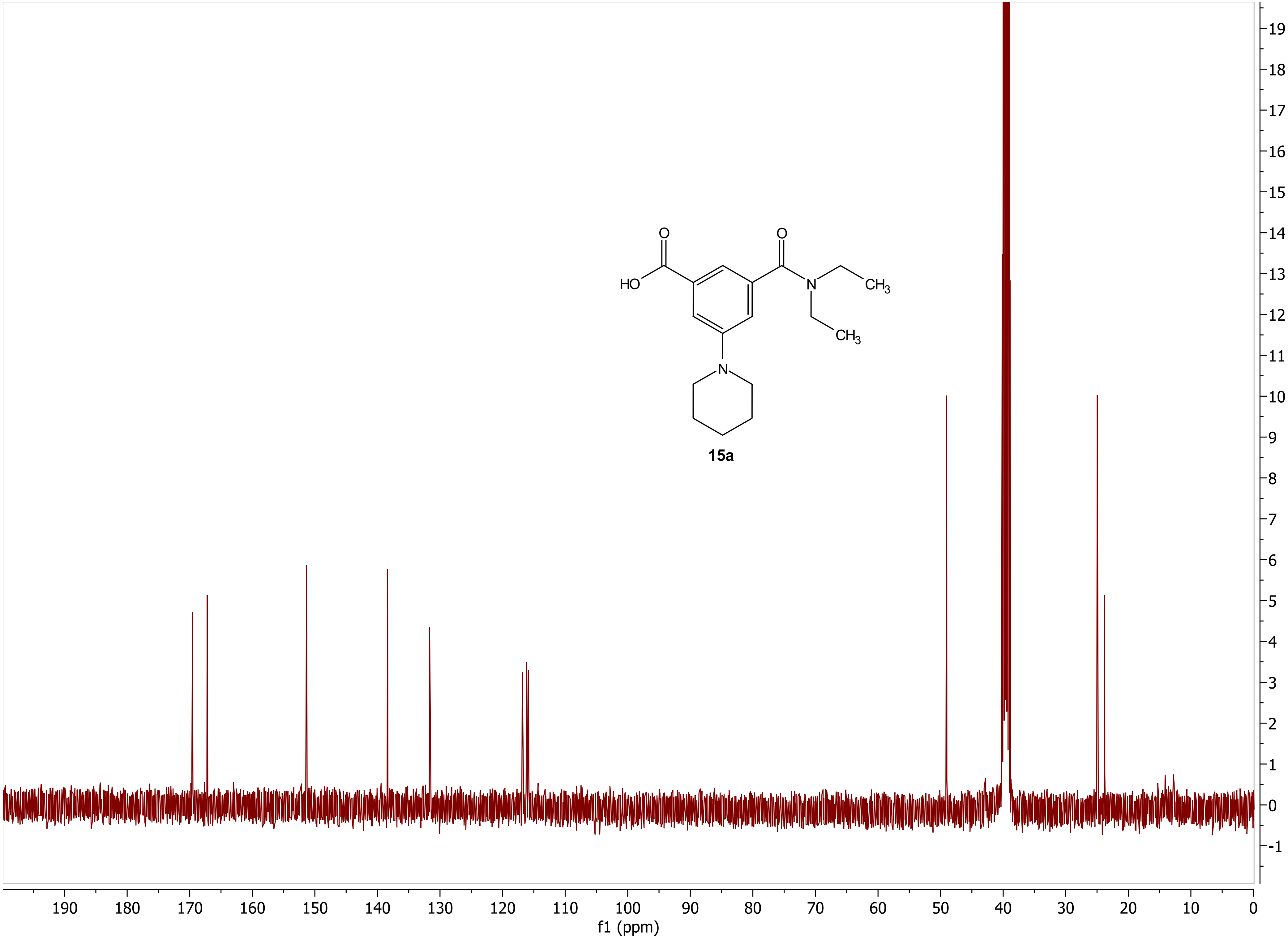
O
HO
O
N
CH3
CH3
N
15a
190 180 170 160 150 140 130 120 110 100 90 80 70 60 50 40 30 20 10 0
f1 (ppm)
19 18 17 16 15 14 13 12 11 10 9 8 7 6 5 4 3 2 1 0 -1

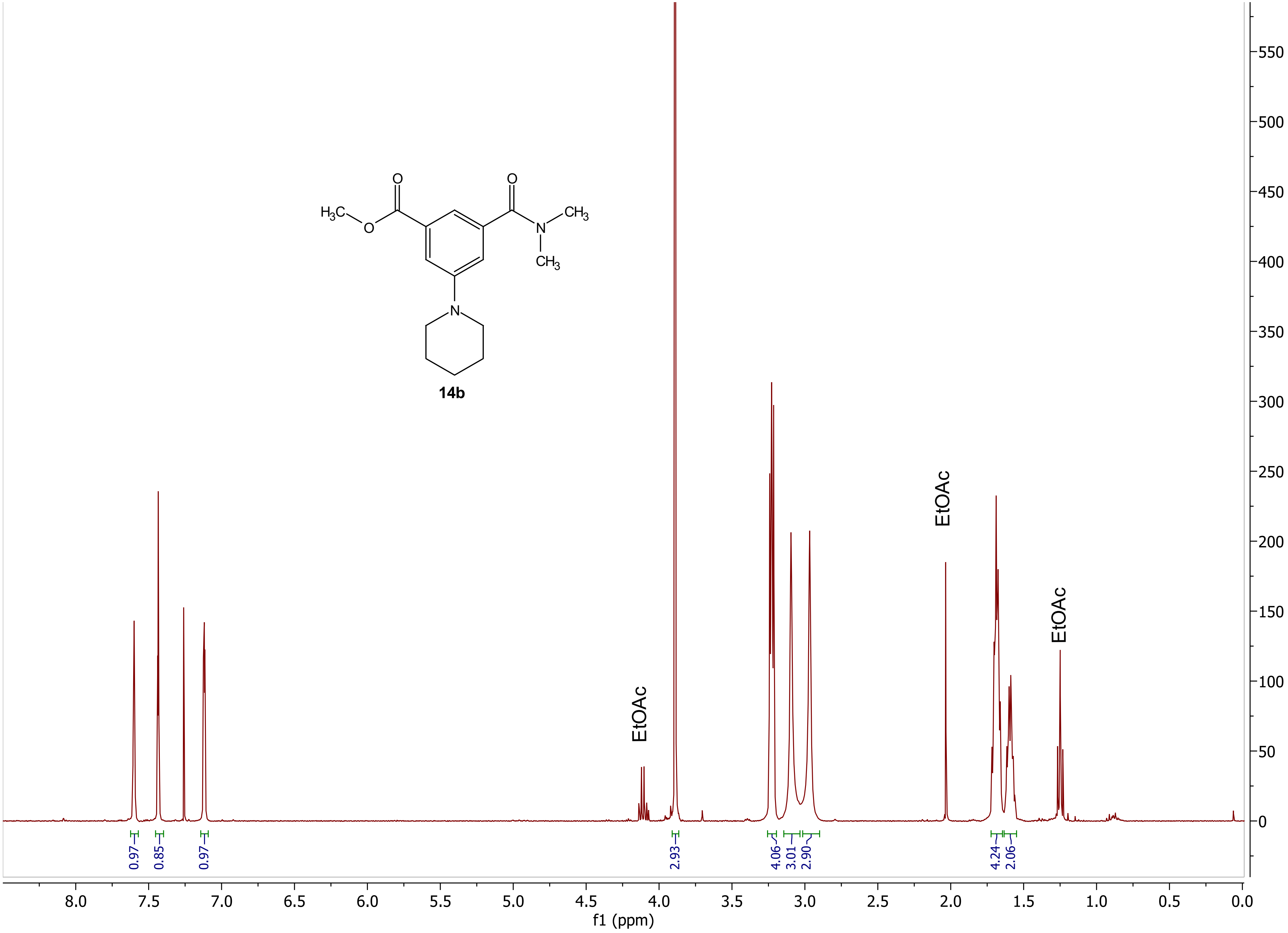

O
O
H_3C
O
N
CH_3
CH_3
N
14b
EtOAc
EtOAc
EtOAc
0.97
0.85
0.97
2.93
4.06
3.01
2.90
4.24
2.06
8.0 7.5 7.0 6.5 6.0 5.5 5.0 4.5 4.0 3.5 3.0 2.5 2.0 1.5 1.0 0.5 0.0
f1 (ppm)
550 500 450 400 350 300 250 200 150 100 50 0

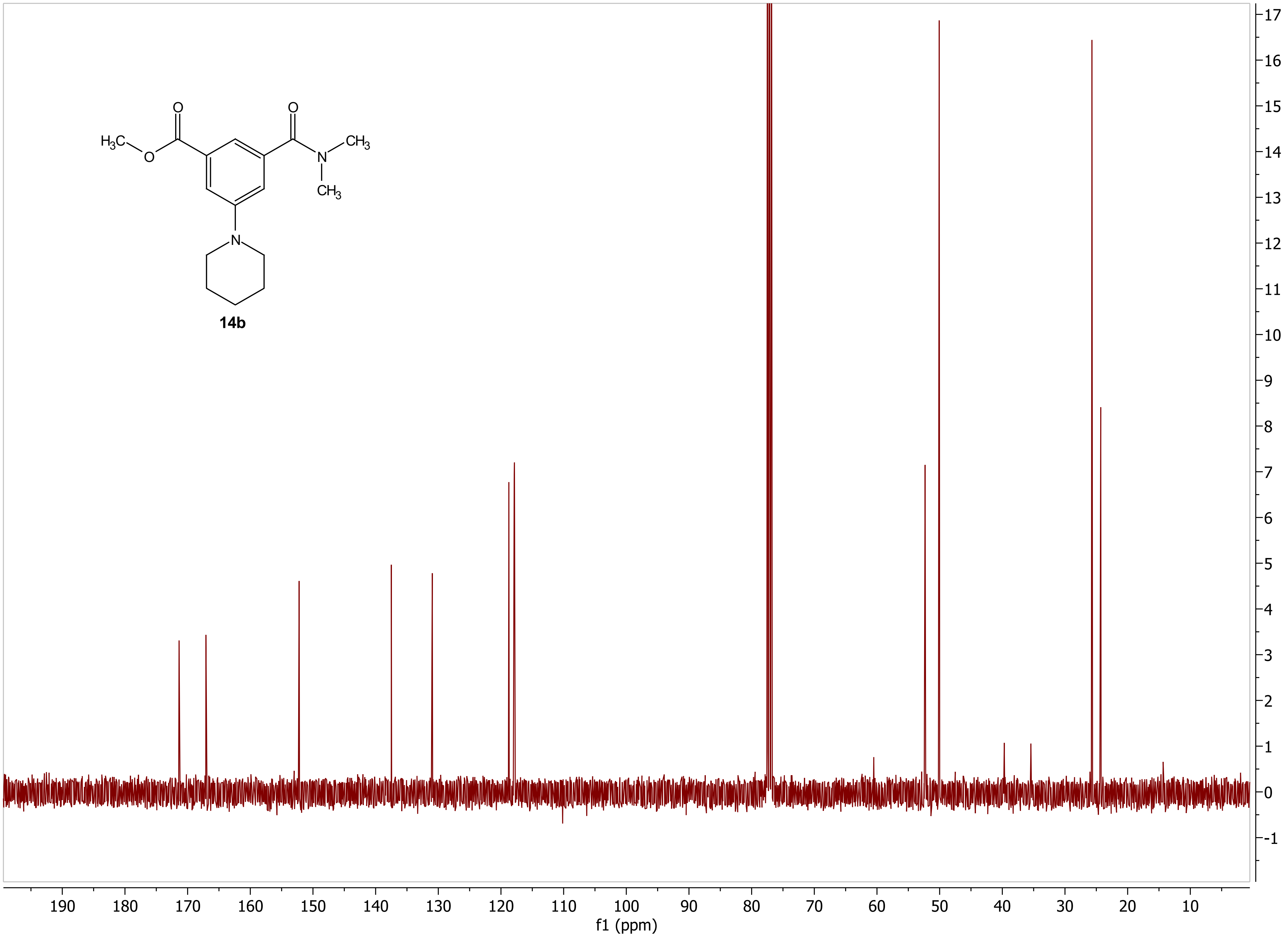
H3C
O
O
O
N
CH3
CH3
N
14b
f1 (ppm)

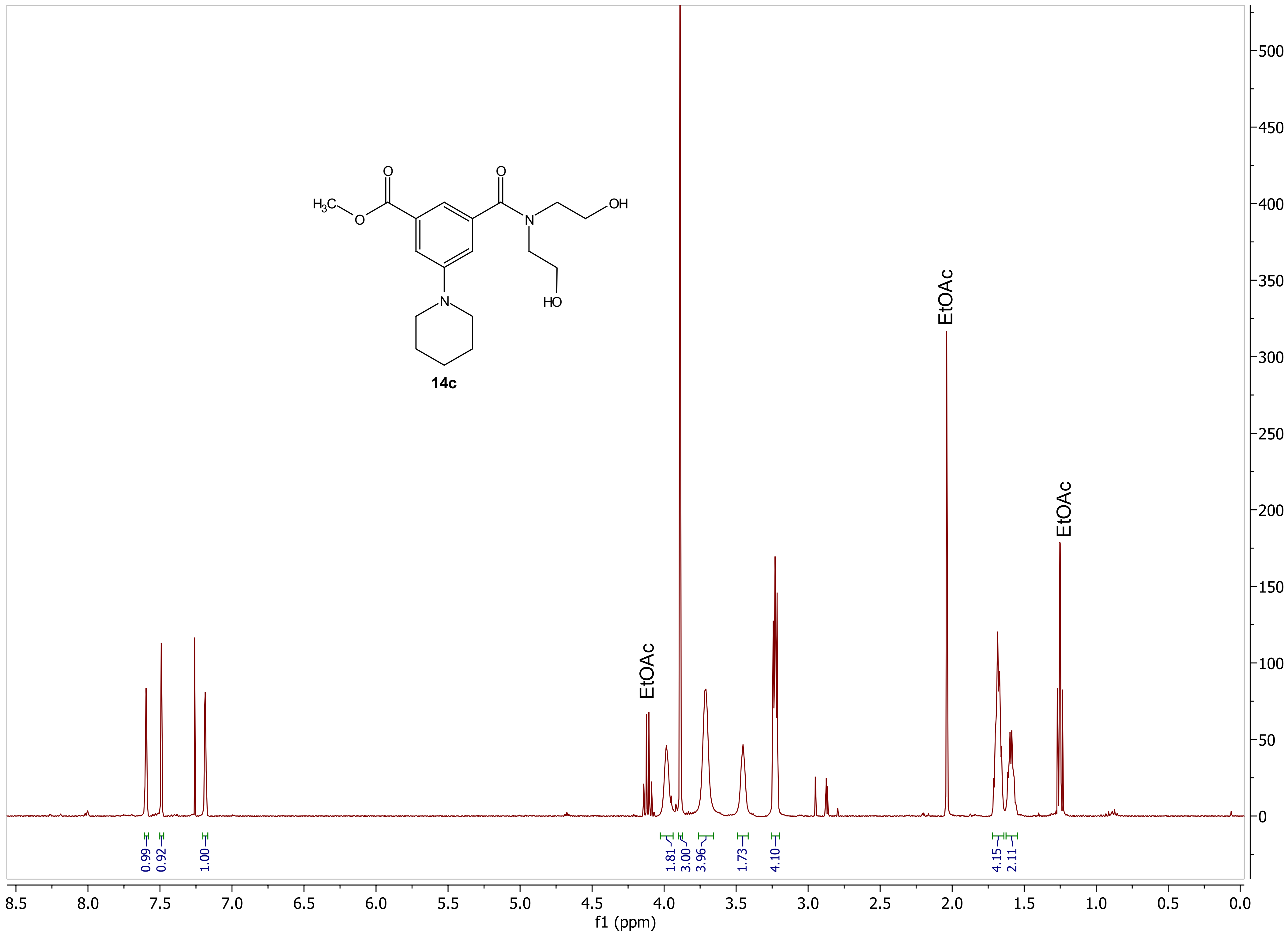

H3C
O
O
O
N
OH
HO
N
14c
EtOAc
EtOAc
EtOAc
0.99
0.92
1.00
1.81
3.00
3.96
1.73
4.10
4.15
2.11
f1 (ppm)

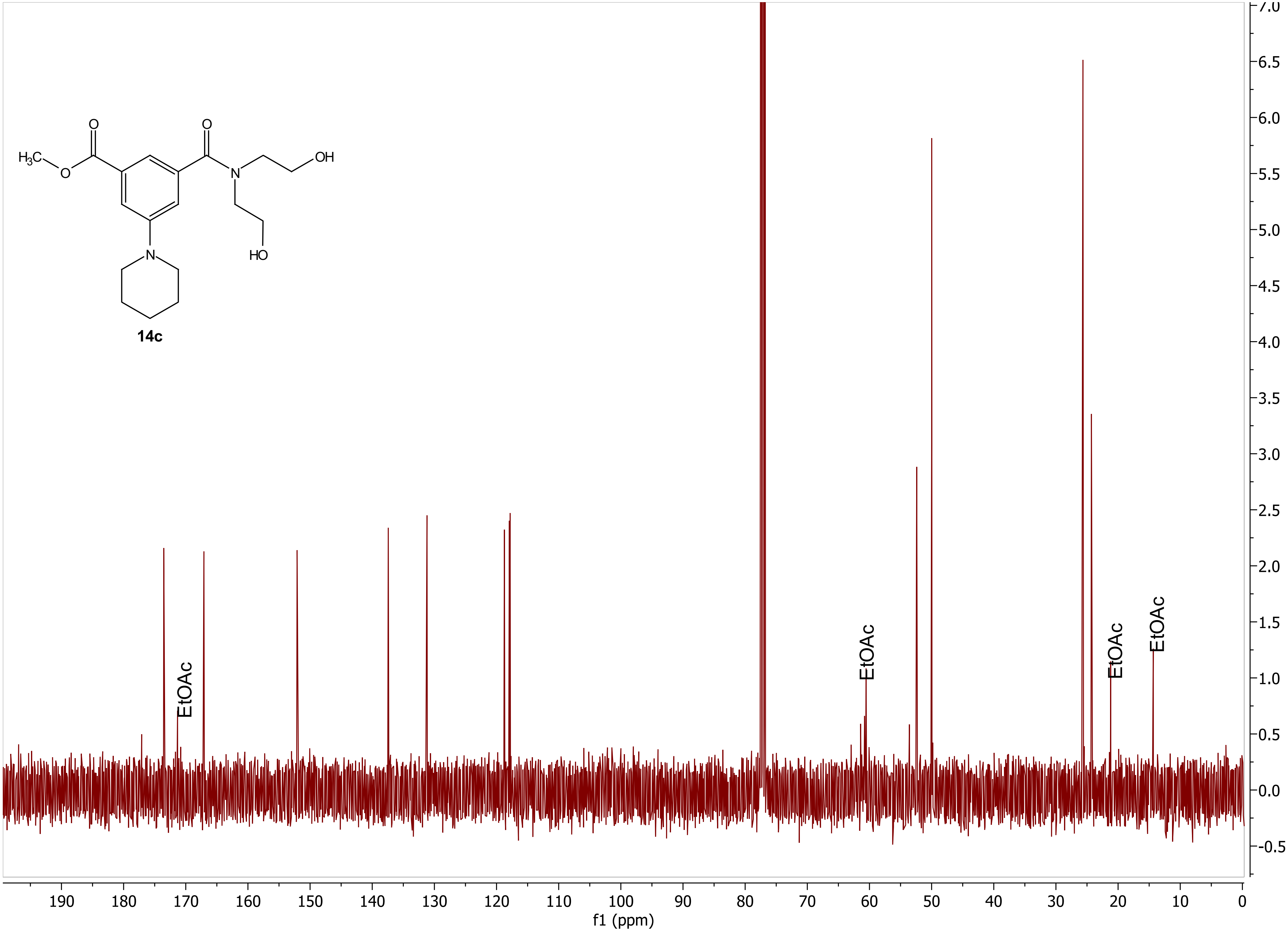

H3C
O
O
O
N
OH
HO
N
14c
EtOAc
EtOAc
EtOAc
EtOAc
190 180 170 160 150 140 130 120 110 100 90 80 70 60 50 40 30 20 10 0
f1 (ppm)
7.0 6.5 6.0 5.5 5.0 4.5 4.0 3.5 3.0 2.5 2.0 1.5 1.0 0.5 0.0 -0.5

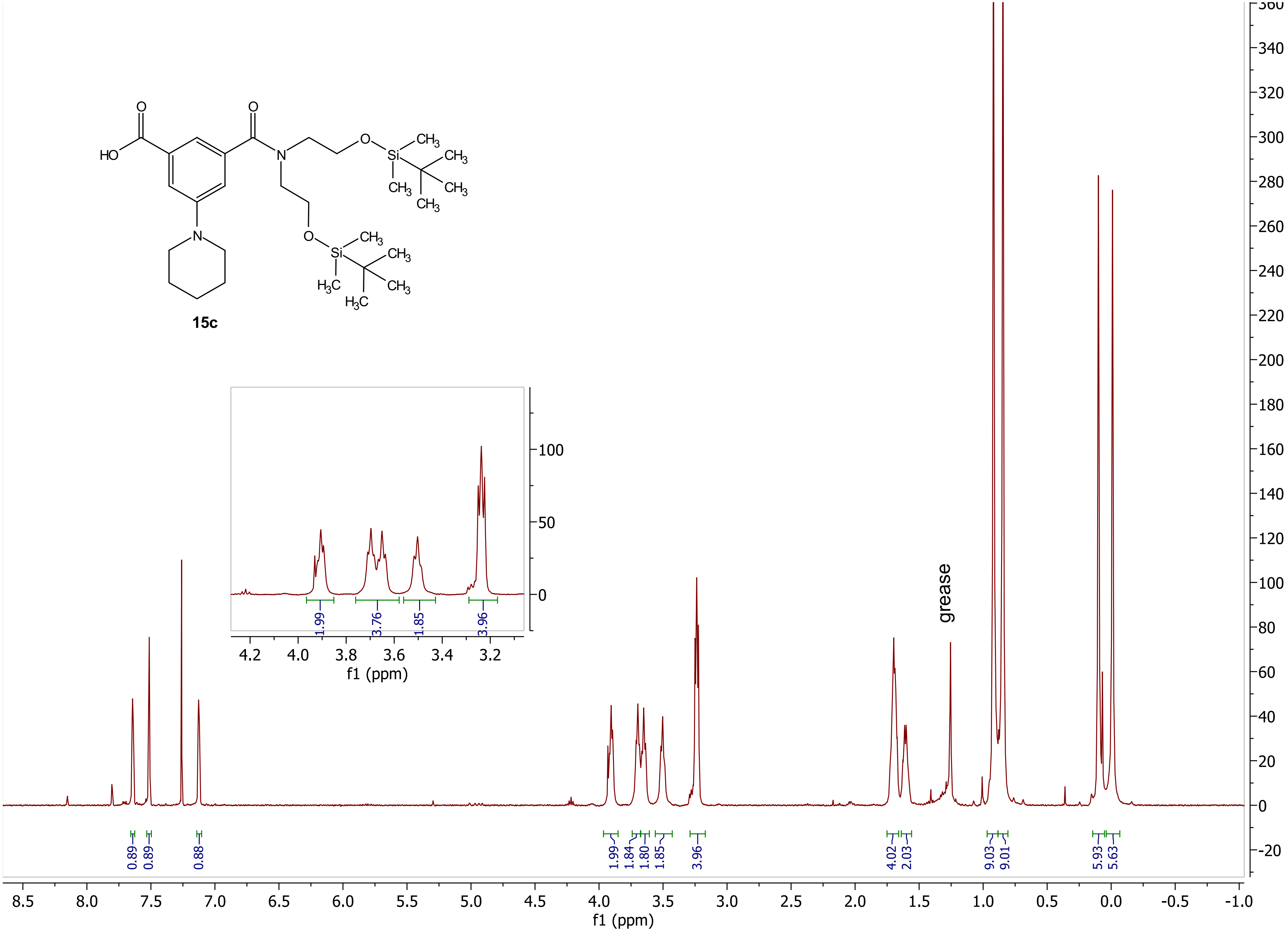
15c
grease
f1 (ppm)

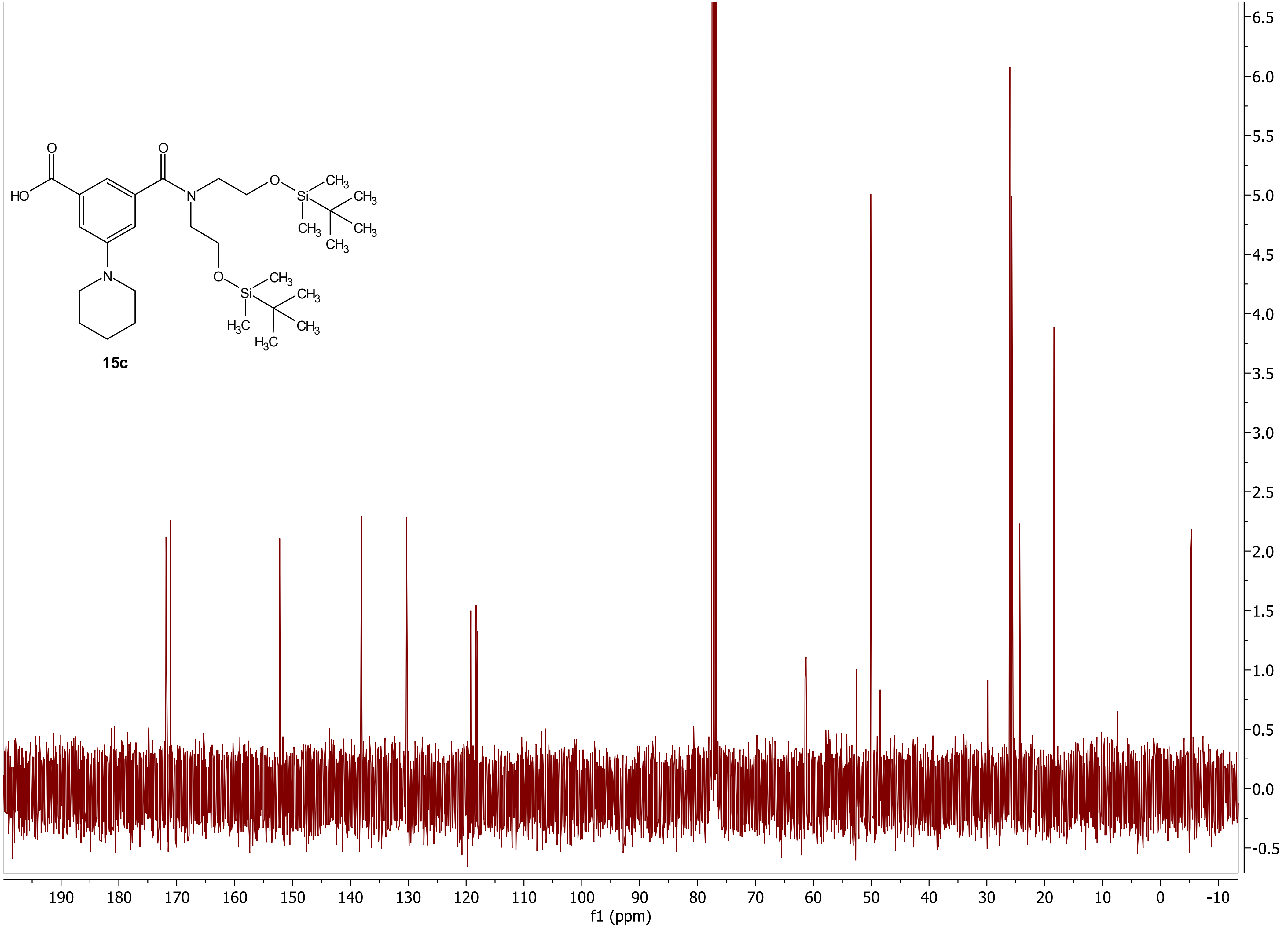
15c
f1 (ppm)
190 180 170 160 150 140 130 120 110 100 90 80 70 60 50 40 30 20 10 0 -10
6.5 6.0 5.5 5.0 4.5 4.0 3.5 3.0 2.5 2.0 1.5 1.0 0.5 0.0 -0.5

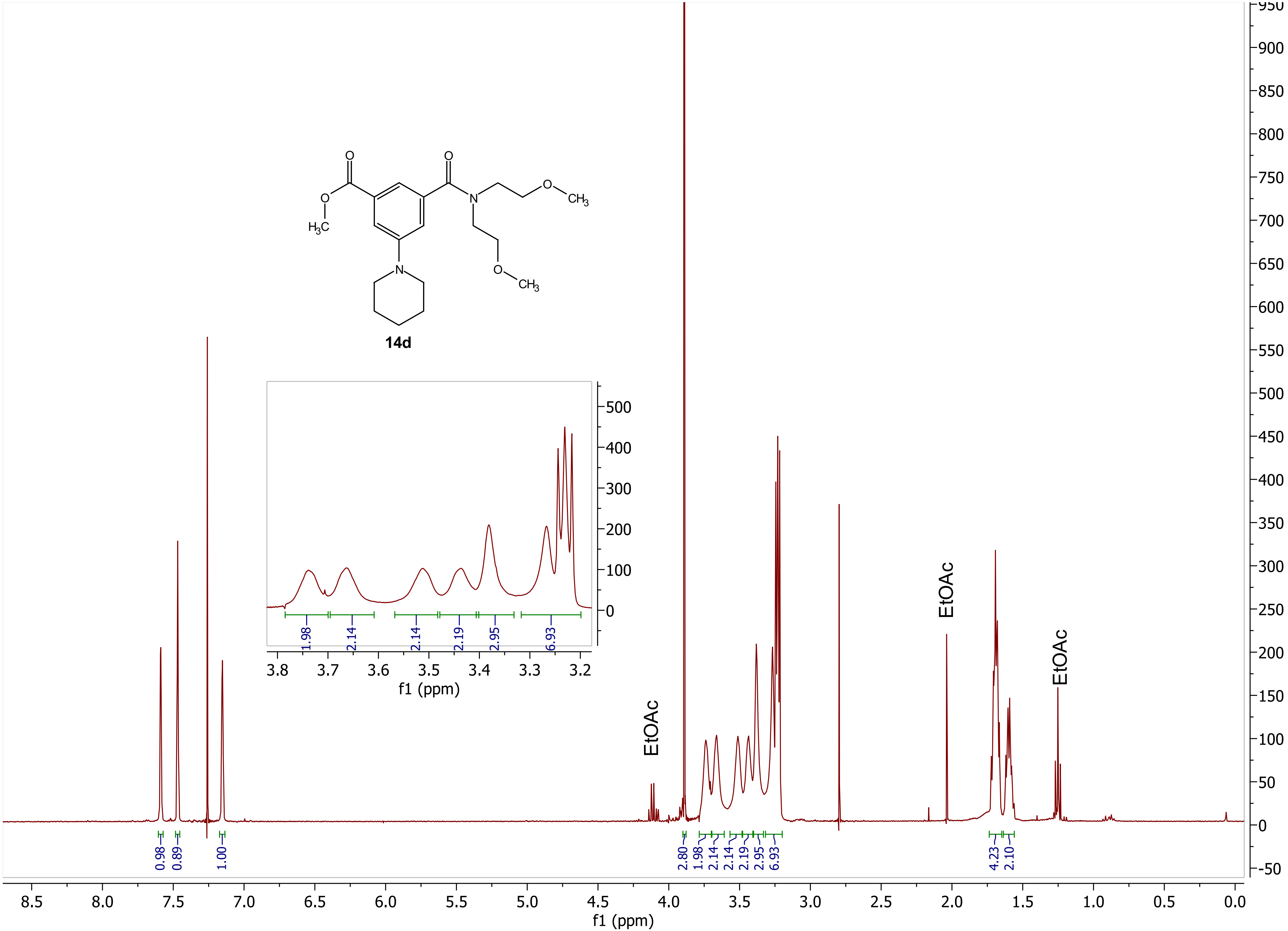

EtOAc
EtOAc
EtOAc
0.98
0.89
1.00
2.80
1.98
2.14
2.14
2.19
2.95
6.93
4.23
2.10
f1 (ppm)

14d

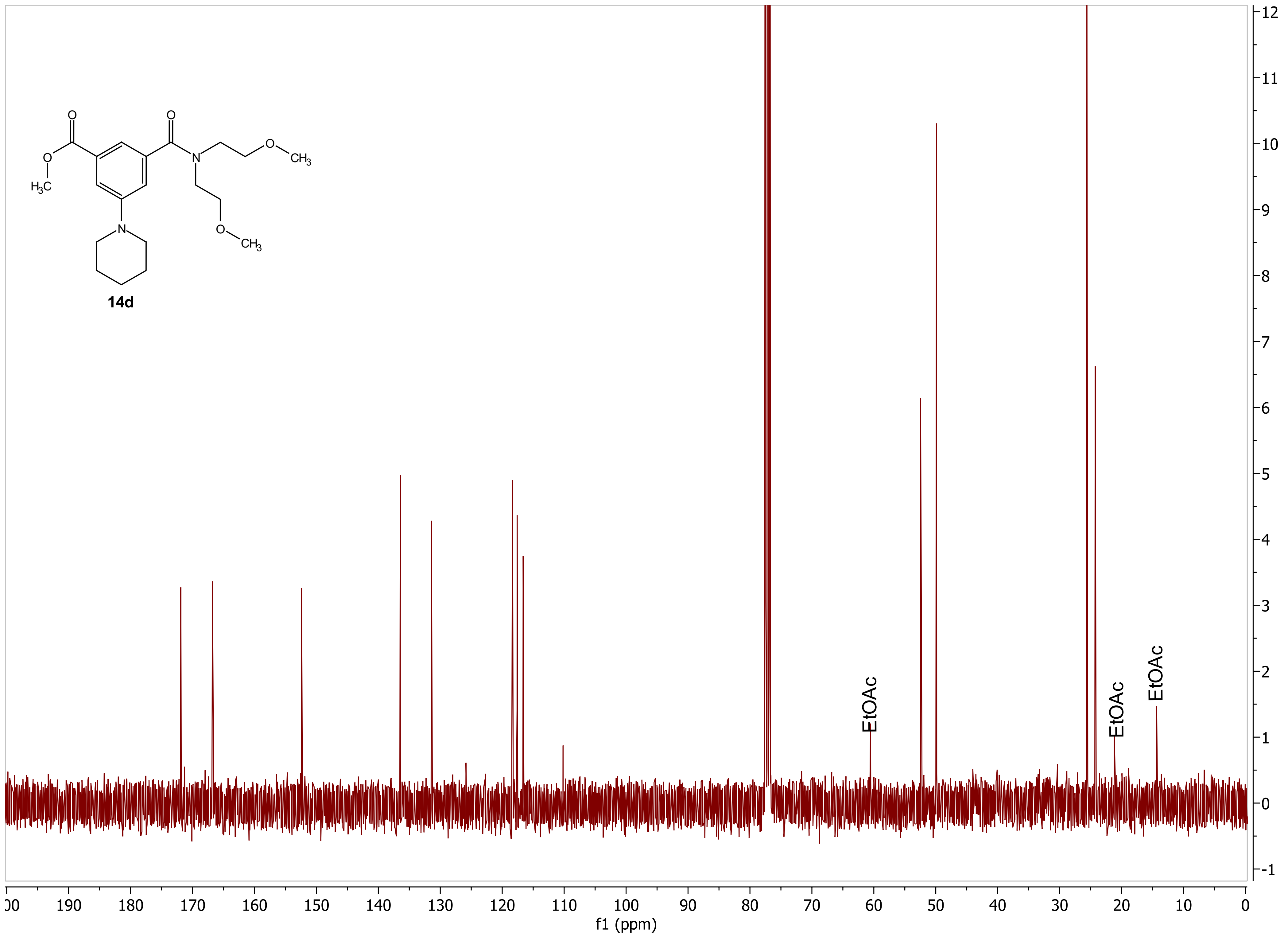

14d
EtOAc
EtOAc
EtOAc
f1 (ppm)

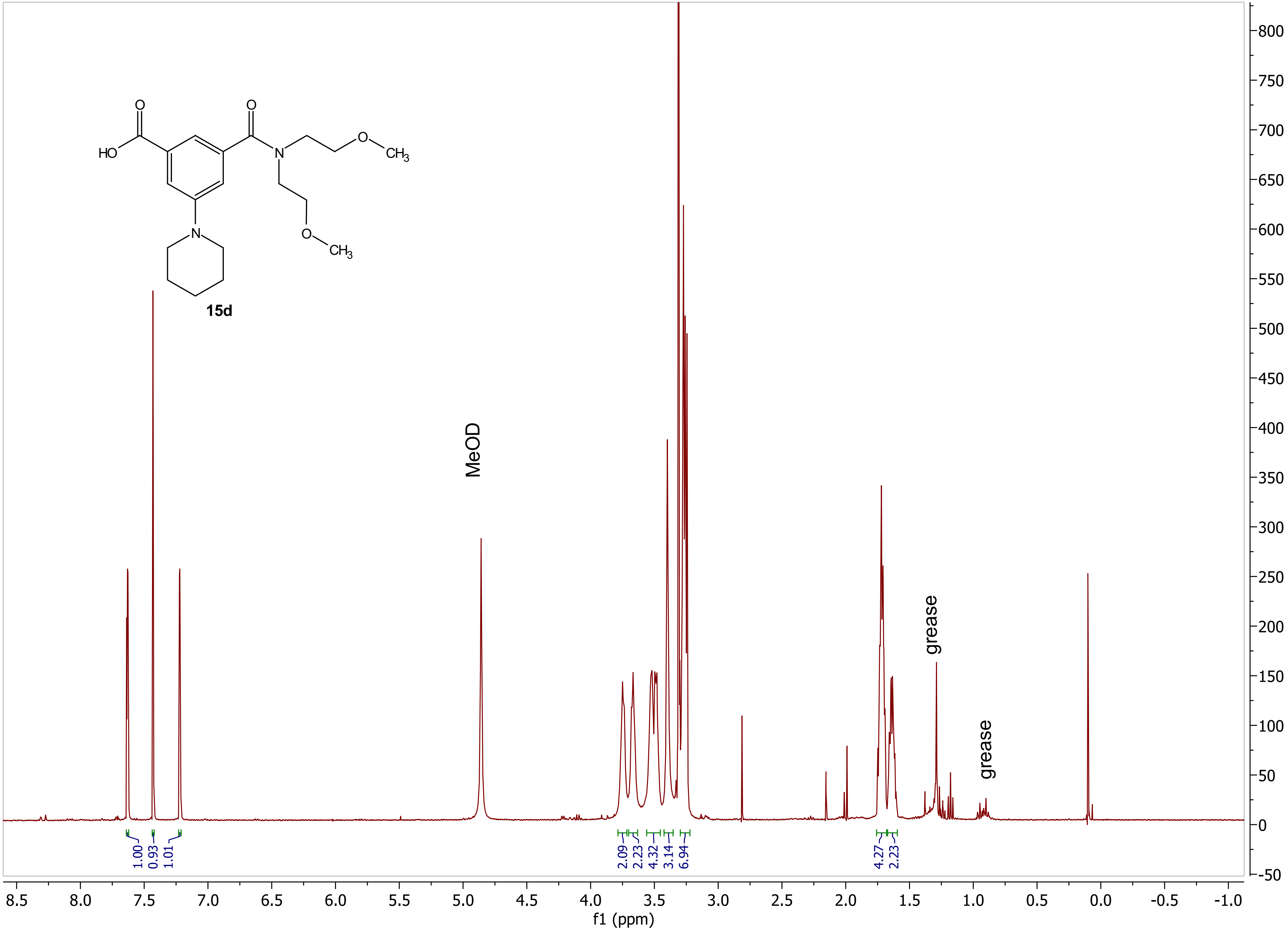
HO
O
O
N
O
CH3
N
O
CH3
15d
MeOD
grease
grease
1.00
0.93
1.01
2.09
2.23
4.32
3.14
6.94
4.27
2.23
f1 (ppm)

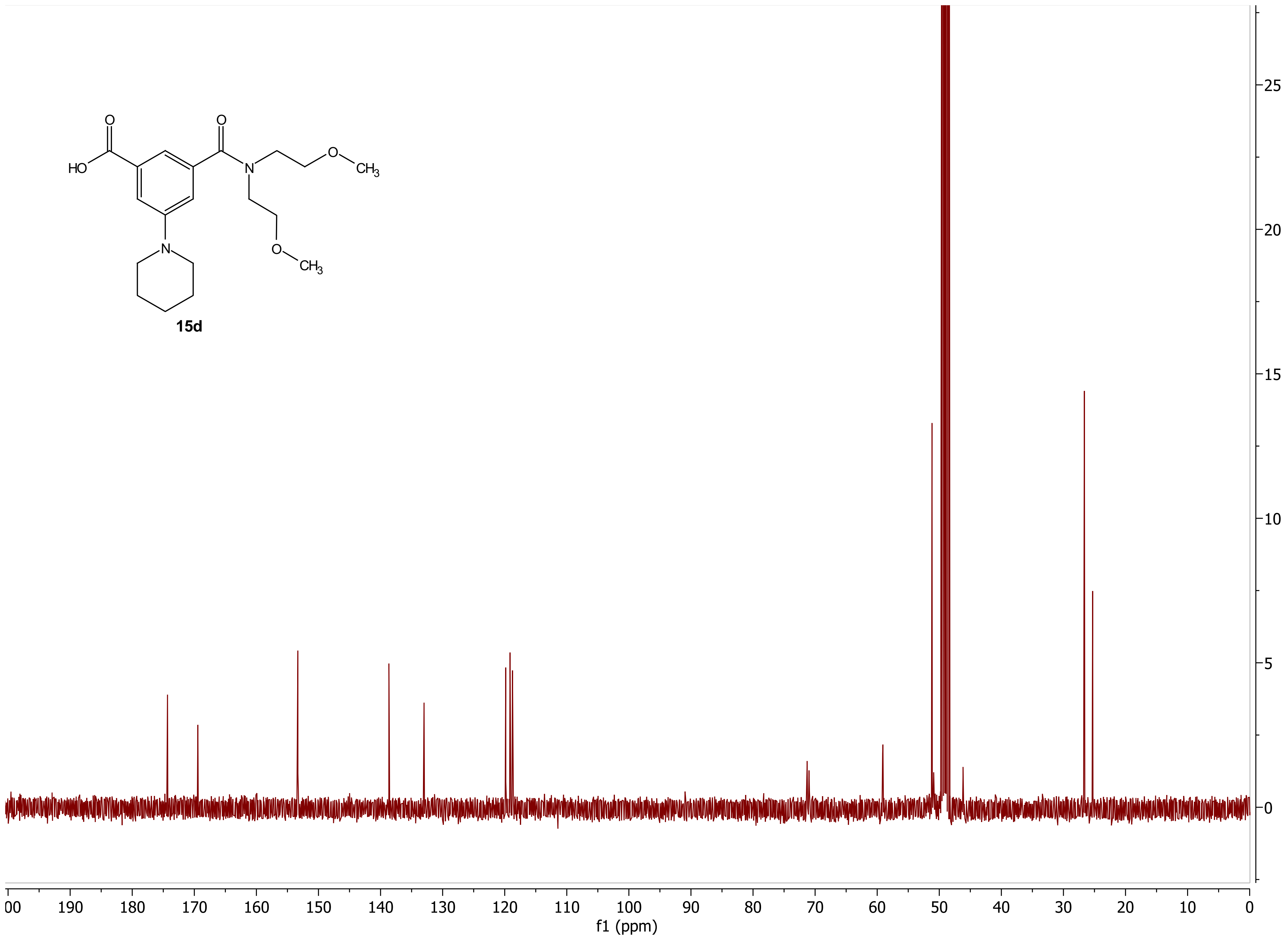
HO
O
N
CH3
15d
f1 (ppm)
00
190
180
170
160
150
140
130
120
110
100
90
80
70
60
50
40
30
20
10
0
25
20
15
10
5

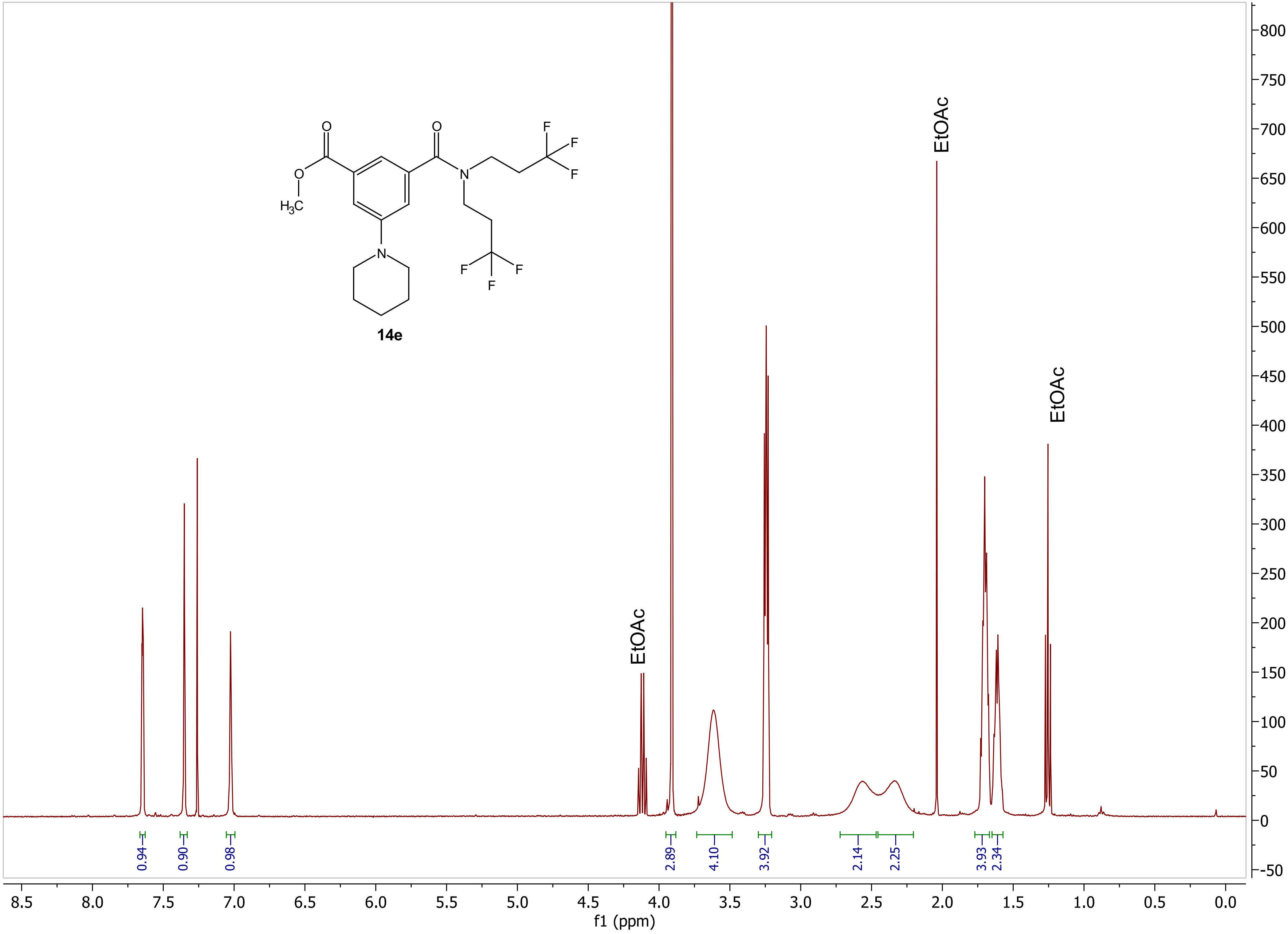
14e
EtOAc
EtOAc
EtOAc
0.94
0.90
0.98
2.89
4.10
3.92
2.14
2.25
3.93
2.34
8.5 8.0 7.5 7.0 6.5 6.0 5.5 5.0 4.5 4.0 3.5 3.0 2.5 2.0 1.5 1.0 0.5 0.0
f1 (ppm)
800 750 700 650 600 550 500 450 400 350 300 250 200 150 100 50 0 -50

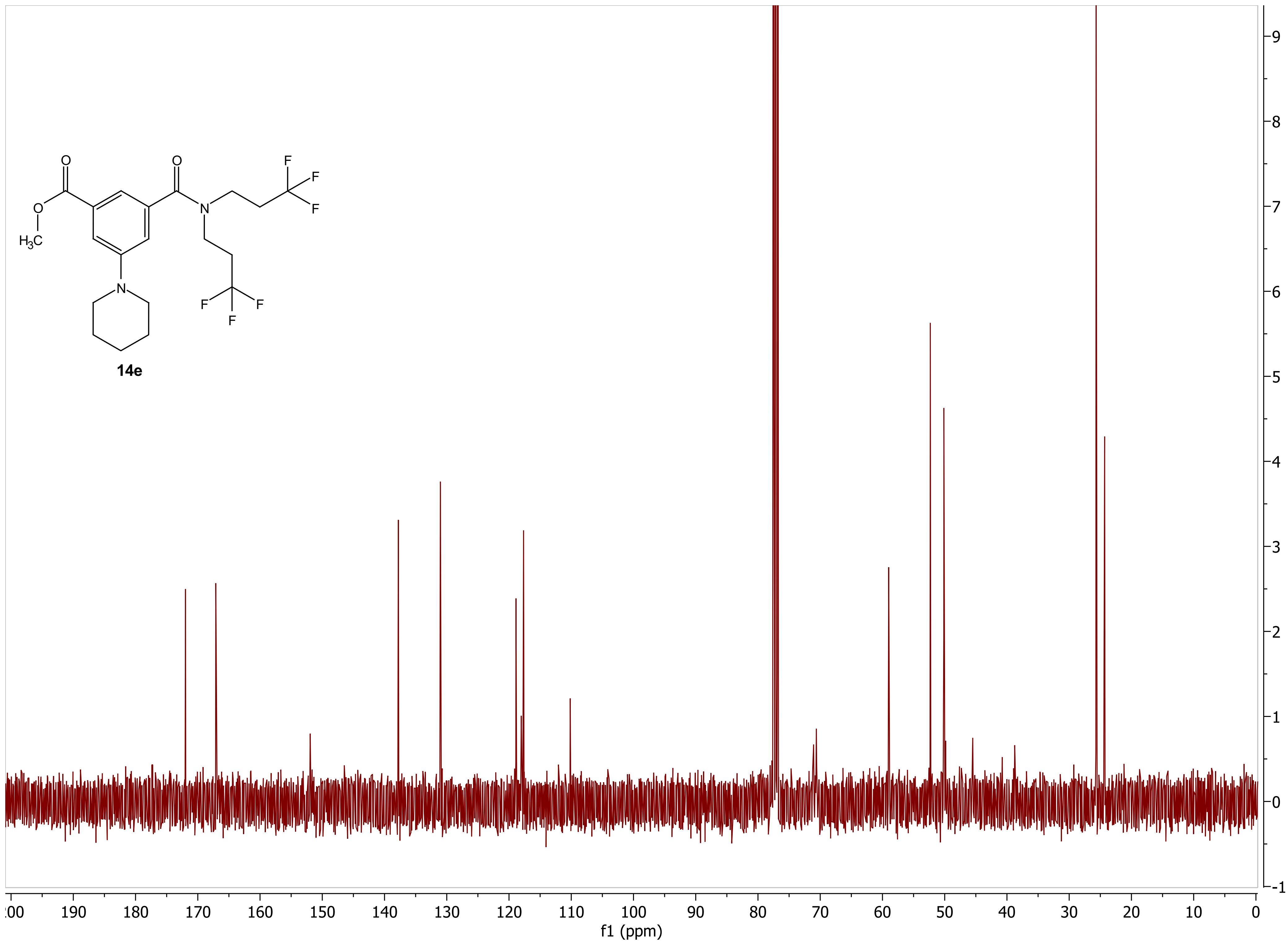
O
O
O
H3C
N
F
F
F
F
F
F
N
14e
200 190 180 170 160 150 140 130 120 110 100 90 80 70 60 50 40 30 20 10 0
f1 (ppm)
9 8 7 6 5 4 3 2 1 0 -1

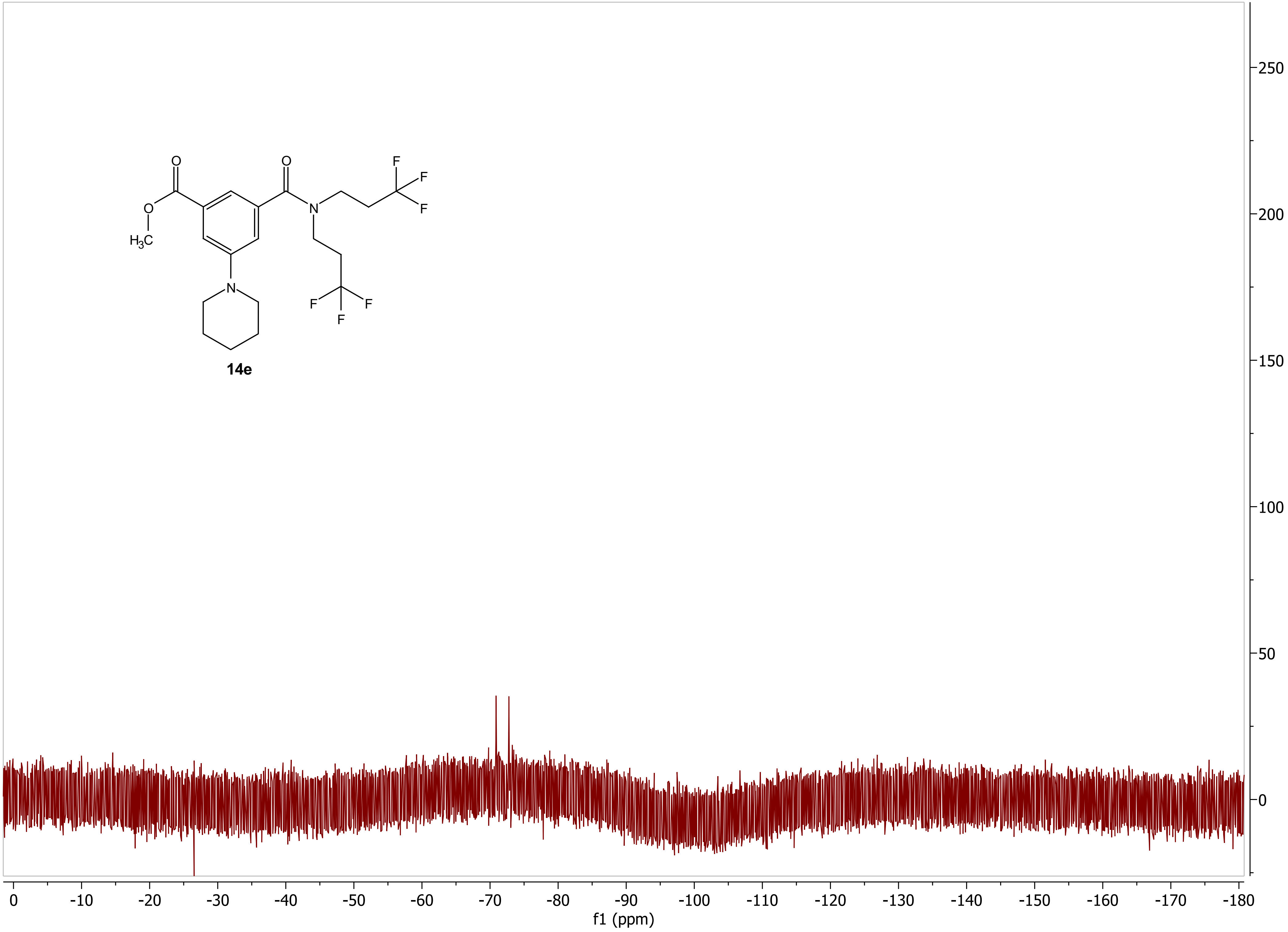
O
O
O
N
F
F
F
H3C
N
F
F
F
14e
250
200
150
100
50
0
0 -10 -20 -30 -40 -50 -60 -70 -80 -90 -100 -110 -120 -130 -140 -150 -160 -170 -180
f1 (ppm)

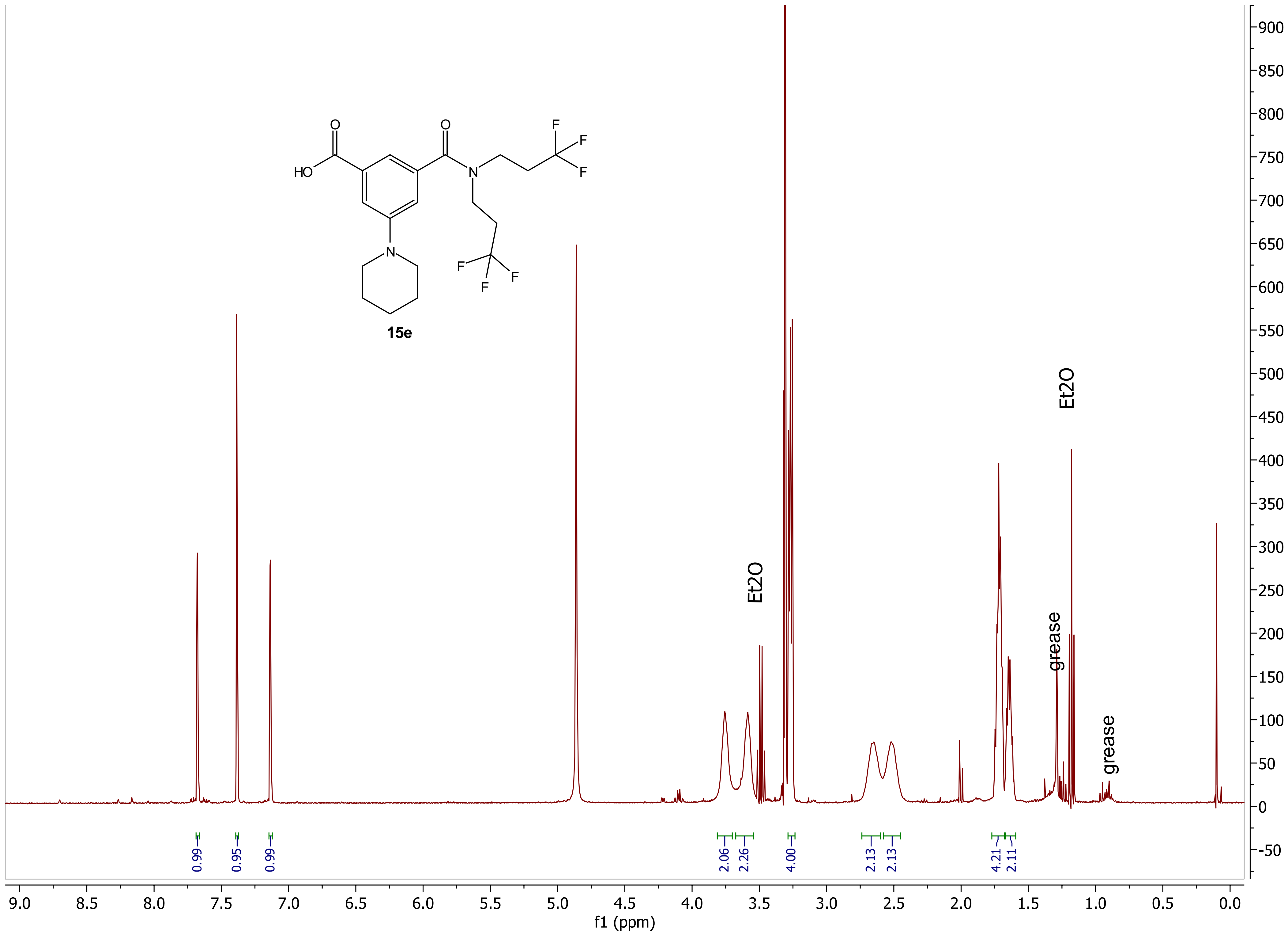
HO
O
O
N
F
F
F
N
F
F
F
15e
Et2O
Et2O
grease
grease
0.99
0.95
0.99
2.06
2.26
4.00
2.13
2.13
4.21
2.11
f1 (ppm)

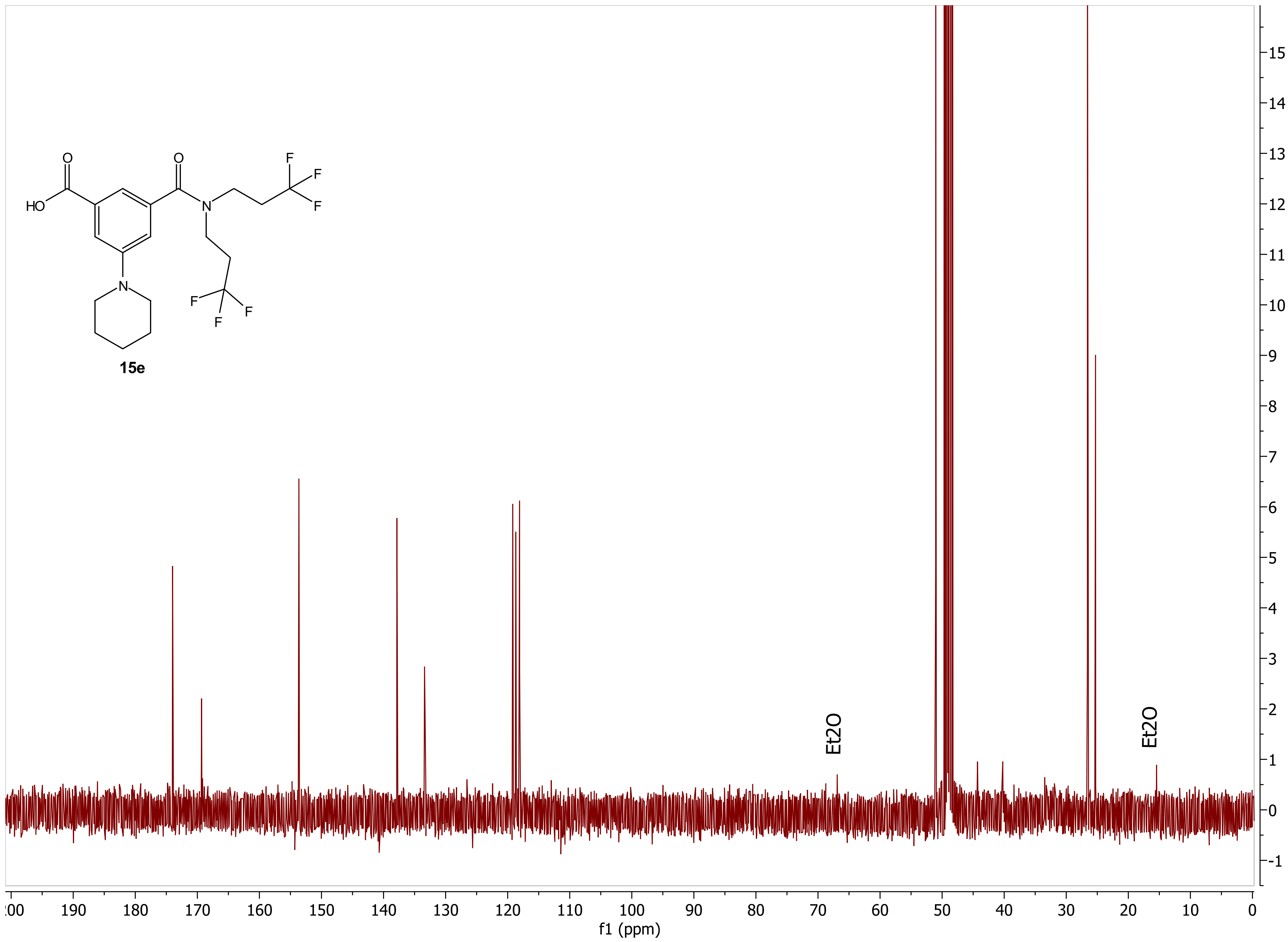

HO
O
O
N
F
F
F
N
F
F
F
15e
Et2O
Et2O
f1 (ppm)

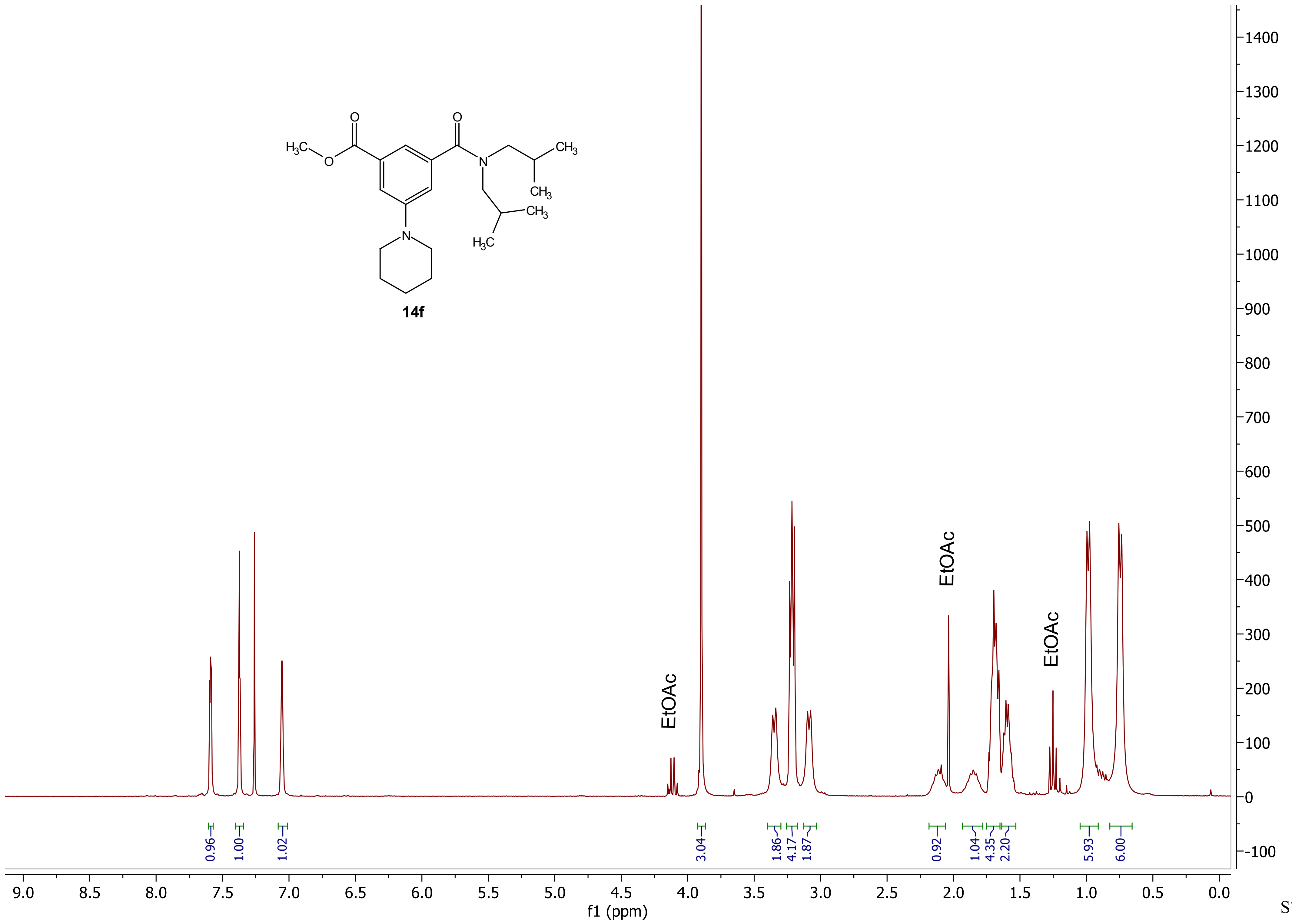

H3C
O
O
O
N
CH3
CH3
CH3
H3C
N
14f
EtOAc
EtOAc
EtOAc
0.96
1.00
1.02
3.04
1.86
4.17
1.87
0.92
1.04
4.35
2.20
5.93
6.00
9.0
8.5
8.0
7.5
7.0
6.5
6.0
5.5
5.0
4.5
4.0
3.5
3.0
2.5
2.0
1.5
1.0
0.5
0.0
f1 (ppm)
1400
1300
1200
1100
1000
900
800
700
600
500
400
300
200
100
0
-100

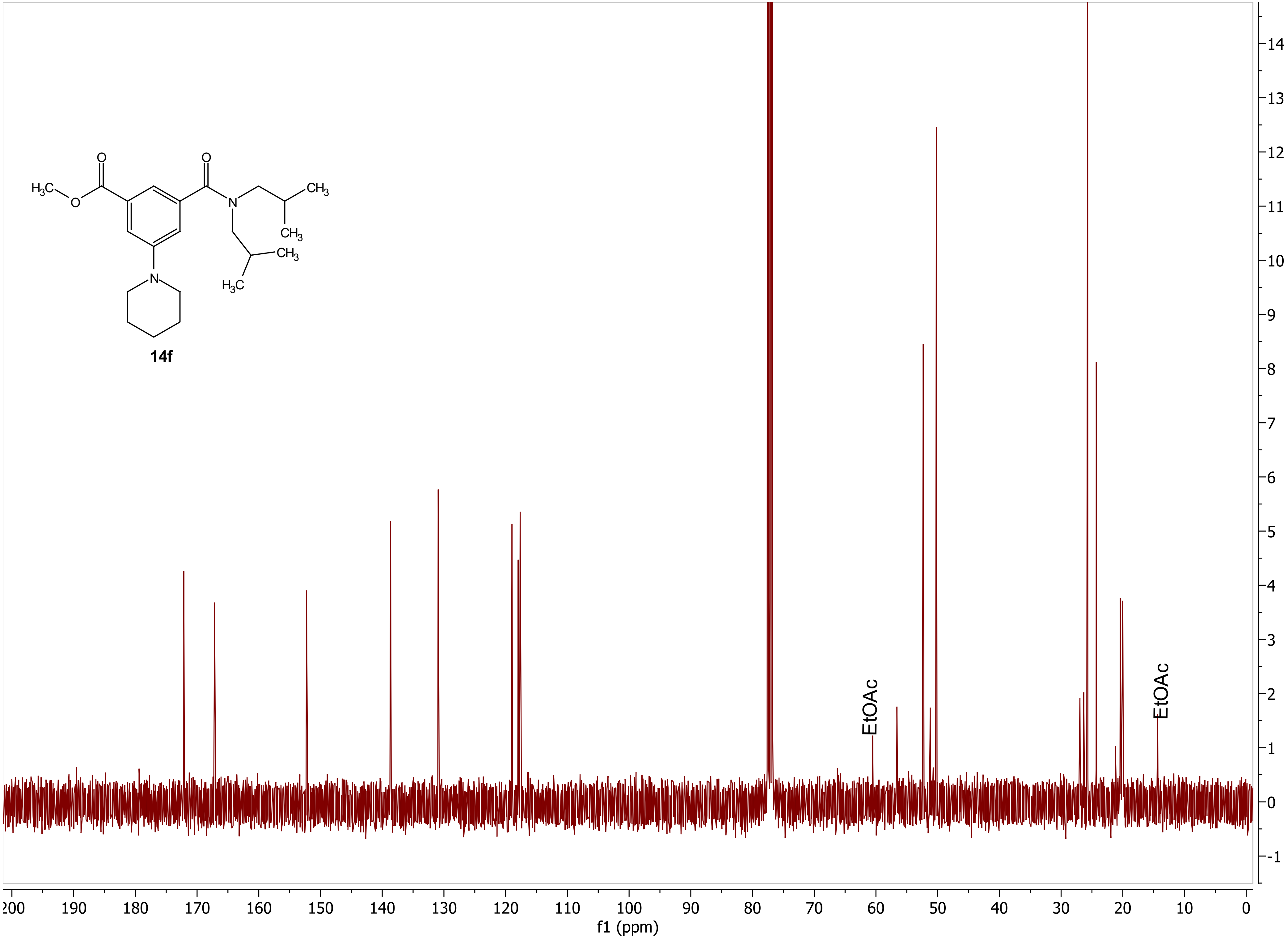
O
O
H_3C
O
N
CH_3
CH_3
CH_3
N
H_3C
14f
EtOAc
EtOAc
200 190 180 170 160 150 140 130 120 110 100 90 80 70 60 50 40 30 20 10 0
f1 (ppm)
14 13 12 11 10 9 8 7 6 5 4 3 2 1 0 -1

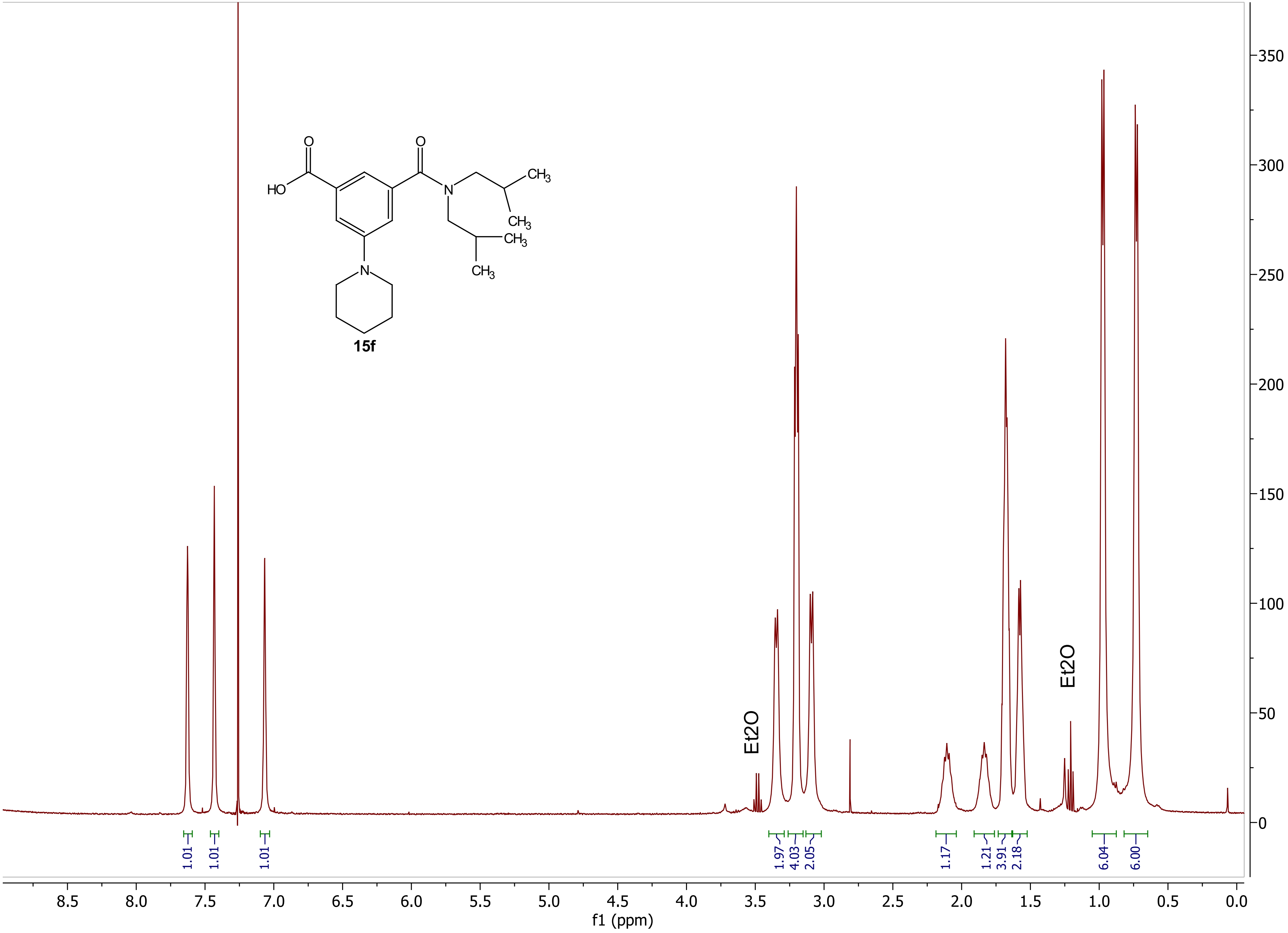

HO
O
O
N
CH3
CH3
CH3
CH3
N
15f
Et2O
Et2O
1.01
1.01
1.01
1.97
4.03
2.05
1.17
1.21
3.91
2.18
6.04
6.00
8.5
8.0
7.5
7.0
6.5
6.0
5.5
5.0
4.5
4.0
3.5
3.0
2.5
2.0
1.5
1.0
0.5
0.0
f1 (ppm)
350
300
250
200
150
100
50
0

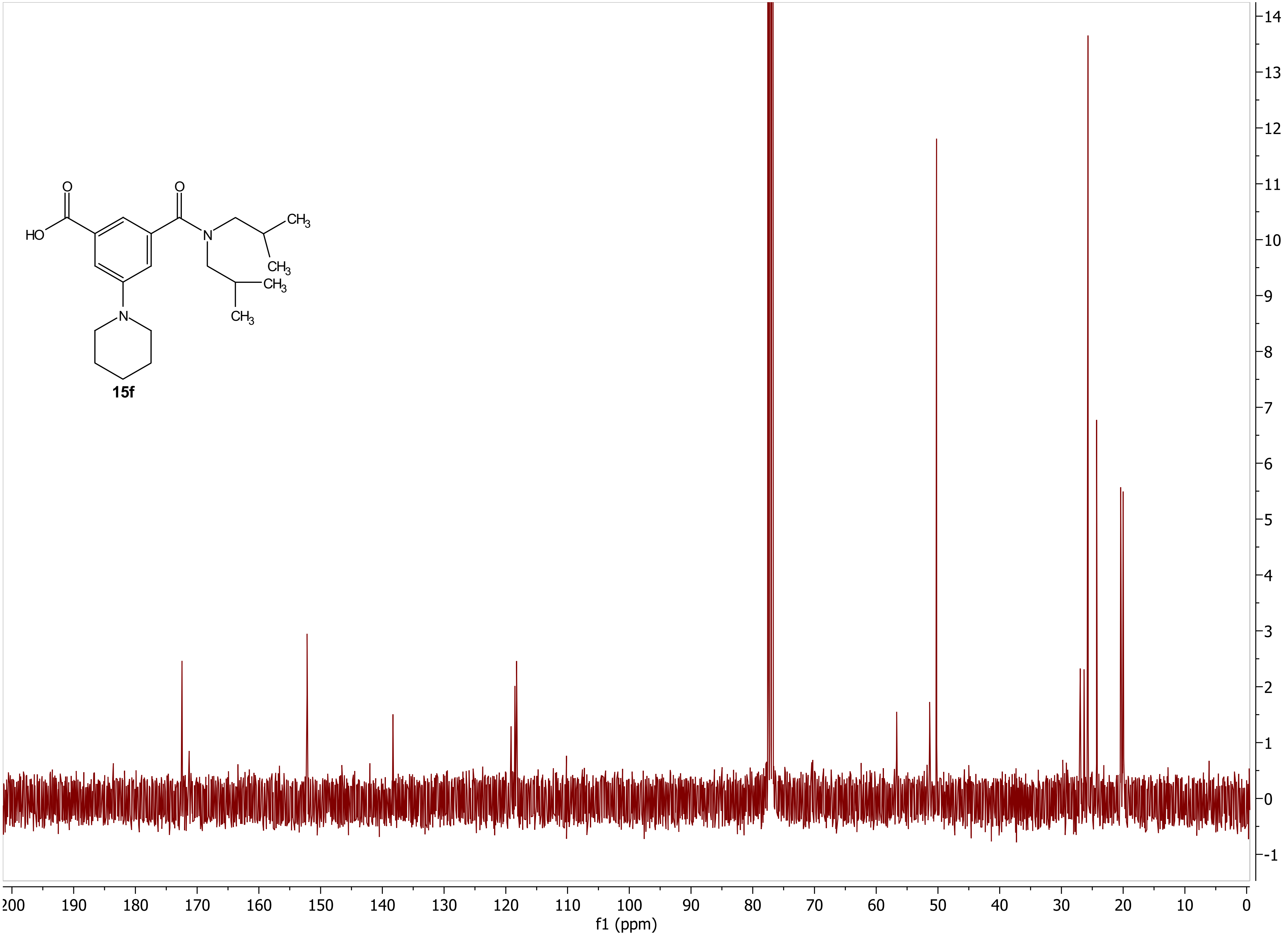
O
O
HO
N
CH3
CH3
CH3
CH3
N
15f
200
190
180
170
160
150
140
130
120
110
100
90
80
70
60
50
40
30
20
10
0
f1 (ppm)
14
13
12
11
10
9
8
7
6
5
4
3
2
1
0
-1

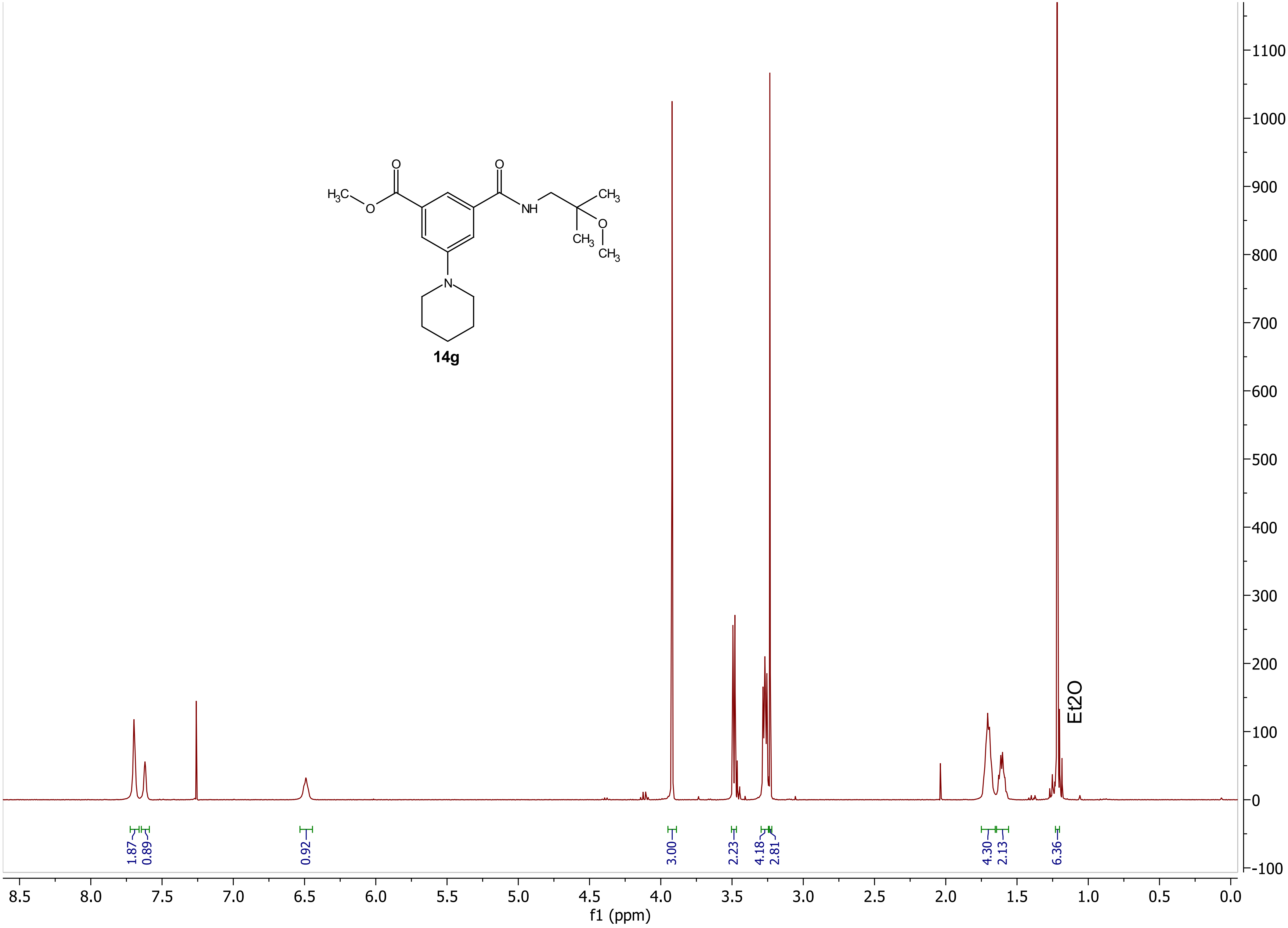
14g
H3C O O
O NH CH3 O CH3 CH3
N
Et2O
1.87 0.89 0.92 3.00 2.23 4.18 2.81 4.30 2.13 6.36
8.5 8.0 7.5 7.0 6.5 6.0 5.5 5.0 4.5 4.0 3.5 3.0 2.5 2.0 1.5 1.0 0.5 0.0
f1 (ppm)
1100 1000 900 800 700 600 500 400 300 200 100 0 -100

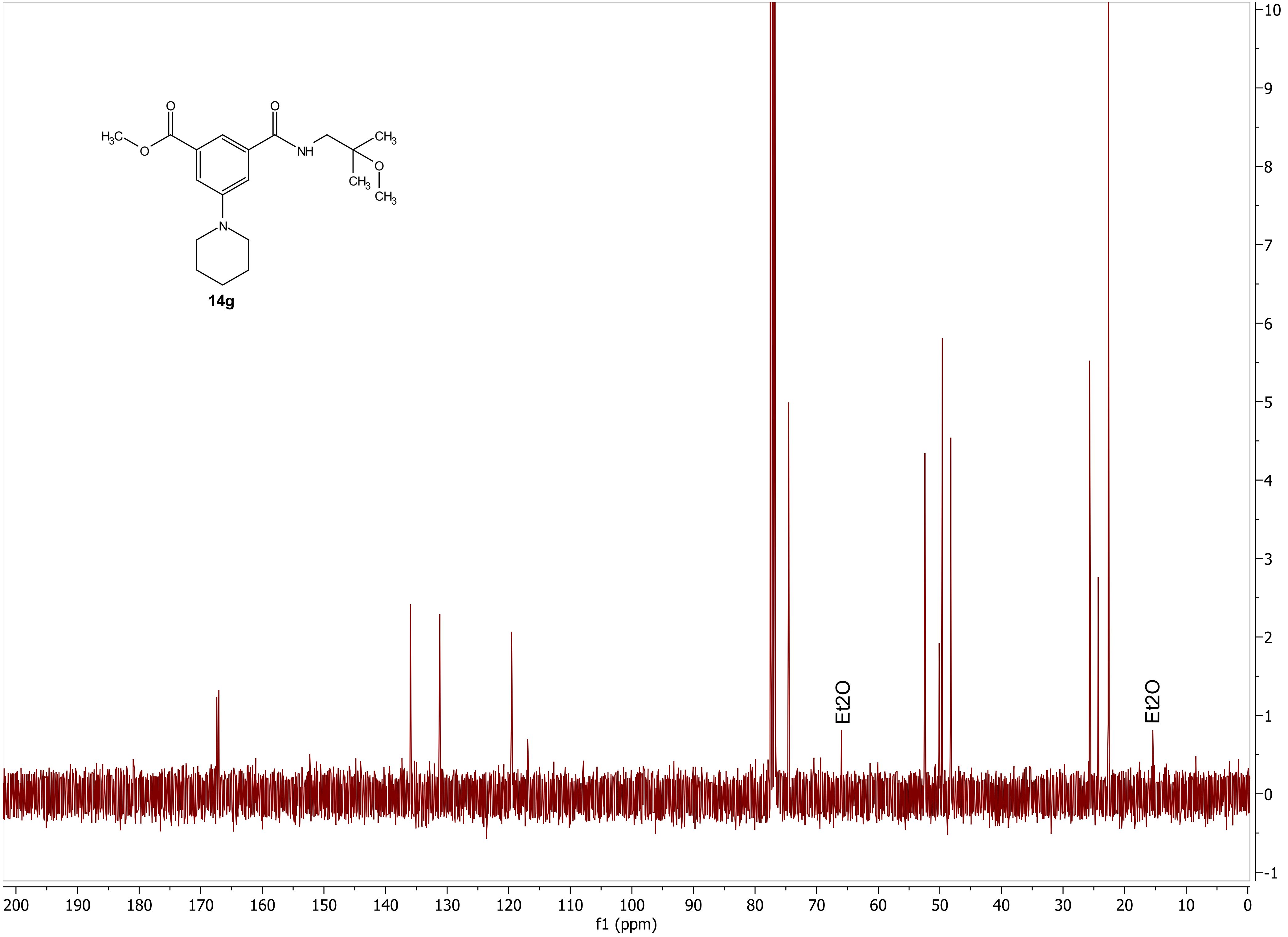
14g
Et2O
Et2O
f1 (ppm)

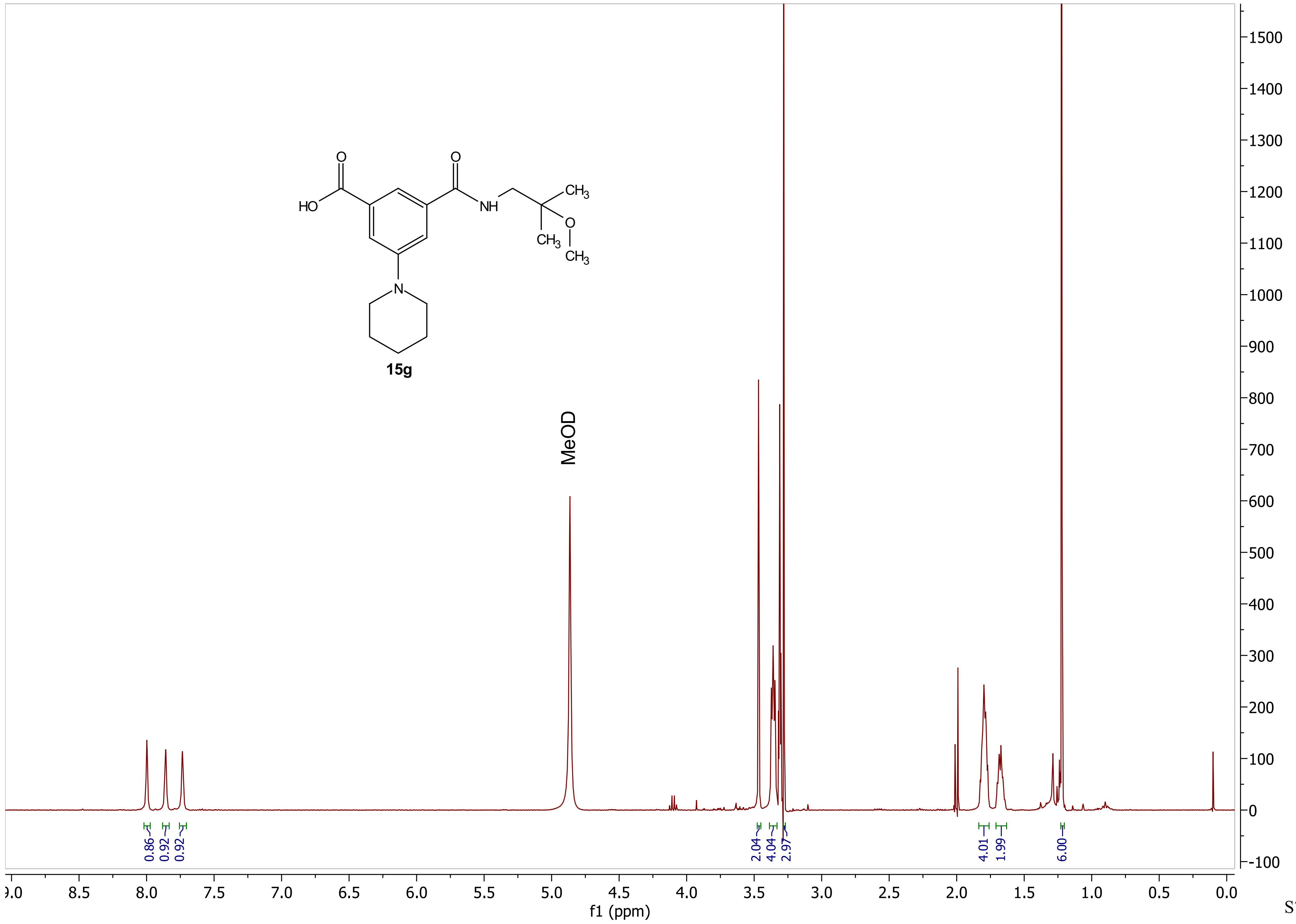
HO
O
O
NH
CH3
O
CH3
CH3
N
15g
MeOD
0.86
0.92
0.92
2.04
4.04
2.97
4.01
1.99
6.00
f1 (ppm)

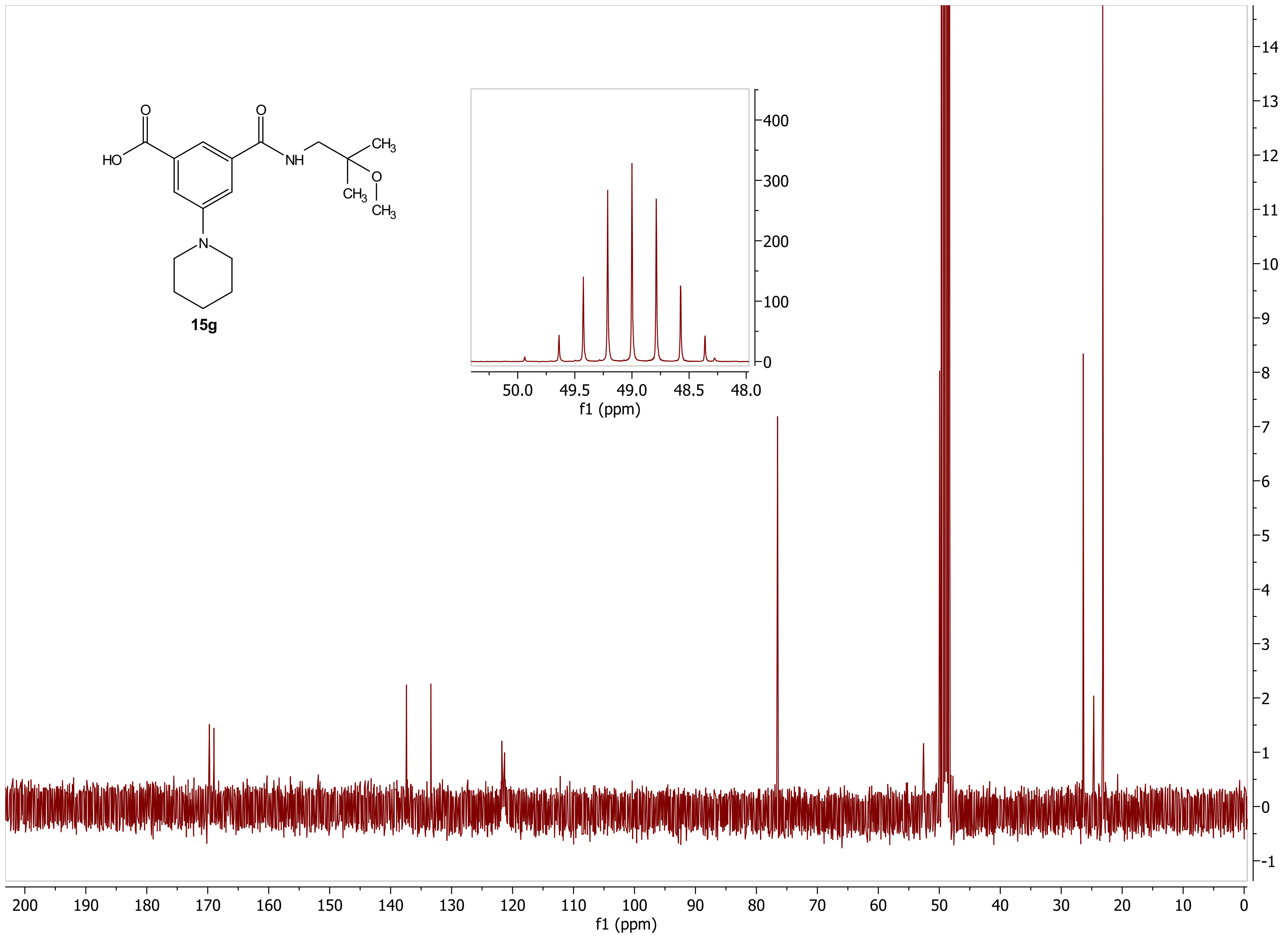
HO
O
O
NH
CH3
O
CH3
CH3
N
15g
f1 (ppm)
f1 (ppm)

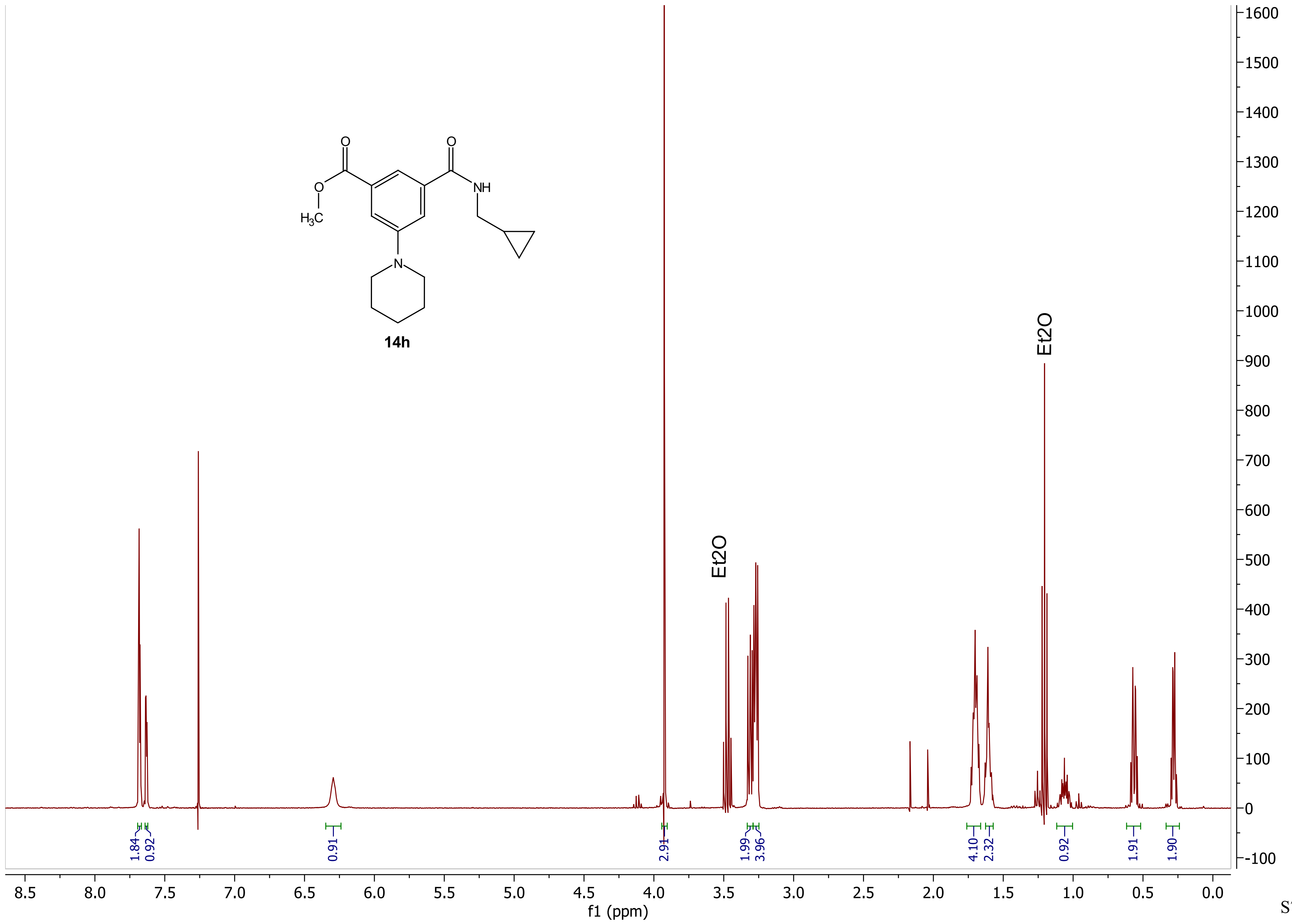
O
O
O
NH
H3C
N
14h
Et2O
Et2O
1.84
0.92
0.91
2.91
1.99
3.96
4.10
2.32
0.92
1.91
1.90
f1 (ppm)

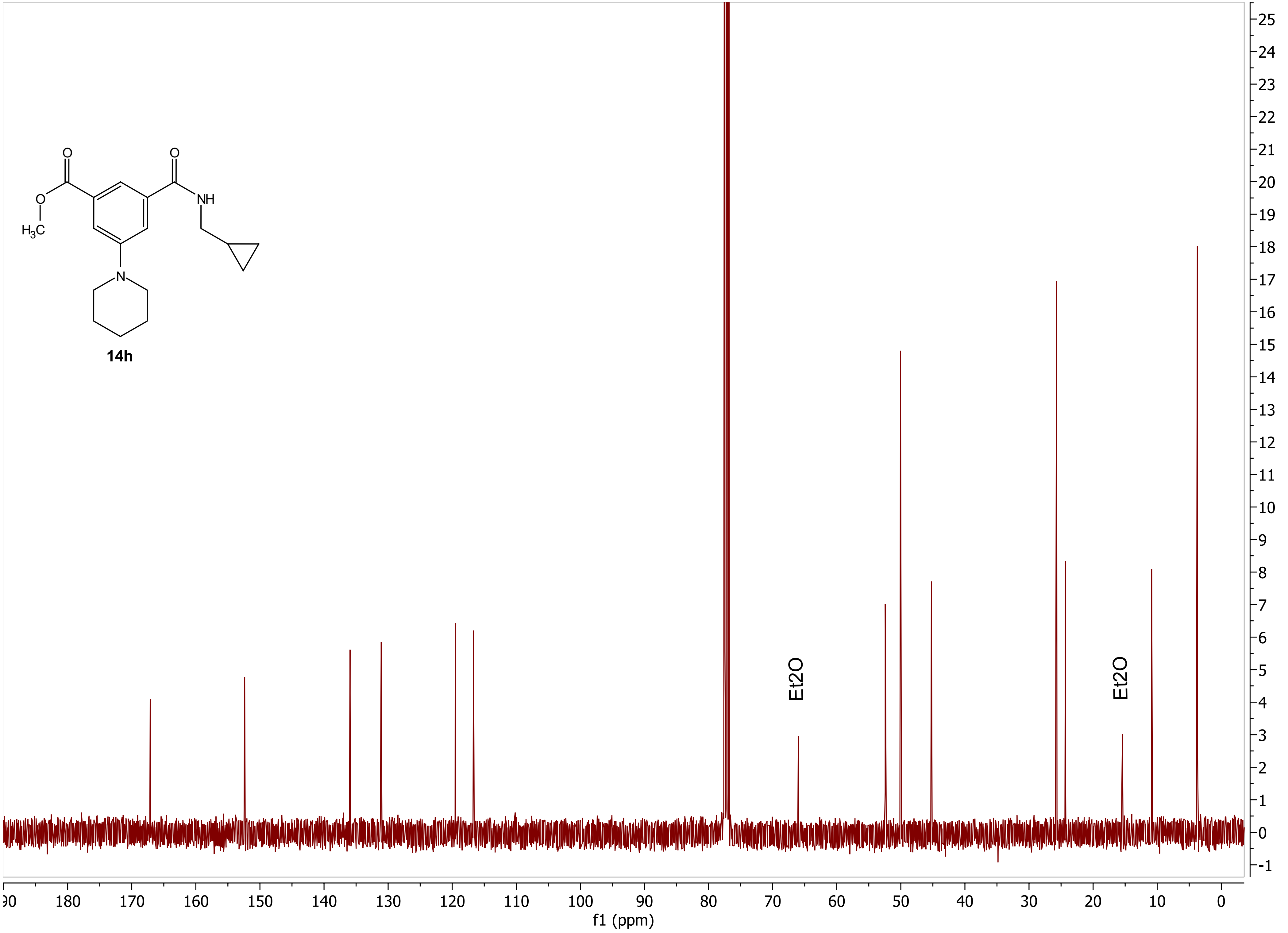

14h
Et2O
Et2O
f1 (ppm)

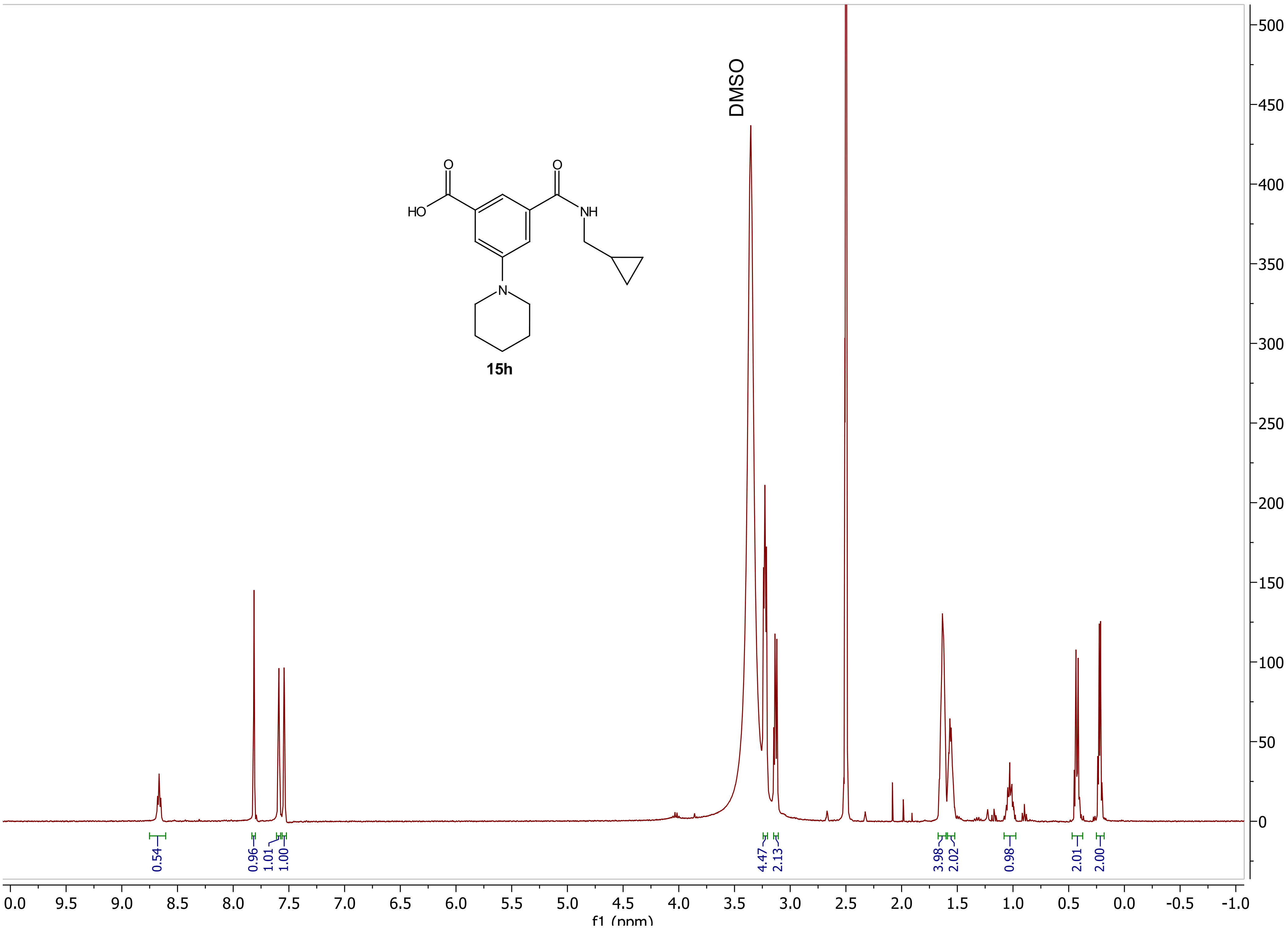
DMSO
HO
O
O
NH
N
15h
0.54
0.96
1.01
1.00
4.47
2.13
3.98
2.02
0.98
2.01
2.00
f1 (ppm)

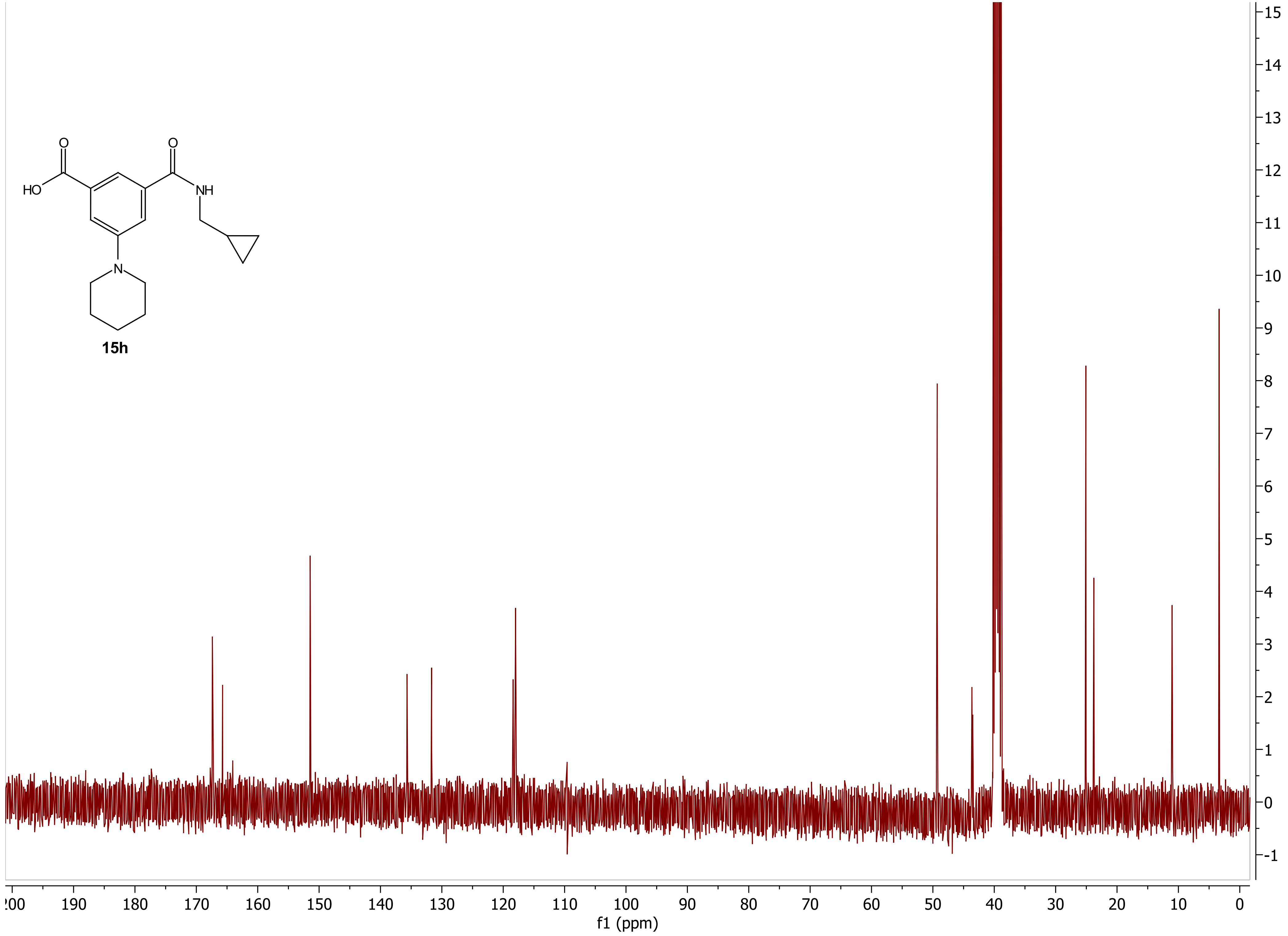

O
O
HO
NH
N
15h
f1 (ppm)

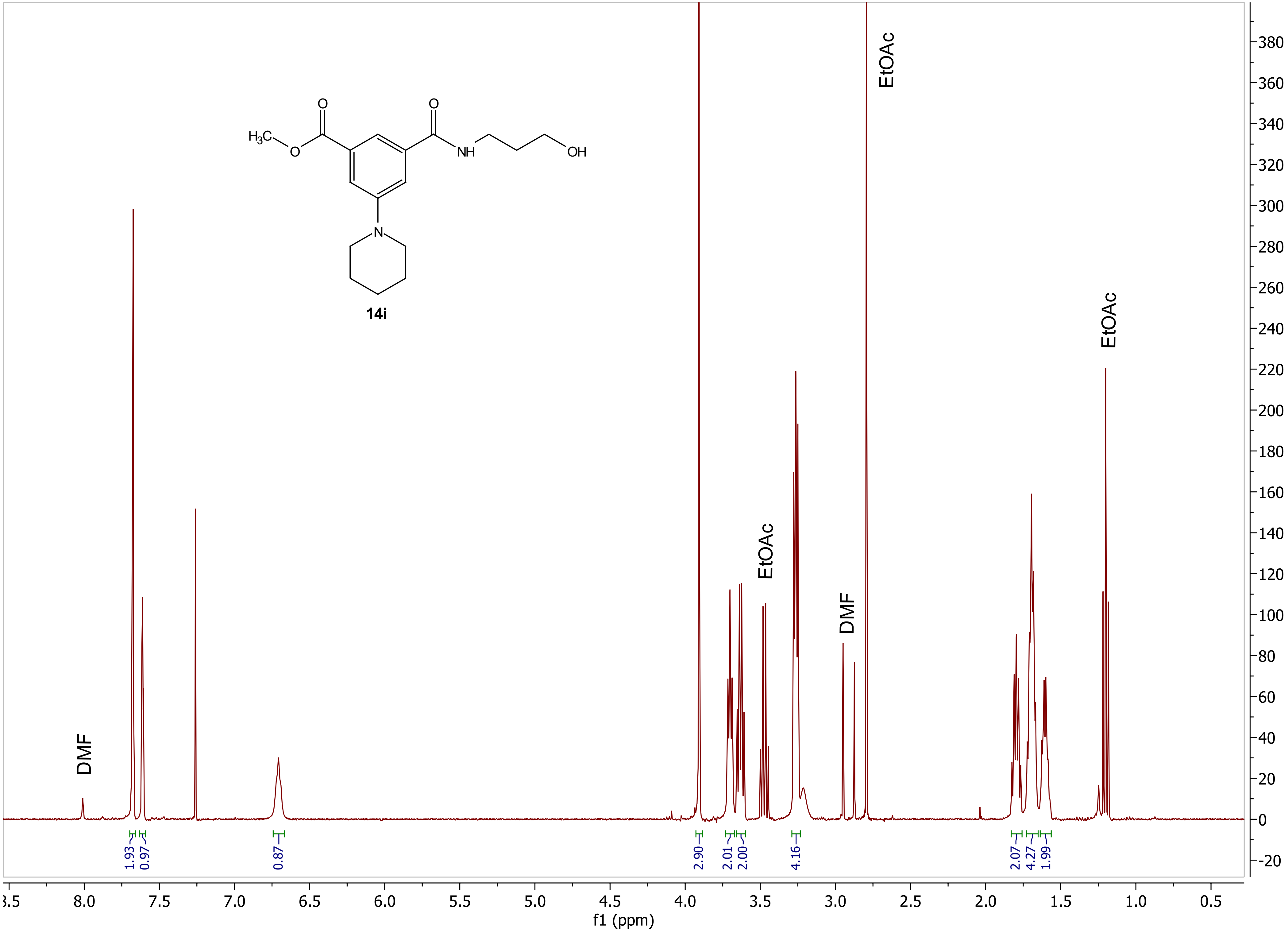

H3C
O
O
O
NH
OH
N
14i
DMF
EtOAc
DMF
EtOAc
EtOAc
1.93
0.97
0.87
2.90
2.01
2.00
4.16
2.07
4.27
1.99
f1 (ppm)

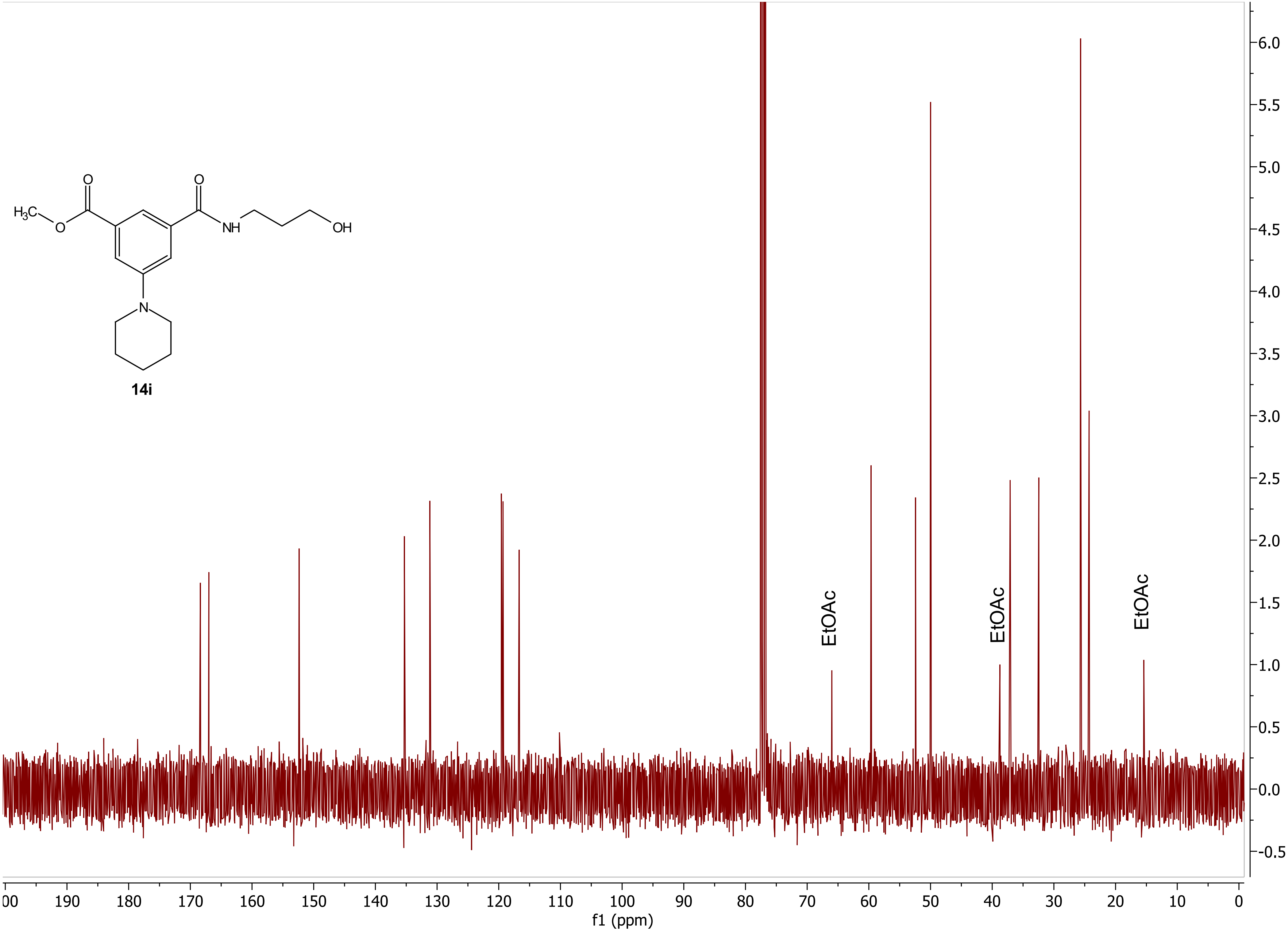
H3C
O
O
O
NH
OH
N
14i
EtOAc
EtOAc
EtOAc
f1 (ppm)

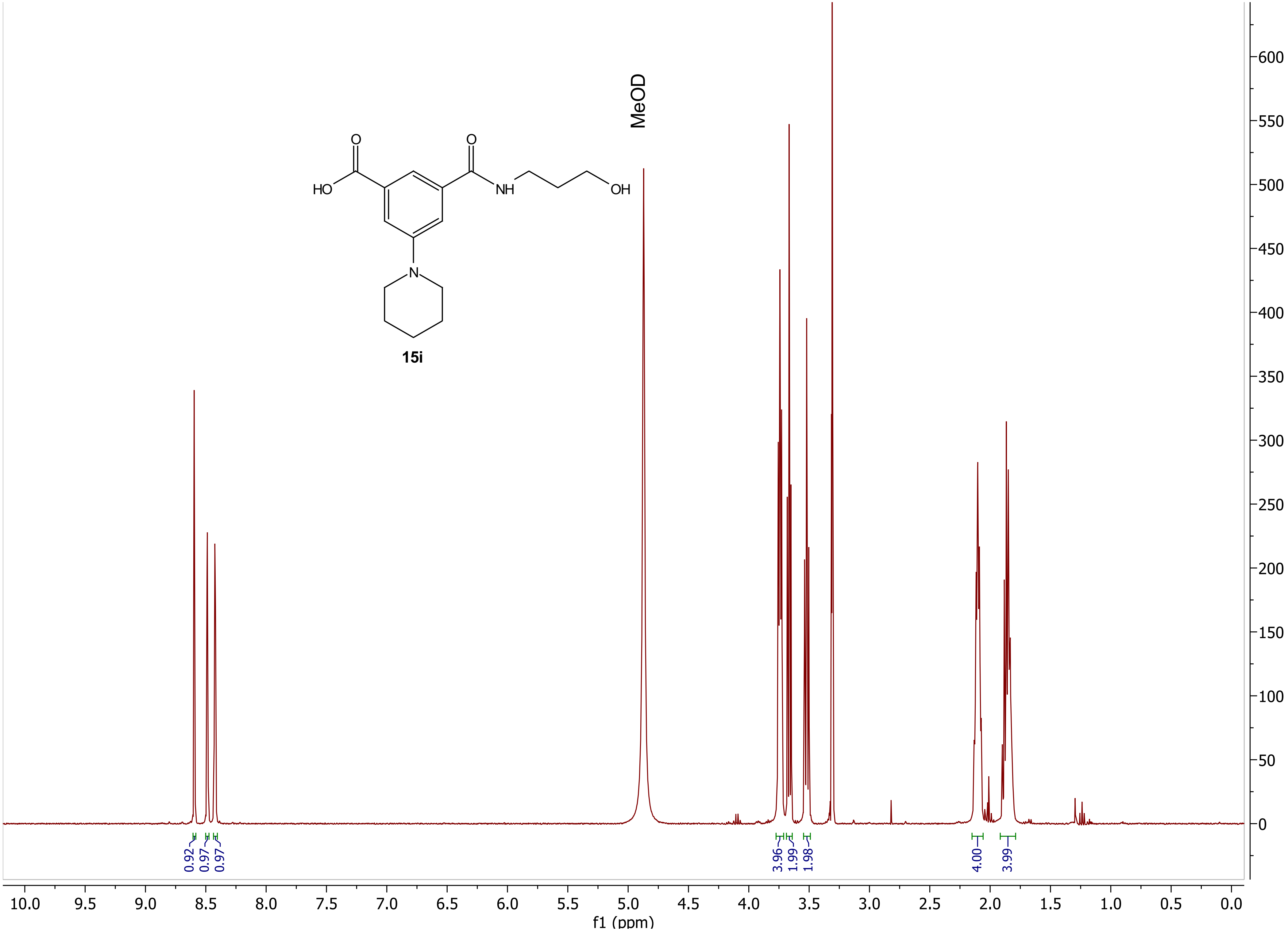

MeOD
15i
HO
O
NH
OH
N
0.92
0.97
0.97
3.96
1.99
1.98
4.00
3.99
f1 (ppm)

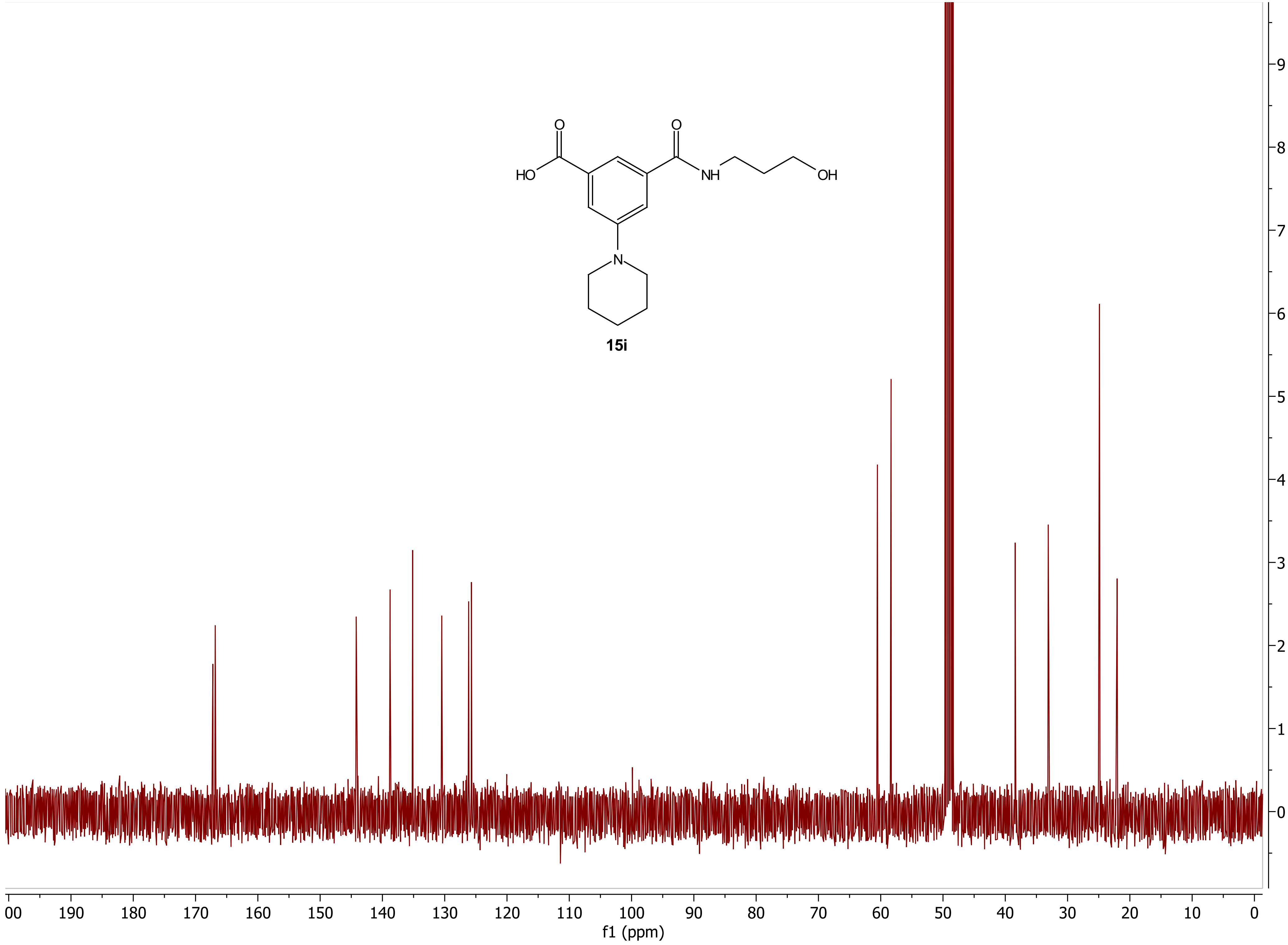
O
O
HO
NH
OH
N
15i
00
190
180
170
160
150
140
130
120
110
100
90
80
70
60
50
40
30
20
10
0
f1 (ppm)
9
8
7
6
5
4
3
2
1
0

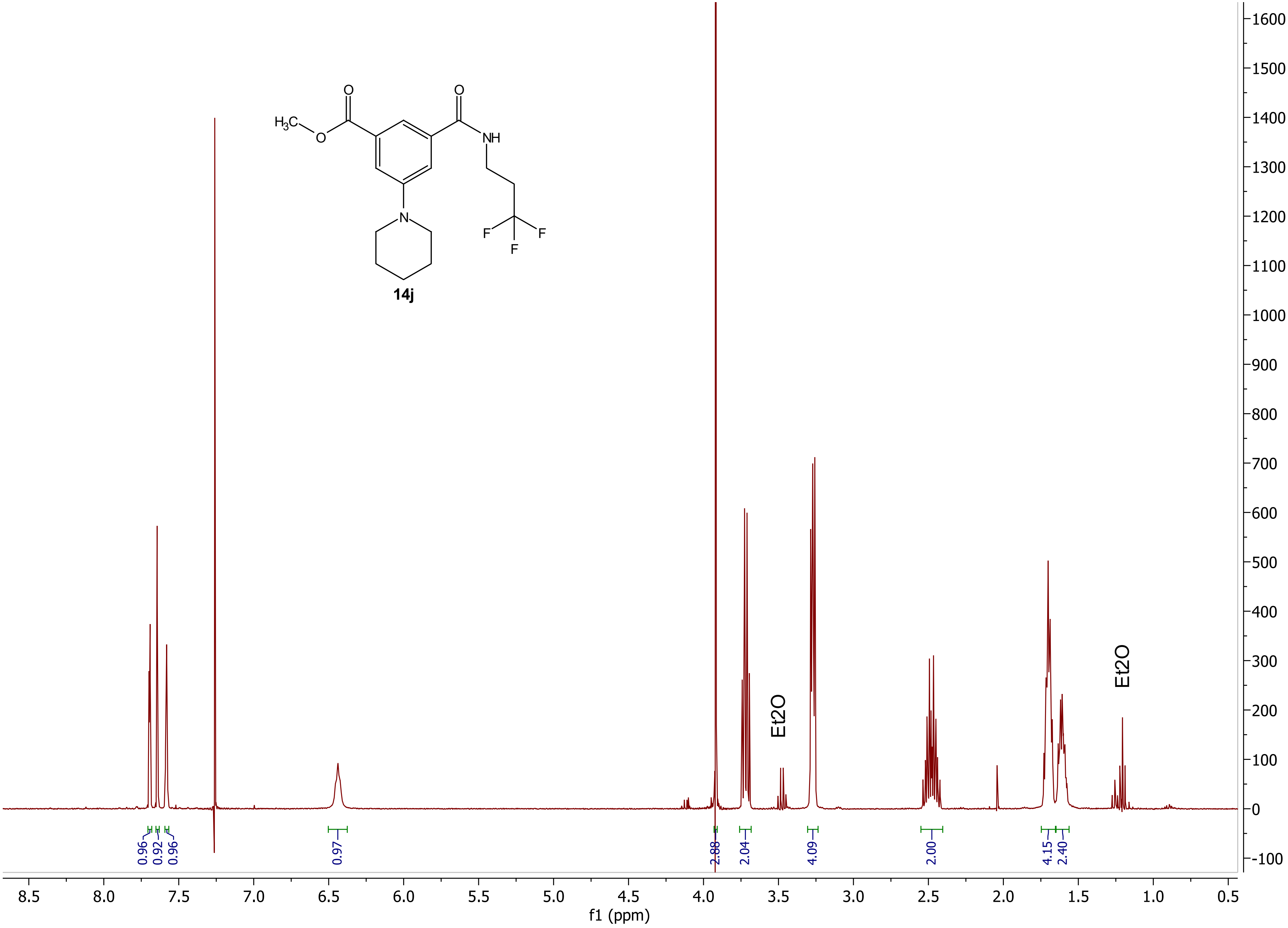

14j
Et2O
Et2O
0.96
0.92
0.96
0.97
2.88
2.04
4.09
2.00
4.15
2.40
f1 (ppm)

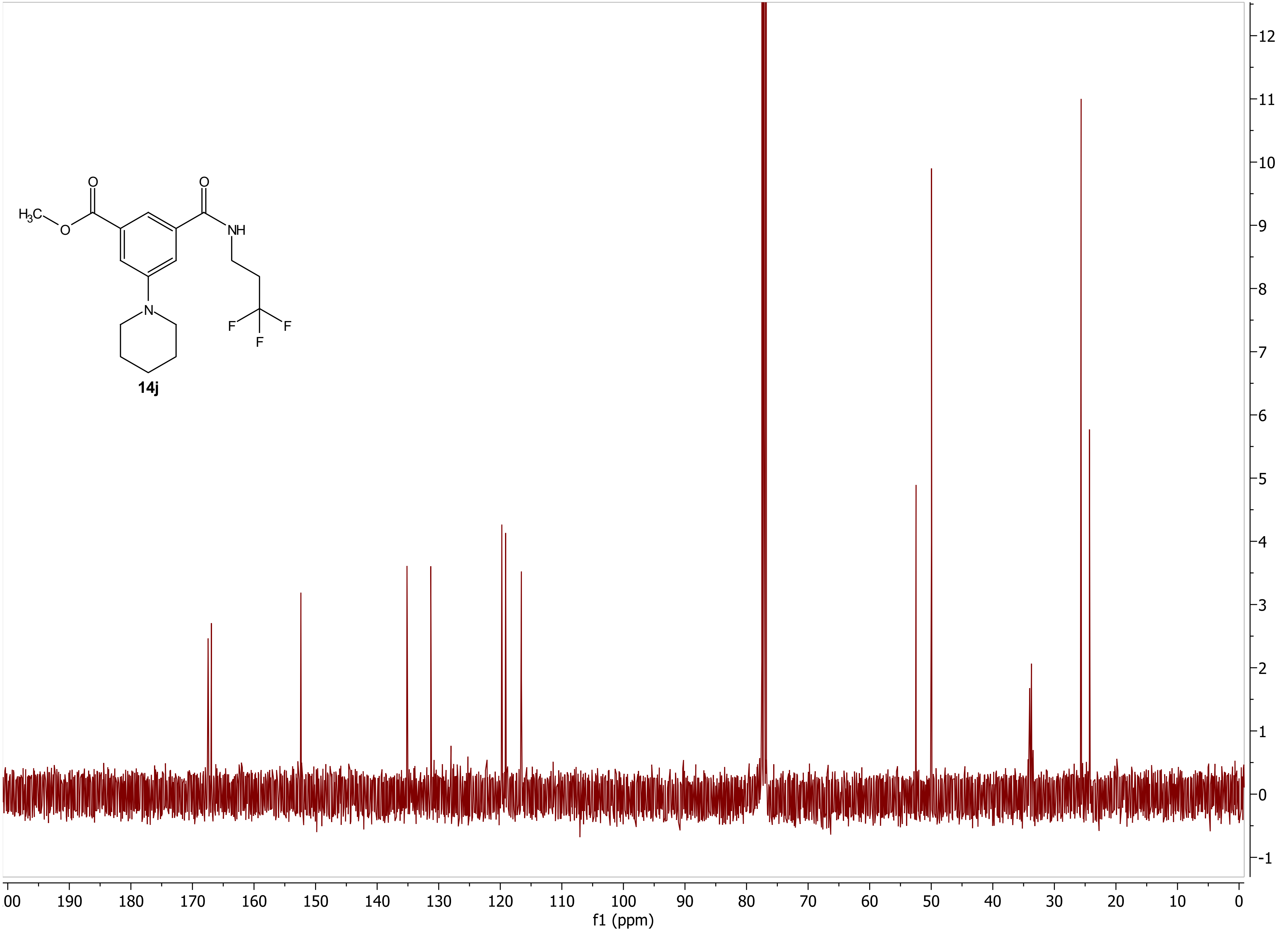

H3C
O
O
O
NH
N
F
F
F
14j
f1 (ppm)

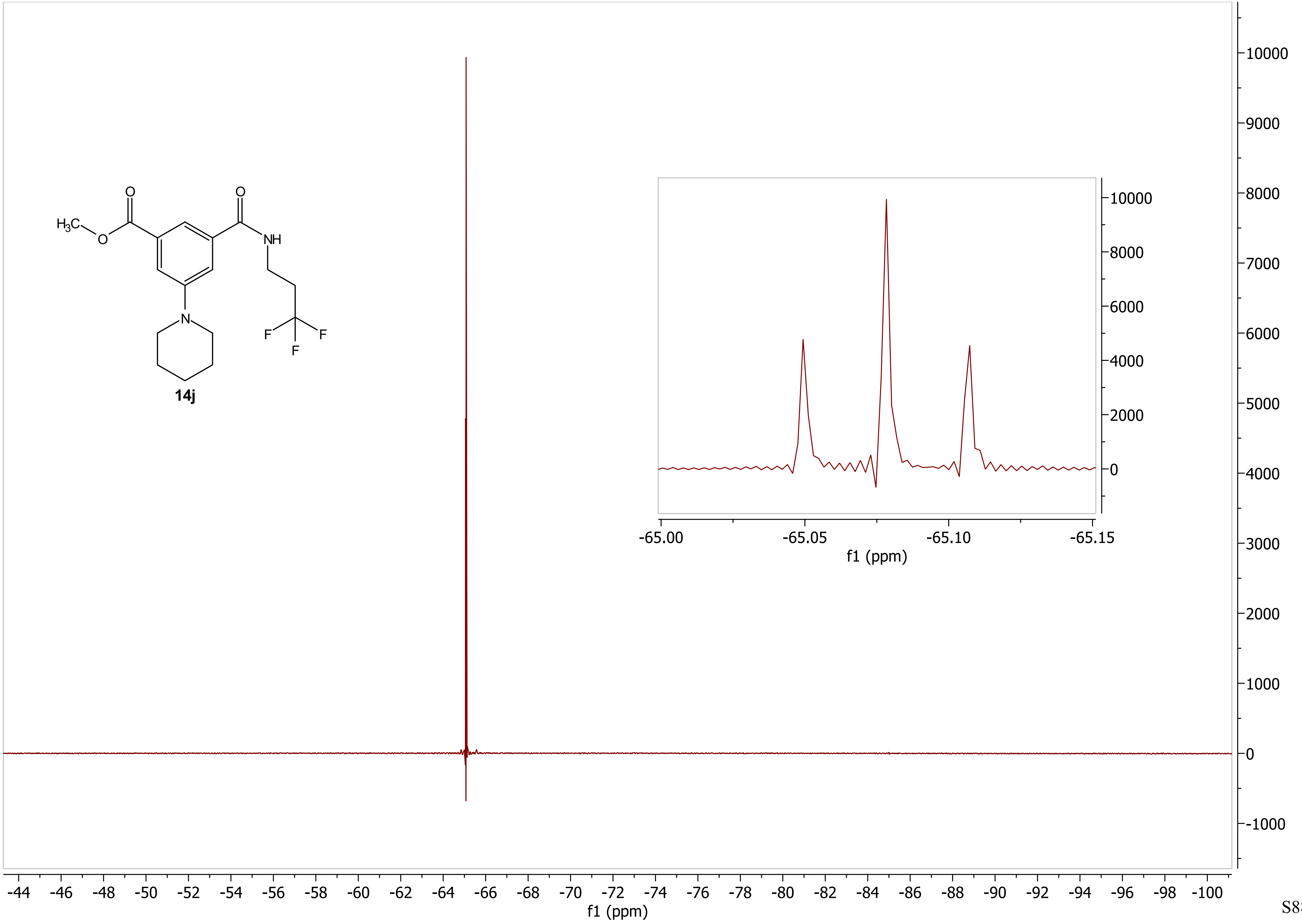

H3C
O
O
O
NH
N
F
F
F
14j
10000
8000
6000
4000
2000
0
-65.00
-65.05
-65.10
-65.15
f1 (ppm)
10000
9000
8000
7000
6000
5000
4000
3000
2000
1000
0
-1000
-44
-46
-48
-50
-52
-54
-56
-58
-60
-62
-64
-66
-68
-70
-72
-74
-76
-78
-80
-82
-84
-86
-88
-90
-92
-94
-96
-98
-100
f1 (ppm)

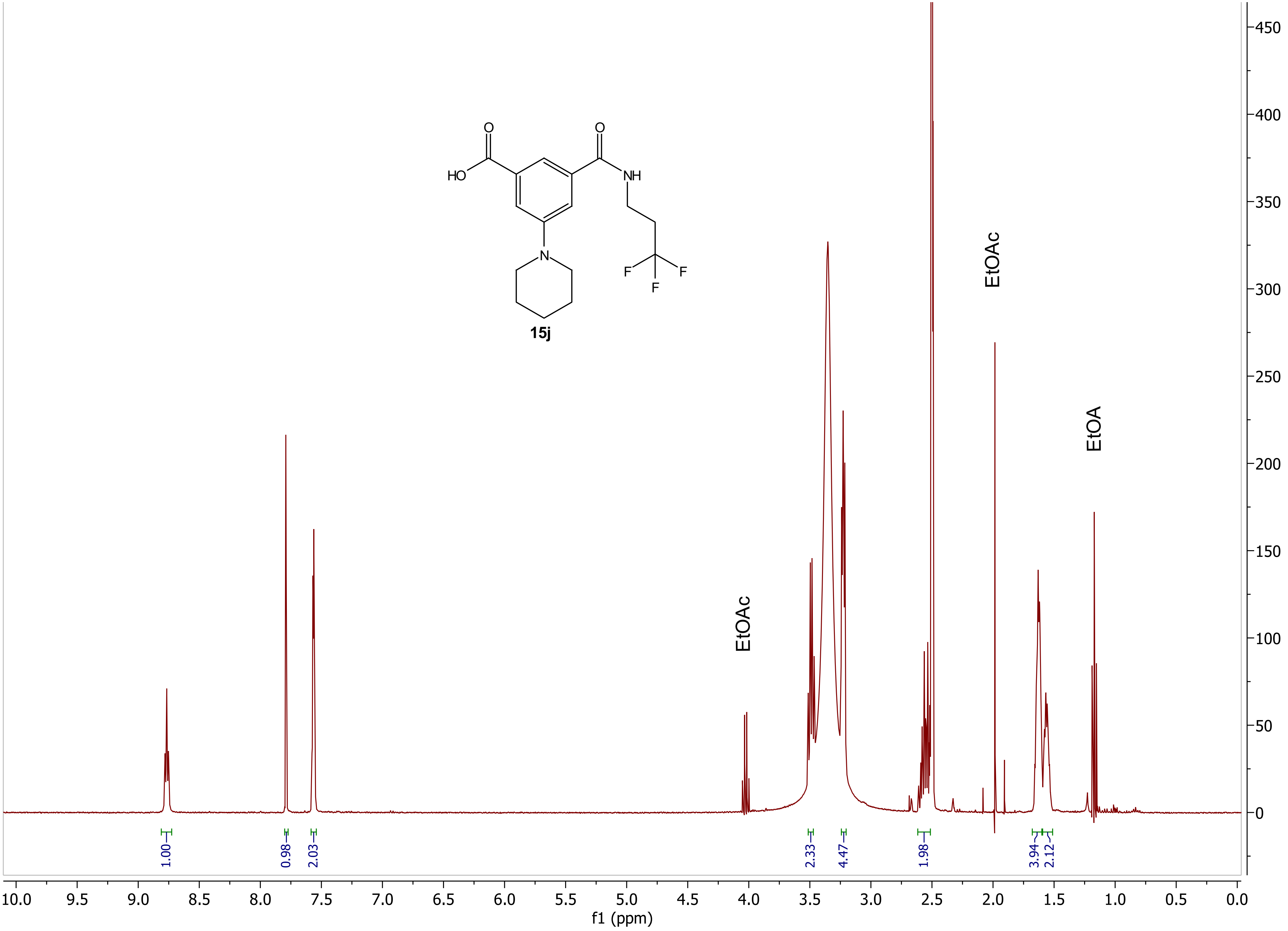
O
HO
O
NH
N
F
F
F
15j
EtOAc
EtOAc
EtOA
1.00
0.98
2.03
2.33
4.47
1.98
3.94
2.12
10.0 9.5 9.0 8.5 8.0 7.5 7.0 6.5 6.0 5.5 5.0 4.5 4.0 3.5 3.0 2.5 2.0 1.5 1.0 0.5 0.0
f1 (ppm)
450 400 350 300 250 200 150 100 50 0

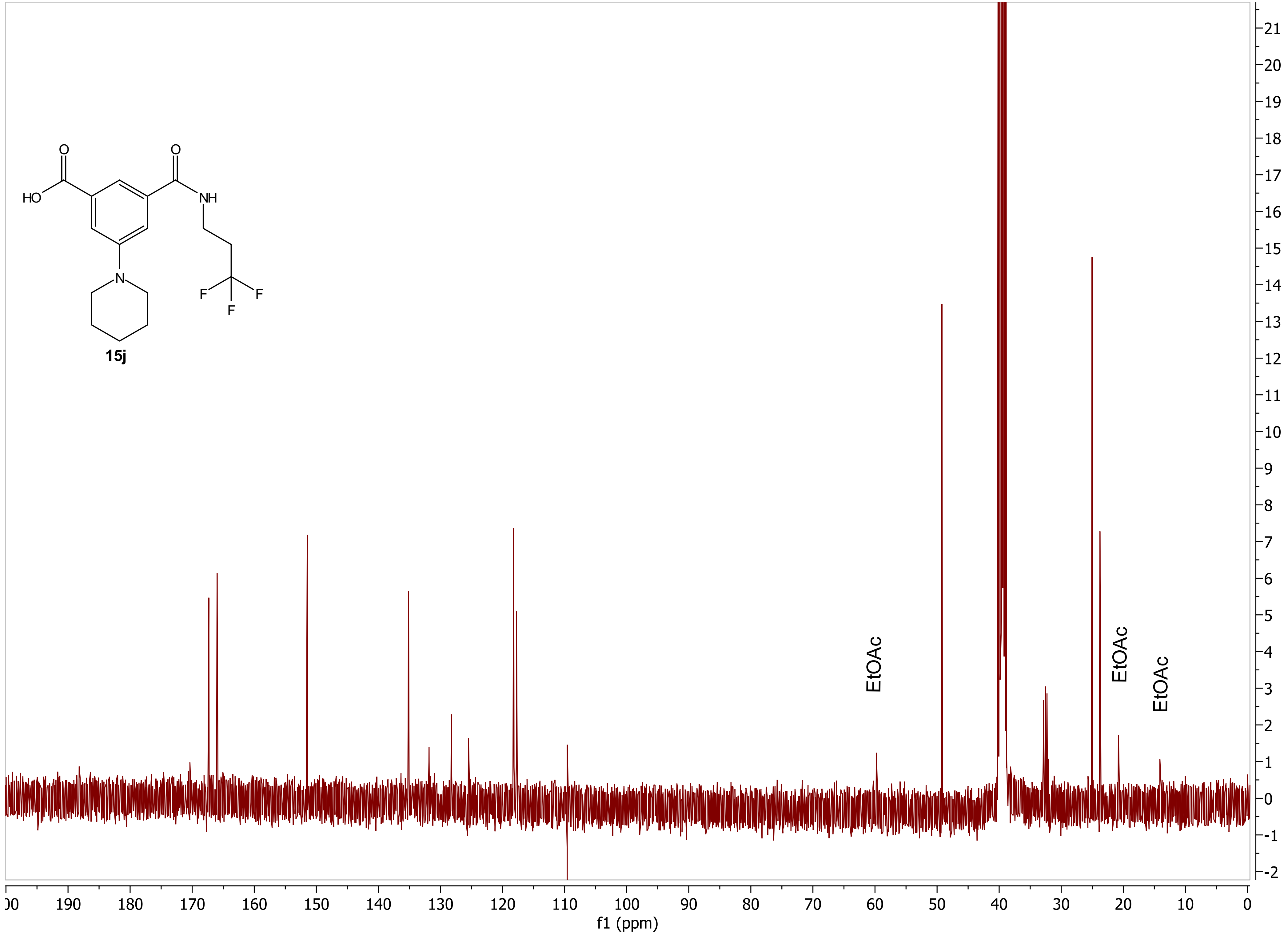

15j
HO
O
NH
N
F
F
F
EtOAc
EtOAc
EtOAc
f1 (ppm)

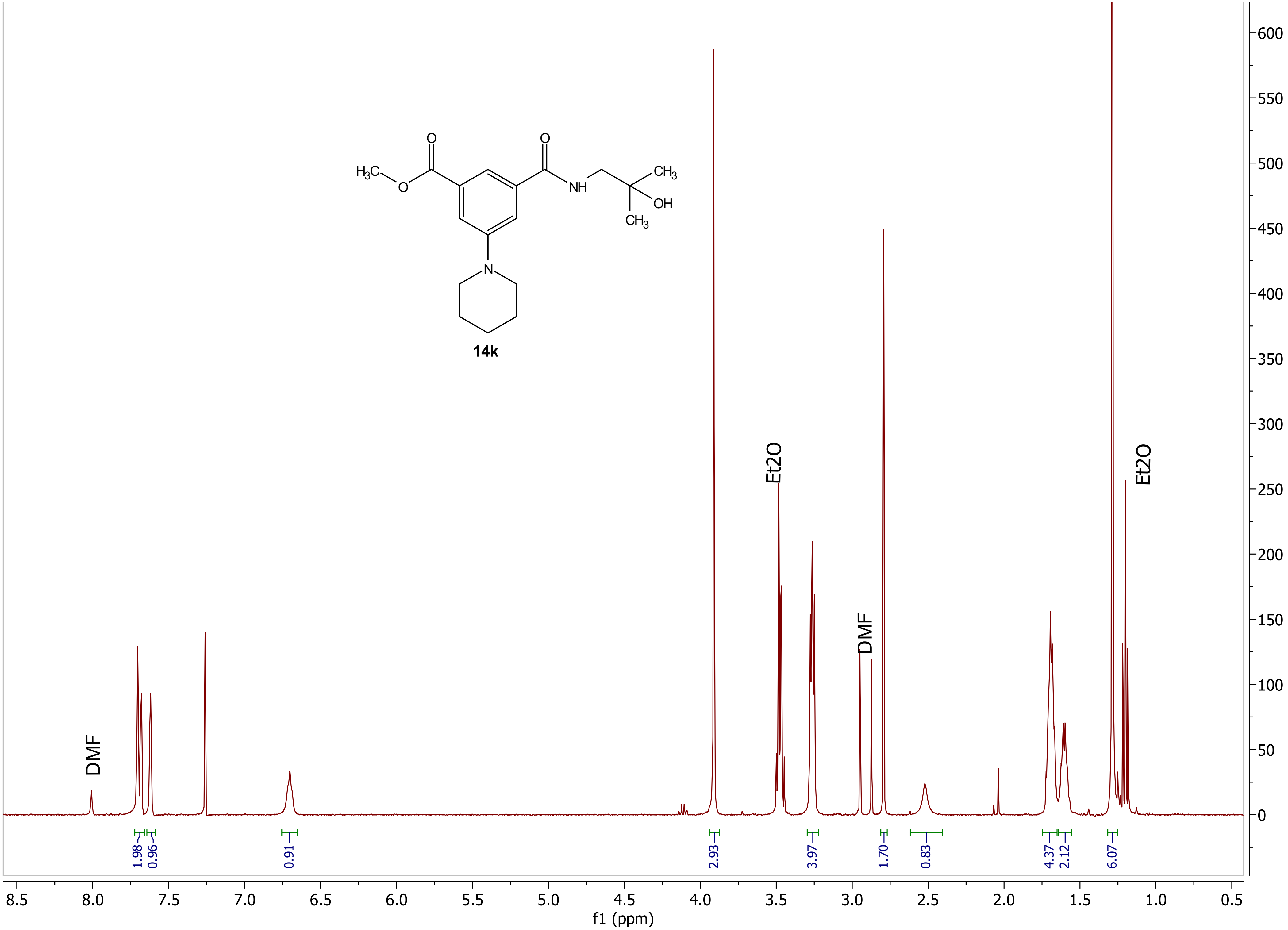
H3C
O
O
O
NH
CH3
OH
CH3
N
14k
DMF
Et2O
DMF
Et2O
1.98
0.96
0.91
2.93
3.97
1.70
0.83
4.37
2.12
6.07
8.5 8.0 7.5 7.0 6.5 6.0 5.5 5.0 4.5 4.0 3.5 3.0 2.5 2.0 1.5 1.0 0.5
f1 (ppm)
600 550 500 450 400 350 300 250 200 150 100 50 0

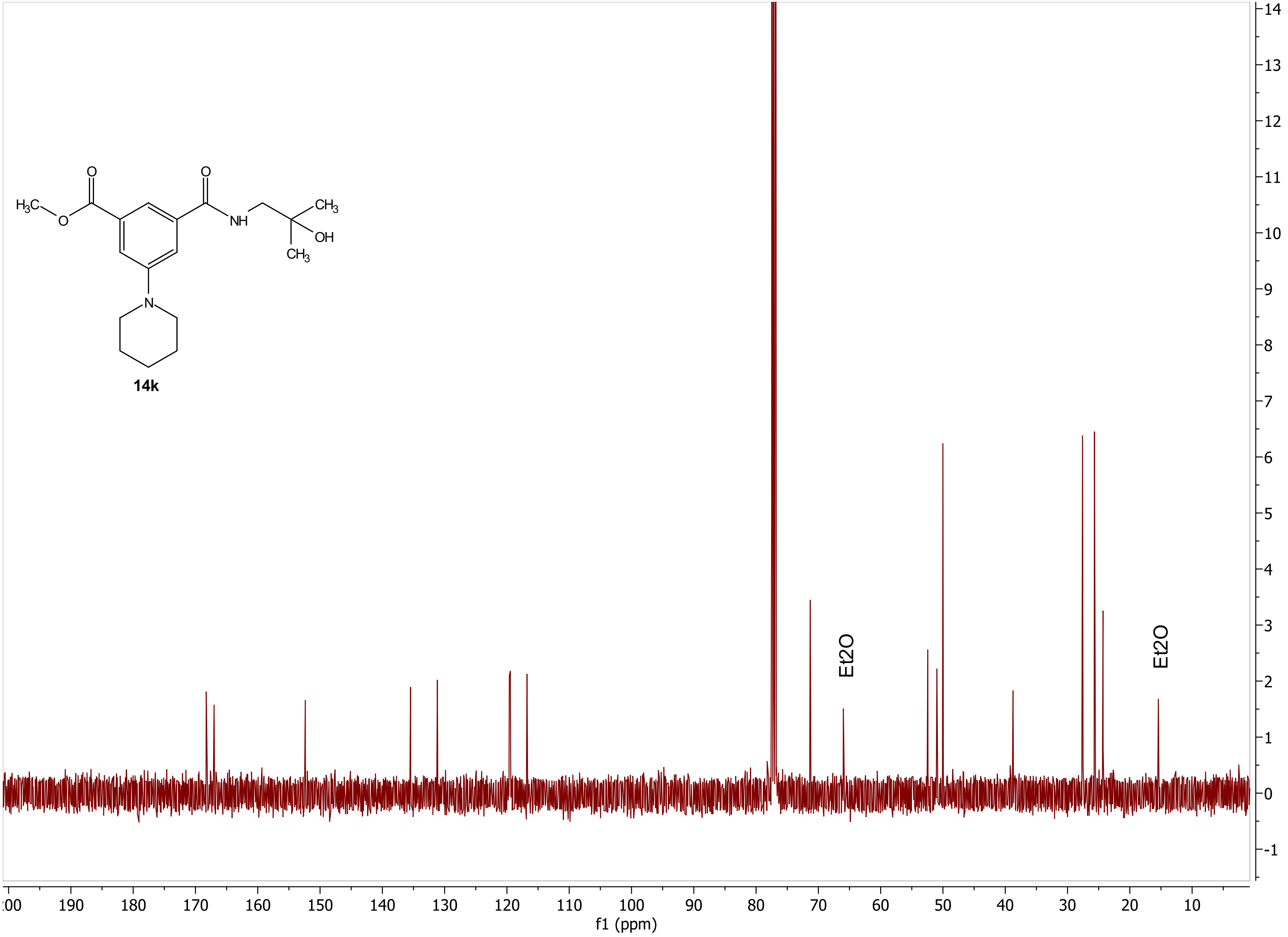

H_3C
O
NH
CH_3
OH
CH_3
N
14k
Et2O
Et2O
f1 (ppm)

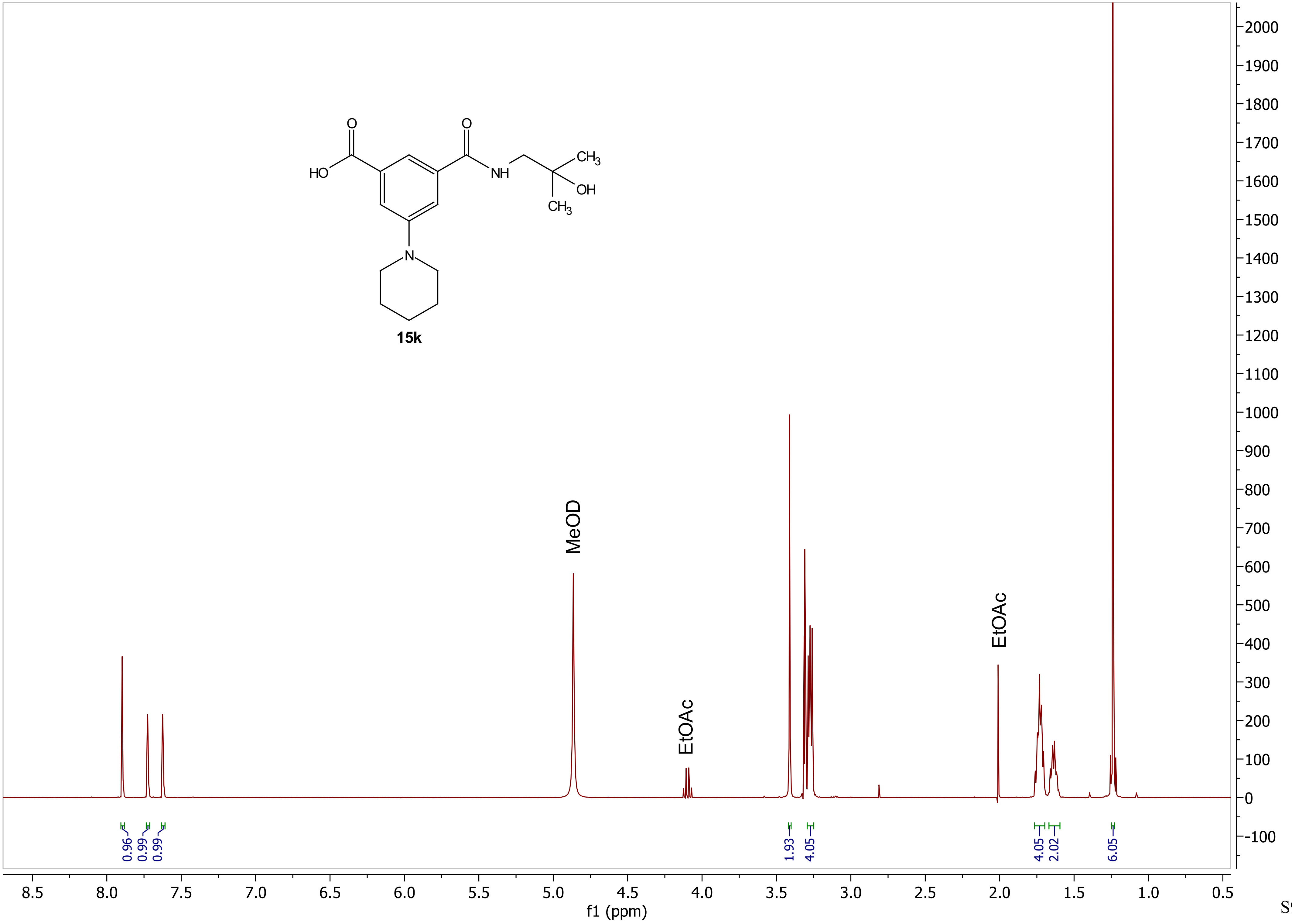

HO
O
O
NH
CH3
OH
CH3
N
15k
MeOD
EtOAc
EtOAc
0.96
0.99
0.99
1.93
4.05
4.05
2.02
6.05
f1 (ppm)

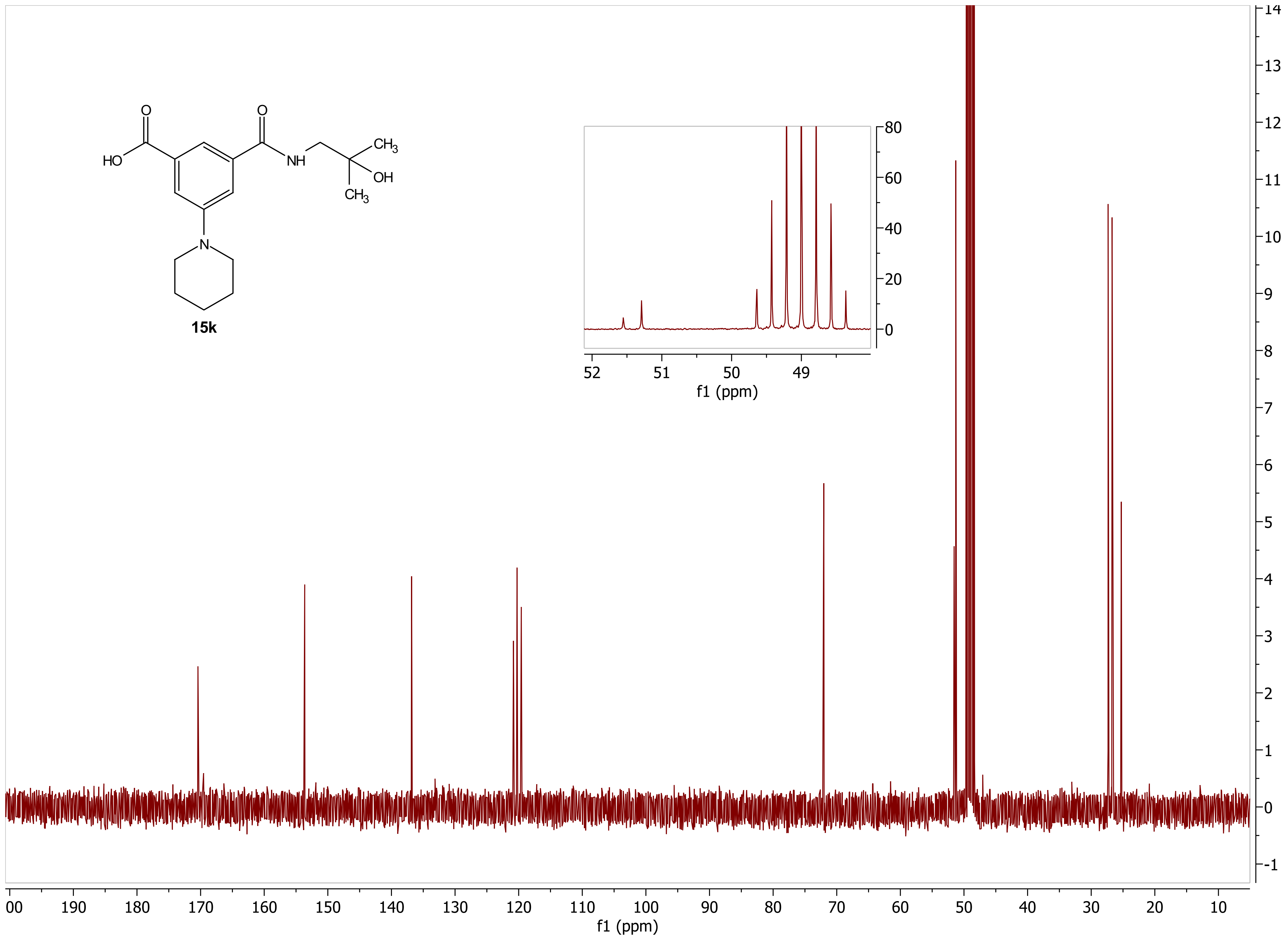

HO
O
O
NH
CH3
OH
CH3
N
15k
52
51
50
49
f1 (ppm)
f1 (ppm)

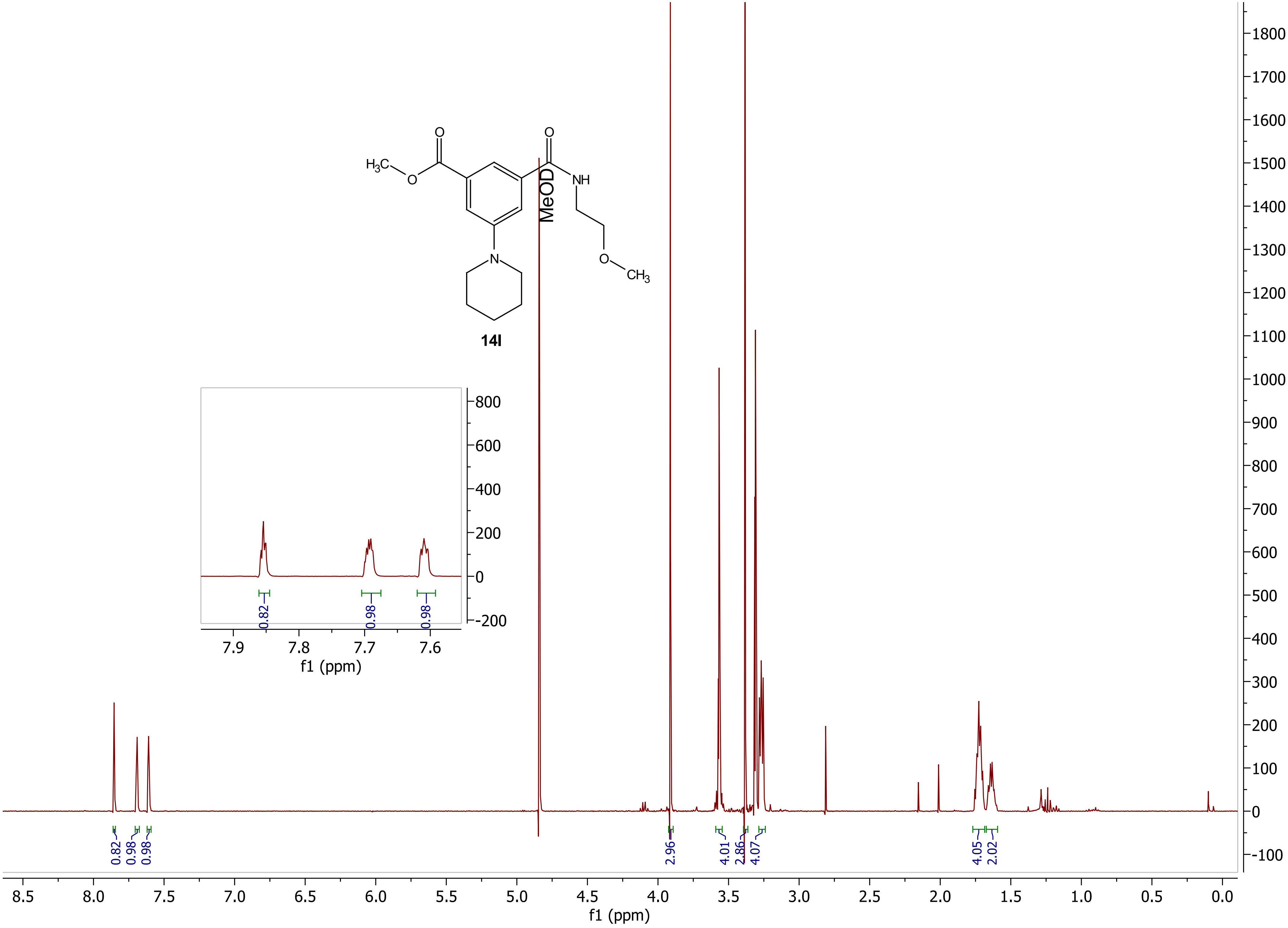
H3C
O
O
O
NH
N
O
CH3
MeOD
7.9
7.8
7.7
7.6
f1 (ppm)
0.82
0.98
0.98
2.96
4.01
2.86
4.07
4.05
2.02
f1 (ppm)

14l

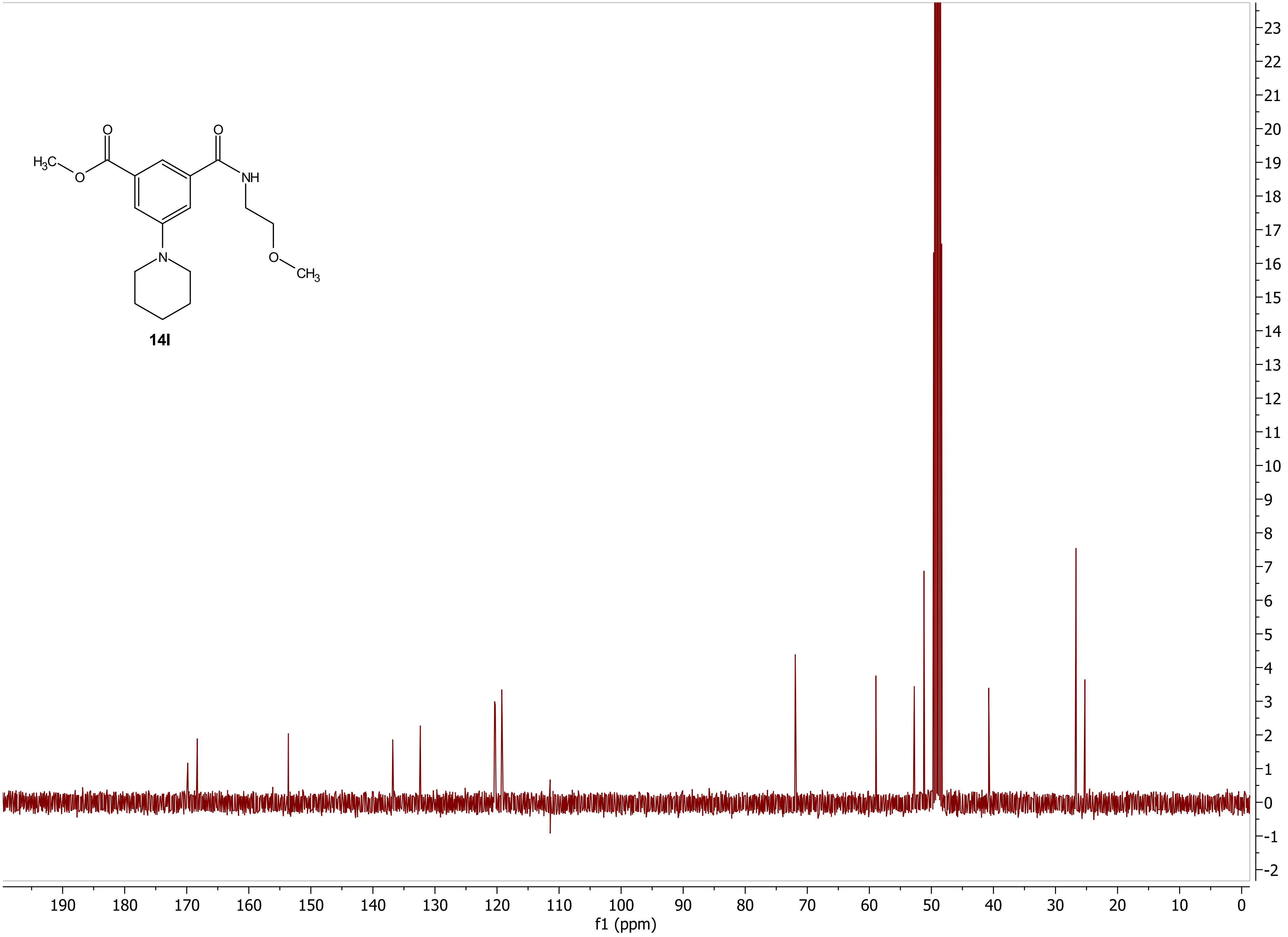

14l

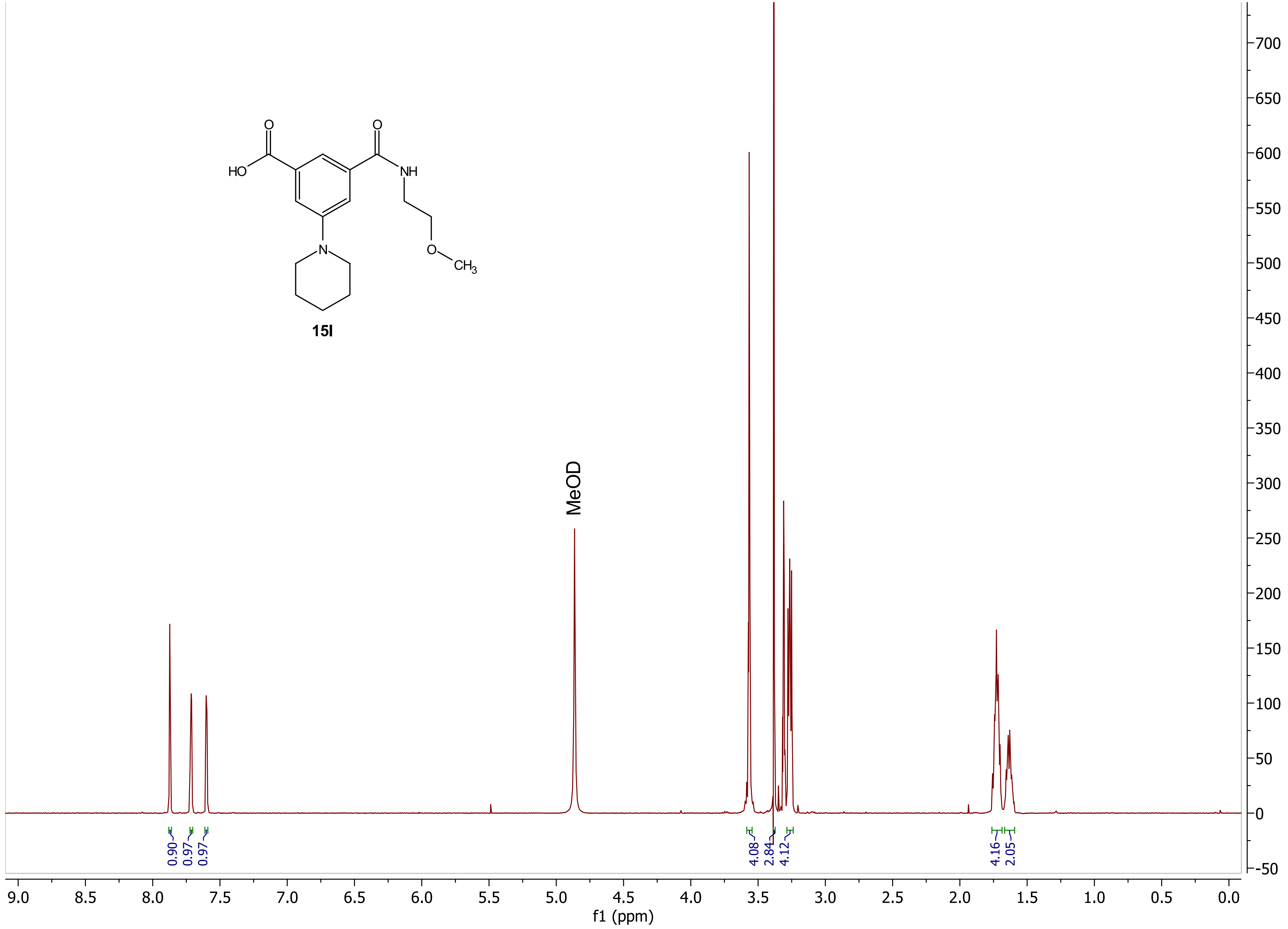
HO
O
O
NH
N
O
CH3
15l
MeOD
0.90
0.97
0.97
4.08
2.84
4.12
4.16
2.05
f1 (ppm)

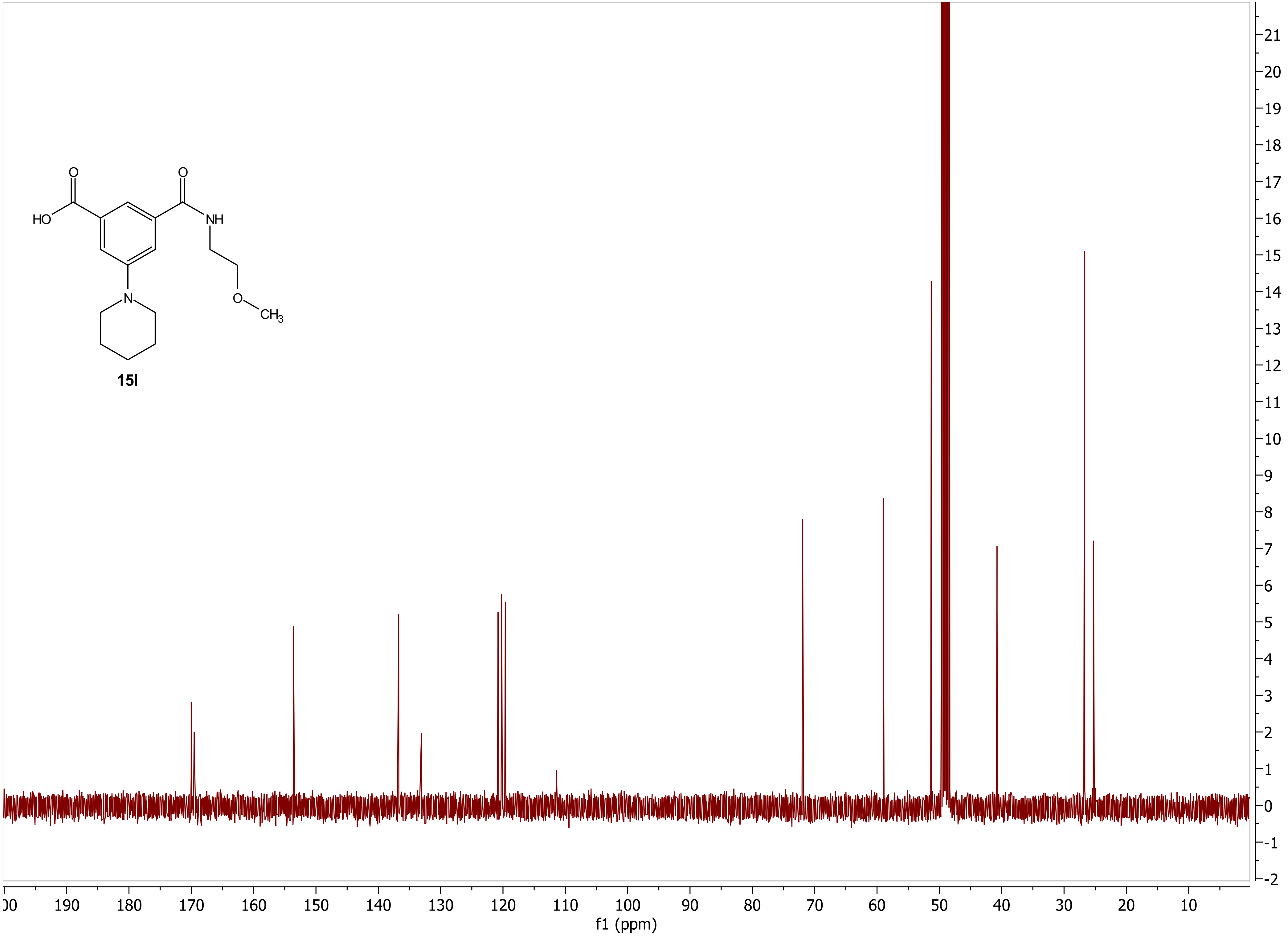

O
O
HO
NH
N
O
CH3
15l
f1 (ppm)

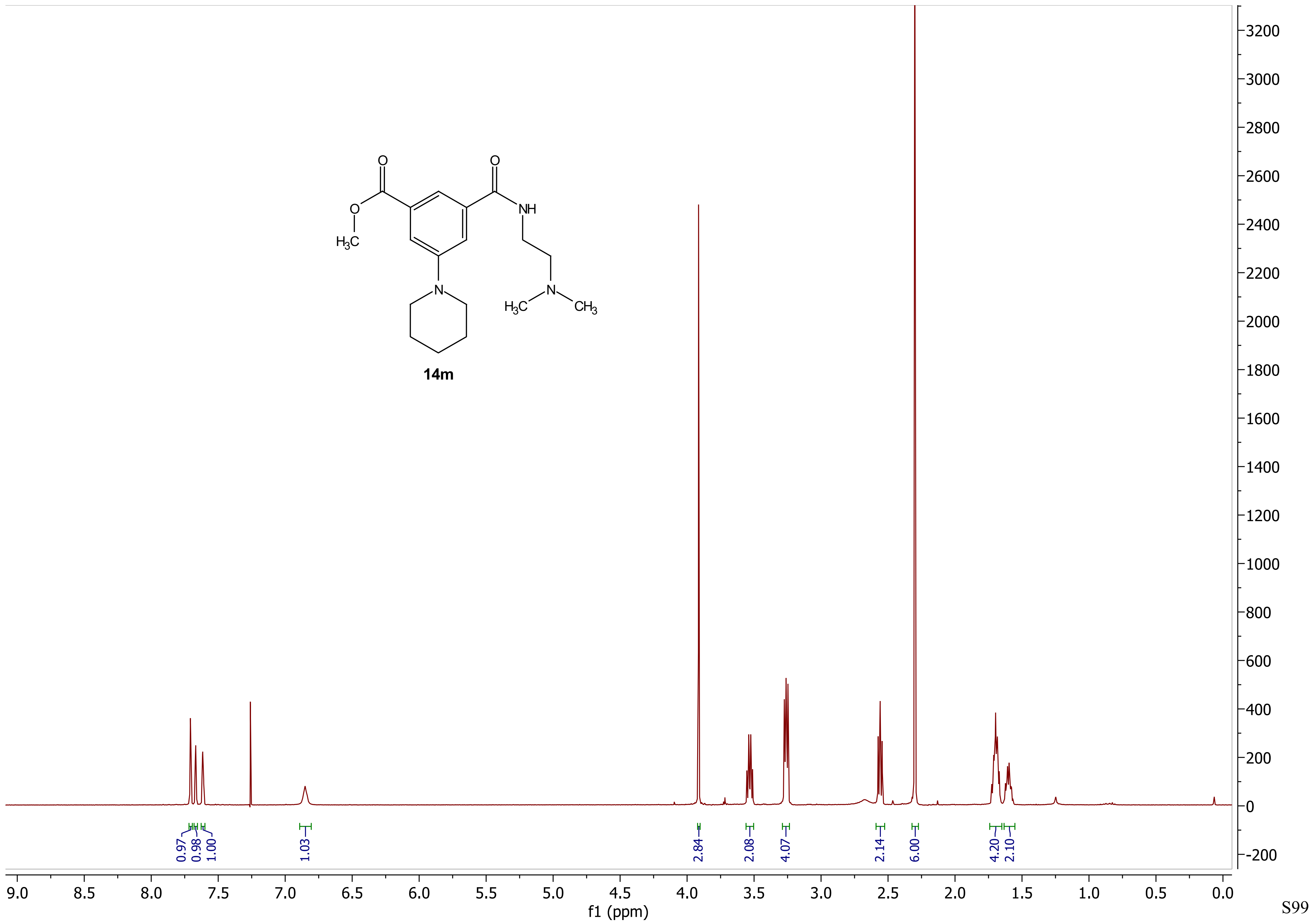
O
O
H3C
O
NH
N
H3C
N
CH3
14m
0.97
0.98
1.00
1.03
2.84
2.08
4.07
2.14
6.00
4.20
2.10
9.0 8.5 8.0 7.5 7.0 6.5 6.0 5.5 5.0 4.5 4.0 3.5 3.0 2.5 2.0 1.5 1.0 0.5 0.0
f1 (ppm)
3200 3000 2800 2600 2400 2200 2000 1800 1600 1400 1200 1000 800 600 400 200 0 -200

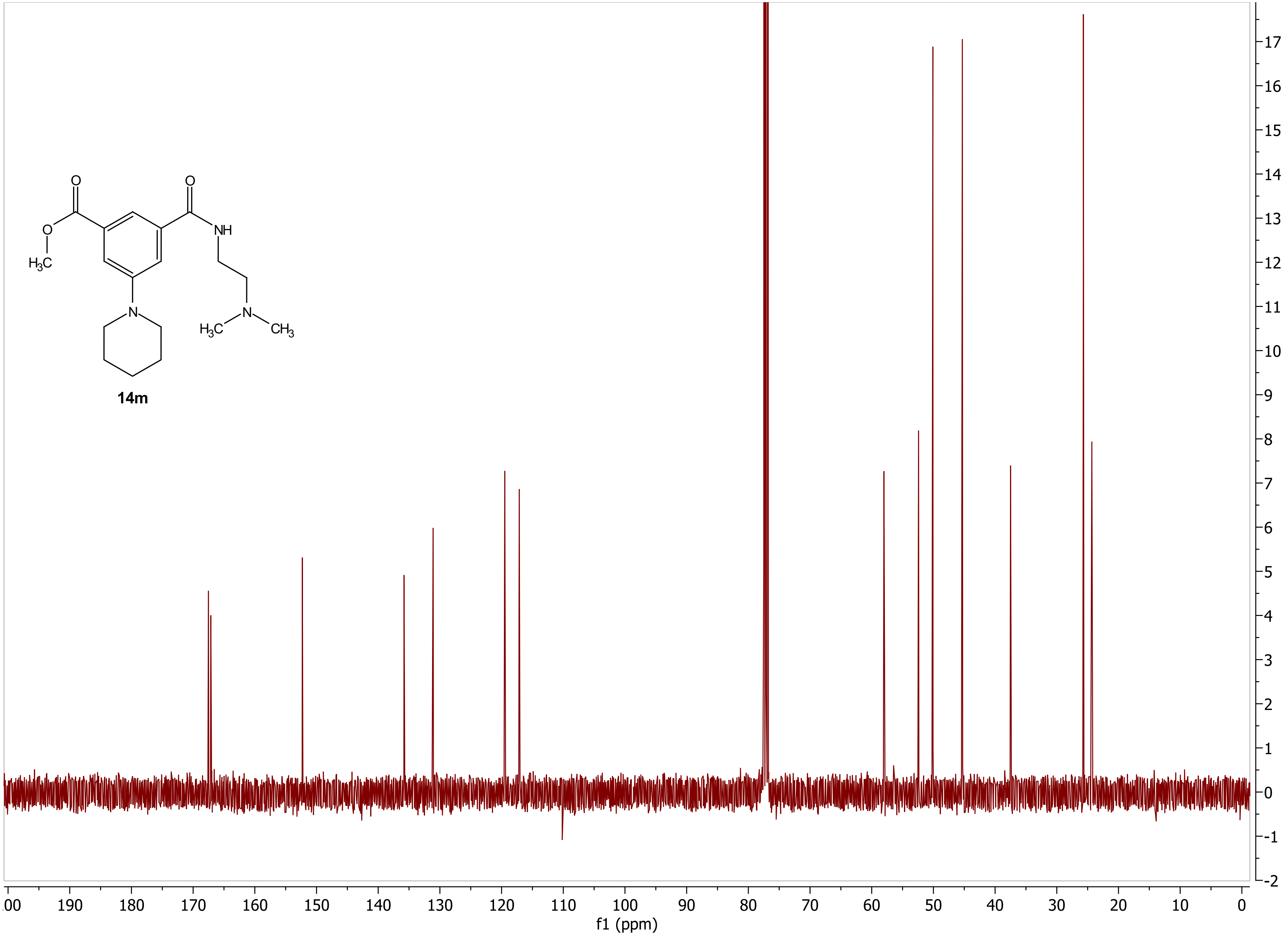

O
O
NH
O
H_3C
N
H_3C
N
CH_3
14m
f1 (ppm)

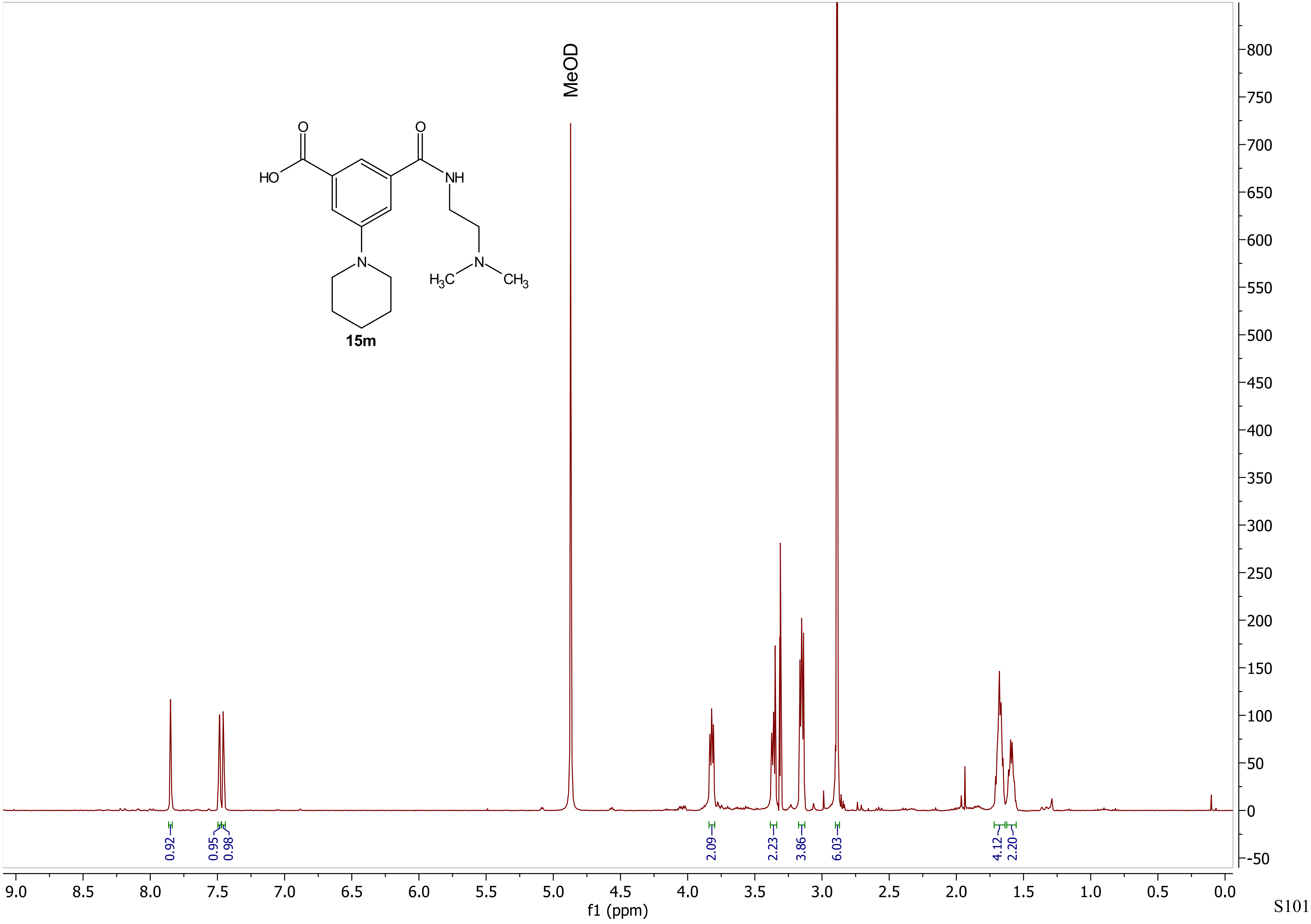
MeOD
HO
O
O
NH
N
H3C
N
CH3
15m
0.92
0.95
0.98
2.09
2.23
3.86
6.03
4.12
2.20
f1 (ppm)
9.0
8.5
8.0
7.5
7.0
6.5
6.0
5.5
5.0
4.5
4.0
3.5
3.0
2.5
2.0
1.5
1.0
0.5
0.0
800
750
700
650
600
550
500
450
400
350
300
250
200
150
100
50
0
-50

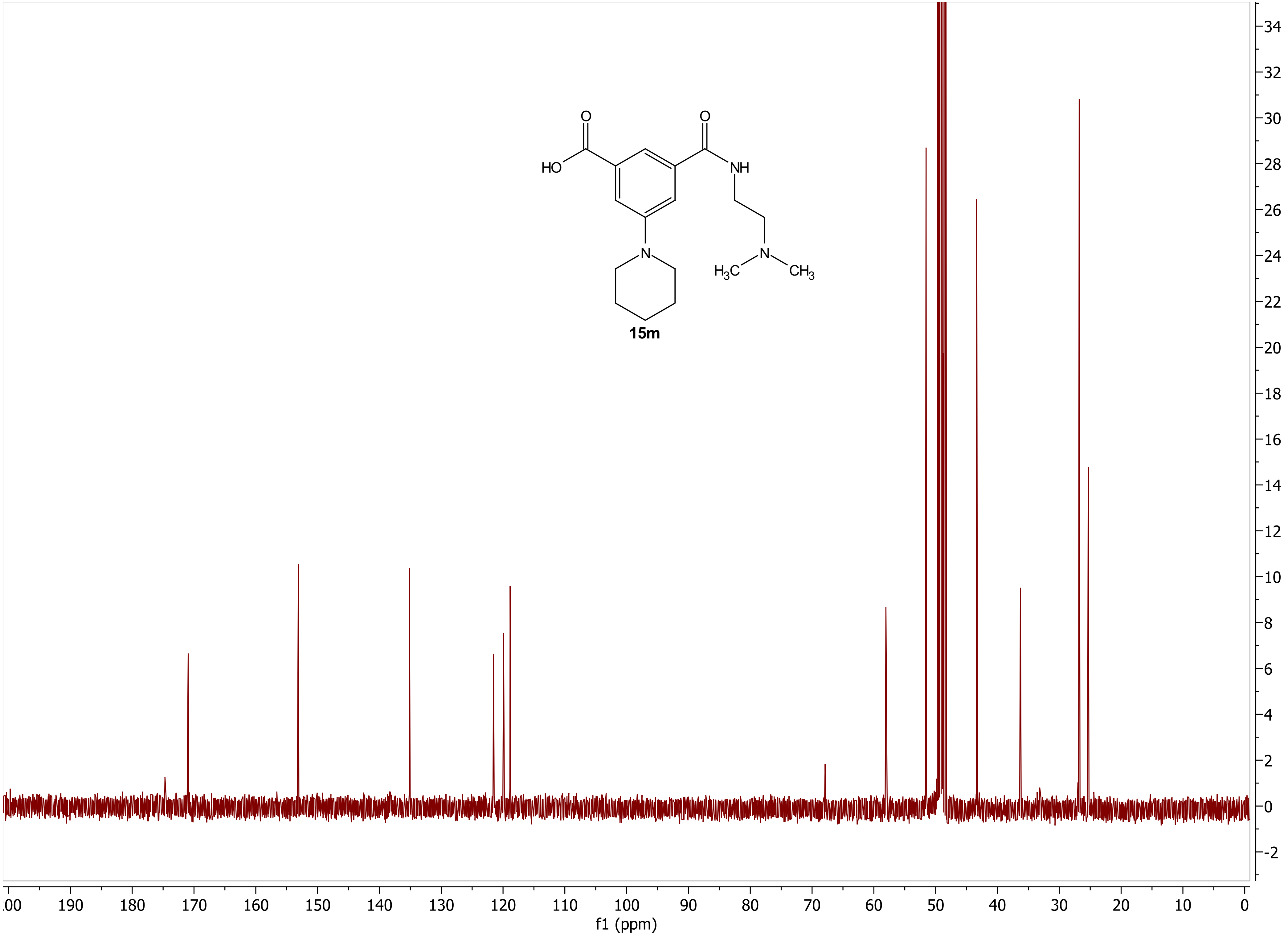
HO
O
O
NH
N
H3C
N
CH3
15m
f1 (ppm)

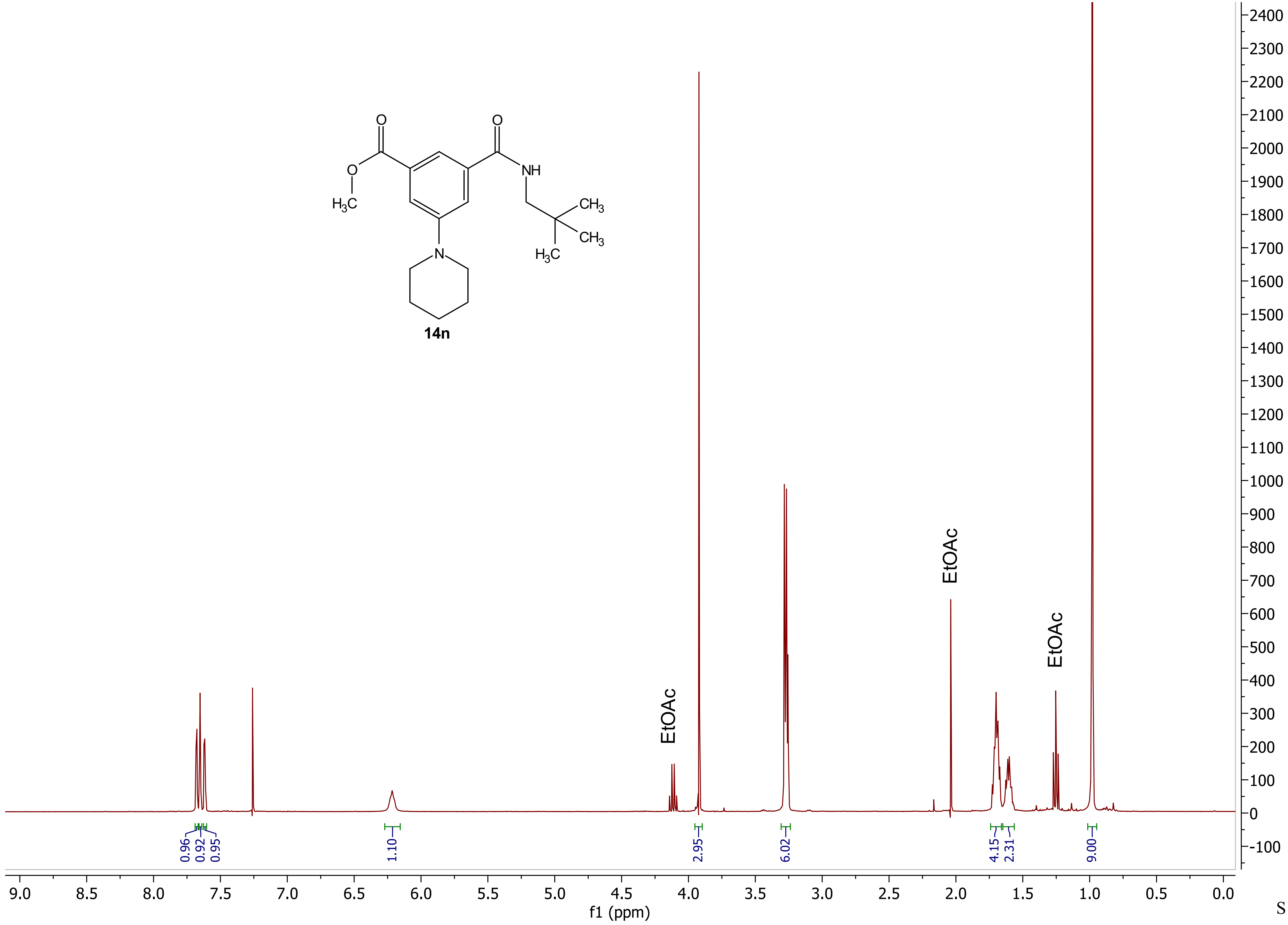
14n
EtOAc
EtOAc
EtOAc
0.96
0.92
0.95
1.10
2.95
6.02
4.15
2.31
9.00
f1 (ppm)

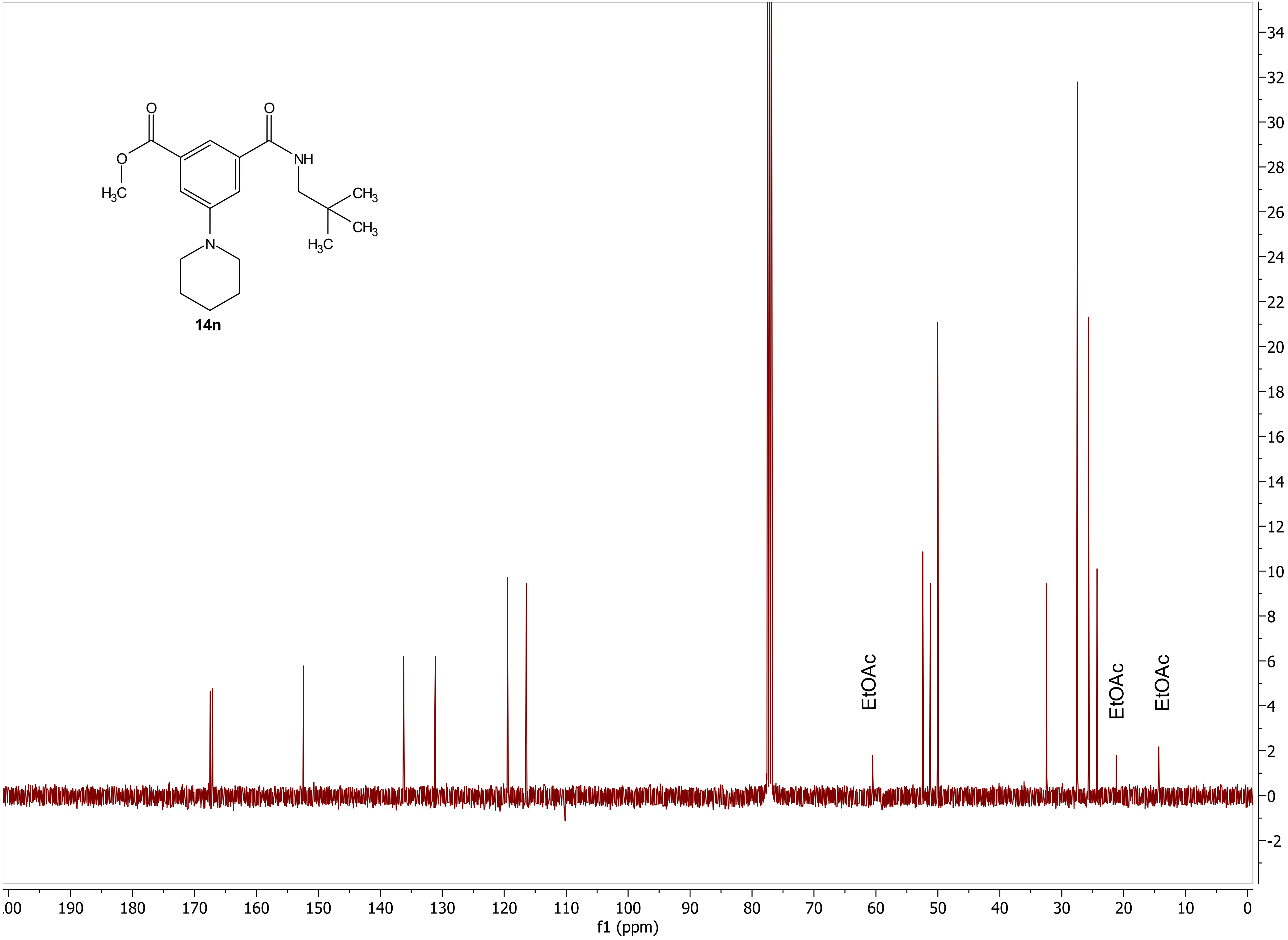

O
O
NH
H3C
O
CH3
CH3
H3C
N
14n
EtOAc
EtOAc
EtOAc
f1 (ppm)

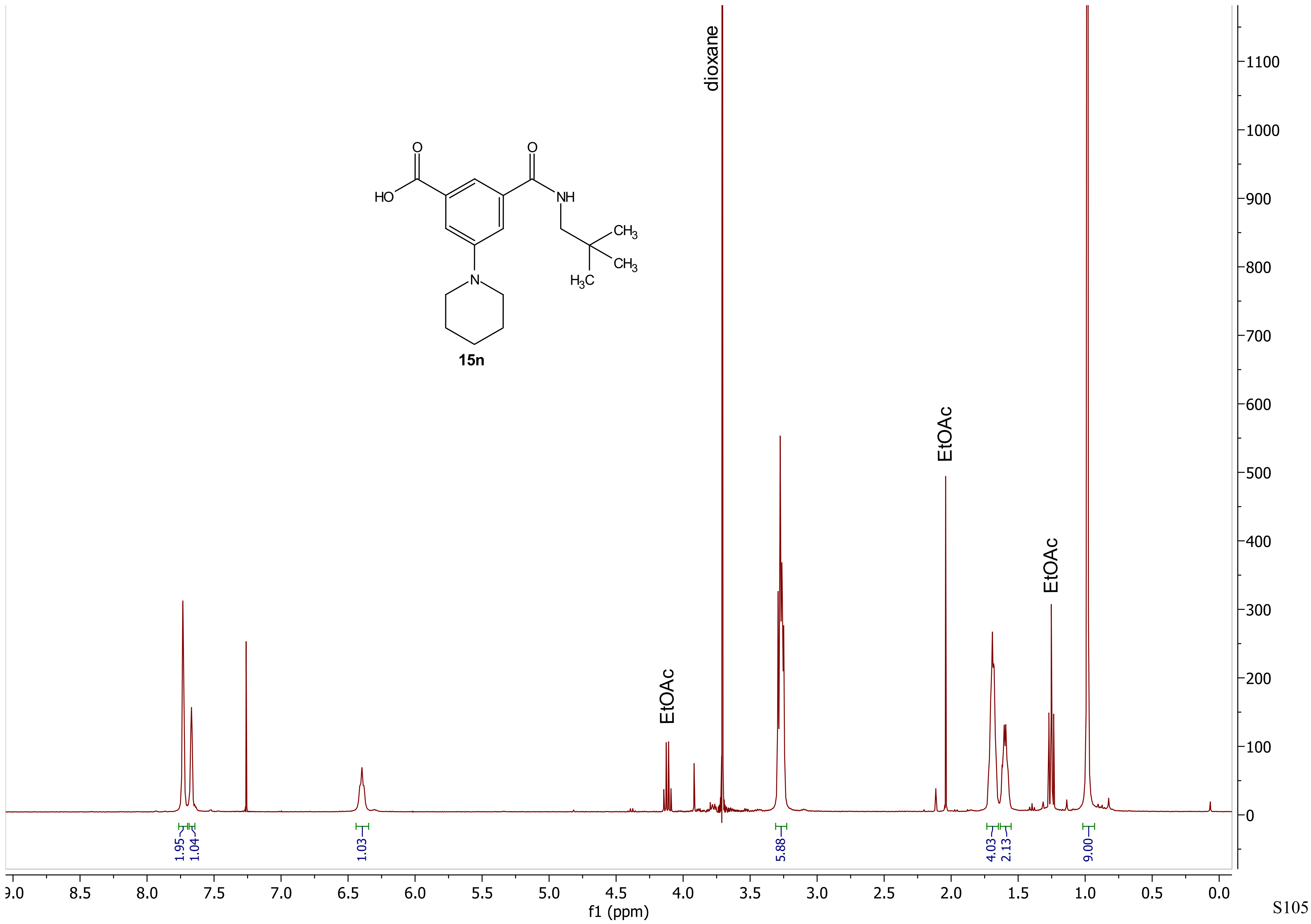
15n
HO
O
NH
CH3
H3C
N
dioxane
EtOAc
EtOAc
EtOAc
1.95
1.04
1.03
5.88
4.03
2.13
9.00
f1 (ppm)

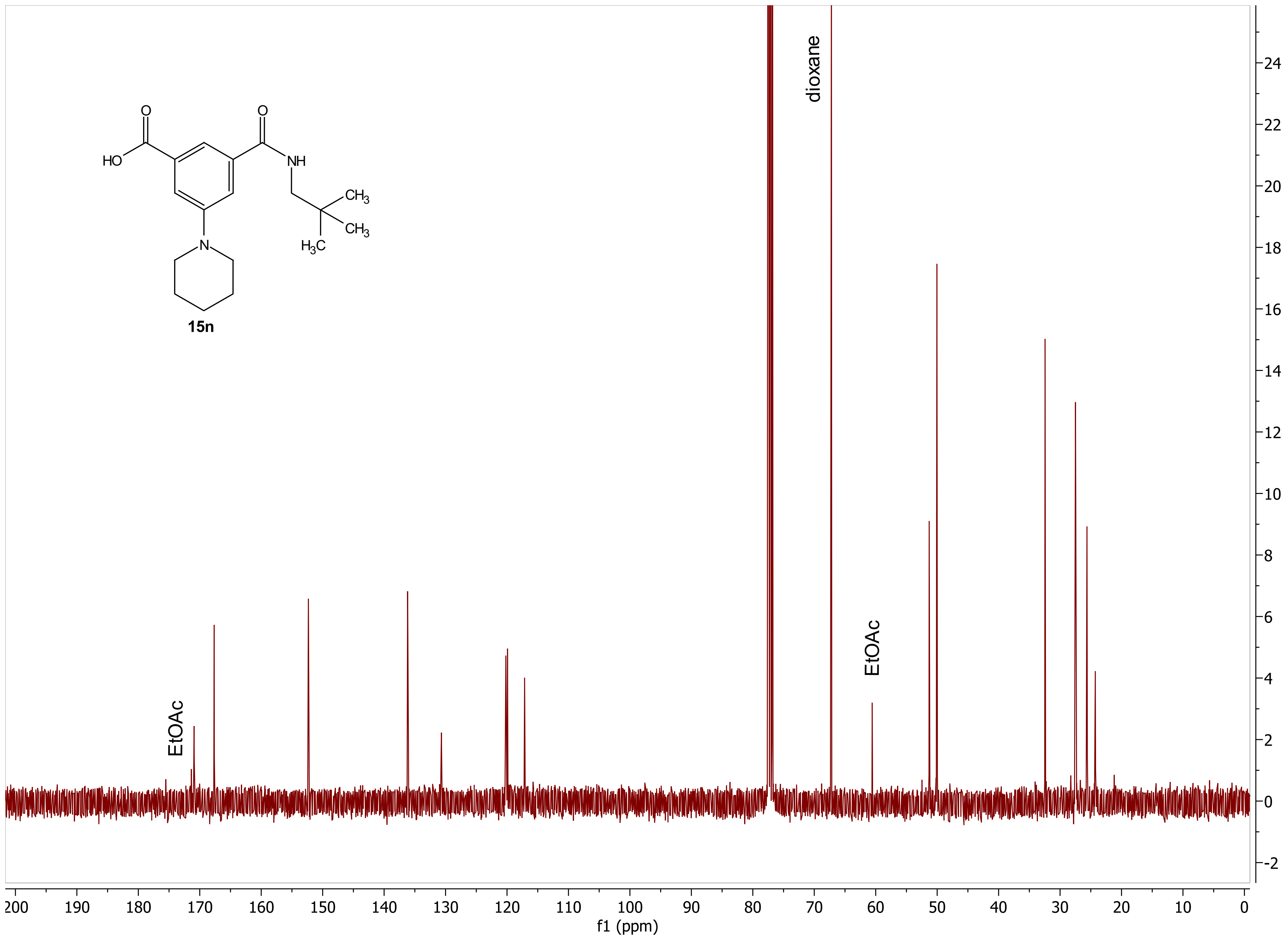
HO
O
O
NH
CH3
CH3
H3C
N
15n
dioxane
EtOAc
EtOAc
f1 (ppm)

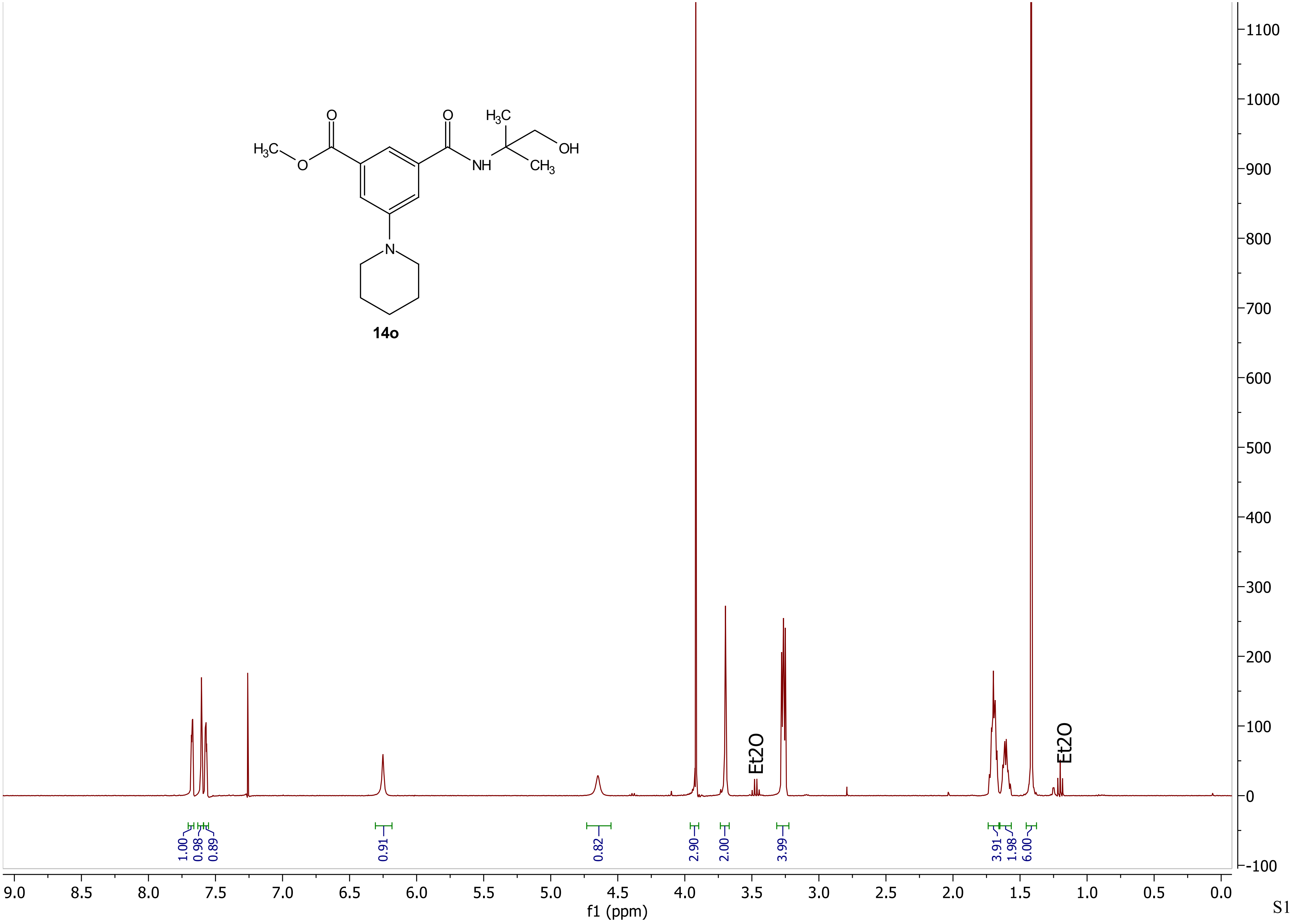

14o
Et2O
Et2O
1.00
0.98
0.89
0.91
0.82
2.90
2.00
3.99
3.91
1.98
6.00
f1 (ppm)

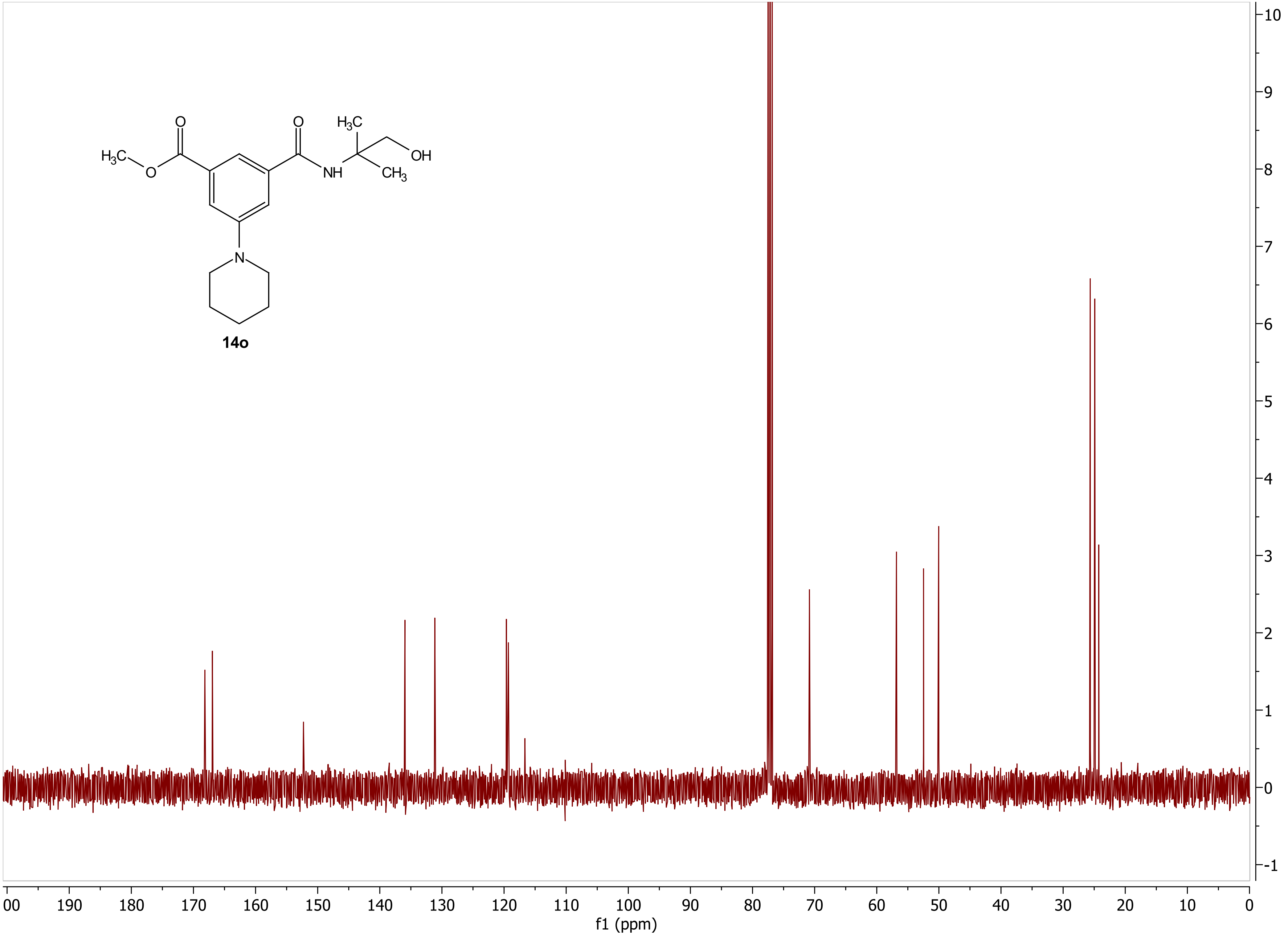
H3C
O
O
O
NH
H3C
CH3
OH
N
14o
f1 (ppm)
00
190
180
170
160
150
140
130
120
110
100
90
80
70
60
50
40
30
20
10
0
10
9
8
7
6
5
4
3
2
1
0
-1

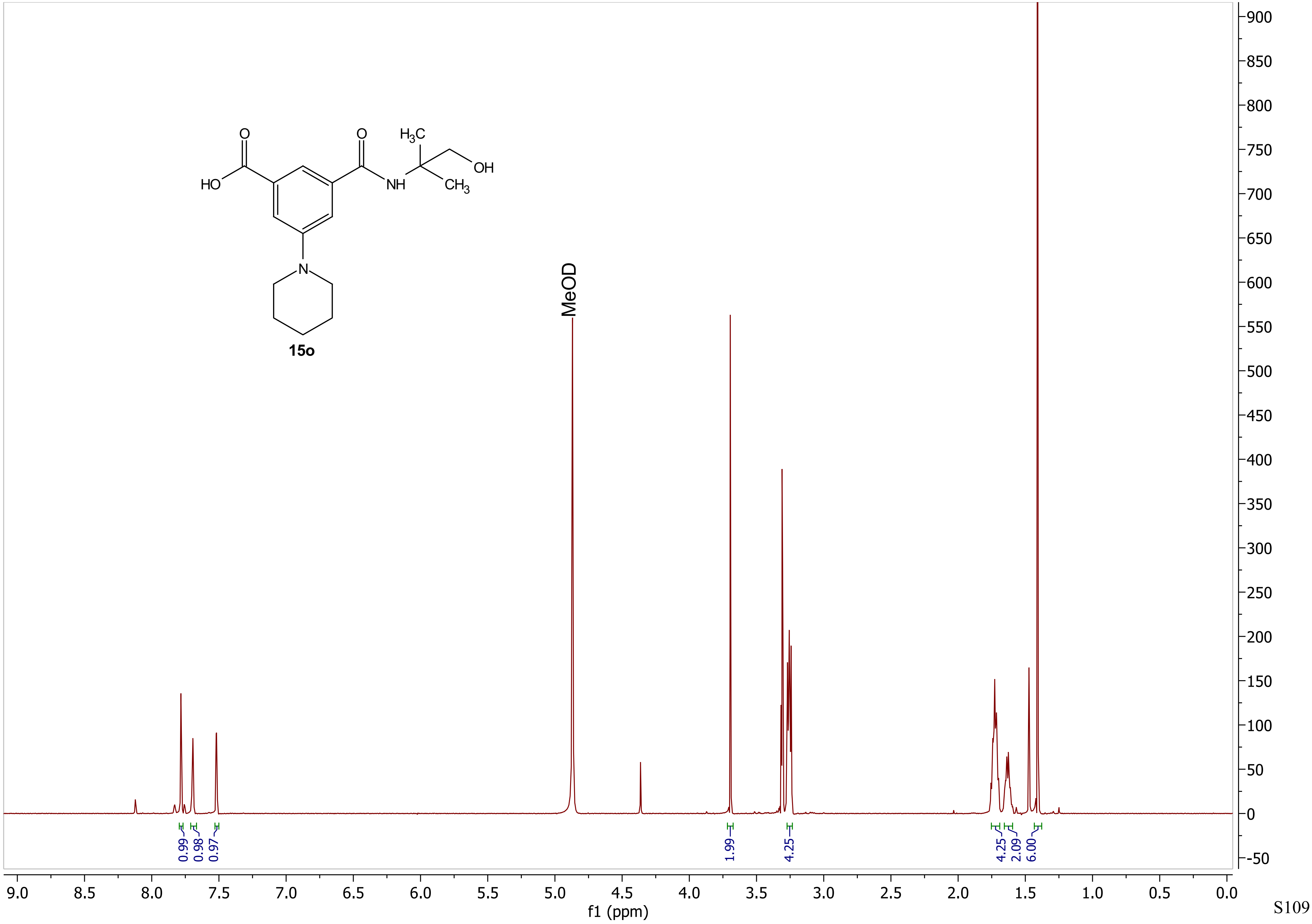

O
O
H3C
HO
NH
OH
CH3
N
15o
MeOD
0.99
0.98
0.97
1.99
4.25
4.25
2.09
6.00
9.0 8.5 8.0 7.5 7.0 6.5 6.0 5.5 5.0 4.5 4.0 3.5 3.0 2.5 2.0 1.5 1.0 0.5 0.0
f1 (ppm)
900 850 800 750 700 650 600 550 500 450 400 350 300 250 200 150 100 50 0 -50

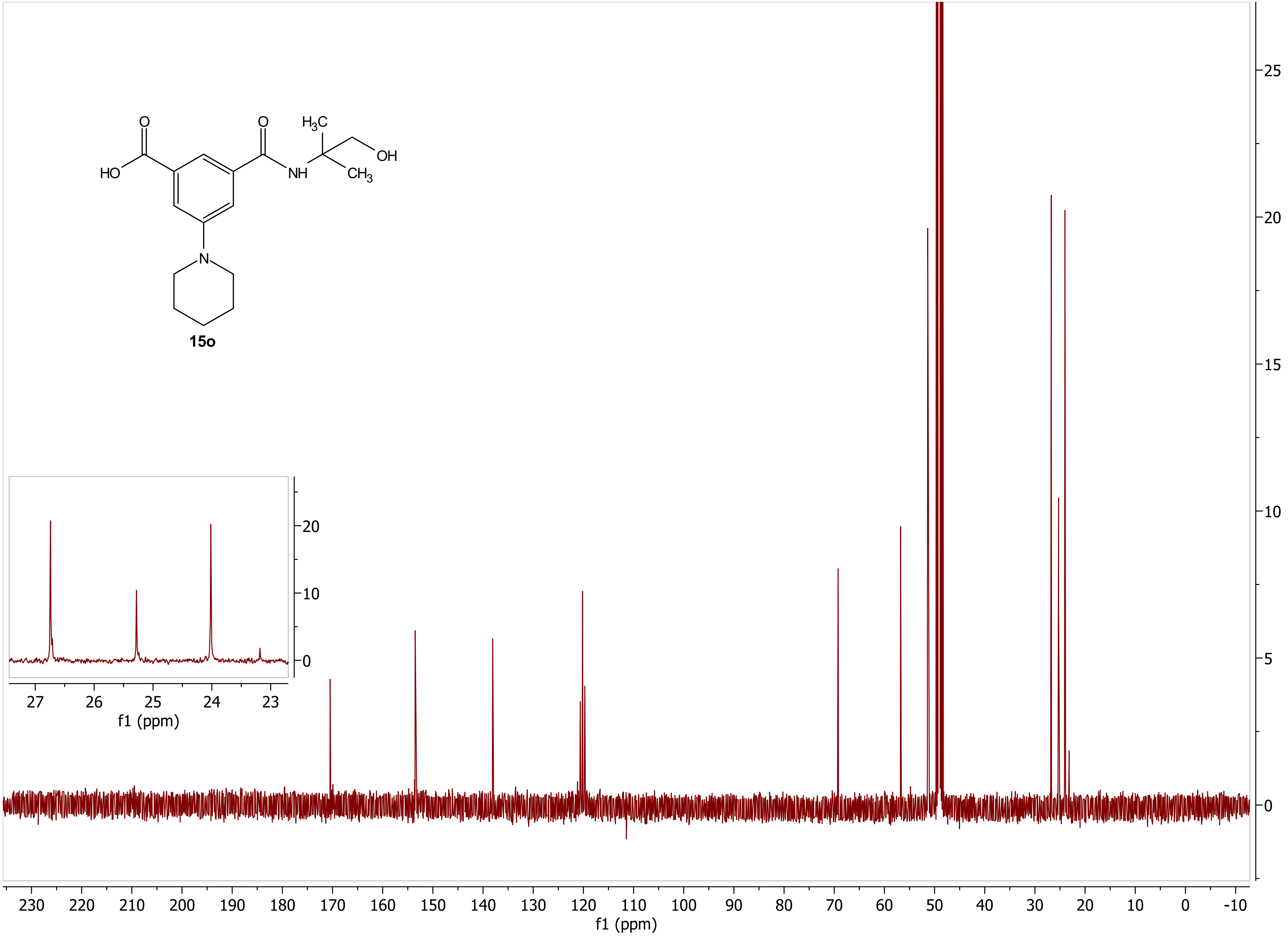

HO
O
O
NH
H_3C
OH
CH_3
N
15o
27
26
25
24
23
f1 (ppm)
20
10
0
230
220
210
200
190
180
170
160
150
140
130
120
110
100
90
80
70
60
50
40
30
20
10
0
-10
f1 (ppm)
25
20
15
10
5
0

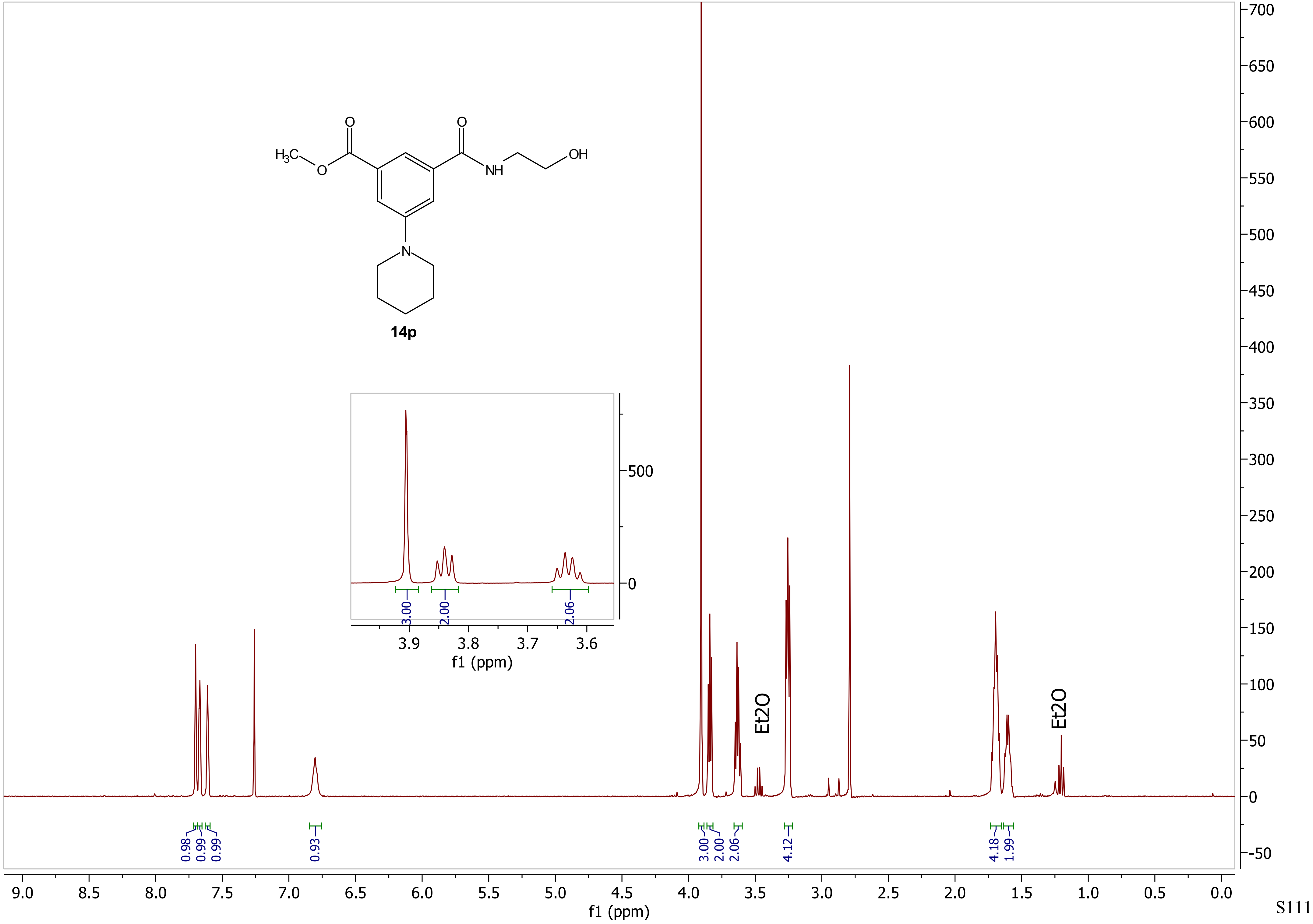

H3C
O
O
O
NH
OH
N
14p
3.00
2.00
2.06
3.9
3.8
3.7
3.6
f1 (ppm)
500
0
Et2O
Et2O
0.98
0.99
0.99
0.93
3.00
2.00
2.06
4.12
4.18
1.99
9.0
8.5
8.0
7.5
7.0
6.5
6.0
5.5
5.0
4.5
4.0
3.5
3.0
2.5
2.0
1.5
1.0
0.5
0.0
f1 (ppm)
700
650
600
550
500
450
400
350
300
250
200
150
100
50
0
-50

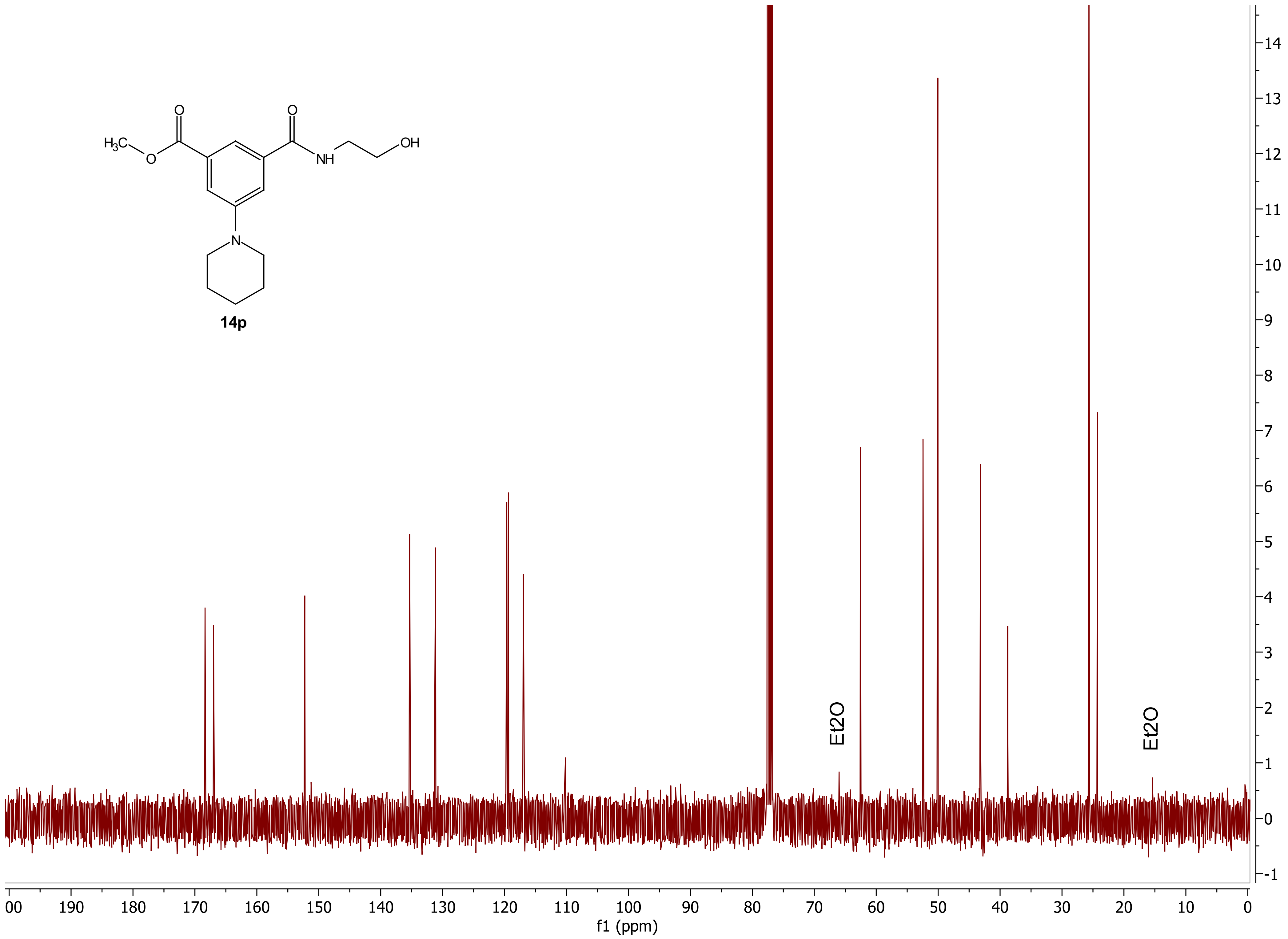
H3C
O
O
O
NH
OH
N
14p
Et2O
Et2O
f1 (ppm)
00 190 180 170 160 150 140 130 120 110 100 90 80 70 60 50 40 30 20 10 0
14 13 12 11 10 9 8 7 6 5 4 3 2 1 0 -1

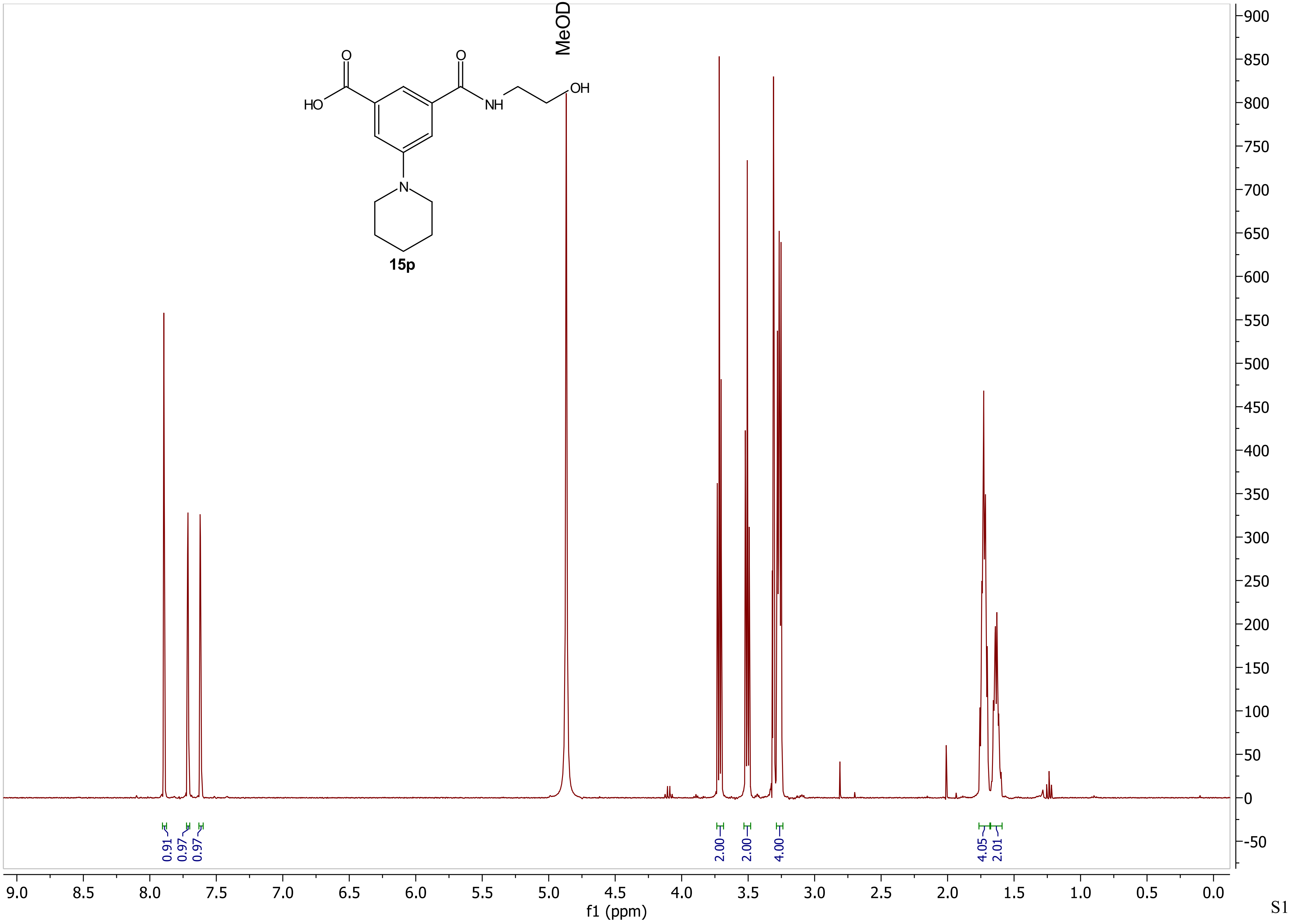

MeOD
HO
O
O
NH
OH
N
15p
0.91
0.97
0.97
2.00
2.00
4.00
4.05
2.01
9.0 8.5 8.0 7.5 7.0 6.5 6.0 5.5 5.0 4.5 4.0 3.5 3.0 2.5 2.0 1.5 1.0 0.5 0.0
f1 (ppm)
900 850 800 750 700 650 600 550 500 450 400 350 300 250 200 150 100 50 0 -50

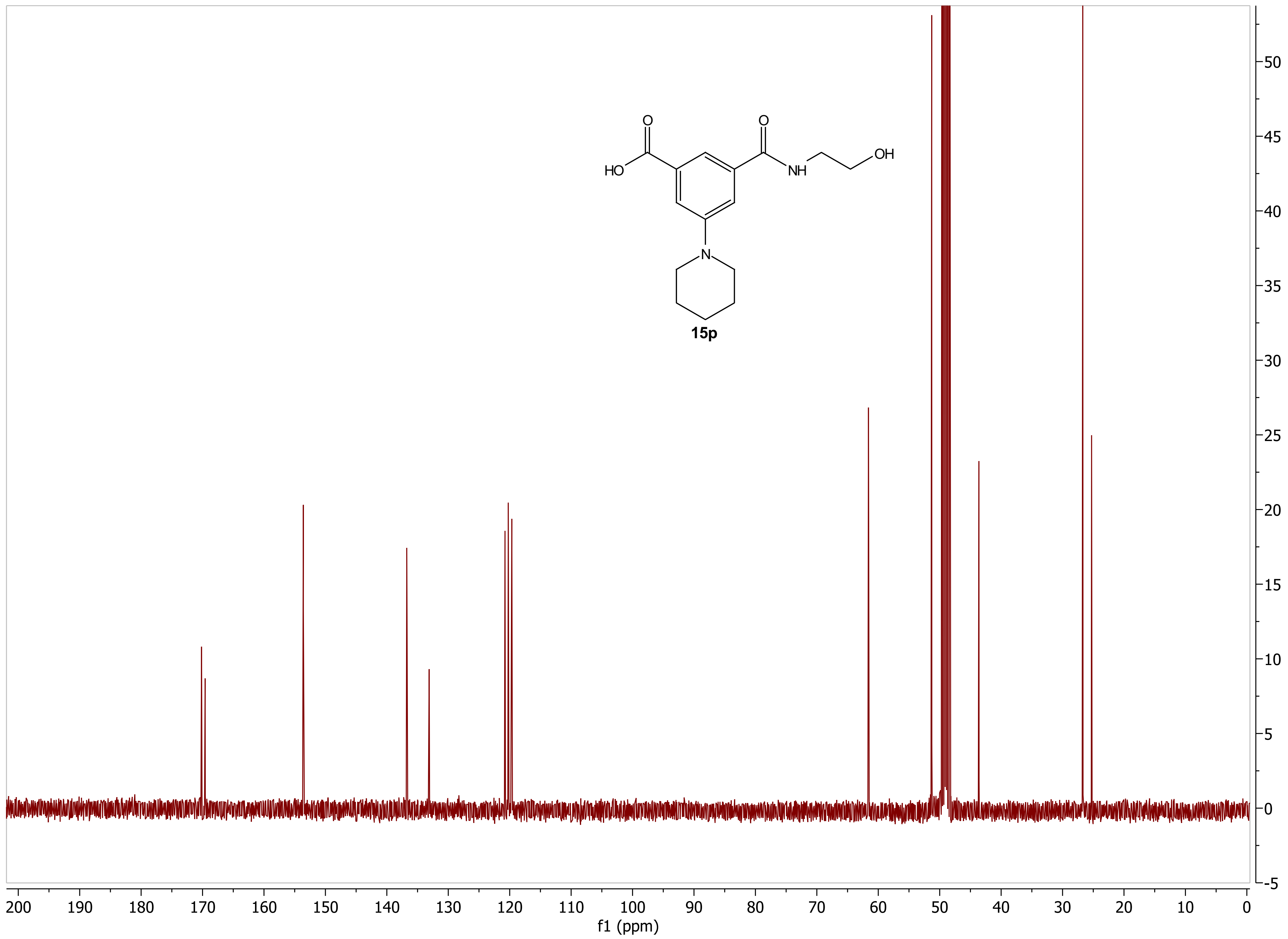

HO
O
O
NH
OH
N
15p
f1 (ppm)

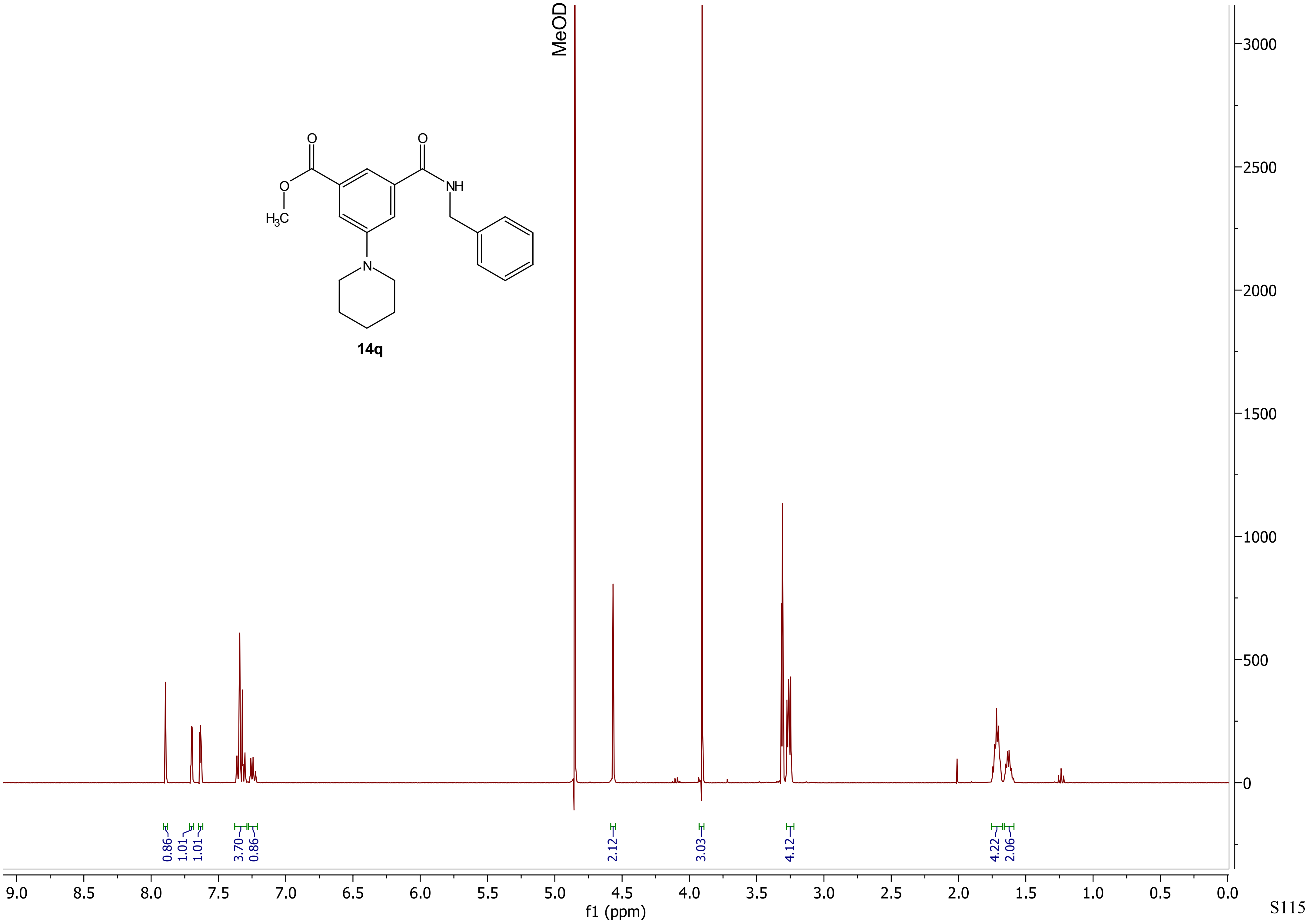
O
O
NH
O
H3C
N
MeOD
3000
2500
2000
1500
1000
500
0
0.86
1.01
1.01
3.70
0.86
2.12
3.03
4.12
4.22
2.06
9.0
8.5
8.0
7.5
7.0
6.5
6.0
5.5
5.0
4.5
4.0
3.5
3.0
2.5
2.0
1.5
1.0
0.5
0.0
f1 (ppm)

14q

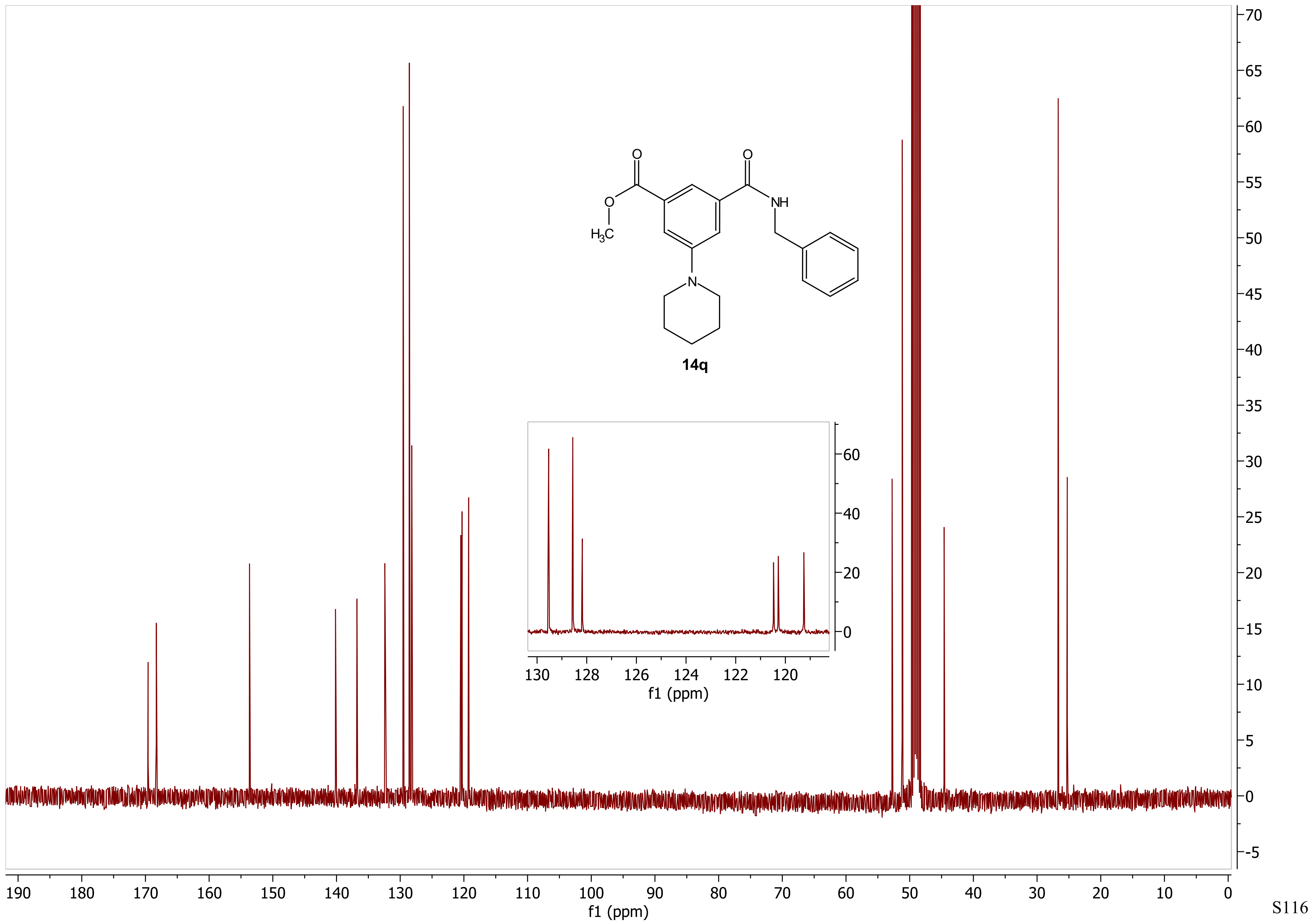

O
O
NH
O
H3C
N
14q
130
128
126
124
122
120
f1 (ppm)
190
180
170
160
150
140
130
120
110
100
90
80
70
60
50
40
30
20
10
0
f1 (ppm)

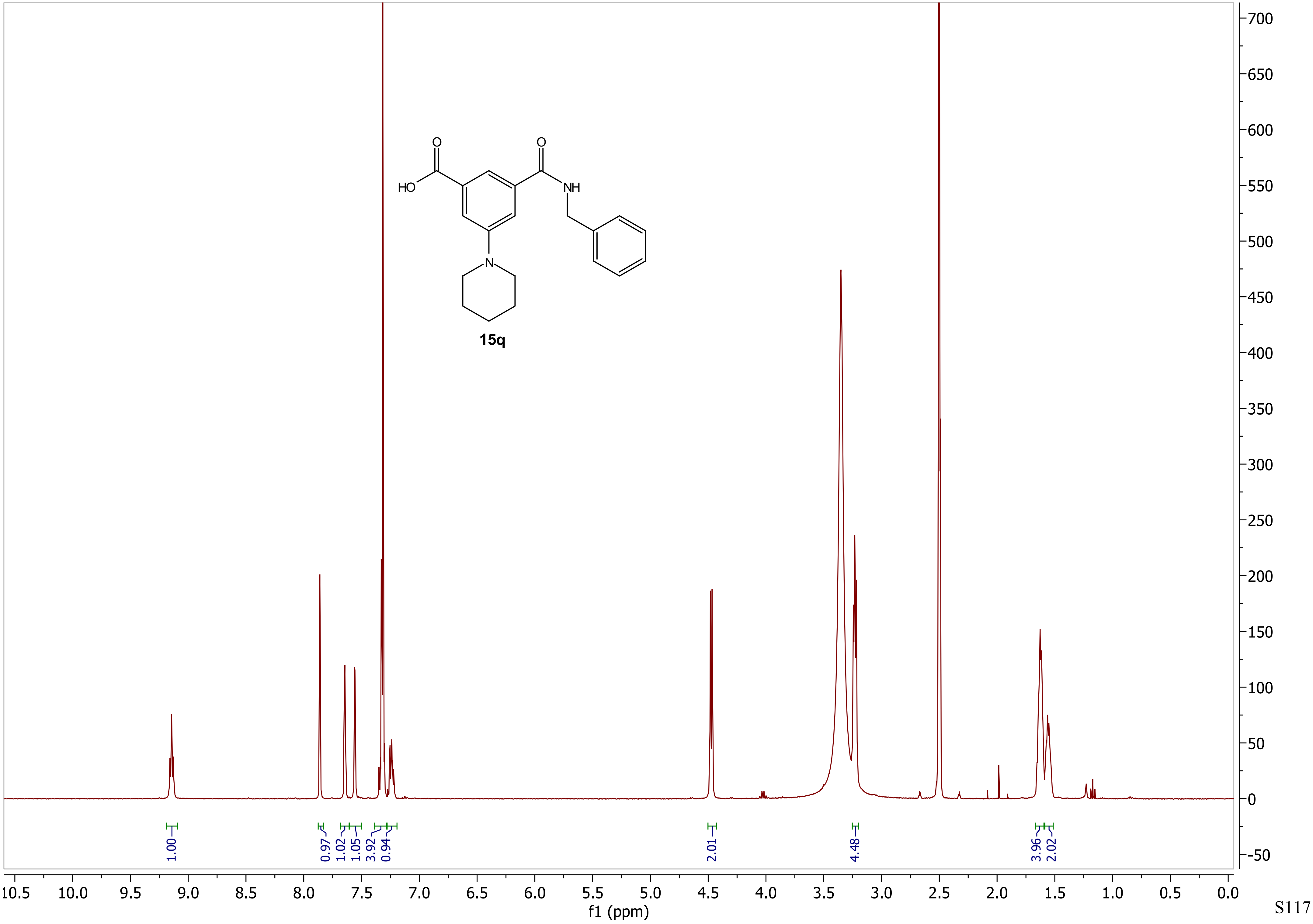

O
O
HO
NH
N
15q
1.00
0.97
1.02
1.05
3.92
0.94
2.01
4.48
3.96
2.02
10.5 10.0 9.5 9.0 8.5 8.0 7.5 7.0 6.5 6.0 5.5 5.0 4.5 4.0 3.5 3.0 2.5 2.0 1.5 1.0 0.5 0.0
f1 (ppm)
700 650 600 550 500 450 400 350 300 250 200 150 100 50 0 -50

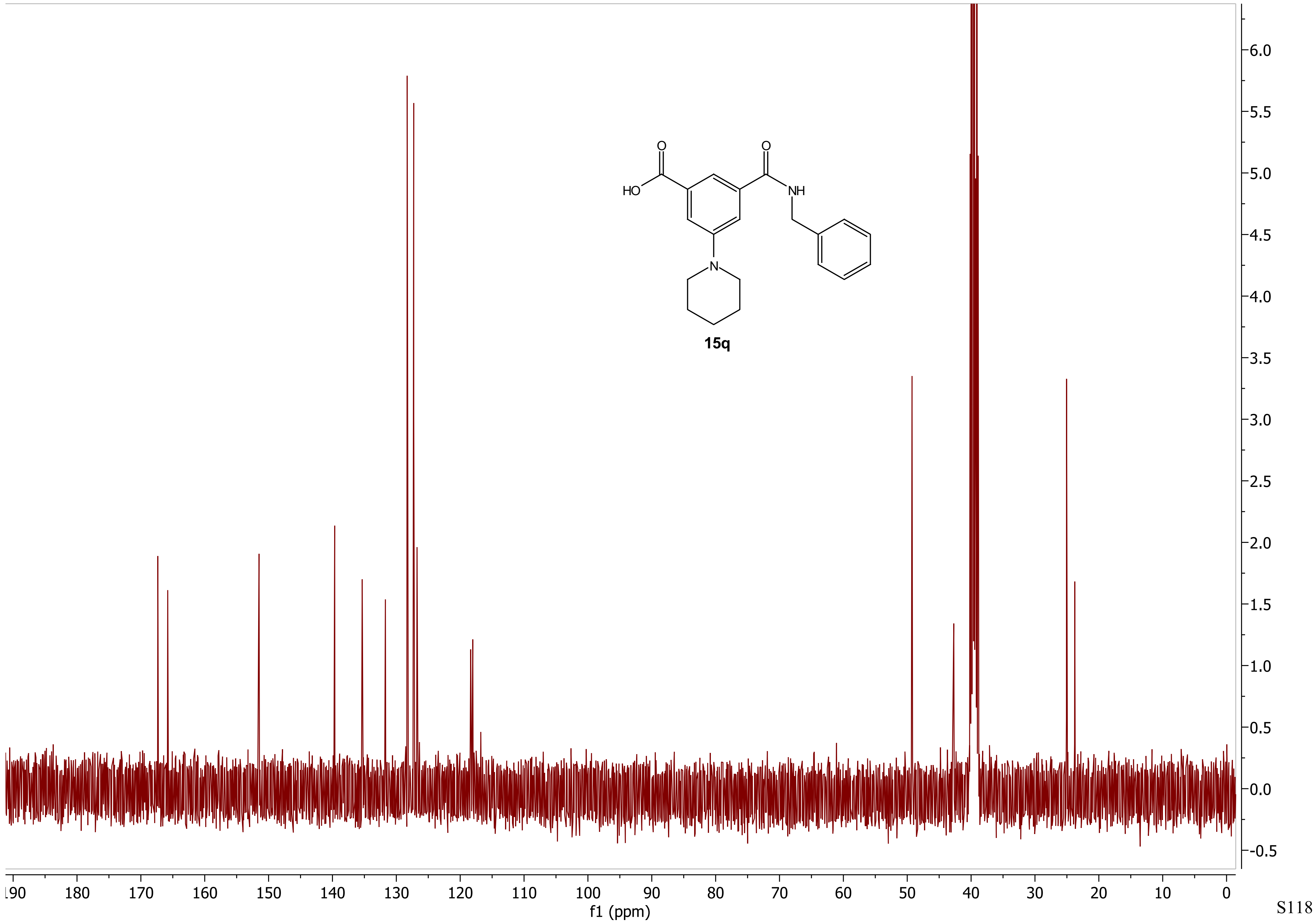

HO
O
O
NH
N
15q
f1 (ppm)

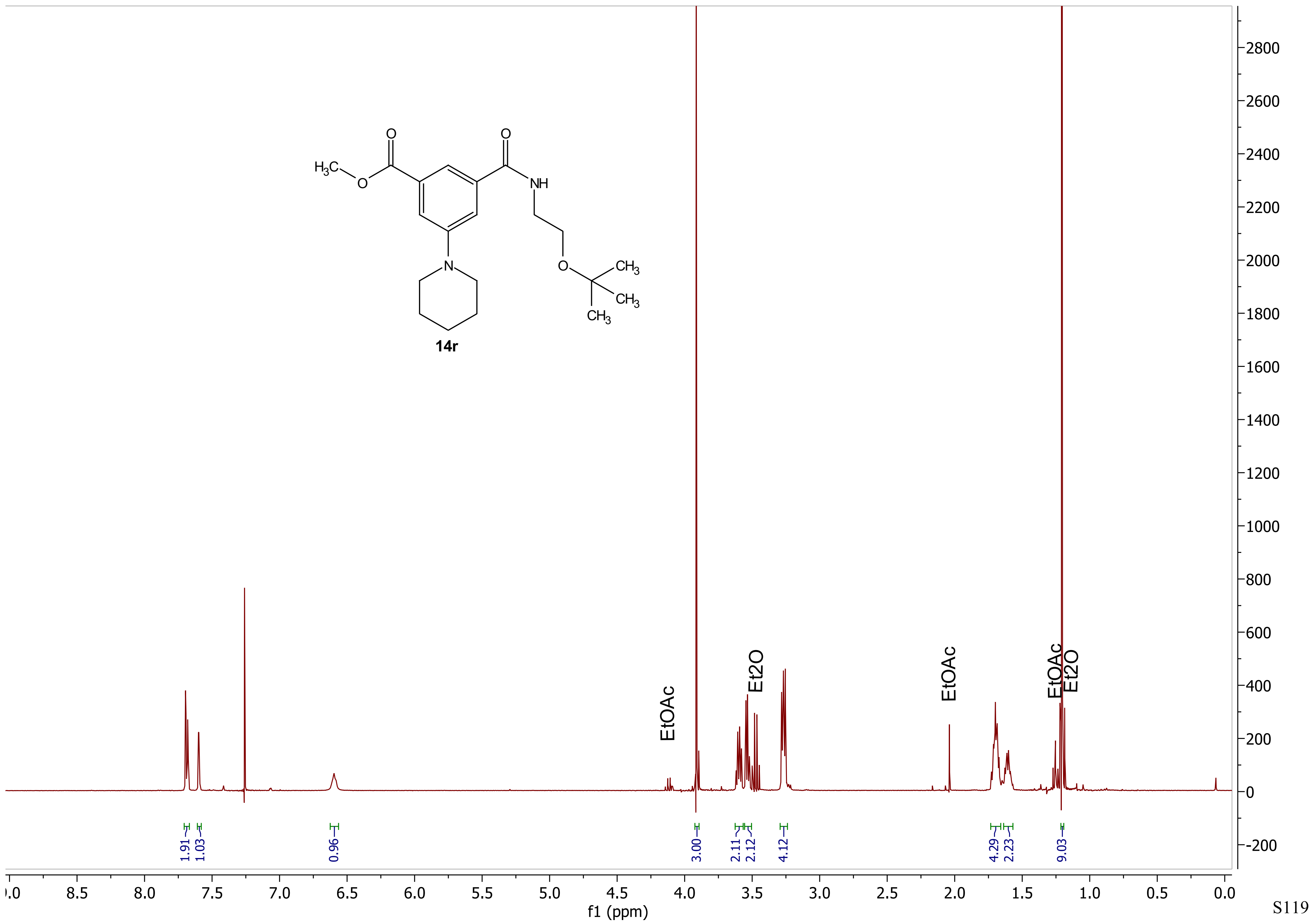

H3C
O
O
O
NH
N
O
CH3
CH3
CH3
14r
EtOAc
Et2O
EtOAc
EtOAc
Et2O
1.91
1.03
0.96
3.00
2.11
2.12
4.12
4.29
2.23
9.03
f1 (ppm)

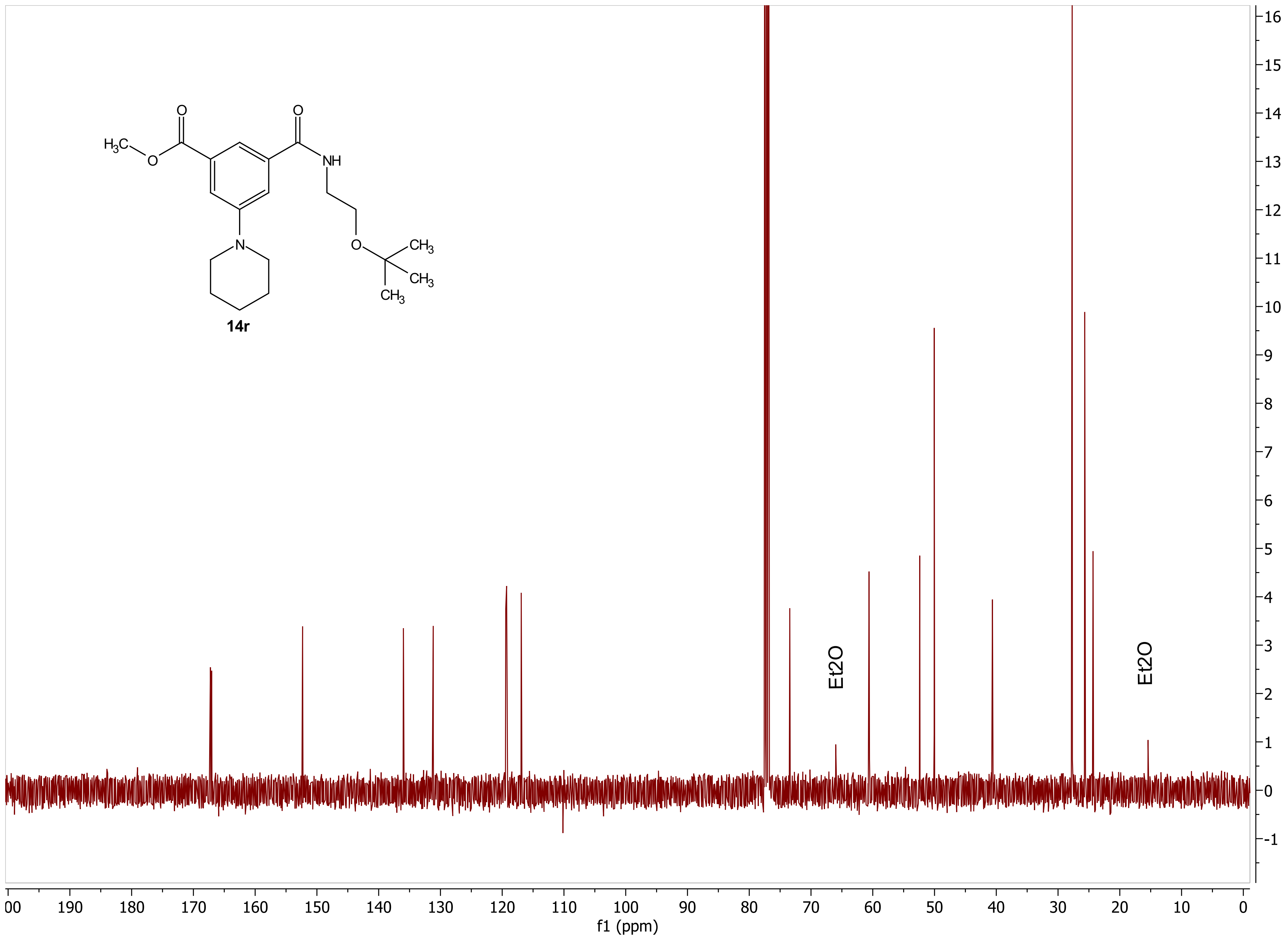
H3C
O
O
O
NH
N
O
CH3
CH3
CH3
14r
Et2O
Et2O
00 190 180 170 160 150 140 130 120 110 100 90 80 70 60 50 40 30 20 10 0
f1 (ppm)
16 15 14 13 12 11 10 9 8 7 6 5 4 3 2 1 0 -1

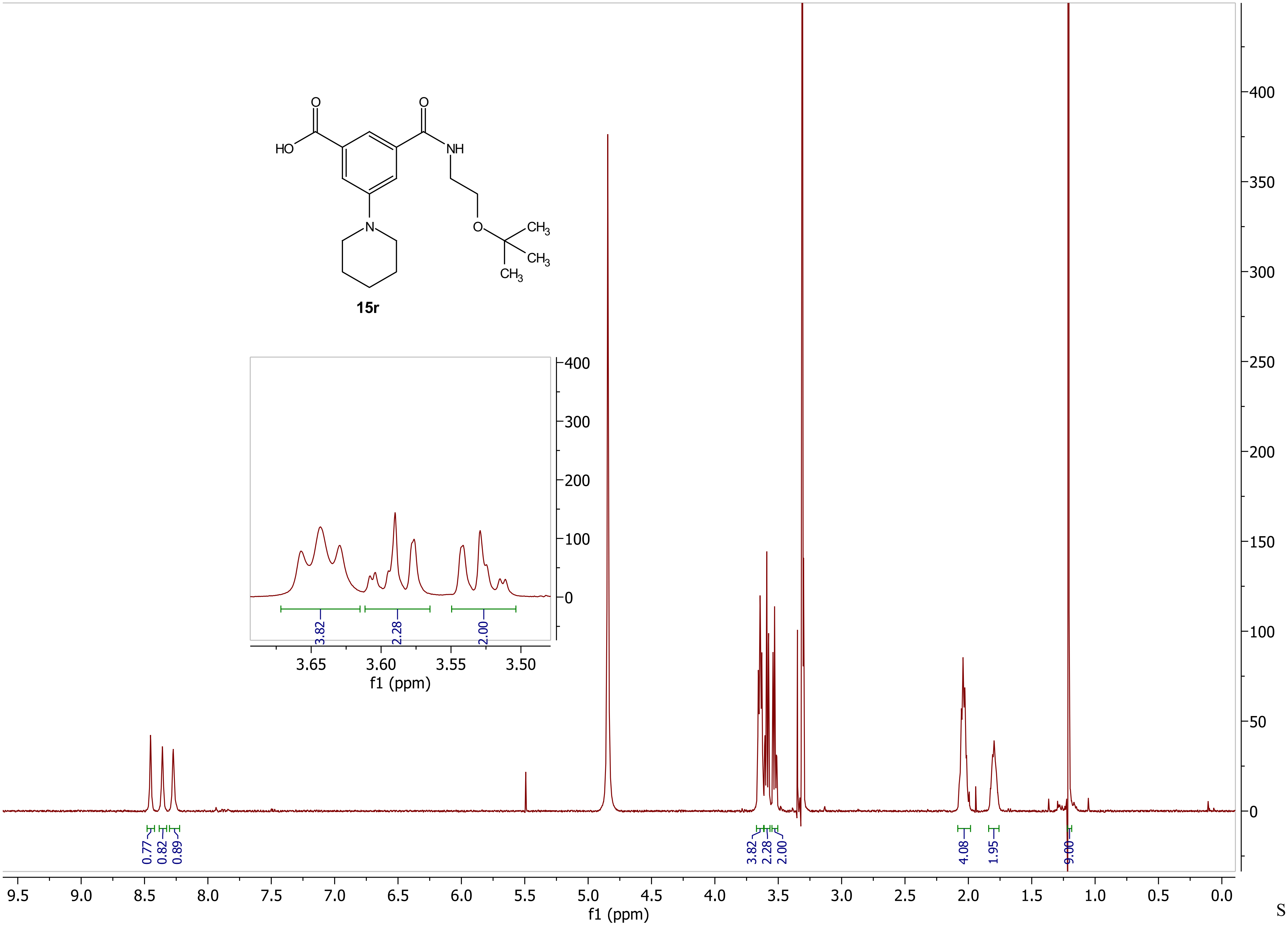

HO
O
NH
N
O
CH3
CH3
CH3
15r
3.82
2.28
2.00
3.65
3.60
3.55
3.50
f1 (ppm)
400
300
200
100
0
0.77
0.82
0.89
4.08
1.95
9.00
9.5
9.0
8.5
8.0
7.5
7.0
6.5
6.0
5.5
5.0
4.5
4.0
3.5
3.0
2.5
2.0
1.5
1.0
0.5
0.0
f1 (ppm)
400
350
300
250
200
150
100
50
0

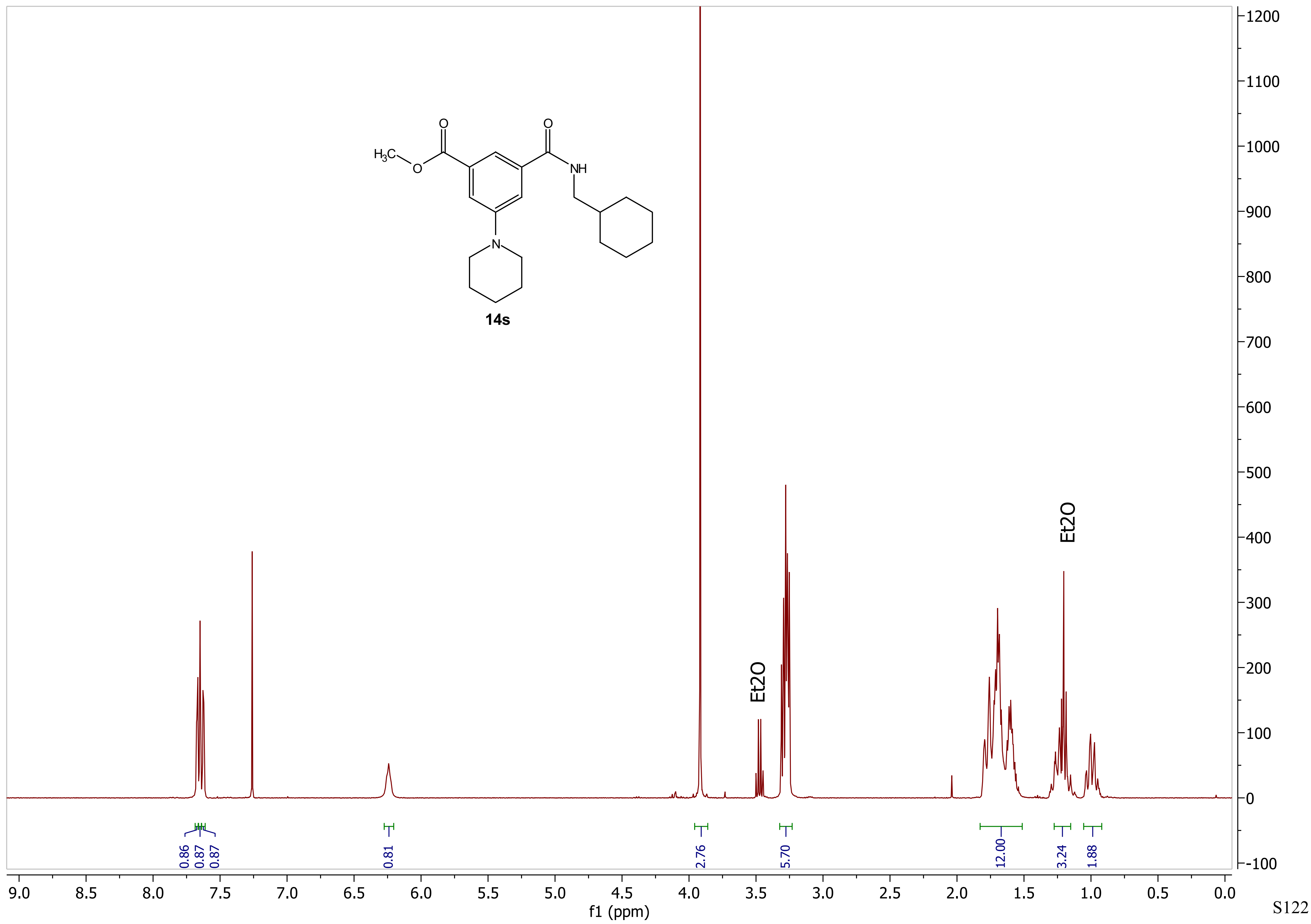

14s
Et2O
Et2O
0.86
0.87
0.87
0.81
2.76
5.70
12.00
3.24
1.88
f1 (ppm)

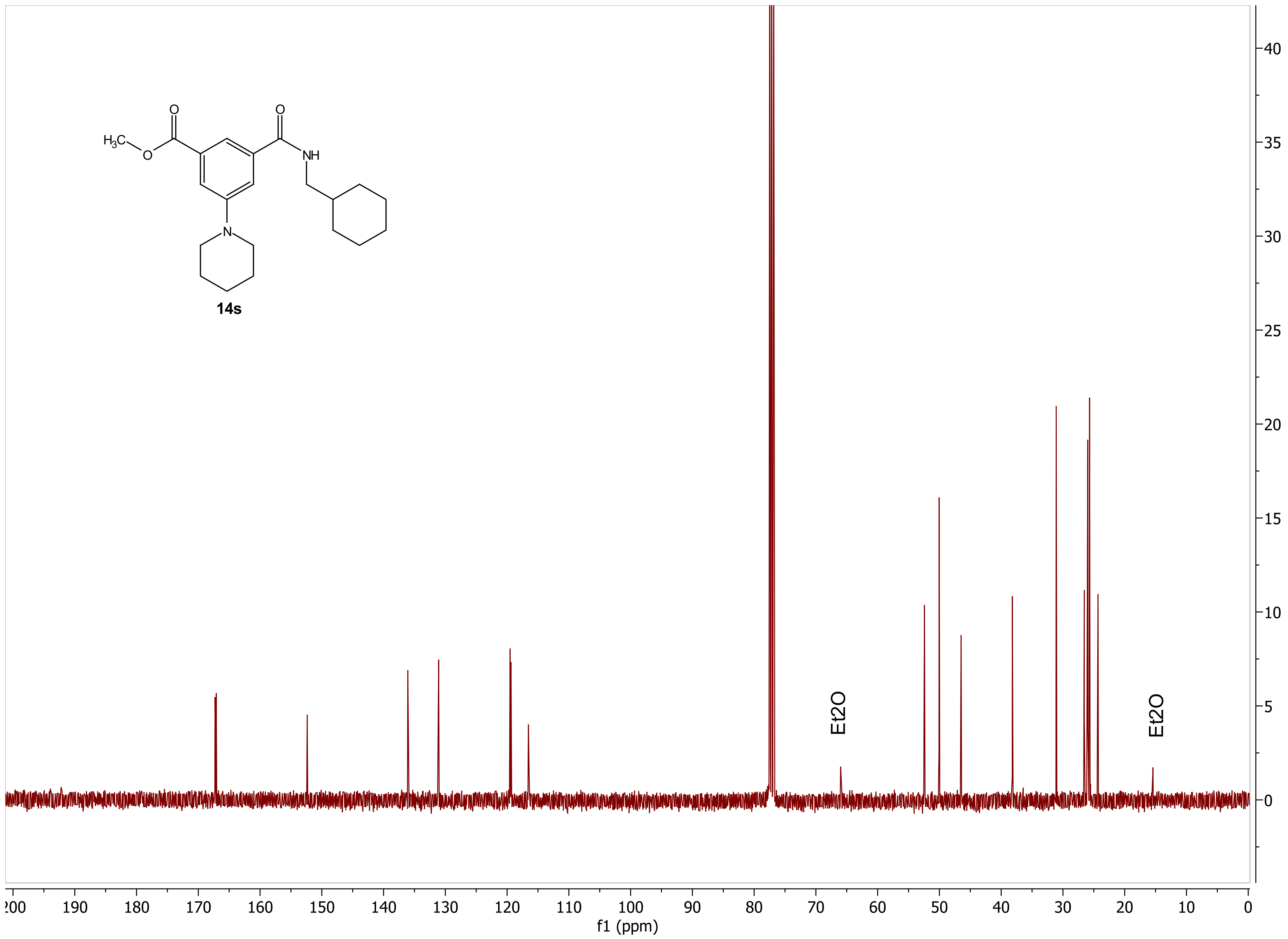
H3C
O
O
O
NH
N
14s
Et2O
Et2O
f1 (ppm)
200 190 180 170 160 150 140 130 120 110 100 90 80 70 60 50 40 30 20 10 0
40 35 30 25 20 15 10 5 0

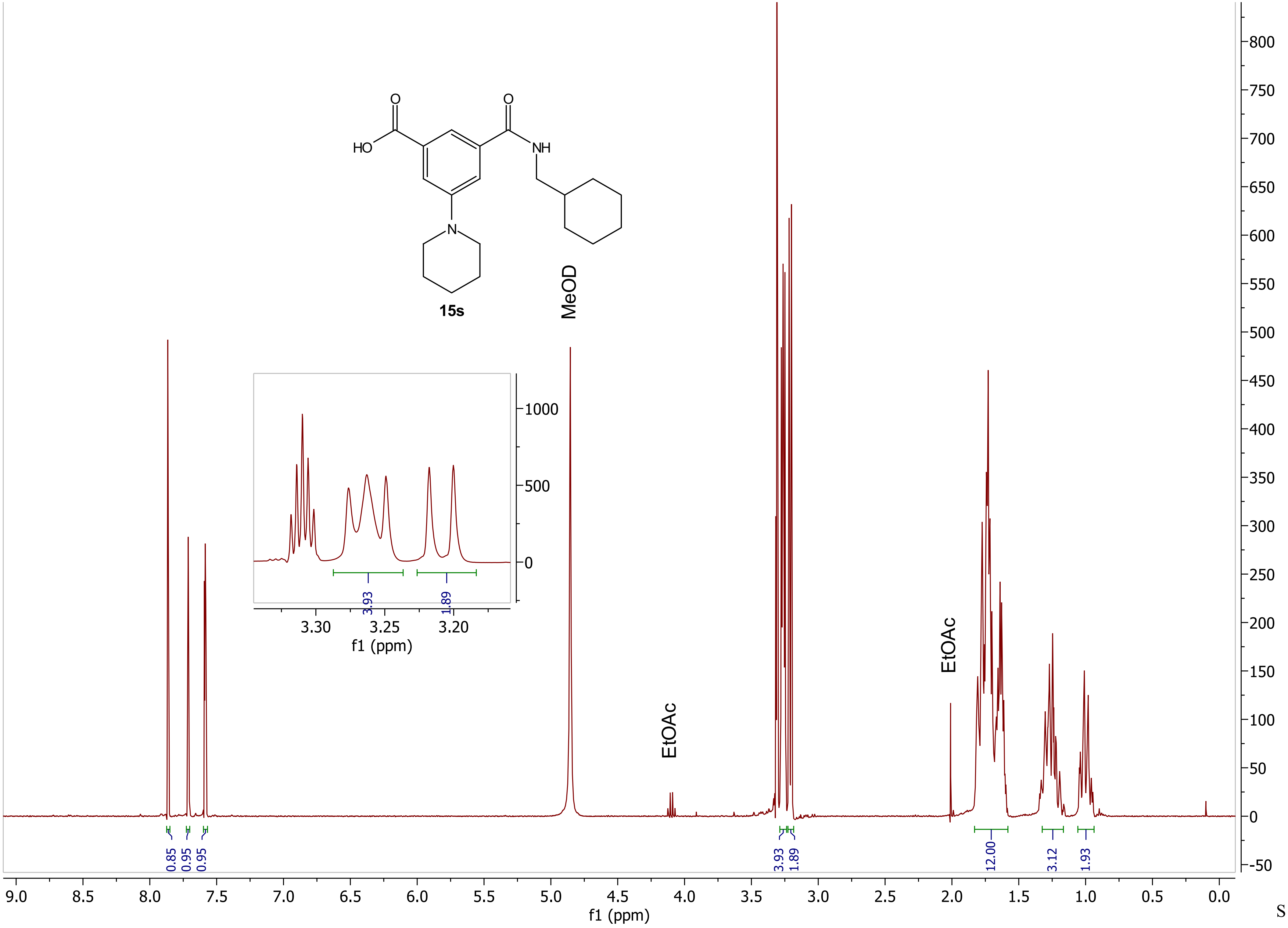
HO
O
O
NH
N
15s
MeOD
EtOAc
EtOAc
3.93
1.89
3.30
3.25
3.20
f1 (ppm)
1000
500
0
0.85
0.95
0.95
3.93
1.89
12.00
3.12
1.93
9.0
8.5
8.0
7.5
7.0
6.5
6.0
5.5
5.0
4.5
4.0
3.5
3.0
2.5
2.0
1.5
1.0
0.5
0.0
f1 (ppm)
800
750
700
650
600
550
500
450
400
350
300
250
200
150
100
50
0
-50

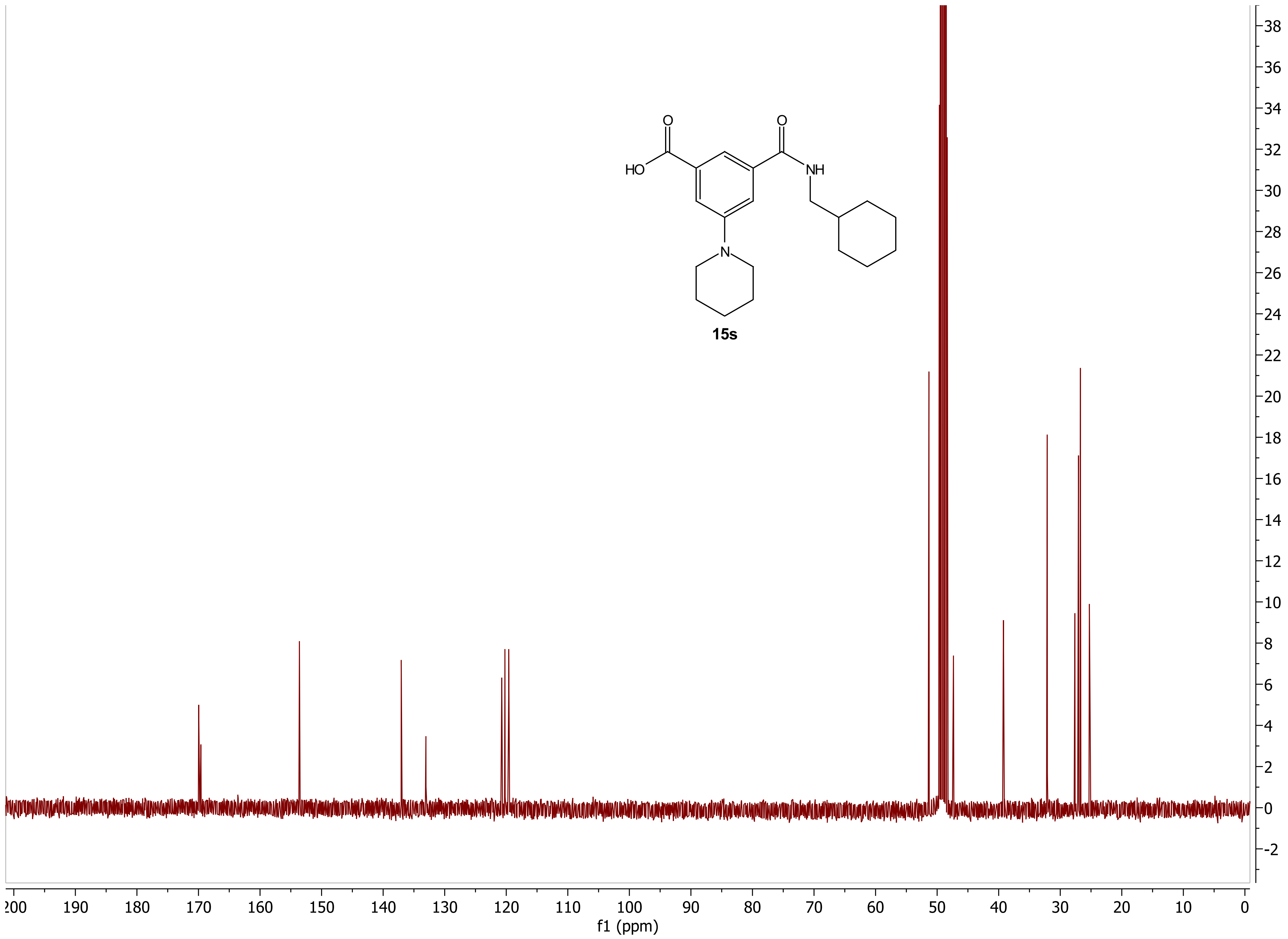

HO
O
O
NH
N
15s
38
36
34
32
30
28
26
24
22
20
18
16
14
12
10
8
6
4
2
0
-2
200
190
180
170
160
150
140
130
120
110
100
90
80
70
60
50
40
30
20
10
0
f1 (ppm)

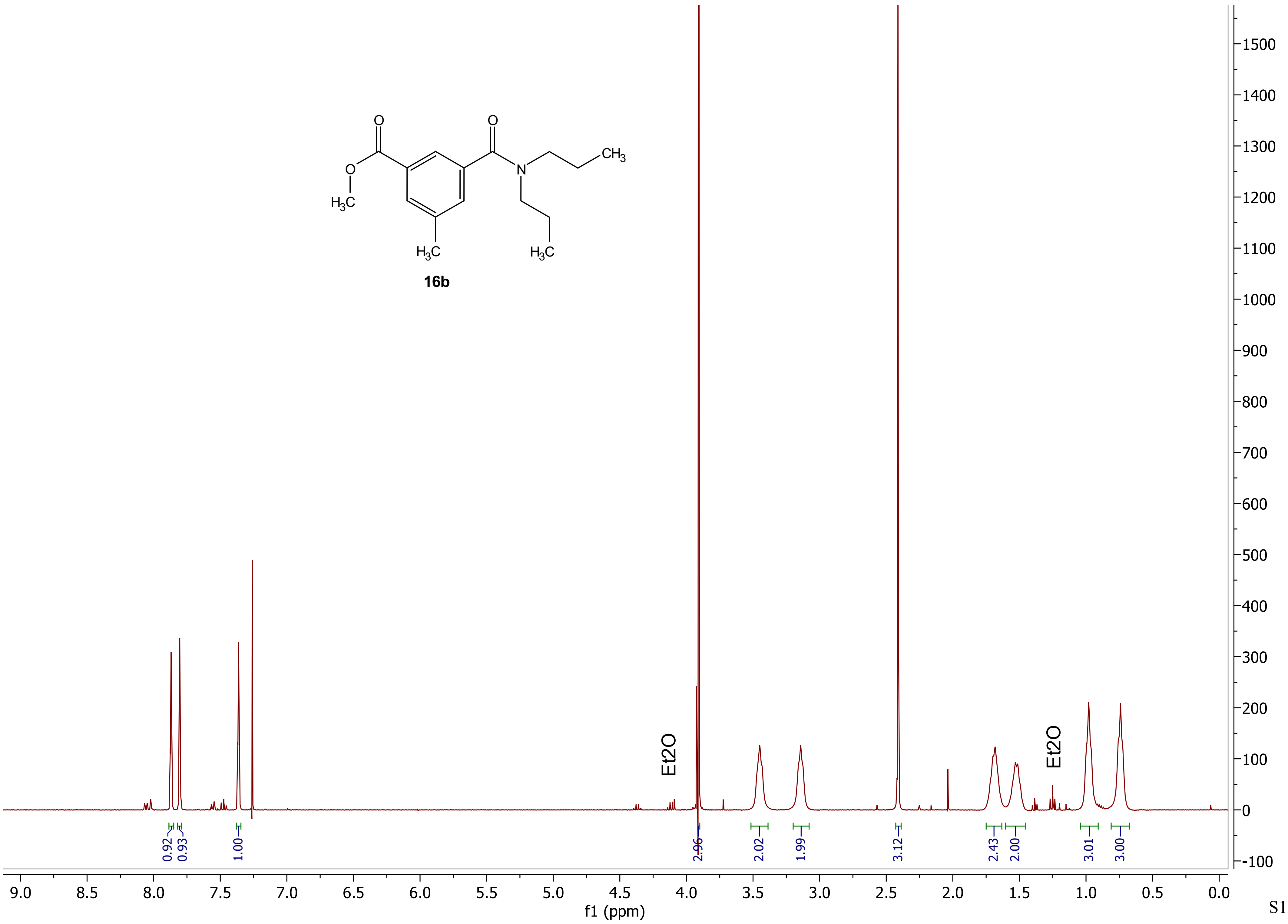
16b
Et2O
Et2O
0.92
0.93
1.00
2.96
2.02
1.99
3.12
2.43
2.00
3.01
3.00
f1 (ppm)

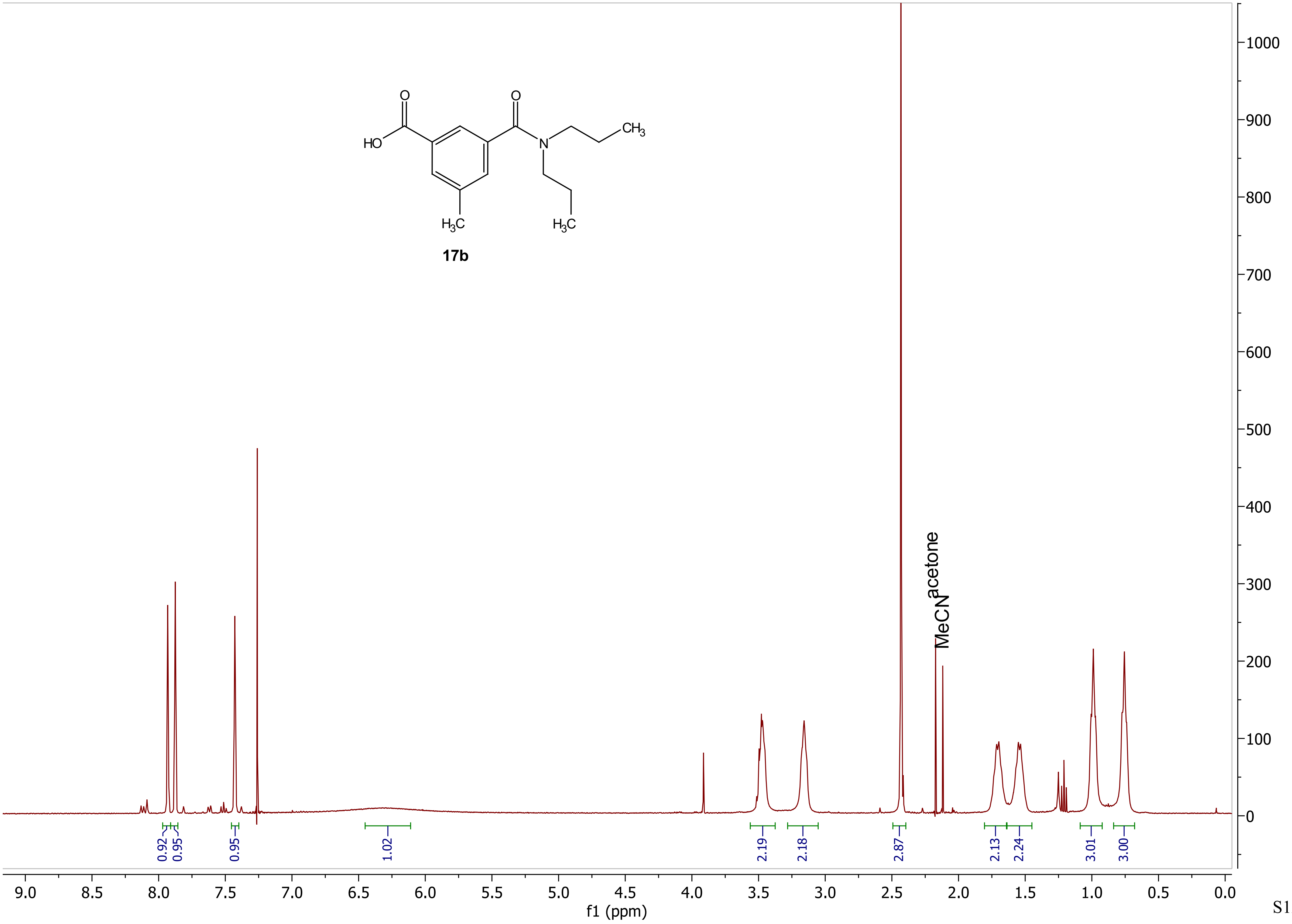

HO
O
O
N
CH3
H3C
H3C
17b
acetone
MeCN
0.92
0.95
0.95
1.02
2.19
2.18
2.87
2.13
2.24
3.01
3.00
9.0 8.5 8.0 7.5 7.0 6.5 6.0 5.5 5.0 4.5 4.0 3.5 3.0 2.5 2.0 1.5 1.0 0.5 0.0
f1 (ppm)
1000 900 800 700 600 500 400 300 200 100 0

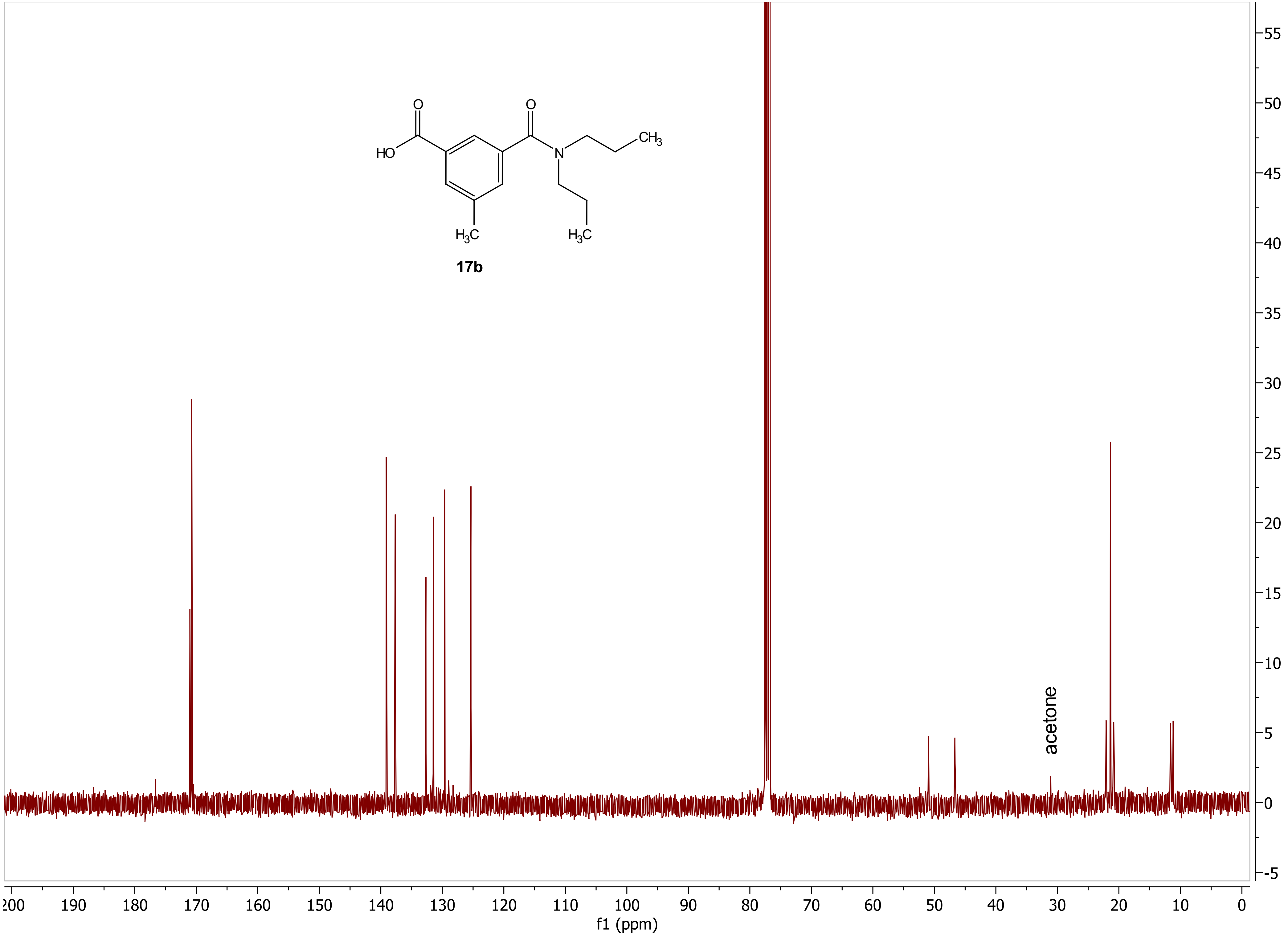

HO
O
O
N
CH3
H3C
H3C
17b
acetone
f1 (ppm)
200 190 180 170 160 150 140 130 120 110 100 90 80 70 60 50 40 30 20 10 0
55 50 45 40 35 30 25 20 15 10 5 0 -5

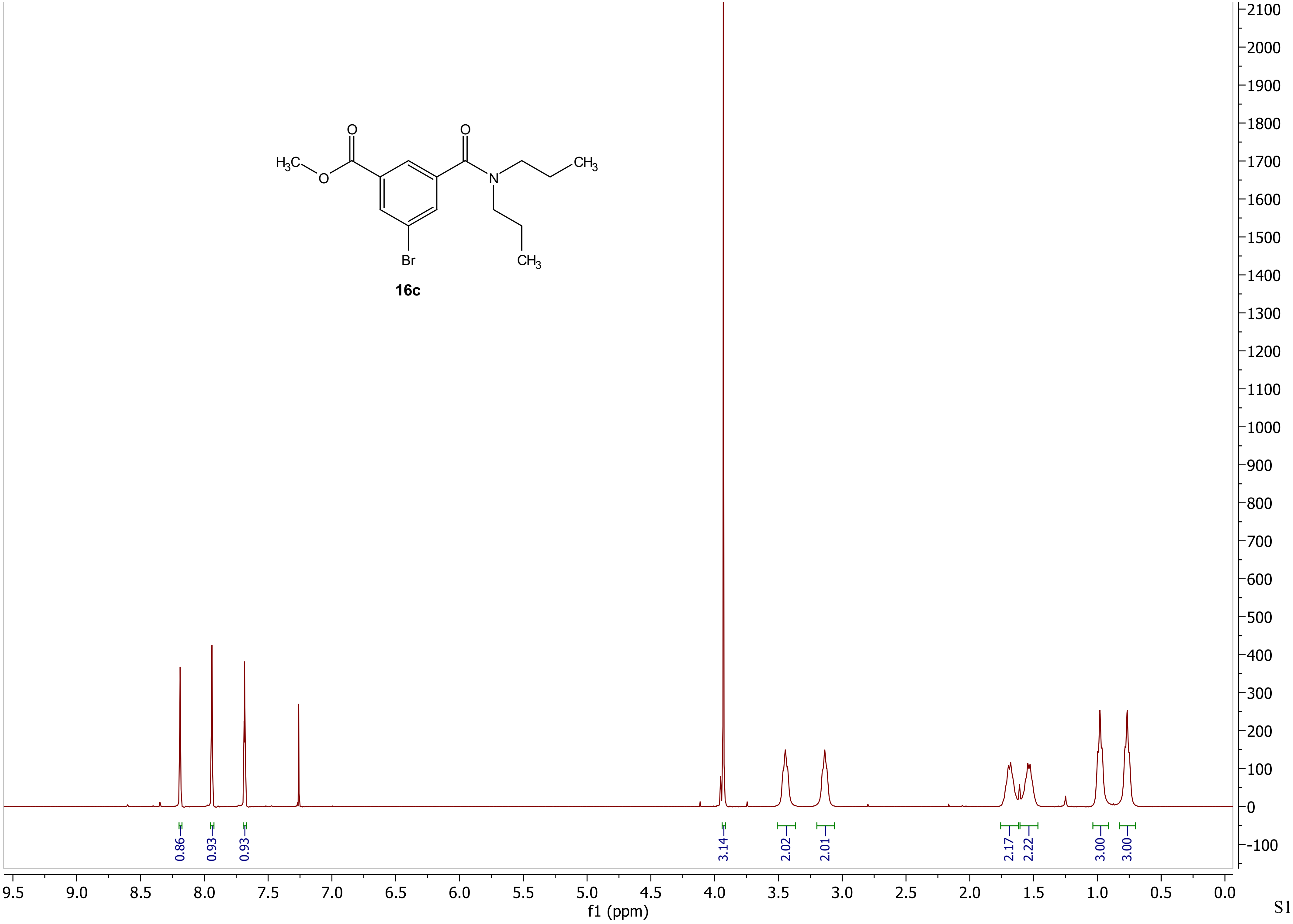
H3C
O
O
O
N
CH3
CH3
Br
16c
0.86
0.93
0.93
3.14
2.02
2.01
2.17
2.22
3.00
3.00
9.5 9.0 8.5 8.0 7.5 7.0 6.5 6.0 5.5 5.0 4.5 4.0 3.5 3.0 2.5 2.0 1.5 1.0 0.5 0.0
f1 (ppm)
2100 2000 1900 1800 1700 1600 1500 1400 1300 1200 1100 1000 900 800 700 600 500 400 300 200 100 0 -100

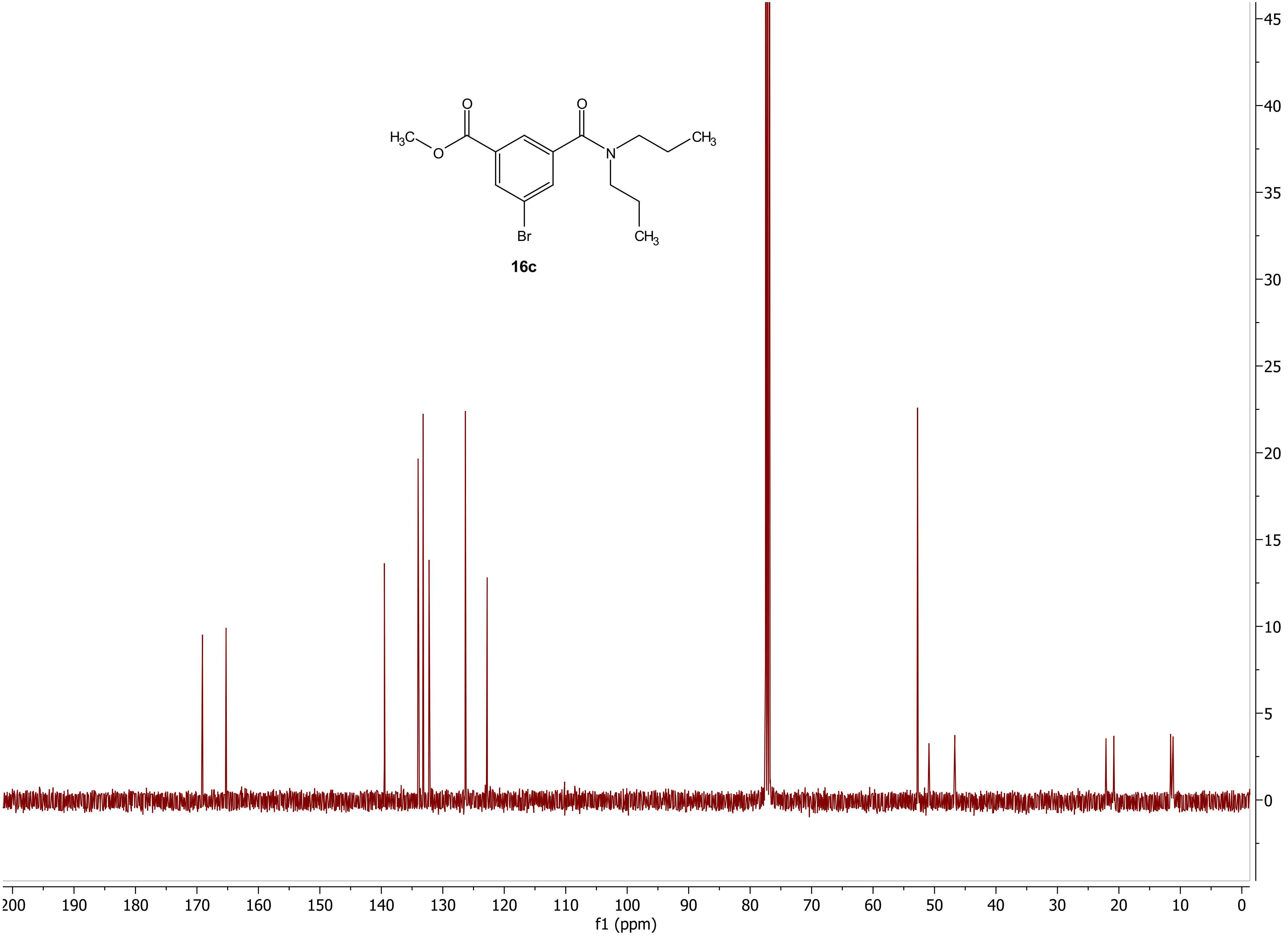

H3C
O
O
O
N
CH3
CH3
Br
16c
f1 (ppm)

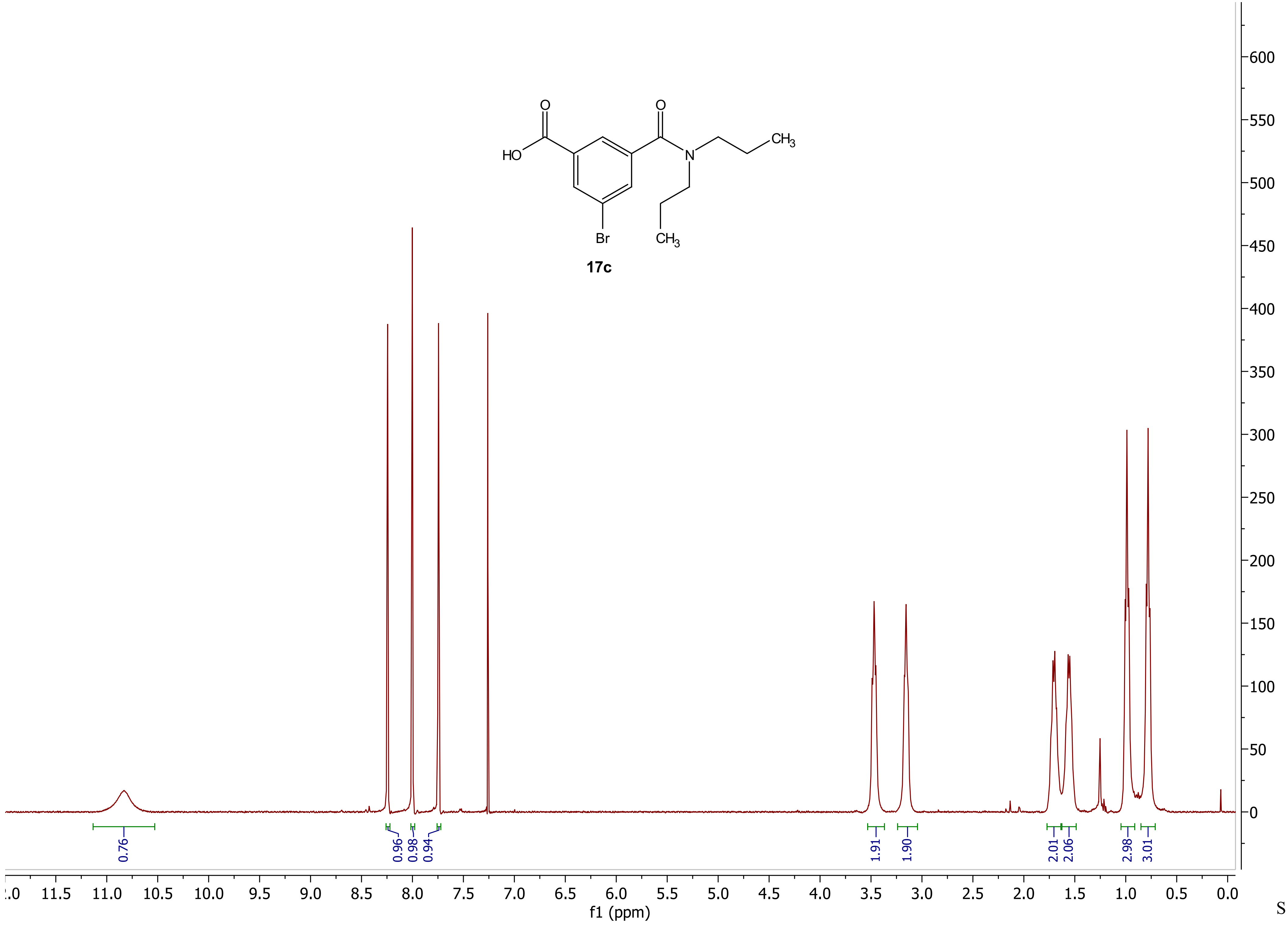
HO
O
O
N
CH3
CH3
Br
17c
600
550
500
450
400
350
300
250
200
150
100
50
0
0.76
0.96
0.98
0.94
1.91
1.90
2.01
2.06
2.98
3.01
11.5
11.0
10.5
10.0
9.5
9.0
8.5
8.0
7.5
7.0
6.5
6.0
5.5
5.0
4.5
4.0
3.5
3.0
2.5
2.0
1.5
1.0
0.5
0.0
f1 (ppm)

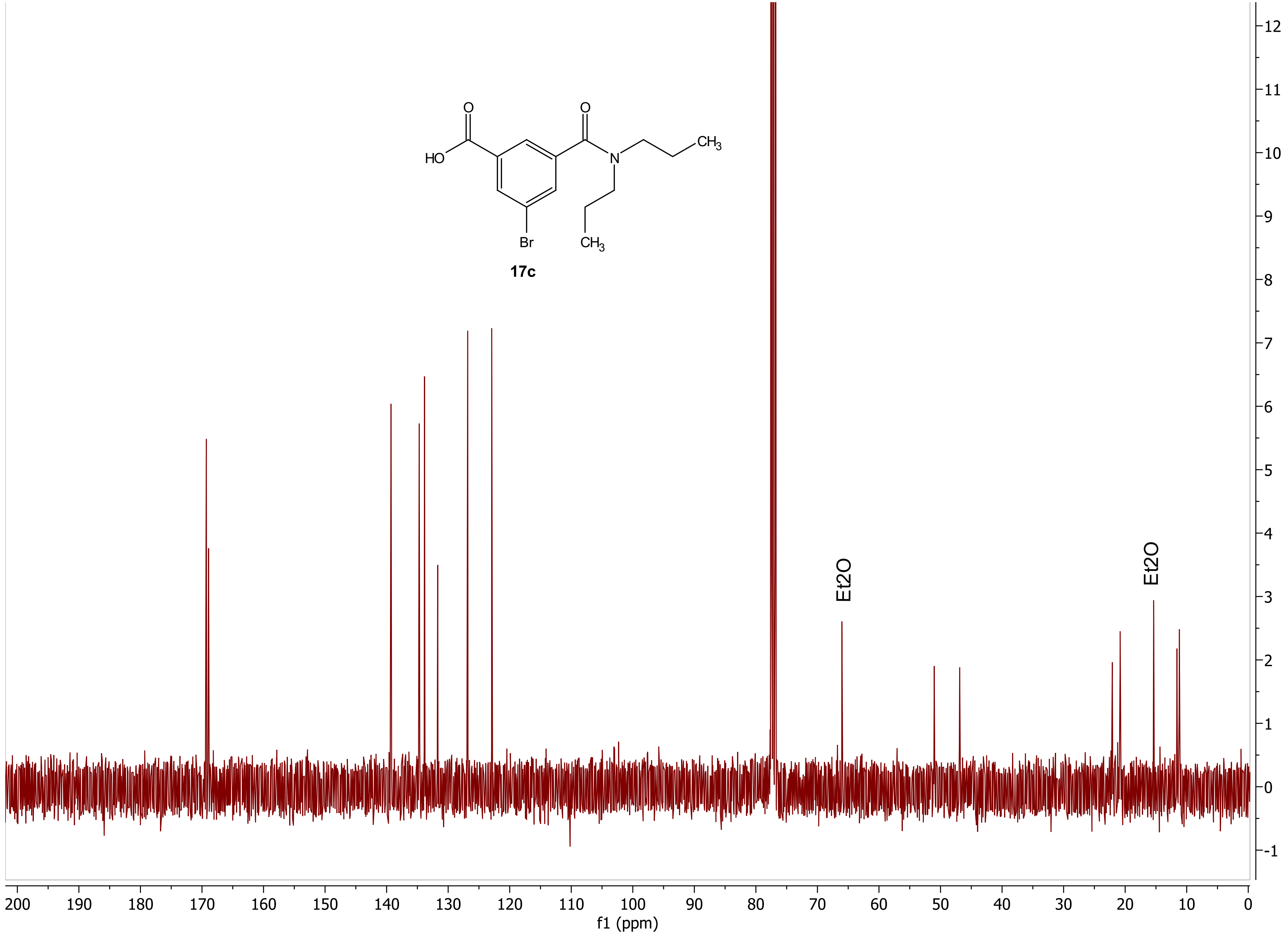

O
O
HO
N
CH3
Br
CH3
17c
Et2O
Et2O
12
11
10
9
8
7
6
5
4
3
2
1
0
-1
200
190
180
170
160
150
140
130
120
110
100
90
80
70
60
50
40
30
20
10
0
f1 (ppm)

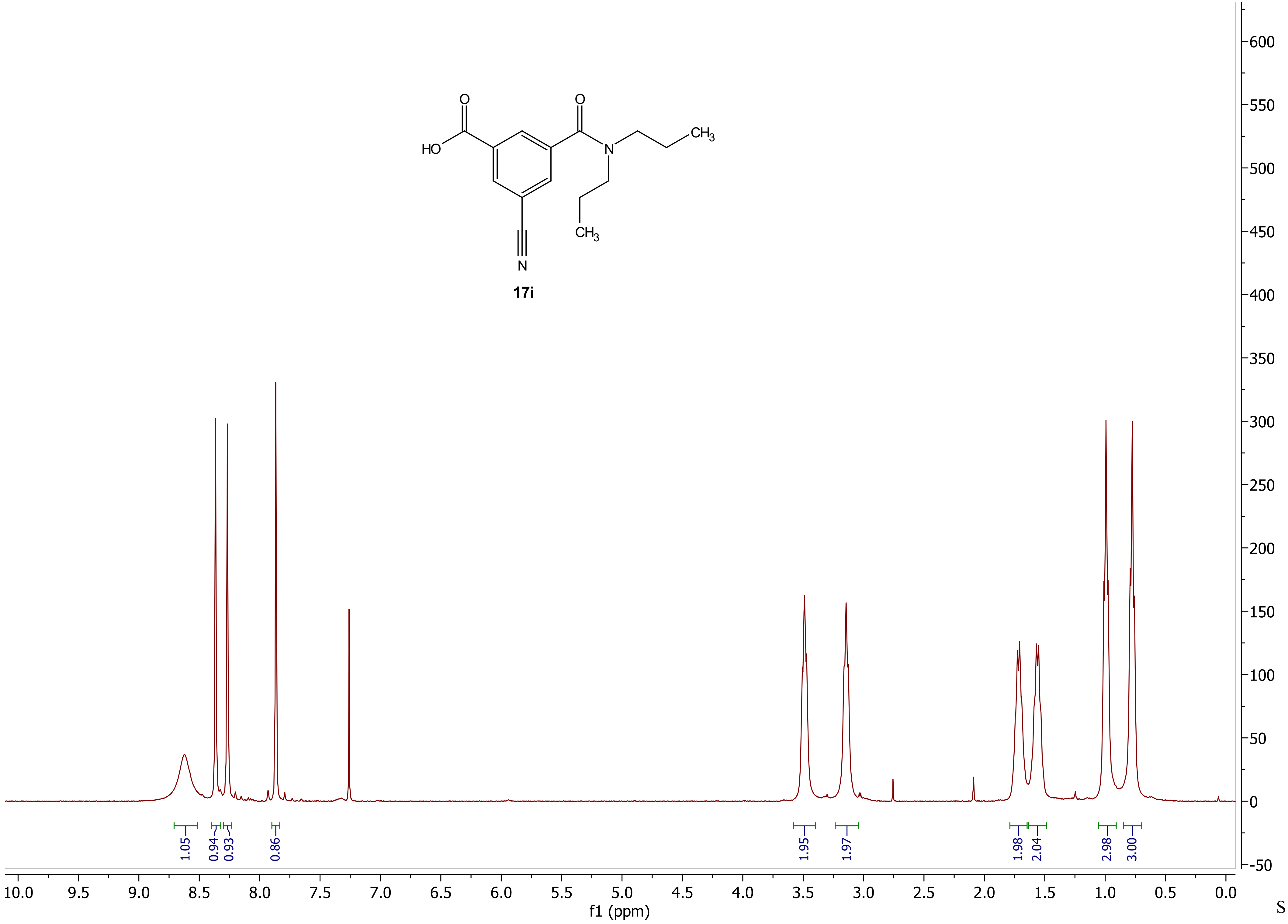

HO
O
O
N
CH3
CH3
N
17i
1.05
0.94
0.93
0.86
1.95
1.97
1.98
2.04
2.98
3.00
f1 (ppm)

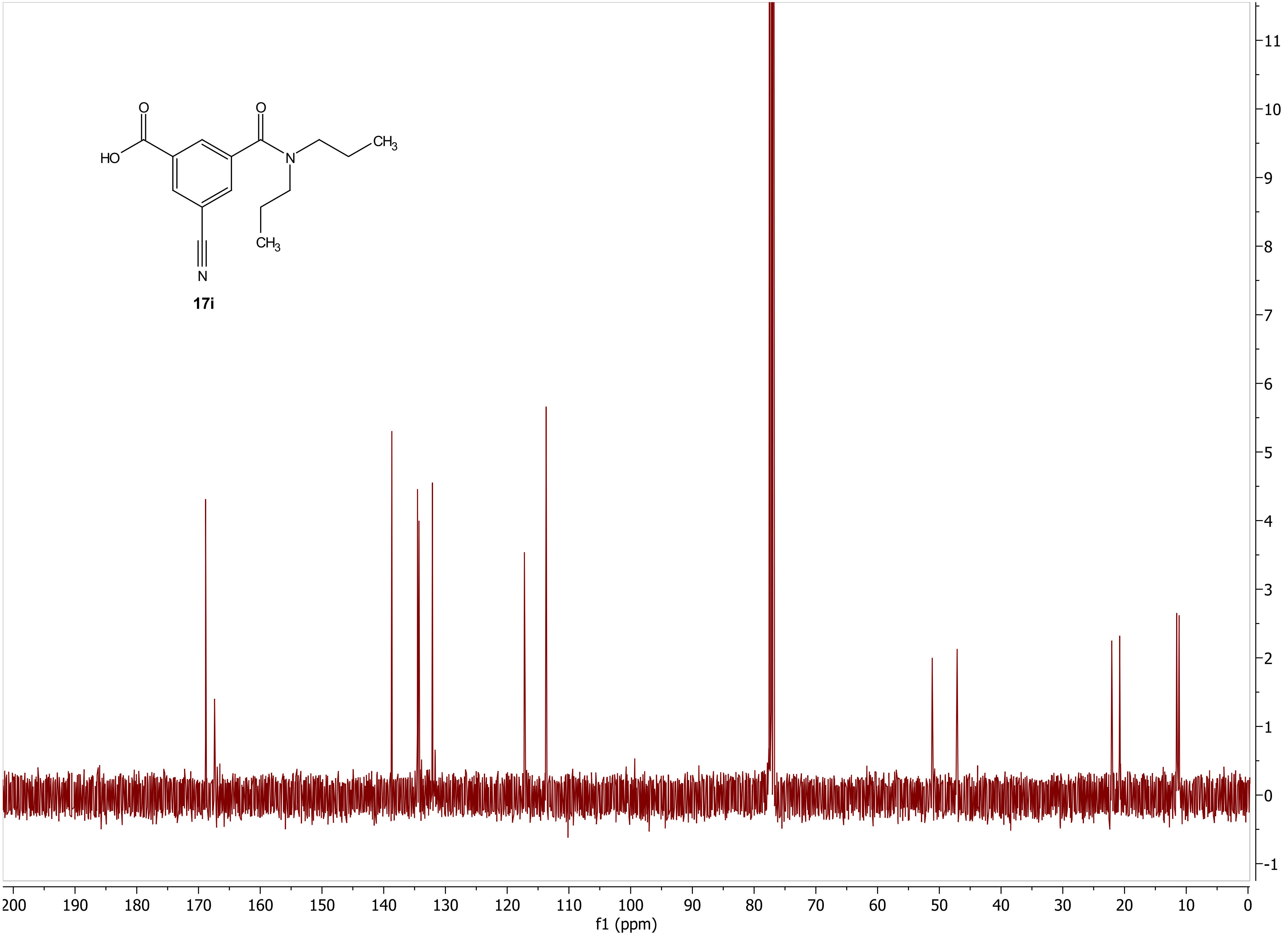

17i
f1 (ppm)

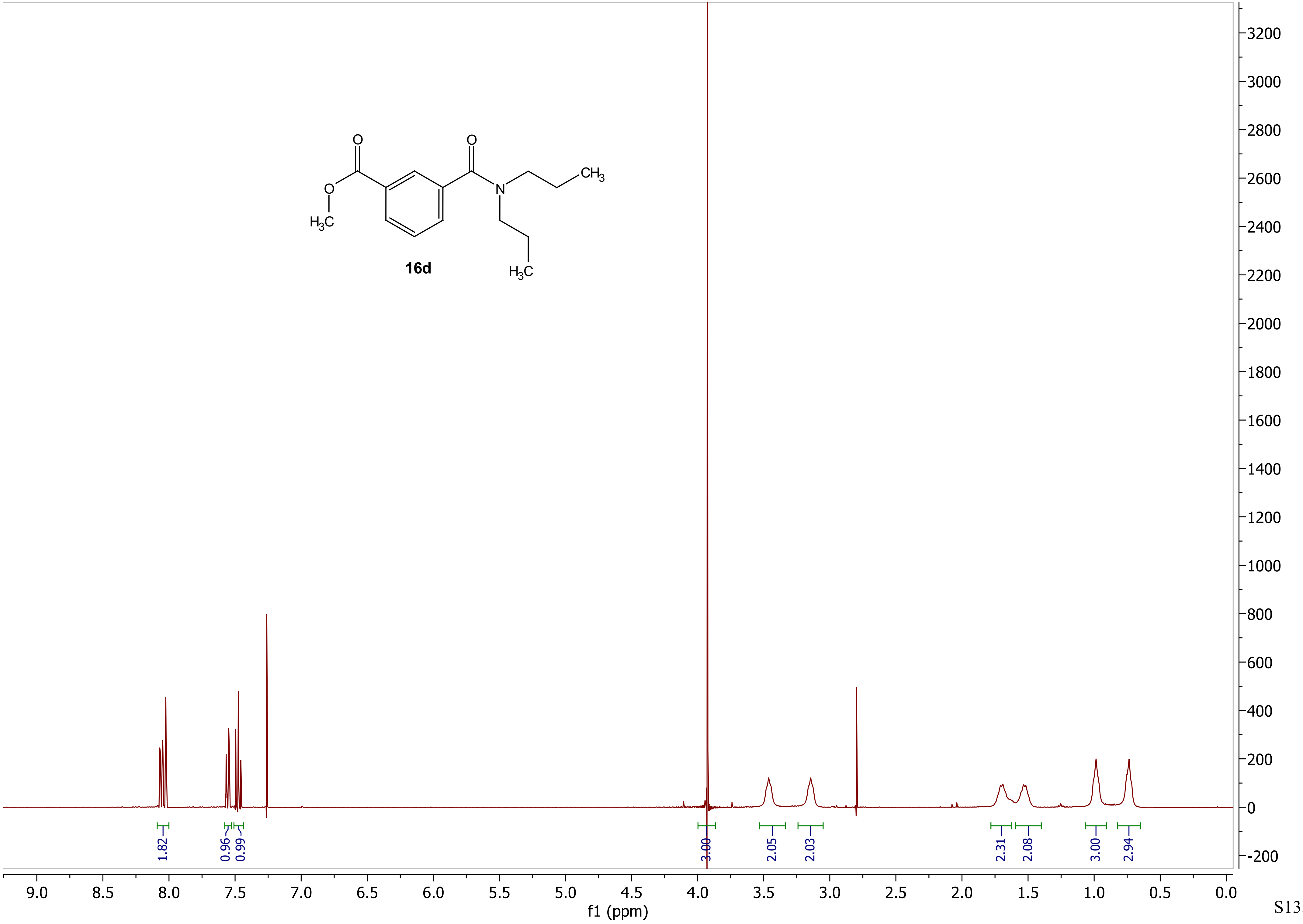

16d
1.82
0.96
0.99
3.00
2.05
2.03
2.31
2.08
3.00
2.94
f1 (ppm)

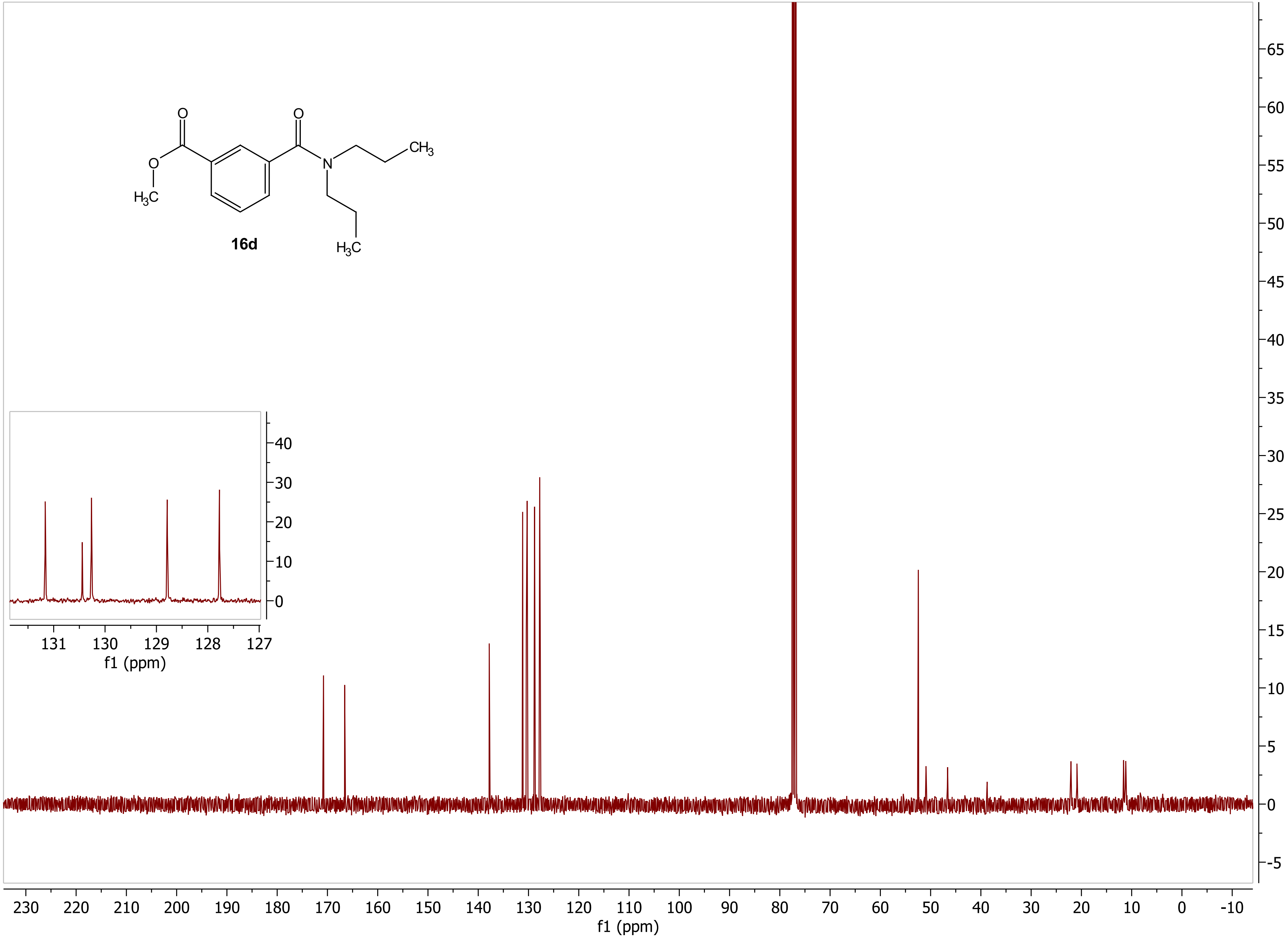
O
O
O
N
CH_3
H_3C
H_3C
16d
f1 (ppm)
f1 (ppm)

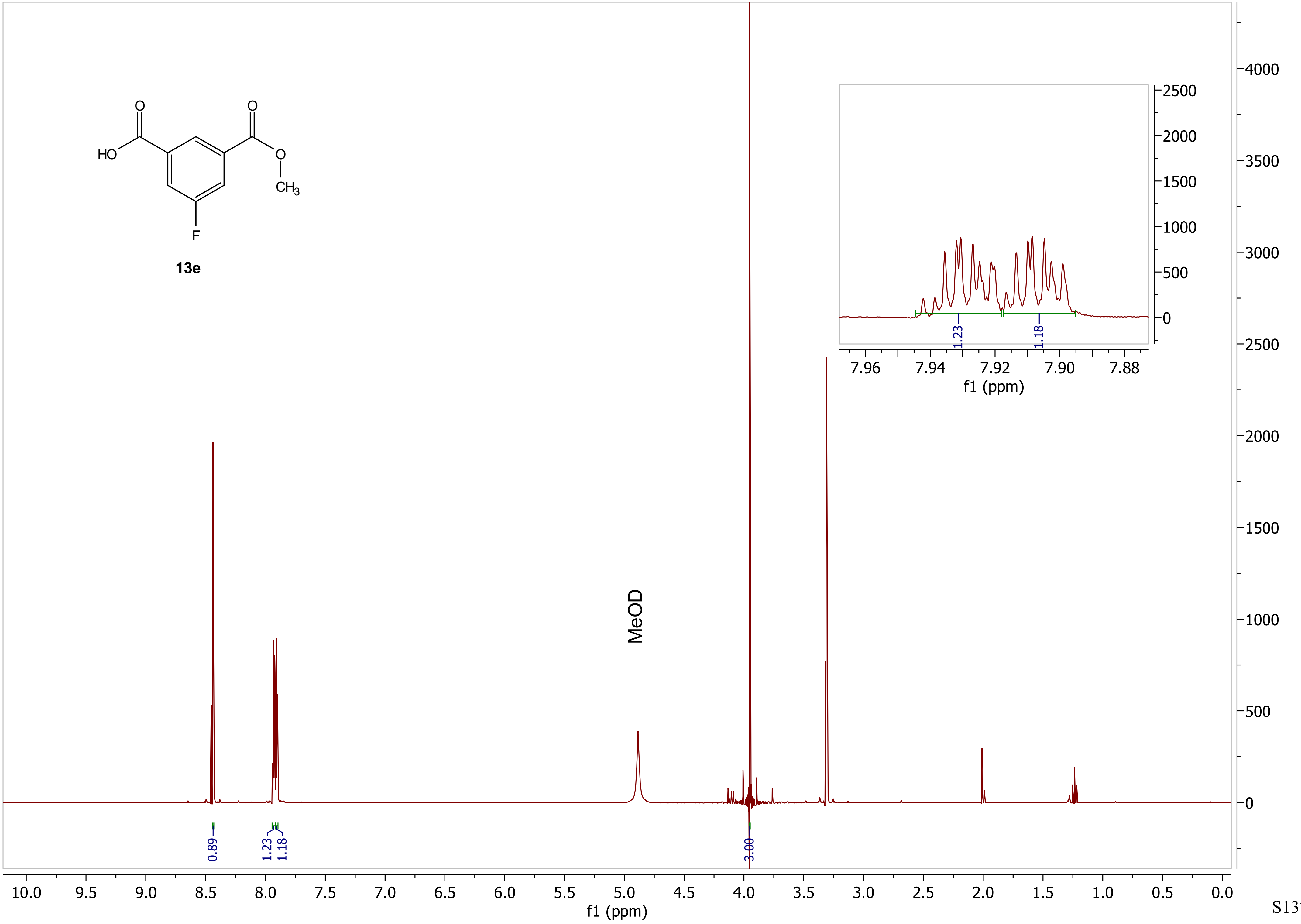
HO
O
O
O
CH3
F
13e
MeOD
0.89
1.23
1.18
3.00
f1 (ppm)
7.96
7.94
7.92
7.90
7.88

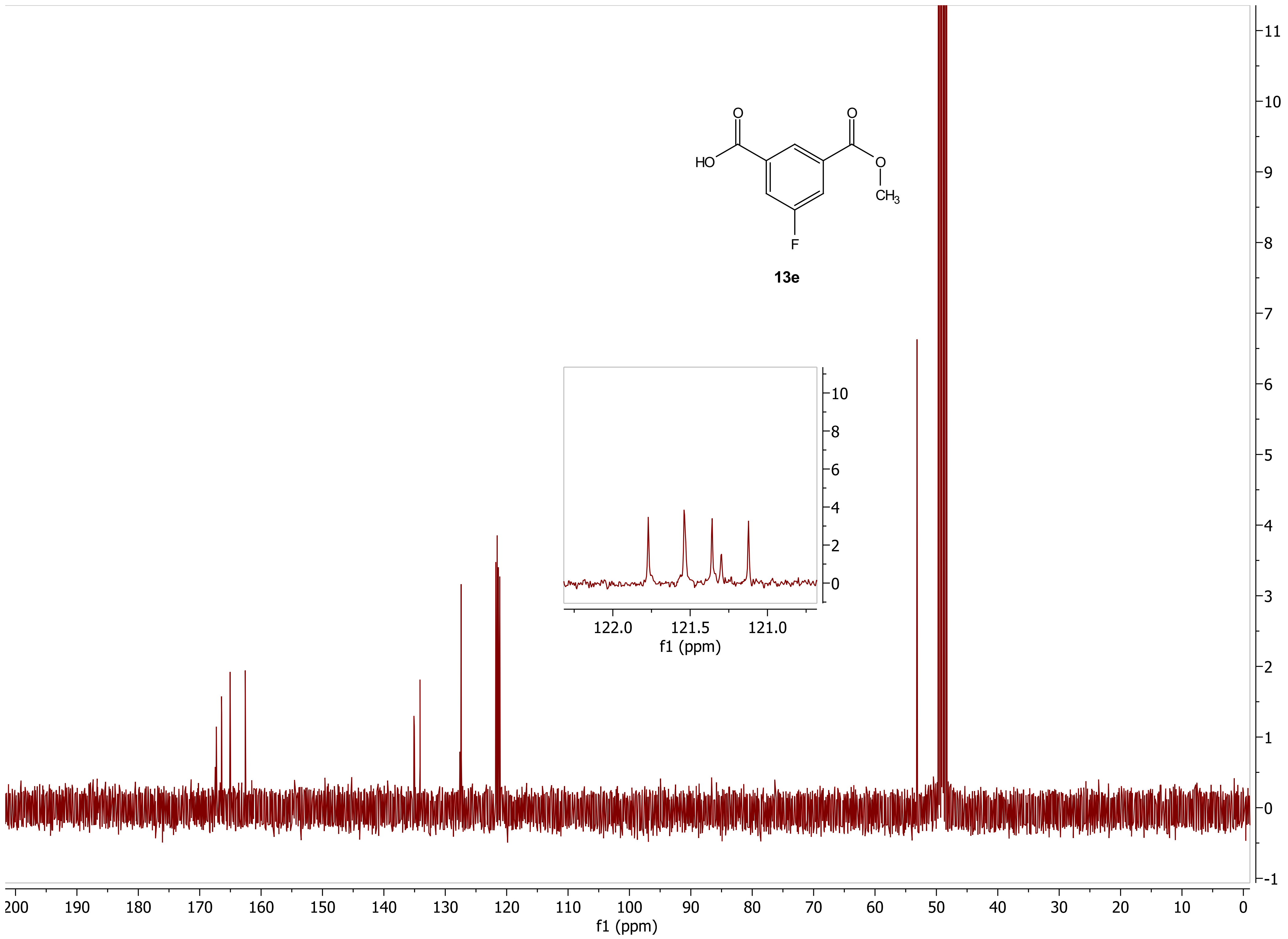

HO
O
O
O
CH3
F
13e
122.0
121.5
121.0
f1 (ppm)
f1 (ppm)

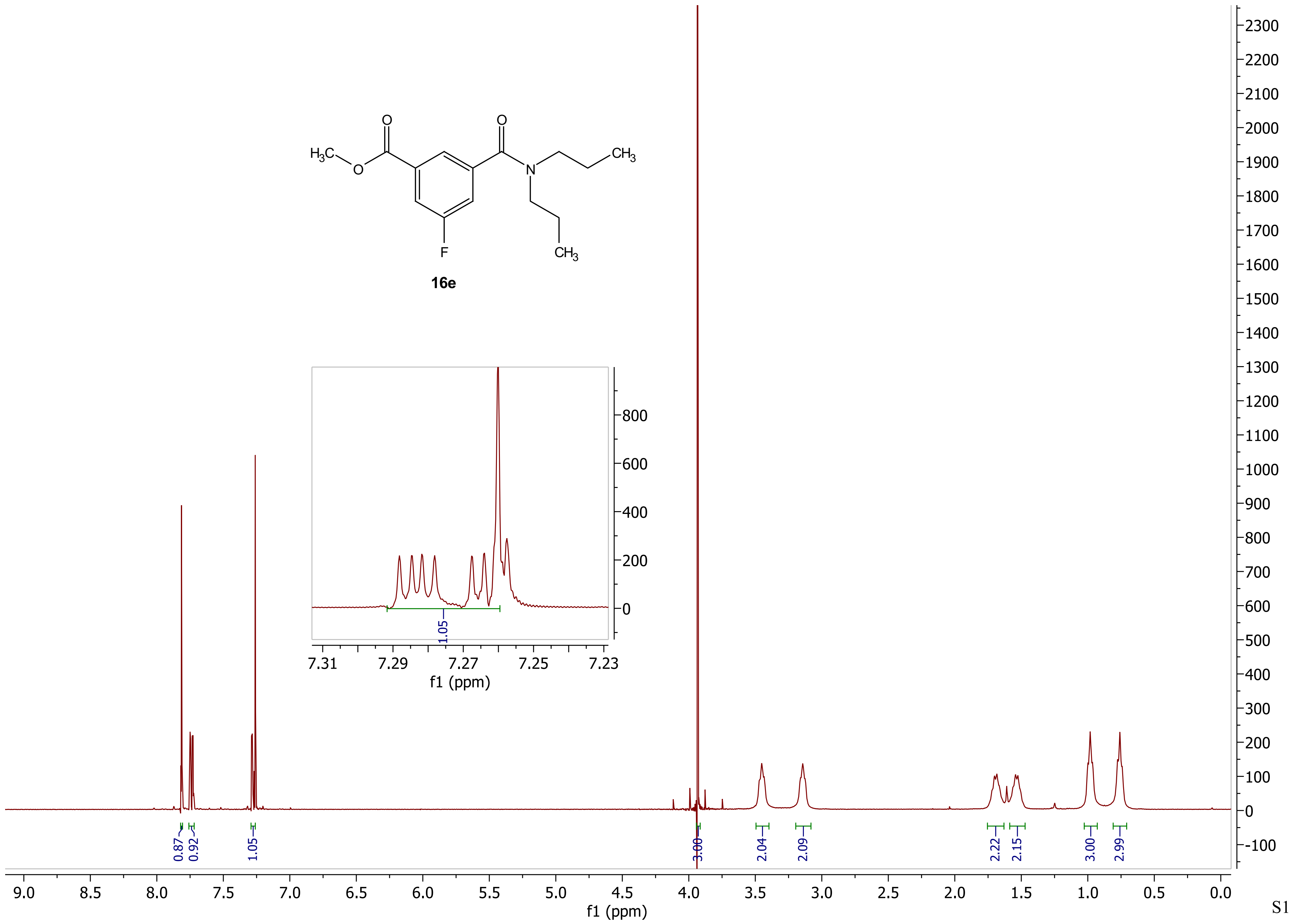

16e
F
CH3
H3C
O
N
f1 (ppm)
1.05
0.87
0.92
3.00
2.04
2.09
2.22
2.15
2.99

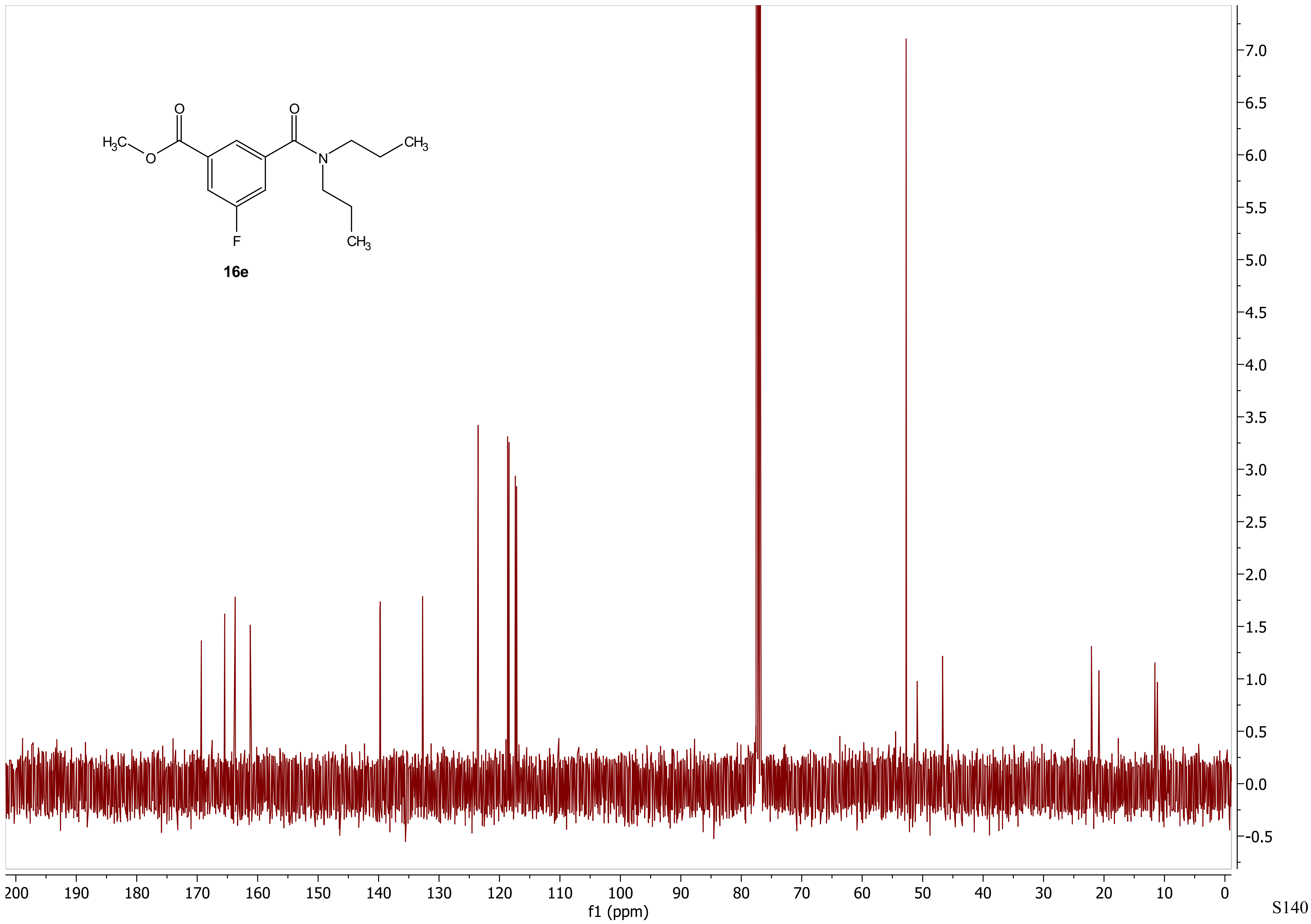

O
O
H3C
O
N
CH3
F
CH3
16e
7.0
6.5
6.0
5.5
5.0
4.5
4.0
3.5
3.0
2.5
2.0
1.5
1.0
0.5
0.0
-0.5
200
190
180
170
160
150
140
130
120
110
100
90
80
70
60
50
40
30
20
10
0
f1 (ppm)

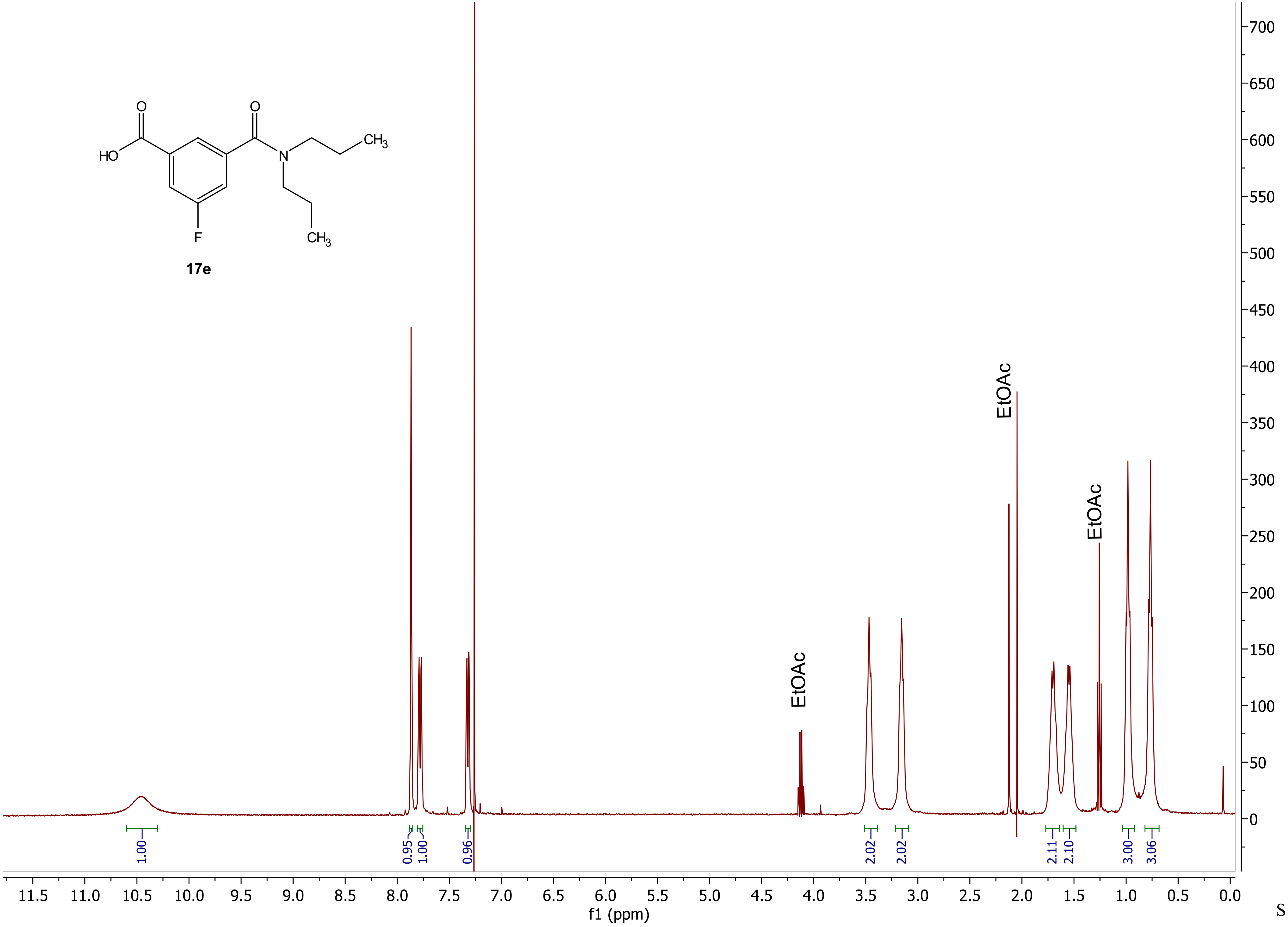

HO
O
O
N
CH3
CH3
F
17e
EtOAc
EtOAc
EtOAc
1.00
0.95
1.00
0.96
2.02
2.02
2.11
2.10
3.00
3.06
f1 (ppm)

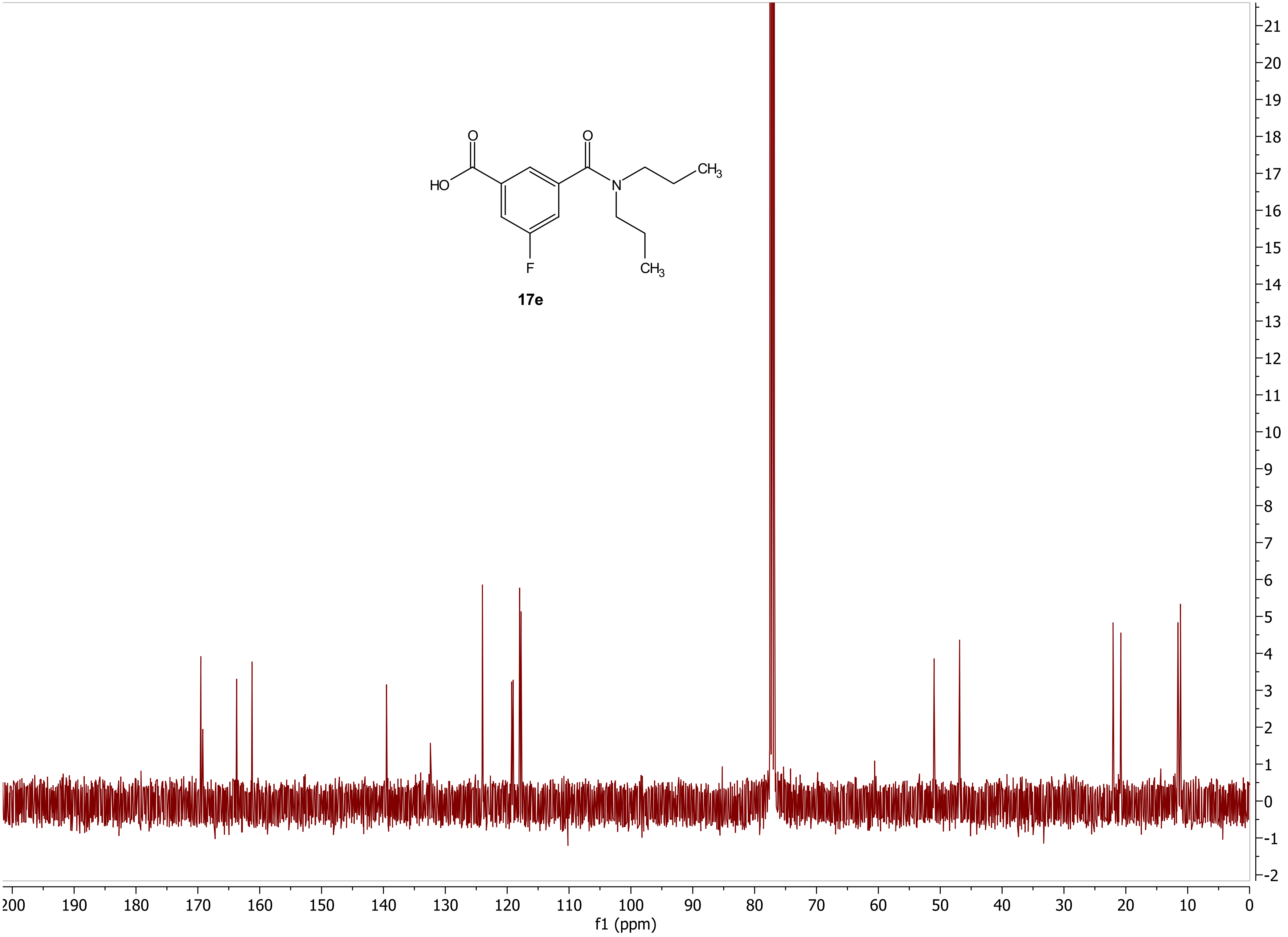

17e

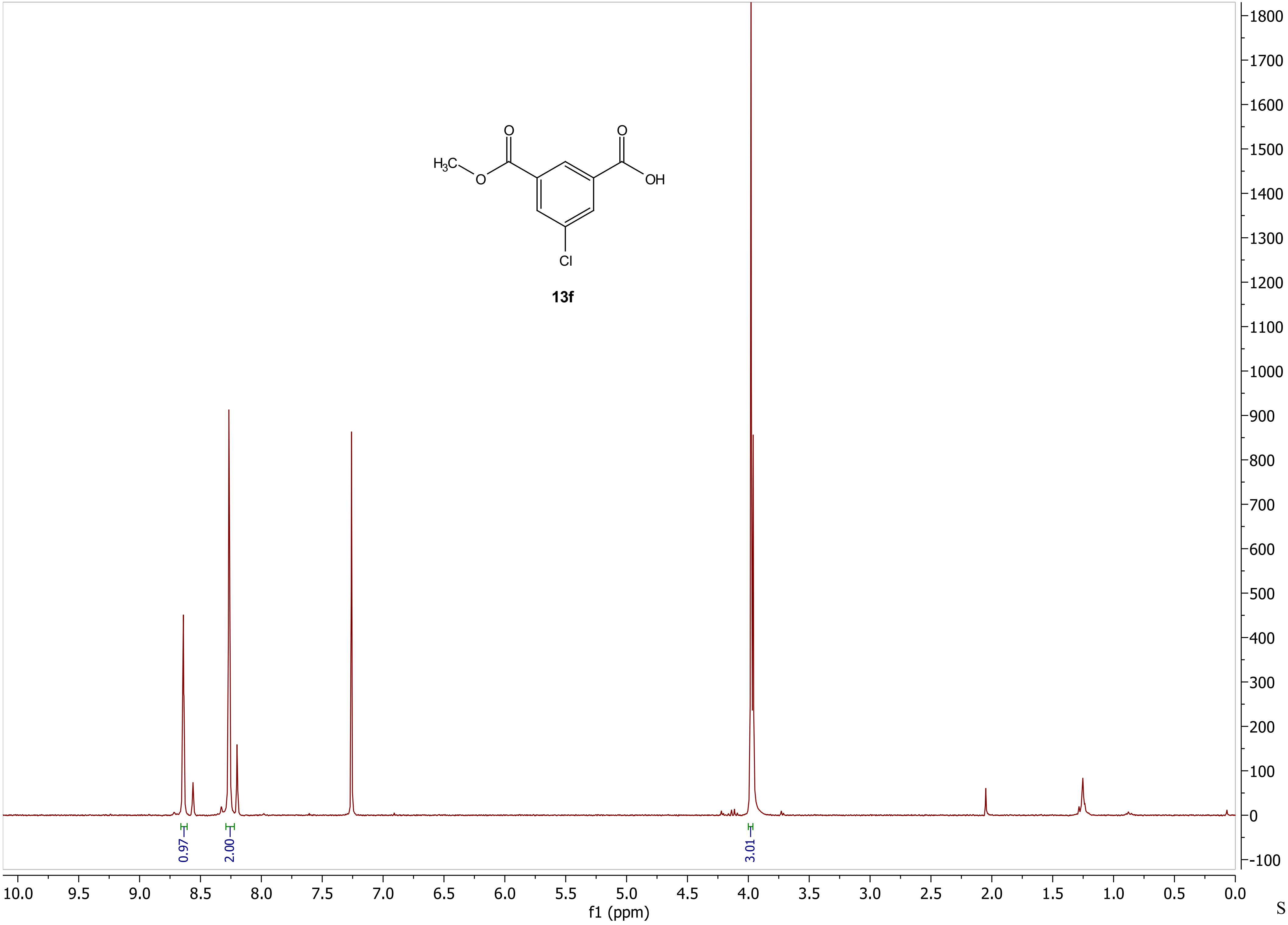

13f
0.97
2.00
3.01
f1 (ppm)

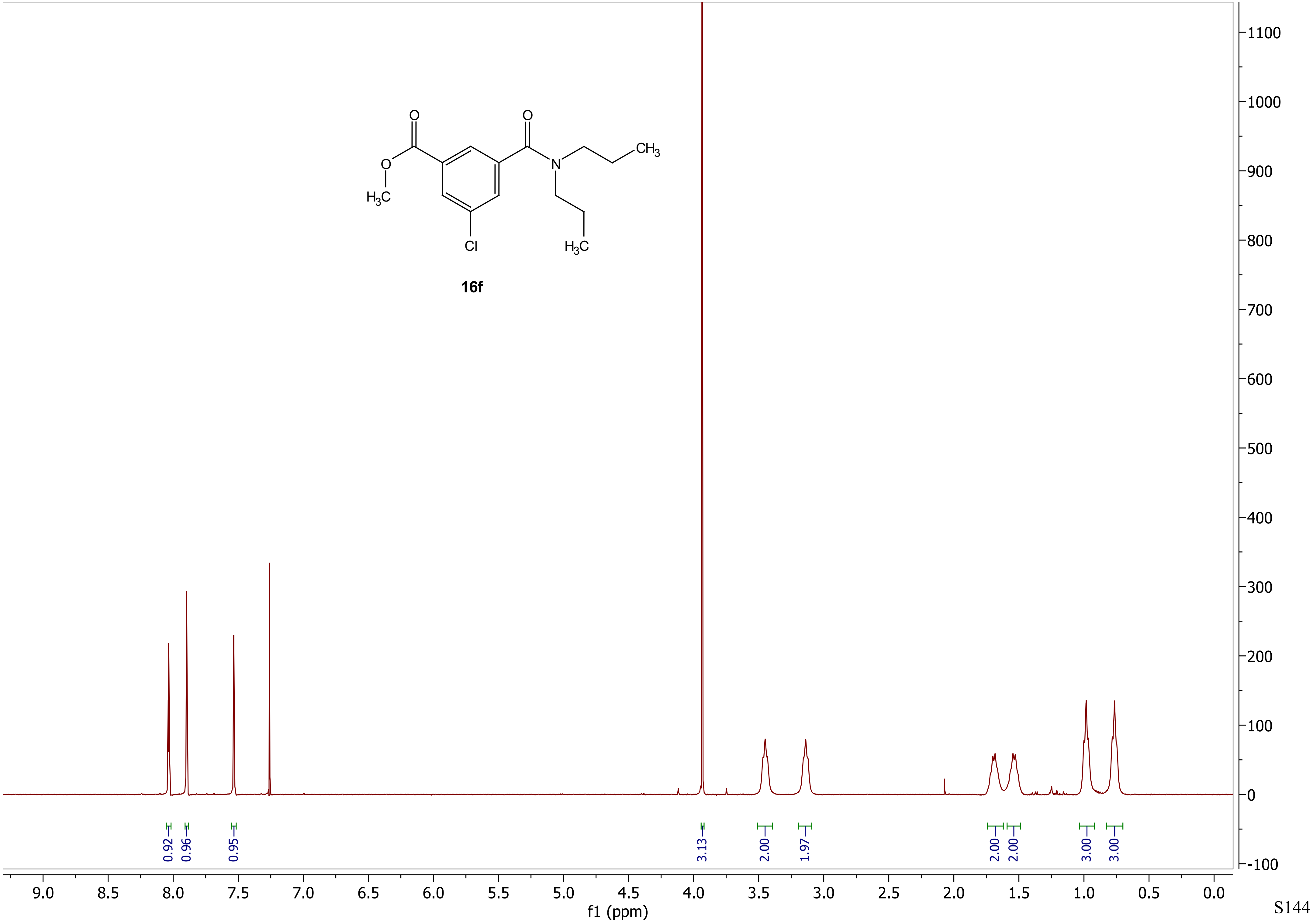
O
O
O
N
CH3
H3C
Cl
H3C
16f
0.92
0.96
0.95
3.13
2.00
1.97
2.00
2.00
3.00
3.00
9.0 8.5 8.0 7.5 7.0 6.5 6.0 5.5 5.0 4.5 4.0 3.5 3.0 2.5 2.0 1.5 1.0 0.5 0.0
f1 (ppm)
1100 1000 900 800 700 600 500 400 300 200 100 0 -100

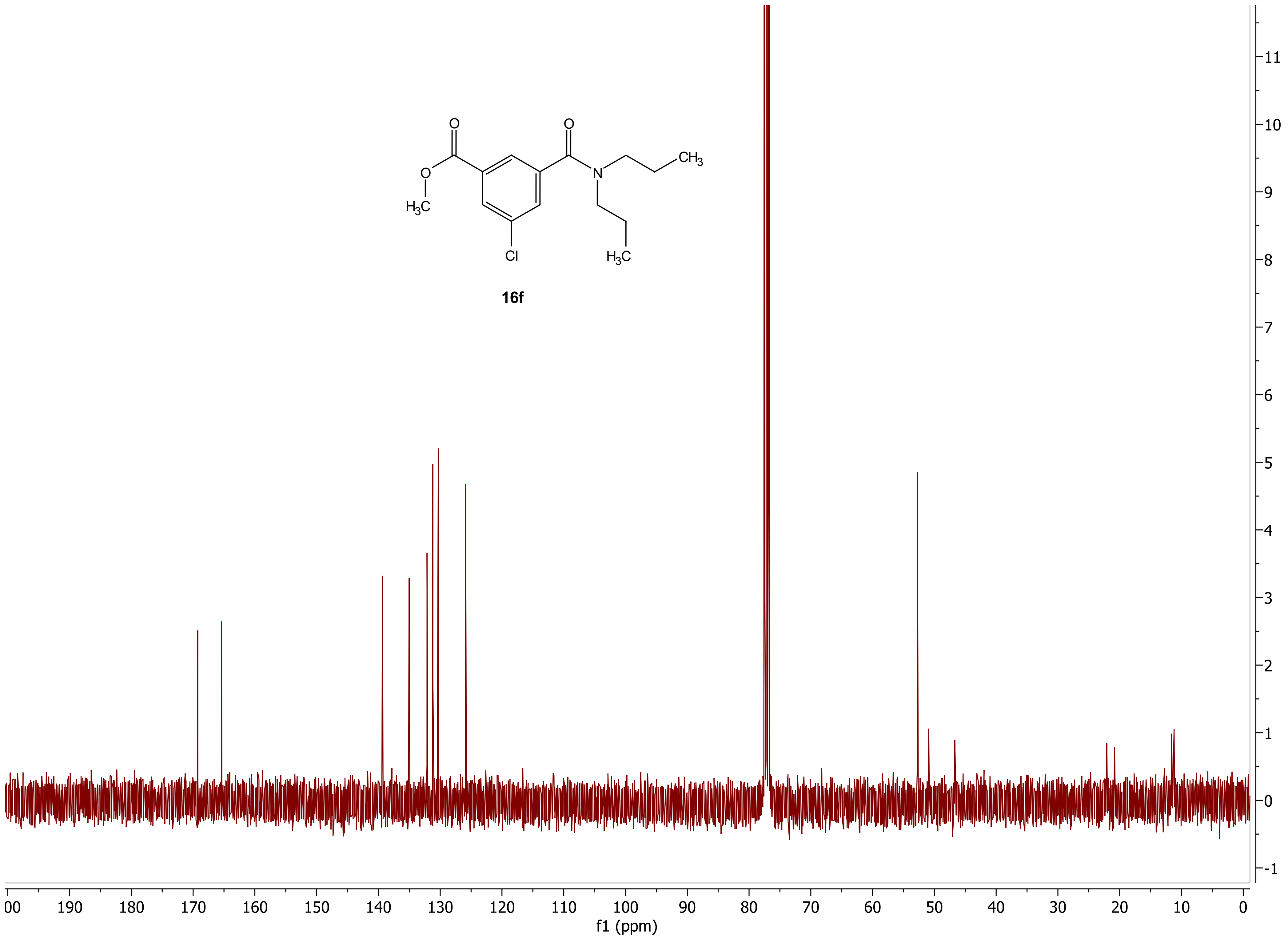

O
O
O
N
H3C
Cl
H3C
CH3
16f
f1 (ppm)

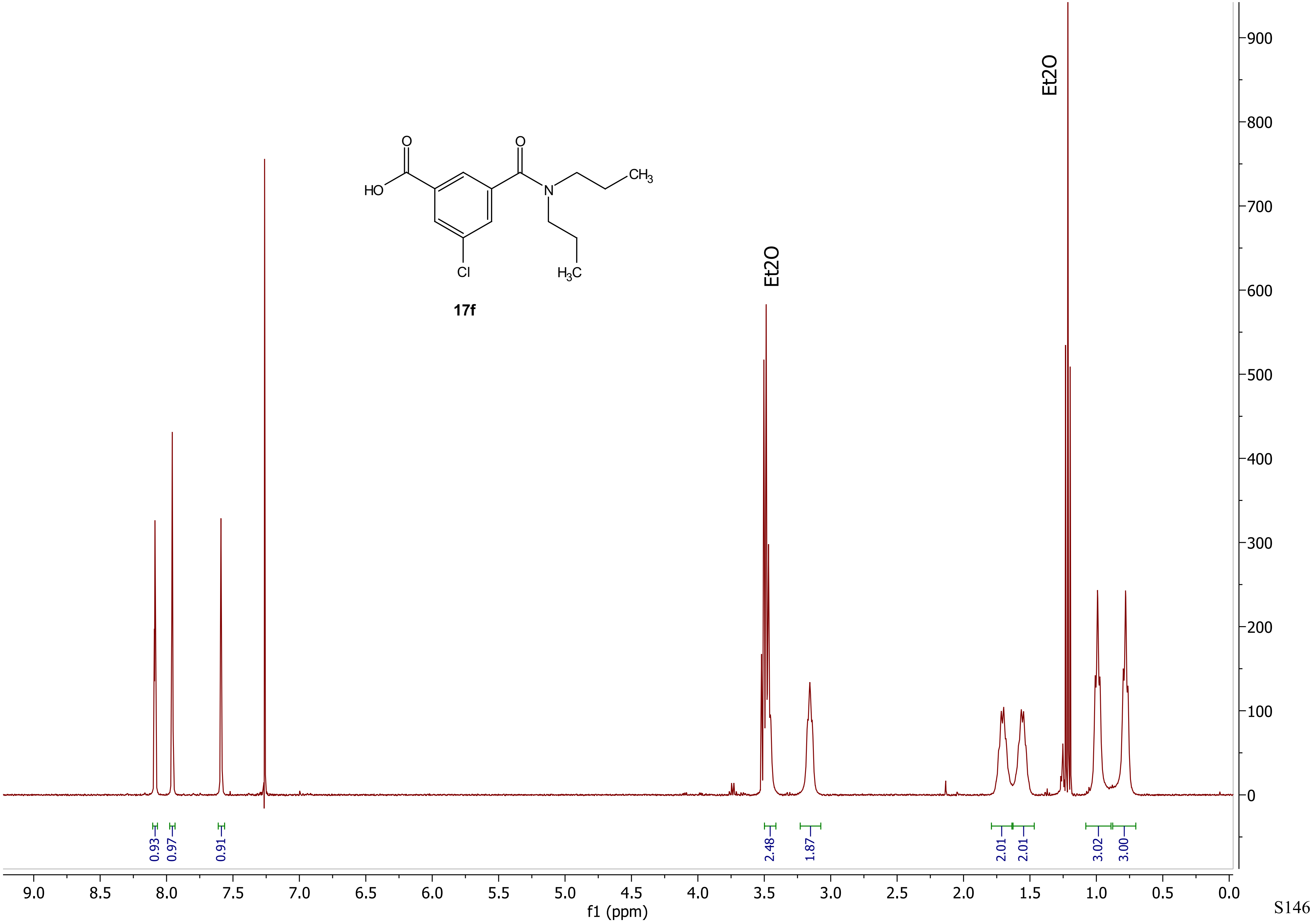

O
O
HO
N
CH3
Cl
H3C
17f
Et2O
Et2O
0.93
0.97
0.91
2.48
1.87
2.01
2.01
3.02
3.00
9.0
8.5
8.0
7.5
7.0
6.5
6.0
5.5
5.0
4.5
4.0
3.5
3.0
2.5
2.0
1.5
1.0
0.5
0.0
f1 (ppm)
900
800
700
600
500
400
300
200
100
0

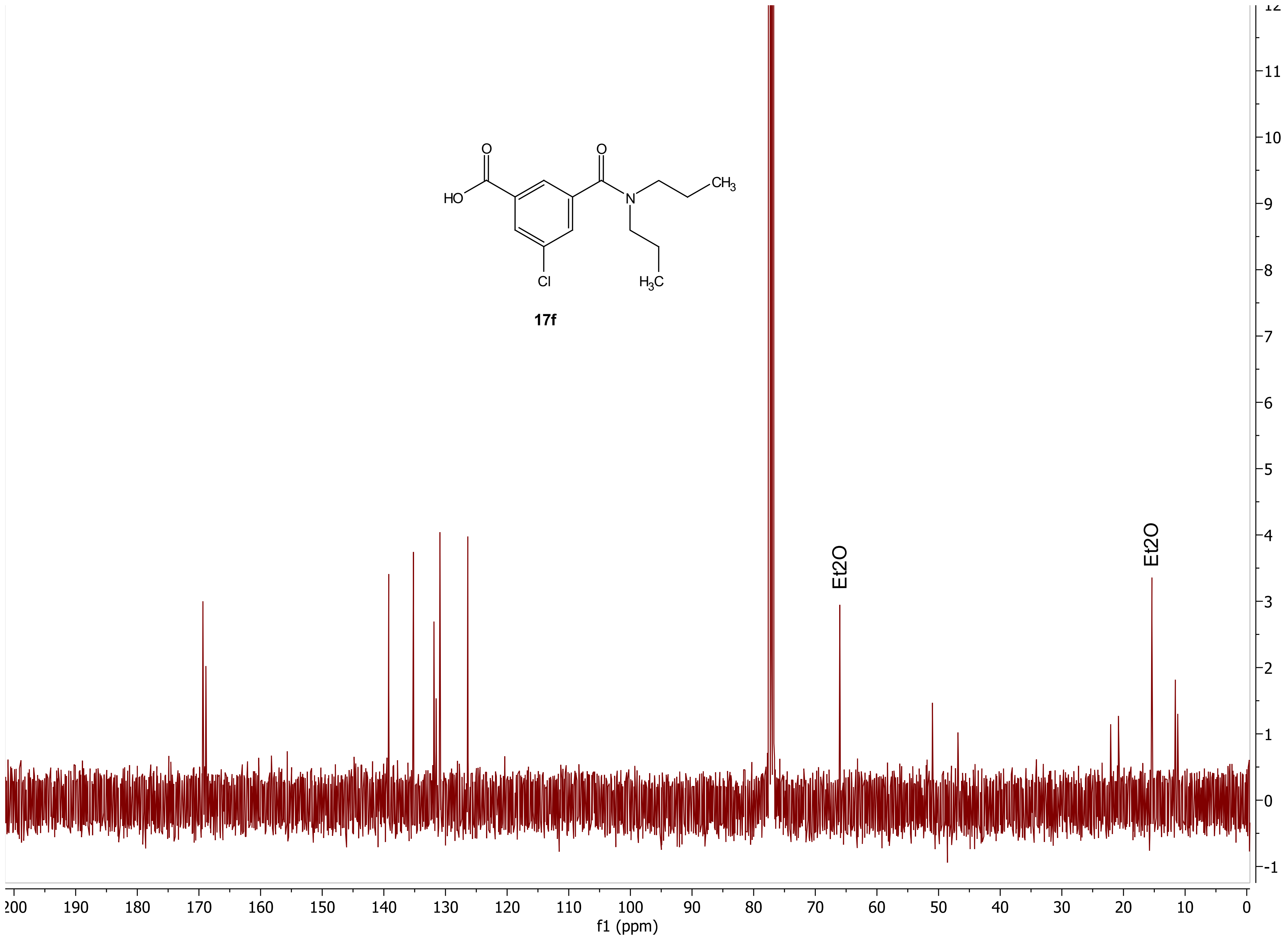
HO
O
O
N
CH3
Cl
H3C
17f
Et2O
Et2O
200 190 180 170 160 150 140 130 120 110 100 90 80 70 60 50 40 30 20 10 0
f1 (ppm)
12 11 10 9 8 7 6 5 4 3 2 1 0 -1

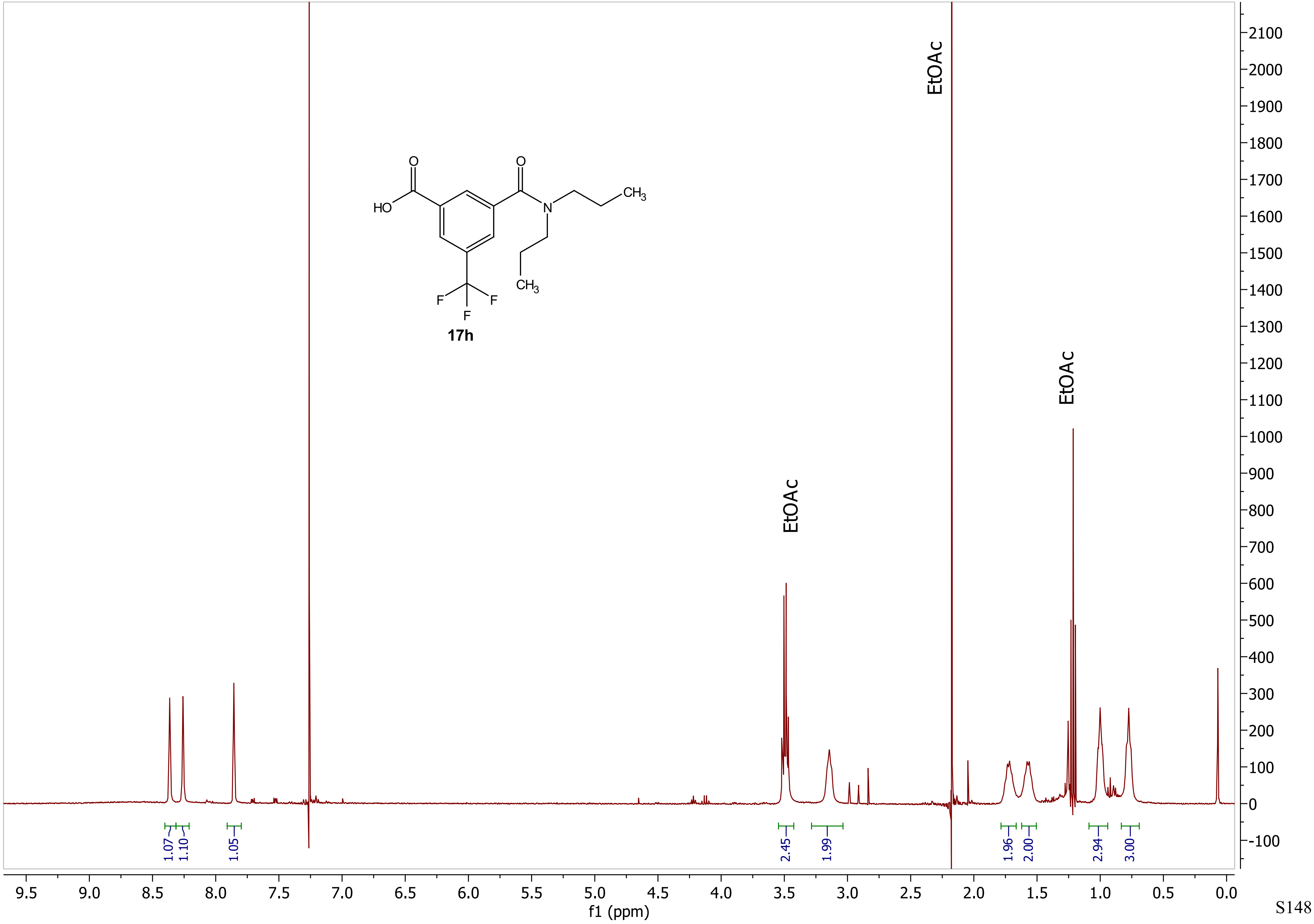

17h
EtOAc
EtOAc
EtOAc
1.07
1.10
1.05
2.45
1.99
1.96
2.00
2.94
3.00
f1 (ppm)

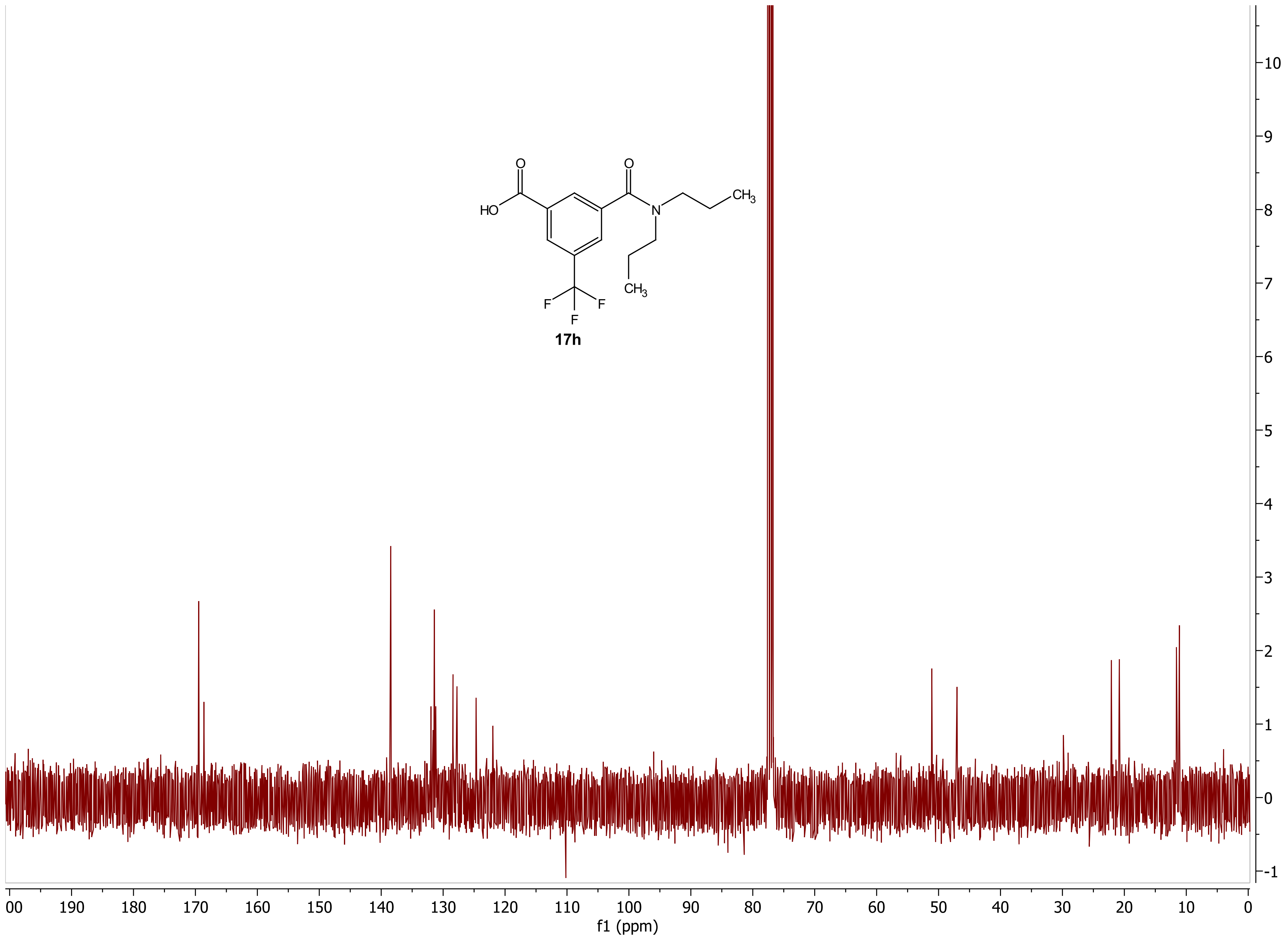

O
O
HO
N
CH3
CH3
F
F
F
17h
f1 (ppm)

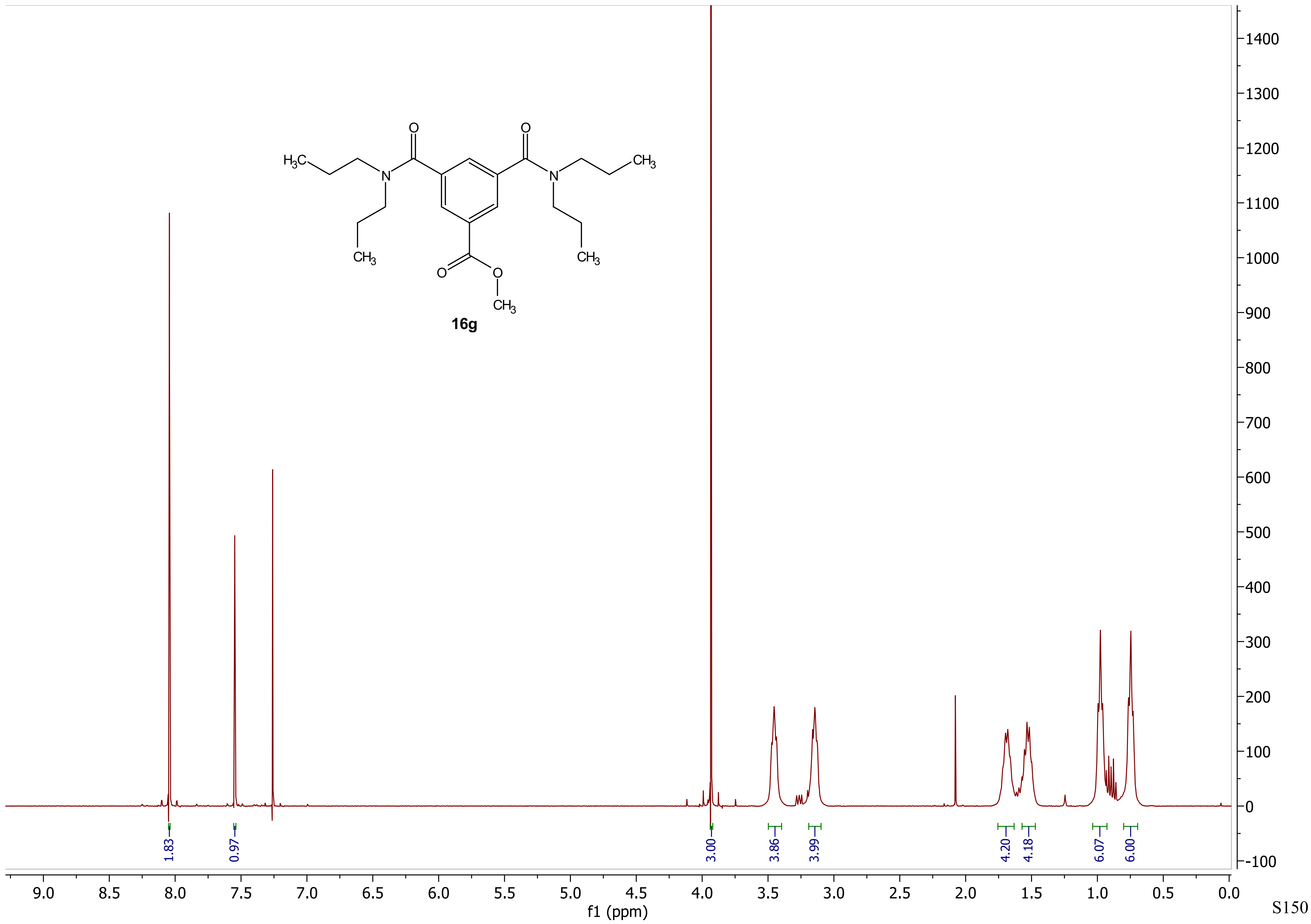
16g
1.83
0.97
3.00
3.86
3.99
4.20
4.18
6.07
6.00
9.0 8.5 8.0 7.5 7.0 6.5 6.0 5.5 5.0 4.5 4.0 3.5 3.0 2.5 2.0 1.5 1.0 0.5 0.0
f1 (ppm)
1400 1300 1200 1100 1000 900 800 700 600 500 400 300 200 100 0 -100

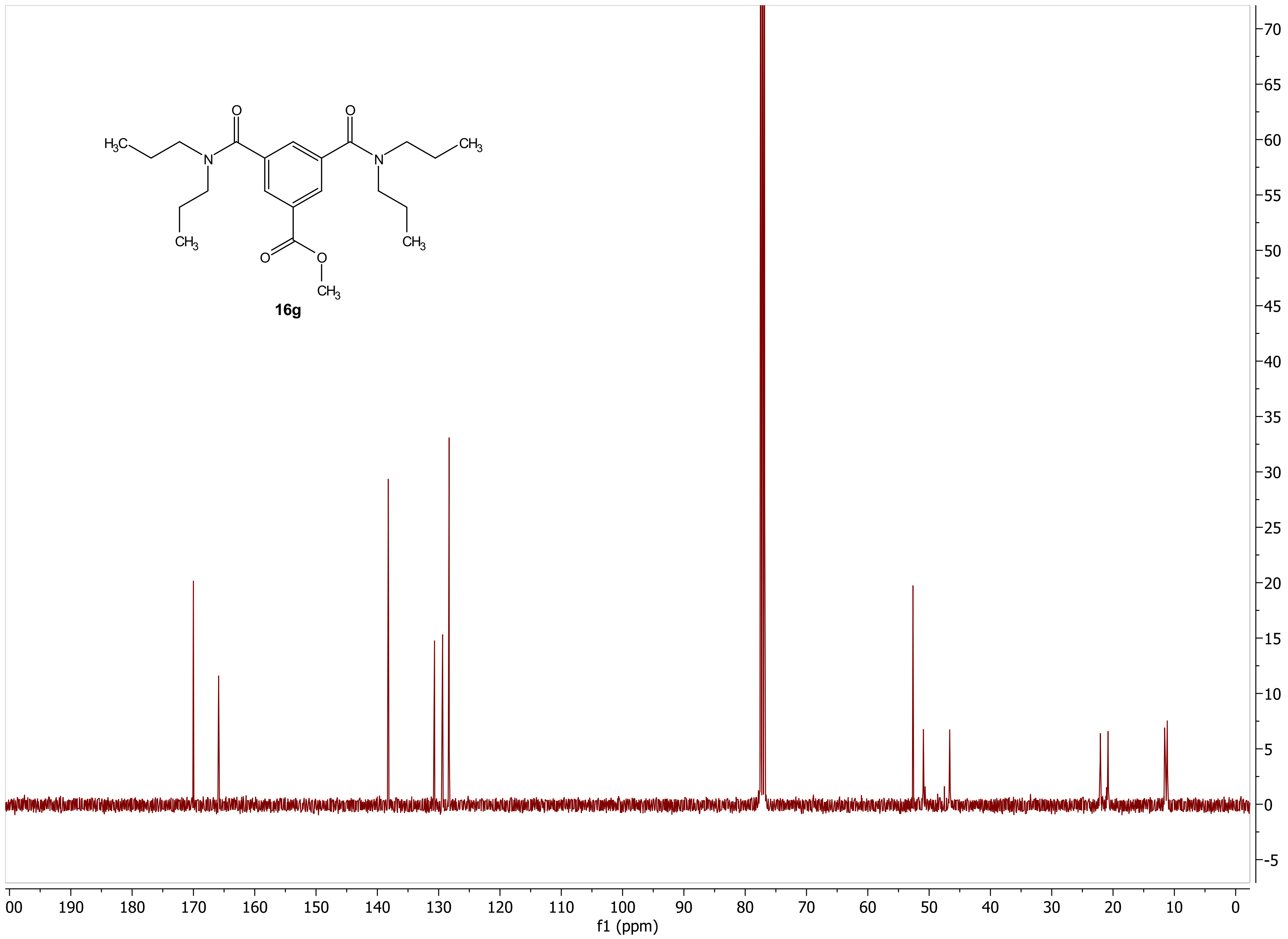

16g
H3C
CH3
N
O
f1 (ppm)

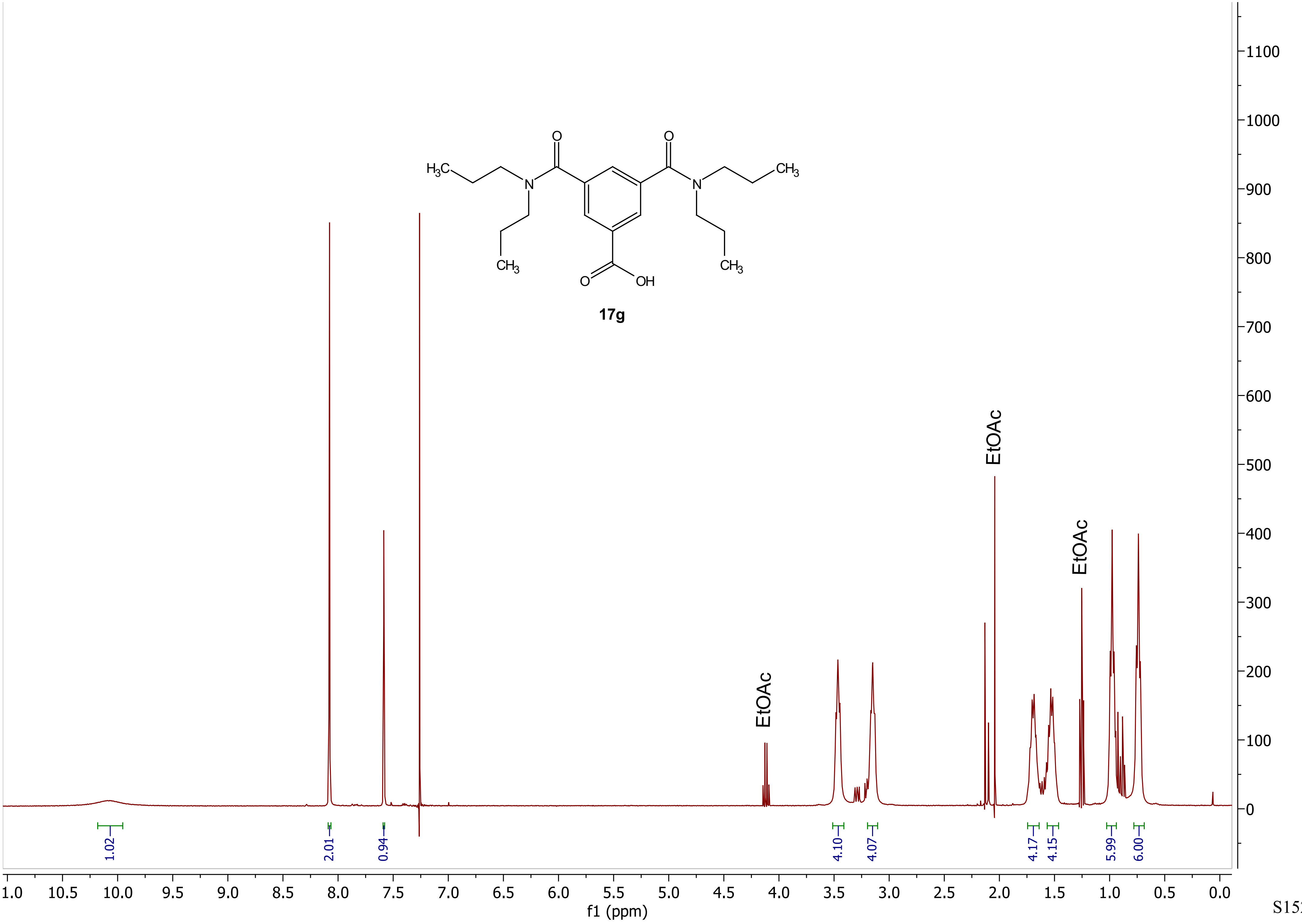
17g
O
O
H_3C
N
N
CH_3
CH_3
CH_3
O
OH
EtOAc
EtOAc
EtOAc
1.02
2.01
0.94
4.10
4.07
4.17
4.15
5.99
6.00
1.0 10.5 10.0 9.5 9.0 8.5 8.0 7.5 7.0 6.5 6.0 5.5 5.0 4.5 4.0 3.5 3.0 2.5 2.0 1.5 1.0 0.5 0.0
f1 (ppm)
1100
1000
900
800
700
600
500
400
300
200
100
0

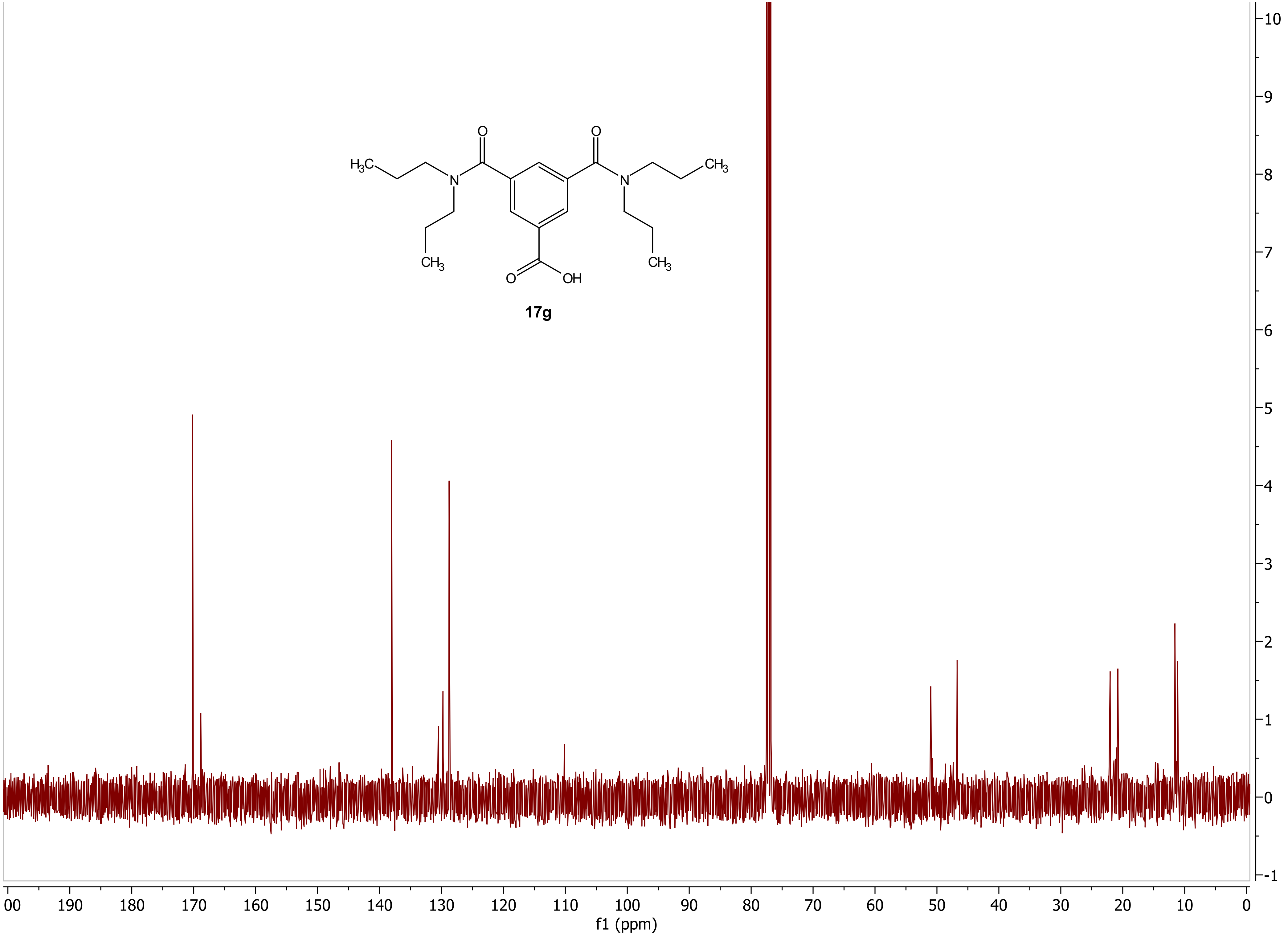

H3C
CH3
CH3
CH3
O
O
O
OH
N
N
17g
f1 (ppm)

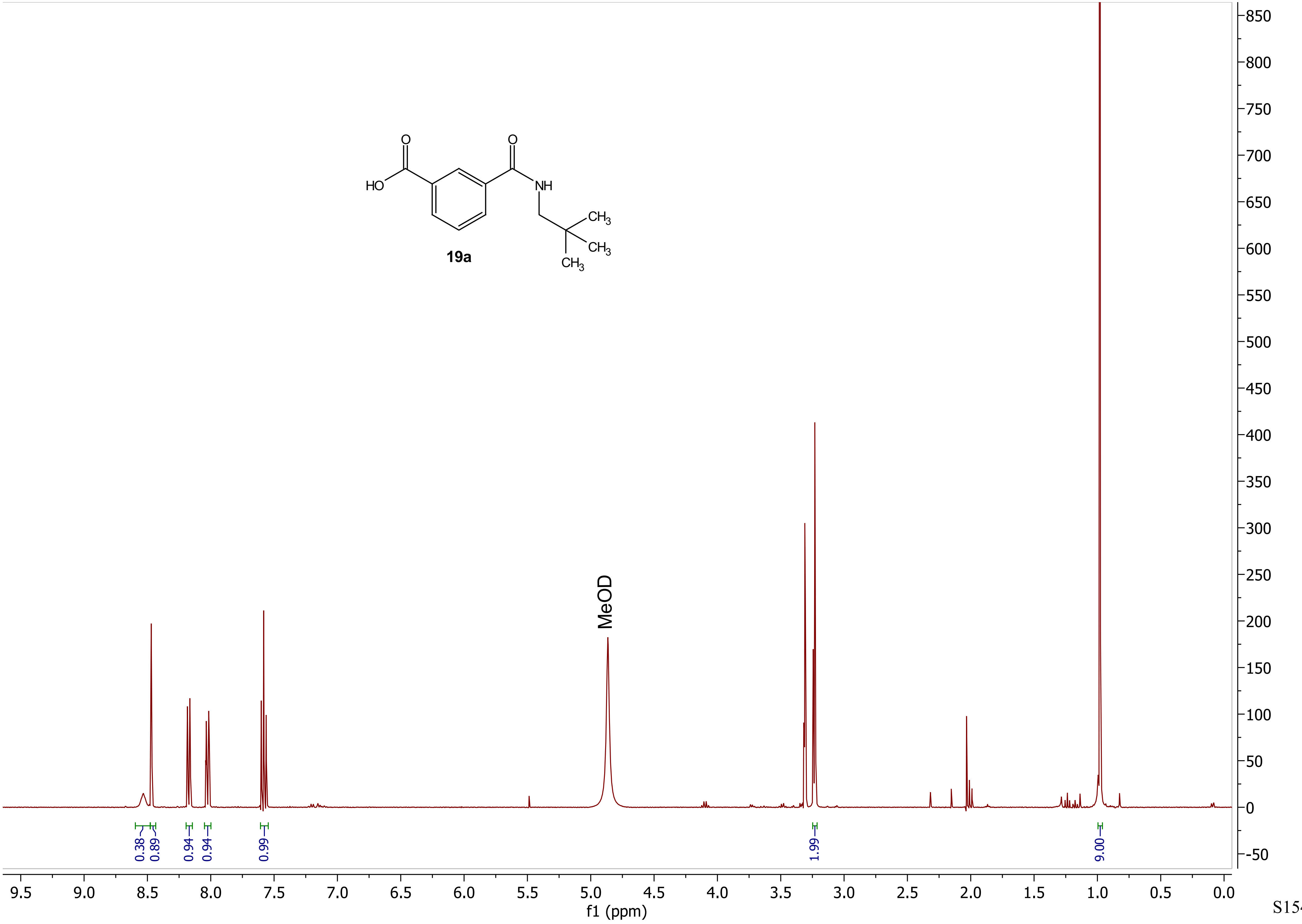

O
O
HO
NH
CH3
CH3
CH3
19a
MeOD
0.38
0.89
0.94
0.94
0.99
1.99
9.00
9.5 9.0 8.5 8.0 7.5 7.0 6.5 6.0 5.5 5.0 4.5 4.0 3.5 3.0 2.5 2.0 1.5 1.0 0.5 0.0
f1 (ppm)
850 800 750 700 650 600 550 500 450 400 350 300 250 200 150 100 50 0 -50

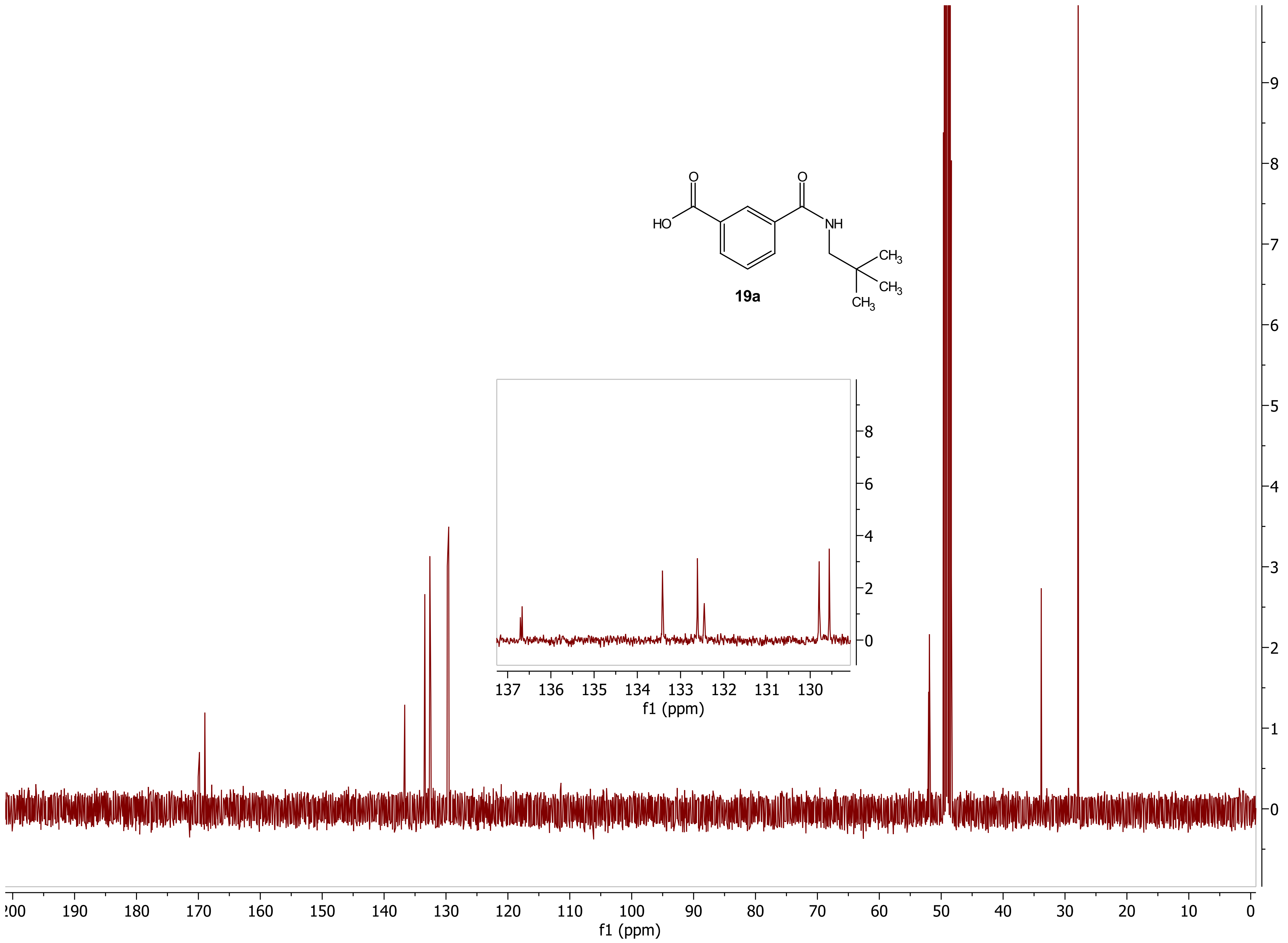
HO
O
O
NH
CH3
CH3
CH3
19a
137 136 135 134 133 132 131 130
f1 (ppm)
200 190 180 170 160 150 140 130 120 110 100 90 80 70 60 50 40 30 20 10 0
f1 (ppm)

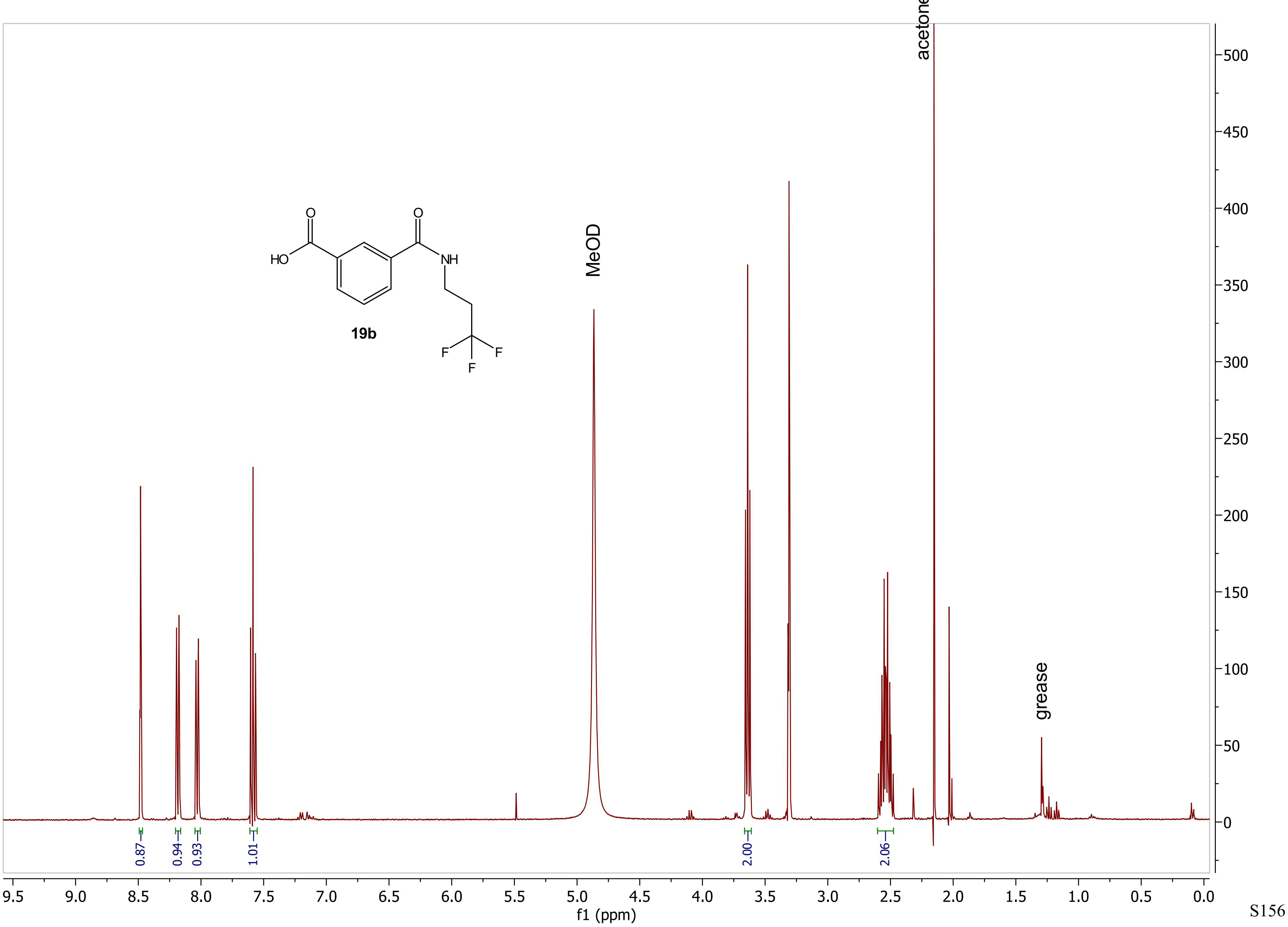
19b
HO
O
NH
F
F
F
MeOD
acetone
grease
0.87
0.94
0.93
1.01
2.00
2.06
f1 (ppm)

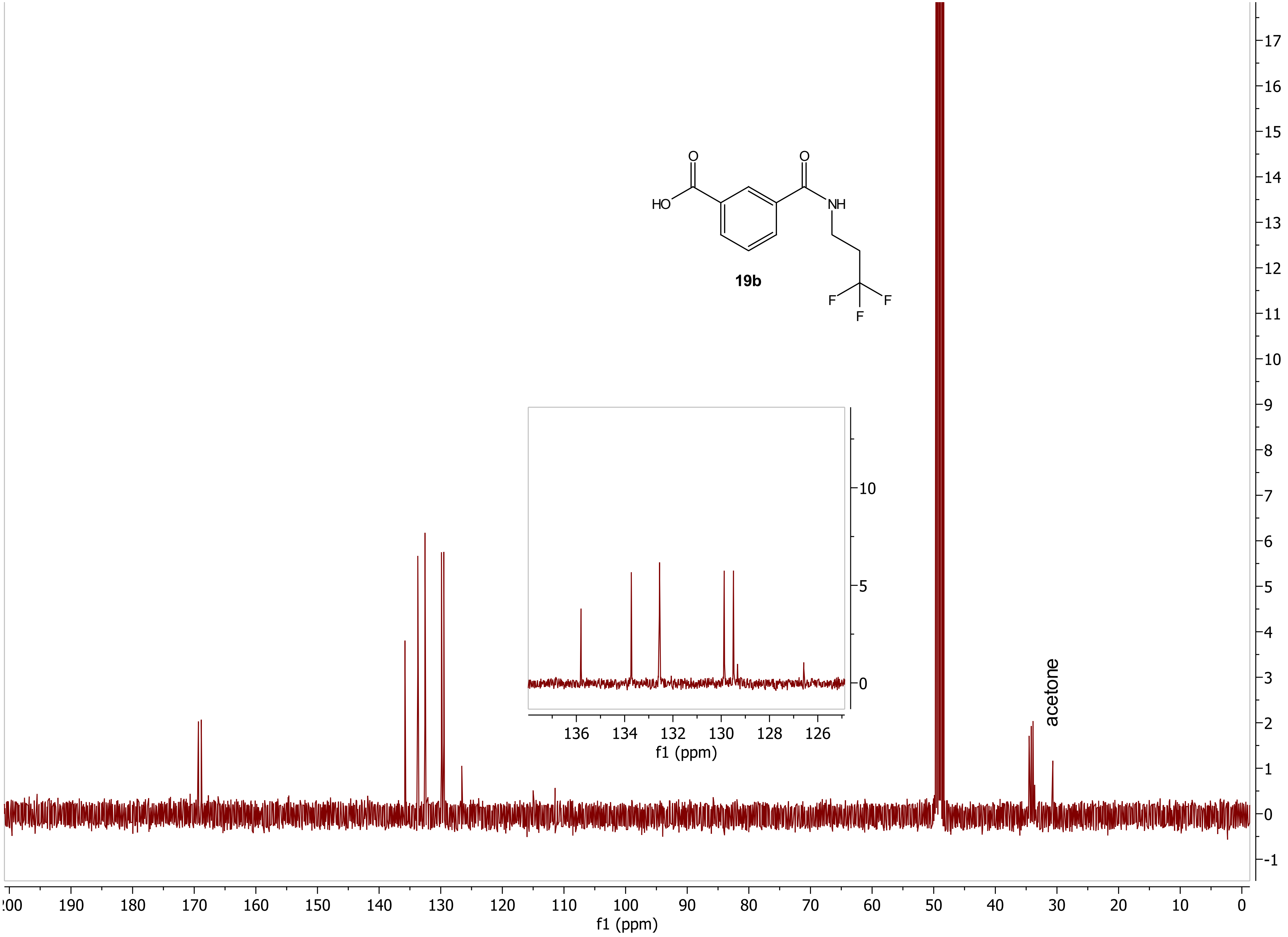

19b
HO
O
NH
F
acetone
136
134
132
130
128
126
f1 (ppm)
f1 (ppm)

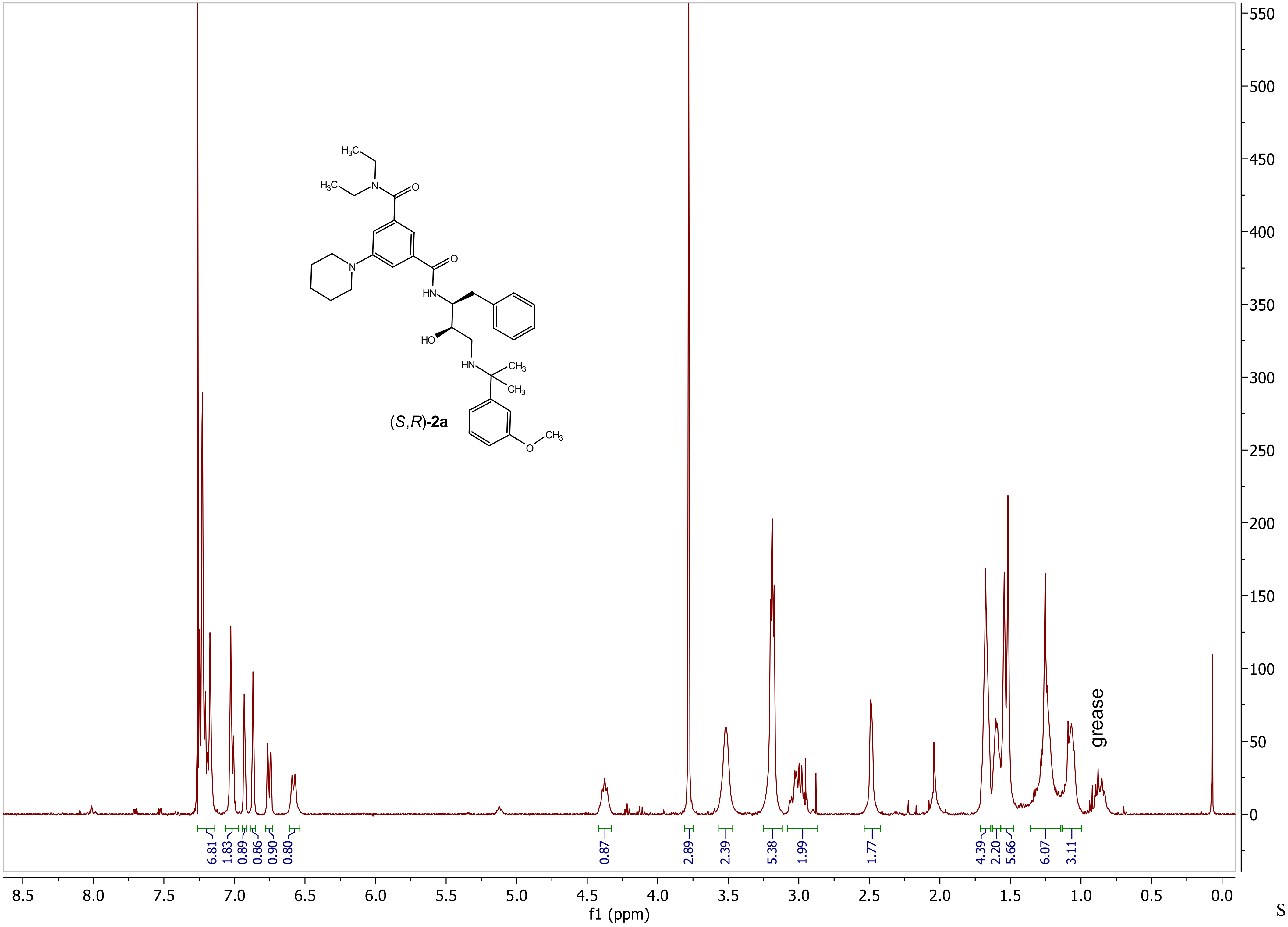
(S,R)-2a
grease
6.81
1.83
0.89
0.86
0.90
0.80
0.87
2.89
2.39
5.38
1.99
1.77
4.39
2.20
5.66
6.07
3.11
8.5 8.0 7.5 7.0 6.5 6.0 5.5 5.0 4.5 4.0 3.5 3.0 2.5 2.0 1.5 1.0 0.5 0.0
f1 (ppm)
550 500 450 400 350 300 250 200 150 100 50 0

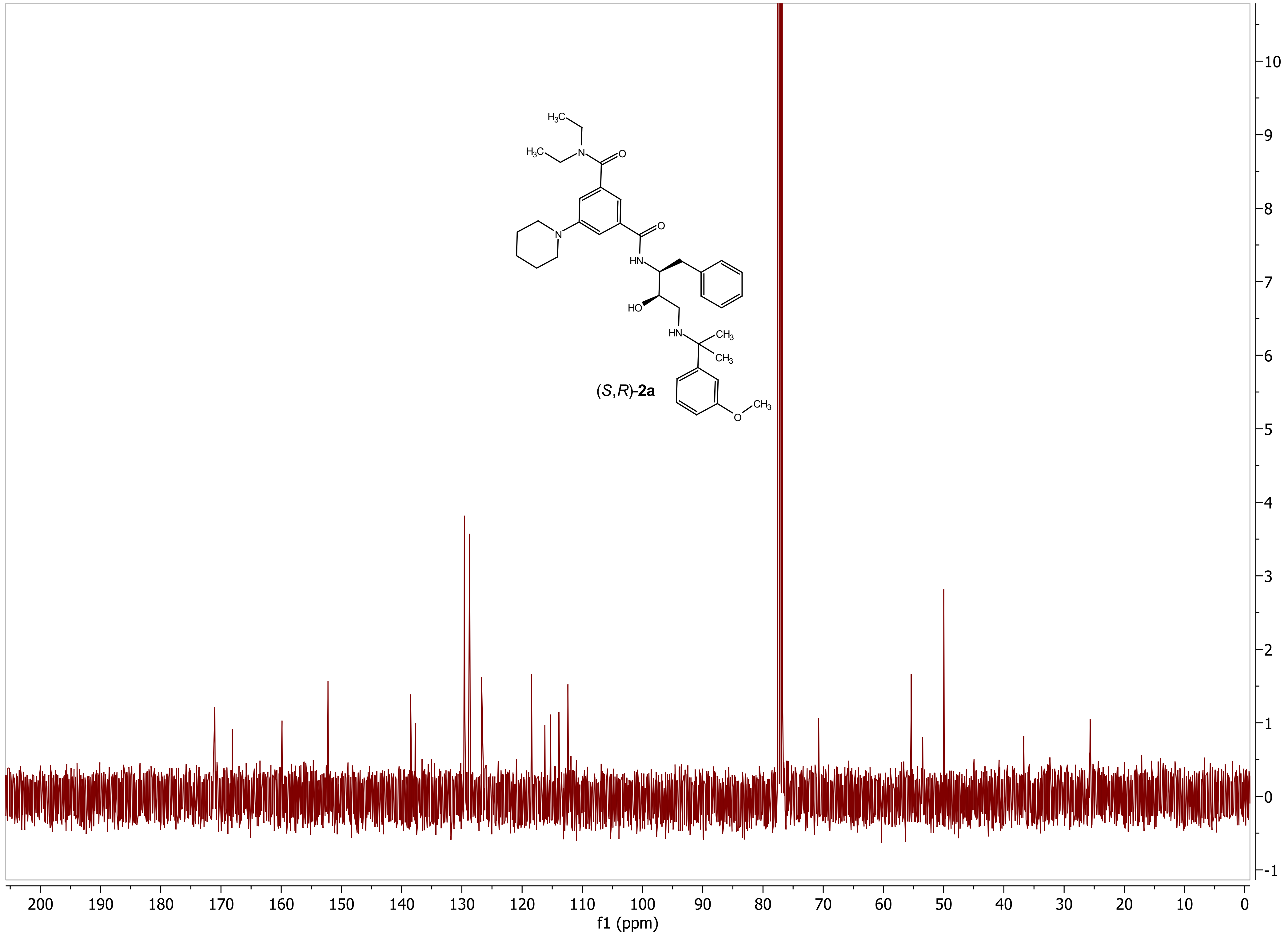
(S,R)-2a
H3C
H3C
N
O
N
O
HN
HO
HN
CH3
CH3
O
CH3
200 190 180 170 160 150 140 130 120 110 100 90 80 70 60 50 40 30 20 10 0
f1 (ppm)
10 9 8 7 6 5 4 3 2 1 0 -1

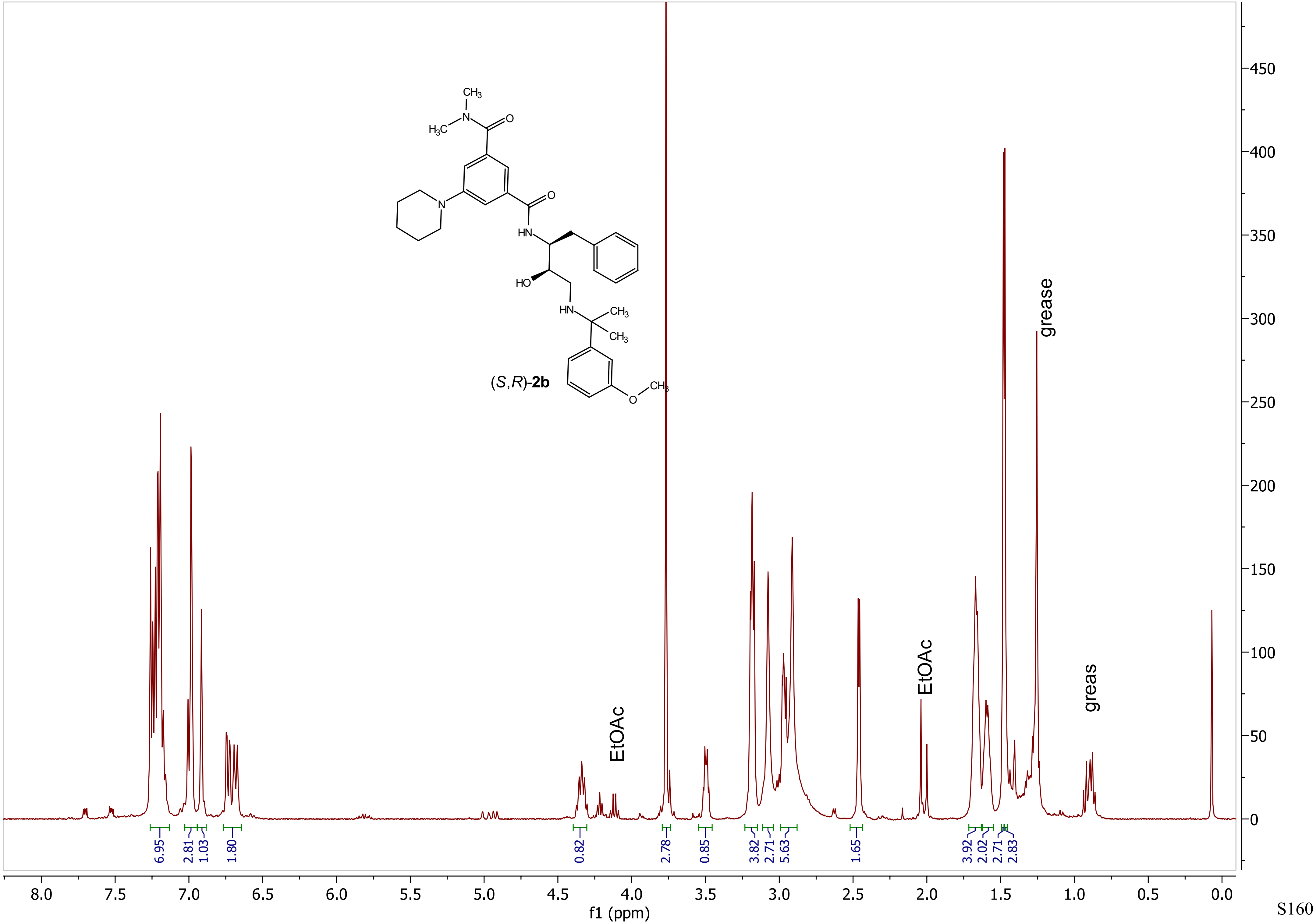
(S,R)-2b
CH3
H3C
N
O
N
O
HN
HO
HN
CH3
CH3
O
CH3
EtOAc
EtOAc
grease
greas
6.95
2.81
1.03
1.80
0.82
2.78
0.85
3.82
2.71
5.63
1.65
3.92
2.02
2.71
2.83
8.0
7.5
7.0
6.5
6.0
5.5
5.0
4.5
4.0
3.5
3.0
2.5
2.0
1.5
1.0
0.5
0.0
f1 (ppm)
450
400
350
300
250
200
150
100
50
0

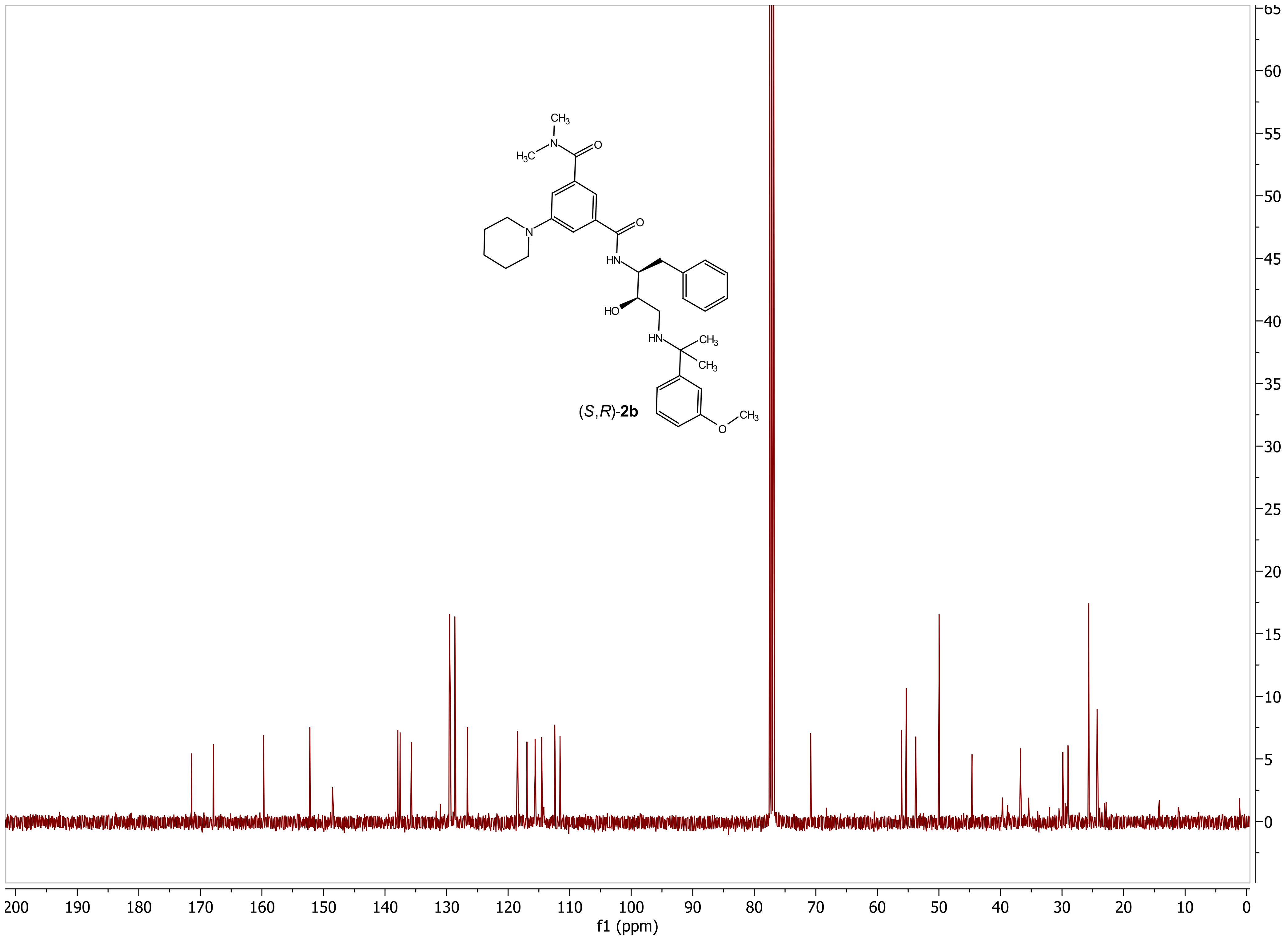

CH3
N
H3C
O
N
O
HN
HO
HN
CH3
CH3
(S,R)-2b
O
CH3
f1 (ppm)

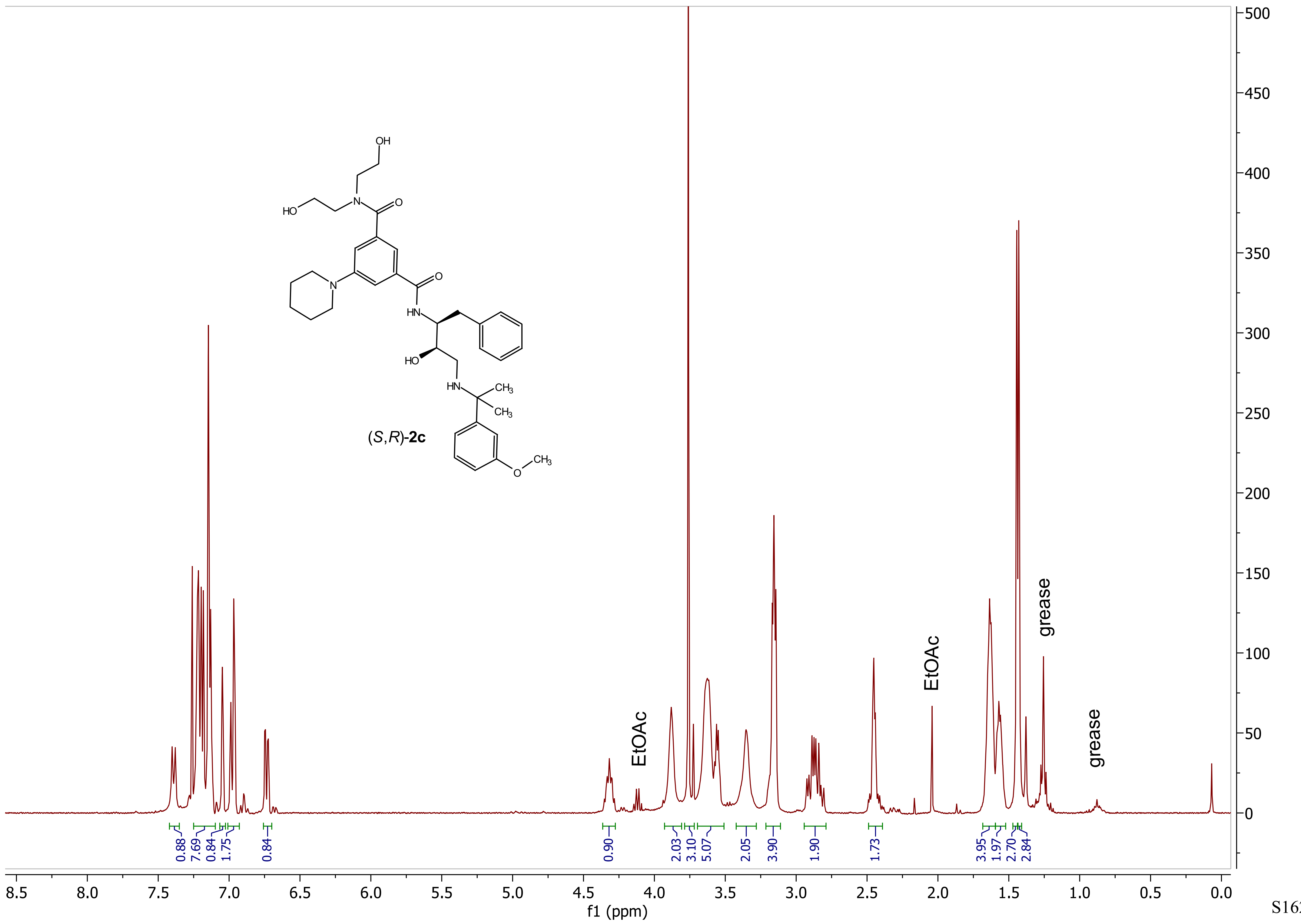
OH
HO
N
O
N
O
HN
HO
HN
CH3
CH3
O
CH3
(S,R)-2c
EtOAc
EtOAc
grease
grease
0.88
7.69
0.84
1.75
0.84
0.90
2.03
3.10
5.07
2.05
3.90
1.90
1.73
3.95
1.97
2.70
2.84
f1 (ppm)

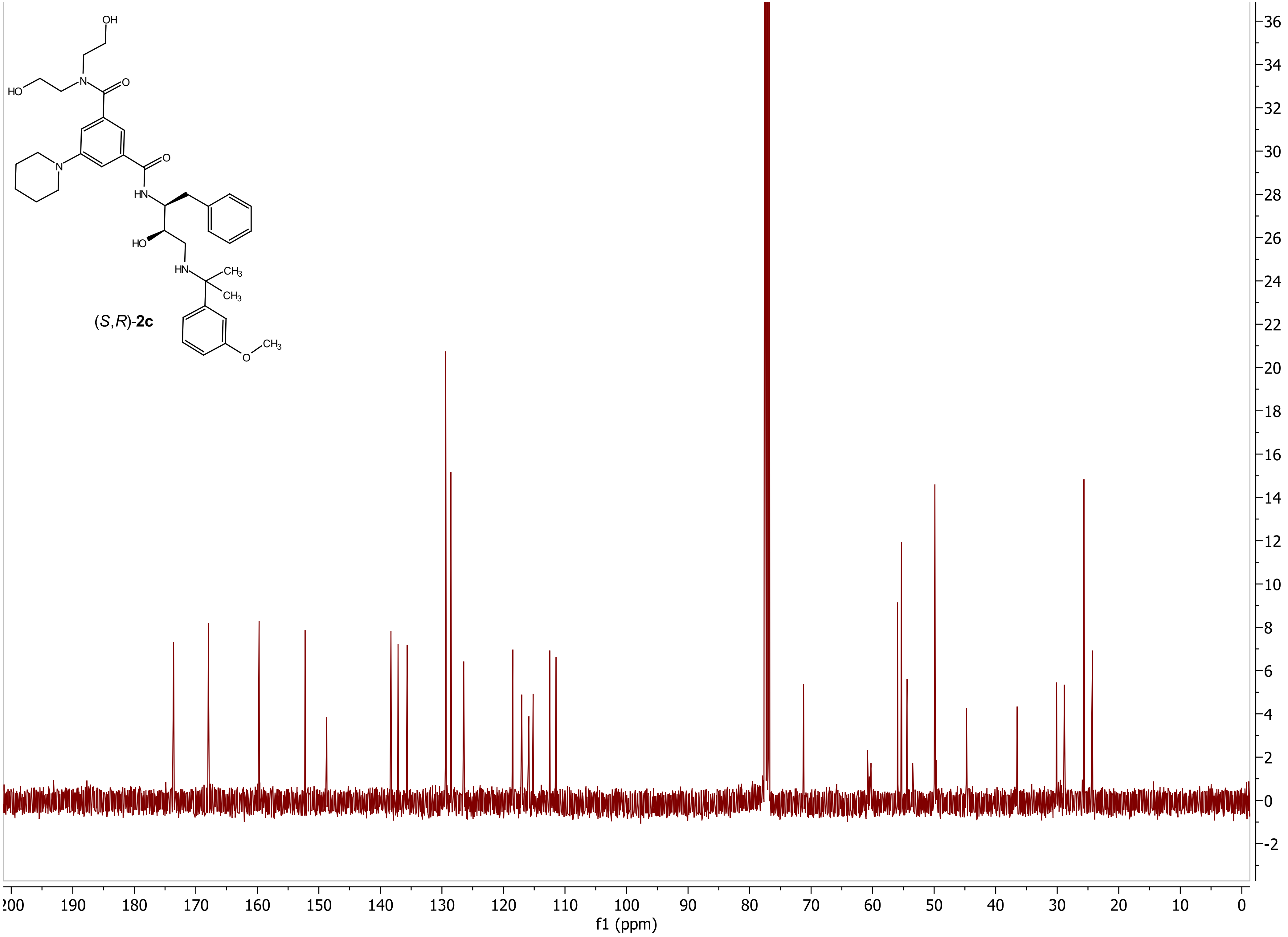
OH
HO
N
O
O
N
HN
HO
HN
CH3
CH3
O
CH3
200 190 180 170 160 150 140 130 120 110 100 90 80 70 60 50 40 30 20 10 0
f1 (ppm)
36 34 32 30 28 26 24 22 20 18 16 14 12 10 8 6 4 2 0 -2

(*S*,*R*)-**2c**

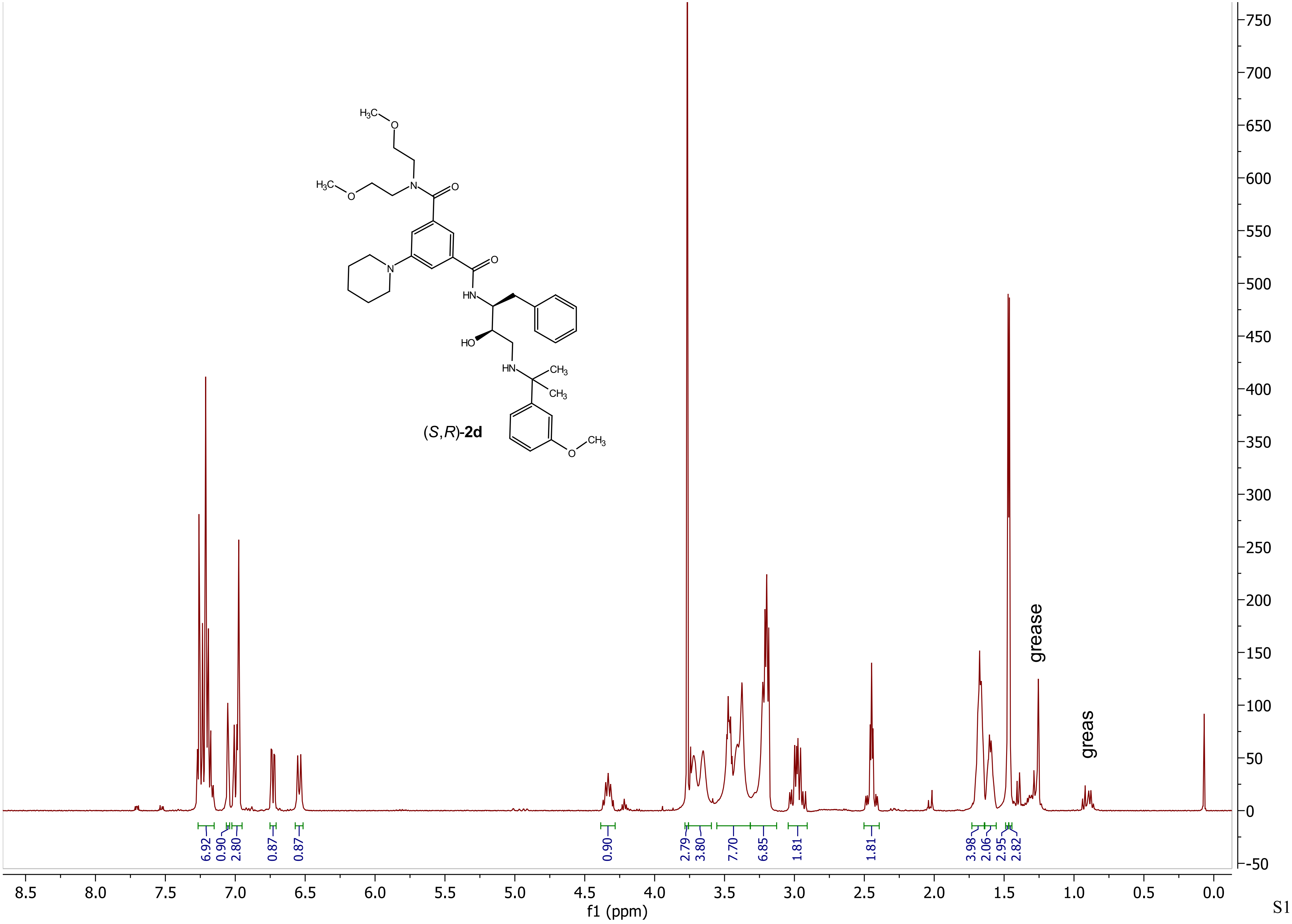

H3C
O
H3C
O
N
O
N
O
HN
HO
HN
CH3
CH3
O
CH3
(S,R)-2d
grease
greas
6.92
0.90
2.80
0.87
0.87
0.90
2.79
3.80
7.70
6.85
1.81
1.81
3.98
2.06
2.95
2.82
f1 (ppm)

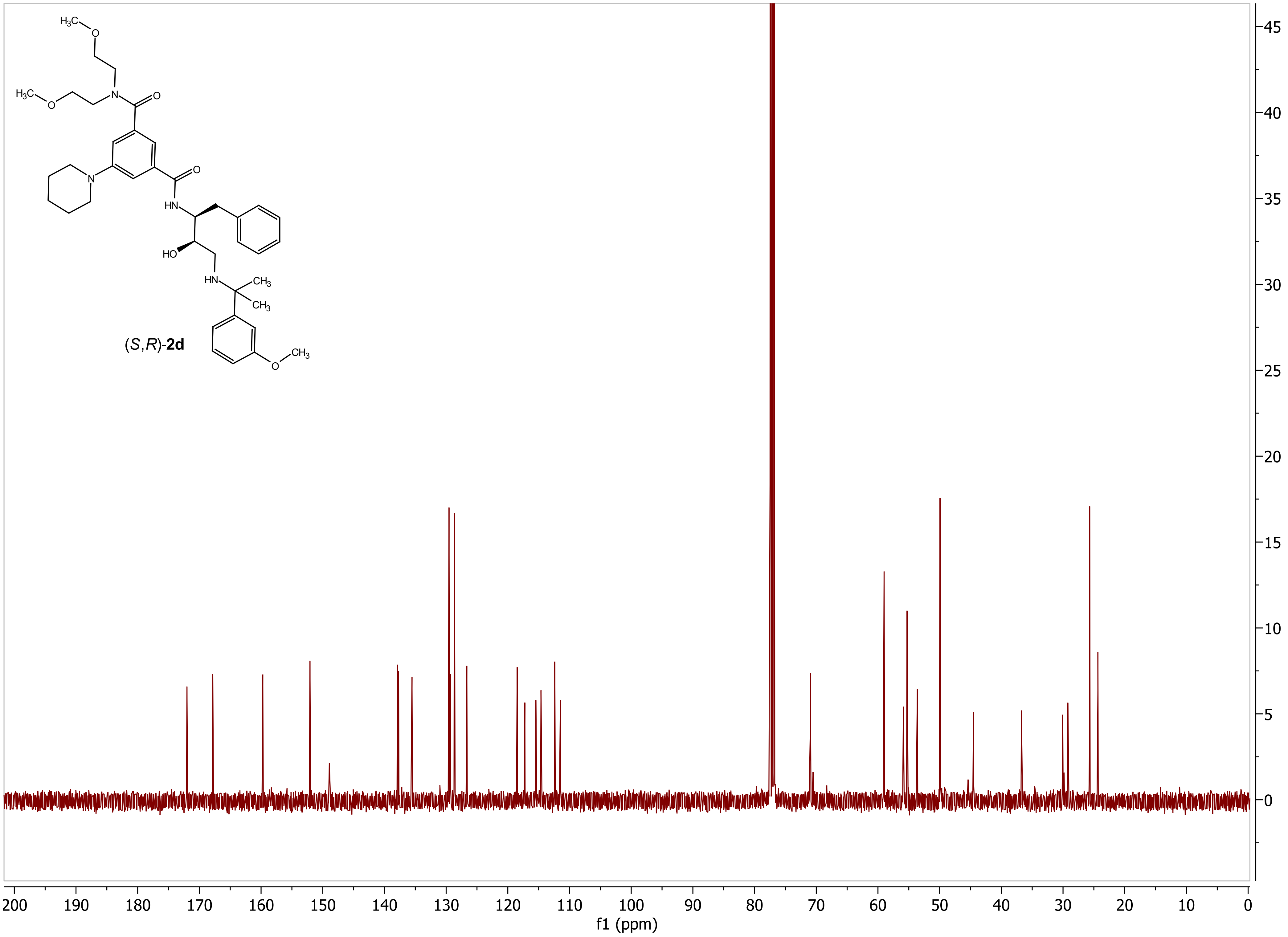

(S,R)-2d
f1 (ppm)

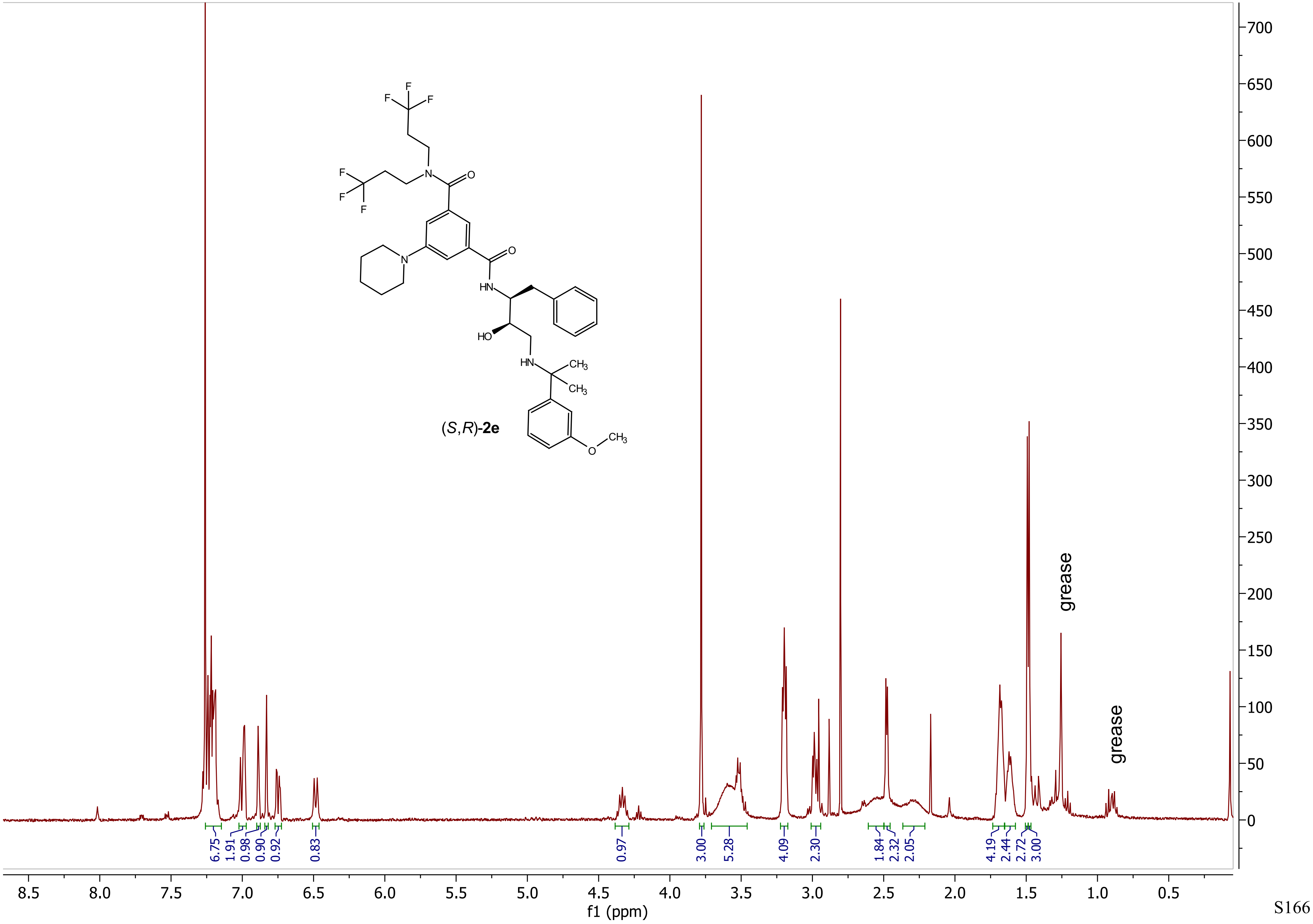

(S,R)-2e
grease
grease
6.75
1.91
0.98
0.90
0.92
0.83
0.97
3.00
5.28
4.09
2.30
1.84
2.32
2.05
4.19
2.44
2.72
3.00
f1 (ppm)

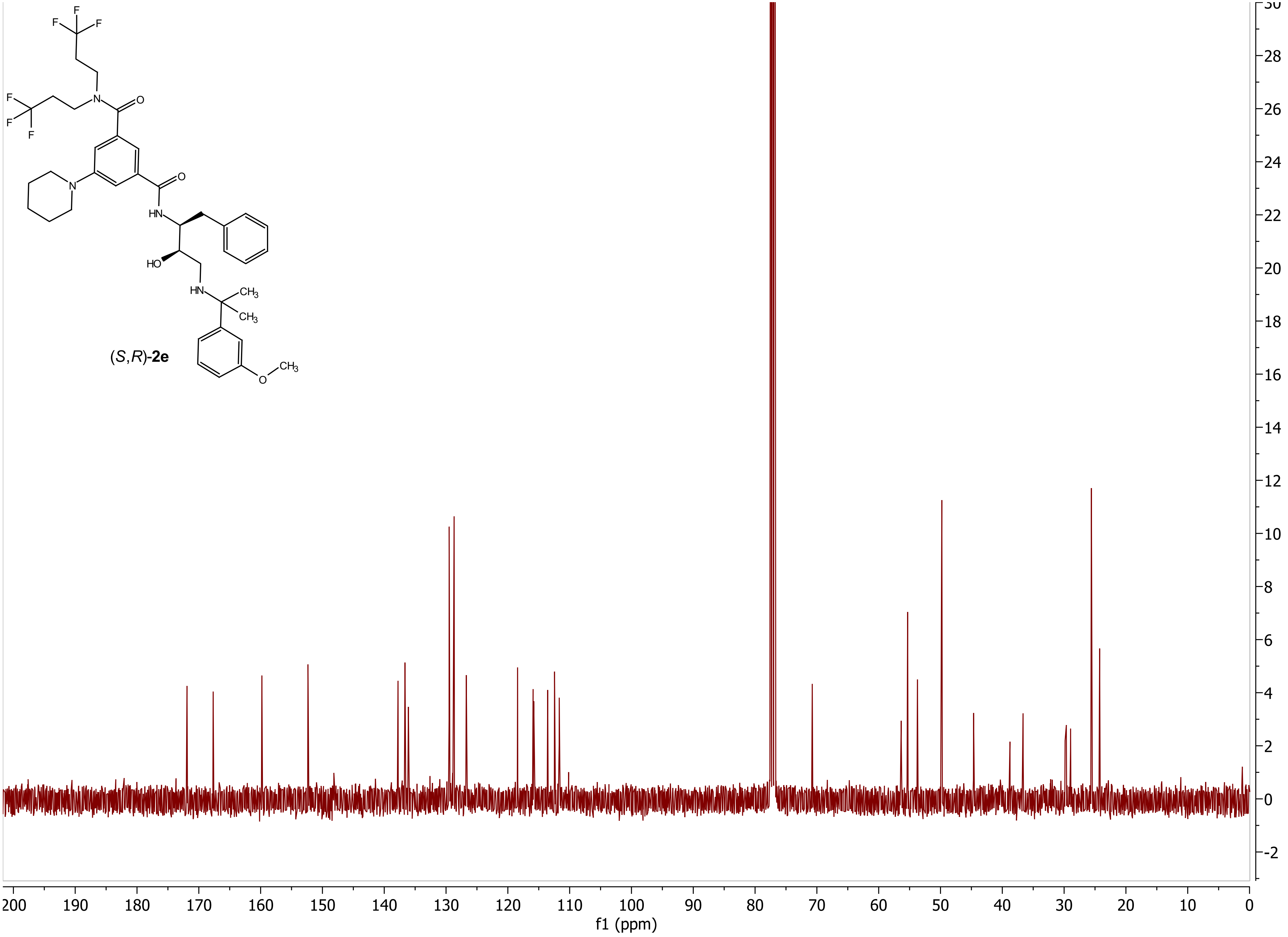
(S,R)-2e
f1 (ppm)

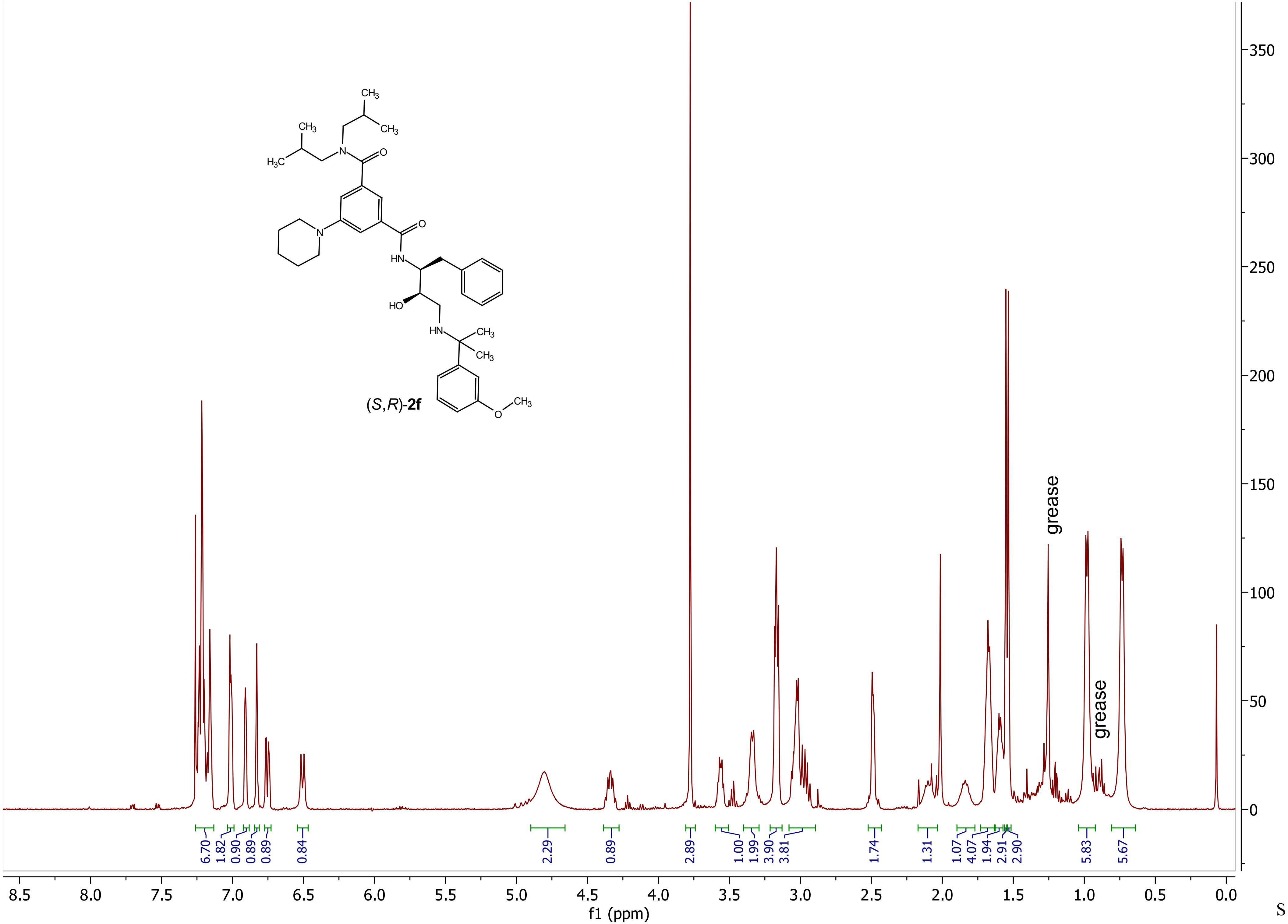

CH3
CH3
CH3
H3C
N
O
O
N
HN
HO
HN
CH3
CH3
O
CH3
(S,R)-2f
grease
grease
6.70
1.82
0.90
0.89
0.89
0.84
2.29
0.89
2.89
1.00
1.99
3.90
3.81
1.74
1.31
1.07
4.07
1.94
2.91
2.90
5.83
5.67
8.5
8.0
7.5
7.0
6.5
6.0
5.5
5.0
4.5
4.0
3.5
3.0
2.5
2.0
1.5
1.0
0.5
0.0
f1 (ppm)
350
300
250
200
150
100
50
0

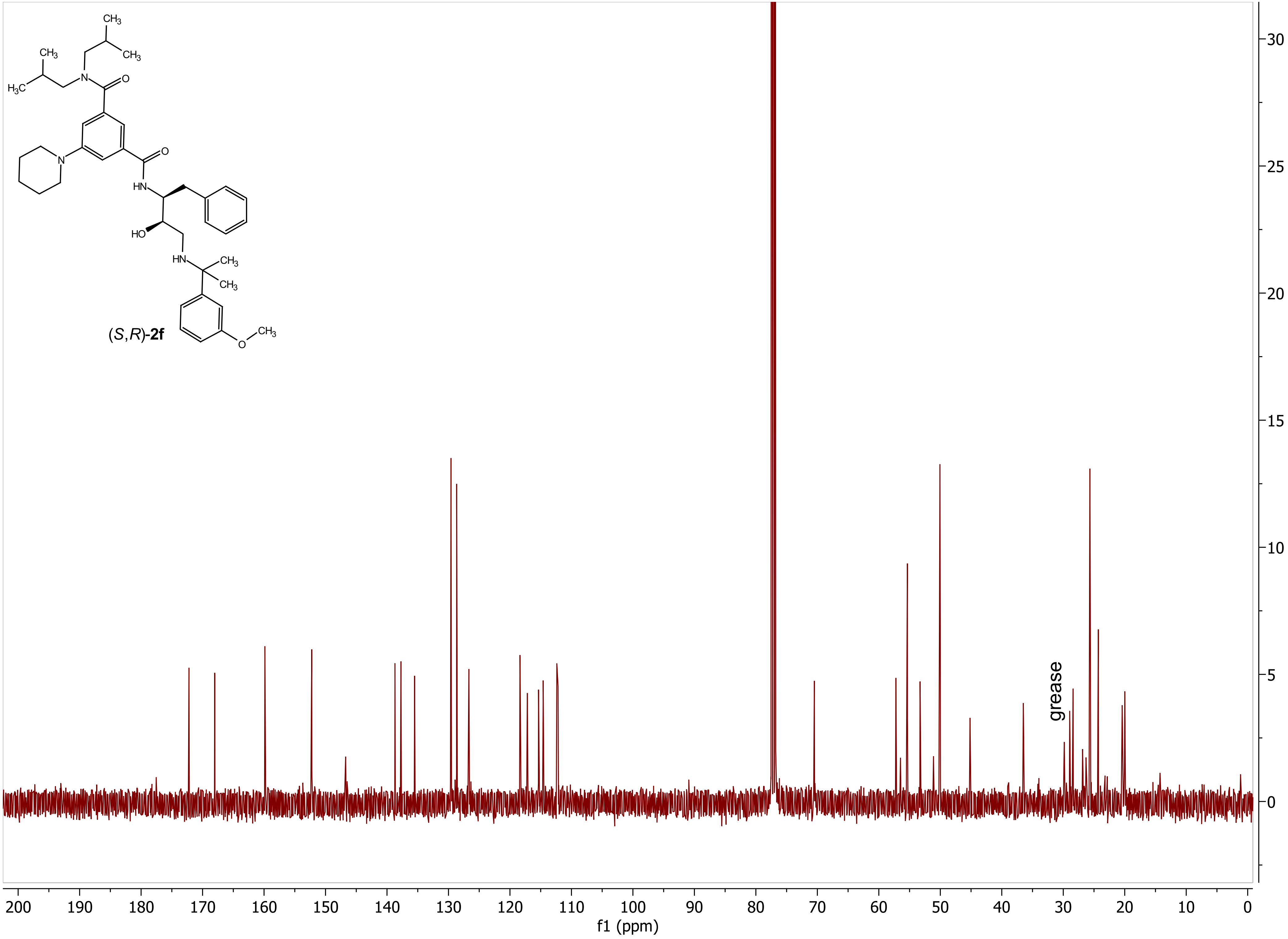
(S,R)-2f
grease
f1 (ppm)

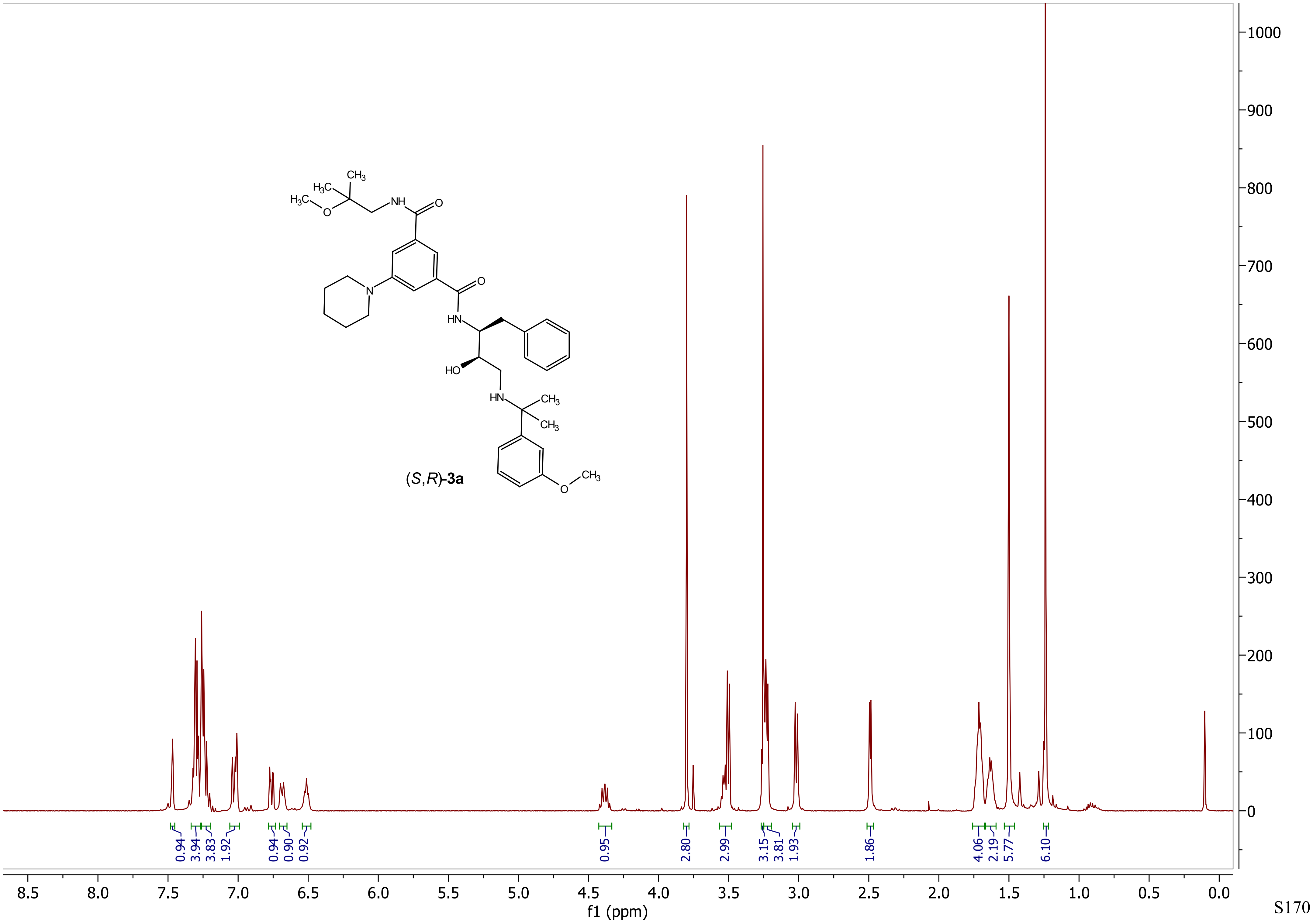

(S,R)-3a
f1 (ppm)
0.94
3.94
3.83
1.92
0.94
0.90
0.92
0.95
2.80
2.99
3.15
3.81
1.93
1.86
4.06
2.19
5.77
6.10

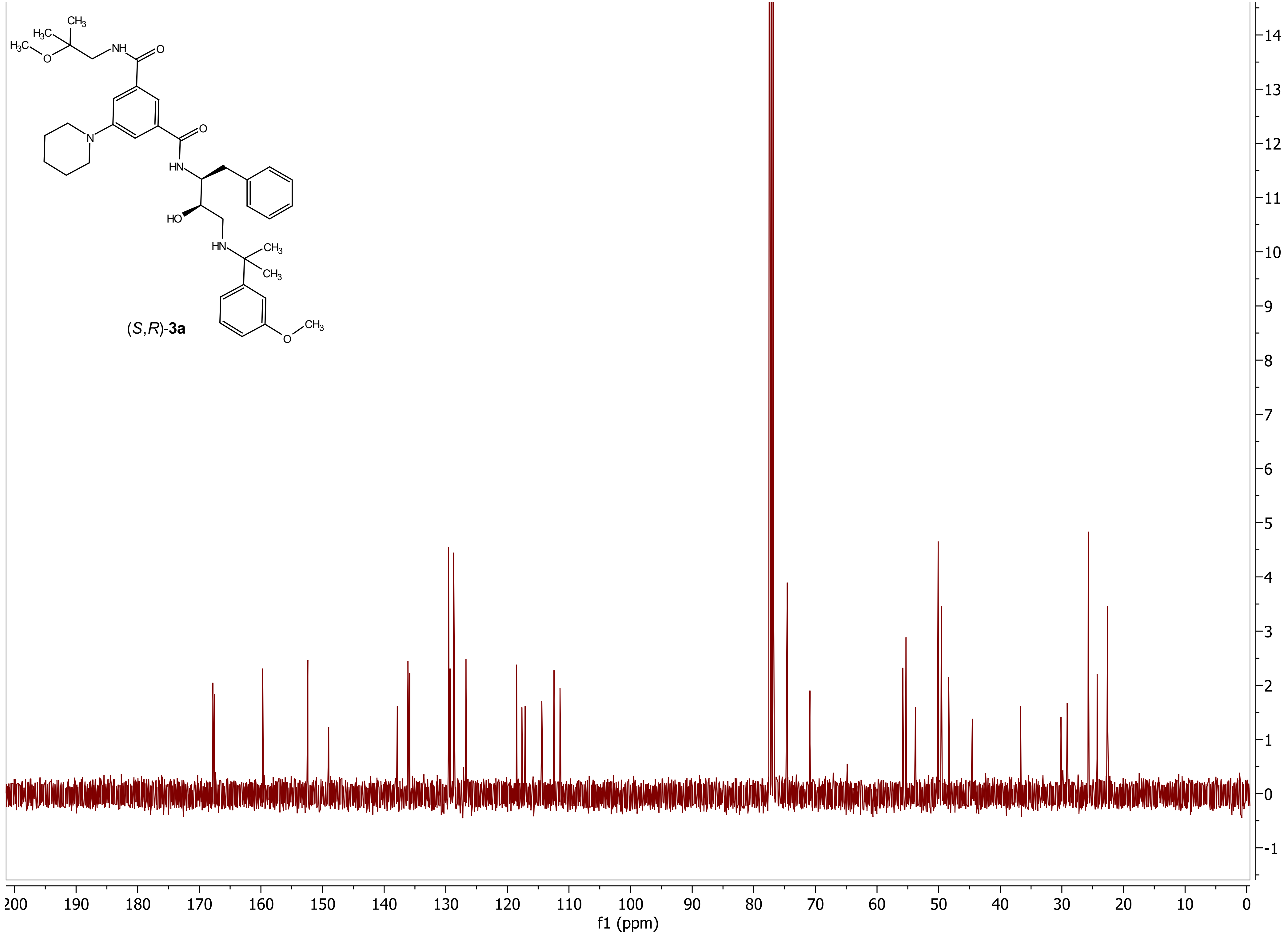
(S,R)-3a
f1 (ppm)
200
190
180
170
160
150
140
130
120
110
100
90
80
70
60
50
40
30
20
10
0
14
13
12
11
10
9
8
7
6
5
4
3
2
1
0
-1

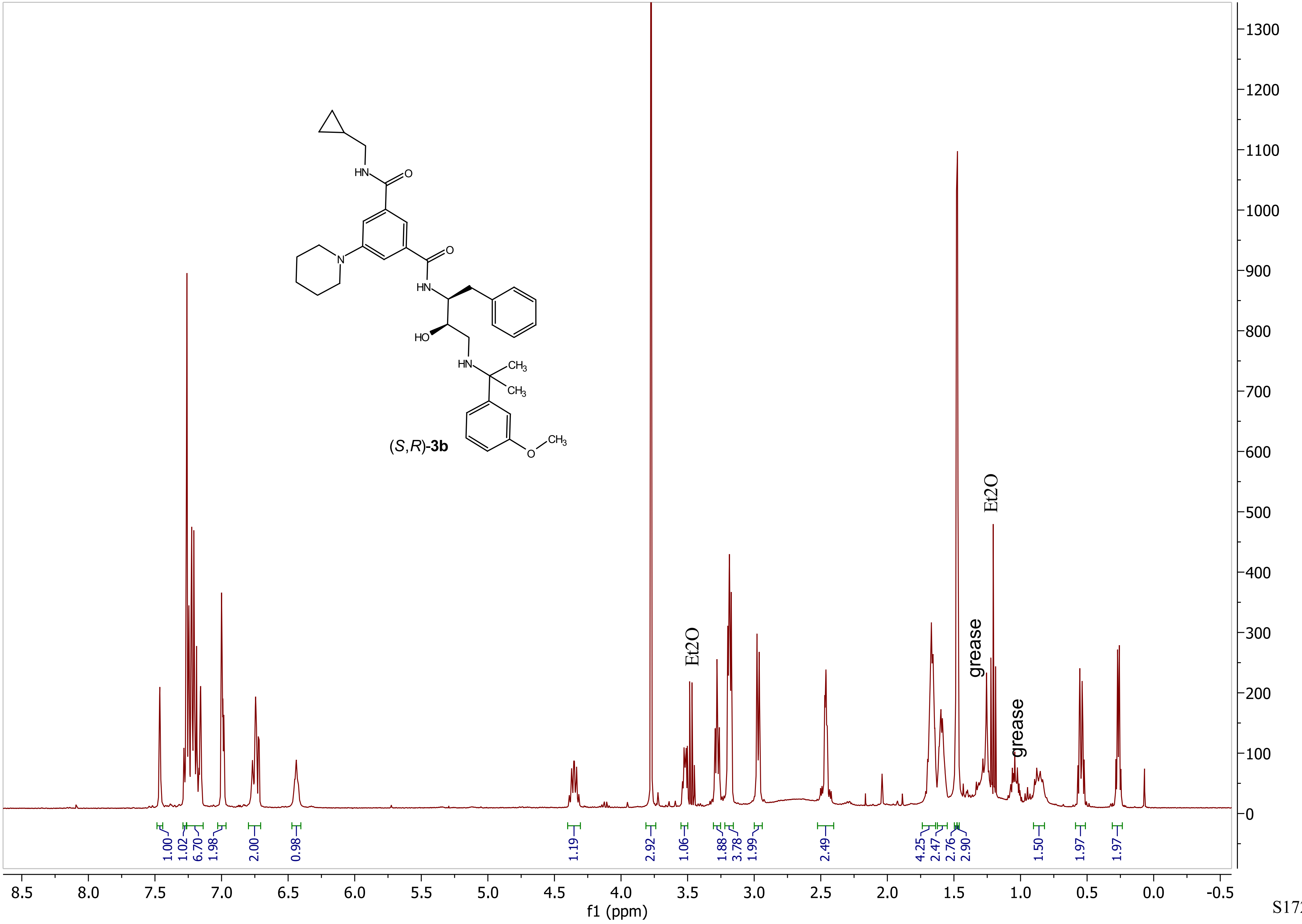

HN
O
N
O
HN
HO
HN
CH3
CH3
O
CH3
(S,R)-3b
Et2O
Et2O
grease
grease
1.00
1.02
6.70
1.98
2.00
0.98
1.19
2.92
1.06
1.88
3.78
1.99
2.49
4.25
2.47
2.76
2.90
1.50
1.97
1.97
8.5
8.0
7.5
7.0
6.5
6.0
5.5
5.0
4.5
4.0
3.5
3.0
2.5
2.0
1.5
1.0
0.5
0.0
-0.5
f1 (ppm)
1300
1200
1100
1000
900
800
700
600
500
400
300
200
100
0

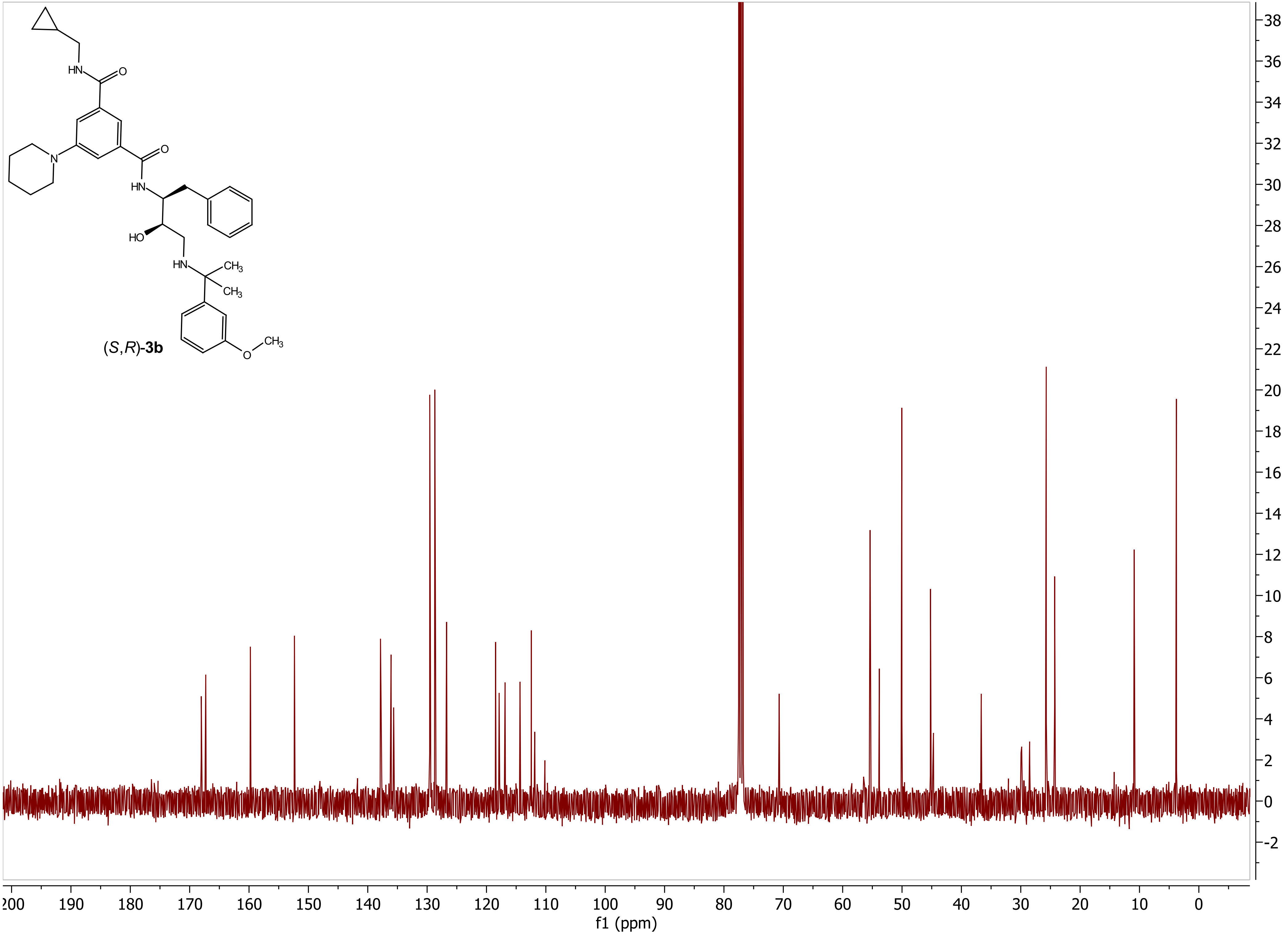

HN
O
N
O
HN
HO
HN
CH3
CH3
O
CH3
(S,R)-3b
200 190 180 170 160 150 140 130 120 110 100 90 80 70 60 50 40 30 20 10 0
f1 (ppm)
38 36 34 32 30 28 26 24 22 20 18 16 14 12 10 8 6 4 2 0 -2

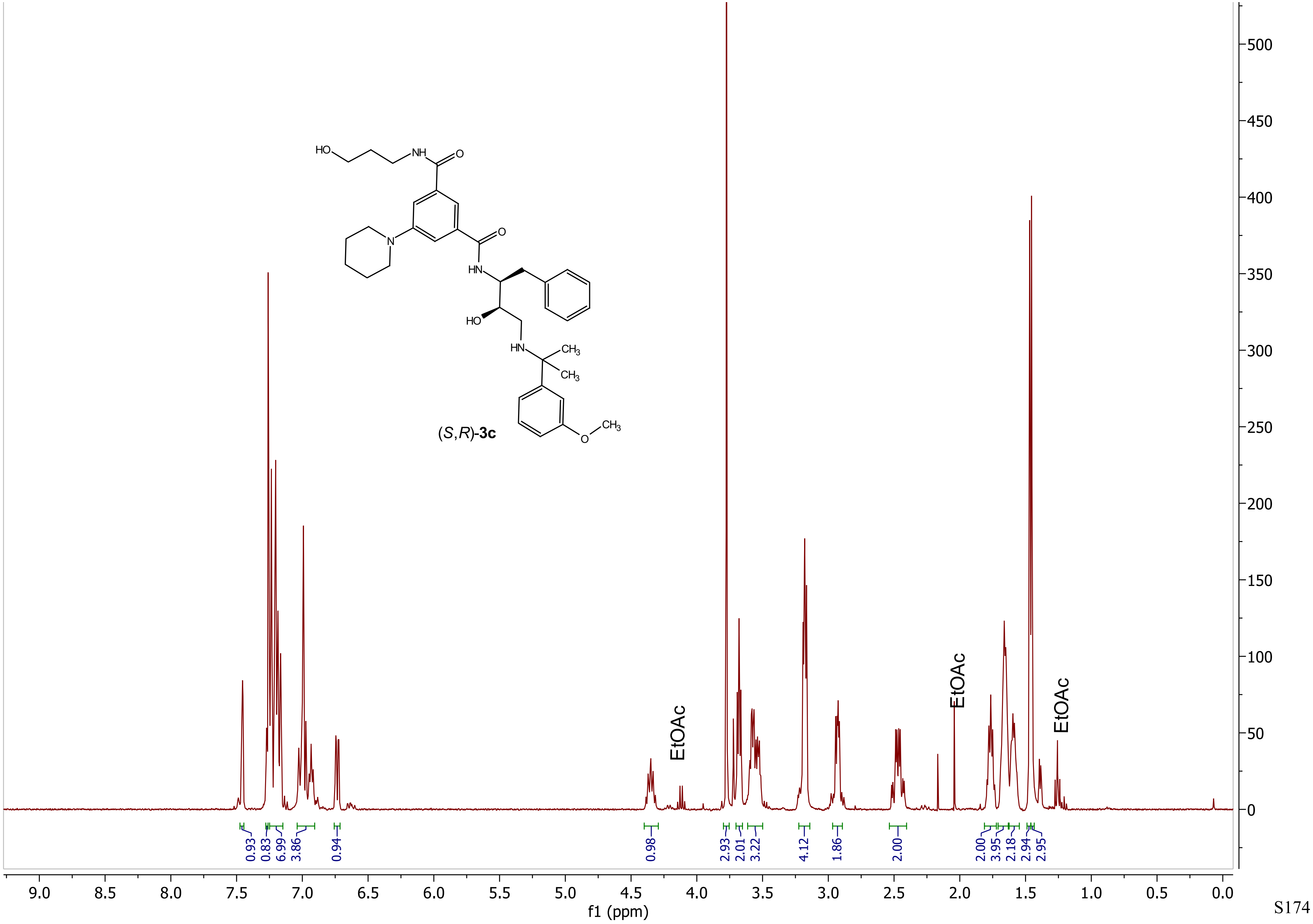

(S,R)-3c
EtOAc
EtOAc
EtOAc
f1 (ppm)

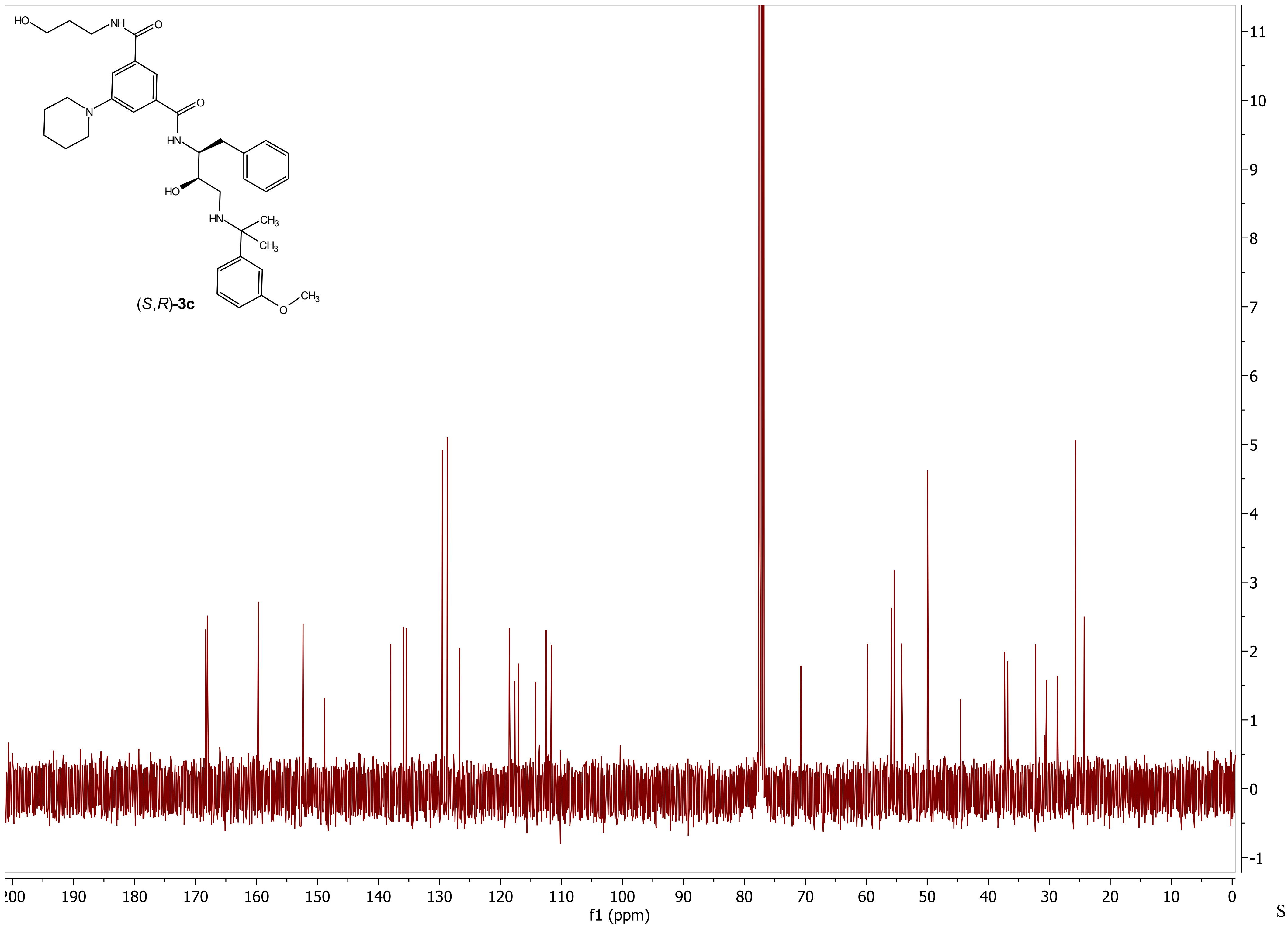

HO
NH
O
N
O
HN
HO
HN
CH3
CH3
O
CH3
(S,R)-3c
f1 (ppm)

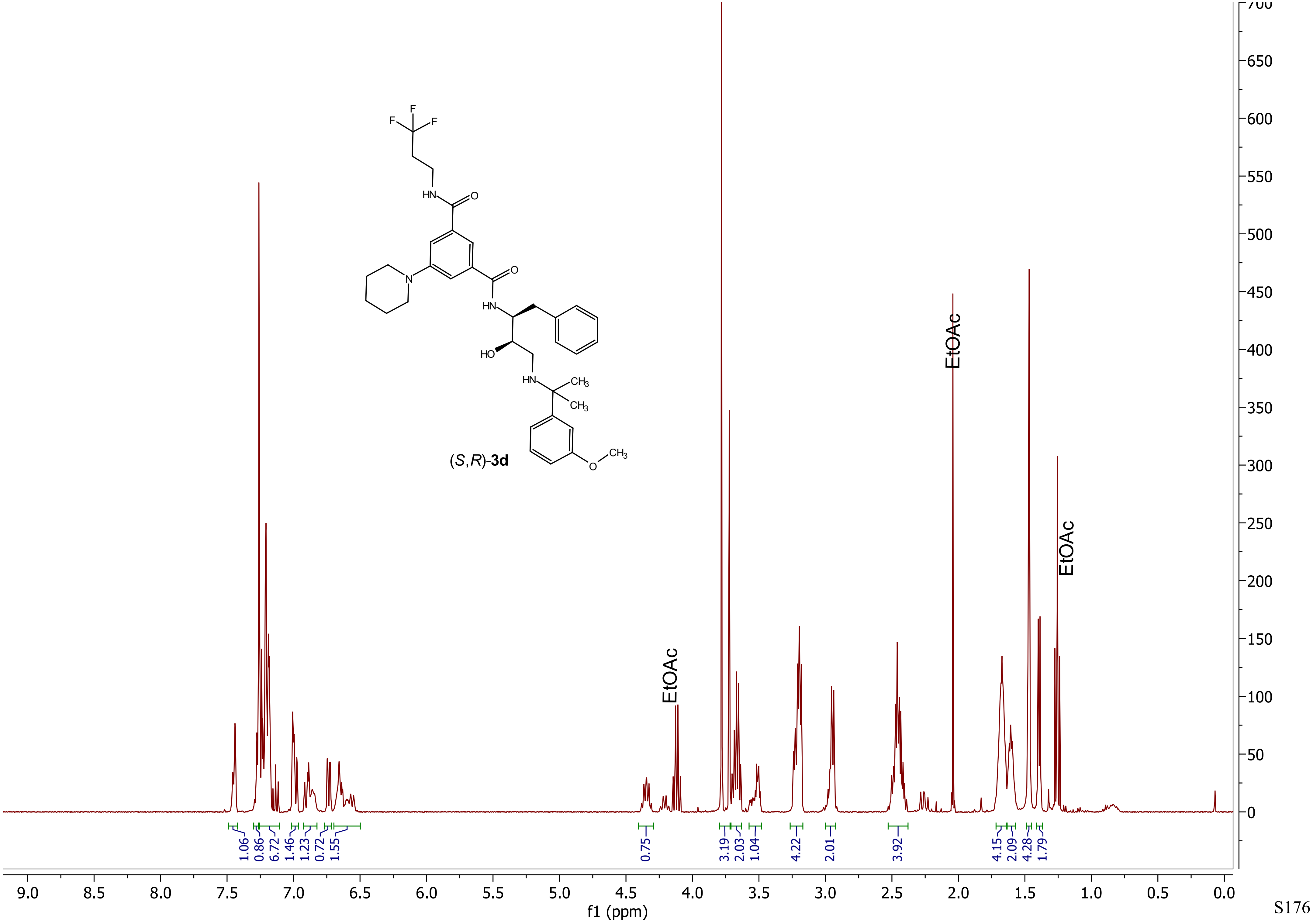
(S,R)-3d
EtOAc
EtOAc
EtOAc
1.06
0.86
6.72
1.46
1.23
0.72
1.55
0.75
3.19
2.03
1.04
4.22
2.01
3.92
4.15
2.09
4.28
1.79
f1 (ppm)

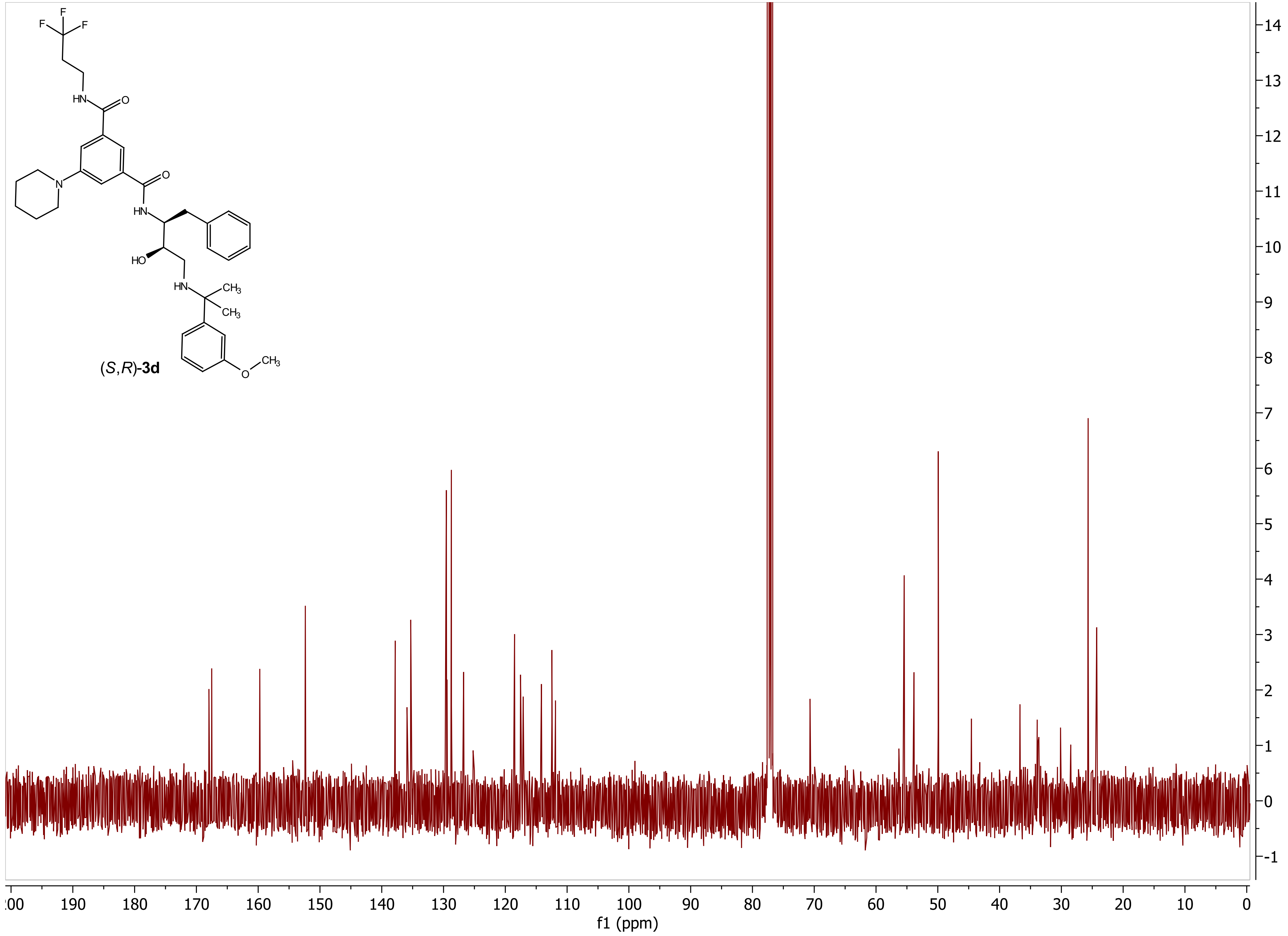
(S,R)-3d
f1 (ppm)

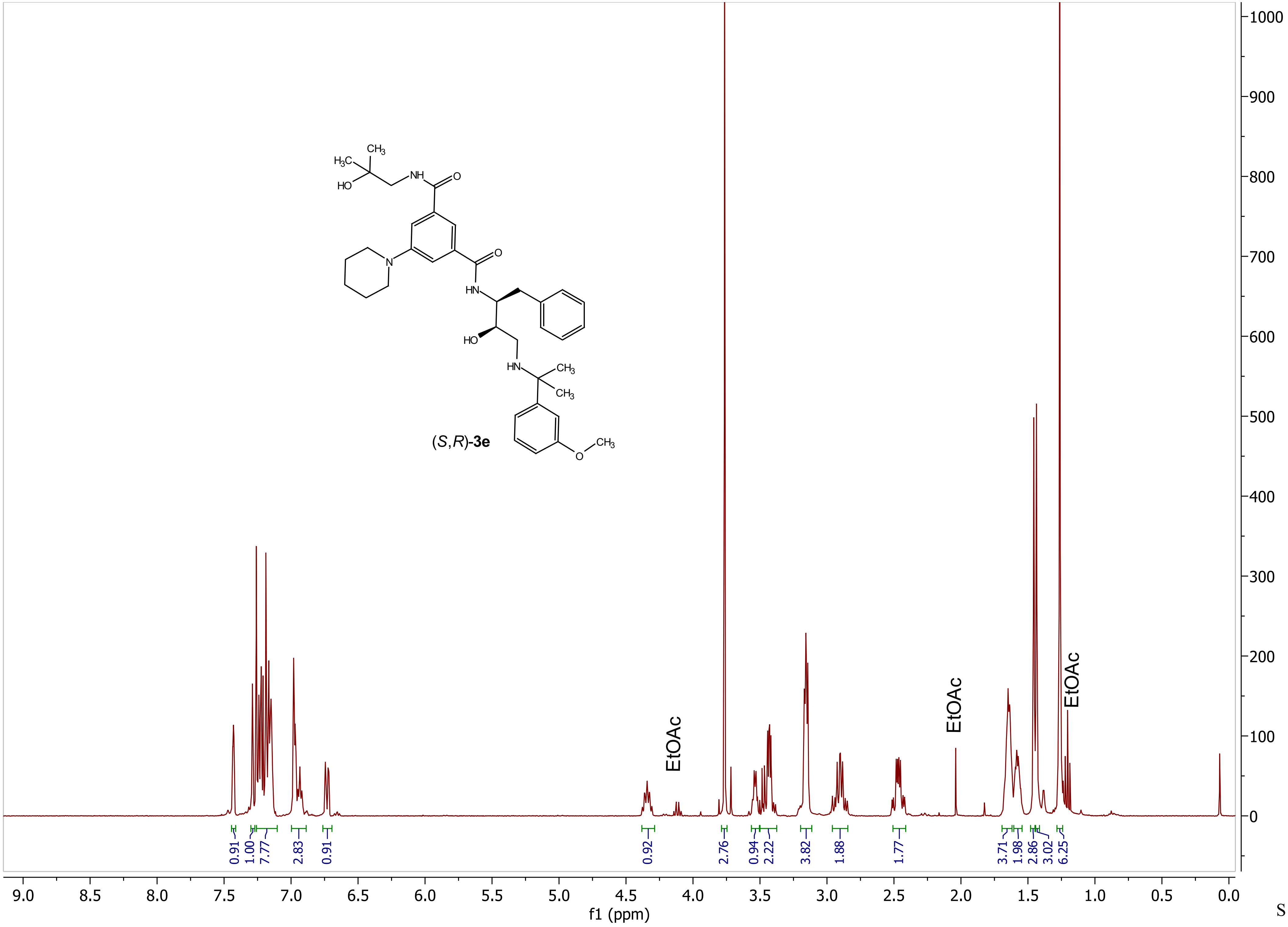

(S,R)-3e
EtOAc
EtOAc
EtOAc
0.91
1.00
7.77
2.83
0.91
0.92
2.76
0.94
2.22
3.82
1.88
1.77
3.71
1.98
2.86
3.02
6.25
9.0 8.5 8.0 7.5 7.0 6.5 6.0 5.5 5.0 4.5 4.0 3.5 3.0 2.5 2.0 1.5 1.0 0.5 0.0
f1 (ppm)
1000 900 800 700 600 500 400 300 200 100 0

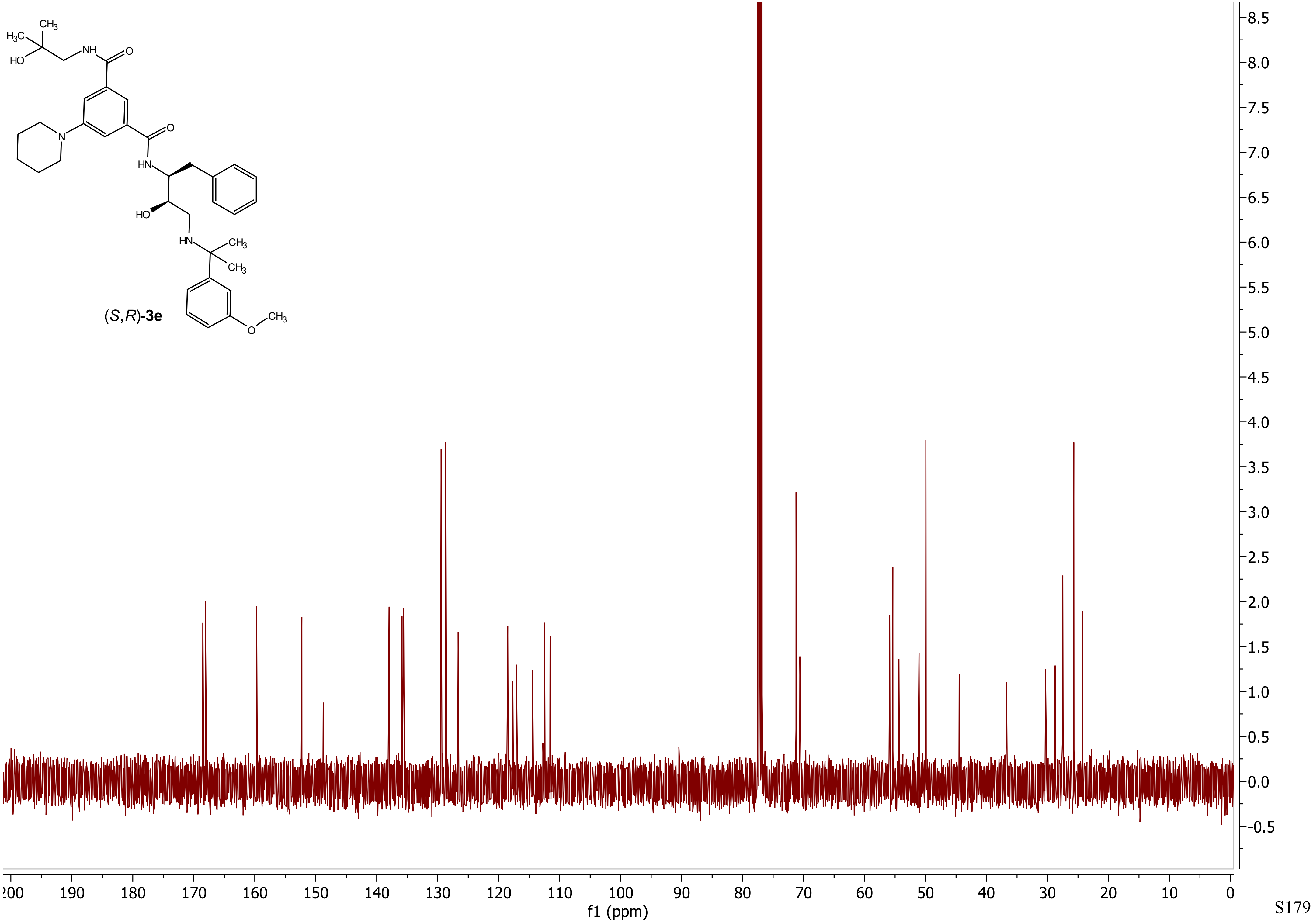

CH3
H3C
HO
NH
O
O
N
HN
HO
HN
CH3
CH3
O
CH3
(S,R)-3e
f1 (ppm)

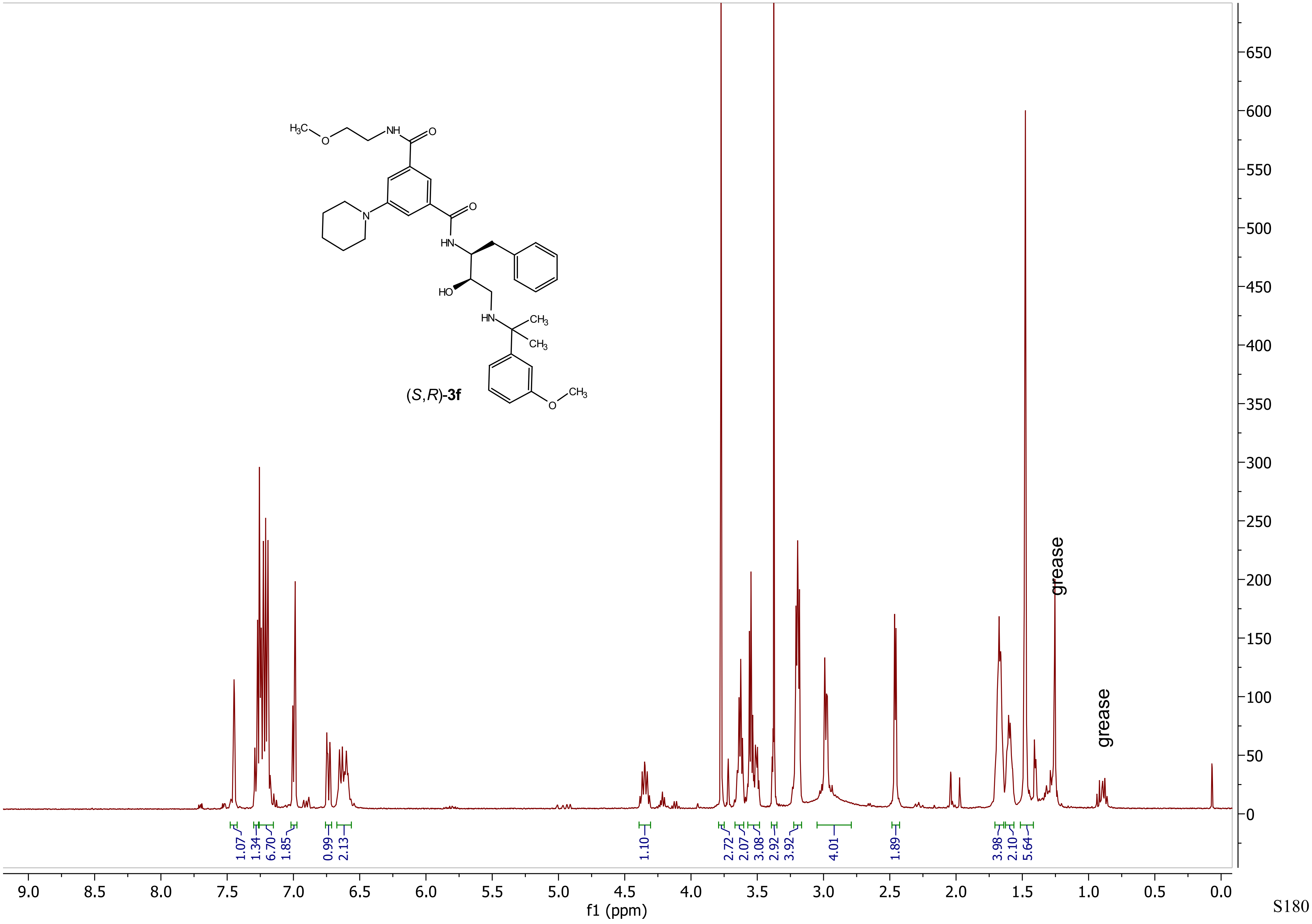
(S,R)-3f
grease
grease
1.07
1.34
6.70
1.85
0.99
2.13
1.10
2.72
2.07
3.08
2.92
3.92
4.01
1.89
3.98
2.10
5.64
f1 (ppm)

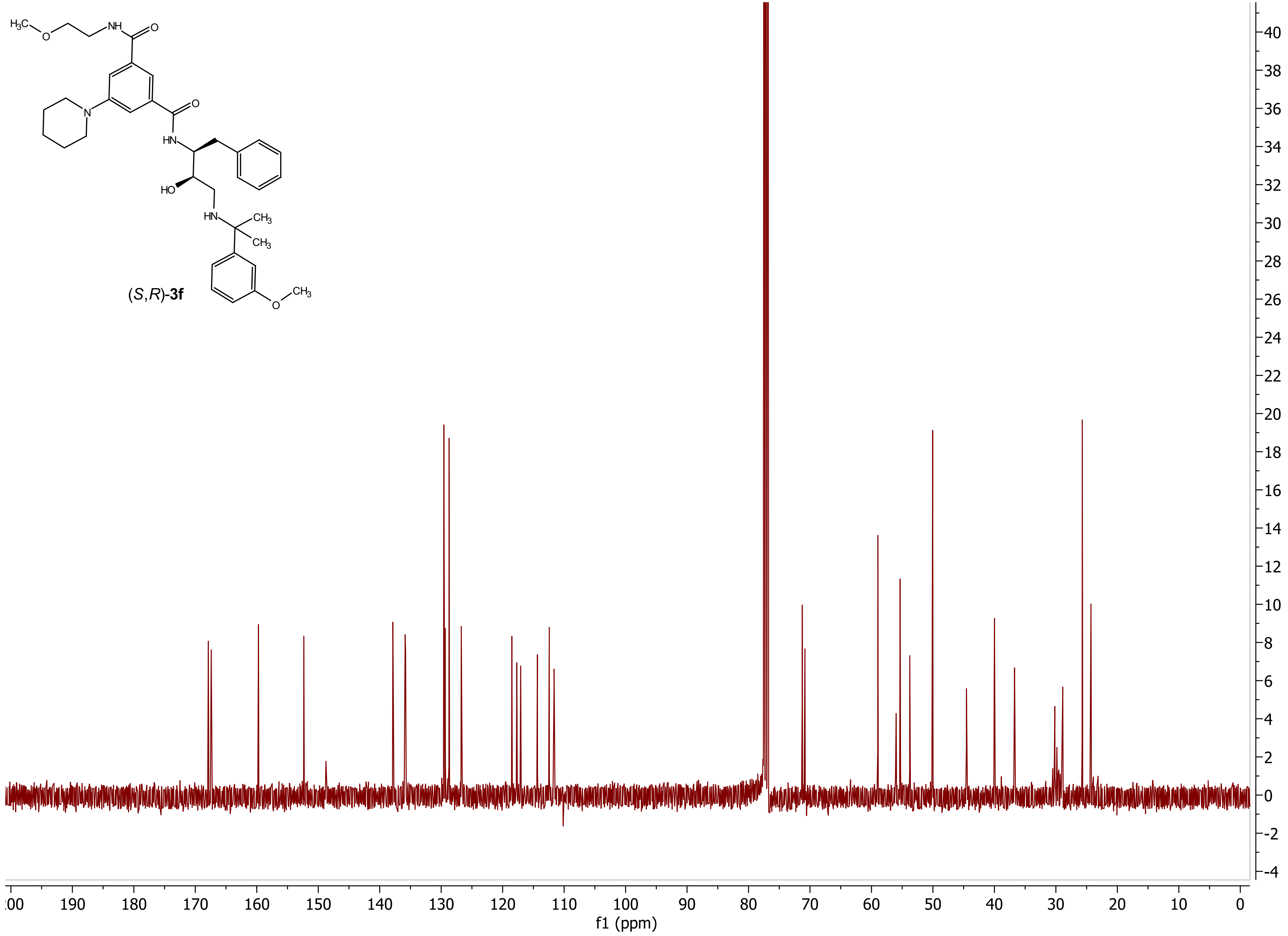

(S,R)-3f
f1 (ppm)

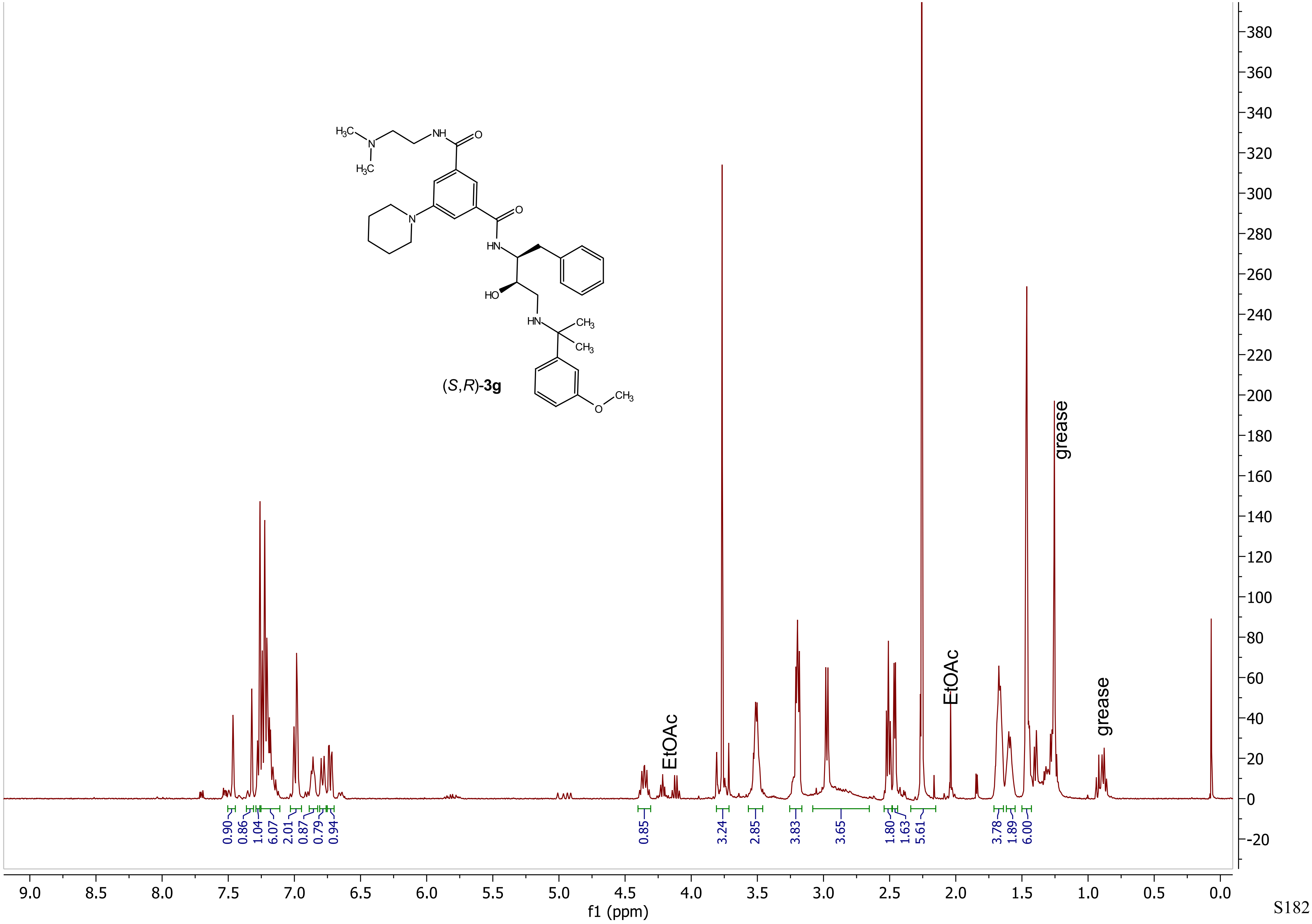
(S,R)-3g
EtOAc
EtOAc
grease
grease
0.90
0.86
1.04
6.07
2.01
0.87
0.79
0.94
0.85
3.24
2.85
3.83
3.65
1.80
1.63
5.61
3.78
1.89
6.00
f1 (ppm)

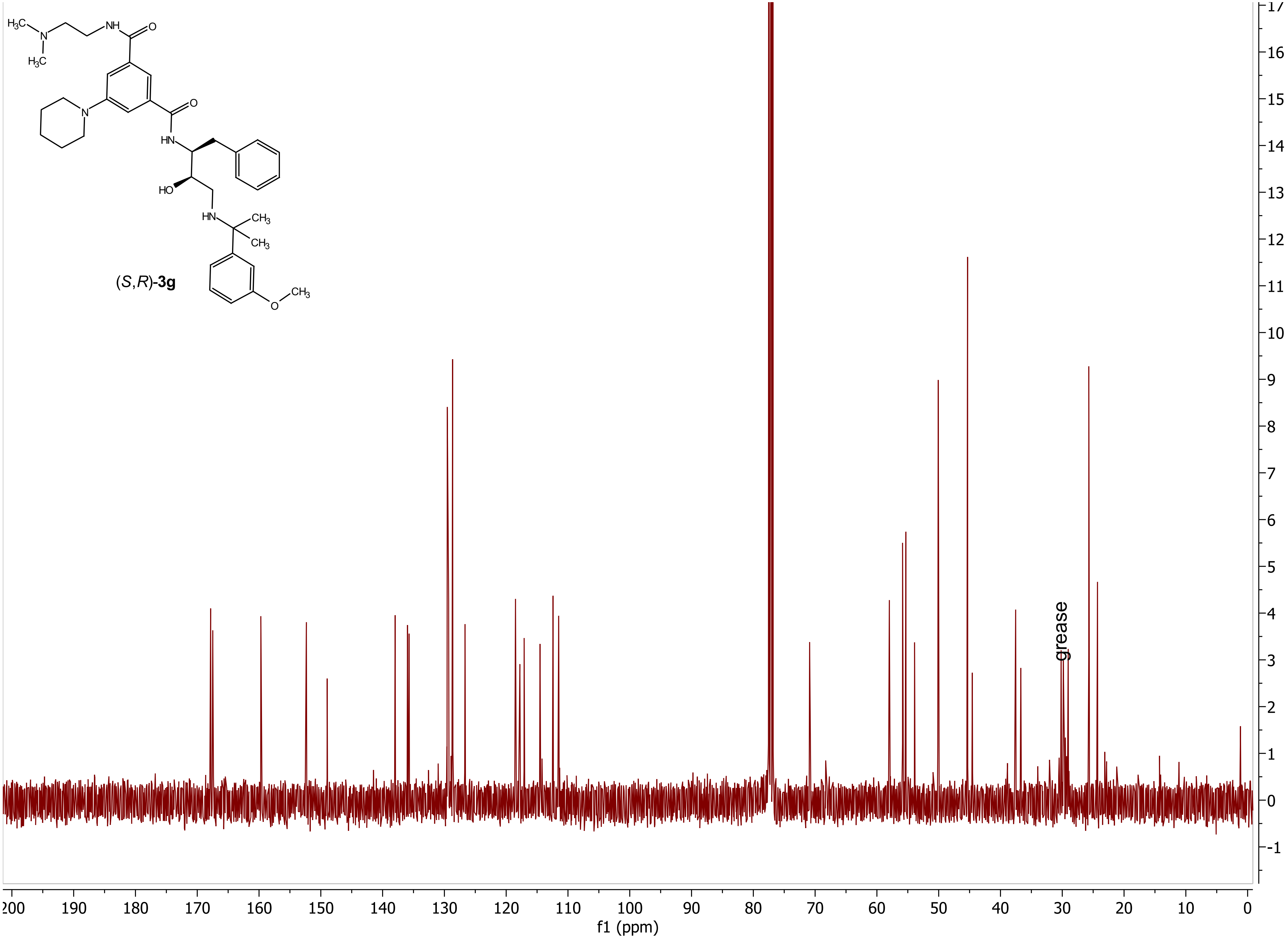

(S,R)-3g
grease
f1 (ppm)

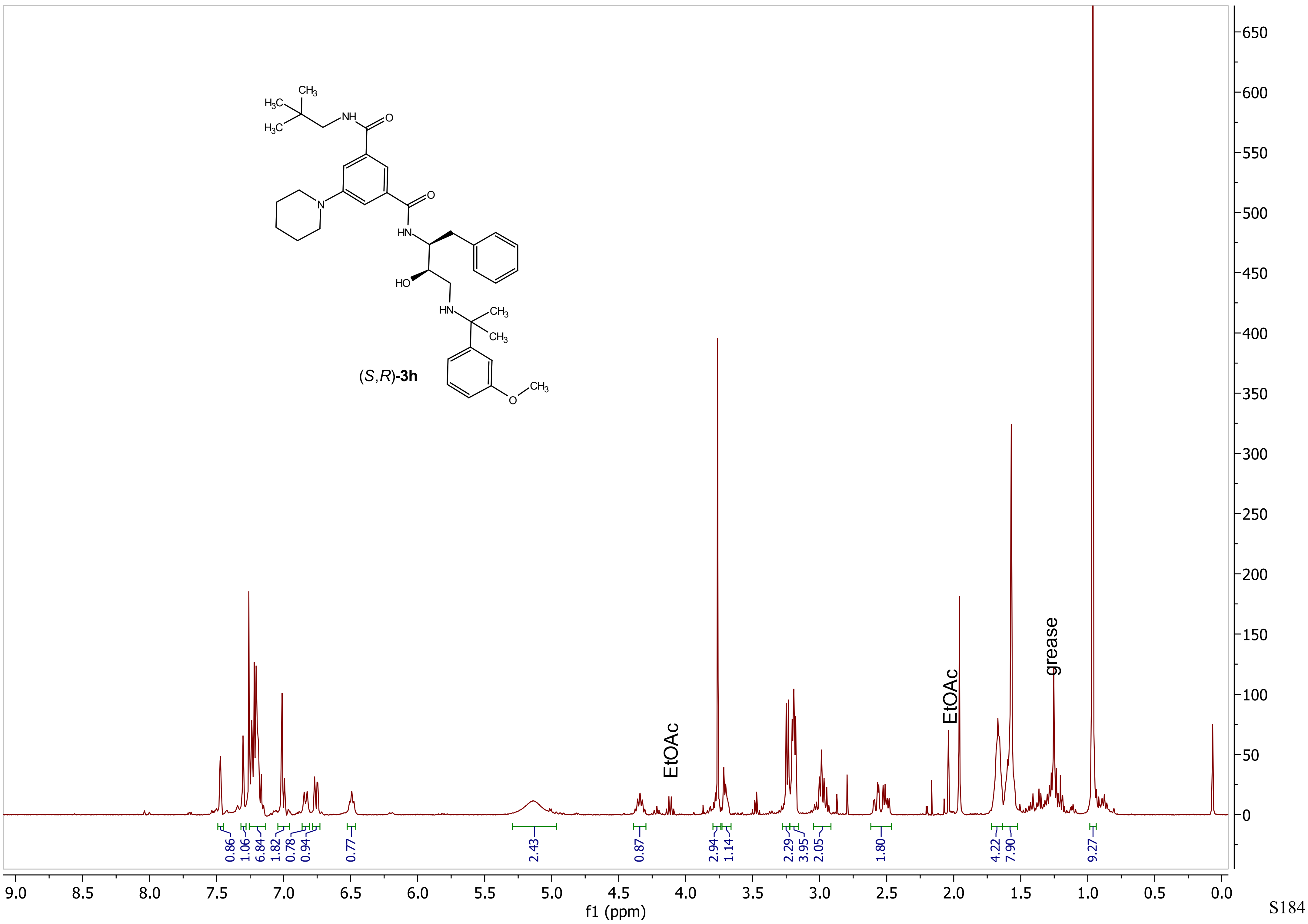

(S,R)-3h
EtOAc
EtOAc
grease
0.86
1.06
6.84
1.82
0.78
0.94
0.77
2.43
0.87
2.94
1.14
2.29
3.95
2.05
1.80
4.22
7.90
9.27
f1 (ppm)

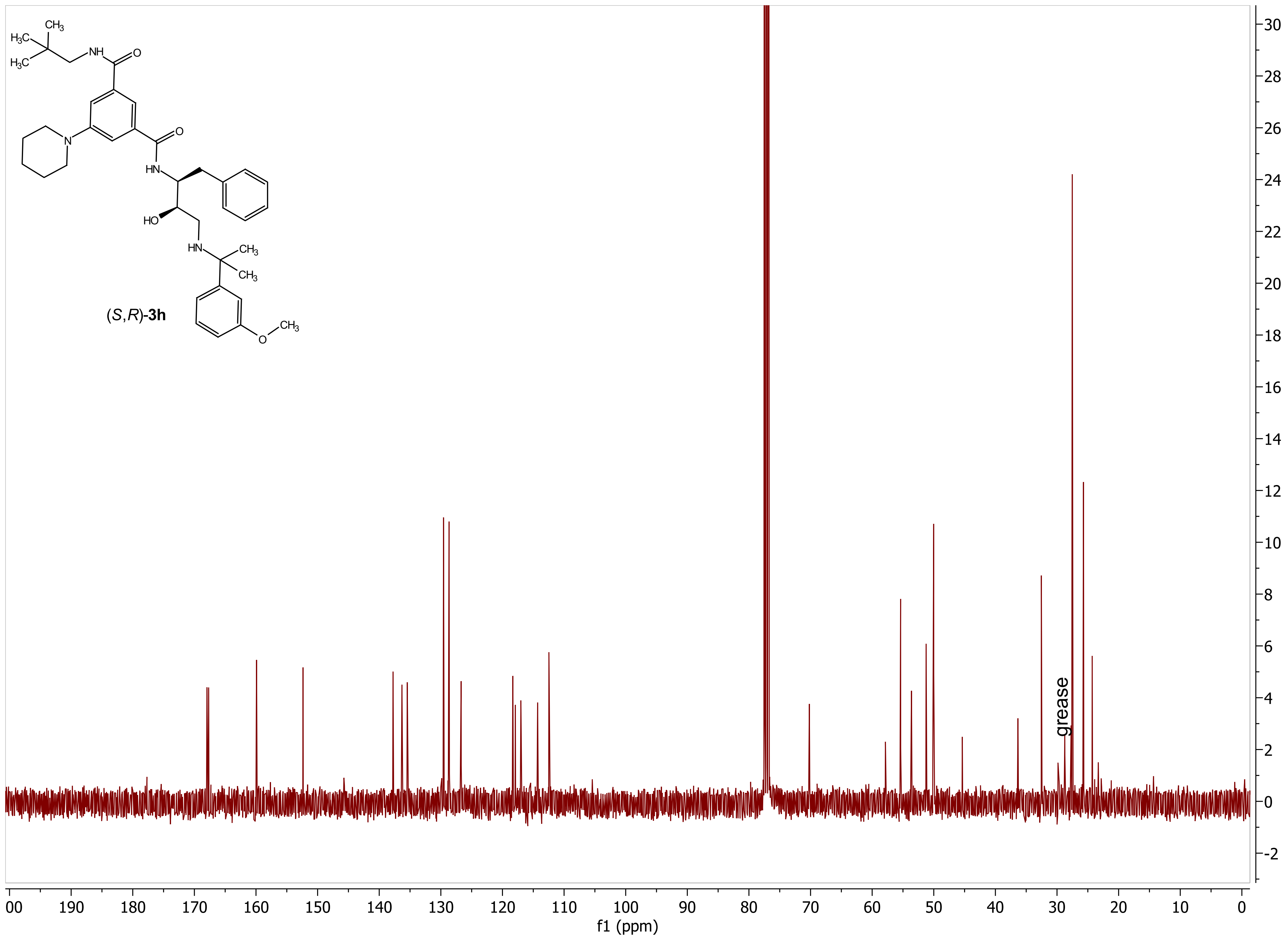

(S,R)-3h
grease
f1 (ppm)

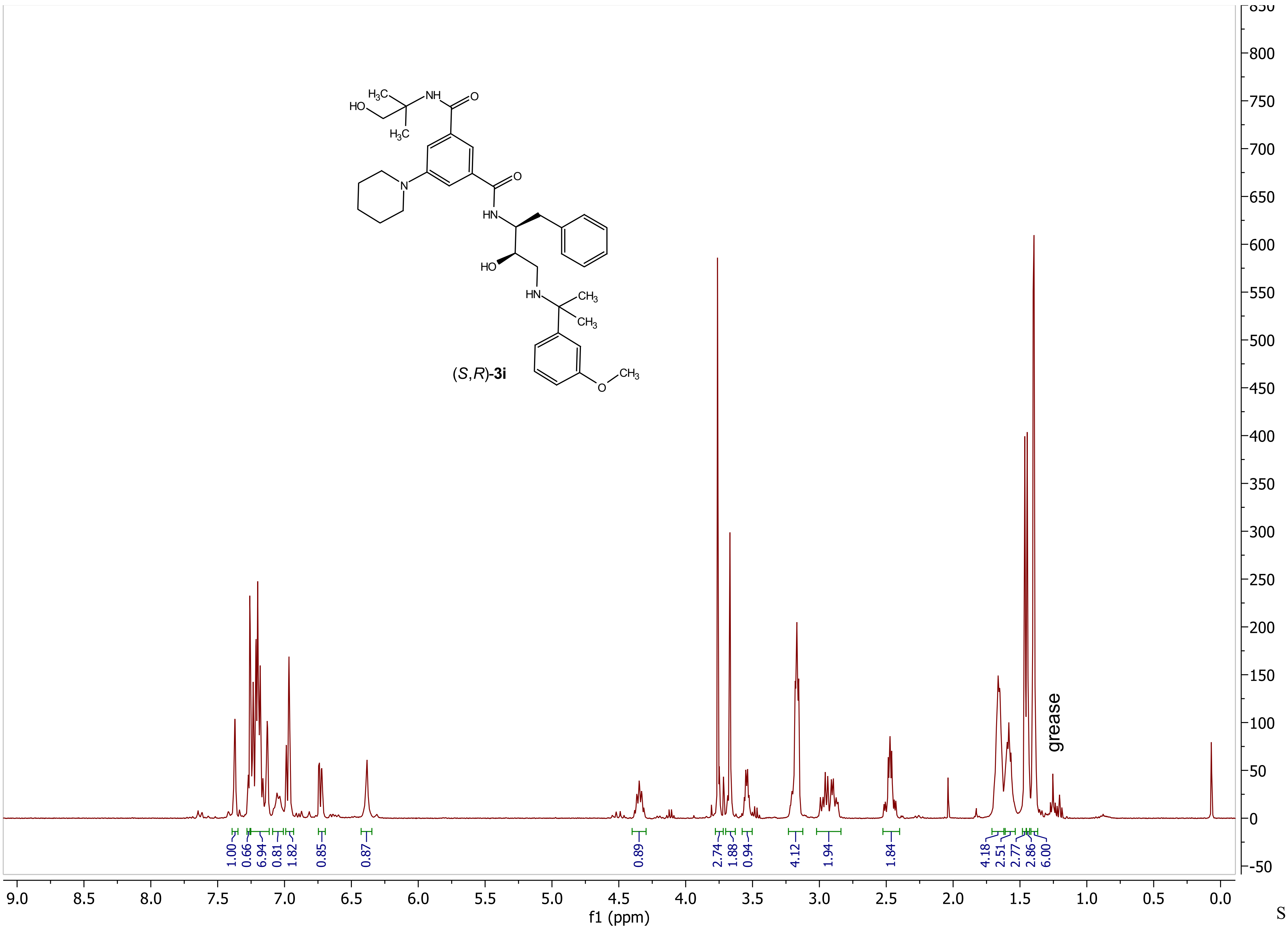
(S,R)-3i
grease
1.00
0.66
6.94
0.81
1.82
0.85
0.87
0.89
2.74
1.88
0.94
4.12
1.94
1.84
4.18
2.51
2.77
2.86
6.00
f1 (ppm)

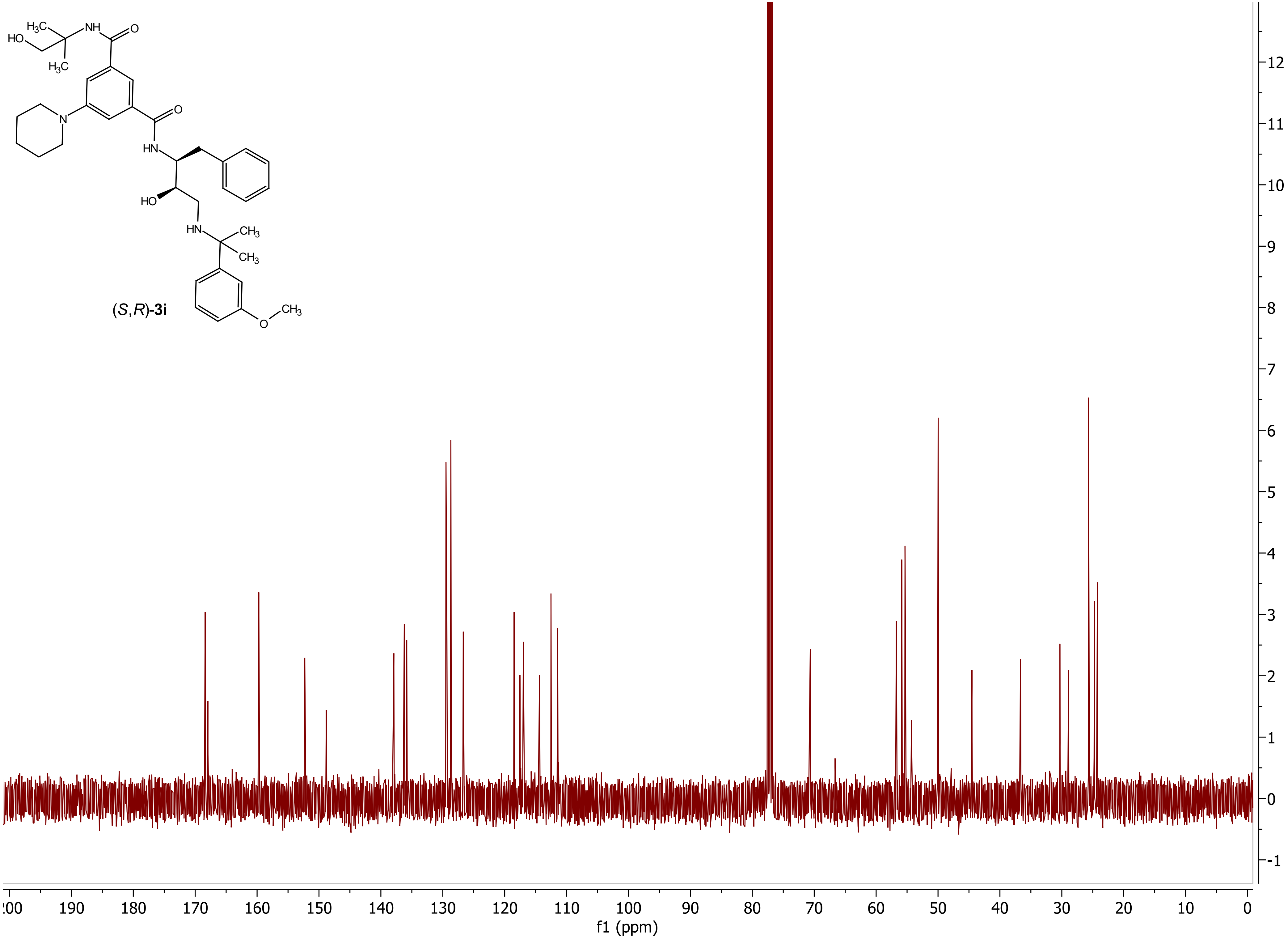
(S,R)-3i
f1 (ppm)
200 190 180 170 160 150 140 130 120 110 100 90 80 70 60 50 40 30 20 10 0
12 11 10 9 8 7 6 5 4 3 2 1 0 -1

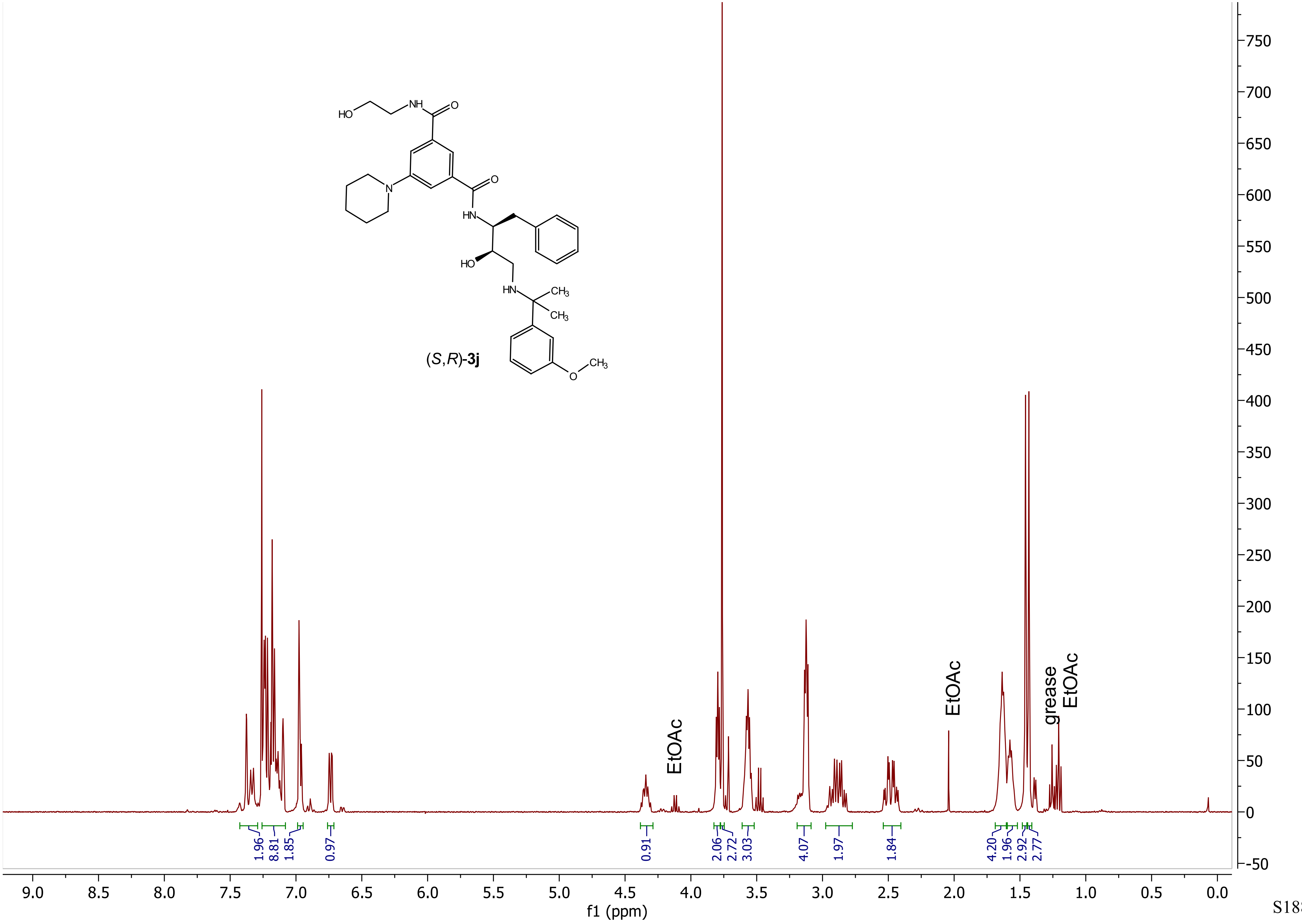
(S,R)-3j
HO
NH
O
N
HN
HO
HN
CH3
CH3
O
CH3
EtOAc
EtOAc
grease
EtOAc
1.96
8.81
1.85
0.97
0.91
2.06
2.72
3.03
4.07
1.97
1.84
4.20
1.96
2.92
2.77
f1 (ppm)

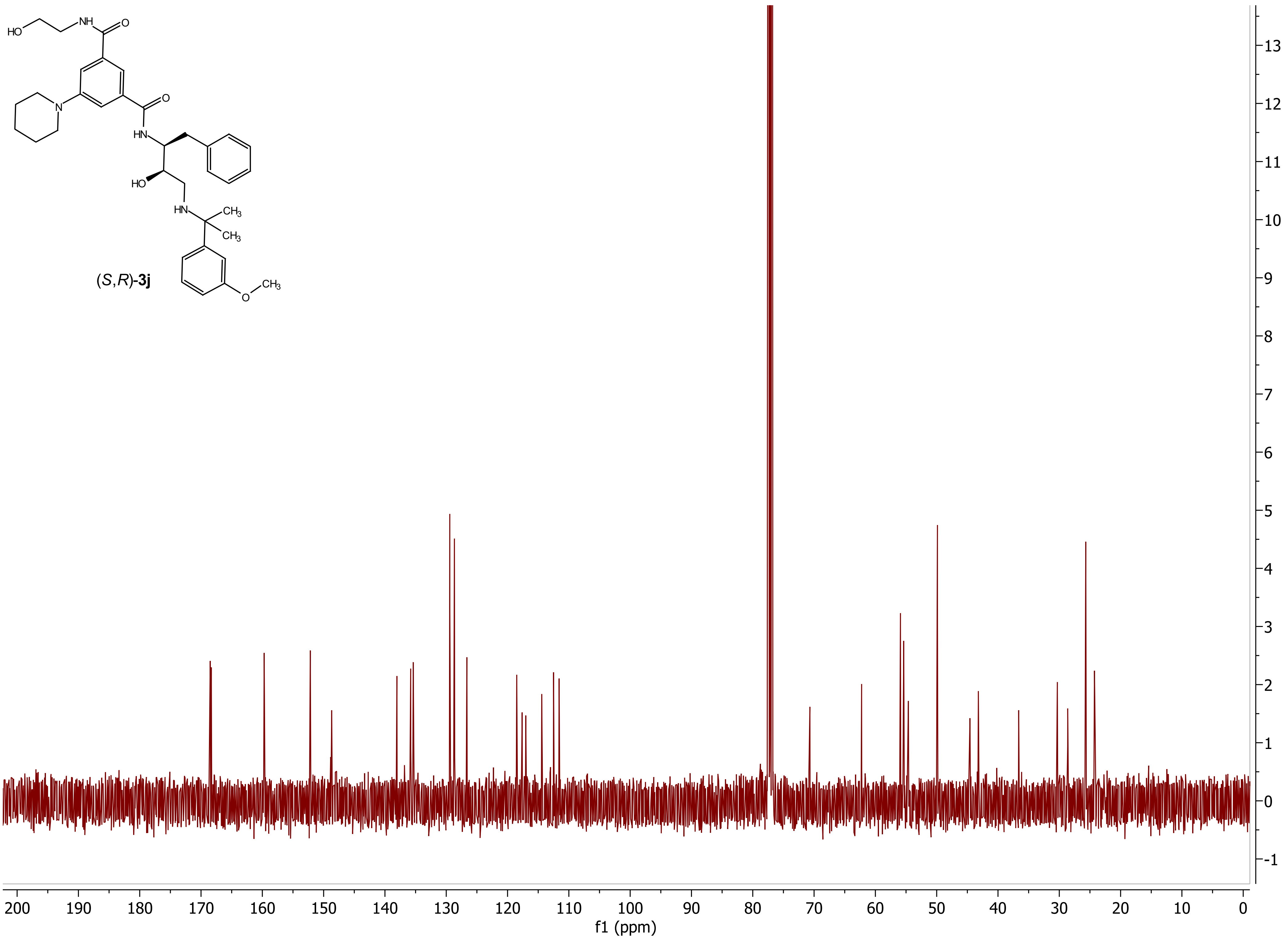
HO
NH
O
N
O
HN
HO
HN
CH3
CH3
O
CH3
(S,R)-3j
f1 (ppm)

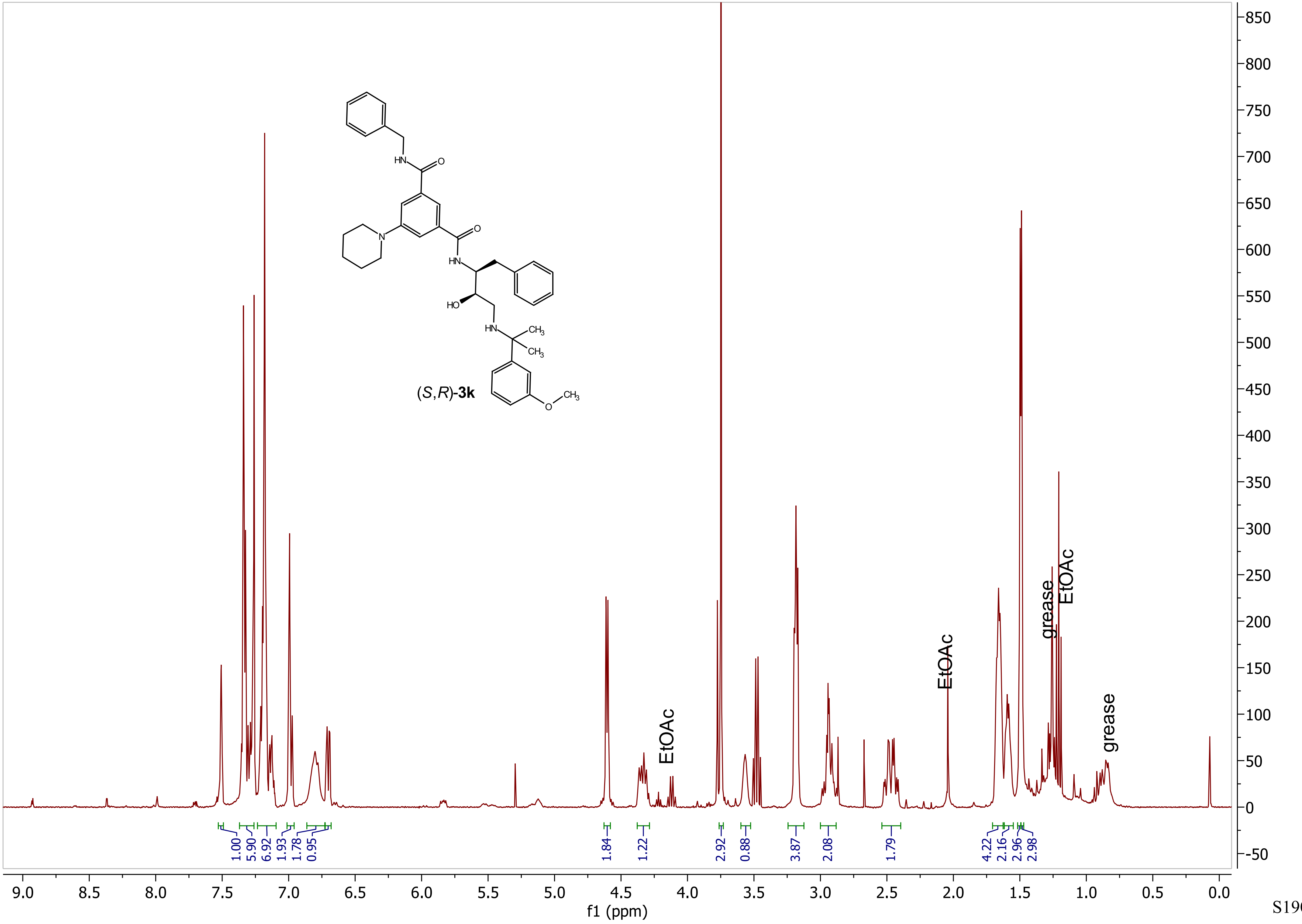
(S,R)-3k
EtOAc
EtOAc
grease
EtOAc
grease
1.00
5.90
6.92
1.93
1.78
0.95
1.84
1.22
2.92
0.88
3.87
2.08
1.79
4.22
2.16
2.96
2.98
f1 (ppm)

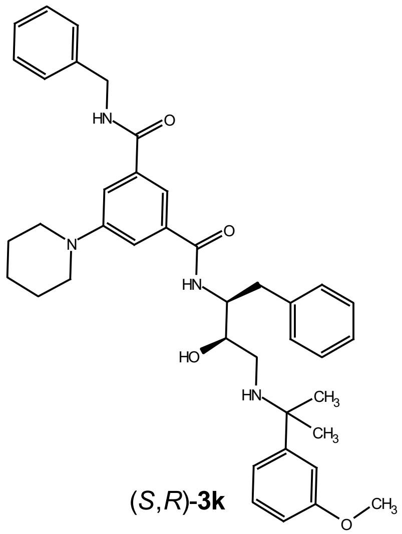

(*S*,*R*)-**3k**

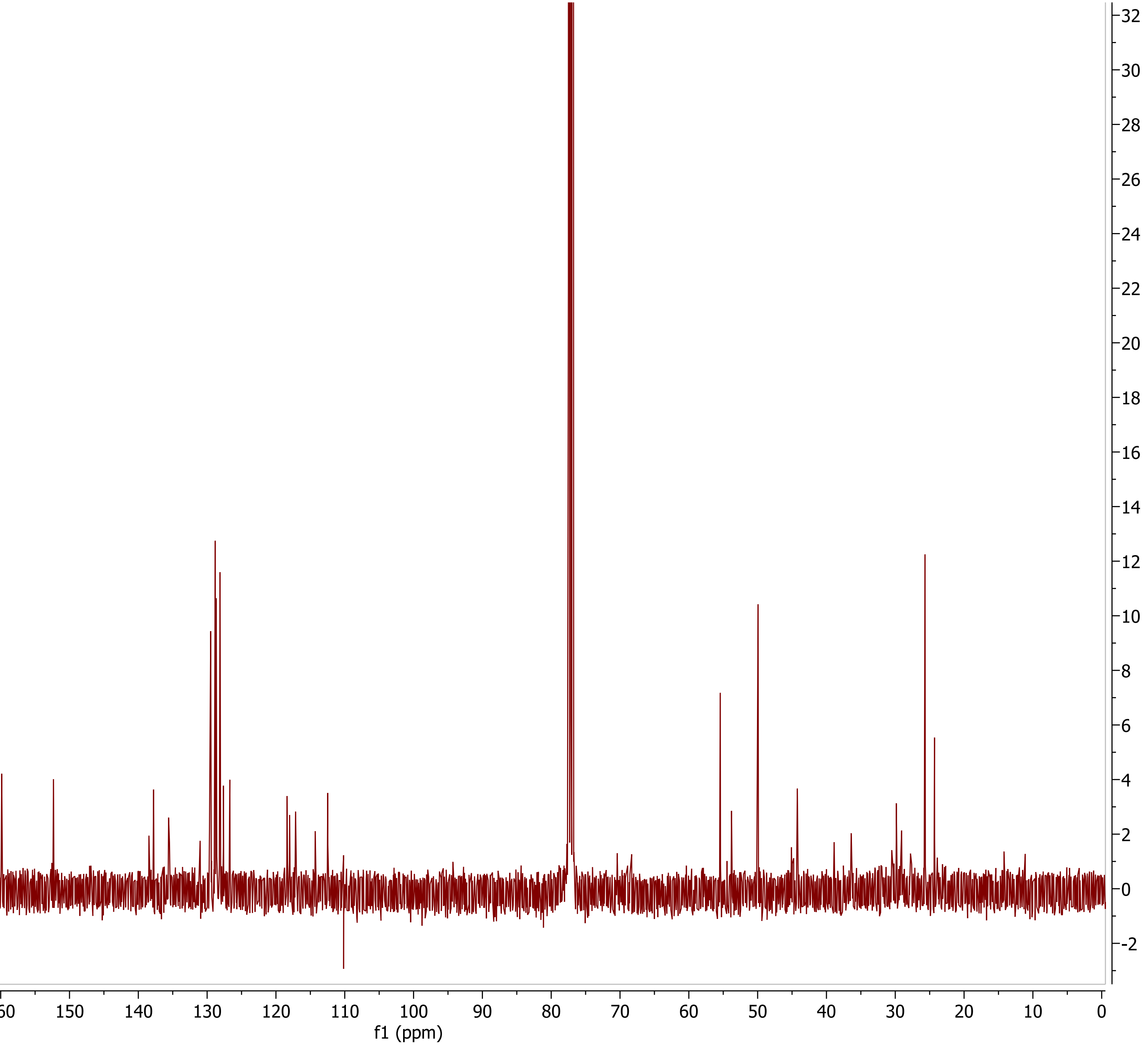

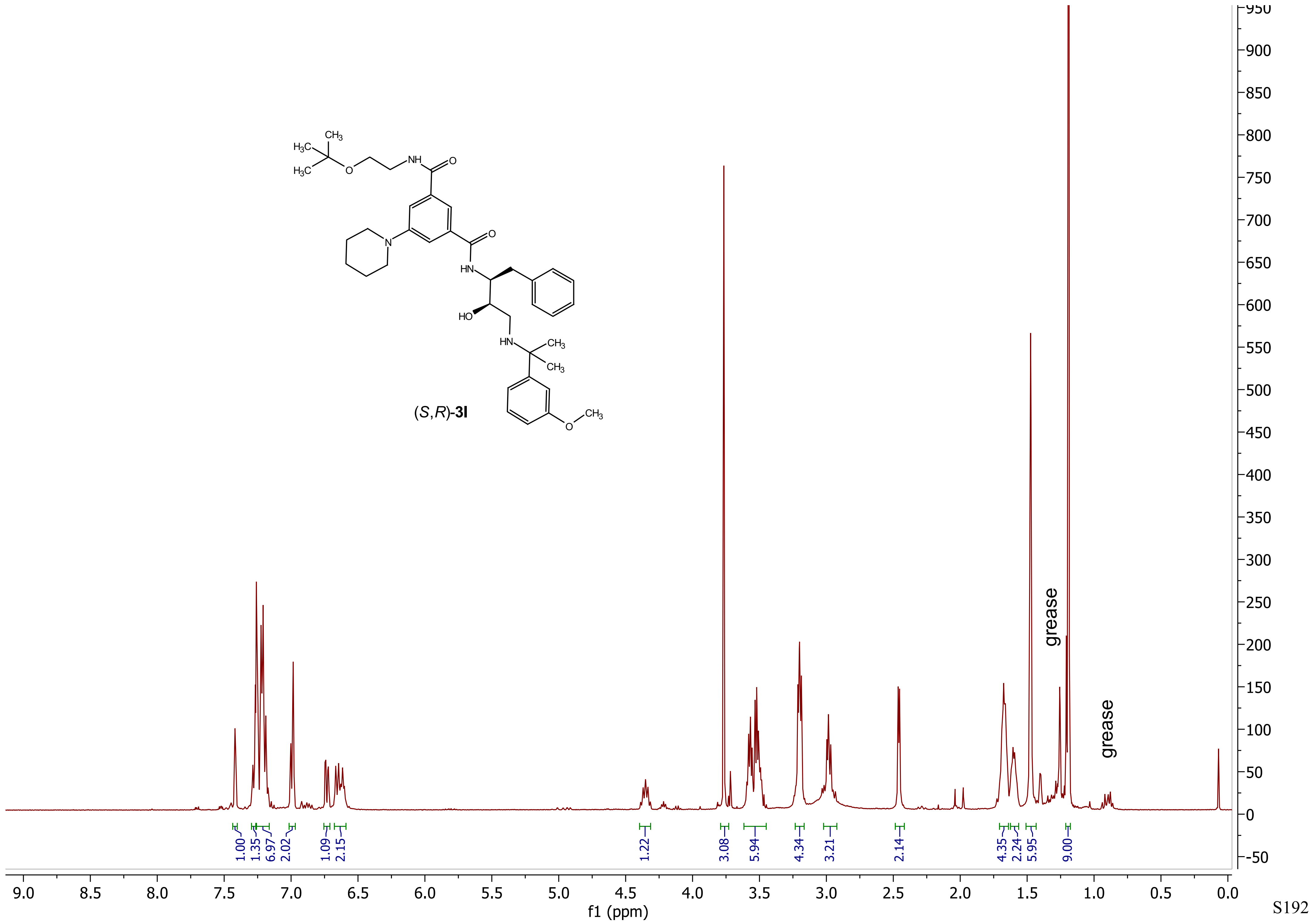

(S,R)-3l
grease
grease
1.00
1.35
6.97
2.02
1.09
2.15
1.22
3.08
5.94
4.34
3.21
2.14
4.35
2.24
5.95
9.00
f1 (ppm)

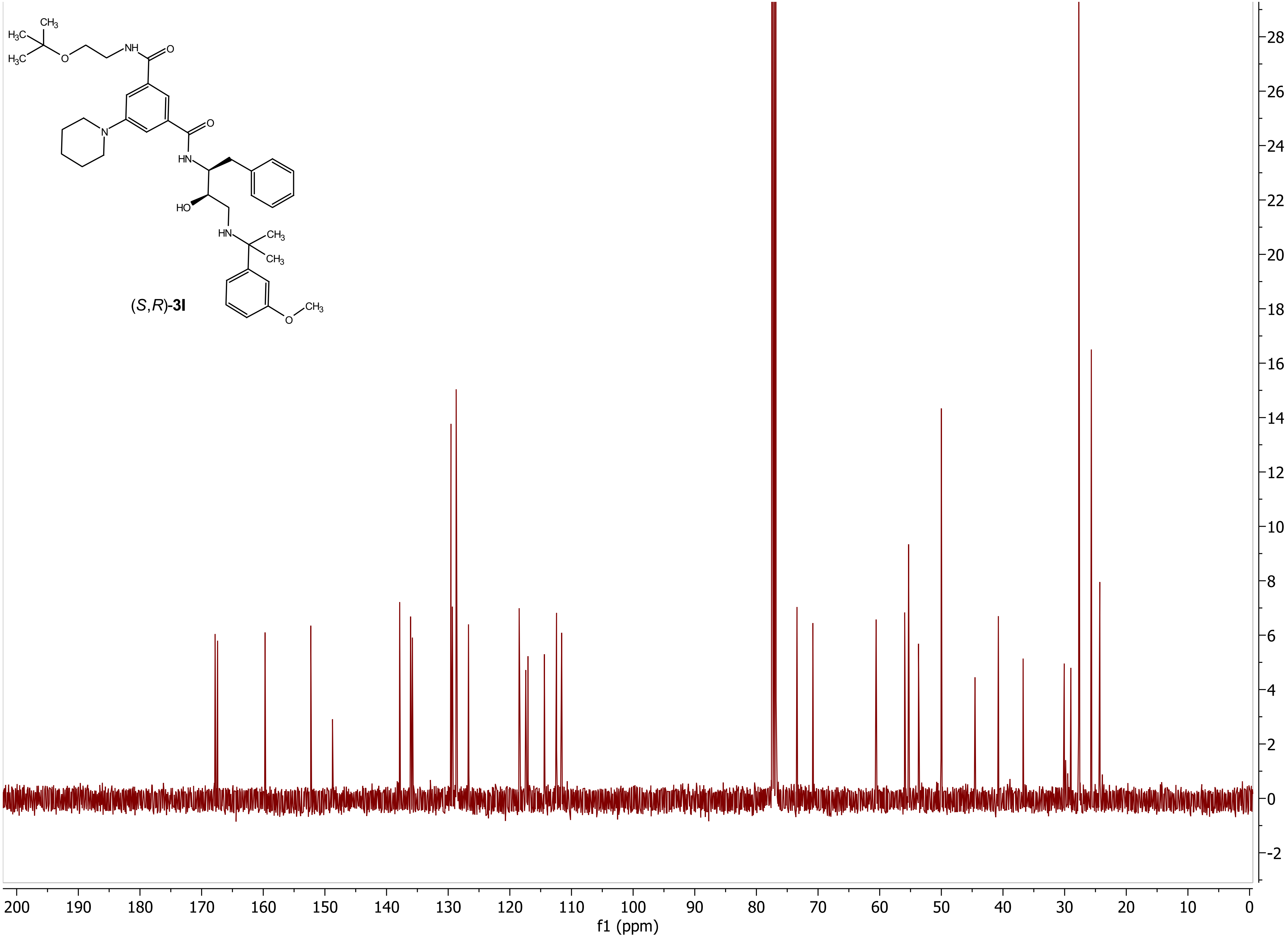

(S,R)-3l
f1 (ppm)
200 190 180 170 160 150 140 130 120 110 100 90 80 70 60 50 40 30 20 10 0
28 26 24 22 20 18 16 14 12 10 8 6 4 2 0 -2

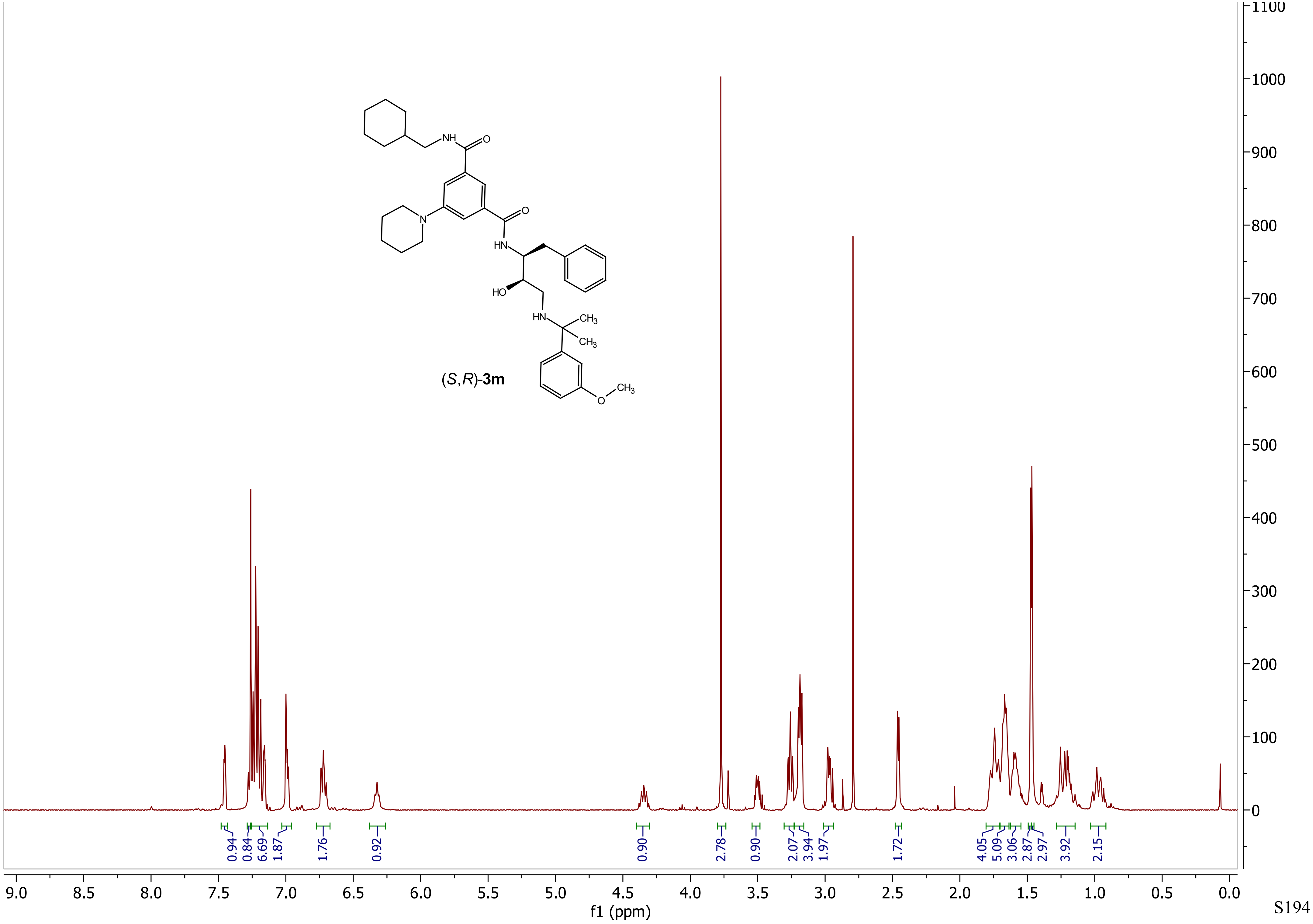

NH
O
O
N
HN
HO
HN
CH3
CH3
O
CH3
(S,R)-3m
0.94
0.84
6.69
1.87
1.76
0.92
0.90
2.78
0.90
2.07
3.94
1.97
1.72
4.05
5.09
3.06
2.87
2.97
3.92
2.15
f1 (ppm)

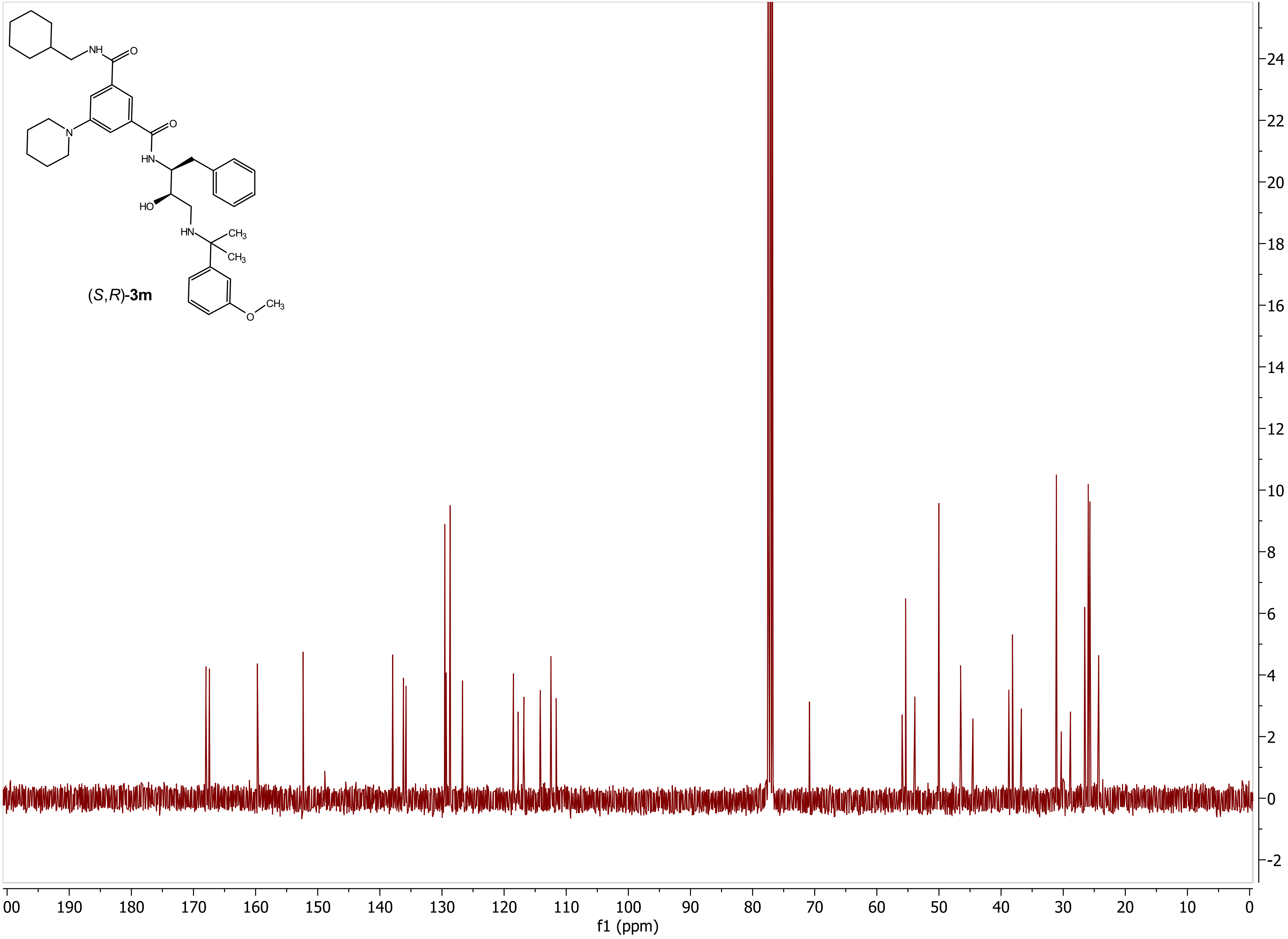

(S,R)-3m
NH
O
HN
HO
CH3
f1 (ppm)

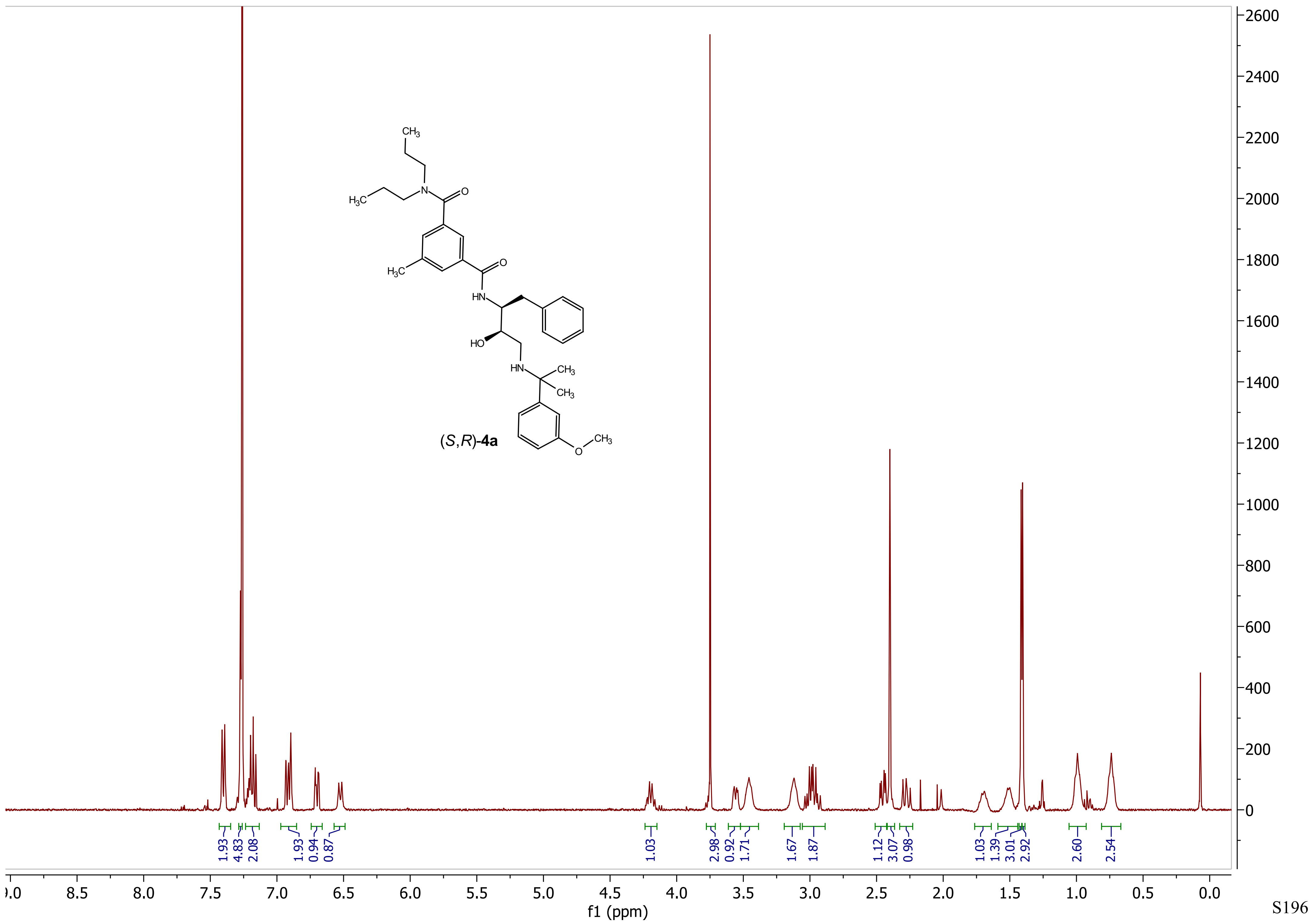
(S,R)-4a
f1 (ppm)

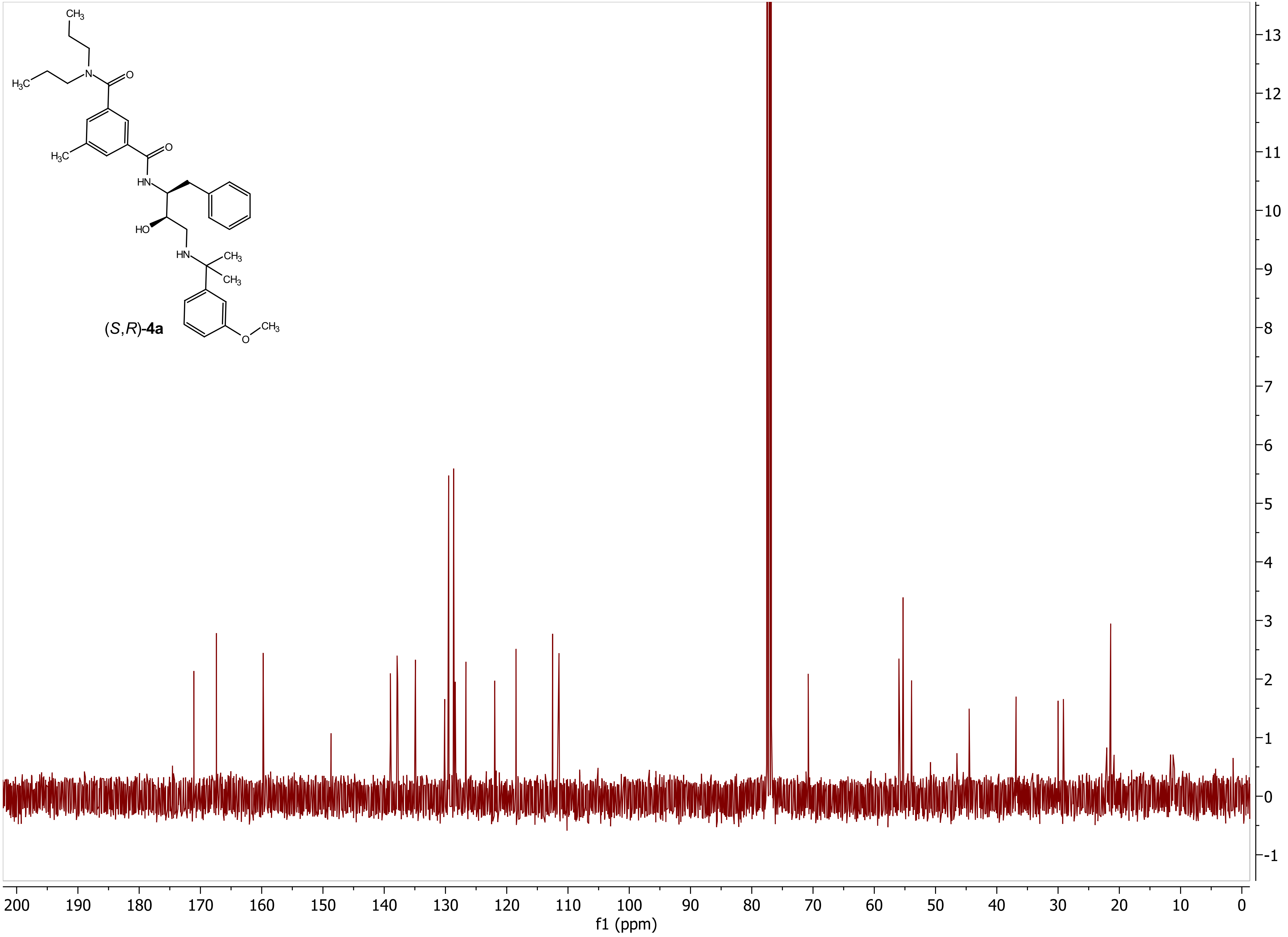

CH3
H3C
N
O
H3C
O
HN
HO
HN
CH3
CH3
(S,R)-4a
O
CH3
200 190 180 170 160 150 140 130 120 110 100 90 80 70 60 50 40 30 20 10 0
f1 (ppm)
13 12 11 10 9 8 7 6 5 4 3 2 1 0 -1

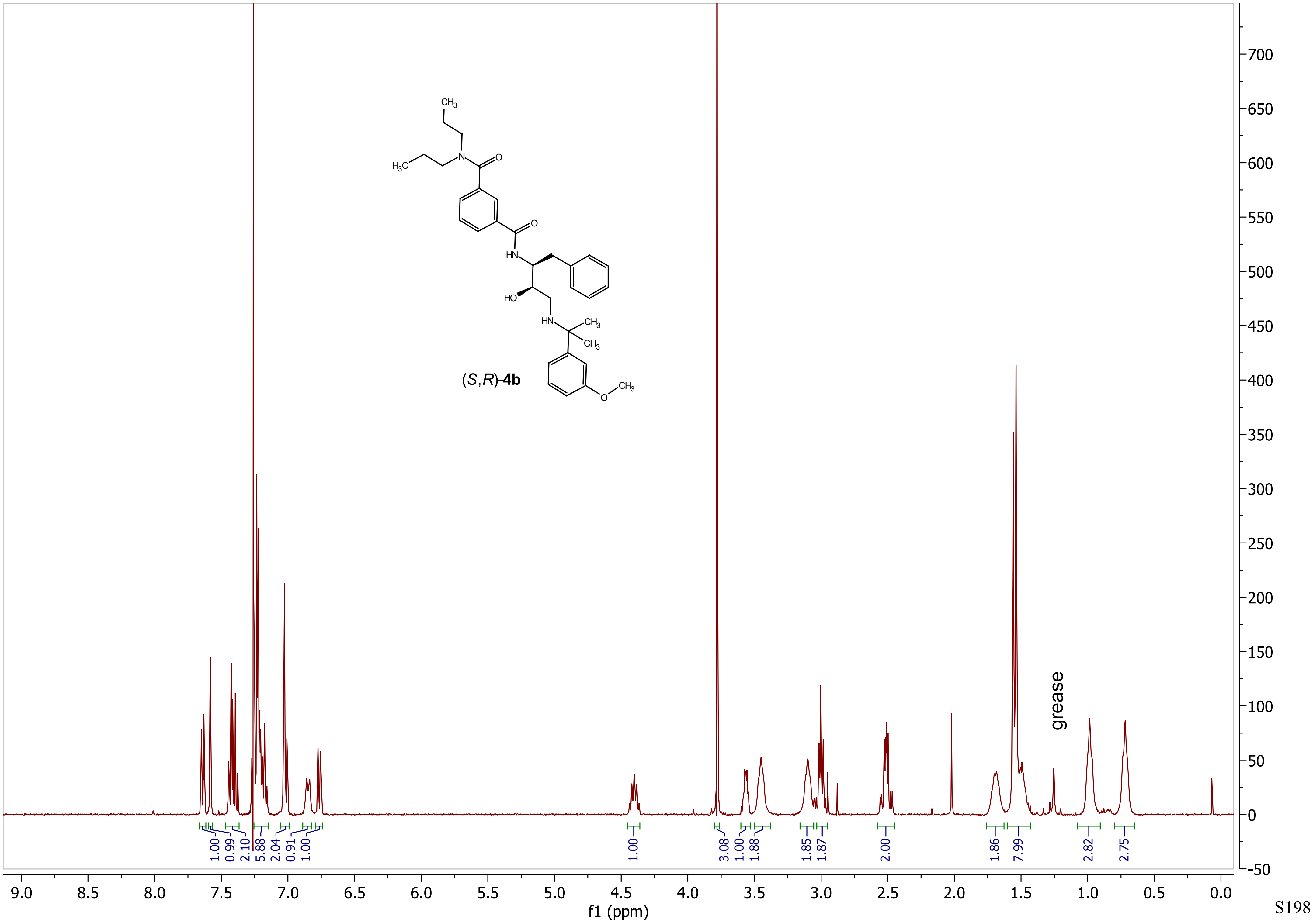

(S,R)-4b
grease
1.00
0.99
2.10
5.88
2.04
0.91
1.00
1.00
3.08
1.00
1.88
1.85
1.87
2.00
1.86
7.99
2.82
2.75
f1 (ppm)

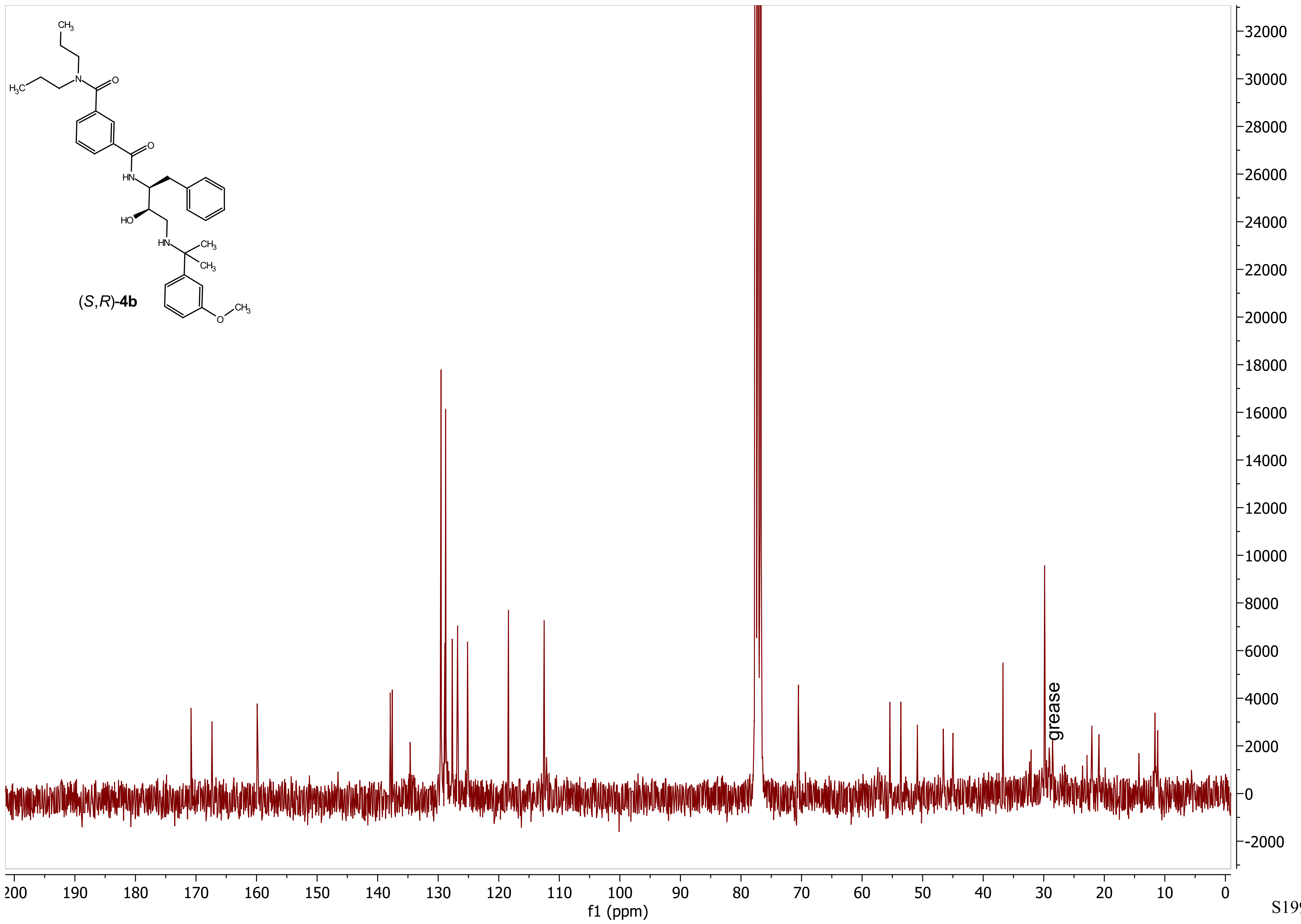
(S,R)-4b
grease
f1 (ppm)

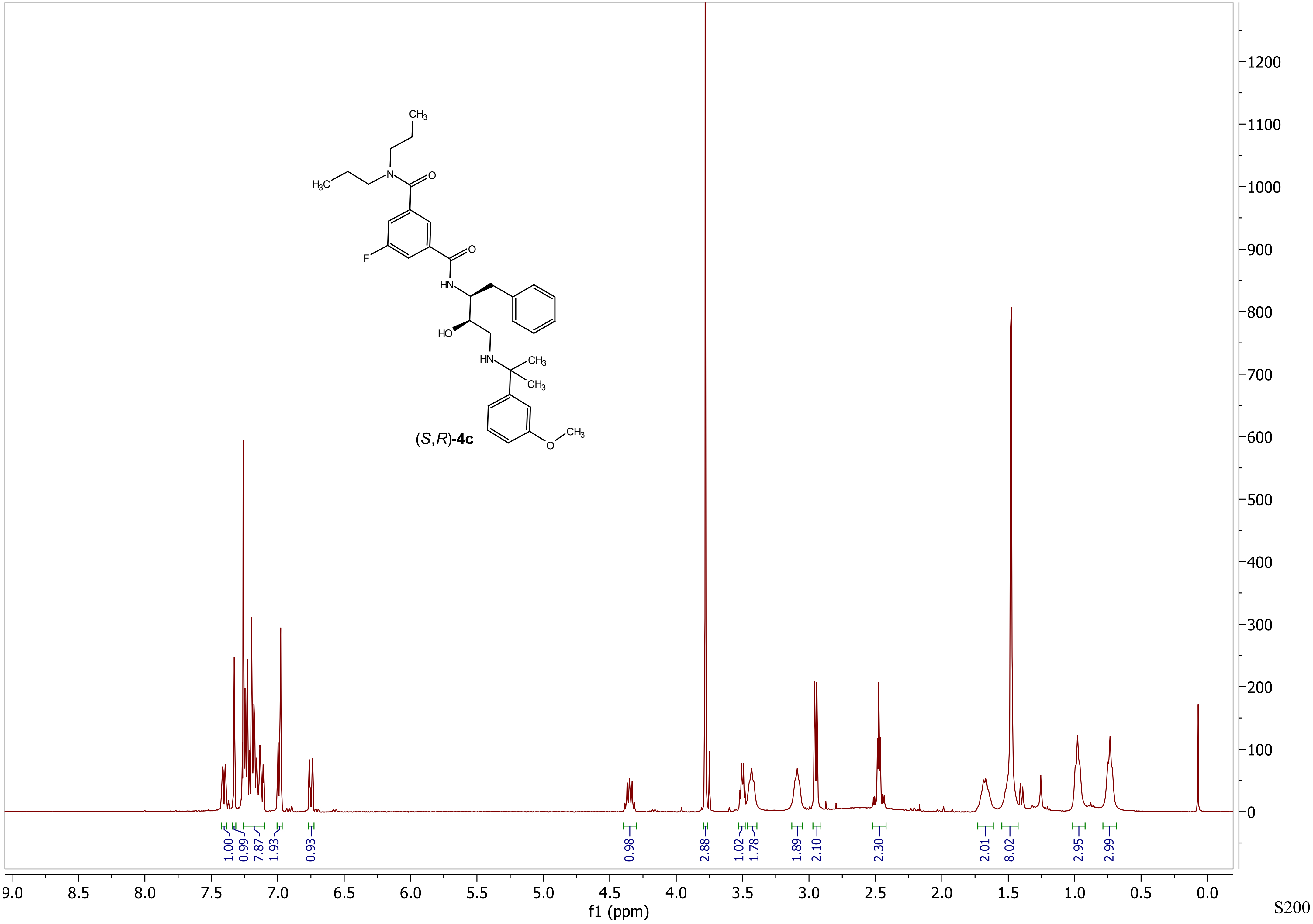
CH3
H3C
N
O
F
O
HN
HO
HN
CH3
CH3
O
CH3
(S,R)-4c
1.00
0.99
7.87
1.93
0.93
0.98
2.88
1.02
1.78
1.89
2.10
2.30
2.01
8.02
2.95
2.99
9.0
8.5
8.0
7.5
7.0
6.5
6.0
5.5
5.0
4.5
4.0
3.5
3.0
2.5
2.0
1.5
1.0
0.5
0.0
f1 (ppm)
1200
1100
1000
900
800
700
600
500
400
300
200
100
0

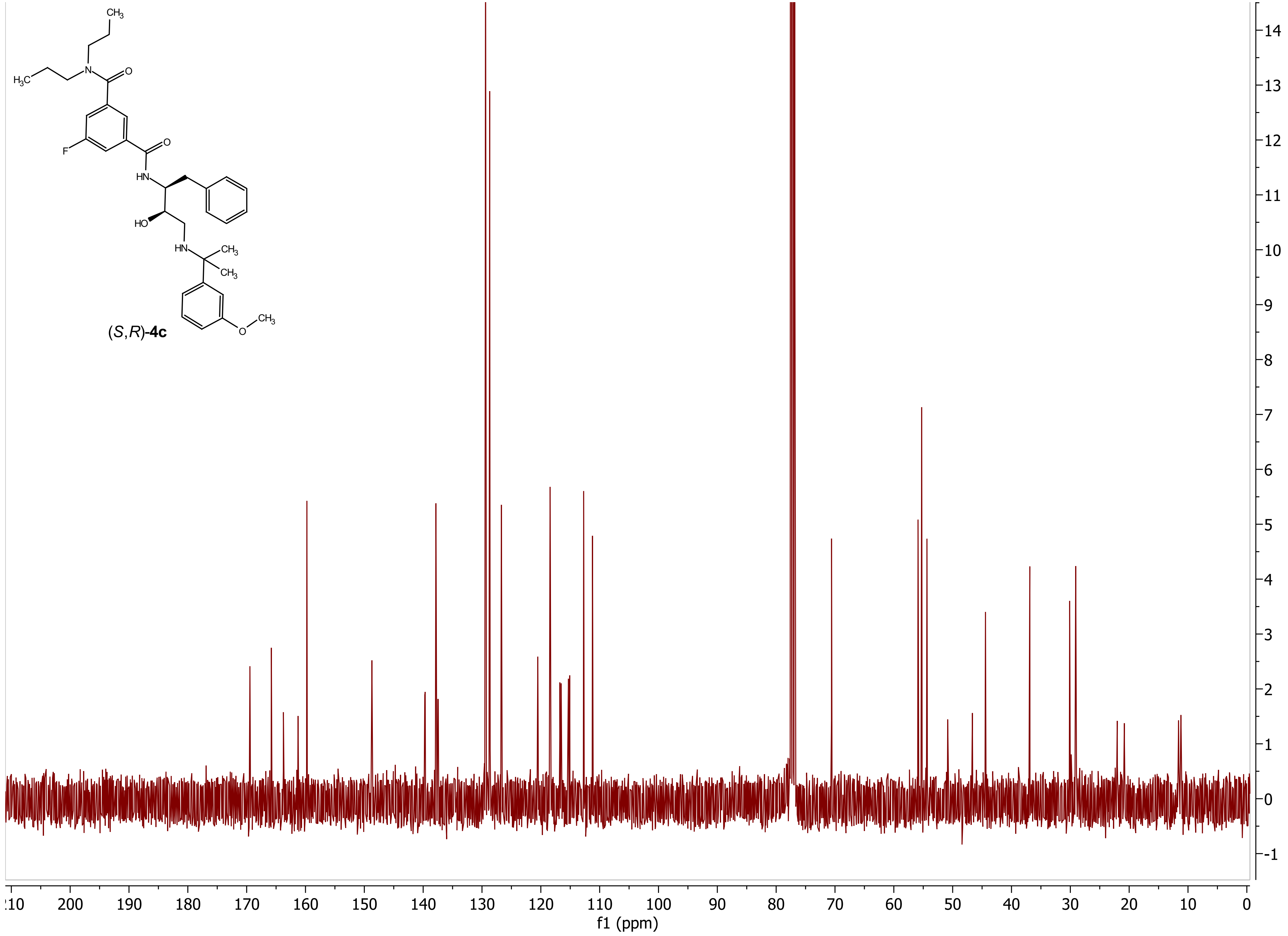
(S,R)-4c
f1 (ppm)

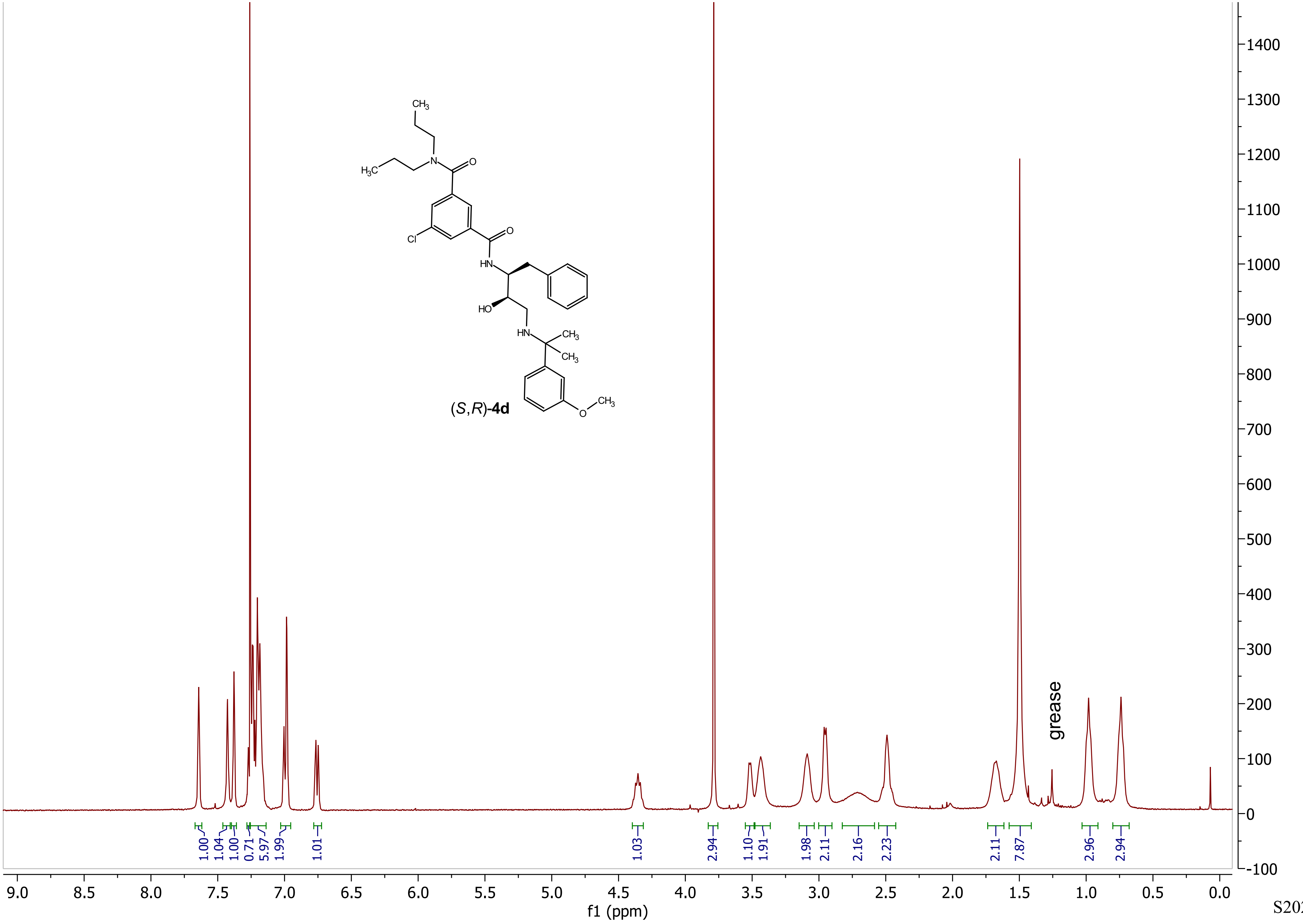

(S,R)-4d
grease
1.00
1.04
1.00
0.71
5.97
1.99
1.01
1.03
2.94
1.10
1.91
1.98
2.11
2.16
2.23
2.11
7.87
2.96
2.94
f1 (ppm)

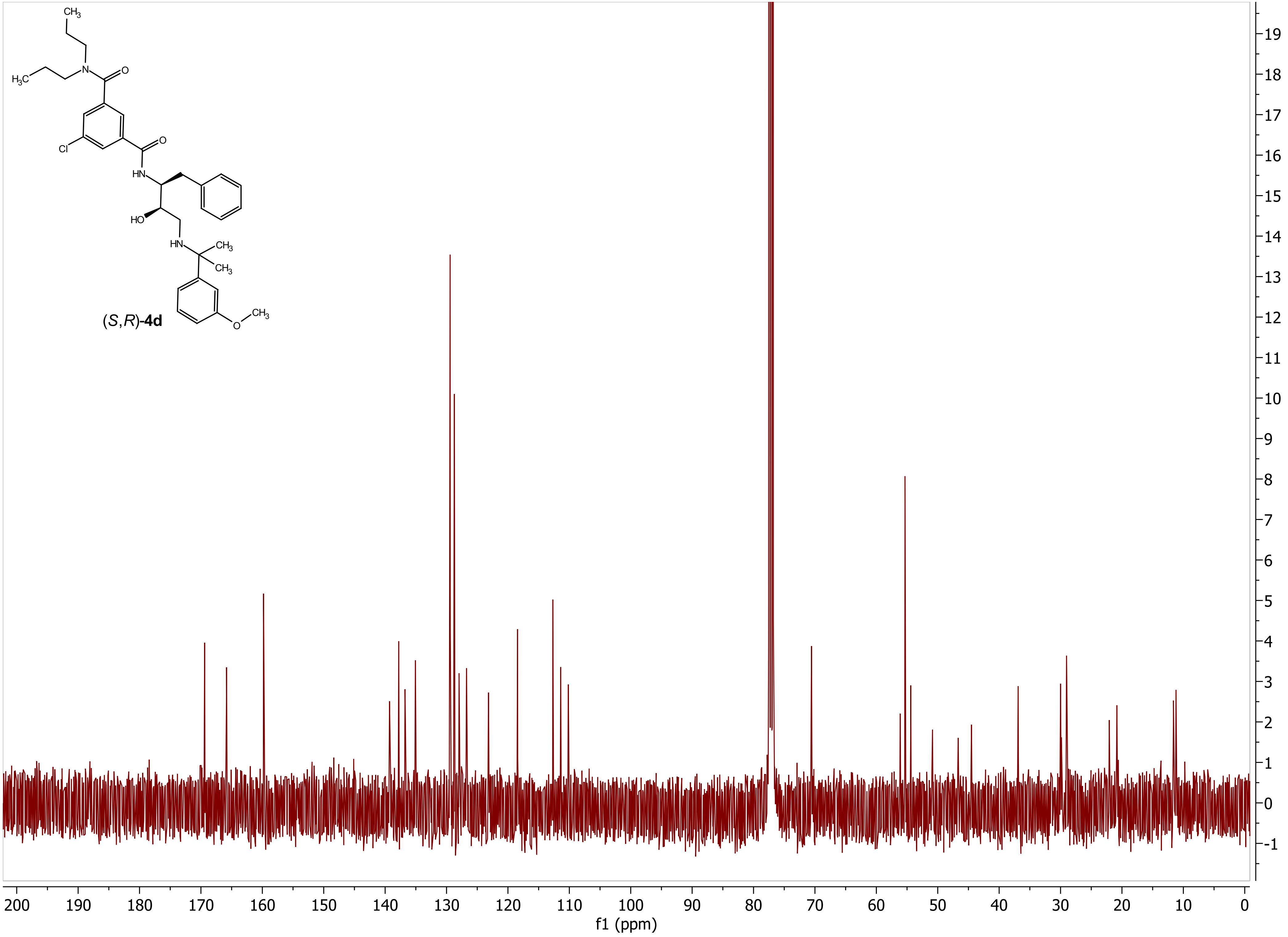

CH3
H3C
N
O
Cl
O
HN
HO
HN
CH3
CH3
O
CH3
(S,R)-4d
f1 (ppm)

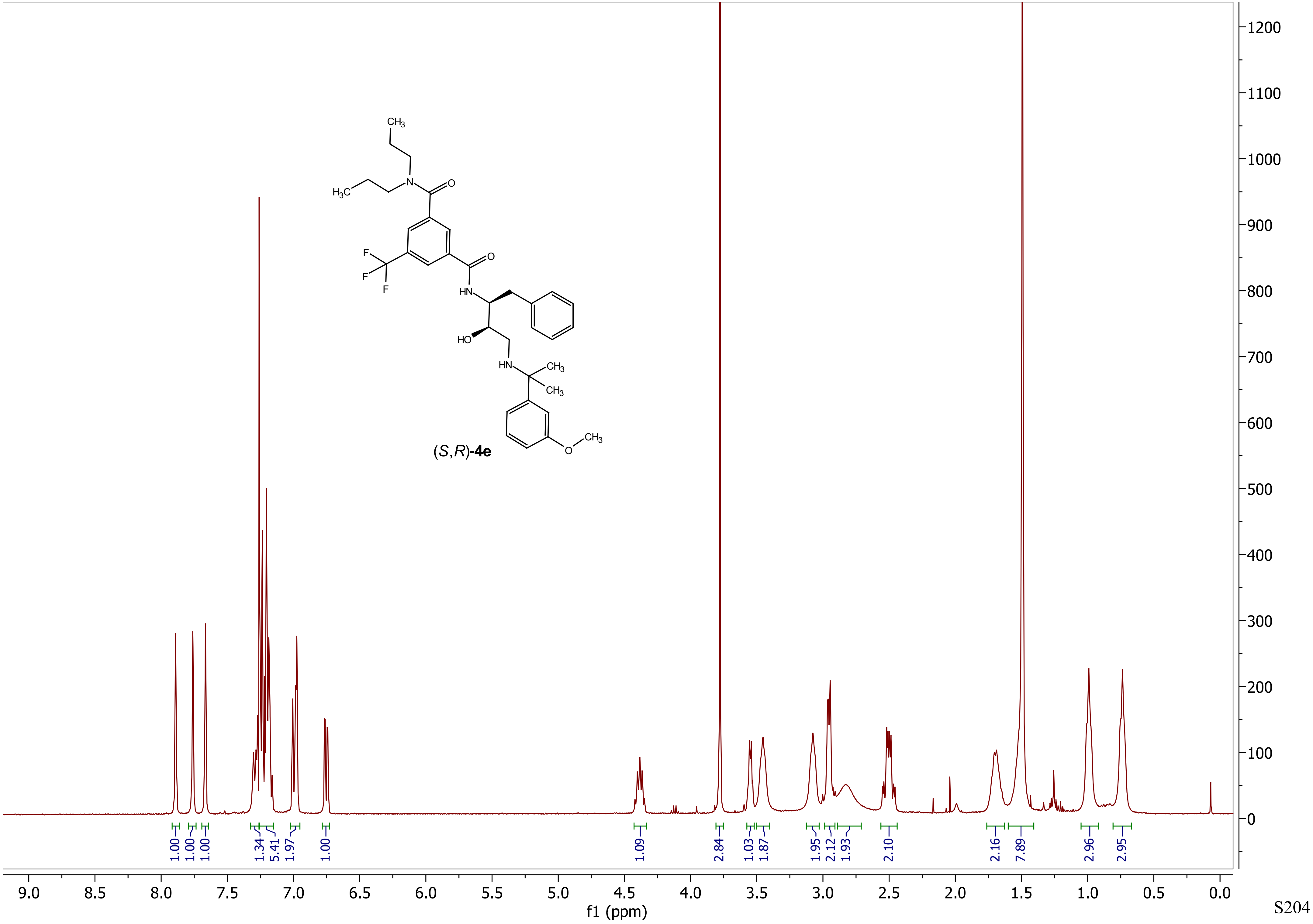

CH3
H3C
N
O
F
F
F
O
HN
HO
HN
CH3
CH3
O
CH3
(S,R)-4e
1.00
1.00
1.00
1.34
5.41
1.97
1.00
1.09
2.84
1.03
1.87
1.95
2.12
1.93
2.10
2.16
7.89
2.96
2.95
9.0 8.5 8.0 7.5 7.0 6.5 6.0 5.5 5.0 4.5 4.0 3.5 3.0 2.5 2.0 1.5 1.0 0.5 0.0
f1 (ppm)
1200 1100 1000 900 800 700 600 500 400 300 200 100 0

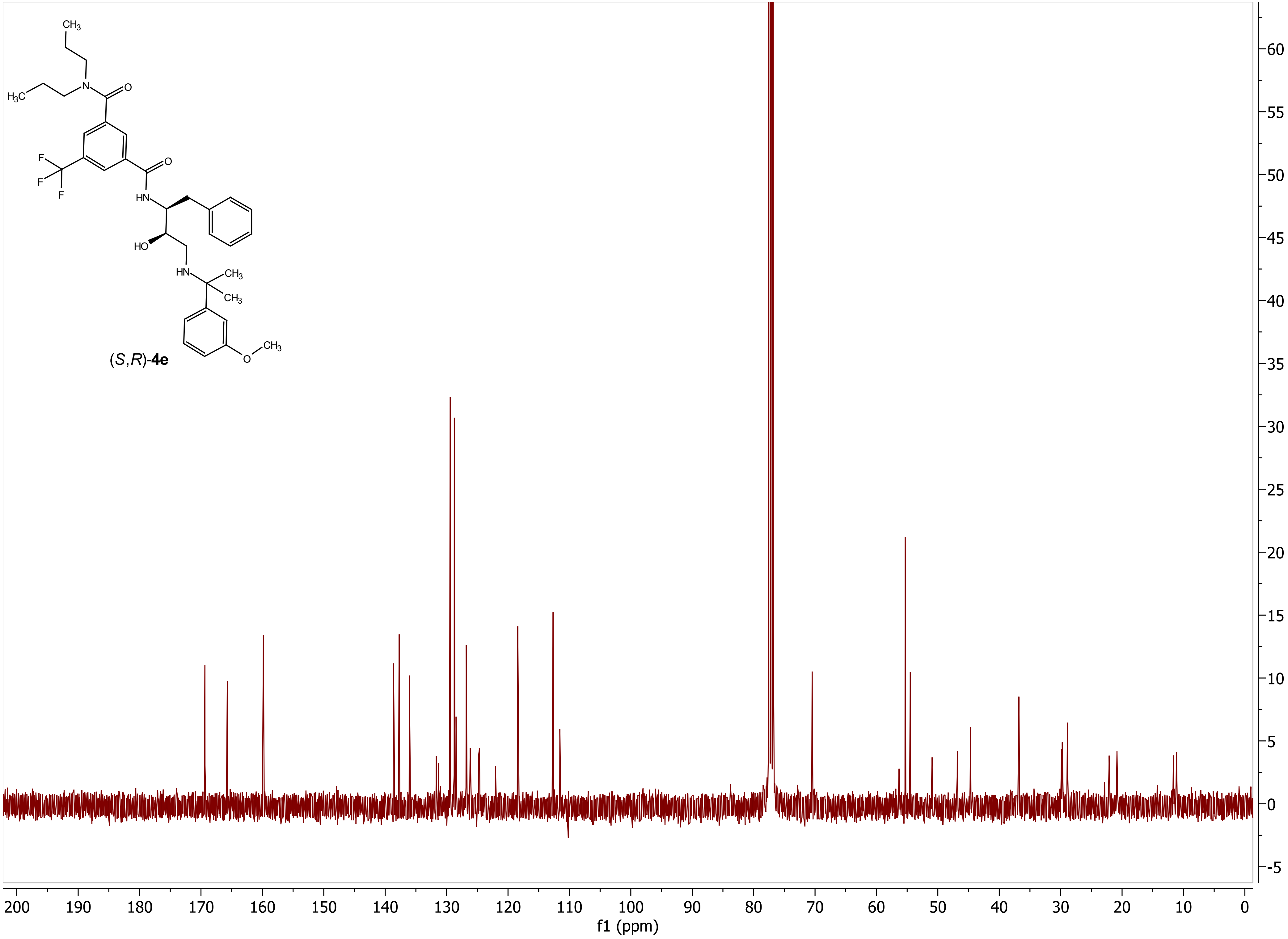
CH3
H3C
N
O
F
F
F
O
HN
HO
HN
CH3
CH3
O
CH3
(S,R)-4e
f1 (ppm)
200
190
180
170
160
150
140
130
120
110
100
90
80
70
60
50
40
30
20
10
0
60
55
50
45
40
35
30
25
20
15
10
5
0
-5

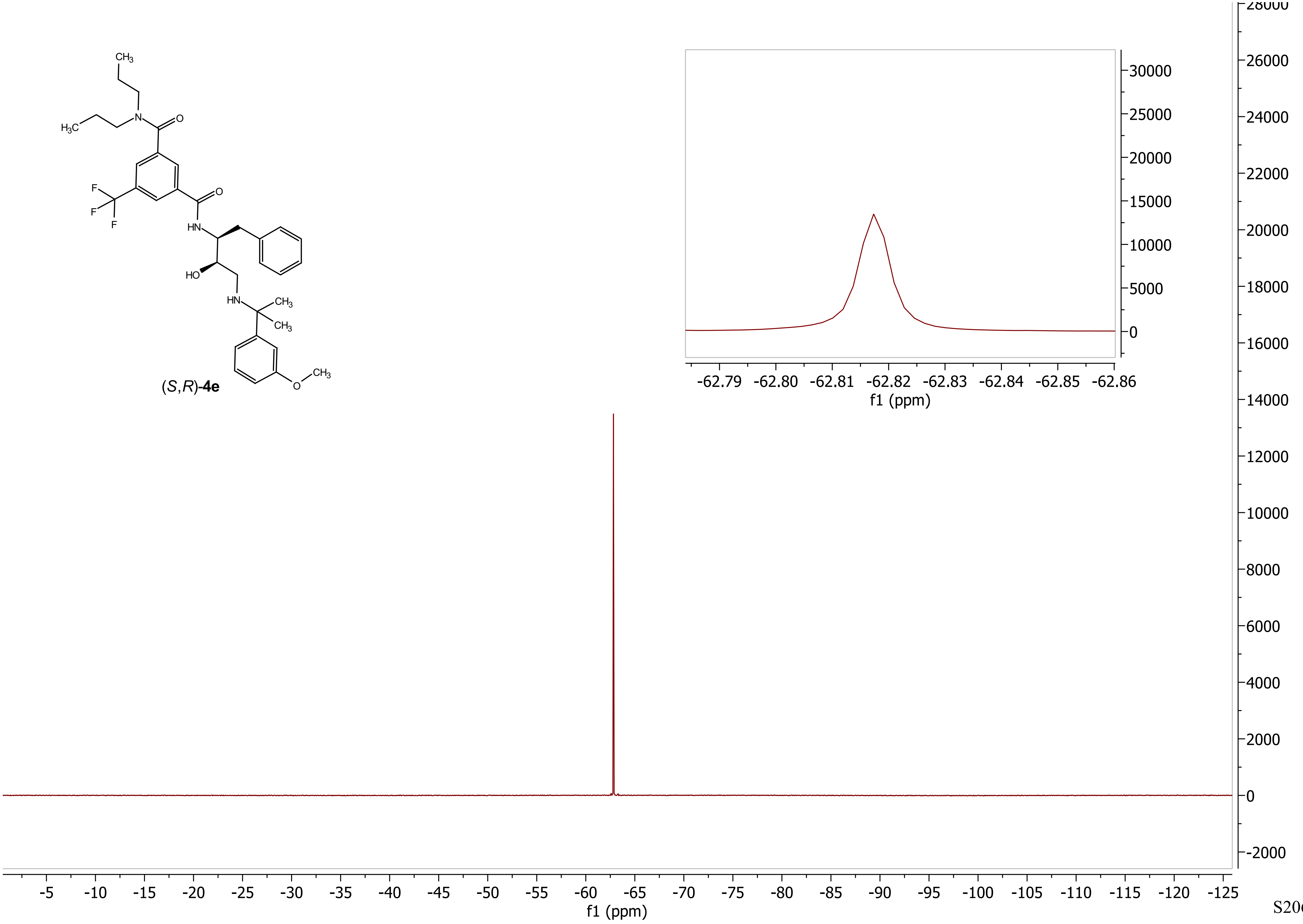
CH3
H3C
N
O
F
F
F
O
HN
HO
HN
CH3
CH3
O
CH3
-62.79 -62.80 -62.81 -62.82 -62.83 -62.84 -62.85 -62.86
f1 (ppm)
30000
25000
20000
15000
10000
5000
0
28000
26000
24000
22000
20000
18000
16000
14000
12000
10000
8000
6000
4000
2000
0
-2000
-5 -10 -15 -20 -25 -30 -35 -40 -45 -50 -55 -60 -65 -70 -75 -80 -85 -90 -95 -100 -105 -110 -115 -120 -125
f1 (ppm)

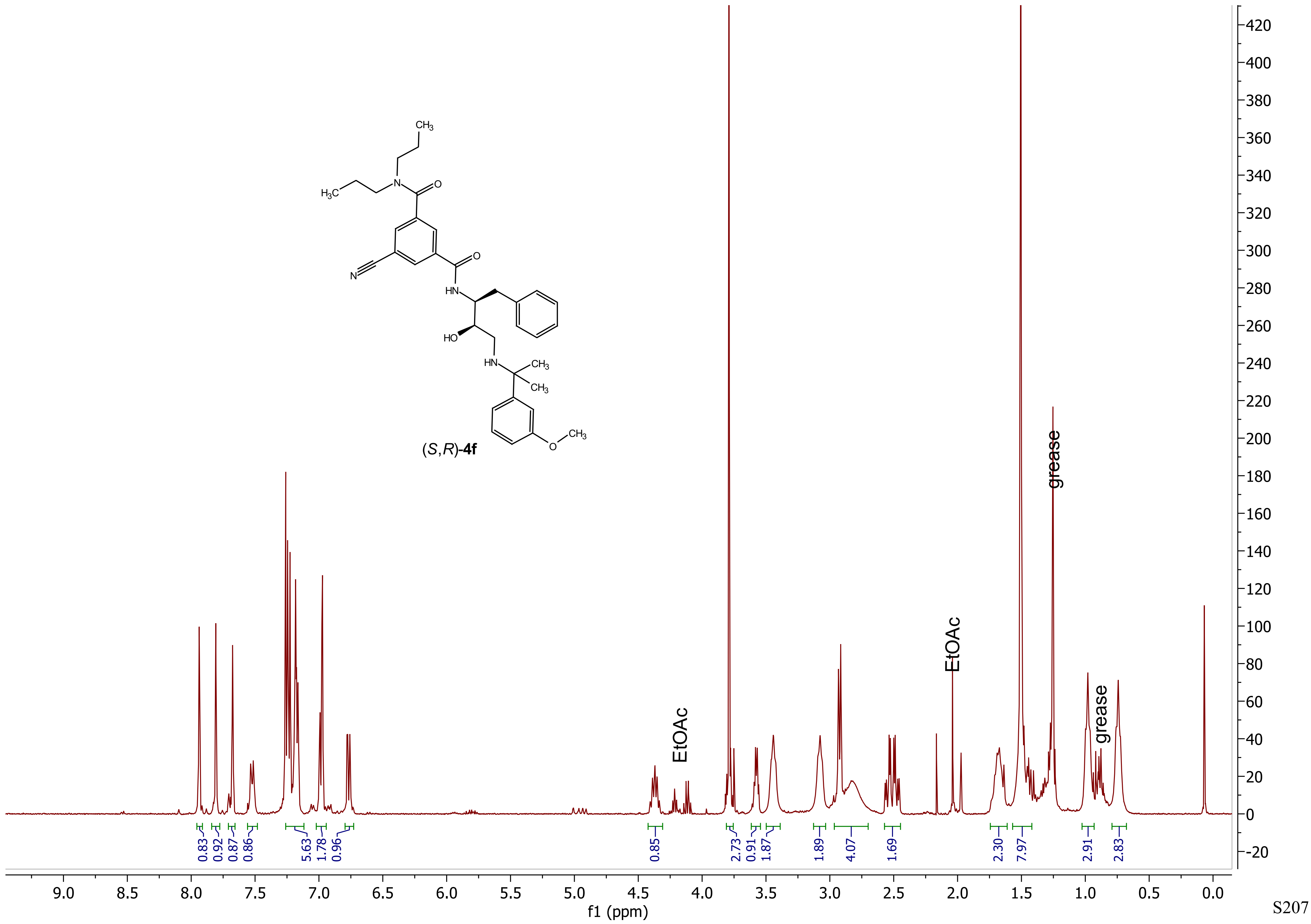

(S,R)-4f
EtOAc
EtOAc
grease
grease
f1 (ppm)

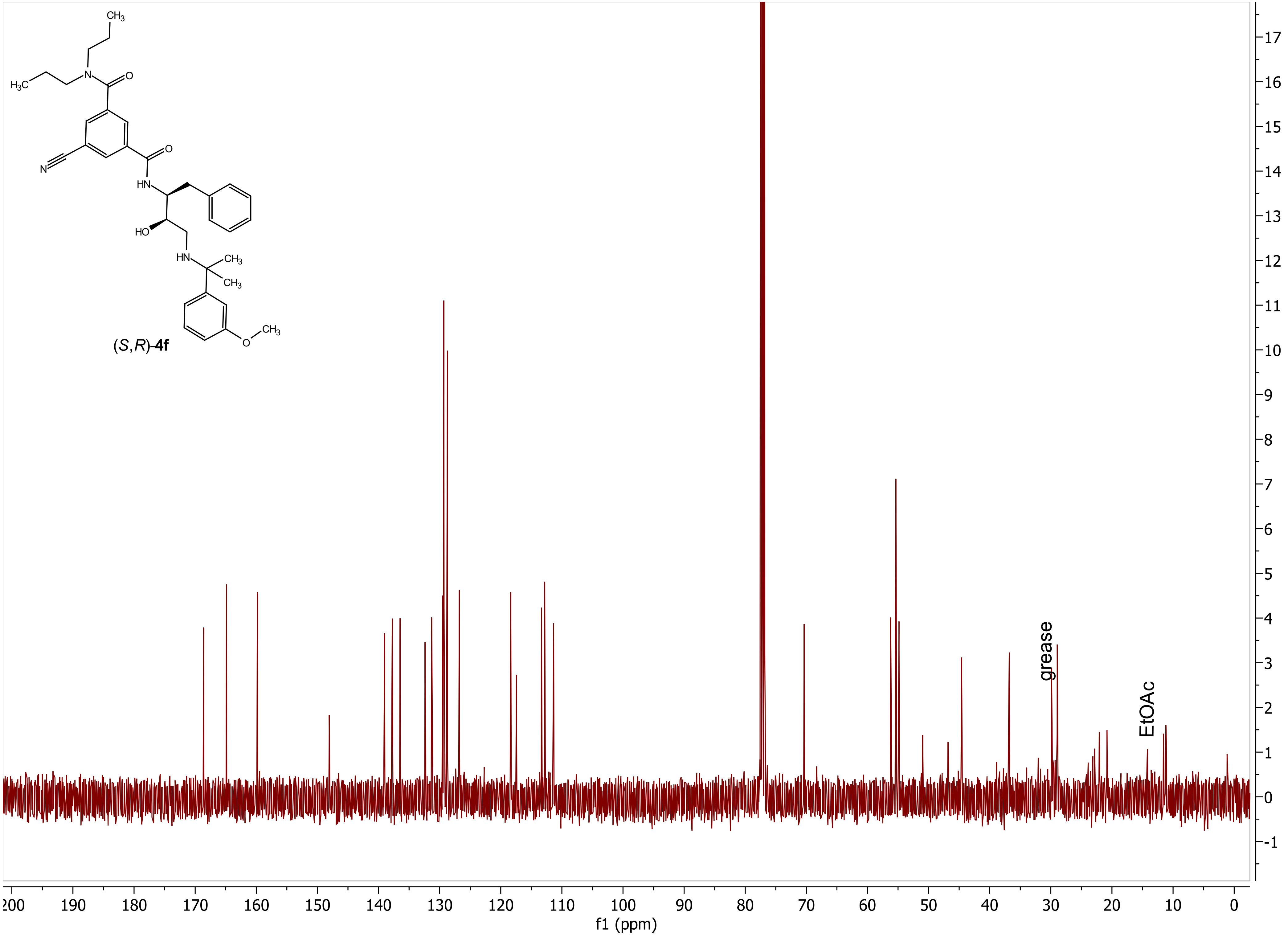
(S,R)-4f
grease
EtOAc
f1 (ppm)

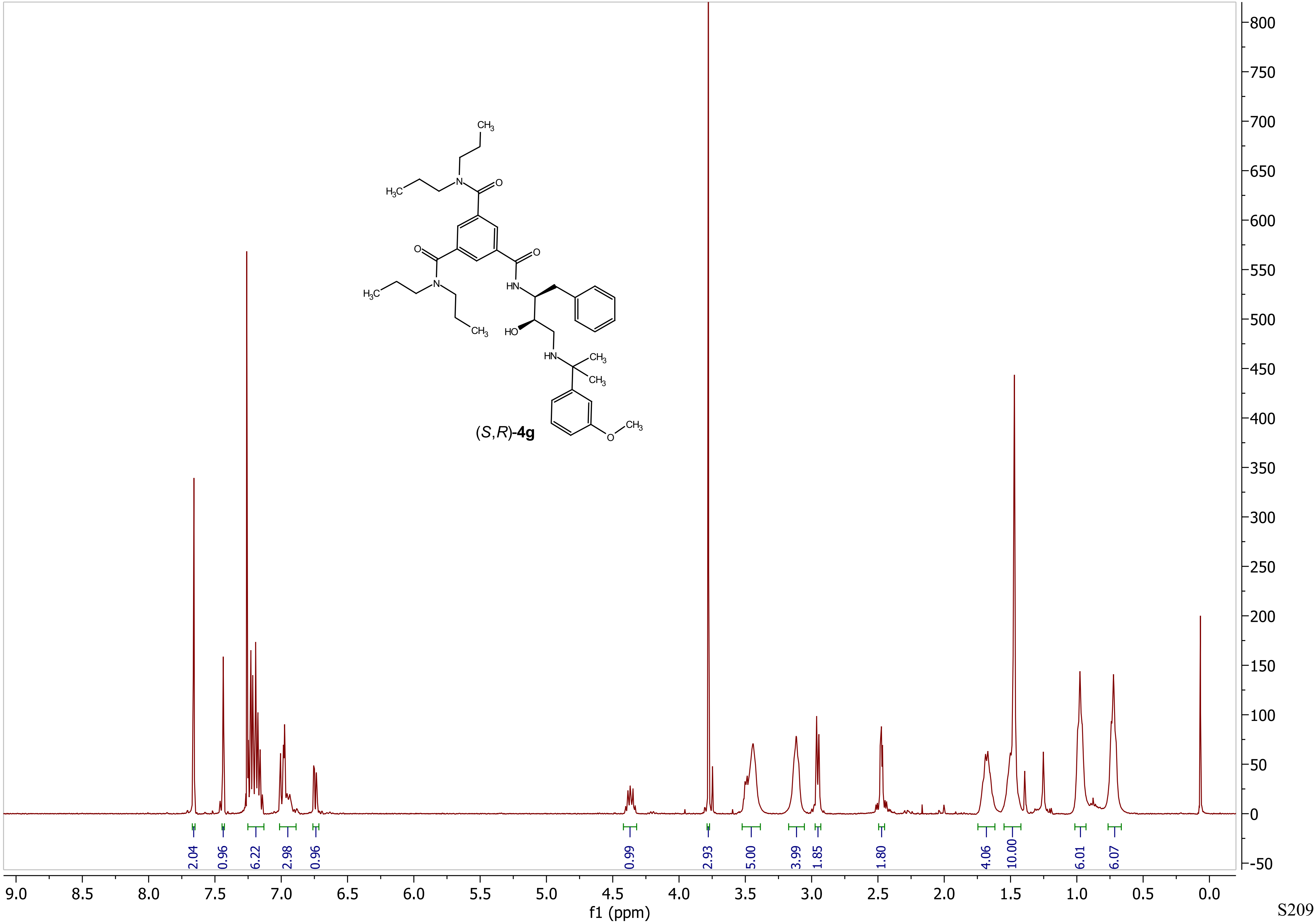

(S,R)-4g
2.04
0.96
6.22
2.98
0.96
0.99
2.93
5.00
3.99
1.85
1.80
4.06
10.00
6.01
6.07
f1 (ppm)

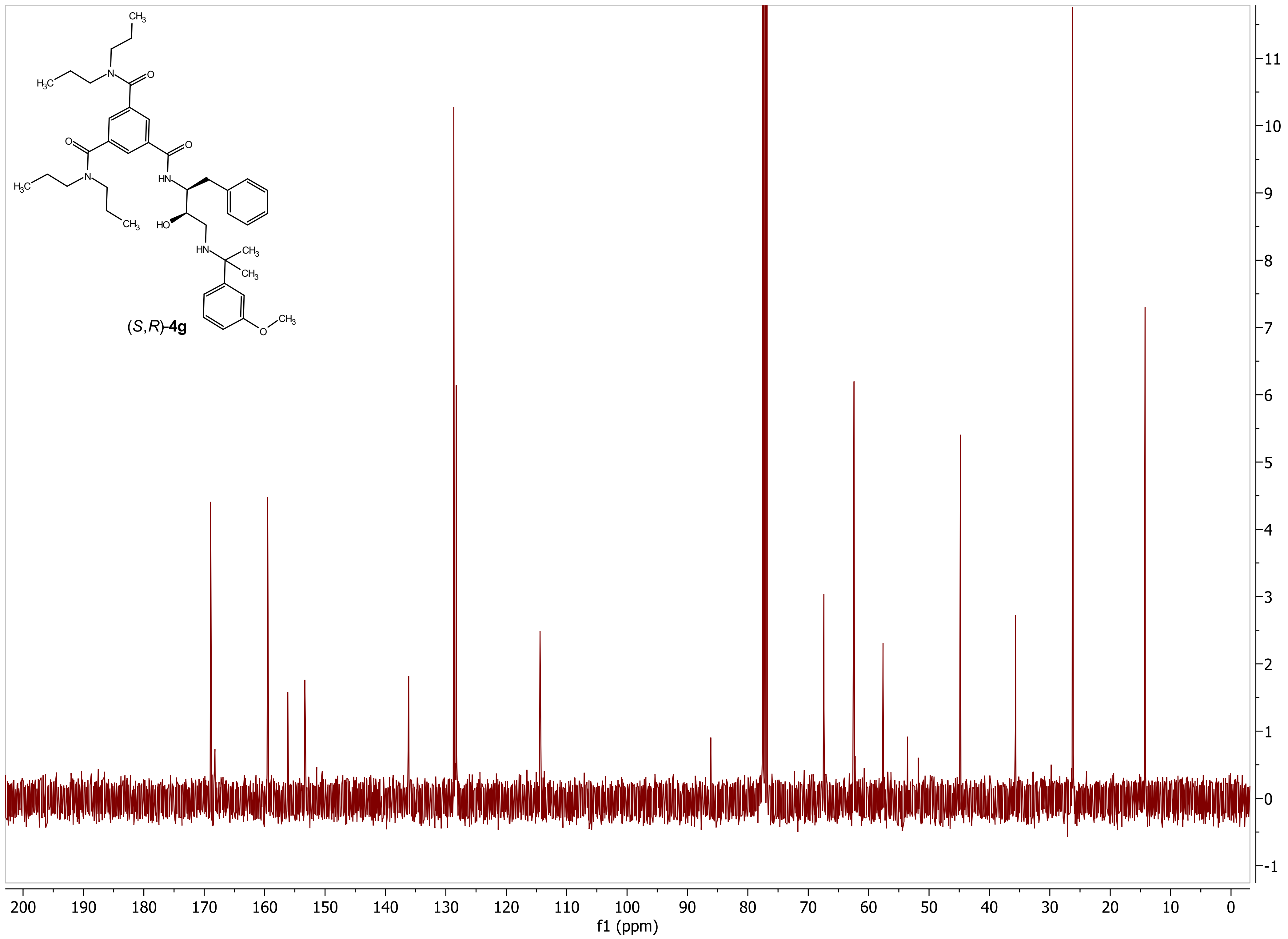
(S,R)-4g
f1 (ppm)
200
190
180
170
160
150
140
130
120
110
100
90
80
70
60
50
40
30
20
10
0
11
10
9
8
7
6
5
4
3
2
1
0
-1

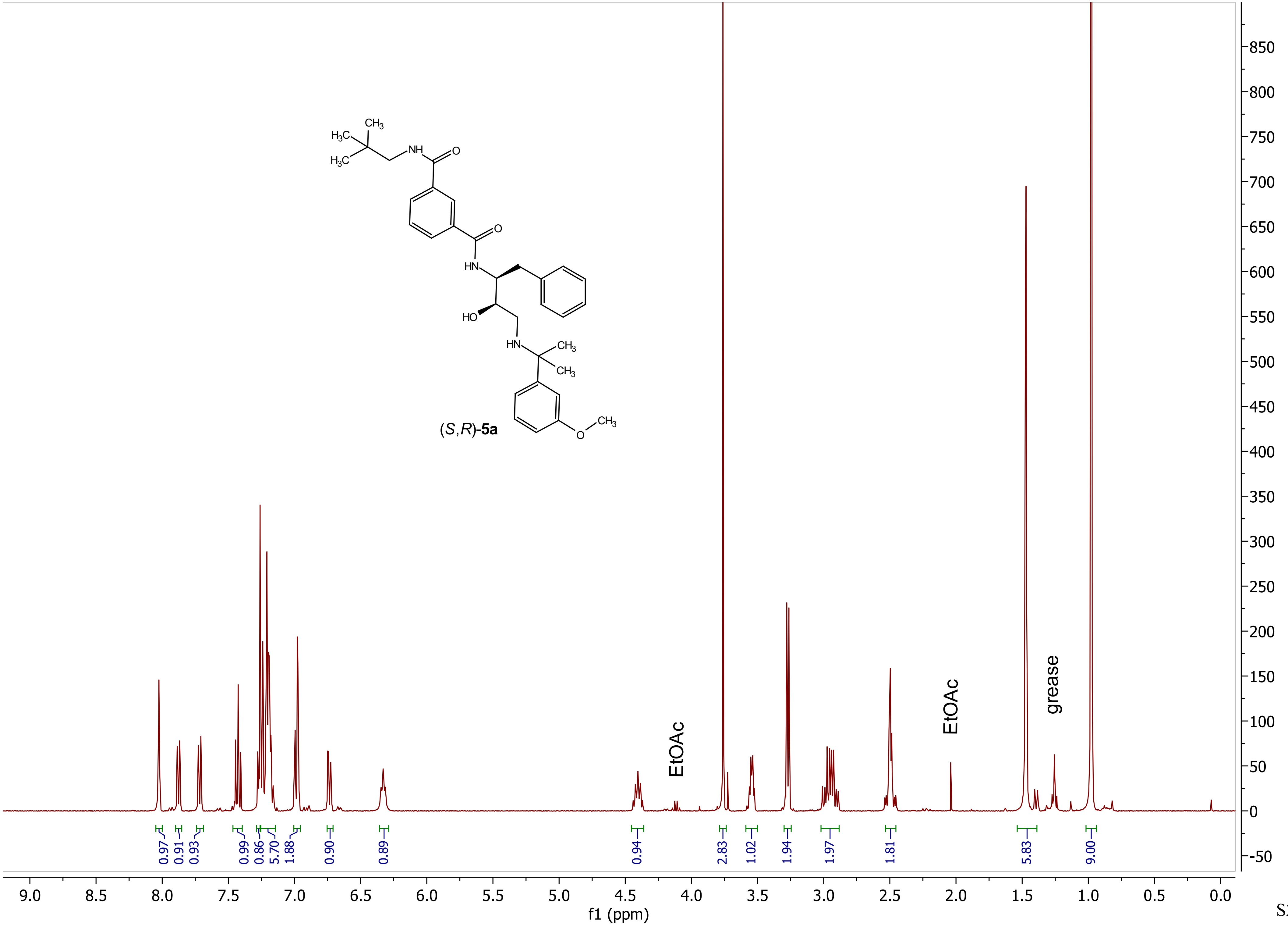

(S,R)-5a
EtOAc
EtOAc
grease
0.97
0.91
0.93
0.99
0.86
5.70
1.88
0.90
0.89
0.94
2.83
1.02
1.94
1.97
1.81
5.83
9.00
f1 (ppm)

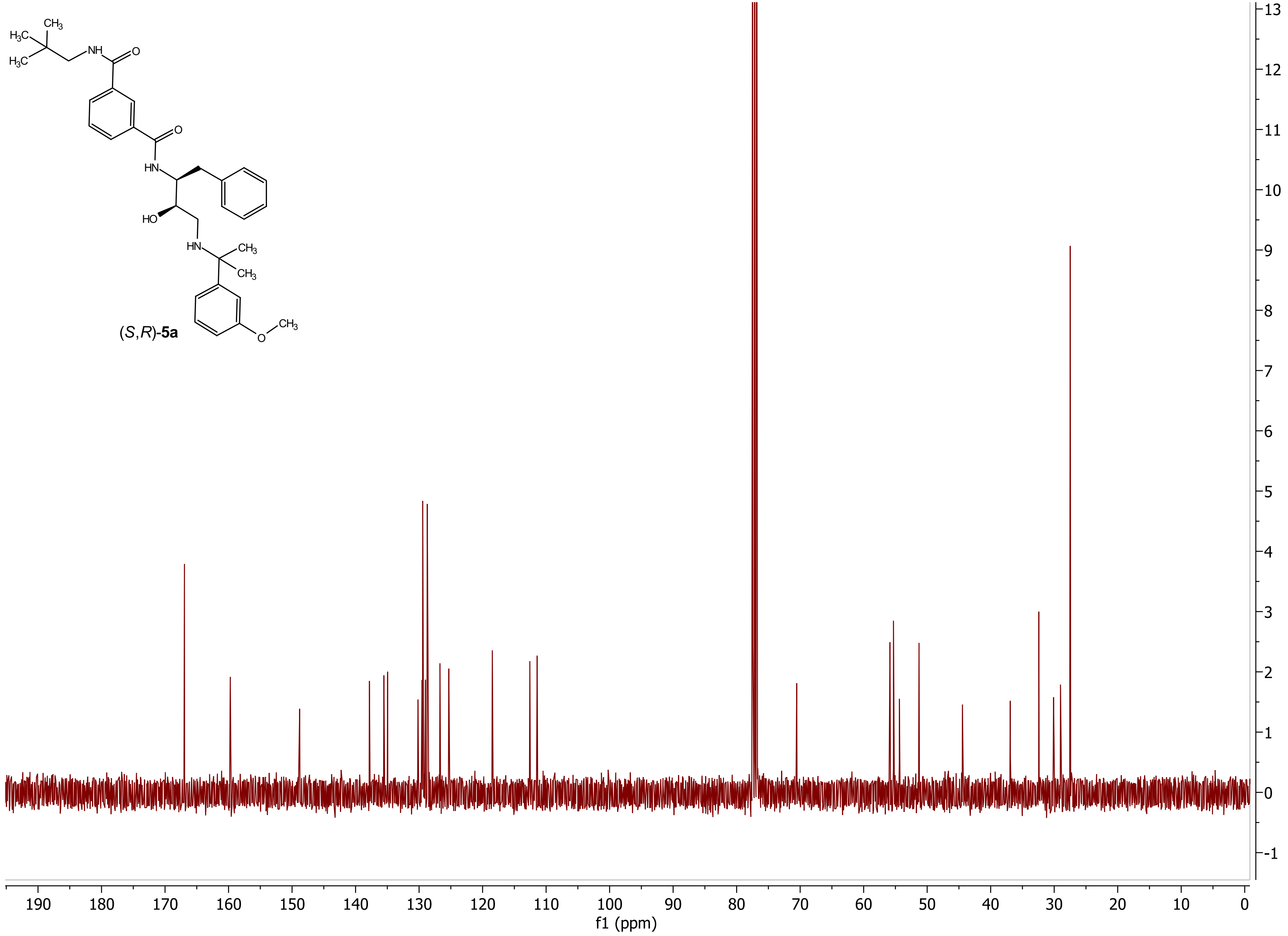

CH3
H3C
H3C
NH
O
O
HN
HO
HN
CH3
CH3
O
CH3
(S,R)-5a
13
12
11
10
9
8
7
6
5
4
3
2
1
0
-1
190
180
170
160
150
140
130
120
110
100
90
80
70
60
50
40
30
20
10
0
f1 (ppm)

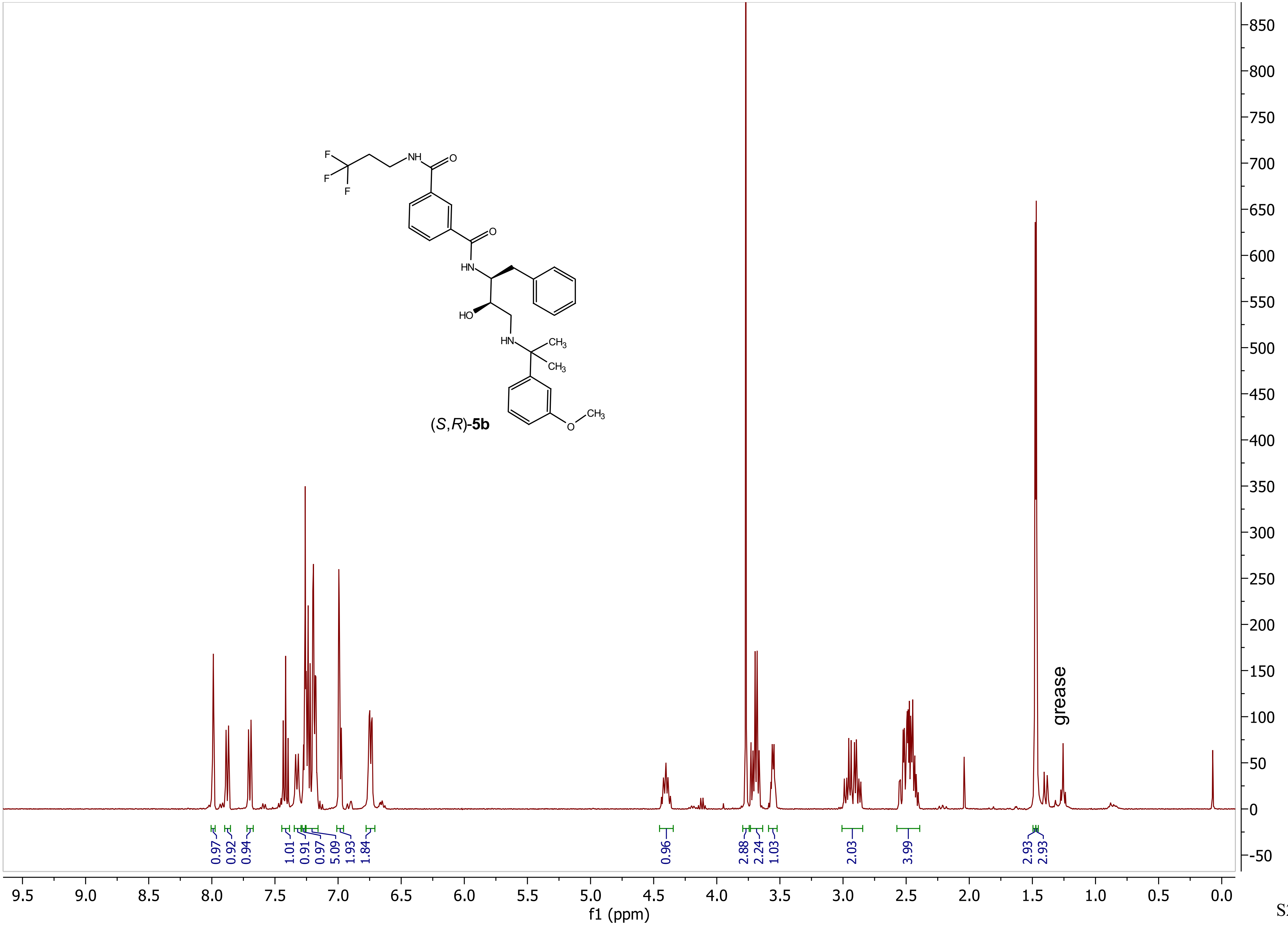

F
F
F
NH
O
O
HN
HO
HN
CH3
CH3
O
CH3
(S,R)-5b
grease
0.97
0.92
0.94
1.01
0.91
0.97
5.09
1.93
1.84
0.96
2.88
2.24
1.03
2.03
3.99
2.93
2.93
9.5
9.0
8.5
8.0
7.5
7.0
6.5
6.0
5.5
5.0
4.5
4.0
3.5
3.0
2.5
2.0
1.5
1.0
0.5
0.0
f1 (ppm)
850
800
750
700
650
600
550
500
450
400
350
300
250
200
150
100
50
0
-50

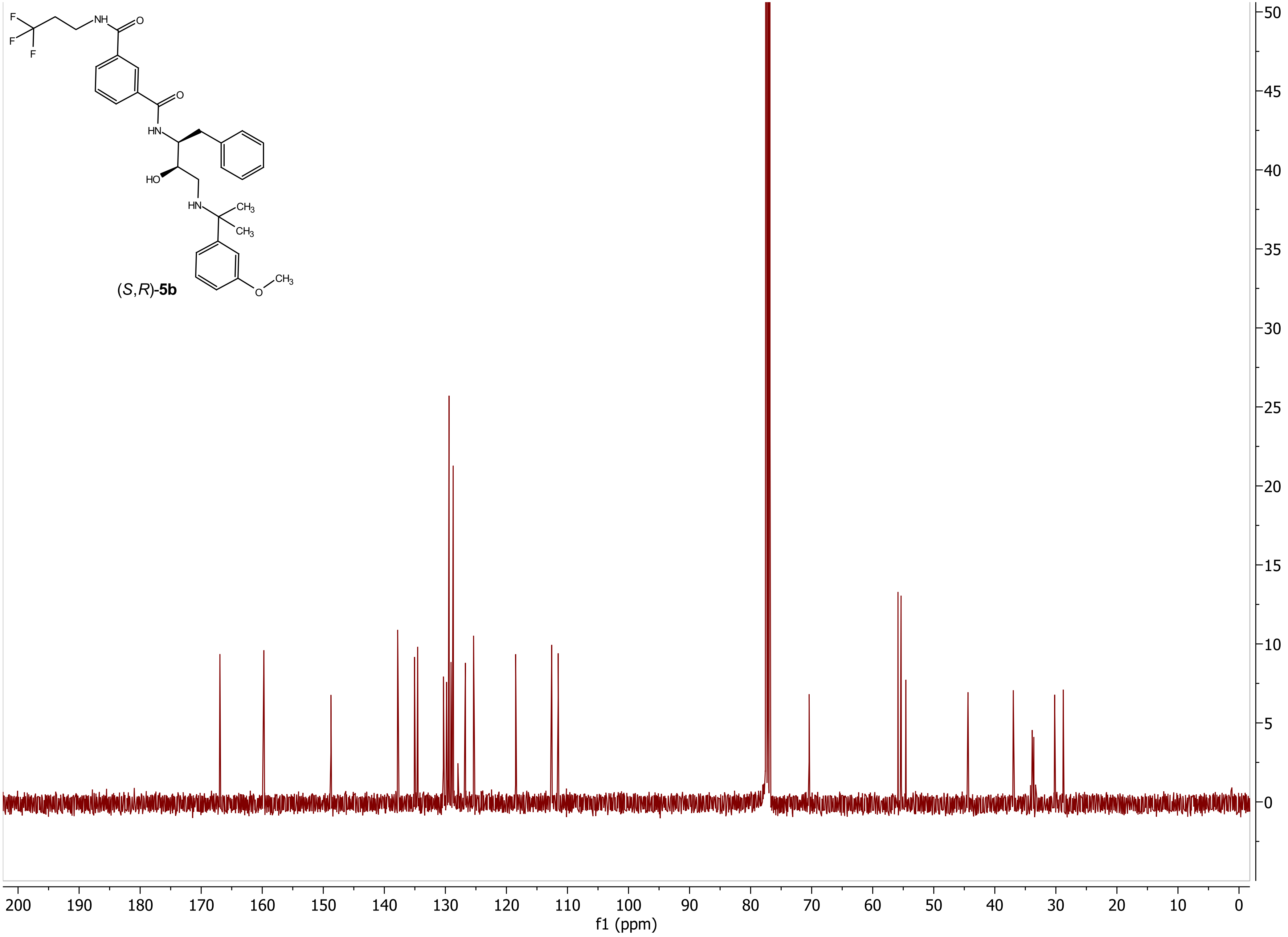
(S,R)-5b
f1 (ppm)
200 190 180 170 160 150 140 130 120 110 100 90 80 70 60 50 40 30 20 10 0
50 45 40 35 30 25 20 15 10 5 0
